# Framing effect method in vaccination status discrimination research

**DOI:** 10.1057/s41599-022-01299-x

**Published:** 2022-08-18

**Authors:** Lyubov Gurevich

**Affiliations:** Moscow State Linguistic University, Moscow, Russia

**Keywords:** Language and linguistics, Social policy

## Abstract

According to Chong and Druckman (J Commun 57:99–118 2007), the “framing effect” is a phenomenon that occurs when even small changes in an issue’s representation cause large changes in people’s opinions. Being a cognitive bias, the framing effect encourages people to disregard their own viewpoints and decide in favour of options, represented in surveys (or articles) with positive or negative connotations. It leads to misrepresentation of findings, which contravenes the accuracy of scientific research. This paper intends to analyse the frame “Discrimination” in relation to the cases of public health (namely vaccination status discrimination) in order to find out which frame dimensions and options can influence people’s opinions on these issues, and to which extent these perspectives can be liable to changes due to the framing effect method. The analysis of social discrimination cases demonstrates how different manipulative technologies form a negative opinion towards the out-group members in public.

## Introduction

It is common knowledge that the world community tends to develop and implement different social management technologies, which will govern social behaviour and establish order.

The framing effect, as a “cognitive bias where people decide on options <…> with positive or negative connotations”, has been widely used as a research subject in psychology (Kahneman and Tversky, 1984), economics (Kahneman and Tversky, 1979), cognitive linguistics (Lakoff, 2004), cognitive psychology (Schank and Abelson, 1977; Whitney, 2001), communication theory (the communicative strategies approach) (Sheigal, 2000), and sociology (Goffman, 1974; Verhoeven, 1985; Scheufele and Tewksbury, 2007, pp. 9–20).

According to Chong and Druckman, the early studies of mass public opinion, conducted in the 1950s and 1960s, discovered that many “citizens have been found to have a low-quality opinion or not to have any opinion at all” (Converse, 1964; Zaller, 1992, cited in Chong and Druckman, 2007, p. 103). Presumably, this is one of the main reasons why people’s opinion manipulation can become an option for social control. Every unstable and flexible social phenomenon is susceptible to framing and reframing under the influence of certain powers. As for mass public opinion, the powerful instrument of its manipulation is information (or sometimes disinformation) pushed through mass media communication. Public opinion is highly dependent on the sight angle presented in sociological questionnaires or op-ed articles, and the way of informing people about something often plays a crucial role in popular verdict formation. On the other hand, many non-standard situations, where people’s health and lives are entirely dependent upon their societal attitudes and viewpoints, have not yet been researched. It is not sufficiently clear whether society can readily yield to somebody’s influence, persuasion, or even threats in this particular situation. The potential for framing technology efficacy has not been defined either. Presumably, the world community has not faced a serious global danger of this kind before, when the choice between life and death becomes highly sensitive to the consensus.

The present article focuses on the analysis of the issue of discrimination in terms of framing effect technologies, using the example of vaccination status discrimination (a part of the “Public health” frame and of its subframe “Vaccination campaign”). This type of discrimination is new and has not yet been defined and researched, although it has given rise to a spirited discussion in society.

The article provides a benchmarking study of different types of discrimination and singles out the distinctive features of vaccination status discrimination (VSD) whilst also describing how government officials are trying to use framing effect technologies in order to calm people’s wrath and to take control of people’s minds.

The VSD analysis is based on the investigation of three basic types of discourse: the medical experts’ discourse, the political officials’ discourse, and the public social media discourse. The research discovers an interesting observation: everything that is initially considered to appear spontaneously in a public discourse turns out to be controlled and directed by power holders. Three types of discourse are interrelated, directed, and controlled within the framework of a person’s consciousness manipulation technology, which is a framing effect method.

## Theoretical background

This investigation is based on a relatively new methodology of the framing effect analysis (Brewer and Sigelman, 2002; Dewitt, 2007; Chong and Druckman, 2007; Kaufman et al., 2003, etc.), critical discourse analysis (van Dijk, 2004), content analysis (Bryman, 2012), frame theory in linguistics (Fillmore and Baker, 2001), logical analysis as a qualitative method (Williams, 1981), the cognitive approach in psychology (Schank and Abelson, 1977; Kahneman and Tversky, 1979, 1984; Whitney, 2001; Pinker, 2003), cognitive linguistic analysis (the cognitive space theory (Gurevich, 2009); the communicative strategies approach (Sheigal, 2000)), and others.

According to the definition, sociological framing is based on the description of the social construction of a social phenomenon. The actors of social discourse included in framing are mass media sources, political leaders, social or political movements’ leaders, and other actors and organisations.

In comparison with the political discourse, which “consists of the language and visuals that people use to promote their interests within the political sphere” (Coe, 2016), social framing is not that contested and “might evolve imperceptibly and organically over cultural time frames, with fewer overt of disputation”. Framing is aimed at social discourse construction, and additionally includes the “individual’s perception of the meaning attributed to words and phrases” (Framing (social sciences): https://en.wikipedia.org/wiki/Framing_(social_sciences)).

Depending on the audience and type of presented information, framing in communication may be positive or negative. Two basic forms of framing are the equivalence frame (with logically equivalent alternatives) and the emphasis frame (described as “focusing on a subset of relevant aspects and issues”) (Druckman, 2001, pp. 225–256, cited in Framing (social sciences), 2021). The most important feature of this method is that “the information being presented is based on the same facts, but the “frame” in which it is presented changes, thus creating a reference-dependent perception” (Druckman, 2001). It is common knowledge that any given situation can be perceived by different people in different ways. Metaphorically, they may look at it through the lens of “the glass is half-full” (which is positive thinking) or “the glass is half-empty” (which is negative thinking) judgements. The probability of contradictory discourse representation of the same facts in a favourable light of positive thinking (see, for details, Wertheimer, 1959, 1996), and without falling back upon lies, makes this method rather useful in political and social contexts.

Almost all sociological research studies have to contend with biased information leading to contradictory data that is highly dependent on the angle of sight presented in a sociological enquiry, public discussion or an article. Thus, of prior importance is how, and with what connotation, this information is conveyed, and what is meant but not expressed in words. Chong and Druckman analysed this phenomenon using the example of entitlements in the USA. When asked about social benefits being paid out to the population, ~20% of the respondents stated that too little was being spent on welfare, although 65% of those surveyed believed that too little was being spent “on assistance to the poor” (Rasinski, 1989, p. 391, cited in Chong and Druckman, 2007, p. 104). This is indicative of how “the alternative phrasing of the same basic issue significantly alters its meaning to respondents, even when the change in connotation is not immediately identifiable by the researcher” (Zaller, 1992, p. 34, cited in Chong and Druckman, 2007, p. 104).

Linguistically, this phenomenon is associated with the existence of some core elements of the above-mentioned alternative phrasings, the immediate emphasis on which can drastically change people’s opinions on the same problematic issue. In Schank and Abelson’s terms, the phenomenon relates to two classes of knowledge, which people bring to bear during the understanding process: general knowledge, with their core elements for understanding, and specific knowledge (Schank and Abelson, 1977, p. 37). The main idea of this approach can help us to understand why people do not follow a stereotyped schema in their understanding of a familiar, but to a certain extent new, situation. “So, people might use a relevant schema whenever possible, but when no relevant schema is triggered another set of processes are initiated that are responsible for constructing new schemata” (Whitney, 2001, p. 13525). The aforementioned process becomes unpredictable from the stereotyped thinking perspective. This specificity of an individual’s thinking plays a crucial role in the manipulative capacity of the framing effect method, which will be analysed further in what follows.

It is important to note that semantic frames are fundamentally different from sociolinguistic frames in this respect. The main group elements in the semantic frame are the set of abstract, ideal elements (labels), which are the basis of the mother frame, constructing the core of the concept they represent (Fillmore and Baker, 2001). They are relatively stable and are an integral part of the ideal construction of a prototypical situation. As compared with the semantic frame, the sociolinguistic frame is flexible, and the status of its basic elements is highly dependent on the applied situation. If we refer back to the entitlement frame, it becomes clear how the emphasis altering from one basic element (“welfare”) to another (“assistance to the poor”) changes people’s perception of relatively the same situation as a whole. This is well explained by Joncas’s study, who “yielded three clear dimensions: positive–negative, intimate–distant, and dominant–submissive” as life themes parameters (Schank and Abelson, 1977, p. 142). He analysed multiple situations in which interpersonal themes are described “by certain default values on the scales (i.e. values assumed in the absence of better information)” (Schank and Abelson, 1977).

Social (or sociolinguistic) frames’ dependence on the applied situation makes it possible for the speaker to change the emphasis in communication in order to achieve a desirable effect. When the person places an ideal case in a positively valued frame, he/she obtains its positive perception via his/her interlocutor, and vice versa. The taxation frame, described in the works of different scholars (Senin, 2009), for instance, demonstrates how people’s understanding of equitable income distribution changes due to the dominant position of different frame elements, such as *taxes*, *equality* and *income*. These frame group elements create or strengthen biased associations, which make the same people change their viewpoint from positive to negative and in reverse order. The mechanism of human information perception instability works in this manner.

The content analysis provides us with the necessary data, which helps us to structure the “Discrimination” frame and to determine basic frame group elements, as well as to discover which elements occupy a dominant position in the vaccination status discrimination domain and to distinguish specific characteristics of this type of discrimination.

Finally, the analysis of different situations of people’s discrimination regarding other people’s health issues reveals where the dominant frame group element can strengthen or weaken positive or negative attitudes of people on the same point.

## A person’s consciousness framing possibility background

Structurally, the description of the framing methodology consists of a set of theoretical perspectives and concepts, analysing how individuals, groups and societies perceive and organise reality and how they communicate about it (Druckman, 2001, pp. 225–256). They tend to use both verbal and non-verbal elements of communication, such as words and phrases (as verbal elements), images and presentation styles (as non-verbal elements), to relay information to their communicational partners. The scholars focus on different “mental filters” (which are of a biological or cultural nature) to examine how social norms and values, interest groups’ pressure, and organisational pressures and constraints influence media content frames (Scheufele, 2000, pp. 297–316). The other highly important aspect is analysed by Schank and Abelson, and concerns the people’s perception, inference and prediction of different stereotyped and new situations; this aspect gives clues as to the understanding of people’s reaction to manipulative technologies used for reframing people’s minds (Schank and Abelson, 1977).

On the other hand, news media can distort information by trivialising or over-complicating the analysed social phenomena. This information manipulation becomes possible due to the complexity of the process of communication, which includes a set of the same complicated actions, such as cognition, interpretation, selection, presentation and emphasis, which often remain unspoken and unacknowledged (Gitlin, 1980). An individual tends to inadvertently focus on and memorise those details, which are vitally important or especially interesting for the person at that moment (Pinker, 2003, p. 397). The person subconsciously filters out the incoming information through his/her individual cognitive space. This filtered-out information leaves a fragmentary represented imprint of the perceived object in the person’s memory (Gurevich, 2009).

According to Meadows, “… each individual has his/her proper cognitive space, i.e. a perceptive capacity. The dimensions of this cognitive space depend on information, training and, finally, on a person’s awareness. All this depends globally on the cultural setting” (Meadows et al., 1972, p. 19). In general terms, there can be no identical perception of the reality by different individuals, just as there is no identical reflection of it in their minds (for details, see, e.g., Capaldi, 2001, p. 1; Gurevich, 2009, p. 70).

The incoming information is identified and put through the existing mental models (or frames) in an individual’s mind (Bukhanovsky et al., 2000; Shpar et al., 2020; Meditsinskaya entsiklopedia, 2006, etc.). Every case of information obtained is accompanied by mental reframing (restructuring) which is going on subconsciously. As Whitney argued, “people might use a relevant schema whenever possible, but when no relevant schema is triggered another set of processes are initiated that are responsible for constructing new schemata” (Whitney, 2001, p. 13524). The other peculiarity of the person’s mental models’ (patterns) functioning is their fragmentary representation when being retrieved from the memory. This explains why cognitive processes are not similar for every particular case.

According to Goodman, similarity does not exist in reality; it exists in a person’s mind. Taking as an example a situation at the bag drop counter at the airport; the viewer will notice such luggage characteristics as shape, size, colour, fabric, and even brand; the pilot will be focused on the luggage weight; the passenger will be anxious about its ownership and destination. The similarity of the luggage units depends *not necessarily on their common objective characteristics*, *but on those people’s minds, who are comparing them, and on the situation itself in general* (cited in Pinker, 2003, p. 397). To summarise, people’s cognition (i.e. perception, memorising, framing and reframing, etc.) is fragmentary and asymmetric, which often leads to misunderstanding or misconception in communication. This is another actual objective reason why sociolinguistic frames are flexible and unstable. The necessity of being compatible in communication makes conditions for individual cognitive spaces (and frames as integral parts of their structures) fall into sync with each other and with the communicative situation on the whole. Thus, the mental structures of communicators exist and function in permanent processes of adjustment and change. In Schank and Abelson’s terms, it is some sort of “default values” processing in a new schema construction (Schank and Abelson, 1977, p. 142). It is important to note that all of these processes are going on subconsciously; it is doubtful that the person could purposely intend to restructure his/her existing mental patterns. What is more, a person is most likely to resist reframing these patterns, and some people possess a low capacity for reframing in general or in certain circumstances, or under the influence of certain factors. This peculiarity can be explained by the fact that “a man is programmed to resist changes: it is the key to his survival and, simultaneously, an obstacle in the path of full implementation of his potential” (Kolesnikov, 2020).

All of the above-mentioned examples are suggestive of a contradictory deduction: on the one hand, a person’s mind is flexible and ready for framing and reframing in every new communicative situation he/she encounters. On the other hand, an individual tends to subconsciously resist any change in his/her state of mind. Being contradictory by nature, this peculiarity provides a basis for the person’s consciousness manipulation and simultaneously becomes a serious obstacle in the process of convincing a person of something which contradicts his/her beliefs. The psychologists argue that *the framing technology can be highly effective when a person is unaware of the issue, and his (her) opinion has not been formed yet*. “Because of imperfections of human perception and decision-making, <…>, changes of perspective often reverse the relative apparent size of objects and the relative desirability of options” (Tversky and Kahneman, 1981, p. 453).

Consequently, the logically sound questions are: To what extent is an individual’s mind susceptible to change under the pressure of somebody’s dominating rhetoric, including discriminatory practice? What is peculiar about vaccination status discrimination and how do people react to it? What is discrimination and how is it performed in contemporary society?

## Discrimination as an object of sociological research

According to a most abstract definition, discrimination is a “treatment or consideration of or making a distinction in favour of or against, a person or thing based on the group, class, or category to which that person or thing belongs rather than on individual merit” (Discrimination, 2021). It has been one of society’s root problems throughout the history of humankind for centuries. Despite the fact that this highly negative social phenomenon causes resentment in society, it is still widely spread nowadays, and new forms of discrimination will continue to emerge for centuries.

The sociologists argue that the reason for this phenomenon’s existence in society stems from people’s inherency to distinguish between “insiders” and “outsiders”, or “in-group” or “out-group” members (Tajfel, 1970; Tajfel et al., 1971, etc.). Furthermore, people tend to have an affinity for their in-group, so that they feel as though they belong to this group as a member, with contempt for, and desire to compete against, out-group members (In-group Favouritism, 2021). A phenomenon known as *in-group bias* is defined as people’s intention to hold positive attitudes towards members of their own group, whilst demonstrating an opposite attitude towards outsiders. “*In-group Bias* (also known as in-group favouritism) is the tendency for people to give preferential treatment to others who belong to the same group that they do. This bias shows up even when people are put into groups randomly, making group membership effectively meaningless” (Why do we treat our in-group better than we do our out-group? 2022).

This bias, having nothing in common with justice, underlies the relations between people in society. It is the basis of the discrimination phenomenon.

The topics of discrimination, researched by sociologists, are structured around the following key points: (1) definition of discrimination; (2) types of discrimination; (3) reasons; (4) characteristic features; (5) legal sufficiency; (6) focus area; (7) line of argument; (8) target-orientated social group; and (9) the means of discrimination elimination in society, etc. (Tkachenko, 2020; Diskriminatsiya—chto eto?, 2021, and others).

All of the above-mentioned topics present complicated issues that are worth researching. Even if to touch upon such a superficially simple topic as “the definition” of discrimination, one can encounter difficulties with “the scope of empirical inquiry and appropriate methods for identification and study of the phenomenon” which can simultaneously correspond to both theoretical and methodological implications. Discrimination often becomes a judicial matter in legal debates, and thus its definition should contain significant normative implications. The subdivision into intentional and non-intentional discrimination gives rise to a cluster of numerous sub-groups and competing sub-definitions (Pager and Shepherd, 2008; Blank et al., 2004, etc.).

Some researchers distinguish between such related phenomena as racism, nationalism, sexism, ageism, prejudice, and stereotypes, considering discrimination in its narrow meaning and defining it as “a set of behaviours”. They define discrimination as “unjust prejudicial *treatment* of different categories of people”, whilst racism, for instance, is described as the “*belief* that race has an effect on human abilities and traits and that a particular race is superior to other” (Hasa, 2016; italics are mine, L.G.).

The aspect of discriminatory actions is referred to as ideology, attitudes or beliefs, which might, or might not, be intentional discrimination (Allport, 1954). Thus, it is arguable whether or not to include this subject matter in the definition or research of the phenomenon of discrimination (Kohler-Hausmann, 2020).

Therefore, various factors have to be taken into account within the discrimination phenomenon research. The above-mentioned topics of discrimination involve different criteria of research and methods of analysis. The specificity of discrimination is highly dependent on the dominant criterion, according to which society can be conditionally split into two (or more) oppositional groups. For instance, *the religious criterion* is at the root of opposition between Christians and Muslims (or other religions), *the gender criterion* underlies the contradiction between men and women, and *the physical ability criterion* becomes the reason for discrimination against disabled people by healthy members of society, etc. The cognitive structure (a frame, a scene, a scenario, etc.) of this type of discrimination is conditioned by the essence of the problem, intergroup relationship specificity, and the social context in which the people are engaged in. Accordingly, the scenarios of age discrimination at work and religious discrimination differ greatly in many characteristic features. For example, undue hardship, job reassignments, lateral transfers, compromised workplace safety, and other discriminatory actions, can hardly be used against a younger employee; they are specific features of a religious discrimination sample (see, for details, Fact Sheet: Religious Discrimination, 2021).

Discrimination as a social phenomenon can spontaneously appear in society based on any long-term conflict between certain groups of people. The sociologists prefer to define them as “target-oriented social groups”, where the target implies the actual reason for intergroup conflict (Taijfel, 1970). Discrimination of this type supposes subdivision of society into “in-group” and “out-group” members.

## The intergroup discrimination as a social phenomenon

This article is devoted to the analysis of a new type of discrimination, which has recently occurred during the COVID-19 pandemic, namely *vaccination status discrimination*. Thus, it is reasonable to focus on the description of the general characteristic features of discrimination and the intergroup discrimination phenomenon as its theoretical background, which will help us to ascertain the essence of this new type of discrimination.

Intergroup discrimination has no national boundaries and its characteristic features coincide in every part of the world, in every community. Henri Tajfel described these features as “stereotypes which are common traits attributed to a large human group” (Tajfel, 1970, p. 96). He showed how intergroup discrimination, practised by rich people in Yugoslavia against Bosnian immigrants, was perceived by the other nation, namely the British. A group of students at the University of Oxford, who were asked to guess by whom this discrimination was used and to whom it referred, unanimously replied that it was used by native Englishmen and aimed at “coloured” immigrants from India and Pakistan (Tajfel, 1970).

As argued by Coser, intergroup discrimination often develops into two kinds of conflict: the “rational” and the “irrational”. These conflicts and attitudes are a “reflection of genuine competition between groups with divergent interests”. Tajfel was of the view that intergroup aggression is “a by-product of in-group bias”. When an in-group feels that its beliefs and interests are being challenged or threatened, it tends to exert aggression towards the out-group. “Evidence suggests that when social identity is salient, the perceived threat is enhanced and will more likely result in aggressive and retaliatory responses, including ‘vicarious retribution’ against out-group members” (Densley and Peterson, 2017, p. 44). It is important to note that intergroup conflicts serve “to release accumulated emotional tensions of various kinds” (Coser, 1956, cited in Tajfel, 1970, p. 96).

The modes of expression of negative attitudes towards out-group members can vary from one type of discrimination to another, but regardless, they have much in common. This makes it possible to distinguish common features as basic characteristics of discrimination.

The most common features of discrimination (almost all types) are (1) unfounded injustice; (2) ostentatious contempt for other people’s values; (3) sense of superiority; (4) abusive conduct; (5) prejudiced attitude, etc. A detailed description of these characteristic features, and comments on their specificity, will be provided in the analysis of framing effect cases.

## The sociolinguistic frame “COVID-19 vaccination”

It is logical to make a point about the topic of COVID-19 vaccination discrimination within the framework of the vaccination campaign which has recently been launched throughout the world. Its non-standard essence has raised a great deal of speculation, and many rumours, whilst also provoking an unprecedented social response. Vaccination status discrimination is one of such phenomena and had not been registered in society before the COVID-19 pandemic.

The non-standard essence of the world’s recent vaccination campaign has been proved by contradictory, biased rhetoric in society. It is contradictory due to the content presented in the language community, and due to the type of social discourse touching upon this issue.

The vaccination object-matter in social discourse rhetoric falls into at least three categories, which differ by their objectives, wording, and stylistics. The latter includes conversational styles and communicative strategies. These categories are (1) experts’ articles; (2) political discourse rhetoric; (3) social media data. The first category essentially includes medical experts’ works, but this category can also consist of publications from other experts and scientists, who possess special professional knowledge of this issue. The second category includes state officials’ speeches, government rules, administrative orders, and laws. The last category presents social network publications, journalists’ articles, public opinion polls, chat forums, etc.

The vaccination campaign social discourse stems from the experts’ articles, where the dominant position is occupied by the medical experts’ scientific explanation of vaccination procedure and of all the important issues, bound with people’s health. This discourse category tends to provide an impartial evaluation of the situation. It generally lacks emphatic judgements. Regardless, the medical COVID-19 vaccination contexts are contradictory on almost every important issue, due to the novelty of the COVID-19 pandemic itself. There are no accurate records or reliable figures concerning the origin of the virus, whether it is artificial or natural, the possible impact of mass vaccination on the virus’ mutation, viable methods of acquiring immunity to the virus, the probability of achieving herd immunity, and many others.

Presumably, the absence of categorical experts’ explanations of important vaccination matters gives rise to a surge of ambiguous sentiments on this point in the community.

The basic biased societal response mirrors the topics of (a) the mass vaccination appropriateness during the pandemic; (b) the existing vaccines’ general effectiveness; (c) the vaccination’s effect on people’s health (including side-effects and lethality); (d) the pandemic predictions (including vaccination and revaccination, the virus’ mutation, and the infection transmission rates); (e) people’s consistency in terms of getting a vaccine (religion, medical contra-indication, antibodies rate), etc. Many other additional object-matters of social rhetoric can be included in the above-mentioned generalised issues.

The vaccination campaign as the object of social discourse analysis demonstrates one very important feature of the recent pandemic: the disease is totally new and unexampled due to its specificity of spreading and clinical course. This creates a serious problem in every point of dealing with this disease, beginning with its treatment and ending with how to stop this pandemic within a short period, in order to avoid drastic consequences for humankind. Thus, all issues, which have been clear enough within the other vaccination campaigns designed to respond to preventable (via vaccine) diseases, become biased and problematic when they concern COVID-19. The medical experts’ opinions have fallen into two oppositional groups on every one of the above-mentioned important issues, which has laid the foundations for social rhetoric ambiguity and offered the potential for people’s consciousness manipulation, which becomes obvious in political discourse rhetoric.

The main experts’ oppositional viewpoints deal with the vaccination appropriateness during the pandemic object-matter. A group of distinguished microbiologists calls “mass vaccination against the coronavirus during the pandemic “unthinkable” and a historical blunder that is “creating the variants” and leading to deaths from the disease” (Mass vaccination during pandemic historical blunder: Nobel laureate, 2021). The other group of medical experts, whose members are open to the idea of vaccination, admits the probability of the emergence of a new virus strain, “against which the vaccines will be less effective”. Regardless, they insist on people being vaccinated as soon as possible in order to prevent the spread of COVID-19 and the emergence of its new variants. They argue that the virus’ mutation takes time, and this time span (used to mutate) is rather shorter for the virus in vaccinated people, whilst non-vaccinated immune-compromised people need much more time to kill the virus, and thus it has enough time to mutate (Invitro Monitoring, 2021).

The medical experts’ biased argumentation has caused a crisis of distrust and vaccination hesitancy amongst the public. Coincidently, their controversial reasoning has enabled a group of political leaders to use the argumentation for semi-truthful information presentation that is “not fully true, yet not false statements” (Urban Dictionary, 2021). An uncertain medical experts’ discourse has provided a basis for the politicians’ use of a manipulative technology, namely a framing effect method.

According to this analysis, semi-truth is often used as the basis of the framing effect methodology with the purpose of convincing people of seemingly real but actually false ideas and making them change their minds on this biased issue. The necessity of semi-truthful (or false) argumentation is conditioned by people’s distrust in the government authorities’ actions and by their resistance to the world governments’ authoritative measures on the vaccination campaign.

The analysis of the COVID-19 vaccination discourse shows that the semi-truth government officials’ argumentation is based on a contradictory representation of the seemingly hard facts. The basic topics, which are biased by their content, are:

- vaccination safety and reliability;
- the actual reason for the virus’ mutation;
- the expediency of COVID-19 vaccination;
- the existing vaccines’ efficacy;
- the percentage of people who need to be immune in order to achieve herd immunity (60% or 80% of the total population);
- the necessity of revaccination and its protection duration;
- mandatory vaccination initiation;
- COVID-19 vaccination side-effects, etc.

These topics have become urgent in mass media discourse within the last three years. Forming the basis of a semi-truthful representation of the vaccination campaign, the above-mentioned issues have stimulated mass intergroup conflicts, which, on the one hand, have served to release accumulated emotional tension in public, and, on the other hand, have become an operating result of a powerful manipulation tool, which is the backbone of framing effect technology.

## Semi-truth in the framing effect methodology: a case analysis of the vaccination status discrimination

Three basic categories of social discourse have actively intersected in public health communication, and thus it is sometimes difficult to figure out who, when, and why has become the initiator of misrepresentation of the most important data concerning vaccination sentiments.

The medical experts’ assumptions have divided, giving rise to not-settled or controversial opinions amongst the public. This situation has become a breeding ground for the manipulation of people’s opinions by authorities, whose primary duty is to control any ongoing process in society, including pandemics. Being mindful of the fact that, as yet, there is no effective remedy for the new disease, the medical experts recommend achieving herd immunity in order to lower the spread of infection. Thus, the majority of government officials across the world have made a point of achieving herd immunity in communities by any means in order to escape humanitarian disaster; indeed, this could lead not only to a catastrophic decrease in the world’s population but also to votes of non-confidence in these governments. Considering people’s cautious attitude towards this unprecedented phenomenon and the existence of a representative number of people who are unsupportive of the COVID-19 vaccination, the authorities have elaborated on numerous measures to achieve their goals by all manners and means.

The discourse analysis of publications in the USA and several European countries makes it possible to argue that *vaccination status discrimination* (VSD) persists in all communities, varying in its intensiveness and amounting to violence and human rights abuse in certain states.

The phenomenon of the VSD in social discourse is analysed here in terms of “target-oriented social groups”. If we compare this type of discrimination with certain other well-known types, it becomes clear that it does not have any permanent specific feature that is used for discriminating against a certain group of people. For instance, race discrimination supposes the *colour of skin* of the discriminated-against individuals, *sex* is a criterion for gender discrimination (it is generally used by men against women), and *age* is the main characteristic for age discrimination (the mature people tend to discriminate against old people or young adults). All of these criteria have the status of permanent characteristics, which cannot ever be changed; they are immanent (inborn or acquired for life), and the people’s affiliation with race, gender and even old age (ageing is an irreversible process) remains the same permanently.

As for the VSD, the category of the discriminated-against people is changeable, and the out-group members can easily become the in-group members after they obtain a vaccine.

The world’s community has fallen into two basic oppositional groups according to the criterion of “*vaccinated/non-vaccinated*”. There is an intermediate group of people, who belong to the non-vaccinated category; they hold contradictory views on the COVID-19 vaccines and have health-related medical exemptions, whilst nothing is clear about these people’s positive or negative attitude toward the vaccination campaign. Additionally, there are two specific sub-groups of people. The first includes those individuals who belong to the group of “vaccinated” due to the fact that they have taken a vaccine, but who are not supportive of this campaign and have been vaccinated only due to the pressure of the mandatory campaign in society. The second sub-group includes non-vaccinated people who support the vaccination campaign, but have not received a vaccine because of their medical exemption. Important to note in this subdivision is that social discrimination on this criterion is based on *the fact of being vaccinated*, but not on people’s support or rejection of the vaccination initiative.

Thus, the discourse analysis of the VSD issue demonstrates the following correlation of viewpoints, which trigger discrimination in society as a phenomenon: (a) the medical experts’ opinions are contradictory on almost every important matter concerning the COVID-19 vaccination; (b) the government officials, who are deeply interested in the stabilisation of the pandemic situation, are supportive of vaccination and are not considering any alternative variants or how to kill the disease, whilst they are also issuing acts and rules on mandatory vaccination; the anti-vaxxers are being punished by numerous social restrictions, fees, and sometimes by imprisonment, amounting to human rights violations; (c) the social media discourse is extremely biased and aggressive. The overall aggressiveness is being stimulated, and kept up in public, by two dominant factors: the official pressure on the people’s will, on the one hand, and the existence of two basic oppositional groups in society, on the other. The latter issue is aggravated by the fact that it greatly concerns people’s health and lives, which are directly dependent on the opinions of the oppositional group.

The dynamics of social discourse tension within the period between the pandemic beginning and a mandatory vaccination enactment in many countries of the world showed the governments’ regulatory failure. The vaccination campaign was lagging, and people were joining in protest movements against governments and their policies.

The authorities tried to curry favour with people. The government of the state of Maryland (USA), for instance, promised a 100 USD reward to any municipal worker who received a vaccination; indeed, this was presented as stimulation but could be considered bribery or corruption (https://www.rbc.ru/society/28/07/2021/61018b3e9a794743244955a1). Additionally, Russia launched a lottery draw that only included vaccinated people (https://www.kp.ru/daily/28319/4461246/). Nevertheless, all of these measures, restrictions and stimulations proved to not be very effective. The first two years (2019 and 2020) showed a very slow pace in the vaccination rate growth.

It is common knowledge that “it may be much more effective to control the minds of others through persuasion by making them comply out of their own free will” (van Dijk, 2004, p. 101). Thus, presumably, the only way to convince the majority that vaccination is an urgent necessity is to use framing effect methods as a strategic tool for influencing people’s opinions. Nevertheless, practically speaking, “persuasion” can be strategically different by its nature: namely, (a) highly argumentative; (b) manipulative; or (c) exerting authoritative pressure as the basic means of argumentation.

Publications in the last year show that the authorities and mass media, supporting governmental structures’ policies, have preferred the latter two methods in order to pit social groups against each other. A routine method to produce a negative perception of those who have not taken a vaccine yet is to manipulate public consciousness through the pejorative labelling of this group of people. There have appeared many neologisms in different languages to describe people “who oppose vaccination or laws that mandate vaccination” (Merriam-Webster Dictionary, 2021), namely “anti-vaxxers” or “dissidents”. These specific words often contain negative connotations. The English social discourse vocabulary used to negatively label those who are unsupportive of vaccination includes newly-invented words and already-existing invectives such as *anti-vaxxers*, *covid dissidents*, *vaccine refuseniks*, *earthy-crunchies*, *vaccine sceptics*, *vaccine-hesitant*, *law-breakers*, and others.

The analysis of vaccination discourse proves that its three categories (experts’ arguments, political rhetoric and social media data) differ from each other in using pejorative vocabulary toward discriminated-against people. The political rhetoric is rather reserved in negative labelling, the medical experts almost never use invectives, and the social media discourse represents a vast variety of slanderous expressions and insulting colloquialisms. Regardless, there is the need for an in-depth examination of the question of who tends to give a boost to this discrimination in society mostly. There can be at least three categories of actors in this process: (1) the government officials who are focused on achieving herd immunity by any means and pitting people against each other via their negatively framed speeches; (2) the people who are anxious and scared because of the threat to their lives and health, and finally, get trapped by this trick; or (3) the journalists whose cheap sensationalism causes them to play off one person against another.

Pejorative labelling is widely used as an effective means to exert a negative impact on the public. These social contexts contain accusations against those who are unsupportive of vaccination, with them being called stupid, mentally disabled or even dumb. The way in which this communicative strategy is represented in social discourse differs in three of its categories. The medical experts’ discourse tends to be neutral and avoid defamation. The social media discourse is overfull of invectives and slanderous expressions. The political rhetoric is filled with hints, allusions and allegories. This is the result of the framing effect method performance. Semi-truth is a prerogative and characteristic feature of political discourse in persuasion, as well as an integrative part of the manipulative communicative strategy.

According to a stereotyped way of thinking, the experts’ viewpoints are considered the most reliable source of information. Therefore, if they are biased, they lay the ground for this information manipulation. Any contradictory data in the experts’ rhetoric can be readily used as semi-truthful information by anyone who is interested in the manipulation of people’s minds. Let us take several excerpts of the articles that illustrate contradictory knowledge in the medical experts’ rhetoric—knowledge that has given rise to a semi-truthful representation of the biased information in the political and mass media discourses.

## The framing effect method analysis on a sample of its group element “vaccination safety and reliability”

We have sorted which factors influence people’s opinions most effectively. These factors, being of prior importance for the communicated issue, serve as basic group elements of the frame. Interestingly enough, a professionally structured social frame can make one basic group element (the most influential social factor) sufficient to form a public opinion with an intended outcome.

**Fig. 1:**
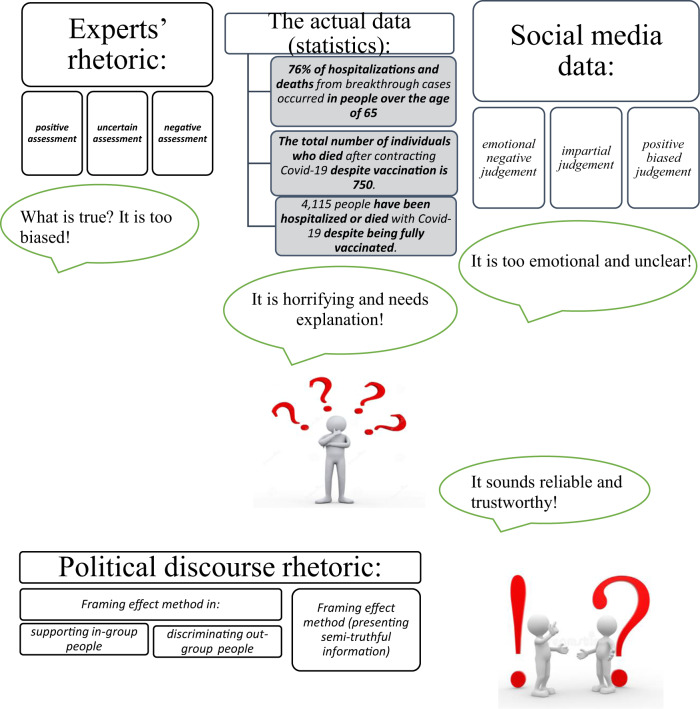
An individual’s choice between different discourse data. A person has to choose among contradictive options. While the experts’ rhetoric remains biased, the actual data are horrifying, social media discourse is negatively emotional, contradictory and full of abusive words, and the political rhetoric (when using the strategy of persuasion) tries to avoid threatening information and tells people about the positive effect of vaccination only. The political discourse serves here as the most strategical type of communication in people convincing.

In order to better understand how the framing effect method performs in public opinion manipulation, let us juxtapose three basic types of social discourse samples that are closely interrelated in this framing process. I have chosen the cases of exploiting the vaccination’s reliability, and the safety factor in mass media resources, as an example of public opinion formation in a manipulative way. It is beyond argument that no one can anticipate all the way through how the framing effect method will influence the opinion of every individual (the reflection of incoming information in people’s minds and the process of cognition, as a whole, are specific and unique, as we have argued before). Regardless of this, the framing effect method targets stereotyped modes of people’s behaviour in order to achieve the desirable effect. The most controllable (and hence predictable) element in people’s communication is the information content assessment (whether it is reliable, complete, uncontroversial, etc.). The clearer and more reliable the incoming information sounds, the more people tend to trust it, because it is their “background knowledge”, in the terms of Abelson’s theory (Schank and Abelson, 1977). If we take vaccination discourse analysis as an example of the framing effect methodology in use, it will become clear that, metaphorically, “all roads lead to Rome”. Whatever an individual tries to read (watch and listen to) and analyse, he/she is highly likely to choose a less contradictory and more pleasant piece of information. Whilst the experts’ rhetoric remains biased, the actual data is horrifying, social media discourse is negatively emotional, contradictory and full of abusive words, and the political rhetoric (when using the strategy of persuasion) tries to avoid threatening information and tells people about the positive effect of vaccination only (see Fig. 1). The political discourse serves here as the most strategic type of communication in convincing people.

The analysis of the following excerpts and the endeavour to deduce an inference from the facts, given below, lead to the conclusion that they do not contain any sufficient argumentation capable of convincing a person to get vaccinated:

The biased experts’ rhetoric:

- *A positive assessment:* “Johns Hopkins Medicine is administering all three COVID-19 vaccines: Pfizer-BioNTech, Moderna and Johnson & Johnson. All three vaccines authorised for emergency use by the Food and Drug Administration (FDA) have been thoroughly tested and *found to be safe and effective in preventing severe COVID-19*. They continue to undergo continuous and intense safety monitoring” (https://www.hopkinsmedicine.org/health/conditions-and-diseases/coronavirus/is-the-covid19-vaccine-safe) (though the information is positive, it lacks argumentation).
- *An uncertain assessment:* “Most people *develop a robust immune response* after getting vaccinated,” but there are those who do not, said Dr. Shahrokh Shabahang, chief innovation officer of Aditx Therapeautics, Inc., or Aditxt. “Why those individuals do not? *They could just be nonresponders to any vaccination protocol,”* he added (https://www.msn.com/en-us/health/medical/why-vaccination-does-not-mean-immunization/ar-BB1foutd).
- *A negative assessment: “Vaccinations*, he (Prof. David Heymann, an American infectious disease epidemiologist and public health expert, based in London—L.G.) explained, *do not guarantee that infectious diseases will be eradicated*” (https://www.herald.ng/covid-19-vaccination-no-guarantee/).

The actual data (statistics):

- *4115 people have been hospitalised or died* from COVID-19 *despite being fully vaccinated*.
- *The total number of individuals who died* after contracting COVID-19 *despite vaccination is 750*.
- *76% of hospitalisations and deaths* from breakthrough cases occurred *in people over the age of 65* (from Health and Science (FRI, June 25, 2021): https://www.cnbc.com/2021/06/25/covid-breakthrough-cases-cdc-says-more-than-4100-people-have-been-hospitalized-or-died-after-vaccination.html).

Social media data:

- *An emotional judgement:* “MailOnline *headline on 13 June read: “Study shows 29% of the 42 people who have died* after catching the new strain *had BOTH vaccinations*.” In Public Health England’s technical briefing on 25 June, that figure had risen to 43% (50 of 117), with the majority (60%) having received at least one dose” (https://www.theguardian.com/theobserver/commentisfree/2021/jun/27/why-most-people-who-now-die-with-covid-have-been-vaccinated).
- *An impartial judgement:* Coverage and effectiveness are important numbers for assessing vaccination programmes. *It is better to look at cool analysis by analysts*, rather than hot takes on social and other media (https://www.theguardian.com/theobserver/commentisfree/2021/jun/27/why-most-people-who-now-die-with-covid-have-been-vaccinated).

The logicality of people’s trust and data reliance is often structured in the definite order of the reliable sources of information they seek out. The majority of people give preference to the most trustworthy—in their opinion—data. Regardless, however, in order to present an accurate analysis of this phenomenon, we need an additional quantitative research study and a series of public opinion surveys.

According to the qualitative research data, we can come to the conclusion that the framing effect method is based on the above-mentioned stereotype, anticipating people’s reliance on the experts’ viewpoints. If the sources are not completely reliable (being biased or contradictory), the second source of information, which is highly exploited for this purpose, is the images of authoritative people. Presumably, the overall authority on the issue of vaccination should belong to the government officials, because one of their primary duties is to take care of the nation’s health. The politicians use at least two basic communicative strategies to sell people on the idea of getting vaccinated: (1) presenting positive semi-truthful information, whilst choosing advantageous points and keeping the disadvantages quiet; (2) setting people at loggerheads, whilst discriminating against people by using offensive labels and nicknames aimed at the “out-group members”. Let us consider:

1. *Framing effect method (presenting semi-truthful information) used in political rhetoric* (a politician is trying to predispose people to get vaccinated, relying on medical data and being very reticent about the deaths of the younger, fully vaccinated people):
  “Despite the power of Covid-19 vaccines in cutting the risk of hospitalization and death from the disease, *fully vaccinated people can get very sick and die* from the virus in rare cases. *Those individuals tend to be older than 65 or have weakened immune systems or other severe medical conditions*, an NBC News survey of health officials nationwide found. “Throughout the pandemic, *people who died of Covid-19 were most likely to be older*, and that continues to be true with breakthrough cases,” a spokesperson for the Massachusetts Department of Public Health said in an email” (https://www.nbcnews.com/health/health-news/rarely-covid-vaccine-breakthrough-infections-can-be-severe-who-s-n1274164).
2. *Framing effect method in discriminating against people* (a politician supports those people who call vaccination-hesitant individuals “selfish and stupid”):
  “Professor Bobby Duffy, director of KCL’s Policy Institute, said responses *describing anti-vaxxers as ‘selfish and stupid’ shows the strength of feeling around vaccinations”* (https://metro.co.uk/2020/12/10/anti-vaxxers-called-stupid-and-selfish-as-1-in-5-unlikely-to-get-vaccine-13730541/).

This is a sort of positive and negative reinforcement method used for public opinion framing: the people, who are reluctant to follow the authorities’ (and support the mass media’s) positive argumentation, come under fire for humiliating rhetoric and non-justified accusations. Strategically, they tend to encourage, reprehend and intimidate the population at the same time.

This is yet another peculiarity of the vaccination status discrimination, where the actors (let us call them “vaccination activists”), who use the framing effect method to dispose other people to get vaccinated, exploit the topic of people’s mental disability, degrading anti-vaxxers in their rhetoric. This type of discrimination is based on pejorative evaluation and negative judgement, which encourages the vaccination-hesitant people to disprove this biased view of their mental abilities by joining the in-group of the “wiser” individuals, who have already been vaccinated.

## Psychological background for the framing effect method’s efficiency

According to the carried out investigation, the framing effect method usually focuses on people’s vulnerabilities, which makes those people feel unaware and uncertain, causing anxiety, phobia, trouble, etc. People’s vulnerabilities can be of an objective and a subjective nature. The subjective factors concern the individual’s sensitivity towards communicative manipulation strategies, which are based on psychological pressure and focused on achieving a certain psychical effect and a person’s reaction. The objective factors, i.e. making people doubt their decision-making, are bound by contradictory official information, such as biased medical experts’ rhetoric (and the other authoritative sources of information from the individual’s perspective), which proves the absence of valid, reliable data on the issue, and that an individual has to choose between indefinite options.

The analysis of social discourse data shows that people tend to be hesitant and sometimes feel uncertain (or, on the contrary, feel negatively certain) about vaccination for the following reasons:

- They have to choose between “probable (non-complete and non-guaranteed) protection” against COVID-19 (which they have not caught yet and probably will not do in the future) and the actual threat of side effects after vaccination and, probably, death.
- They have active antibodies after recovery from COVID-19 and do not think that they need the vaccine.
- They have already recovered from COVID-19 and are anxious about their health condition, but the government has enforced mandatory vaccination for certain categories of the population.
- They are anxious because their government is disregarding people’s chronic diseases and other medical problems, thus disregarding their lives.
- They fear possible consequences of vaccination, such as serious long-term complications—including lupus, infertility, blindness, paralysis, and neurological damage.
- They resist violence, regarding mandatory vaccination as a violation of human rights.
- They do not trust pharma and government authorities and do not consider their rhetoric and actions to be trustworthy.
- They have religious objections.
- They believe in conspiracy theories, etc.

All of these arguments, and many others that make people feel uncertain (or negatively certain) and hesitant about vaccination, are widely used by politicians and mass media in order to discriminate against people, putting those people in a sticky situation. These reasons for people’s hesitation are used in aggressive rhetoric, which is usually a combination of a negative assessment of a person (stupid, non-educated, dumb, etc.) and a proposition, a judgement based on one of the above-mentioned reasons for people’s hesitation. “When anti-vaxxers mount massive protests against immunisation laws, as they did not long ago in California, it is an easy out to characterise their motives as *a lack of intelligence or a generalised hostility* toward science” (https://undark.org/2019/04/24/anti-vaxxers-vaccines-trust-big-pharma/).

Real facts being substituted for a less reasonable false explanation in order to prove the people’s mental handicap is the other strategic tool in framing effect technology. Therefore, the authorities do not use this tool for the purpose of forcing people to get vaccinated; they possess some more efficient instruments of pressure, such as restrictions and orders, but they frame the social discourse in a certain way, making the in-group members (i.e. vaccination activists) actively use their rhetoric in discriminating against and humiliating the out-group people (anti-vaxxers).

The vaccination activists also tend to choose the most incredible reasons from the list of all existing ones. For instance, they ascribe “rolling out their latest conspiracy theories” to all out-group members, seriously talking “about microchips and a new world order”. For the sake of clarity, they pathetically juxtapose these “crazy ideas” to a very serious activity of those people who “rush to create an effective vaccine for the novel coronavirus in order to save potentially millions of people from unnecessary death” (https://god.dailydot.com/anti-vaxxers-coronavirus/). It is only fair to say that not all vaccination-hesitant people’s anticipations, which seemed to be incredible two years ago, have proved to be “stupid fictions”. For instance, at the beginning of 2020, no one could even imagine “that a coronavirus vaccine would be in any way mandatory”, and the people who were trying to predict “literal Nazi-style roundups of people who would refuse it” were called “crazy” (https://god.dailydot.com/anti-vaxxers-coronavirus/). For now, the USA has already “forced vaccination of all military personnel with the present COVID-19 vaccines” (https://www.lifesitenews.com/opinion/mandatory-covid-19-vaccination-for-the-us-military-is-a-national-security-threat/), and more than 18 other countries have “ordered all workers with public-facing roles to be vaccinated against COVID-19” (https://news.trust.org/item/20210804140458-ari9l). Many people understand that it is “an unlawful discriminatory practice for a person or governmental entity to deny services, goods, privileges, licensing, educational opportunities or employment opportunities based on vaccination status or whether someone has an immunity passport” (https://www.usnews.com/news/best-states/montana/articles/2021-04-27/bill-to-prohibit-work-discrimination-based-on-vaccine-status). Despite the fact that Montana’s legislature (USA) passed a bill “that would prohibit employers from requiring vaccinations as a condition of employment” in April 2021, it was sent back by the governor with an amendment. The amendment exempted “nursing homes and long-term care facilities from the measure’s provisions” and allowed “health care facilities to ask employees to volunteer information about their vaccination status, to consider employees who do not volunteer that information to be unvaccinated, and to implement policies specific to unvaccinated staff, patients and visitors that are designed to protect against the spread of communicable diseases” (https://www.usnews.com/news/best-states/montana/articles/2021-04-27/bill-to-prohibit-work-discrimination-based-on-vaccine-status). The bill was later approved with this amendment.

The framing effect method in discriminatory practice helps the government officials to avoid responsibility for unlawful regulations. They accentuate those aspects in regulating only, which do not contravene the law, keeping quiet the discriminatory methods, which are based on violation of human rights. When they formulate the demand “to ask employees to volunteer information about their vaccination status” this way, the key term in this regulation is “volunteering information”, which does not contain any meaning of “the act of violence”. The person is not going to be punished if he/she does not volunteer this information, according to the above-mentioned regulation. Regardless, the consequence of the individual’s refusal to volunteer the said information will take a heavy toll on the person. The individual will be automatically considered unvaccinated and will fall into a discriminated-against out-group, whose members are supposed to be fired, subjected to restrictions, and denied services and opportunities.

It should be emphasised that the government officials do not use the framing effect method as a discriminatory tool directly. Their speeches are usually symbolic, containing the settings of their talks instead of direct wording. The setting of the official speeches’ fragments extends beyond governments through the media with implied meaning “to be addressed below” (see, for details, van Dijk, 2004, p. 122). The authorities do not deliver unlawful orders directly. They imply the consequences of disobedience in their official speeches. This implication is usually perceived by their subordinates as the guideline to drive the process. Let us consider the following example:

“The reality is such that *discrimination will inevitably set in*. People without vaccination or immunity *will not be able to work everywhere*. It’s not possible. It will pose a threat to those around them,” Kremlin spokesman Dmitry Peskov said on 22 June 2021 (https://news.yahoo.com/kremlin-says-people-without-vaccine-094232897.html?fr=sycsrp_catchall).

Indeed, two days after this government leader’s “anticipation” concerning inevitable discrimination, many Moscow and St. Petersburg university lecturers and workers from other municipal organisations received official notices with the demand to submit vaccination or medical exemption certificates. If they failed or refused to do so, they were threatened by suspension without pay. Nevertheless, “… *vaccination remains voluntary*,” Kremlin spokesman Dmitry Peskov said (https://edition.cnn.com/2021/06/29/europe/russia-vaccine-voluntary-compulsory-cmd-intl/index.html).

The unlawfulness of vaccination mandating makes the authorities adopt measures that contradict their stereotyped actions and declarations. As I have already mentioned, and mass media sources prove it to be true, they “are trying to coax and compel people to get vaccinated, offering those who do the chance to win new cars and flats, while threatening others who do not with loss of earnings and dismissal” (https://www.reuters.com/world/europe/kremlin-says-people-without-vaccine-or-immunity-russia-will-have-limited-work-2021-06-22/). The discriminating orders and coaxing rhetoric alternate with each other, producing a contradictory effect on the public, and making people balance between two oppositional options in their choice.

Despite the government officials’ active efforts to persuade people to get vaccinated, the vaccination campaign had been stagnating until the authorities of many countries imposed mandatory vaccination and severe punishment for refusal to take a vaccine. The framing effect has proved to be not very effective for this particular case.

## Analytics

It is reasonable now to comment on the whole process by summing up the most important points that have drastically influenced the cavalcade of recent social events dealing with the vaccination campaign and discrimination against people according to their vaccination status.

Although at the time, there were five vaccines in the world approved by the WHO, and 16 national health organisations adopted their own vaccines by 28 August 2021, the total number of fully vaccinated people in the world amounted to 26.8% of the world’s population. The vaccination process was stagnating, and it was far removed from achieving the desirable condition for herd immunity. After imposing mandatory vaccination, restrictions and severe punishment for refusal to get vaccinated, the government authorities achieved a more or less desirable effect: “65% of the world’s population has received at least one dose of a COVID-19 vaccine; 11.49 billion doses have been administered globally, and 10.66 million are now administered each day. Only 15.2% of people in low-income countries have received at least one dose” as of 20 April 2022 (https://ourworldindata.org/covid-vaccinations). Regardless, it is unlikely that all vaccinated people will definitely fall into the vaccination-supporting category. The achieved vaccination campaign data is more likely to be the result of authoritative violence rather than of the people’s own volition. A valid answer to this question can be given after additional quantitative research and public inquiry.

The framing effect method has failed, regardless of its proven efficiency as a manipulative tool in public opinion formation. What is the root cause of this after-effect?

We can conclude that an individual’s decision-making process depends on the deep interrelation of many superficial and internal factors, which make this process unique and almost unpredictable in a certain way. However, there are stereotypical behavioural patterns that can be exploited effectively in order to achieve a desirable result.

Presumably, all strategies and technologies used by the authorities have been destined to be a success. The framing effect method as a powerful manipulative tool should have succeeded, but it failed. Why so?

Suggestively, there are many internal factors that can outweigh any strategic framing mechanism, used as a tool, aimed at public opinion and decision-making formation.

Research has shown that people’s opinions can be comparatively easily framed and reframed when the basic point in decision-making does not directly concern any question of vital importance to them. Mandatory vaccination has become a question of vital importance.

According to the analysis of social communication resources, the inferential causes of the people’s hesitancy in vaccination decision-making are:

- They have to choose between two negative options (e.g. between a high probability of catching the virus without vaccination and probable side effects after vaccination).
- They do not trust government officials and do not believe in their sincere concern for their lives.
- They resist any sort of violence, including mandatory vaccination.
- They believe in a conspiracy theory.
- They do not trust any of the existing vaccines.
- They cannot find reliable non-contradictory information about vaccination.
- They cannot get vaccinated for religious reasons, etc.

Collectively, people tend to resist any encroachment on their freedom and become extremely cautious when they face a threat to their lives and health. Biased data and misinformation also tend to erode people’s trust in vaccines. Another constraining factor is that many people do not consider this dangerous disease to be a real threat to their lives. Being healthy enough, they tend to rely on their immunity. A certain category of people does not believe in any “snake in the grass” as a potential danger but reacts strongly against restrictive measures and punishment for non-observance of rules.

Interestingly enough, the framing effect phenomenon has revealed a contradiction in its functioning within the COVID-19 vaccination campaign. Theoretically, it was initially supposed to succeed because of its adjustable instrument of persuasion, namely semi-truth. Regardless, however, at its strategical level, the framing effect has failed due to the absence of solid facts and argumentation, and because of the authorities' reluctance to be honest about certain negative facts. Semi-truth regarding vaccination side-effects, for instance, has turned out to be a lie: whilst designating some possible unserious short-term side-effects, such as pain or swelling where the shot was given, as well as fatigue and headache, there has been a silencing of effects such as myocarditis and pericarditis, in addition to severe allergic reactions, including anaphylaxis, thromboembolism and death. Even if strong vaccine side-effects are extremely rare, misinformation on this point threatens to erode the public’s trust in the whole vaccination campaign.

Within the first two years of the COVID-19 pandemic, the world’s authorities did not take into account the following aspect: psychologically, different oppositional strategic methods combined in public persuasion tend to mislead people and limit their trust in authorities and their actions. Alternating threats, restrictions, and punishment with moral suasion and eye-wash produce unfavourable effects. Everything spirals into mistrust amongst the public and hesitation in decision-making on vaccination.

The only still-effective methods when it comes to influencing people are the mandating of vaccination and the imposing of limitations. Regardless, these are both bordering on human rights violations and lead to protest movements amongst the public. Even if more and more people are getting vaccinated under the pressure of the government’s mandatory vaccination campaign, the quantity of people speaking out against vaccination status discrimination has been gradually increasing in society during that time.

## Conclusion

The logicality of the vaccination discrimination framing and, coincidently, the sketch of the VSD frame is as follows: the VSD spontaneously emerged as a new type of intergroup discrimination. It acquired the status of discrimination as a social phenomenon when the government officials issued restricting regulations and declared vaccination-hesitant people to be lawbreakers. This discriminatory practice has been taken up by some representatives of mass media. Common people, being misled and threatened by medical experts, abused by authorities and humiliated and insulted by bystanders, have become extremely cautious and aggressive. The community has split into two antagonistic groups. All of these events have caused aggressive discriminatory rhetoric in society, and the process of discrimination has expanded globally.

The framing effect method used by government authorities, which was initially supposed to succeed in persuading people to take a vaccine, has proved to be non-effective. Severe restrictions, punishment for disobedience, and mandatory vaccination have had a negative effect only. They have triggered numerous protesting movements in the world.

Objectively, a strategy of partial information presentation will be effective if it is supported by solid facts. Biased information tends to mislead people and make them hesitant in their decision-making. Subjectively, the framing effect method proves to be a success in cases that are not of vital importance to the audience, when the people have to decide their own destiny, their health and their future lives.

## Supplementary information

Supplementray Data Sources

## Data Availability

All data underpinning this study are available from the sources listed in the Supplementary File. **Theoretical background data** were obtained from the following sources: Pinker S (1994) The language instinct. How mind creates language. http://f.javier.io/rep/books/The-Language-Instinct-How-the-Mind-Creates-Language,-Steven-Pinker.pdf. Densley J, Peterson J (2017) Group aggression. Curr Opin Psychol 19:43–48. https://daneshyari.com/article/preview/5033457.pdf/. DeWitt L (2007) Framing the future social security debate. http://socialwelfare.library.vcu.edu/social-security/framing-the-future-social-security-debate/. Tajfel H (1970) Experiments in intergroup discrimination. http://www.holah.karoo.net/tajfestudy.htm. What is discrimination? Reasons, features, types. https://amrom.ru/diskriminatsiya/. Meadows DH, Meadows DL, Randers J, Behrens WW (1972) The limits to growth; a report for the Club of Rome’s project on the predicament of mankind. Universe Book, New York. https://web.ics.purdue.edu/~wggray/Teaching/His300/Illustrations/Limits-to-Growth.pdf. Tkachenko D (2020) Chto takoye discriminatsiya: 5 glavnykh priznakov (What discrimination is: 5 main characteristic features). https://dnevnik-znaniy.ru/znaj-i-umej/chto-takoe-diskriminaciya.html. Senin (2009) Communication across culture. https://linguistics1.blogspot.com/2009/. Tajfel H, Billig M, Bundy RP, Flament C (1971) Social categorisation and intergroup behaviour. https://onlinelibrary.wiley.com/doi/epdf/10.1002/ejsp.2420010202. Coe K (2016) Rhetoric, political. In: The international encyclopaedia of political communication. https://www.researchgate.net/publication/314581209_Rhetoric_Politicalhttps://onlinelibrary.wiley.com/doi/pdf/10.1111/1467-9566.ep11343880. Cognitive space. https://psychology.wikia.org/wiki/Cognitive_space#:~:text=A%20cognitive%20space%20consists%20of%20two%20elements%3A%20the,views%2C%20symbols%2C%20common%20language%20use%2C%20common%20ways-to-do-things%2C%20etc.%29. Kolesnikov A (2020) Neirobiologiya peremen. Pochemu nash mozg soprotivlyaetsya vsemu novomu i kak ego nastroit’ na uspekh (Neurobiology of transition. Why our brain resists new things and how to set it up for success). In: Britt A (ed) Popurri. https://smart-lab.ru/blog/632144.php. Discrimination. Dictionary.com. https://www.dictionary.com/browse/discrimination. Kohler-Hausmann I (2020) Discrimination. Oxford bibliographies. https://www.oxfordbibliographies.com/view/document/obo-9780199756384/obo-9780199756384-0013.xml. Kaufman S, Elliott M, Shmueli D (2003) Frames, framing and reframing. https://www.researchgate.net/publication/259558652_Frames_Framing_and_Reframing. Dijk TA (2004) Analysing racism through discourse analysis some methodological reflections TEUN. https://www.semanticscholar.org/paper/Analysing-Racism-Through-Discourse-Analysis-Some-Dijk/2f69df430a9132cf8ea063a9e0286d47ce738918)/. Semi-truth. Urban dictionary. https://www.urbandictionary.com/define.php?term=Semi-Truth. Framing (social sciences). Wikipedia. https://en.wikipedia.org/wiki/Framing_(social_sciences). In-group and out-group. Wikipedia. https://en.wikipedia.org/wiki/In-group_and_out-group. Wertheimer M (1996) A contemporary perspective on the psychology of productive thinking. University of Boulder, Colorado. https://files.eric.ed.gov/fulltext/ED406635.pdf. Difference between racism and discrimination (2016) Pediaa. https://pediaa.com/difference-between-racism-and-discrimination/. Why do we treat our in-group better than we do our out-group? (2022). https://thedecisionlab.com/biases/in-group-bias. **Social discourse data** were obtained from the following sources: Zhitelyam Nyu-Yorka zaplatyat po 100 $ za privivku ot koronavirusa (The New Yorkers will be paid 100$ each for a coronavirus vaccination shot) (2021). https://www.rbc.ru/society/28/07/2021/61018b3e9a794743244955a1. Chernova A (2021) Russia says people can decline its vaccine. But for many, they’ll get fired if they do. https://edition.cnn.com/2021/06/29/europe/russia-vaccine-voluntary-compulsory-cmd-intl/index.html). 19 mind-numbingly stupid anti-vaxxer posts about the coronavirus pandemic (2020). https://god.dailydot.com/anti-vaxxers-coronavirus/. Jones H (2020) People think anti-vaxxers are ‘stupid and selfish’. https://metro.co.uk/2020/12/10/anti-vaxxers-called-stupid-and-selfish-as-1-in-5-unlikely-to-get-vaccine-13730541/. FACTBOX-Countries making COVID-19 vaccines mandatory (2021). https://news.trust.org/item/20210804140458-ari9l. Kremlin says people without vaccine or immunity in Russia will have limited work options (2021). https://news.yahoo.com/kremlin-says-people-without-vaccine-094232897.html?fr=sycsrp_catchall. Many anti-vaxxers don’t trust big pharma. Here’s why. https://undark.org/2019/04/24/anti-vaxxers-vaccines-trust-big-pharma/. Furman J (2021) Mandatory COVID-19 vaccination for the US Military is a national security threat. https://www.lifesitenews.com/opinion/mandatory-covid-19-vaccination-for-the-us-military-is-a-national-security-threat/. Spiegelholter D, Master A (2021) Why most people who now die with COVID in England have had a vaccination. https://www.theguardian.com/theobserver/commentisfree/2021/jun/27/why-most-people-who-now-die-with-covid-have-been-vaccinated. Every single COVID-19 vaccination is a vote for the new global dictatorship (2021). The expose. https://expose-news.com/2021/12/29/every-vaccination-is-a-vote-for-global-dictatorship/. **Medical experts’ discourse data** were obtained from the following sources: McNeil T (2021) How viruses mutate and create new variants. https://now.tufts.edu/articles/how-viruses-mutate-and-create-new-variants. Coronavirus (COVID-19) Vaccinations (2022). https://ourworldindata.org/covid-vaccinations. Ians (2021) Mass vaccination during pandemic historical blunder: Nobel laureate. https://telanganatoday.com/mass-vaccination-during-pandemic-historical-blunder-nobel-laureate. Omicron Variant: What You Need to Know (2021). https://www.cdc.gov/coronavirus/2019-ncov/variants/omicron-variant.html. Mendez R (2021) CDC says roughly 4100 people have been hospitalised or died with COVID breakthrough infections after vaccination. https://www.cnbc.com/2021/06/25/covid-breakthrough-cases-cdc-says-more-than-4100-people-have-been-hospitalized-or-died-after-vaccination.html. Ologunagba C (2020) COVID-19 vaccination no guarantee of virus eradication—WHO. https://www.herald.ng/covid-19-vaccination-no-guarantee/. Maragakis L, Kelen GD (2022) Is the COVID-19 vaccine safe? https://www.hopkinsmedicine.org/health/conditions-and-diseases/coronavirus/is-the-covid19-vaccine-safe. Vrach-infektsionist: otkaz ot privivki mozhet privesti k kardinal’nomu izmeneniyu koronavirusa (An Infectious Disease Physician says that the vaccine refusal may cause a cardinal mutation in the coronavirus) (2021). https://www.invitro.ru/about/press_relizes/vrach-infektsionist-otkaz-ot-privivki-mozhet-privesti-k-kardinalnomu-izmeneniyu-koronavirusa/. Herd immunity and COVID-19: What you need to know (2022). https://www.mayoclinic.org/diseases-conditions/coronavirus/in-depth/herd-immunity-and-coronavirus/art-20486808. Why vaccination doesn’t mean immunisation. https://mph.chm.msu.edu/news-items/covid-19/the-vaccination-and-building-trust/322-why-vaccination-does-not-mean-immunization. Edwards E, Strickler L (2021) Rarely, COVID vaccine breakthrough infections can be severe. Who’s at risk? https://www.nbcnews.com/health/health-news/rarely-covid-vaccine-breakthrough-infections-can-be-severe-who-s-n1274164. Sycheva I (2021) V Rossii—139 shtammov koronavirusa. Kakiye iz nikh samyie opasnyie? (There are 139 strains of coronavirus in Russia. Which of them are the most virulent ones?). https://www.pravmir.ru/v-rossii-139-shtammov-koronavirusa-kakie-iz-nih-samye-opasnye/. McNamara D (2021) ‘A few mutations away’: the threat of a vaccine-proof variant. https://www.webmd.com/vaccines/covid-19-vaccine/news/20210730/threat-of-vaccine-proof-covid-variant. Macmillan C (2021) Herd immunity: will we ever get there? https://www.yalemedicine.org/news/herd-immunity. **Political discourse data** were obtained from the following sources: Rozygrysh 100 tysyach rubley za privivku v Rossii: kak poluchit’ priz za vaktsinatsiyu ot koronavirusa s 1 sentyabrya 2021? (100 thousand ruble Lottery draw in Russia for getting vaccinated: how can you win the prize for a vaccination shot till 1 September 2021)? https://www.kp.ru/daily/28319/4461246/. Montana Bill aims to stop work bias based on vaccine status (usnews.com). https://www.usnews.com/news/best-states/montana/articles/2021-04-27/bill-to-prohibit-work-discrimination-based-on-vaccine-status. Mandatory coronavirus vaccination bill introduced in New York State Assembly (28 Dec 2020). https://www.naturalnews.com/2020-12-28-mandatory-coronavirus-vaccination-bill-introduced-ny-assembly.html. 50-State update on legislation pertaining to employer-mandated vaccinations (23 Feb 2022). https://www.huschblackwell.com/newsandinsights/50-state-update-on-pending-legislation-pertaining-to-employer-mandated-vaccinations. State efforts to ban or enforce COVID-19 Vaccine Mandates and Passports (11 Jul 2022). https://www.nashp.org/state-lawmakers-submit-bills-to-ban-employer-vaccine-mandates/. Scientists worry about political influence over Coronavirus Vaccine Project (2020). https://www.nytimes.com/2020/08/02/us/politics/coronavirus-vaccine.html.

## References

[CR1] Allport GW (1954). The nature of prejudice.

[CR2] Blank RM, Dabady M, Citro CF, Dabady M, Citro CF (2004). Defining discrimination. Measuring racial discrimination.

[CR3] Brewer PR, Sigelman L (2002). Political scientists as color commentators: framing and expert commentary in media campaign coverage. Harvard Int J Press/Politics.

[CR4] Bryman A (2012). Social research methods.

[CR5] Bukhanovsky AO, Kutayvin YUA, Litvak ME (2000). Obschaya psikhopatalogiya: posobiye dlya vrachey (General psychology: a training manual for physicians).

[CR6] Capaldi N (2001) The meaning of equality. Hoover Press

[CR7] Chong D, Druckman J (2007). A theory of framing and opinion formation in competitive elite environments. J Commun.

[CR8] Coe K (2016) Rhetoric, political. In: The international encyclopedia of political communication. https://www.researchgate.net/publication/314581209_Rhetoric_Political. Accessed 3 Aug 2022

[CR9] Converse PE (1964) The nature of belief systems in mass publics. In: Apter DE (ed.) Ideology and Its Discontent. New York: Free Press of Glencoe, pp. 206–261

[CR10] Coser LA (1956). The functions of social conflict.

[CR11] Densley J, Peterson J (2017). Group aggression. Curr Opin Psychol.

[CR12] Dewitt L (2007) Framing the future social security debate. Social welfare history project. http://socialwelfare.library.vcu.edu/social-security/framing-the-future-social-security-debate/. Accessed 12 Jul 2021

[CR13] Discrimination (2021) https://www.dictionary.com/browse/discrimination. Accessed 7 July 2021

[CR14] Discrimination (article) (2021). In: Merriam-Webster dictionary. https://www.merriamwebster.com/dictionary/discrimination

[CR15] Diskriminatsiya—chto eto? (2021) Discrimination—what is this? Amrom J. https://amrom.ru/diskriminatsiya/. Accessed 25 Aug 2021

[CR16] Druckman JN (2001). The implications of framing effects for citizen competence. Political Behav.

[CR17] Fillmore CHJ, Baker CF (2001) Frame semantics for text understanding. In: ICSI Research group (Al) (eds) Proceedings of WordNet and other lexical resources workshop, NACL, 3–4 June 2001

[CR18] Framing (social sciences) (2021) https://en.wikipedia.org/wiki/Framing_(social_sciences). Accessed 13 Aug 2021

[CR19] Gitlin T (1980). The whole world is watching: Mass media in the making and unmaking of the new left.

[CR20] Goffman E (1974). Frame analysis: an essay on the organization of experience.

[CR21] Gurevich L (2009). Kognitivnoye prostranstvo metacommunicatsii (Cognitive space of metacommunication).

[CR22] Hasa (2016) Difference between racism and discrimination. https://pediaa.com/difference-between-racism-and-discrimination/. Accessed 17 Aug 2021

[CR23] In-group favouritism (2021) https://en.wikipedia.org/wiki/In-group_and_out-group. Accessed 7 Jul 2021

[CR24] Invitro monitoring (2021) https://www.invitro.ru/about/press_relizes/vrach-infektsionist-otkaz-ot-privivki-mozhet-privesti-k-kardinalnomu-izmeneniyu-koronavirusa/. Accessed 21 Aug 2021

[CR25] Kahneman D, Tversky A (1979). Prospect theory: an analysis of decision under risk. Econometrica.

[CR26] Kahneman D, Tversky A (1984). Choices, values, and frames. Am Psychol.

[CR27] Kaufman S, Elliot M, Shmueli D (2003) Frames, framing and reframing. https://www.researchgate.net/publication/259558652_Frames_Framing_and_Reframing. Accessed 27 Aug 2021

[CR28] Kohler-Hausmann I (2020) Discrimination. https://www.oxfordbibliographies.com/view/document/obo-9780199756384/obo-9780199756384-0013.xml. Accessed 22 Aug 2021

[CR29] Kolesnikov A (2020) Neirobiologiya peremen. Pochemu nash mozg soprotivlyaetsya vsemu novomu i kak ego nastroit’ na uspekh (Neurobiology of transition. Why our brain resists new things and how to set it up for success). In: Britt A (ed) Popurri. https://smart-lab.ru/blog/632144.php. Accessed 24 Aug 2021

[CR30] Lakoff G (2004) Do not think of an elephant!: know your values and frame the debate: the essential guide for progressives. Chelsea Green Publishing Company

[CR31] Macmillan C (2021) Herd immunity: will we ever get there? https://www.yalemedicine.org/news/herd-immunity. Accessed 26 Aug 2021

[CR32] Mass vaccination during pandemic historical blunder: Nobel laureate (2021). SocialNews.XYZ (25 May 2021). https://telanganatoday.com/mass-vaccination-during-pandemic-historical-blunder-nobel-laureate. Accessed 20 Aug 2021

[CR33] McNamara D (2021) A few mutations away: the threat of a vaccine-proof variant. https://www.webmd.com/vaccines/covid-19-vaccine/news/20210730/threat-of-vaccine-proof-covid-variant. Accessed 23 Aug 2021

[CR34] McNeil T (2021) How viruses mutate and create new variants. https://now.tufts.edu/articles/how-viruses-mutate-and-create-new-variants. Accessed 25 Aug 2021

[CR35] Meadows DH, Meadows DL, Randers J, Behrens WW (1972). The Limits to growth; a report for the Club of Rome’s project on the predicament of mankind.

[CR36] Meditsinskaya entsiklopedia: noveishii spravochnik prakticheskogo psikhologa (Medical Encyclopedia: a contemporary book of reference for a practical psychologist) (2006) (Medical Encyclopedia: a contemporary book of reference for a practical psychologist. AST, Moscow; Sova, Saint Petersburg

[CR37] Omicron variant: what you need to know (2021) Centers of disease control and prevention (CDC) (Updated 20 Dec 2021). https://www.cdc.gov/coronavirus/2019-ncov/variants/omicron-variant.html. Accessed 27 Jan 2022

[CR38] Pager D, Shepherd H (2008). The sociology of discrimination: racial discrimination in employment, housing, credit, and consumer markets. Annu Rev Sociol.

[CR39] Pinker S (2003) The language instinct. http://f.javier.io/rep/books/The-Language-Instinct-How-the-Mind-Creates-Language,-Steven-Pinker.pdf. Accessed 30 Aug 2021

[CR40] Schank R, Abelson RP (1977). Scripts, plans, goals and understanding: an inquiry into human knowledge structures.

[CR41] Scheufele DA (2000). Agenda-setting, priming, and framing revisited: another look at cognitive effects of political communication. Mass Commun Soc.

[CR42] Scheufele DA, Tewksbury D (2007). Framing, agenda-setting, and priming: the evolution of three media effects models. J Commun.

[CR43] Senin (2009) Framing theory: framing and interpreting. https://linguistics1.blogspot.com/2009/. Accessed 30 Aug 2021

[CR44] Sheigal EI (2000). Semiotika politicheskogo diskursa (Semiotics of political discourse). Institut Yazykoznaniya RAN, Volgogr.

[CR45] Shpar VB, Timchenko AV, Shvydchenko VN (2020). Prakticheskaya psikhologiya. Instrumentariy (Practical psychology. A range of tools).

[CR46] Sycheva I (2021) V Rossii—139 shtammov koronavirusa. Kakiye iz nikh samyie opasnyie? (There are 139 strains of coronavirus in Russia. Which of them are the most virulent ones?). https://www.pravmir.ru/v-rossii-139-shtammov-koronavirusa-kakie-iz-nih-samye-opasnye/. Accessed 29 Aug 2021

[CR47] Tajfel H (1970) Experiments in intergroup discrimination. Scientific American, 233 (5):96–102. Reprinted in R. C. Atkinson (Ed.), (1971). *Contemporary psychology*. San Francisco: W. H. Freeman 5482577

[CR48] Tajfel H, Billig MG, Bundy RP, Flament C (1971) Social categorization and intergroup behaviour. Eur J Soc Psychol 1(2):149–178. https://onlinelibrary.wiley.com/doi/epdf/10.1002/ejsp.2420010202

[CR49] Tkachenko D (2020) Chto takoye discriminatsiya: 5 glavnykh priznakov (What discrimination is: 5 main characteristic features). https://dnevnik-znaniy.ru/znaj-i-umej/chto-takoe-diskriminaciya.html. Accessed 1 Nov 2021

[CR50] Tversky A, Kahneman D (1981). The framing of decisions and the psychology of choice. Science.

[CR51] Urban dictionary (2021) https://www.urbandictionary.com/define.php?term=Semi-Truth. Accessed 26 Aug 2021

[CR52] van Dijk TA (2004) Analyzing racism through discourse analysis. Some methodological reflections. https://www.semanticscholar.org/paper/Analyzing-Racism-Through-Discourse-Analysis-Some-Dijk/2f69df430a9132cf8ea063a9e0286d47ce738918)/. Accessed 26 Apr 2021

[CR53] Verhoeven J, Helle HJ (1985). Goffman’s frame. Analysis in relation to modern micro-sociological paradigms. Perspectives on micro-sociological theory.

[CR54] Wertheimer M (1959). Productive thinking. Enlarged edn.

[CR55] Wertheimer M (1996) A contemporary perspective on the psychology of productive thinking. University of Boulder, Boulder, CO. https://files.eric.ed.gov/fulltext/ED406635.pdf. Accessed 25 Aug 2021

[CR56] Whitney P (2001). Schemas, frames and scripts in cognitive psychology. Int Encycl Soc Behav Sci.

[CR57] Why do we treat our in-group better than we do our out-group? (2022) https://thedecisionlab.com/biases/in-group-bias. Accessed 3 Aug 2022

[CR58] Williams RG (1981) Logical analysis as a qualitative method II: conflict of ideas and the topic of illness. Sociol Health Illn https://onlinelibrary.wiley.com/doi/pdf/10.1111/1467-9566.ep11343880. Accessed 27 Aug 2021

