# Supplementary material for: Framing effect method in vaccination status discrimination research
Source: Humanit Soc Sci Commun. 2022 Aug 18;9(1):278. doi: 10.1057/s41599-022-01299-x (PMC9386658; doi:10.1057/s41599-022-01299-x)
Supplement: Supplementary file 1 — Supplementray Data Sources [file 41599_2022_1299_MOESM1_ESM.docx]

Database

<https://blogs.bmj.com/medical-ethics/2021/03/01/discrimination-on-the-basis-of-vaccination-status-is-inherently-wrong/>

<https://constitutionwatch.com.au/wp-content/uploads/COVID-19-VACCINATION-STATUS-PREVENTION-OF-DISCRIMINATION-BILL-2021.pdf>

<https://forums.hardwarezone.com.sg/threads/say-no-to-using-vaccination-status-discrimination-to-encourage-vaccination.6557204/>

<https://gerardrennick.com.au/covid-19-vaccination-status-prevention-of-discrimination-bill-2021/>

<https://law.stackexchange.com/questions/70537/discrimination-due-to-covid-19-certificate-requirements>

<https://ohiocapitaljournal.com/2021/04/07/bill-would-prevent-discrimination-against-unvaccinated-people/>

<https://petition.parliament.uk/petitions/596004>

<https://www.abc.net.au/news/2021-09-17/small-businesses-worried-about-confrontations-with-unvaccinated/100467952>

<https://www.ageofautism.com/2015/12/vaccine-discrimination-in-america.html>

<https://www.aph.gov.au/Parliamentary_Business/Bills_Legislation/Bills_Search_Results/Result?bId=s1325>

<https://www.brookings.edu/blog/how-we-rise/2021/10/20/discrimination-in-the-healthcare-system-is-leading-to-vaccination-hesitancy/>

<https://www.change.org/p/paul-green-parliament-nsw-gov-au-say-no-to-discrimination-of-children-based-on-vaccination-status>

<https://www.hrexchangenetwork.com/dei/columns/how-vaccination-status-can-lead-to-discrimination>

<https://www.jonathanlea.net/blog/generic-discrimination-on-the-basis-of-vaccination-status-letter/>

<https://www.labordaysblog.com/2021/09/the-new-employee-status-vaccinated-or-unvaccinated/>

<https://www.lexology.com/library/detail.aspx?g=5694c83a-49fe-4a48-b9c0-301231a5c89c>

<https://www.littler.com/publication-press/publication/montana-releases-faqs-vaccine-discrimination-law>

<https://www.naturalnews.com/2021-10-20-employers-following-vaccine-mandates-violate-non-discrimination-clause.html>

<https://www.onenation.org.au/vaccine-mandates>

<https://www.pinsentmasons.com/out-law/news/vaccine-passports-discrimination-risk-for-employers>

<https://www.quora.com/Is-discrimination-based-on-vaccination-status-being-an-acceptable-way-to-fight-Covid19-even-though-it-has-been-successful>

<https://www.reignitedemocracyaustralia.com.au/sunrise-discrimination/>

<https://www.researchgate.net/publication/361187507_Is_intergroup_discrimination_triggered_by_vaccination_status_Exploring_some_social_aspects_of_vaccinations_during_the_COVID-19_pandemic>

<https://www.ricemedia.co/features-covid-19-vaccine-passport-discriminatory/>

<https://www.thehastingscenter.org/should-covid-vaccinated-patients-get-priority-treatment/>

<https://www.theorganicprepper.com/unvaccinated-discrimination/>

<https://www.thesundaily.my/local/don-t-discriminate-on-vaccination-status-CX7426994>

<https://www.venable.com/insights/publications/2021/04/vaccination-discrimination-a-new-shot-at-employ>

<https://www.washingtonpost.com/outlook/2020/12/15/vaccine-cards-discrimination-immunity-passports/>

<https://www.zenefits.com/workest/who-is-entitled-to-know-an-employees-vaccination-status/>

The supplementary material includes more that 500 resourses analysed.

Here is the list of my search in Corpus of contemporary American English and other articles:

| The Coronavirus Corpus (COCA)  FIND SAMPLE:  [100](https://www.english-corpora.org/corona/x3.asp?node=&sample=100&w10=coronavirus&w11=vaccination&r=)  [200](https://www.english-corpora.org/corona/x3.asp?node=&sample=200&w10=coronavirus&w11=vaccination&r=)  [500](https://www.english-corpora.org/corona/x3.asp?node=&sample=500&w10=coronavirus&w11=vaccination&r=)  [1000](https://www.english-corpora.org/corona/x3.asp?node=&sample=1000&w10=coronavirus&w11=vaccination&r=) PAGE:   [**<<**](https://www.english-corpora.org/corona/x3.asp?node=&p=1&w10=coronavirus&w11=vaccination&r=) [**<**](https://www.english-corpora.org/corona/x3.asp?node=&p=1&w10=coronavirus&w11=vaccination&r=)   1 / 42  [**>**](https://www.english-corpora.org/corona/x3.asp?node=&p=2&w10=coronavirus&w11=vaccination&r=) [**>>**](https://www.english-corpora.org/corona/x3.asp?node=&p=42&w10=coronavirus&w11=vaccination&r=) |  |
| --- | --- |

Начало формы

| 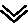  CLICK FOR MORE CONTEXT | [**HELP**](javascript:newFeatures()) [[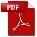](https://www.english-corpora.org/files/kwic_tools.pdf)](https://www.english-corpora.org/files/kwic_tools.pdf) | [[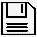](javascript:chooser('s'))  SAVE](javascript:chooser('s'))    [[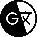](javascript:chooser('t'))  TRANSLATE](javascript:chooser('t'))    [[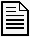](javascript:chooser('p'))  ANALYZE](javascript:chooser('p')) |
| --- | --- | --- |

| [1](https://www.english-corpora.org/corona/x4.asp?rs=&t=33783541&ID=23544302288) | [22-02-18 US](https://www.english-corpora.org/corona/x4.asp?rs=&t=33783541&ID=23544302288) | [bostonglobe.com](https://www.bostonglobe.com/2022/02/18/world/statewide-mask-mandates-being-lifted-across-us-mainland/) | [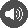](https://translate.google.com/?sl=en&tl=en&text=++NEW+YORK+TIMES++++Vaccination+rates+for+nursing+home+workers+varies+widely++++Coronavirus+vaccination+rates+among+nursing+home+staff+varied+widely+by+state%2C+from+70+percent+to+nearly&op=translate) | [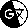](javascript:xtrans('1')) | [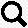](javascript:xword('1')) | # NEW YORK TIMES # Vaccination rates for nursing home workers varies widely # **Coronavirus** **vaccination** rates among nursing home staff varied widely by state, from 70 percent to nearly |
| --- | --- | --- | --- | --- | --- | --- |
| [2](https://www.english-corpora.org/corona/x4.asp?rs=&t=33783733&ID=23539290995) | [22-02-18 US](https://www.english-corpora.org/corona/x4.asp?rs=&t=33783733&ID=23539290995) | [bostonherald.com](https://www.bostonglobe.com/2022/02/18/world/statewide-mask-mandates-being-lifted-across-us-mainland/) | [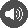](https://translate.google.com/?sl=en&tl=en&text=the+city+calls+the+mandate+that+many+businesses+require+patrons+to+provide+proof+of+coronavirus+vaccination%2E++++%22+The+public+health+data+shows+that+we%27re+ready+to+take+this&op=translate) | [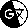](javascript:xtrans('2')) | [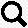](javascript:xword('2')) | the city calls the mandate that many businesses require patrons to provide proof of **coronavirus** **vaccination**. # " The public health data shows that we're ready to take this |
| [3](https://www.english-corpora.org/corona/x4.asp?rs=&t=33785958&ID=23544769746) | [22-02-18 US](https://www.english-corpora.org/corona/x4.asp?rs=&t=33785958&ID=23544769746) | [dailynews.com](https://www.bostonglobe.com/2022/02/18/world/statewide-mask-mandates-being-lifted-across-us-mainland/) | [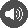](https://translate.google.com/?sl=en&tl=en&text=predicts+there+could+be+a+fourth+shot+%2D%2D+either+a+booster+or+a+generalized+coronavirus+vaccination+%2D%2D+coming+down+the+pike+in+late+summer+or+fall%2E++++She+hopes+more&op=translate) | [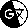](javascript:xtrans('3')) | [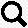](javascript:xword('3')) | predicts there could be a fourth shot -- either a booster or a generalized **coronavirus** **vaccination** -- coming down the pike in late summer or fall. # She hopes more |
| [4](https://www.english-corpora.org/corona/x4.asp?rs=&t=33787542&ID=23562288675) | [22-02-19 US](https://www.english-corpora.org/corona/x4.asp?rs=&t=33787542&ID=23562288675) | [nytimes.com](https://www.bostonglobe.com/2022/02/18/world/statewide-mask-mandates-being-lifted-across-us-mainland/) | [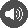](https://translate.google.com/?sl=en&tl=en&text=of+conspiring+with+a+nurse+to+steal%2C+forge+and+distribute+hundreds+of+fraudulent+coronavirus+vaccination+cards%2C+prosecutors+said+on+Thursday%2E++++The+reservist%2C+Jia+Liu%2C+26&op=translate) | [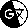](javascript:xtrans('4')) | [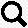](javascript:xword('4')) | of conspiring with a nurse to steal, forge and distribute hundreds of fraudulent **coronavirus** **vaccination** cards, prosecutors said on Thursday. # The reservist, Jia Liu, 26 |
| [5](https://www.english-corpora.org/corona/x4.asp?rs=&t=33790749&ID=23573970043) | [22-02-20 US](https://www.english-corpora.org/corona/x4.asp?rs=&t=33790749&ID=23573970043) | [nytimes.com](https://www.bostonglobe.com/2022/02/18/world/statewide-mask-mandates-being-lifted-across-us-mainland/) | [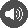](https://translate.google.com/?sl=en&tl=en&text=last+week+about+a+%22+next%2Dphase+%22+plan%2C+which+would+prioritize+strategies+like+coronavirus+vaccination+and+stockpiling+supplies+while+easing+away+from+emergency+response+measures+like+mask+mandates%2E+++&op=translate) | [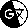](javascript:xtrans('5')) | [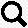](javascript:xword('5')) | last week about a " next-phase " plan, which would prioritize strategies like **coronavirus** **vaccination** and stockpiling supplies while easing away from emergency response measures like mask mandates. # |
| [6](https://www.english-corpora.org/corona/x4.asp?rs=&t=33797285&ID=23588268061) | [22-02-21 US](https://www.english-corpora.org/corona/x4.asp?rs=&t=33797285&ID=23588268061) | [freep.com](https://www.bostonglobe.com/2022/02/18/world/statewide-mask-mandates-being-lifted-across-us-mainland/) | [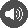](https://translate.google.com/?sl=en&tl=en&text=administered%2E+Recipients+must+be+at+least+18+years+old+and+have+completed+the+coronavirus+vaccination+series+by+June+10%2E+Residents+must+bring+their+vaccination+card+and+health+insurance+information&op=translate) | [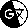](javascript:xtrans('6')) | [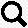](javascript:xword('6')) | administered. Recipients must be at least 18 years old and have completed the **coronavirus** **vaccination** series by June 10. Residents must bring their vaccination card and health insurance information |
| [7](https://www.english-corpora.org/corona/x4.asp?rs=&t=33804498&ID=23619213660) | [22-02-23 US](https://www.english-corpora.org/corona/x4.asp?rs=&t=33804498&ID=23619213660) | [pilotonline.com](https://www.bostonglobe.com/2022/02/18/world/statewide-mask-mandates-being-lifted-across-us-mainland/) | [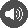](https://translate.google.com/?sl=en&tl=en&text=The+Hampton+and+Peninsula+Health+Districts+announced+the+pendingclosure+of+the+Sherwood+Shopping+Center+coronavirus+vaccination+site+Wednesday%2C+citing+a+decrease+in+demand+as+more+people+are+fully+vaccinated%2E&op=translate) | [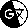](javascript:xtrans('7')) | [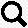](javascript:xword('7')) | The Hampton and Peninsula Health Districts announced the pendingclosure of the Sherwood Shopping Center **coronavirus** **vaccination** site Wednesday, citing a decrease in demand as more people are fully vaccinated. |
| [8](https://www.english-corpora.org/corona/x4.asp?rs=&t=44009737&ID=23546101317) | [22-02-18 US](https://www.english-corpora.org/corona/x4.asp?rs=&t=44009737&ID=23546101317) | [whnt.com](https://www.bostonglobe.com/2022/02/18/world/statewide-mask-mandates-being-lifted-across-us-mainland/) | [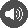](https://translate.google.com/?sl=en&tl=en&text=R%2DTexas%29+that+would+block+federal+funding+for+schools+and+childcare+centers+that+require+coronavirus+vaccination+failed%2C+along+with+one+from+Sen%2E+Mike+Lee+%28R%2DUtah%29+and+other+GOP&op=translate) | [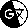](javascript:xtrans('8')) | [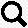](javascript:xword('8')) | R-Texas) that would block federal funding for schools and childcare centers that require **coronavirus** **vaccination** failed, along with one from Sen. Mike Lee (R-Utah) and other GOP |
| [9](https://www.english-corpora.org/corona/x4.asp?rs=&t=88795810&ID=23534683580) | [22-02-17 US](https://www.english-corpora.org/corona/x4.asp?rs=&t=88795810&ID=23534683580) | [The Washington Post on MSN.com](https://www.bostonglobe.com/2022/02/18/world/statewide-mask-mandates-being-lifted-across-us-mainland/) | [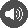](https://translate.google.com/?sl=en&tl=en&text=Schemidt%2FAFP%2FGetty+Images%29++++Israel+and+European+countries+are+dropping+some+travel+restrictions+and+coronavirus+vaccination+and+masking+requirements%2E++++Israeli+officials+announced+that+some+restrictions+on+foreign+tourists+would&op=translate) | [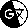](javascript:xtrans('9')) | [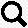](javascript:xword('9')) | Schemidt/AFP/Getty Images) # Israel and European countries are dropping some travel restrictions and **coronavirus** **vaccination** and masking requirements. # Israeli officials announced that some restrictions on foreign tourists would |
| [10](https://www.english-corpora.org/corona/x4.asp?rs=&t=88795922&ID=23535078716) | [22-02-17 US](https://www.english-corpora.org/corona/x4.asp?rs=&t=88795922&ID=23535078716) | [CNN](https://www.bostonglobe.com/2022/02/18/world/statewide-mask-mandates-being-lifted-across-us-mainland/) | [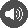](https://translate.google.com/?sl=en&tl=en&text=role%2E+%22++++The+memo+also+states%2C+%22+completion+of+SARS+CoV2+coronavirus+vaccination+including+booster+vaccine+is+strongly+recommended+at+least+two+weeks+prior+to+the+event%2E&op=translate) | [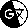](javascript:xtrans('10')) | [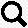](javascript:xword('10')) | role. " # The memo also states, " completion of SARS CoV2 **coronavirus** **vaccination** including booster vaccine is strongly recommended at least two weeks prior to the event. |
| [11](https://www.english-corpora.org/corona/x4.asp?rs=&t=88796102&ID=23535118283) | [22-02-17 US](https://www.english-corpora.org/corona/x4.asp?rs=&t=88796102&ID=23535118283) | [The Guardian on MSN.com](https://www.bostonglobe.com/2022/02/18/world/statewide-mask-mandates-being-lifted-across-us-mainland/) | [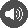](https://translate.google.com/?sl=en&tl=en&text=than+50%2C000+last+month%2E++++Israel%27s+prime+minister+says+the+country%27s+coronavirus+vaccination+%22+green+pass+%22+system+will+be+suspended+as+new+daily+cases+of+Covid+continue&op=translate) | [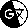](javascript:xtrans('11')) | [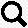](javascript:xword('11')) | than 50,000 last month. # Israel's prime minister says the country's **coronavirus** **vaccination** " green pass " system will be suspended as new daily cases of Covid continue |
| [15](https://www.english-corpora.org/corona/x4.asp?rs=&t=88796471&ID=23537827456) | [22-02-17 US](https://www.english-corpora.org/corona/x4.asp?rs=&t=88796471&ID=23537827456) | [ABC](https://www.bostonglobe.com/2022/02/18/world/statewide-mask-mandates-being-lifted-across-us-mainland/)[(3)](https://www.english-corpora.org/corona/duplicates1.asp?xx=14&n=3) | [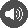](https://translate.google.com/?sl=en&tl=en&text=also+schemed+with+a+nurse+to+steal%2C+forge+and+sell+hundreds+of+coronavirus+vaccination+cards++++By+JENNIFER+PELTZ+Associated+Press++++February+18%2C+2022%2C+1%3A51+AM&op=translate) | [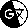](javascript:xtrans('12')) | [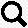](javascript:xword('12')) | also schemed with a nurse to steal, forge and sell hundreds of **coronavirus** **vaccination** cards # By JENNIFER PELTZ Associated Press # February 18, 2022, 1:51 AM |
| [16](https://www.english-corpora.org/corona/x4.asp?rs=&t=88796471&ID=23537827536) | [22-02-17 US](https://www.english-corpora.org/corona/x4.asp?rs=&t=88796471&ID=23537827536) | [ABC](https://www.bostonglobe.com/2022/02/18/world/statewide-mask-mandates-being-lifted-across-us-mainland/) | [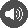](https://translate.google.com/?sl=en&tl=en&text=also+schemed+with+a+nurse+to+steal%2C+forge+and+sell+hundreds+of+fake+coronavirus+vaccination+cards+and+destroy+vaccine+doses+to+fake+inoculations%2C+federal+authorities+said+Thursday%2E+++&op=translate) | [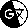](javascript:xtrans('13')) | [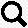](javascript:xword('13')) | also schemed with a nurse to steal, forge and sell hundreds of fake **coronavirus** **vaccination** cards and destroy vaccine doses to fake inoculations, federal authorities said Thursday. # |
| [17](https://www.english-corpora.org/corona/x4.asp?rs=&t=88809705&ID=23569410549) | [22-02-19 US](https://www.english-corpora.org/corona/x4.asp?rs=&t=88809705&ID=23569410549) | [CNBC](https://www.bostonglobe.com/2022/02/18/world/statewide-mask-mandates-being-lifted-across-us-mainland/) | [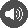](https://translate.google.com/?sl=en&tl=en&text=week+over+allegations+that+Joe+Rogan%2C+Spotify%27s+star+podcaster%2C+was+spreading+coronavirus+vaccination+misinformation+with+his+show%2E++++%22+We+support+Neil+and+we+agree+with+him&op=translate) | [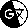](javascript:xtrans('14')) | [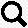](javascript:xword('14')) | week over allegations that Joe Rogan, Spotify's star podcaster, was spreading **coronavirus** **vaccination** misinformation with his show. # " We support Neil and we agree with him |
| [18](https://www.english-corpora.org/corona/x4.asp?rs=&t=88811064&ID=23578464074) | [22-02-20 US](https://www.english-corpora.org/corona/x4.asp?rs=&t=88811064&ID=23578464074) | [Reuters on MSN.com](https://www.bostonglobe.com/2022/02/18/world/statewide-mask-mandates-being-lifted-across-us-mainland/) | [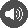](https://translate.google.com/?sl=en&tl=en&text=anti%2Dvaxxer+and+that+his+refusal+to+be+vaccinated+stems+from+uncertainty+over+how+a+coronavirus+vaccination+could+affect+his+playing+performance%2E++++World+number+two+Medvedev%2C+who+is+playing&op=translate) | [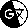](javascript:xtrans('15')) | [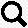](javascript:xword('15')) | anti-vaxxer and that his refusal to be vaccinated stems from uncertainty over how a **coronavirus** **vaccination** could affect his playing performance. # World number two Medvedev, who is playing |
| [19](https://www.english-corpora.org/corona/x4.asp?rs=&t=88813200&ID=23576850150) | [22-02-20 US](https://www.english-corpora.org/corona/x4.asp?rs=&t=88813200&ID=23576850150) | [Washington Post](https://www.bostonglobe.com/2022/02/18/world/statewide-mask-mandates-being-lifted-across-us-mainland/) | [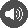](https://translate.google.com/?sl=en&tl=en&text=long+list+of+Report24+articles+on+the+coronavirus+appears+to+demonstrate+a+bias+against+coronavirus+vaccination%2E+But+in+an+email+exchange+with+The+Fact+Checker%2C+a+person+who+responded&op=translate) | [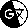](javascript:xtrans('16')) | [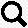](javascript:xword('16')) | long list of Report24 articles on the coronavirus appears to demonstrate a bias against **coronavirus** **vaccination**. But in an email exchange with The Fact Checker, a person who responded |
| [20](https://www.english-corpora.org/corona/x4.asp?rs=&t=88817954&ID=23594291168) | [22-02-21 US](https://www.english-corpora.org/corona/x4.asp?rs=&t=88817954&ID=23594291168) | [Washington Post](https://www.bostonglobe.com/2022/02/18/world/statewide-mask-mandates-being-lifted-across-us-mainland/) | [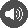](https://translate.google.com/?sl=en&tl=en&text=%2E++++Story+continues+below+advertisement++++A+majority+of+voters+also+support+requiring+coronavirus+vaccination+for+people+in+jail+or+prison%2C+first+responders%2C+members+of+the+military%2C&op=translate) | [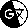](javascript:xtrans('17')) | [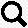](javascript:xword('17')) | . # Story continues below advertisement # A majority of voters also support requiring **coronavirus** **vaccination** for people in jail or prison, first responders, members of the military, |
| [21](https://www.english-corpora.org/corona/x4.asp?rs=&t=88817954&ID=23594291231) | [22-02-21 US](https://www.english-corpora.org/corona/x4.asp?rs=&t=88817954&ID=23594291231) | [Washington Post](https://www.bostonglobe.com/2022/02/18/world/statewide-mask-mandates-being-lifted-across-us-mainland/) | [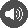](https://translate.google.com/?sl=en&tl=en&text=and+48+percent+no+for+both%2E++++There+was+less+support+for+requiring+coronavirus+vaccination+in+public+schools%2E+Voters+were+evenly+split+%2849%2D49%29+on+whether+to+require&op=translate) | [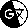](javascript:xtrans('18')) | [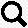](javascript:xword('18')) | and 48 percent no for both. # There was less support for requiring **coronavirus** **vaccination** in public schools. Voters were evenly split (49-49) on whether to require |
| [22](https://www.english-corpora.org/corona/x4.asp?rs=&t=88819970&ID=23609781352) | [22-02-22 US](https://www.english-corpora.org/corona/x4.asp?rs=&t=88819970&ID=23609781352) | [Forbes](https://www.bostonglobe.com/2022/02/18/world/statewide-mask-mandates-being-lifted-across-us-mainland/) | [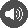](https://translate.google.com/?sl=en&tl=en&text=++Hahn+says+Los+Angeles+County%27s+rule+will+only+apply+to+spaces+with+coronavirus+vaccination+requirements%2C+such+as+restaurants%2C+bars+and+gyms%2C+meaning+grocery+stores+and+other&op=translate) | [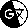](javascript:xtrans('19')) | [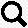](javascript:xword('19')) | # Hahn says Los Angeles County's rule will only apply to spaces with **coronavirus** **vaccination** requirements, such as restaurants, bars and gyms, meaning grocery stores and other |
| [23](https://www.english-corpora.org/corona/x4.asp?rs=&t=88821512&ID=23607141497) | [22-02-22 US](https://www.english-corpora.org/corona/x4.asp?rs=&t=88821512&ID=23607141497) | [SFGate](https://www.bostonglobe.com/2022/02/18/world/statewide-mask-mandates-being-lifted-across-us-mainland/) | [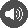](https://translate.google.com/?sl=en&tl=en&text=faces+scrutiny+by+a+parliamentary+health+committee%2EHajarah+Nalwadda%2FAPShow+MoreShow+Less5of12A+Ugandan+receives+a+Pfizer+coronavirus+vaccination+at+the+Kiswa+Health+Centre+III+in+the+Bugolobi+neighborhood+of+Kampala%2C+Uganda+Tuesday&op=translate) | [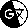](javascript:xtrans('20')) | [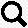](javascript:xword('20')) | faces scrutiny by a parliamentary health committee.Hajarah Nalwadda/APShow MoreShow Less5of12A Ugandan receives a Pfizer **coronavirus** **vaccination** at the Kiswa Health Centre III in the Bugolobi neighborhood of Kampala, Uganda Tuesday |
| [25](https://www.english-corpora.org/corona/x4.asp?rs=&t=88821512&ID=23607141821) | [22-02-22 US](https://www.english-corpora.org/corona/x4.asp?rs=&t=88821512&ID=23607141821) | [SFGate](https://www.bostonglobe.com/2022/02/18/world/statewide-mask-mandates-being-lifted-across-us-mainland/)[(1)](https://www.english-corpora.org/corona/duplicates1.asp?xx=24&n=1) | [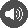](https://translate.google.com/?sl=en&tl=en&text=scrutiny+by+a+parliamentary+health+committee%2EHajarah+Nalwadda%2FAPShow+MoreShow+Less11of12A+nurse+prepares+a+Pfizer+coronavirus+vaccination+at+the+Kiswa+Health+Centre+III+in+the+Bugolobi+neighborhood+of+Kampala%2C+Uganda+Tuesday&op=translate) | [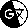](javascript:xtrans('21')) | [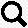](javascript:xword('21')) | scrutiny by a parliamentary health committee.Hajarah Nalwadda/APShow MoreShow Less11of12A nurse prepares a Pfizer **coronavirus** **vaccination** at the Kiswa Health Centre III in the Bugolobi neighborhood of Kampala, Uganda Tuesday |
| [26](https://www.english-corpora.org/corona/x4.asp?rs=&t=88828587&ID=23627038382) | [22-02-23 US](https://www.english-corpora.org/corona/x4.asp?rs=&t=88828587&ID=23627038382) | [Reuters](https://www.bostonglobe.com/2022/02/18/world/statewide-mask-mandates-being-lifted-across-us-mainland/) | [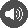](https://translate.google.com/?sl=en&tl=en&text=DTEGn%2EDE%29+subsidiary+T%2DSystems+to+build+a+software+solution+for+global+electronic+verification+of+coronavirus+vaccination+certificates%2C+the+telecoms+company+said%2E++++The+QR+code%2Dbased+software+solution+will+be&op=translate) | [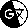](javascript:xtrans('22')) | [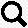](javascript:xword('22')) | DTEGn.DE) subsidiary T-Systems to build a software solution for global electronic verification of **coronavirus** **vaccination** certificates, the telecoms company said. # The QR code-based software solution will be |
| [27](https://www.english-corpora.org/corona/x4.asp?rs=&t=88829642&ID=23641367531) | [22-02-24 US](https://www.english-corpora.org/corona/x4.asp?rs=&t=88829642&ID=23641367531) | [The Washington Post on MSN.com](https://www.bostonglobe.com/2022/02/18/world/statewide-mask-mandates-being-lifted-across-us-mainland/) | [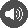](https://translate.google.com/?sl=en&tl=en&text=new+report+sought+to+investigate+potential+cases+of+MIS%2DC+in+youngsters+who+had+received+coronavirus+vaccination+and+examined+a+large+pool+of+data+reported+to+a+national+MIS%2DC+surveillance+system%2C&op=translate) | [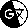](javascript:xtrans('23')) | [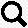](javascript:xword('23')) | new report sought to investigate potential cases of MIS-C in youngsters who had received **coronavirus** **vaccination** and examined a large pool of data reported to a national MIS-C surveillance system, |
| [28](https://www.english-corpora.org/corona/x4.asp?rs=&t=88829642&ID=23641368338) | [22-02-24 US](https://www.english-corpora.org/corona/x4.asp?rs=&t=88829642&ID=23641368338) | [The Washington Post on MSN.com](https://www.bostonglobe.com/2022/02/18/world/statewide-mask-mandates-being-lifted-across-us-mainland/) | [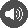](https://translate.google.com/?sl=en&tl=en&text=is+important+to+remember+that+MIS%2DC+is+rare+and+that+the+best+defense+is+coronavirus+vaccination%2E++++Paul+Offit%2C+director+of+the+Vaccine+Education+Center+at+Children%27s+Hospital&op=translate) | [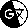](javascript:xtrans('24')) | [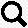](javascript:xword('24')) | is important to remember that MIS-C is rare and that the best defense is **coronavirus** **vaccination**. # Paul Offit, director of the Vaccine Education Center at Children's Hospital |
| [29](https://www.english-corpora.org/corona/x4.asp?rs=&t=88830888&ID=23643854411) | [22-02-24 US](https://www.english-corpora.org/corona/x4.asp?rs=&t=88830888&ID=23643854411) | [The Guardian on MSN.com](https://www.bostonglobe.com/2022/02/18/world/statewide-mask-mandates-being-lifted-across-us-mainland/) | [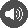](https://translate.google.com/?sl=en&tl=en&text=sub%2Dvariant%2C+according+to+data+from+South+Africa%2E++++The+rollout+of+the+coronavirus+vaccination+programme+has+been+praised+by+the+UK%27s+public+spending+watchdog+for+meeting+%22+stretching&op=translate) | [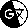](javascript:xtrans('25')) | [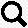](javascript:xword('25')) | sub-variant, according to data from South Africa. # The rollout of the **coronavirus** **vaccination** programme has been praised by the UK's public spending watchdog for meeting " stretching |
| [31](https://www.english-corpora.org/corona/x4.asp?rs=&t=88846436&ID=23681797785) | [22-02-27 US](https://www.english-corpora.org/corona/x4.asp?rs=&t=88846436&ID=23681797785) | [Washington Post](https://www.bostonglobe.com/2022/02/18/world/statewide-mask-mandates-being-lifted-across-us-mainland/)[(1)](https://www.english-corpora.org/corona/duplicates1.asp?xx=30&n=1) | [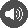](https://translate.google.com/?sl=en&tl=en&text=updated+guidance+also+says+it+is+no+longer+necessary+for+people+to+delay+coronavirus+vaccination+after+receiving+treatments+with+monoclonal+antibodies%2C+a+treatment+authorized+for+early+stage+covid%2D19%2C&op=translate) | [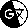](javascript:xtrans('26')) | [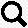](javascript:xword('26')) | updated guidance also says it is no longer necessary for people to delay **coronavirus** **vaccination** after receiving treatments with monoclonal antibodies, a treatment authorized for early stage covid-19, |
| [32](https://www.english-corpora.org/corona/x4.asp?rs=&t=44029979&ID=23604799415) | [22-02-22 GB](https://www.english-corpora.org/corona/x4.asp?rs=&t=44029979&ID=23604799415) | [stv.tv](https://www.bostonglobe.com/2022/02/18/world/statewide-mask-mandates-being-lifted-across-us-mainland/) | [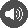](https://translate.google.com/?sl=en&tl=en&text=care%2E++++So+far+4%2C432%2C566+people+have+received+their+first+dose+of+a+coronavirus+vaccination%2C+4%2C151%2C403+have+received+their+second+dose%2C+and+3%2C397%2C270+have+received+a+third+dose&op=translate) | [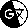](javascript:xtrans('27')) | [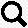](javascript:xword('27')) | care. # So far 4,432,566 people have received their first dose of a **coronavirus** **vaccination**, 4,151,403 have received their second dose, and 3,397,270 have received a third dose |
| [33](https://www.english-corpora.org/corona/x4.asp?rs=&t=44038927&ID=23628399080) | [22-02-24 GB](https://www.english-corpora.org/corona/x4.asp?rs=&t=44038927&ID=23628399080) | [expressandstar.com](https://www.bostonglobe.com/2022/02/18/world/statewide-mask-mandates-being-lifted-across-us-mainland/) | [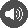](https://translate.google.com/?sl=en&tl=en&text=was+good+value+for+public+money%2C+says+watchdog++++The+rollout+of+the+coronavirus+vaccination+programme+has+been+praised+by+the+UK%27s+public+spending+watchdog+for+meeting+%22+stretching&op=translate) | [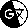](javascript:xtrans('28')) | [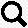](javascript:xword('28')) | was good value for public money, says watchdog # The rollout of the **coronavirus** **vaccination** programme has been praised by the UK's public spending watchdog for meeting " stretching |
| [34](https://www.english-corpora.org/corona/x4.asp?rs=&t=74090212&ID=23599702952) | [22-02-22 AU](https://www.english-corpora.org/corona/x4.asp?rs=&t=74090212&ID=23599702952) | [illawarramercury.com.au](https://www.bostonglobe.com/2022/02/18/world/statewide-mask-mandates-being-lifted-across-us-mainland/) | [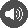](https://translate.google.com/?sl=en&tl=en&text=+++74090212++++Travellers+entering+Tasmania+won%27t+have+to+prove+their+coronavirus+vaccination+status+as+of+this+weekend%2E+The+change%2C+which+means+unvaccinated+people+no+longer&op=translate) | [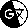](javascript:xtrans('29')) | [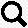](javascript:xword('29')) | 74090212 # Travellers entering Tasmania won't have to prove their **coronavirus** **vaccination** status as of this weekend. The change, which means unvaccinated people no longer |
| [37](https://www.english-corpora.org/corona/x4.asp?rs=&t=74100911&ID=23631082698) | [22-02-24 AU](https://www.english-corpora.org/corona/x4.asp?rs=&t=74100911&ID=23631082698) | [illawarramercury.com.au](https://www.bostonglobe.com/2022/02/18/world/statewide-mask-mandates-being-lifted-across-us-mainland/)[(2)](https://www.english-corpora.org/corona/duplicates1.asp?xx=36&n=2) | [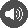](https://translate.google.com/?sl=en&tl=en&text=%2E+Two+patients+are+in+intensive+care%2E+Tasmania+will+drop+proof+of+coronavirus+vaccination+requirements+for+incoming+travellers+at+12%2E01am+on+Saturday%2C+opening+the+door+for+unvaccinated+people&op=translate) | [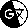](javascript:xtrans('30')) | [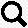](javascript:xword('30')) | . Two patients are in intensive care. Tasmania will drop proof of **coronavirus** **vaccination** requirements for incoming travellers at 12.01am on Saturday, opening the door for unvaccinated people |
| [40](https://www.english-corpora.org/corona/x4.asp?rs=&t=33789976&ID=23565938412) | [22-02-19 PK](https://www.english-corpora.org/corona/x4.asp?rs=&t=33789976&ID=23565938412) | [geo.tv](https://www.bostonglobe.com/2022/02/18/world/statewide-mask-mandates-being-lifted-across-us-mainland/)[(2)](https://www.english-corpora.org/corona/duplicates1.asp?xx=39&n=2) | [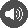](https://translate.google.com/?sl=en&tl=en&text=19%2C+2022++++Health+worker+administrates+anti%2DCovid%2D19+vaccine+to+citizens+during+the+coronavirus+vaccination+door+to+door+campaign%2C+located+on+Buffer+Zone+area+of+Karachi+on+Wednesday%2C&op=translate) | [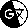](javascript:xtrans('31')) | [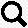](javascript:xword('31')) | 19, 2022 # Health worker administrates anti-Covid-19 vaccine to citizens during the **coronavirus** **vaccination** door to door campaign, located on Buffer Zone area of Karachi on Wednesday, |
| [41](https://www.english-corpora.org/corona/x4.asp?rs=&t=53683984&ID=23589493878) | [22-02-21 BD](https://www.english-corpora.org/corona/x4.asp?rs=&t=53683984&ID=23589493878) | [bdnews24.com](https://www.bostonglobe.com/2022/02/18/world/statewide-mask-mandates-being-lifted-across-us-mainland/) | [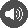](https://translate.google.com/?sl=en&tl=en&text=of+the+coronavirus+pandemic%2C+the+authorities+have+made+it+mandatory+to+present+a+coronavirus+vaccination+certificate%2C+besides+wearing+a+facemask%2C+to+pay+homage+to+the+martyrs+at+the&op=translate) | [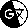](javascript:xtrans('32')) |  | of the coronavirus pandemic, the authorities have made it mandatory to present a **coronavirus** **vaccination** certificate, besides wearing a facemask, to pay homage to the martyrs at the |
| [42](https://www.english-corpora.org/corona/x4.asp?rs=&t=63929850&ID=23651104552) | [22-02-25 ZA](https://www.english-corpora.org/corona/x4.asp?rs=&t=63929850&ID=23651104552) | [dailymaverick.co.za](https://www.bostonglobe.com/2022/02/18/world/statewide-mask-mandates-being-lifted-across-us-mainland/) |  |  |  | the one initially received. # These are some of the changes to the **coronavirus** **vaccination** rules as set out by the National Department of Health: # The interval between |
| [43](https://www.english-corpora.org/corona/x4.asp?rs=&t=53696114&ID=23629167377) | [22-02-24 NG](https://www.english-corpora.org/corona/x4.asp?rs=&t=53696114&ID=23629167377) | [nairametrics.com](https://www.bostonglobe.com/2022/02/18/world/statewide-mask-mandates-being-lifted-across-us-mainland/) |  |  |  | , T-Systems is aimed at building a software solution for global electronic verification of **coronavirus** **vaccination** certificates, the telecoms company said. The App will be designed to display certificates |
| [44](https://www.english-corpora.org/corona/x4.asp?rs=&t=33825573&ID=23698523929) | [22-03-01 US](https://www.english-corpora.org/corona/x4.asp?rs=&t=33825573&ID=23698523929) | [nytimes.com](https://www.bostonglobe.com/2022/02/18/world/statewide-mask-mandates-being-lifted-across-us-mainland/) |  |  |  | " light duties, " the palace said. # She received a first **coronavirus** **vaccination** in January 2021, but the palace has not confirmed whether she received subsequent doses |
| [45](https://www.english-corpora.org/corona/x4.asp?rs=&t=33836134&ID=23718866979) | [22-03-03 US](https://www.english-corpora.org/corona/x4.asp?rs=&t=33836134&ID=23718866979) | [nbcchicago.com](https://www.bostonglobe.com/2022/02/18/world/statewide-mask-mandates-being-lifted-across-us-mainland/) |  |  |  | settings. # For dogs and cats, Arwady said there is no routine **coronavirus** **vaccination** recommended at this time, and she doesn't expect there to be one. |
| [46](https://www.english-corpora.org/corona/x4.asp?rs=&t=33849851&ID=23764817238) | [22-03-07 US](https://www.english-corpora.org/corona/x4.asp?rs=&t=33849851&ID=23764817238) | [washingtonpost.com](https://www.bostonglobe.com/2022/02/18/world/statewide-mask-mandates-being-lifted-across-us-mainland/) |  |  |  | More than 40 of those deaths were in Florida. # The CDC recommends **coronavirus** **vaccination** for children 5 and older, saying the shots are safe and effective against serious |
| [47](https://www.english-corpora.org/corona/x4.asp?rs=&t=34028293&ID=23837084928) | [22-03-14 US](https://www.english-corpora.org/corona/x4.asp?rs=&t=34028293&ID=23837084928) | [theguardian.com](https://www.bostonglobe.com/2022/02/18/world/statewide-mask-mandates-being-lifted-across-us-mainland/) |  |  |  | of people aged five and older in rural counties had received at least one **coronavirus** **vaccination** shot, compared with 75.4% in urban counties. # Paxlovid and molnupiravir are authorized |
| [48](https://www.english-corpora.org/corona/x4.asp?rs=&t=34039044&ID=23878848557) | [22-03-16 US](https://www.english-corpora.org/corona/x4.asp?rs=&t=34039044&ID=23878848557) | [washingtonpost.com](https://www.bostonglobe.com/2022/02/18/world/statewide-mask-mandates-being-lifted-across-us-mainland/) |  |  |  | Tavern, 8401 Georgia Ave., Silver Spring. quarryhousetavern.com. $12. Proof of **coronavirus** **vaccination** is required for admittance. 34039045 4 concerts |
| [49](https://www.english-corpora.org/corona/x4.asp?rs=&t=34050840&ID=23913311296) | [22-03-18 US](https://www.english-corpora.org/corona/x4.asp?rs=&t=34050840&ID=23913311296) | [washingtonexaminer.com](https://www.bostonglobe.com/2022/02/18/world/statewide-mask-mandates-being-lifted-across-us-mainland/) |  |  |  | this month, the Department of Defense asked the Supreme Court to allow its **coronavirus** **vaccination** requirements to apply to Navy SEALs who have refused on religious grounds after a federal |
| [50](https://www.english-corpora.org/corona/x4.asp?rs=&t=34065672&ID=23995234218) | [22-03-24 US](https://www.english-corpora.org/corona/x4.asp?rs=&t=34065672&ID=23995234218) | [sfchronicle.com](https://www.bostonglobe.com/2022/02/18/world/statewide-mask-mandates-being-lifted-across-us-mainland/) |  |  |  | Francisco. San Francisco health officials recently highlighted widening racial and ethnic disparities in **coronavirus** **vaccination** rates among children ages 5 to 11. # Moderna says its COVID-19 vaccine is |
| [52](https://www.english-corpora.org/corona/x4.asp?rs=&t=34067052&ID=24007377888) | [22-03-25 US](https://www.english-corpora.org/corona/x4.asp?rs=&t=34067052&ID=24007377888) | [washingtonpost.com](https://www.bostonglobe.com/2022/02/18/world/statewide-mask-mandates-being-lifted-across-us-mainland/)[(1)](https://www.english-corpora.org/corona/duplicates1.asp?xx=51&n=1) |  |  |  | not been convinced. # Kyle Elston was so eager to get a **coronavirus** **vaccination** that he leaped ahead of his age group, listing himself as obese. # |
| [53](https://www.english-corpora.org/corona/x4.asp?rs=&t=34070154&ID=24008182482) | [22-03-25 US](https://www.english-corpora.org/corona/x4.asp?rs=&t=34070154&ID=24008182482) | [pilotonline.com](https://www.bostonglobe.com/2022/02/18/world/statewide-mask-mandates-being-lifted-across-us-mainland/) |  |  |  | Army has now discharged 27 soldiers for refusing the order to receive the mandatory **coronavirus** **vaccination**, the service announced Thursday. # The Army announced last week that the first |
| [54](https://www.english-corpora.org/corona/x4.asp?rs=&t=34084791&ID=24067242901) | [22-03-29 US](https://www.english-corpora.org/corona/x4.asp?rs=&t=34084791&ID=24067242901) | [sfchronicle.com](https://www.bostonglobe.com/2022/02/18/world/statewide-mask-mandates-being-lifted-across-us-mainland/) |  |  |  | or wait? # Matthew Fletcher, who is experiencing homelessness, gets his **coronavirus** **vaccination** Serenity House in Oakland. The FDA approved second boosters for people 50 and over |
| [55](https://www.english-corpora.org/corona/x4.asp?rs=&t=88856821&ID=23701262357) | [22-03-01 US](https://www.english-corpora.org/corona/x4.asp?rs=&t=88856821&ID=23701262357) | [YAHOO!News](https://www.bostonglobe.com/2022/02/18/world/statewide-mask-mandates-being-lifted-across-us-mainland/) |  |  |  | been suspended in Ukraine because of the fighting. WHO has received reports that **coronavirus** **vaccination** campaigns have also been put on hold in many parts of the country, he |
| [56](https://www.english-corpora.org/corona/x4.asp?rs=&t=88859352&ID=23703072887) | [22-03-01 US](https://www.english-corpora.org/corona/x4.asp?rs=&t=88859352&ID=23703072887) | [Washington Post](https://www.bostonglobe.com/2022/02/18/world/statewide-mask-mandates-being-lifted-across-us-mainland/) |  |  |  | unrest. " # Governments, employers and public health officials globally have encouraged **coronavirus** **vaccination** and championed its primary role in quelling the spread of the deadly virus. In |
| [57](https://www.english-corpora.org/corona/x4.asp?rs=&t=88859473&ID=23704429316) | [22-03-01 US](https://www.english-corpora.org/corona/x4.asp?rs=&t=88859473&ID=23704429316) | [New York Post](https://www.bostonglobe.com/2022/02/18/world/statewide-mask-mandates-being-lifted-across-us-mainland/) |  |  |  | ' health team is quietly launching a $1.9 million campaign to boost **coronavirus** **vaccination** rates in the mostly white neighborhoods of orthodox Jewish Brooklyn as well as parts of |
| [58](https://www.english-corpora.org/corona/x4.asp?rs=&t=88868513&ID=23724370512) | [22-03-03 US](https://www.english-corpora.org/corona/x4.asp?rs=&t=88868513&ID=23724370512) | [Washington Post](https://www.bostonglobe.com/2022/02/18/world/statewide-mask-mandates-being-lifted-across-us-mainland/) |  |  |  | " The View " last year, during which Tafoya clashed with hosts on **coronavirus** **vaccination** mandates, Colin Kaepernick and racial progress in the country. # Advertisement # Story |
| [59](https://www.english-corpora.org/corona/x4.asp?rs=&t=88870064&ID=23725942661) | [22-03-03 US](https://www.english-corpora.org/corona/x4.asp?rs=&t=88870064&ID=23725942661) | [seattlepi.com](https://www.bostonglobe.com/2022/02/18/world/statewide-mask-mandates-being-lifted-across-us-mainland/) |  |  |  | also schemed with a nurse to steal, forge and sell hundreds of fake **coronavirus** **vaccination** cards and destroy vaccine doses to fake inoculations, federal authorities said Thursday. # |
| [60](https://www.english-corpora.org/corona/x4.asp?rs=&t=88894286&ID=23785757323) | [22-03-09 US](https://www.english-corpora.org/corona/x4.asp?rs=&t=88894286&ID=23785757323) | [Washington Examiner](https://www.bostonglobe.com/2022/02/18/world/statewide-mask-mandates-being-lifted-across-us-mainland/) |  |  |  | the pro-life movement? # If the United States sees an uptick in its **coronavirus** **vaccination** rate in the coming months, it might partly be thanks to a new vaccine |
| [61](https://www.english-corpora.org/corona/x4.asp?rs=&t=88895110&ID=23782831754) | [22-03-09 US](https://www.english-corpora.org/corona/x4.asp?rs=&t=88895110&ID=23782831754) | [Yahoo](https://www.bostonglobe.com/2022/02/18/world/statewide-mask-mandates-being-lifted-across-us-mainland/) |  |  |  | # The 20-time major winner revealed last month that he had not received any **coronavirus** **vaccination**, insisting he would forego big tournaments " because the principles of decision-making on my |
| [62](https://www.english-corpora.org/corona/x4.asp?rs=&t=88924156&ID=23872413672) | [22-03-15 US](https://www.english-corpora.org/corona/x4.asp?rs=&t=88924156&ID=23872413672) | [New York Daily News](https://www.bostonglobe.com/2022/02/18/world/statewide-mask-mandates-being-lifted-across-us-mainland/) |  |  |  | the New York State Department of Health, speaks during a news conference on **coronavirus** **vaccination** at Suffolk County Community College on Monday, April 12, 2021 in Brentwood, |
| [63](https://www.english-corpora.org/corona/x4.asp?rs=&t=88935998&ID=23908610502) | [22-03-17 US](https://www.english-corpora.org/corona/x4.asp?rs=&t=88935998&ID=23908610502) | [New York Daily News](https://www.bostonglobe.com/2022/02/18/world/statewide-mask-mandates-being-lifted-across-us-mainland/) |  |  |  | benefits of illness-curbing vaccines. His country has been a world leader in its **coronavirus** **vaccination** and booster shot effort. # Biden, meanwhile, used the annual display of |
| [64](https://www.english-corpora.org/corona/x4.asp?rs=&t=88936765&ID=23906330709) | [22-03-17 US](https://www.english-corpora.org/corona/x4.asp?rs=&t=88936765&ID=23906330709) | [NBC Chicago](https://www.bostonglobe.com/2022/02/18/world/statewide-mask-mandates-being-lifted-across-us-mainland/) |  |  |  | settings. # For dogs and cats, Arwady said there is no routine **coronavirus** **vaccination** recommended at this time, and she doesn't expect there to be one. |
| [65](https://www.english-corpora.org/corona/x4.asp?rs=&t=88980868&ID=24018759400) | [22-03-25 US](https://www.english-corpora.org/corona/x4.asp?rs=&t=88980868&ID=24018759400) | [Forbes](https://www.bostonglobe.com/2022/02/18/world/statewide-mask-mandates-being-lifted-across-us-mainland/) |  |  |  | in Australia to stay and play in the Australian Open tennis tournament over his **coronavirus** **vaccination** status. (Photo by AFPTV / AFP) (Photo by STR/AFPTV/AFP via Getty |
| [66](https://www.english-corpora.org/corona/x4.asp?rs=&t=53741225&ID=23762787216) | [22-03-07 CA](https://www.english-corpora.org/corona/x4.asp?rs=&t=53741225&ID=23762787216) | [cbc.ca](https://www.bostonglobe.com/2022/02/18/world/statewide-mask-mandates-being-lifted-across-us-mainland/) |  |  |  | thousands disperse to other countries from a country that had a low level of **coronavirus** **vaccination**. # The total of six million represents tracking by the Johns Hopkins University Coronavirus |
| [67](https://www.english-corpora.org/corona/x4.asp?rs=&t=44127936&ID=23844163222) | [22-03-14 GB](https://www.english-corpora.org/corona/x4.asp?rs=&t=44127936&ID=23844163222) | [uk.businessinsider.com](https://www.bostonglobe.com/2022/02/18/world/statewide-mask-mandates-being-lifted-across-us-mainland/) |  |  |  | strategy for the virus to stop evolving. " # A nurse marks a **coronavirus** **vaccination** card with a booster dose at a vaccine clinic in Pasadena, California, on |
| [68](https://www.english-corpora.org/corona/x4.asp?rs=&t=44191216&ID=24060175162) | [22-03-29 GB](https://www.english-corpora.org/corona/x4.asp?rs=&t=44191216&ID=24060175162) | [newsletter.co.uk](https://www.bostonglobe.com/2022/02/18/world/statewide-mask-mandates-being-lifted-across-us-mainland/) |  |  |  | (PHA) confirmed on Tuesday that the next phase of the rollout of **coronavirus** **vaccination** boosters will begin in April. It is advised that spring boosters are spaced out |
| [69](https://www.english-corpora.org/corona/x4.asp?rs=&t=88994039&ID=24057330630) | [22-03-28 GB](https://www.english-corpora.org/corona/x4.asp?rs=&t=88994039&ID=24057330630) | [The Guardian](https://www.bostonglobe.com/2022/02/18/world/statewide-mask-mandates-being-lifted-across-us-mainland/) |  |  |  | of people aged five and older in rural counties had received at least one **coronavirus** **vaccination** shot, compared with 75.4% in urban counties. # Paxlovid and molnupiravir are authorized |
| [70](https://www.english-corpora.org/corona/x4.asp?rs=&t=74229459&ID=23865514106) | [22-03-15 IN](https://www.english-corpora.org/corona/x4.asp?rs=&t=74229459&ID=23865514106) | [outlookindia.com](https://www.bostonglobe.com/2022/02/18/world/statewide-mask-mandates-being-lifted-across-us-mainland/) |  |  |  | be vaccinated under the new age group that has now been included in the **coronavirus** **vaccination** program. # Children above 15 years of age were included in the vaccination program |
| [72](https://www.english-corpora.org/corona/x4.asp?rs=&t=74229537&ID=23865519116) | [22-03-15 IN](https://www.english-corpora.org/corona/x4.asp?rs=&t=74229537&ID=23865519116) | [businesstoday.in](https://www.bostonglobe.com/2022/02/18/world/statewide-mask-mandates-being-lifted-across-us-mainland/)[(1)](https://www.english-corpora.org/corona/duplicates1.asp?xx=71&n=1) |  |  |  | giant Biological E Limited, govt had stated. # Registration for the **coronavirus** **vaccination** for children in the age group of 12-14 years will begin from Wednesday, i.e. |
| [74](https://www.english-corpora.org/corona/x4.asp?rs=&t=53831004&ID=23877327185) | [22-03-16 PK](https://www.english-corpora.org/corona/x4.asp?rs=&t=53831004&ID=23877327185) | [nation.com.pk](https://www.bostonglobe.com/2022/02/18/world/statewide-mask-mandates-being-lifted-across-us-mainland/)[(1)](https://www.english-corpora.org/corona/duplicates1.asp?xx=73&n=1) |  |  |  | vaccination was the only and effective treatment against corona. He said that **coronavirus** **vaccination** were available in all vaccination centres. 53831005 |
| [75](https://www.english-corpora.org/corona/x4.asp?rs=&t=44146272&ID=23901739642) | [22-03-17 SG](https://www.english-corpora.org/corona/x4.asp?rs=&t=44146272&ID=23901739642) | [sg.news.yahoo.com](https://www.bostonglobe.com/2022/02/18/world/statewide-mask-mandates-being-lifted-across-us-mainland/) |  |  |  | Moderna booster for those above 60 who took Pfizer earlier: NCID # A **coronavirus** **vaccination** site at a community centre in Singapore. (AFP via Getty Images file photo |
| [76](https://www.english-corpora.org/corona/x4.asp?rs=&t=34111403&ID=24234708574) | [22-04-06 US](https://www.english-corpora.org/corona/x4.asp?rs=&t=34111403&ID=24234708574) | [nytimes.com](https://www.bostonglobe.com/2022/02/18/world/statewide-mask-mandates-being-lifted-across-us-mainland/) |  |  |  | behalf of Ukraine in 2014. 34111404 # A **coronavirus** **vaccination** site in Bari, Italy, in January.Credit... Donato Fasano/Getty Images # European regulators |
| [77](https://www.english-corpora.org/corona/x4.asp?rs=&t=34116994&ID=24258352829) | [22-04-07 US](https://www.english-corpora.org/corona/x4.asp?rs=&t=34116994&ID=24258352829) | [washingtonpost.com](https://www.bostonglobe.com/2022/02/18/world/statewide-mask-mandates-being-lifted-across-us-mainland/) |  |  |  | so they decided to stick it out. 8 p.m. $12-$15. Proof of **coronavirus** **vaccination** is required for admittance. 34116995 Annual Georgetown |
| [79](https://www.english-corpora.org/corona/x4.asp?rs=&t=34122511&ID=24267236606) | [22-04-08 US](https://www.english-corpora.org/corona/x4.asp?rs=&t=34122511&ID=24267236606) | [washingtonpost.com](https://www.bostonglobe.com/2022/02/18/world/statewide-mask-mandates-being-lifted-across-us-mainland/)[(1)](https://www.english-corpora.org/corona/duplicates1.asp?xx=78&n=1) |  |  |  | The White House told federal agencies Thursday to hold off on reinstating a **coronavirus** **vaccination** mandate for millions of employees, hours after an appeals court rejected an earlier injunction |
| [85](https://www.english-corpora.org/corona/x4.asp?rs=&t=89167329&ID=24422412842) | [22-04-22 US](https://www.english-corpora.org/corona/x4.asp?rs=&t=89167329&ID=24422412842) | [YAHOO!News](https://www.bostonglobe.com/2022/02/18/world/statewide-mask-mandates-being-lifted-across-us-mainland/)[(5)](https://www.english-corpora.org/corona/duplicates1.asp?xx=84&n=5) |  |  |  | in Singapore can take second booster if they wish # People enter a **coronavirus** **vaccination** centre set up at a community centre in Singapore. (AFP via Getty Images |
| [86](https://www.english-corpora.org/corona/x4.asp?rs=&t=90035252&ID=24294544852) | [22-04-10 US](https://www.english-corpora.org/corona/x4.asp?rs=&t=90035252&ID=24294544852) | [washingtonpost.com](https://www.bostonglobe.com/2022/02/18/world/statewide-mask-mandates-being-lifted-across-us-mainland/) |  |  |  | nostril # When Dan Wagner of Cincinnati finally had the chance to get a **coronavirus** **vaccination** last year, he put it off. The 33-year-old runs an online business selling |
| [88](https://www.english-corpora.org/corona/x4.asp?rs=&t=90092169&ID=24323046805) | [22-04-13 US](https://www.english-corpora.org/corona/x4.asp?rs=&t=90092169&ID=24323046805) | [hindustantimes.com](https://www.bostonglobe.com/2022/02/18/world/statewide-mask-mandates-being-lifted-across-us-mainland/)[(1)](https://www.english-corpora.org/corona/duplicates1.asp?xx=87&n=1) |  |  |  | 1 to August 31, the public will no longer need to present **coronavirus** **vaccination** or illness certificates to enter indoor or open spaces such as restaurants, and authorities |
| [94](https://www.english-corpora.org/corona/x4.asp?rs=&t=74313026&ID=24237827411) | [22-04-06 AU](https://www.english-corpora.org/corona/x4.asp?rs=&t=74313026&ID=24237827411) | [afr.com](https://www.bostonglobe.com/2022/02/18/world/statewide-mask-mandates-being-lifted-across-us-mainland/)[(5)](https://www.english-corpora.org/corona/duplicates1.asp?xx=93&n=5) |  |  |  | hand it back, and keep a smaller amount on you. **Coronavirus** **vaccination** cards # You'll want to have your vaccination records on you at all times |
| [95](https://www.english-corpora.org/corona/x4.asp?rs=&t=90226111&ID=24397403295) | [22-04-20 AU](https://www.english-corpora.org/corona/x4.asp?rs=&t=90226111&ID=24397403295) | [watoday.com.au](https://www.bostonglobe.com/2022/02/18/world/statewide-mask-mandates-being-lifted-across-us-mainland/) |  |  |  | rejected dozens of challenges from workers who were sacked after refusing to disclose their **coronavirus** **vaccination** status or telling their employer they were not jabbed, even as vaccine mandates look |
| [98](https://www.english-corpora.org/corona/x4.asp?rs=&t=34096504&ID=24168041750) | [22-04-01 NZ](https://www.english-corpora.org/corona/x4.asp?rs=&t=34096504&ID=24168041750) | [odt.co.nz](https://www.bostonglobe.com/2022/02/18/world/statewide-mask-mandates-being-lifted-across-us-mainland/)[(2)](https://www.english-corpora.org/corona/duplicates1.asp?xx=97&n=2) |  |  |  | in Tasmania have been sacked for failing to comply with the state's **coronavirus** **vaccination** mandate. # The Tasmanian government implemented the mandate from October 31, requiring healthcare |
| [99](https://www.english-corpora.org/corona/x4.asp?rs=&t=90150101&ID=24349243044) | [22-04-16 IN](https://www.english-corpora.org/corona/x4.asp?rs=&t=90150101&ID=24349243044) | [economictimes.indiatimes.com](https://www.bostonglobe.com/2022/02/18/world/statewide-mask-mandates-being-lifted-across-us-mainland/) |  |  |  | crore # Union Ministry of Health and Family Welfare informed that India's cumulative **Coronavirus** **vaccination** coverage crossed 186.38 crores on Saturday, as per the provisional reports by 7 am |
| [100](https://www.english-corpora.org/corona/x4.asp?rs=&t=90262824&ID=24414767131) | [22-04-21 IN](https://www.english-corpora.org/corona/x4.asp?rs=&t=90262824&ID=24414767131) | [timesnownews.com](https://www.bostonglobe.com/2022/02/18/world/statewide-mask-mandates-being-lifted-across-us-mainland/) |  |  |  | all eligible beneficiaries between the 18 and 59 yearsof age group in all government **coronavirus** **vaccination** centres. # The decision comes as Delhi witnessed the latest spike of more than |
| [101](https://www.english-corpora.org/corona/x4.asp?rs=&t=90382147&ID=24474197821) | [22-04-26 PH](https://www.english-corpora.org/corona/x4.asp?rs=&t=90382147&ID=24474197821) | [philstar.com](https://www.bostonglobe.com/2022/02/18/world/statewide-mask-mandates-being-lifted-across-us-mainland/) |  |  |  | Duterte's COVID-19 response -- OCTA # Residents queue up at a Covid-19 **coronavirus** **vaccination** centre in Mandaluyong City, suburban Manila on July 15, 2021. # AFP |

| PAGE:   [**<<**](https://www.english-corpora.org/corona/x3.asp?node=&p=1&w10=coronavirus&w11=vaccination&r=) [**<**](https://www.english-corpora.org/corona/x3.asp?node=&p=1&w10=coronavirus&w11=vaccination&r=)   1 / 42  [**>**](https://www.english-corpora.org/corona/x3.asp?node=&p=2&w10=coronavirus&w11=vaccination&r=) [**>>**](https://www.english-corpora.org/corona/x3.asp?node=&p=42&w10=coronavirus&w11=vaccination&r=) |
| --- |

Конец формы

| CLICK FOR MORE CONTEXT | [**HELP**](javascript:newFeatures()) | [SAVE](javascript:chooser('s'))    [TRANSLATE](javascript:chooser('t'))    [ANALYZE](javascript:chooser('p')) |
| --- | --- | --- |

| [101](https://www.english-corpora.org/corona/x4.asp?rs=&t=33647111&ID=23092456609) | [22-01-20 US](https://www.english-corpora.org/corona/x4.asp?rs=&t=33647111&ID=23092456609) | [arkansasonline.com](https://www.english-corpora.org/corona/x3.asp?node=&p=2&w10=coronavirus&w11=vaccination&r=) |  |  |  | Human Services Secretary Cindy Gillespie asked for an exemption from state law to implement **coronavirus** **vaccination** requirements at seven of the agency's facilities across Arkansas. # " This request |
| --- | --- | --- | --- | --- | --- | --- |
| [102](https://www.english-corpora.org/corona/x4.asp?rs=&t=33649592&ID=23102800211) | [22-01-20 US](https://www.english-corpora.org/corona/x4.asp?rs=&t=33649592&ID=23102800211) | [news.yahoo.com](https://www.english-corpora.org/corona/x3.asp?node=&p=2&w10=coronavirus&w11=vaccination&r=) |  |  |  | The White House is considering requiring migrants aged 5 and older to receive a **coronavirus** **vaccination** as a condition for crossing the U.S.-Mexico border to await court hearings, Axios has |
| [103](https://www.english-corpora.org/corona/x4.asp?rs=&t=33677301&ID=23187125057) | [22-01-26 US](https://www.english-corpora.org/corona/x4.asp?rs=&t=33677301&ID=23187125057) | [journalstar.com](https://www.english-corpora.org/corona/x3.asp?node=&p=2&w10=coronavirus&w11=vaccination&r=) |  |  |  | Gov. Kathy Hochul signed a law that made it clear that forging a **coronavirus** **vaccination** record would fall under the fraud statute. # Flynn told reporters Wednesday that the |
| [104](https://www.english-corpora.org/corona/x4.asp?rs=&t=33679226&ID=23187483898) | [22-01-26 US](https://www.english-corpora.org/corona/x4.asp?rs=&t=33679226&ID=23187483898) | [sfgate.com](https://www.english-corpora.org/corona/x3.asp?node=&p=2&w10=coronavirus&w11=vaccination&r=) |  |  |  | above 50, or no mandate at all.Markus Schreiber/APShow MoreShow Less5of18A man enters a **coronavirus** **vaccination** center in Duisburg, Germany, Tuesday, Jan. 25, 2022. Germany's |
| [105](https://www.english-corpora.org/corona/x4.asp?rs=&t=33679226&ID=23187484044) | [22-01-26 US](https://www.english-corpora.org/corona/x4.asp?rs=&t=33679226&ID=23187484044) | [sfgate.com](https://www.english-corpora.org/corona/x3.asp?node=&p=2&w10=coronavirus&w11=vaccination&r=) |  |  |  | everyone above 50, or no mandate at all.Markus Schreiber/APShow MoreShow Less8of18Families enter a **coronavirus** **vaccination** center for children in Duisburg, Germany, Tuesday, Jan. 25, 2022. |
| [106](https://www.english-corpora.org/corona/x4.asp?rs=&t=43875392&ID=23122014706) | [22-01-21 US](https://www.english-corpora.org/corona/x4.asp?rs=&t=43875392&ID=23122014706) | [cnn.com](https://www.english-corpora.org/corona/x3.asp?node=&p=2&w10=coronavirus&w11=vaccination&r=) |  |  |  | the second service available via that hotline, which also provides information on local **coronavirus** **vaccination** sites. # The expanded hotline began accepting calls on Friday morning, days after |
| [107](https://www.english-corpora.org/corona/x4.asp?rs=&t=43886950&ID=23155011901) | [22-01-24 US](https://www.english-corpora.org/corona/x4.asp?rs=&t=43886950&ID=23155011901) | [patch.com](https://www.english-corpora.org/corona/x3.asp?node=&p=2&w10=coronavirus&w11=vaccination&r=) |  |  |  | those elibible. # SWAMPSCOTT, MA -- Swampscott students in need of a **coronavirus** **vaccination** and residents eligible for a booster shot are invited to get their next dose at |
| [108](https://www.english-corpora.org/corona/x4.asp?rs=&t=43897849&ID=23192325828) | [22-01-26 US](https://www.english-corpora.org/corona/x4.asp?rs=&t=43897849&ID=23192325828) | [fox6now.com](https://www.english-corpora.org/corona/x3.asp?node=&p=2&w10=coronavirus&w11=vaccination&r=) |  |  |  | in South Carolina introduced legislation to make it illegal for employers to ask about **coronavirus** **vaccination** status, saying it's private medical information. # The bill's author said |
| [109](https://www.english-corpora.org/corona/x4.asp?rs=&t=63798857&ID=23175703404) | [22-01-25 US](https://www.english-corpora.org/corona/x4.asp?rs=&t=63798857&ID=23175703404) | [fox2now.com](https://www.english-corpora.org/corona/x3.asp?node=&p=2&w10=coronavirus&w11=vaccination&r=) |  |  |  | state House committee on Tuesday heard more than a dozen bills aimed at stopping **coronavirus** **vaccination** requirements imposed by businesses. But the proposals are splitting Republicans and the business community |
| [110](https://www.english-corpora.org/corona/x4.asp?rs=&t=73992048&ID=23273235670) | [22-01-31 US](https://www.english-corpora.org/corona/x4.asp?rs=&t=73992048&ID=23273235670) | [theweek.com](https://www.english-corpora.org/corona/x3.asp?node=&p=2&w10=coronavirus&w11=vaccination&r=) |  |  |  | Australia kicked him out due to his failure to comply with the country's **coronavirus** **vaccination** rules. North Korea confirms test of missile that could reach Guam # North |
| [111](https://www.english-corpora.org/corona/x4.asp?rs=&t=88659327&ID=23107885906) | [22-01-20 US](https://www.english-corpora.org/corona/x4.asp?rs=&t=88659327&ID=23107885906) | [Yahoo](https://www.english-corpora.org/corona/x3.asp?node=&p=2&w10=coronavirus&w11=vaccination&r=) |  |  |  | The White House is considering requiring migrants aged 5 and older to receive a **coronavirus** **vaccination** as a condition for crossing the U.S.-Mexico border to await court hearings, Axios has |
| [112](https://www.english-corpora.org/corona/x4.asp?rs=&t=88660099&ID=23111725884) | [22-01-20 US](https://www.english-corpora.org/corona/x4.asp?rs=&t=88660099&ID=23111725884) | [Washington Post](https://www.english-corpora.org/corona/x3.asp?node=&p=2&w10=coronavirus&w11=vaccination&r=) |  |  |  | , started discussions with its labor unions on the mandate and begun revising its **coronavirus** **vaccination**, testing and face-covering policy. # Story continues below advertisement # He said the |
| [113](https://www.english-corpora.org/corona/x4.asp?rs=&t=88669211&ID=23137350085) | [22-01-22 US](https://www.english-corpora.org/corona/x4.asp?rs=&t=88669211&ID=23137350085) | [The Independent on MSN.com](https://www.english-corpora.org/corona/x3.asp?node=&p=2&w10=coronavirus&w11=vaccination&r=) |  |  |  | 08:40, Liam James # The government is considering pausing its plans to make **coronavirus** **vaccination** mandatory for NHS staff, according to a report, over fears that some 70,000 |
| [114](https://www.english-corpora.org/corona/x4.asp?rs=&t=88671971&ID=23140138543) | [22-01-22 US](https://www.english-corpora.org/corona/x4.asp?rs=&t=88671971&ID=23140138543) | [Reuters](https://www.english-corpora.org/corona/x3.asp?node=&p=2&w10=coronavirus&w11=vaccination&r=) |  |  |  | due to problems with the medical exemption from the country's immigration requirement for **coronavirus** **vaccination** that he presented. # The drama has caused tensions between Serbia and Australia and |
| [115](https://www.english-corpora.org/corona/x4.asp?rs=&t=88673168&ID=23147265552) | [22-01-23 US](https://www.english-corpora.org/corona/x4.asp?rs=&t=88673168&ID=23147265552) | [New York Times](https://www.english-corpora.org/corona/x3.asp?node=&p=2&w10=coronavirus&w11=vaccination&r=) |  |  |  | to a patchwork of state and private efforts to track inoculations and the paper **coronavirus** **vaccination** record cards that can be lost or counterfeited. # Image # Vaccines had been |
| [116](https://www.english-corpora.org/corona/x4.asp?rs=&t=88676408&ID=23144844125) | [22-01-23 US](https://www.english-corpora.org/corona/x4.asp?rs=&t=88676408&ID=23144844125) | [New York Times](https://www.english-corpora.org/corona/x3.asp?node=&p=2&w10=coronavirus&w11=vaccination&r=) |  |  |  | daily parsing of Rodgers's word and deed, his status in everything from **coronavirus** **vaccination** to job satisfaction the dominant plot point in the melodrama that is America's most |
| [117](https://www.english-corpora.org/corona/x4.asp?rs=&t=88676496&ID=23147185688) | [22-01-23 US](https://www.english-corpora.org/corona/x4.asp?rs=&t=88676496&ID=23147185688) | [Washington Post](https://www.english-corpora.org/corona/x3.asp?node=&p=2&w10=coronavirus&w11=vaccination&r=) |  |  |  | Mordkin recommends confirming with your physician that you are still not a candidate for **coronavirus** **vaccination**, as it remains the most effective defense against severe illness or death from a |
| [118](https://www.english-corpora.org/corona/x4.asp?rs=&t=88677159&ID=23146156227) | [22-01-23 US](https://www.english-corpora.org/corona/x4.asp?rs=&t=88677159&ID=23146156227) | [Fox News](https://www.english-corpora.org/corona/x3.asp?node=&p=2&w10=coronavirus&w11=vaccination&r=) |  |  |  | Gonzaga's rules say fans 12 and older are required to provide proof of **coronavirus** **vaccination** or a negative PCR test 72 hours before the start of a game. Masks |
| [119](https://www.english-corpora.org/corona/x4.asp?rs=&t=88680110&ID=23163263831) | [22-01-24 US](https://www.english-corpora.org/corona/x4.asp?rs=&t=88680110&ID=23163263831) | [Yahoo](https://www.english-corpora.org/corona/x3.asp?node=&p=2&w10=coronavirus&w11=vaccination&r=) |  |  |  | Monday # 11:05, Elly Blake # Prime Minister Boris Johnson has visited a **coronavirus** **vaccination** training hub at Milton Keynes University Hospital in Buckinghamshire on Monday. # (REUTERS |
| [120](https://www.english-corpora.org/corona/x4.asp?rs=&t=88704177&ID=23237990076) | [22-01-28 US](https://www.english-corpora.org/corona/x4.asp?rs=&t=88704177&ID=23237990076) | [The Washington Post on MSN.com](https://www.english-corpora.org/corona/x3.asp?node=&p=2&w10=coronavirus&w11=vaccination&r=) |  |  |  | # A number of major Virginia universities and colleges require students to have a **coronavirus** **vaccination**, including the University of Virginia, Virginia Tech, George Mason University, James |
| [121](https://www.english-corpora.org/corona/x4.asp?rs=&t=88706254&ID=23236021196) | [22-01-28 US](https://www.english-corpora.org/corona/x4.asp?rs=&t=88706254&ID=23236021196) | [The Washington Post on MSN.com](https://www.english-corpora.org/corona/x3.asp?node=&p=2&w10=coronavirus&w11=vaccination&r=) |  |  |  | four-year, public universities and colleges in the commonwealth require students to get a **coronavirus** **vaccination**. The schools include the University of Virginia, Virginia Tech, James Madison University |
| [122](https://www.english-corpora.org/corona/x4.asp?rs=&t=88707364&ID=23236099225) | [22-01-28 US](https://www.english-corpora.org/corona/x4.asp?rs=&t=88707364&ID=23236099225) | [The Boston Globe on MSN.com](https://www.english-corpora.org/corona/x3.asp?node=&p=2&w10=coronavirus&w11=vaccination&r=) |  |  |  | suspended from working in the sport after it was ruled Wednesday they used fake **coronavirus** **vaccination** records. # A disciplinary panel at the German soccer federation ruled former Werder Bremen |
| [123](https://www.english-corpora.org/corona/x4.asp?rs=&t=88715573&ID=23261424374) | [22-01-30 US](https://www.english-corpora.org/corona/x4.asp?rs=&t=88715573&ID=23261424374) | [New York Times](https://www.english-corpora.org/corona/x3.asp?node=&p=2&w10=coronavirus&w11=vaccination&r=) |  |  |  | December 2020 became the first country in the world to kick off a mass **coronavirus** **vaccination** campaign, but expanding the program to children has happened at a slower pace. |
| [124](https://www.english-corpora.org/corona/x4.asp?rs=&t=88717646&ID=23280967115) | [22-01-31 US](https://www.english-corpora.org/corona/x4.asp?rs=&t=88717646&ID=23280967115) | [Yahoo! Sports](https://www.english-corpora.org/corona/x3.asp?node=&p=2&w10=coronavirus&w11=vaccination&r=) |  |  |  | crimes linked to the demonstrations. Several facilities, including City Hall and a **coronavirus** **vaccination** clinic, were closed. The Rideau Center, a major shopping mall, was |
| [125](https://www.english-corpora.org/corona/x4.asp?rs=&t=88720752&ID=23280508795) | [22-01-31 US](https://www.english-corpora.org/corona/x4.asp?rs=&t=88720752&ID=23280508795) | [NJ.com](https://www.english-corpora.org/corona/x3.asp?node=&p=2&w10=coronavirus&w11=vaccination&r=) |  |  |  | , even if her job depends on it. # " I was for **coronavirus** **vaccination** because I do not want to wear these masks everywhere for the rest of my |
| [126](https://www.english-corpora.org/corona/x4.asp?rs=&t=53549539&ID=23099337138) | [22-01-20 CA](https://www.english-corpora.org/corona/x4.asp?rs=&t=53549539&ID=23099337138) | [canadianinquirer.net](https://www.english-corpora.org/corona/x3.asp?node=&p=2&w10=coronavirus&w11=vaccination&r=) |  |  |  | MANILA -- The Department of Education (DepEd) said Thursday its requirement of **coronavirus** **vaccination** for teaching and non-teaching personnel who will participate in limited face-to-face classes is to prevent |
| [127](https://www.english-corpora.org/corona/x4.asp?rs=&t=53559539&ID=23133546006) | [22-01-22 CA](https://www.english-corpora.org/corona/x4.asp?rs=&t=53559539&ID=23133546006) | [o.canada.com](https://www.english-corpora.org/corona/x3.asp?node=&p=2&w10=coronavirus&w11=vaccination&r=) |  |  |  | a particular line of conduct. Effectively, a faith-based calling to decline the **coronavirus** **vaccination**. " # Fu says there are " a very limited " number of religions |
| [128](https://www.english-corpora.org/corona/x4.asp?rs=&t=53559539&ID=23133546156) | [22-01-22 CA](https://www.english-corpora.org/corona/x4.asp?rs=&t=53559539&ID=23133546156) | [o.canada.com](https://www.english-corpora.org/corona/x3.asp?node=&p=2&w10=coronavirus&w11=vaccination&r=) |  |  |  | that this person is following this faith and it's not just limited to **coronavirus** **vaccination**. " This Week in Flyers Comments # Postmedia is committed to maintaining |
| [129](https://www.english-corpora.org/corona/x4.asp?rs=&t=53573975&ID=23196695125) | [22-01-26 CA](https://www.english-corpora.org/corona/x4.asp?rs=&t=53573975&ID=23196695125) | [sportsnet.ca](https://www.english-corpora.org/corona/x3.asp?node=&p=2&w10=coronavirus&w11=vaccination&r=) |  |  |  | suspended from working in the sport after it was ruled Wednesday they used fake **coronavirus** **vaccination** records. # A disciplinary panel at the German soccer federation ruled former Werder Bremen |
| [130](https://www.english-corpora.org/corona/x4.asp?rs=&t=53578827&ID=23205932393) | [22-01-27 CA](https://www.english-corpora.org/corona/x4.asp?rs=&t=53578827&ID=23205932393) | [ca.news.yahoo.com](https://www.english-corpora.org/corona/x3.asp?node=&p=2&w10=coronavirus&w11=vaccination&r=) |  |  |  | suspended from working in the sport after it was ruled Wednesday they used fake **coronavirus** **vaccination** records. A disciplinary panel at the German soccer federation ruled former Werder Bremen head |
| [131](https://www.english-corpora.org/corona/x4.asp?rs=&t=73973443&ID=23212215880) | [22-01-27 CA](https://www.english-corpora.org/corona/x4.asp?rs=&t=73973443&ID=23212215880) | [infotel.ca](https://www.english-corpora.org/corona/x3.asp?node=&p=2&w10=coronavirus&w11=vaccination&r=) |  |  |  | vaccine doses, distributed American antibiotics to pilgrims and controlled the spread. # **Coronavirus** **vaccination** marks the nation's first mass inoculation campaign outside of childhood illnesses since the 1979 |
| [132](https://www.english-corpora.org/corona/x4.asp?rs=&t=33656357&ID=23117148576) | [22-01-21 GB](https://www.english-corpora.org/corona/x4.asp?rs=&t=33656357&ID=23117148576) | [eurosport.co.uk](https://www.english-corpora.org/corona/x3.asp?node=&p=2&w10=coronavirus&w11=vaccination&r=) |  |  |  | The world No. 1 was sent back to Serbia because of his stance on **coronavirus** **vaccination**, but there are now questions about whether other countries will adopt the same approach |
| [133](https://www.english-corpora.org/corona/x4.asp?rs=&t=33656357&ID=23117148639) | [22-01-21 GB](https://www.english-corpora.org/corona/x4.asp?rs=&t=33656357&ID=23117148639) | [eurosport.co.uk](https://www.english-corpora.org/corona/x3.asp?node=&p=2&w10=coronavirus&w11=vaccination&r=) |  |  |  | react to him going forward. # The world No. 1's stance on **coronavirus** **vaccination** eventually caught up with him just days before he was due to start the defence |
| [134](https://www.english-corpora.org/corona/x4.asp?rs=&t=33666819&ID=23148709362) | [22-01-24 GB](https://www.english-corpora.org/corona/x4.asp?rs=&t=33666819&ID=23148709362) | [eurosport.co.uk](https://www.english-corpora.org/corona/x3.asp?node=&p=2&w10=coronavirus&w11=vaccination&r=) |  |  |  | to challenge for more major titles if he does not change his stance on **coronavirus** **vaccination**. # He described the 20-time Grand Slam champion's troubles - and deportation - |
| [135](https://www.english-corpora.org/corona/x4.asp?rs=&t=43870948&ID=23117196050) | [22-01-21 GB](https://www.english-corpora.org/corona/x4.asp?rs=&t=43870948&ID=23117196050) | [independent.co.uk](https://www.english-corpora.org/corona/x3.asp?node=&p=2&w10=coronavirus&w11=vaccination&r=) |  |  |  | # (PA) # The government is considering pausing its plans to make **coronavirus** **vaccination** mandatory for NHS staff, according to a report, over fears that some 70,000 |
| [136](https://www.english-corpora.org/corona/x4.asp?rs=&t=43893702&ID=23187560607) | [22-01-26 GB](https://www.english-corpora.org/corona/x4.asp?rs=&t=43893702&ID=23187560607) | [heraldscotland.com](https://www.english-corpora.org/corona/x3.asp?node=&p=2&w10=coronavirus&w11=vaccination&r=) |  |  |  | # A spokesman added: " Whilst we strongly encourage those eligible to receive **coronavirus** **vaccination** to do so, the decision is one of personal choice and the Scottish Government |
| [137](https://www.english-corpora.org/corona/x4.asp?rs=&t=43899241&ID=23209189595) | [22-01-27 GB](https://www.english-corpora.org/corona/x4.asp?rs=&t=43899241&ID=23209189595) | [heraldscotland.com](https://www.english-corpora.org/corona/x3.asp?node=&p=2&w10=coronavirus&w11=vaccination&r=) |  |  |  | , with someone who has a weakened immune system, are being offered the **coronavirus** **vaccination**. # Absences among Scottish teachers also appear to be on the rise. Nearly |
| [138](https://www.english-corpora.org/corona/x4.asp?rs=&t=43900431&ID=23204908506) | [22-01-27 GB](https://www.english-corpora.org/corona/x4.asp?rs=&t=43900431&ID=23204908506) | [yorkpress.co.uk](https://www.english-corpora.org/corona/x3.asp?node=&p=2&w10=coronavirus&w11=vaccination&r=) |  |  |  | (RCOG) has called for the Government to reconsider introducing regulations on mandatory **coronavirus** **vaccination** due to the impact of staff shortages on pregnant mothers. # Frontline staff in |
| [140](https://www.english-corpora.org/corona/x4.asp?rs=&t=88715525&ID=23259217811) | [22-01-30 GB](https://www.english-corpora.org/corona/x4.asp?rs=&t=88715525&ID=23259217811) | [The Scotsman](https://www.english-corpora.org/corona/x3.asp?node=&p=2&w10=coronavirus&w11=vaccination&r=)[(1)](https://www.english-corpora.org/corona/duplicates1.asp?xx=139&n=1) |  |  |  | to depart from Melbourne Airport after losing a sensational legal battle over his **coronavirus** **vaccination** status with his dream of clinching a record 21st Grand Slam in tatters. ( |
| [141](https://www.english-corpora.org/corona/x4.asp?rs=&t=63780526&ID=23095938049) | [22-01-20 IE](https://www.english-corpora.org/corona/x4.asp?rs=&t=63780526&ID=23095938049) | [trtworld.com](https://www.english-corpora.org/corona/x3.asp?node=&p=2&w10=coronavirus&w11=vaccination&r=) |  |  |  | the world with 18 million cases last week, people in countries with low **coronavirus** **vaccination** rates are at risk of severe illness and death, the head of the World |
| [142](https://www.english-corpora.org/corona/x4.asp?rs=&t=73982417&ID=23252932344) | [22-01-30 AU](https://www.english-corpora.org/corona/x4.asp?rs=&t=73982417&ID=23252932344) | [au.finance.yahoo.com](https://www.english-corpora.org/corona/x3.asp?node=&p=2&w10=coronavirus&w11=vaccination&r=) |  |  |  | as ministers made a fresh push for people to come forward for their third **coronavirus** **vaccination** dose. # Speaking to broadcasters during a visit to Northamptonshire, the Prime Minister |
| [143](https://www.english-corpora.org/corona/x4.asp?rs=&t=73943206&ID=23096378381) | [22-01-20 IN](https://www.english-corpora.org/corona/x4.asp?rs=&t=73943206&ID=23096378381) | [business-standard.com](https://www.english-corpora.org/corona/x3.asp?node=&p=2&w10=coronavirus&w11=vaccination&r=) |  |  |  | Council, the parliament's lower house, voted on Thursday to make **coronavirus** **vaccination** mandatory from February. # A simple majority of deputies of only two ruling parties |
| [144](https://www.english-corpora.org/corona/x4.asp?rs=&t=73944852&ID=23101470575) | [22-01-20 IN](https://www.english-corpora.org/corona/x4.asp?rs=&t=73944852&ID=23101470575) | [republicworld.com](https://www.english-corpora.org/corona/x3.asp?node=&p=2&w10=coronavirus&w11=vaccination&r=) |  |  |  | government will move to the 12-14 age group after it is done with the **Coronavirus** **vaccination** in the 15-18 group. # " The age will keep coming down. The |
| [145](https://www.english-corpora.org/corona/x4.asp?rs=&t=53585487&ID=23231138853) | [22-01-28 PK](https://www.english-corpora.org/corona/x4.asp?rs=&t=53585487&ID=23231138853) | [thenews.com.pk](https://www.english-corpora.org/corona/x3.asp?node=&p=2&w10=coronavirus&w11=vaccination&r=) |  |  |  | , home of the US Open, also under stringent virus rules. # **Coronavirus** **vaccination** is not a requirement to enter the United Arab Emirates, where 100 percent of |
| [146](https://www.english-corpora.org/corona/x4.asp?rs=&t=53552763&ID=23118030252) | [22-01-21 BD](https://www.english-corpora.org/corona/x4.asp?rs=&t=53552763&ID=23118030252) | [bdnews24.com](https://www.english-corpora.org/corona/x3.asp?node=&p=2&w10=coronavirus&w11=vaccination&r=) |  |  |  | two weeks, the authorities have now made it mandatory for everyone to show **coronavirus** **vaccination** certificates to enter the venue. # Bangla Academy Director General Muhammad Nurul Huda informed |
| [147](https://www.english-corpora.org/corona/x4.asp?rs=&t=73944335&ID=23101385909) | [22-01-20 PH](https://www.english-corpora.org/corona/x4.asp?rs=&t=73944335&ID=23101385909) | [pageone.ph](https://www.english-corpora.org/corona/x3.asp?node=&p=2&w10=coronavirus&w11=vaccination&r=) |  |  |  | in the province. # The Department of Education said Thursday its requirement of **coronavirus** **vaccination** for teaching and non-teaching personnel who will participate in limited face-to-face classes is to prevent |
| [150](https://www.english-corpora.org/corona/x4.asp?rs=&t=43875207&ID=23113608872) | [22-01-21 ZA](https://www.english-corpora.org/corona/x4.asp?rs=&t=43875207&ID=23113608872) | [northcoastcourier.co.za](https://www.english-corpora.org/corona/x3.asp?node=&p=2&w10=coronavirus&w11=vaccination&r=)[(2)](https://www.english-corpora.org/corona/duplicates1.asp?xx=149&n=2) |  |  |  | between Djokovic and Australia quickly became symbolic of the battle between supporters of **coronavirus** **vaccination** and restrictions on travel and those who oppose them. # Tennis star Novak Djokovic |
| [152](https://www.english-corpora.org/corona/x4.asp?rs=&t=53554896&ID=23122600339) | [22-01-21 NG](https://www.english-corpora.org/corona/x4.asp?rs=&t=53554896&ID=23122600339) | [guardian.ng](https://www.english-corpora.org/corona/x3.asp?node=&p=2&w10=coronavirus&w11=vaccination&r=)[(1)](https://www.english-corpora.org/corona/duplicates1.asp?xx=151&n=1) |  |  |  | restaurants, bars or shopping malls. Brazil had a late start with **coronavirus** **vaccination** but is now the country with the fourth-most doses administered. # Covid-19 has caused |
| [153](https://www.english-corpora.org/corona/x4.asp?rs=&t=33703377&ID=23291951648) | [22-02-01 US](https://www.english-corpora.org/corona/x4.asp?rs=&t=33703377&ID=23291951648) | [bostonherald.com](https://www.english-corpora.org/corona/x3.asp?node=&p=2&w10=coronavirus&w11=vaccination&r=) |  |  |  | that many businesses need to require patrons to mask up and provide proof of **coronavirus** **vaccination**, Wu said she did not. # Wu said the Boston Public Health Commission |
| [154](https://www.english-corpora.org/corona/x4.asp?rs=&t=33706131&ID=23283592703) | [22-02-01 US](https://www.english-corpora.org/corona/x4.asp?rs=&t=33706131&ID=23283592703) | [news.yahoo.com](https://www.english-corpora.org/corona/x3.asp?node=&p=2&w10=coronavirus&w11=vaccination&r=) |  |  |  | and boosted, according to a new study that further highlights the importance of **coronavirus** **vaccination** and booster shots. The study, released Tuesday, from the Centers for Disease |
| [155](https://www.english-corpora.org/corona/x4.asp?rs=&t=33713305&ID=23317620869) | [22-02-03 US](https://www.english-corpora.org/corona/x4.asp?rs=&t=33713305&ID=23317620869) | [theguardian.com](https://www.english-corpora.org/corona/x3.asp?node=&p=2&w10=coronavirus&w11=vaccination&r=) |  |  |  | has dropped by more than 17,000 since the government launched its consultation on mandatory **coronavirus** **vaccination**, figures suggest. There were 406,365 domiciliary care staff in registered settings reported by |
| [156](https://www.english-corpora.org/corona/x4.asp?rs=&t=33720509&ID=23336354301) | [22-02-04 US](https://www.english-corpora.org/corona/x4.asp?rs=&t=33720509&ID=23336354301) | [stltoday.com](https://www.english-corpora.org/corona/x3.asp?node=&p=2&w10=coronavirus&w11=vaccination&r=) |  |  |  | spread the virus # Two nurses in New York are being charged with forging **coronavirus** **vaccination** cards and entering the fake vaccinations into the state's database. Julie DeVuono, |
| [157](https://www.english-corpora.org/corona/x4.asp?rs=&t=33720509&ID=23336354423) | [22-02-04 US](https://www.english-corpora.org/corona/x4.asp?rs=&t=33720509&ID=23336354423) | [stltoday.com](https://www.english-corpora.org/corona/x3.asp?node=&p=2&w10=coronavirus&w11=vaccination&r=) |  |  |  | , New York Gov. Kathy Hochul signed a bill into law criminalizing fake **coronavirus** **vaccination** cards. # Medical personnel are beyond the breaking point with coronavirus cases. Four |
| [158](https://www.english-corpora.org/corona/x4.asp?rs=&t=33732412&ID=23373334420) | [22-02-07 US](https://www.english-corpora.org/corona/x4.asp?rs=&t=33732412&ID=23373334420) | [foxnews.com](https://www.english-corpora.org/corona/x3.asp?node=&p=2&w10=coronavirus&w11=vaccination&r=) |  |  |  | South Carolina introduced legislation to make it illegal for employers to ask about **coronavirus** **vaccination** status, saying it's private medical information. # The bill's author said |
| [159](https://www.english-corpora.org/corona/x4.asp?rs=&t=33747512&ID=23429829758) | [22-02-11 US](https://www.english-corpora.org/corona/x4.asp?rs=&t=33747512&ID=23429829758) | [abcnews.go.com](https://www.english-corpora.org/corona/x3.asp?node=&p=2&w10=coronavirus&w11=vaccination&r=) |  |  |  | # Health authorities in Denmark are considering " winding down " the country's **coronavirus** **vaccination** program in the spring and see no reason now to administer a booster dose to |
| [161](https://www.english-corpora.org/corona/x4.asp?rs=&t=33760033&ID=23474382582) | [22-02-14 US](https://www.english-corpora.org/corona/x4.asp?rs=&t=33760033&ID=23474382582) | [nytimes.com](https://www.english-corpora.org/corona/x3.asp?node=&p=2&w10=coronavirus&w11=vaccination&r=)[(1)](https://www.english-corpora.org/corona/duplicates1.asp?xx=160&n=1) |  |  |  | and be free. " Advertisement # Waiting in line at a **coronavirus** **vaccination** site at Stockholm City Terminal station in Sweden last month.Credit... Anders Wiklund/TT News Agency |
| [162](https://www.english-corpora.org/corona/x4.asp?rs=&t=33760951&ID=23479435103) | [22-02-14 US](https://www.english-corpora.org/corona/x4.asp?rs=&t=33760951&ID=23479435103) | [stltoday.com](https://www.english-corpora.org/corona/x3.asp?node=&p=2&w10=coronavirus&w11=vaccination&r=) |  |  |  | but in fact it's the logical outcome of the irrational conservative backlash against **coronavirus** **vaccination** mandates. # Vaccines have saved an incalculable number of lives in the past century |
| [163](https://www.english-corpora.org/corona/x4.asp?rs=&t=33766859&ID=23499072419) | [22-02-15 US](https://www.english-corpora.org/corona/x4.asp?rs=&t=33766859&ID=23499072419) | [euronews.com](https://www.english-corpora.org/corona/x3.asp?node=&p=2&w10=coronavirus&w11=vaccination&r=) |  |  |  | restrictions, while also considering whether to " wind down " the country's **coronavirus** **vaccination** programme. # " The very high vaccine coverage in Denmark, especially with the |
| [164](https://www.english-corpora.org/corona/x4.asp?rs=&t=43924409&ID=23284224806) | [22-02-01 US](https://www.english-corpora.org/corona/x4.asp?rs=&t=43924409&ID=23284224806) | [whnt.com](https://www.english-corpora.org/corona/x3.asp?node=&p=2&w10=coronavirus&w11=vaccination&r=) |  |  |  | . A sprawling, lumbering winter storm walloped the Eastern U.S., shutting down **coronavirus** **vaccination** sites, closing schools and halting transit. The U.S. government said it would not |
| [165](https://www.english-corpora.org/corona/x4.asp?rs=&t=43924409&ID=23284224828) | [22-02-01 US](https://www.english-corpora.org/corona/x4.asp?rs=&t=43924409&ID=23284224828) | [whnt.com](https://www.english-corpora.org/corona/x3.asp?node=&p=2&w10=coronavirus&w11=vaccination&r=) |  |  |  | transit. The U.S. government said it would not conduct immigration enforcement arrests at **coronavirus** **vaccination** sites around the country. Former AFL-CIO President John Sweeney died at age 86 at |
| [166](https://www.english-corpora.org/corona/x4.asp?rs=&t=53608199&ID=23311843547) | [22-02-02 US](https://www.english-corpora.org/corona/x4.asp?rs=&t=53608199&ID=23311843547) | [tallahassee.com](https://www.english-corpora.org/corona/x3.asp?node=&p=2&w10=coronavirus&w11=vaccination&r=) |  |  |  | of her passion for health education, she obtained a grant from Leon County **Coronavirus** **Vaccination** Community Education and Engagement Task Force to educate and decrease hesitancy of the COVID-19 vaccine |
| [167](https://www.english-corpora.org/corona/x4.asp?rs=&t=53659646&ID=23501580842) | [22-02-15 US](https://www.english-corpora.org/corona/x4.asp?rs=&t=53659646&ID=23501580842) | [dailyprogress.com](https://www.english-corpora.org/corona/x3.asp?node=&p=2&w10=coronavirus&w11=vaccination&r=) |  |  |  | Denmark said Friday that they were considering " winding down " the country's **coronavirus** **vaccination** program in the spring and see no reason now to administer a booster dose to |
| [169](https://www.english-corpora.org/corona/x4.asp?rs=&t=88730669&ID=23317352687) | [22-02-02 US](https://www.english-corpora.org/corona/x4.asp?rs=&t=88730669&ID=23317352687) | [The Washington Post on MSN.com](https://www.english-corpora.org/corona/x3.asp?node=&p=2&w10=coronavirus&w11=vaccination&r=)[(1)](https://www.english-corpora.org/corona/duplicates1.asp?xx=168&n=1) |  |  |  | of which include routine childhood immunizations -- all of them opportunities to discuss **coronavirus** **vaccination**. During winter months, youngsters often come in for flu shots. # He |
| [170](https://www.english-corpora.org/corona/x4.asp?rs=&t=88734645&ID=23334987784) | [22-02-03 US](https://www.english-corpora.org/corona/x4.asp?rs=&t=88734645&ID=23334987784) | [The Guardian on MSN.com](https://www.english-corpora.org/corona/x3.asp?node=&p=2&w10=coronavirus&w11=vaccination&r=) |  |  |  | has dropped by more than 17,000 since the government launched its consultation on mandatory **coronavirus** **vaccination**, figures suggest. # There were 406,365 domiciliary care staff in registered settings reported |
| [171](https://www.english-corpora.org/corona/x4.asp?rs=&t=88738404&ID=23350252600) | [22-02-04 US](https://www.english-corpora.org/corona/x4.asp?rs=&t=88738404&ID=23350252600) | [Washington Post](https://www.english-corpora.org/corona/x3.asp?node=&p=2&w10=coronavirus&w11=vaccination&r=) |  |  |  | , while mask mandates go largely unenforced. It also has an especially low **coronavirus** **vaccination** rate, compared with other European nations, leaving much of the population vulnerable to |
| [172](https://www.english-corpora.org/corona/x4.asp?rs=&t=88751759&ID=23414538554) | [22-02-09 US](https://www.english-corpora.org/corona/x4.asp?rs=&t=88751759&ID=23414538554) | [The Guardian on MSN.com](https://www.english-corpora.org/corona/x3.asp?node=&p=2&w10=coronavirus&w11=vaccination&r=) |  |  |  | in New York had previously been required to wear masks or show proof of **coronavirus** **vaccination**. " Given the declining cases, given declining hospitalizations, that is why we |
| [175](https://www.english-corpora.org/corona/x4.asp?rs=&t=88751759&ID=23414543159) | [22-02-09 US](https://www.english-corpora.org/corona/x4.asp?rs=&t=88751759&ID=23414543159) | [The Guardian on MSN.com](https://www.english-corpora.org/corona/x3.asp?node=&p=2&w10=coronavirus&w11=vaccination&r=)[(2)](https://www.english-corpora.org/corona/duplicates1.asp?xx=174&n=2) |  |  |  | at indoor businesses had been required to wear masks or show proof of **coronavirus** **vaccination**, but Hochul said the time had come to lift that " emergency temporary measure |
| [176](https://www.english-corpora.org/corona/x4.asp?rs=&t=88755035&ID=23426850378) | [22-02-10 US](https://www.english-corpora.org/corona/x4.asp?rs=&t=88755035&ID=23426850378) | [YAHOO!News](https://www.english-corpora.org/corona/x3.asp?node=&p=2&w10=coronavirus&w11=vaccination&r=) |  |  |  | a pitch to Los Angeles deputies who may be upset over the county's **coronavirus** **vaccination** policy and the political climate over the Grapevine. # The roughly one-minute video was |
| [178](https://www.english-corpora.org/corona/x4.asp?rs=&t=88769081&ID=23456215221) | [22-02-12 US](https://www.english-corpora.org/corona/x4.asp?rs=&t=88769081&ID=23456215221) | [Washington Post](https://www.english-corpora.org/corona/x3.asp?node=&p=2&w10=coronavirus&w11=vaccination&r=)[(1)](https://www.english-corpora.org/corona/duplicates1.asp?xx=177&n=1) |  |  |  | to use its massive European recovery fund stimulus. He had imposed rigid **coronavirus** **vaccination** rules with minimal political blowback -- all while pushing Italy to become one of the |
| [179](https://www.english-corpora.org/corona/x4.asp?rs=&t=88774208&ID=23471550385) | [22-02-13 US](https://www.english-corpora.org/corona/x4.asp?rs=&t=88774208&ID=23471550385) | [San Diego Union-Tribune](https://www.english-corpora.org/corona/x3.asp?node=&p=2&w10=coronavirus&w11=vaccination&r=) |  |  |  | fortune. Although most of today's travelers are obsessed with carrying proof of **coronavirus** **vaccination** or negative test results, few take any other health information. That's a |
| [180](https://www.english-corpora.org/corona/x4.asp?rs=&t=88775863&ID=23490643025) | [22-02-14 US](https://www.english-corpora.org/corona/x4.asp?rs=&t=88775863&ID=23490643025) | [ABC](https://www.english-corpora.org/corona/x3.asp?node=&p=2&w10=coronavirus&w11=vaccination&r=) |  |  |  | authorities there said that they were considering " winding down " the country's **coronavirus** **vaccination** program in the spring and saw no reason to administer a booster dose to children |
| [181](https://www.english-corpora.org/corona/x4.asp?rs=&t=88776235&ID=23490926761) | [22-02-14 US](https://www.english-corpora.org/corona/x4.asp?rs=&t=88776235&ID=23490926761) | [Patch](https://www.english-corpora.org/corona/x3.asp?node=&p=2&w10=coronavirus&w11=vaccination&r=) |  |  |  | the map below, and click on a pin to see that community's **coronavirus** **vaccination** rate. You can also view the town-by-town coronavirus vaccination data in the spreadsheet we |
| [182](https://www.english-corpora.org/corona/x4.asp?rs=&t=88776235&ID=23490926771) | [22-02-14 US](https://www.english-corpora.org/corona/x4.asp?rs=&t=88776235&ID=23490926771) | [Patch](https://www.english-corpora.org/corona/x3.asp?node=&p=2&w10=coronavirus&w11=vaccination&r=) |  |  |  | see that community's coronavirus vaccination rate. You can also view the town-by-town **coronavirus** **vaccination** data in the spreadsheet we used to create this map. # Colors reflected the |
| [183](https://www.english-corpora.org/corona/x4.asp?rs=&t=88785248&ID=23512153307) | [22-02-15 US](https://www.english-corpora.org/corona/x4.asp?rs=&t=88785248&ID=23512153307) | [Washington Post](https://www.english-corpora.org/corona/x3.asp?node=&p=2&w10=coronavirus&w11=vaccination&r=) |  |  |  | Lewis, 37, said she experienced a severe allergic reaction after her first **coronavirus** **vaccination**. She came from St. Catharines in southern Ontario to stand with the protesters. |
| [184](https://www.english-corpora.org/corona/x4.asp?rs=&t=88794204&ID=23535352090) | [22-02-17 US](https://www.english-corpora.org/corona/x4.asp?rs=&t=88794204&ID=23535352090) | [ABC](https://www.english-corpora.org/corona/x3.asp?node=&p=2&w10=coronavirus&w11=vaccination&r=) |  |  |  | passport system as omicron wanes # Israel's prime minister says the country's **coronavirus** **vaccination** " green pass " system will be suspended as new daily cases of COVID-19 continue |
| [185](https://www.english-corpora.org/corona/x4.asp?rs=&t=88794204&ID=23535352214) | [22-02-17 US](https://www.english-corpora.org/corona/x4.asp?rs=&t=88794204&ID=23535352214) | [ABC](https://www.english-corpora.org/corona/x3.asp?node=&p=2&w10=coronavirus&w11=vaccination&r=) |  |  |  | Naftali Bennett said Thursday, Feb. 17, 2022, that the country's **coronavirus** **vaccination** " green pass " system will be suspended as new daily cases of COVID-19 continue |
| [186](https://www.english-corpora.org/corona/x4.asp?rs=&t=88794204&ID=23535352253) | [22-02-17 US](https://www.english-corpora.org/corona/x4.asp?rs=&t=88794204&ID=23535352253) | [ABC](https://www.english-corpora.org/corona/x3.asp?node=&p=2&w10=coronavirus&w11=vaccination&r=) |  |  |  | , File) # JERUSALEM -- Israel's prime minister says the country's **coronavirus** **vaccination** " green pass " system will be suspended as new daily cases of COVID-19 continue |
| [187](https://www.english-corpora.org/corona/x4.asp?rs=&t=53639434&ID=23420560225) | [22-02-10 CA](https://www.english-corpora.org/corona/x4.asp?rs=&t=53639434&ID=23420560225) | [vice.com](https://www.english-corpora.org/corona/x3.asp?node=&p=2&w10=coronavirus&w11=vaccination&r=) |  |  |  | complaint at London's Hammersmith police station in December claiming that the government's **coronavirus** **vaccination** programme was criminal in nature. # Police issued the crime reference number (CRN |
| [188](https://www.english-corpora.org/corona/x4.asp?rs=&t=53641319&ID=23431764564) | [22-02-11 CA](https://www.english-corpora.org/corona/x4.asp?rs=&t=53641319&ID=23431764564) | [ca.news.yahoo.com](https://www.english-corpora.org/corona/x3.asp?node=&p=2&w10=coronavirus&w11=vaccination&r=) |  |  |  | Denmark said Friday that they were considering " winding down " the country's **coronavirus** **vaccination** program in the spring and see no reason now to administer a booster dose to |
| [189](https://www.english-corpora.org/corona/x4.asp?rs=&t=53656556&ID=23501036601) | [22-02-15 CA](https://www.english-corpora.org/corona/x4.asp?rs=&t=53656556&ID=23501036601) | [jamaica-gleaner.com](https://www.english-corpora.org/corona/x3.asp?node=&p=2&w10=coronavirus&w11=vaccination&r=) |  |  |  | at Kingston Wharves recently. # Approximately 1,200 people have been inoculated across four **coronavirus** **vaccination** blitzes held in Newport West. # The fourth session, held by the Ministry |
| [190](https://www.english-corpora.org/corona/x4.asp?rs=&t=43933030&ID=23318879522) | [22-02-03 GB](https://www.english-corpora.org/corona/x4.asp?rs=&t=43933030&ID=23318879522) | [countypress.co.uk](https://www.english-corpora.org/corona/x3.asp?node=&p=2&w10=coronavirus&w11=vaccination&r=) |  |  |  | has dropped by more than 17,000 since the Government launched its consultation on mandatory **coronavirus** **vaccination**, figures suggest. # There were 406,365 domiciliary care staff in registered settings reported |
| [191](https://www.english-corpora.org/corona/x4.asp?rs=&t=74001702&ID=23304211232) | [22-02-02 IN](https://www.english-corpora.org/corona/x4.asp?rs=&t=74001702&ID=23304211232) | [ibtimes.co.in](https://www.english-corpora.org/corona/x3.asp?node=&p=2&w10=coronavirus&w11=vaccination&r=) |  |  |  | also accepted that Snehal's death was the result of side effects associated with **coronavirus** **vaccination**. # " She had bleeding, clot formation with low platelets which are all |
| [192](https://www.english-corpora.org/corona/x4.asp?rs=&t=74015579&ID=23356920445) | [22-02-05 IN](https://www.english-corpora.org/corona/x4.asp?rs=&t=74015579&ID=23356920445) | [scroll.in](https://www.english-corpora.org/corona/x3.asp?node=&p=2&w10=coronavirus&w11=vaccination&r=) |  |  |  | The petition had sought the removal of Prime Minister Narendra Modi's photograph from **coronavirus** **vaccination** certificates. # The division bench said that the photograph was an attempt by the |
| [193](https://www.english-corpora.org/corona/x4.asp?rs=&t=33735823&ID=23374043382) | [22-02-07 PK](https://www.english-corpora.org/corona/x4.asp?rs=&t=33735823&ID=23374043382) | [geo.tv](https://www.english-corpora.org/corona/x3.asp?node=&p=2&w10=coronavirus&w11=vaccination&r=) |  |  |  | in the total number of recuperations reported so far. Pakistan registers new **coronavirus** **vaccination** record # Meanwhile, Pakistan registered a new coronavirus vaccination record as more people follow |
| [194](https://www.english-corpora.org/corona/x4.asp?rs=&t=33735823&ID=23374043392) | [22-02-07 PK](https://www.english-corpora.org/corona/x4.asp?rs=&t=33735823&ID=23374043392) | [geo.tv](https://www.english-corpora.org/corona/x3.asp?node=&p=2&w10=coronavirus&w11=vaccination&r=) |  |  |  | Pakistan registers new coronavirus vaccination record # Meanwhile, Pakistan registered a new **coronavirus** **vaccination** record as more people follow the advice of Pakistan's Special Assistant to the Prime |
| [195](https://www.english-corpora.org/corona/x4.asp?rs=&t=53644539&ID=23437936740) | [22-02-11 PK](https://www.english-corpora.org/corona/x4.asp?rs=&t=53644539&ID=23437936740) | [dailytimes.com.pk](https://www.english-corpora.org/corona/x3.asp?node=&p=2&w10=coronavirus&w11=vaccination&r=) |  |  |  | took the total recoveries count to 1,354,298. # Pakistan has registered a new **coronavirus** **vaccination** record as more people follow the advice of Pakistan's Special Assistant to the Prime |
| [196](https://www.english-corpora.org/corona/x4.asp?rs=&t=43999281&ID=23524929157) | [22-02-16 SG](https://www.english-corpora.org/corona/x4.asp?rs=&t=43999281&ID=23524929157) | [sg.news.yahoo.com](https://www.english-corpora.org/corona/x3.asp?node=&p=2&w10=coronavirus&w11=vaccination&r=) |  |  |  | just yet: MOH top official # A man looks at signs outside a **coronavirus** **vaccination** centre set up at a community centre in Singapore. (AFP via Getty Images |
| [197](https://www.english-corpora.org/corona/x4.asp?rs=&t=63864658&ID=23408097924) | [22-02-09 ZA](https://www.english-corpora.org/corona/x4.asp?rs=&t=63864658&ID=23408097924) | [ewn.co.za](https://www.english-corpora.org/corona/x3.asp?node=&p=2&w10=coronavirus&w11=vaccination&r=) |  |  |  | which includes that the vaccine is supplied and administered in accordance with the national **coronavirus** **vaccination** programme. # It's administered as two doses by intramuscular injection at an interval |
| [198](https://www.english-corpora.org/corona/x4.asp?rs=&t=63868587&ID=23444210084) | [22-02-11 ZA](https://www.english-corpora.org/corona/x4.asp?rs=&t=63868587&ID=23444210084) | [ewn.co.za](https://www.english-corpora.org/corona/x3.asp?node=&p=2&w10=coronavirus&w11=vaccination&r=) |  |  |  | which includes that the vaccine is supplied and administered in accordance with the national **coronavirus** **vaccination** programme. It's administered as two doses by intramuscular injection at an interval of |
| [199](https://www.english-corpora.org/corona/x4.asp?rs=&t=63847487&ID=23370415526) | [22-02-06 KE](https://www.english-corpora.org/corona/x4.asp?rs=&t=63847487&ID=23370415526) | [the-star.co.ke](https://www.english-corpora.org/corona/x3.asp?node=&p=2&w10=coronavirus&w11=vaccination&r=) |  |  |  | to reach the unvaccinated. # On Wednesday, Karoki said all its 29 **Coronavirus** **vaccination** centers will also be offering the doses. # Speaking while launching the initiative at |
| [200](https://www.english-corpora.org/corona/x4.asp?rs=&t=63843127&ID=23339308728) | [22-02-04 JM](https://www.english-corpora.org/corona/x4.asp?rs=&t=63843127&ID=23339308728) | [jamaicaobserver.com](https://www.english-corpora.org/corona/x3.asp?node=&p=2&w10=coronavirus&w11=vaccination&r=) |  |  |  | the country's cultural calendar in March this year, but cautioned that the **coronavirus** **vaccination** rate would determine the size of the festival. # -- Canadian Prime Minister Prime |

| PAGE:   [**<<**](https://www.english-corpora.org/corona/x3.asp?node=&p=1&w10=coronavirus&w11=vaccination&r=) [**<**](https://www.english-corpora.org/corona/x3.asp?node=&p=1&w10=coronavirus&w11=vaccination&r=)   2 / 42  [**>**](https://www.english-corpora.org/corona/x3.asp?node=&p=3&w10=coronavirus&w11=vaccination&r=) [**>>**](https://www.english-corpora.org/corona/x3.asp?node=&p=42&w10=coronavirus&w11=vaccination&r=) | | |
| --- | --- | --- |
| FIND SAMPLE:  [100](https://www.english-corpora.org/corona/x3.asp?node=&sample=100&w10=coronavirus&w11=vaccination&r=)  [200](https://www.english-corpora.org/corona/x3.asp?node=&sample=200&w10=coronavirus&w11=vaccination&r=)  [500](https://www.english-corpora.org/corona/x3.asp?node=&sample=500&w10=coronavirus&w11=vaccination&r=)  [1000](https://www.english-corpora.org/corona/x3.asp?node=&sample=1000&w10=coronavirus&w11=vaccination&r=) PAGE:   [**<<**](https://www.english-corpora.org/corona/x3.asp?node=&p=1&w10=coronavirus&w11=vaccination&r=) [**<**](https://www.english-corpora.org/corona/x3.asp?node=&p=2&w10=coronavirus&w11=vaccination&r=)   3 / 42  [**>**](https://www.english-corpora.org/corona/x3.asp?node=&p=4&w10=coronavirus&w11=vaccination&r=) [**>>**](https://www.english-corpora.org/corona/x3.asp?node=&p=42&w10=coronavirus&w11=vaccination&r=) |  |

Начало формы

| CLICK FOR MORE CONTEXT | [**HELP**](javascript:newFeatures()) | [SAVE](javascript:chooser('s'))    [TRANSLATE](javascript:chooser('t'))    [ANALYZE](javascript:chooser('p')) |
| --- | --- | --- |

| [201](https://www.english-corpora.org/corona/x4.asp?rs=&t=33599107&ID=22910320073) | [22-01-08 US](https://www.english-corpora.org/corona/x4.asp?rs=&t=33599107&ID=22910320073) | [aljazeera.com](https://www.english-corpora.org/corona/x3.asp?node=&p=3&w10=coronavirus&w11=vaccination&r=) |  |  |  | due to problems with the medical exemption from the country's immigration requirement for **coronavirus** **vaccination** that he presented. # The drama has caused tensions between Serbia and Australia and |
| --- | --- | --- | --- | --- | --- | --- |
| [202](https://www.english-corpora.org/corona/x4.asp?rs=&t=33604930&ID=22932429524) | [22-01-10 US](https://www.english-corpora.org/corona/x4.asp?rs=&t=33604930&ID=22932429524) | [chron.com](https://www.english-corpora.org/corona/x3.asp?node=&p=3&w10=coronavirus&w11=vaccination&r=) |  |  |  | and customers at restaurants, gyms and many other indoor locations show proof of **coronavirus** **vaccination** to curb a rise in COVID-19 cases. # The new digital vaccine cards use |
| [203](https://www.english-corpora.org/corona/x4.asp?rs=&t=33606631&ID=22932747030) | [22-01-10 US](https://www.english-corpora.org/corona/x4.asp?rs=&t=33606631&ID=22932747030) | [pbs.org](https://www.english-corpora.org/corona/x3.asp?node=&p=3&w10=coronavirus&w11=vaccination&r=) |  |  |  | to stay in Australia to contest the Australian Open after his exemption from strict **coronavirus** **vaccination** rules was questioned, but the drama might not be finished, with the government |
| [204](https://www.english-corpora.org/corona/x4.asp?rs=&t=33609071&ID=22942734722) | [22-01-10 US](https://www.english-corpora.org/corona/x4.asp?rs=&t=33609071&ID=22942734722) | [pressherald.com](https://www.english-corpora.org/corona/x3.asp?node=&p=3&w10=coronavirus&w11=vaccination&r=) |  |  |  | to require proof of vaccination # Quebec will require people to show proof of **coronavirus** **vaccination** when entering government-run stores selling cannabis or alcohol, the region's health minister, |
| [205](https://www.english-corpora.org/corona/x4.asp?rs=&t=33613406&ID=22951697360) | [22-01-11 US](https://www.english-corpora.org/corona/x4.asp?rs=&t=33613406&ID=22951697360) | [medicalxpress.com](https://www.english-corpora.org/corona/x3.asp?node=&p=3&w10=coronavirus&w11=vaccination&r=) |  |  |  | than 400,000 vulnerable Israelis -- mostly people over 60 -- have gotten a fourth **coronavirus** **vaccination** and hospitals are prepared for an influx of severe illness. Bennett argued that these |
| [207](https://www.english-corpora.org/corona/x4.asp?rs=&t=33617326&ID=22970059883) | [22-01-12 US](https://www.english-corpora.org/corona/x4.asp?rs=&t=33617326&ID=22970059883) | [yahoo.com](https://www.english-corpora.org/corona/x3.asp?node=&p=3&w10=coronavirus&w11=vaccination&r=)[(1)](https://www.english-corpora.org/corona/duplicates1.asp?xx=206&n=1) |  |  |  | opponents said he should resign. * Denmark is to offer a fourth **coronavirus** **vaccination** to vulnerable citizens and will ease restrictions at the end of the week, while |
| [208](https://www.english-corpora.org/corona/x4.asp?rs=&t=33621435&ID=22990227619) | [22-01-13 US](https://www.english-corpora.org/corona/x4.asp?rs=&t=33621435&ID=22990227619) | [dailynews.com](https://www.english-corpora.org/corona/x3.asp?node=&p=3&w10=coronavirus&w11=vaccination&r=) |  |  |  | fired or face termination despite filing for exemptions to the district's mandatory employee **coronavirus** **vaccination** mandate. # The plaintiffs in the Los Angeles Superior Court lawsuit brought Monday are |
| [209](https://www.english-corpora.org/corona/x4.asp?rs=&t=33628298&ID=23023828583) | [22-01-15 US](https://www.english-corpora.org/corona/x4.asp?rs=&t=33628298&ID=23023828583) | [cleveland.com](https://www.english-corpora.org/corona/x3.asp?node=&p=3&w10=coronavirus&w11=vaccination&r=) |  |  |  | on Thursday decided to put a hold on the implementation of a new federal **coronavirus** **vaccination** and testing mandate for large employers while lower courts weigh the policy's legality, |
| [210](https://www.english-corpora.org/corona/x4.asp?rs=&t=33635326&ID=23041947702) | [22-01-17 US](https://www.english-corpora.org/corona/x4.asp?rs=&t=33635326&ID=23041947702) | [news.yahoo.com](https://www.english-corpora.org/corona/x3.asp?node=&p=3&w10=coronavirus&w11=vaccination&r=) |  |  |  | from Australia on Sunday. # It ended a sensational legal battle over his **coronavirus** **vaccination** status and dashed his dream for the moment of an unprecedented 21st Grand Slam singles |
| [211](https://www.english-corpora.org/corona/x4.asp?rs=&t=33641390&ID=23082957180) | [22-01-19 US](https://www.english-corpora.org/corona/x4.asp?rs=&t=33641390&ID=23082957180) | [nytimes.com](https://www.english-corpora.org/corona/x3.asp?node=&p=3&w10=coronavirus&w11=vaccination&r=) |  |  |  | placed on administrative leave after sending an email to his employees noting their lackluster **coronavirus** **vaccination** rates and urging them to get the shots. # The official -- Dr. Raul |
| [213](https://www.english-corpora.org/corona/x4.asp?rs=&t=53510745&ID=22914873283) | [22-01-08 US](https://www.english-corpora.org/corona/x4.asp?rs=&t=53510745&ID=22914873283) | [ibtimes.com](https://www.english-corpora.org/corona/x3.asp?node=&p=3&w10=coronavirus&w11=vaccination&r=)[(1)](https://www.english-corpora.org/corona/duplicates1.asp?xx=212&n=1) |  |  |  | over 71 percent of the population double jabbed, Germany has a lower **coronavirus** **vaccination** rate than France, Italy or Spain. # Almost 42 percent of Germans have |
| [214](https://www.english-corpora.org/corona/x4.asp?rs=&t=53519814&ID=22953354394) | [22-01-11 US](https://www.english-corpora.org/corona/x4.asp?rs=&t=53519814&ID=22953354394) | [beaumontenterprise.com](https://www.english-corpora.org/corona/x3.asp?node=&p=3&w10=coronavirus&w11=vaccination&r=) |  |  |  | law offers citizens new power to sue if they believe companies slighted them with **coronavirus** **vaccination** requirements. # McNally and Sexton have been cool to the idea of changing the |
| [215](https://www.english-corpora.org/corona/x4.asp?rs=&t=53526149&ID=22990995175) | [22-01-13 US](https://www.english-corpora.org/corona/x4.asp?rs=&t=53526149&ID=22990995175) | [dailyprogress.com](https://www.english-corpora.org/corona/x3.asp?node=&p=3&w10=coronavirus&w11=vaccination&r=) |  |  |  | was spending Thursday holed up in Downing Street. A planned visit to a **coronavirus** **vaccination** center was called off after a family member tested positive for the coronavirus, the |
| [216](https://www.english-corpora.org/corona/x4.asp?rs=&t=63739163&ID=22940511704) | [22-01-10 US](https://www.english-corpora.org/corona/x4.asp?rs=&t=63739163&ID=22940511704) | [reuters.com](https://www.english-corpora.org/corona/x3.asp?node=&p=3&w10=coronavirus&w11=vaccination&r=) |  |  |  | cancelled because of problems with the medical exemption from Australia's immigration requirement for **coronavirus** **vaccination** that he presented. # He was released on Monday, after a court quashed |
| [217](https://www.english-corpora.org/corona/x4.asp?rs=&t=73892543&ID=22925815303) | [22-01-09 US](https://www.english-corpora.org/corona/x4.asp?rs=&t=73892543&ID=22925815303) | [theweek.com](https://www.english-corpora.org/corona/x3.asp?node=&p=3&w10=coronavirus&w11=vaccination&r=) |  |  |  | # The Supreme Court is scheduled to review two challenges to President Biden's **coronavirus** **vaccination** requirements for private employers and health-care facilities on Friday. Biden has said the rules |
| [219](https://www.english-corpora.org/corona/x4.asp?rs=&t=73904192&ID=22964172179) | [22-01-11 US](https://www.english-corpora.org/corona/x4.asp?rs=&t=73904192&ID=22964172179) | [theweek.com](https://www.english-corpora.org/corona/x3.asp?node=&p=3&w10=coronavirus&w11=vaccination&r=)[(1)](https://www.english-corpora.org/corona/duplicates1.asp?xx=218&n=1) |  |  |  | said Djokovic failed to comply with rules regarding his exemption from Australia's **coronavirus** **vaccination** mandate. The judge's decision potentially cleared Djokovic to play in next week's |
| [220](https://www.english-corpora.org/corona/x4.asp?rs=&t=73911322&ID=22989677457) | [22-01-13 US](https://www.english-corpora.org/corona/x4.asp?rs=&t=73911322&ID=22989677457) | [sports.yahoo.com](https://www.english-corpora.org/corona/x3.asp?node=&p=3&w10=coronavirus&w11=vaccination&r=) |  |  |  | These are bureaucrats... # COPENHAGEN (Reuters) -Denmark will offer a fourth **coronavirus** **vaccination** to the most vulnerable citizens as it faces record infections from the Omicron variant, |
| [221](https://www.english-corpora.org/corona/x4.asp?rs=&t=73935493&ID=23069955538) | [22-01-18 US](https://www.english-corpora.org/corona/x4.asp?rs=&t=73935493&ID=23069955538) | [washingtontimes.com](https://www.english-corpora.org/corona/x3.asp?node=&p=3&w10=coronavirus&w11=vaccination&r=) |  |  |  | # Under the new mandate, people are required to present proof of their **coronavirus** **vaccination** status to enter public venues. Businesses also must display signs telling customers that they |
| [222](https://www.english-corpora.org/corona/x4.asp?rs=&t=73940748&ID=23082713044) | [22-01-19 US](https://www.english-corpora.org/corona/x4.asp?rs=&t=73940748&ID=23082713044) | [adn.com](https://www.english-corpora.org/corona/x3.asp?node=&p=3&w10=coronavirus&w11=vaccination&r=) |  |  |  | to be the latest attempt to shame and boycott a company over its mandatory **coronavirus** **vaccination** policy for employees. The company has also faced protests from employees opposed to the |
| [223](https://www.english-corpora.org/corona/x4.asp?rs=&t=88601662&ID=22948398072) | [22-01-10 US](https://www.english-corpora.org/corona/x4.asp?rs=&t=88601662&ID=22948398072) | [New York Times](https://www.english-corpora.org/corona/x3.asp?node=&p=3&w10=coronavirus&w11=vaccination&r=) |  |  |  | had been detained after the Australian government canceled his visa over questions about his **coronavirus** **vaccination** exemption. But he isn't necessarily guaranteed to appear at the Australian Open next |
| [224](https://www.english-corpora.org/corona/x4.asp?rs=&t=88602212&ID=22946929749) | [22-01-10 US](https://www.english-corpora.org/corona/x4.asp?rs=&t=88602212&ID=22946929749) | [Voice of America](https://www.english-corpora.org/corona/x3.asp?node=&p=3&w10=coronavirus&w11=vaccination&r=) |  |  |  | # FILE - Israelis receive a Pfizer-BioNTech COVID-19 vaccine from medical professionals at a **coronavirus** **vaccination** center set up on a shopping mall parking lot in Givataim, Israel, Feb. |
| [225](https://www.english-corpora.org/corona/x4.asp?rs=&t=88607156&ID=22967372860) | [22-01-11 US](https://www.english-corpora.org/corona/x4.asp?rs=&t=88607156&ID=22967372860) | [Washington Post](https://www.english-corpora.org/corona/x3.asp?node=&p=3&w10=coronavirus&w11=vaccination&r=) |  |  |  | all-time among men's tennis players. But Djokovic has yet to receive a **coronavirus** **vaccination**, explaining over the years that he is against forced vaccination and hopes to always |
| [226](https://www.english-corpora.org/corona/x4.asp?rs=&t=88609071&ID=22968119024) | [22-01-11 US](https://www.english-corpora.org/corona/x4.asp?rs=&t=88609071&ID=22968119024) | [U.S. Chamber of Commerce](https://www.english-corpora.org/corona/x3.asp?node=&p=3&w10=coronavirus&w11=vaccination&r=) |  |  |  | # First, compared to other occupations, construction craft workers report much lower **coronavirus** **vaccination** rates and higher " vaccine hesitancy " rates. That makes them more susceptible to |
| [227](https://www.english-corpora.org/corona/x4.asp?rs=&t=88611203&ID=22981743141) | [22-01-12 US](https://www.english-corpora.org/corona/x4.asp?rs=&t=88611203&ID=22981743141) | [The Guardian on MSN.com](https://www.english-corpora.org/corona/x3.asp?node=&p=3&w10=coronavirus&w11=vaccination&r=) |  |  |  | three months of the Covid-19 pandemic. # Denmark is to offer a fourth **coronavirus** **vaccination** to vulnerable citizens as it faces record infection numbers from the Omicron variant, the |
| [229](https://www.english-corpora.org/corona/x4.asp?rs=&t=88614477&ID=22986616495) | [22-01-12 US](https://www.english-corpora.org/corona/x4.asp?rs=&t=88614477&ID=22986616495) | [San Francisco Chronicle](https://www.english-corpora.org/corona/x3.asp?node=&p=3&w10=coronavirus&w11=vaccination&r=)[(1)](https://www.english-corpora.org/corona/duplicates1.asp?xx=228&n=1) |  |  |  | , introduced the bill after protests last year briefly shut down a mass **coronavirus** **vaccination** site at Dodger Stadium in Los Angeles, and said it was needed to protect |
| [230](https://www.english-corpora.org/corona/x4.asp?rs=&t=88615394&ID=23000044895) | [22-01-13 US](https://www.english-corpora.org/corona/x4.asp?rs=&t=88615394&ID=23000044895) | [ABC](https://www.english-corpora.org/corona/x3.asp?node=&p=3&w10=coronavirus&w11=vaccination&r=) |  |  |  | percentage died. # ABCNews.com # COPENHAGEN, Denmark -- Denmark will offer fourth **coronavirus** **vaccination** shots to risk groups and vulnerable citizens as the pandemic situation in the country has |
| [231](https://www.english-corpora.org/corona/x4.asp?rs=&t=88616308&ID=22997372001) | [22-01-13 US](https://www.english-corpora.org/corona/x4.asp?rs=&t=88616308&ID=22997372001) | [Patch](https://www.english-corpora.org/corona/x3.asp?node=&p=3&w10=coronavirus&w11=vaccination&r=) |  |  |  | on the map below and click on a pin to see that community's **coronavirus** **vaccination** rates. You can also view the town-by-town coronavirus vaccination data in the spreadsheet we |
| [232](https://www.english-corpora.org/corona/x4.asp?rs=&t=88616308&ID=22997372011) | [22-01-13 US](https://www.english-corpora.org/corona/x4.asp?rs=&t=88616308&ID=22997372011) | [Patch](https://www.english-corpora.org/corona/x3.asp?node=&p=3&w10=coronavirus&w11=vaccination&r=) |  |  |  | see that community's coronavirus vaccination rates. You can also view the town-by-town **coronavirus** **vaccination** data in the spreadsheet we used to create this map. # Colors reflect the |
| [233](https://www.english-corpora.org/corona/x4.asp?rs=&t=88616730&ID=22996080887) | [22-01-13 US](https://www.english-corpora.org/corona/x4.asp?rs=&t=88616730&ID=22996080887) | [Patch](https://www.english-corpora.org/corona/x3.asp?node=&p=3&w10=coronavirus&w11=vaccination&r=) |  |  |  | an insurance card, if available. Insurance is not required to receive a **coronavirus** **vaccination** or booster shot. Find out what's happening in Salem with free, |
| [234](https://www.english-corpora.org/corona/x4.asp?rs=&t=88617445&ID=22998539490) | [22-01-13 US](https://www.english-corpora.org/corona/x4.asp?rs=&t=88617445&ID=22998539490) | [Reuters](https://www.english-corpora.org/corona/x3.asp?node=&p=3&w10=coronavirus&w11=vaccination&r=) |  |  |  | access to Reuters.com # On Wednesday, Denmark said it would offer a fourth **coronavirus** **vaccination** to the most vulnerable citizens. # The European Union's drug regulator has expressed |
| [235](https://www.english-corpora.org/corona/x4.asp?rs=&t=88623436&ID=23013837791) | [22-01-14 US](https://www.english-corpora.org/corona/x4.asp?rs=&t=88623436&ID=23013837791) | [The Guardian on MSN.com](https://www.english-corpora.org/corona/x3.asp?node=&p=3&w10=coronavirus&w11=vaccination&r=) |  |  |  | administration had seen major successes on a variety of other fronts, including boosting **coronavirus** **vaccination** rates and helping schools reopen safely. # " So the message from the president |
| [236](https://www.english-corpora.org/corona/x4.asp?rs=&t=88635139&ID=23039531155) | [22-01-16 US](https://www.english-corpora.org/corona/x4.asp?rs=&t=88635139&ID=23039531155) | [New York Times](https://www.english-corpora.org/corona/x3.asp?node=&p=3&w10=coronavirus&w11=vaccination&r=) |  |  |  | on the day that Mr. Djokovic left Australia after a legal dispute surrounding his **coronavirus** **vaccination** status. # " They think that they humiliated Djokovic with this 10-day harassment, |
| [237](https://www.english-corpora.org/corona/x4.asp?rs=&t=88635139&ID=23039532154) | [22-01-16 US](https://www.english-corpora.org/corona/x4.asp?rs=&t=88635139&ID=23039532154) | [New York Times](https://www.english-corpora.org/corona/x3.asp?node=&p=3&w10=coronavirus&w11=vaccination&r=) |  |  |  | the truth. In particular, he took issue with the way Serbia's **coronavirus** **vaccination** rates were presented in court. # " They say that less than 50 percent |
| [238](https://www.english-corpora.org/corona/x4.asp?rs=&t=88636492&ID=23040133374) | [22-01-16 US](https://www.english-corpora.org/corona/x4.asp?rs=&t=88636492&ID=23040133374) | [New York Times](https://www.english-corpora.org/corona/x3.asp?node=&p=3&w10=coronavirus&w11=vaccination&r=) |  |  |  | not telling the truth. In particular, he took issue with the way **coronavirus** **vaccination** rates in Serbia were presented in court. # " They say that less than |
| [239](https://www.english-corpora.org/corona/x4.asp?rs=&t=88640213&ID=23054454877) | [22-01-17 US](https://www.english-corpora.org/corona/x4.asp?rs=&t=88640213&ID=23054454877) | [Hindustan Times](https://www.english-corpora.org/corona/x3.asp?node=&p=3&w10=coronavirus&w11=vaccination&r=) |  |  |  | for vaccination # The next stage of the **coronavirus** **vaccination** process begins with teens getting the jab as Omicron hastens the need to expand coverage |
| [240](https://www.english-corpora.org/corona/x4.asp?rs=&t=88640213&ID=23054454958) | [22-01-17 US](https://www.english-corpora.org/corona/x4.asp?rs=&t=88640213&ID=23054454958) | [Hindustan Times](https://www.english-corpora.org/corona/x3.asp?node=&p=3&w10=coronavirus&w11=vaccination&r=) |  |  |  | Via # Copy Link # ByHT Editorial # A new phase of India's **coronavirus** **vaccination** began on Monday, with children in the 15 to 18 age group getting their |
| [241](https://www.english-corpora.org/corona/x4.asp?rs=&t=88642224&ID=23054849980) | [22-01-17 US](https://www.english-corpora.org/corona/x4.asp?rs=&t=88642224&ID=23054849980) | [The Washington Post on MSN.com](https://www.english-corpora.org/corona/x3.asp?node=&p=3&w10=coronavirus&w11=vaccination&r=) |  |  |  | ? Why did he refuse to follow the Australian government edict that visitors receive **coronavirus** **vaccination** and instead seek an iffy " medical exemption, " only to be caught violating |
| [242](https://www.english-corpora.org/corona/x4.asp?rs=&t=88650600&ID=23086659122) | [22-01-19 US](https://www.english-corpora.org/corona/x4.asp?rs=&t=88650600&ID=23086659122) | [YAHOO!News](https://www.english-corpora.org/corona/x3.asp?node=&p=3&w10=coronavirus&w11=vaccination&r=) |  |  |  | The White House is considering requiring migrants aged 5 and older to receive a **coronavirus** **vaccination** as a condition for crossing the U.S.-Mexico border to await court hearings, Axios has |
| [243](https://www.english-corpora.org/corona/x4.asp?rs=&t=88650634&ID=23089173043) | [22-01-19 US](https://www.english-corpora.org/corona/x4.asp?rs=&t=88650634&ID=23089173043) | [The Guardian on MSN.com](https://www.english-corpora.org/corona/x3.asp?node=&p=3&w10=coronavirus&w11=vaccination&r=) |  |  |  | " the president said. # Biden touted his administration's success in boosting **coronavirus** **vaccination** rates and lowering the US unemployment rate, despite widespread criticism of Democrats' failure |
| [244](https://www.english-corpora.org/corona/x4.asp?rs=&t=88650634&ID=23089175485) | [22-01-19 US](https://www.english-corpora.org/corona/x4.asp?rs=&t=88650634&ID=23089175485) | [The Guardian on MSN.com](https://www.english-corpora.org/corona/x3.asp?node=&p=3&w10=coronavirus&w11=vaccination&r=) |  |  |  | instead stressed the accomplishments of Biden's first year in office, including boosting **coronavirus** **vaccination** rates and getting the bipartisan infrastructure bill signed into law. # This progress on |
| [245](https://www.english-corpora.org/corona/x4.asp?rs=&t=88652542&ID=23088920957) | [22-01-19 US](https://www.english-corpora.org/corona/x4.asp?rs=&t=88652542&ID=23088920957) | [CNN](https://www.english-corpora.org/corona/x3.asp?node=&p=3&w10=coronavirus&w11=vaccination&r=) |  |  |  | following an outcry over his controversial " medical exemption " from the country's **coronavirus** **vaccination** rules. # Djokovic, the men's tennis world no.1, hasn't publicly |
| [246](https://www.english-corpora.org/corona/x4.asp?rs=&t=53531420&ID=23017636037) | [22-01-15 CA](https://www.english-corpora.org/corona/x4.asp?rs=&t=53531420&ID=23017636037) | [ca.sports.yahoo.com](https://www.english-corpora.org/corona/x3.asp?node=&p=3&w10=coronavirus&w11=vaccination&r=) |  |  |  | The nine-time Australian Open champion had received an exemption through Tennis Australia from strict **coronavirus** **vaccination** rules for arrivals into the country by virtue of having tested positive last month. |
| [248](https://www.english-corpora.org/corona/x4.asp?rs=&t=53538686&ID=23051616246) | [22-01-17 CA](https://www.english-corpora.org/corona/x4.asp?rs=&t=53538686&ID=23051616246) | [ca.news.yahoo.com](https://www.english-corpora.org/corona/x3.asp?node=&p=3&w10=coronavirus&w11=vaccination&r=)[(1)](https://www.english-corpora.org/corona/duplicates1.asp?xx=247&n=1) |  |  |  | a.m. *12 min read # A railway staff checks a passenger's Covid-19 **coronavirus** **vaccination** certificate before boarding a train at a railway station in Chennai on January 10, |
| [249](https://www.english-corpora.org/corona/x4.asp?rs=&t=33619090&ID=22978191547) | [22-01-12 GB](https://www.english-corpora.org/corona/x4.asp?rs=&t=33619090&ID=22978191547) | [eurosport.co.uk](https://www.english-corpora.org/corona/x3.asp?node=&p=3&w10=coronavirus&w11=vaccination&r=) |  |  |  | held for four days after it was decided that his medical exemption from the **coronavirus** **vaccination** to enter the country was not sufficient. He was released on Monday after a |
| [250](https://www.english-corpora.org/corona/x4.asp?rs=&t=43823372&ID=22942869465) | [22-01-10 GB](https://www.english-corpora.org/corona/x4.asp?rs=&t=43823372&ID=22942869465) | [standard.co.uk](https://www.english-corpora.org/corona/x3.asp?node=&p=3&w10=coronavirus&w11=vaccination&r=) |  |  |  | Johnson in Uxbridge, west London, after a visit to a Boots Pharmacy **coronavirus** **vaccination** clinic # / PA Wire # PCR tests scrapped for asymptomatic cases # From Tuesday |
| [251](https://www.english-corpora.org/corona/x4.asp?rs=&t=43823661&ID=22938150699) | [22-01-10 GB](https://www.english-corpora.org/corona/x4.asp?rs=&t=43823661&ID=22938150699) | [politico.eu](https://www.english-corpora.org/corona/x3.asp?node=&p=3&w10=coronavirus&w11=vaccination&r=) |  |  |  | annul his visa because of concerns over the validity of his medical exemption from **coronavirus** **vaccination**. # Speaking to Serbian media on Saturday, Brnabic said her government had been |
| [252](https://www.english-corpora.org/corona/x4.asp?rs=&t=43824425&ID=22933456502) | [22-01-10 GB](https://www.english-corpora.org/corona/x4.asp?rs=&t=43824425&ID=22933456502) | [shropshirestar.com](https://www.english-corpora.org/corona/x3.asp?node=&p=3&w10=coronavirus&w11=vaccination&r=) |  |  |  | Johnson in Uxbridge, west London, after a visit to a Boots Pharmacy **coronavirus** **vaccination** clinic (Dominic Lipinski/PA) # The Prime Minister is under pressure from Tory MPs |
| [253](https://www.english-corpora.org/corona/x4.asp?rs=&t=43828762&ID=22956426105) | [22-01-11 GB](https://www.english-corpora.org/corona/x4.asp?rs=&t=43828762&ID=22956426105) | [standard.co.uk](https://www.english-corpora.org/corona/x3.asp?node=&p=3&w10=coronavirus&w11=vaccination&r=) |  |  |  | as ministers made a fresh push for people to come forward for their third **coronavirus** **vaccination** dose. # Speaking to broadcasters during a visit to Northamptonshire, the Prime Minister |
| [254](https://www.english-corpora.org/corona/x4.asp?rs=&t=43829508&ID=22952086801) | [22-01-11 GB](https://www.english-corpora.org/corona/x4.asp?rs=&t=43829508&ID=22952086801) | [dailyrecord.co.uk](https://www.english-corpora.org/corona/x3.asp?node=&p=3&w10=coronavirus&w11=vaccination&r=) |  |  |  | The latest local vaccination data shows that 333,186 people have had at least one **coronavirus** **vaccination**, 308,854 have received at least two, while 241,739 have also received a third |
| [255](https://www.english-corpora.org/corona/x4.asp?rs=&t=43840019&ID=23002417626) | [22-01-14 GB](https://www.english-corpora.org/corona/x4.asp?rs=&t=43840019&ID=23002417626) | [thenational.scot](https://www.english-corpora.org/corona/x3.asp?node=&p=3&w10=coronavirus&w11=vaccination&r=) |  |  |  | The nine-time Australian Open champion had received an exemption through Tennis Australia from strict **coronavirus** **vaccination** rules for arrivals into the country by virtue of having tested positive last month. |
| [256](https://www.english-corpora.org/corona/x4.asp?rs=&t=43848952&ID=23033003838) | [22-01-16 GB](https://www.english-corpora.org/corona/x4.asp?rs=&t=43848952&ID=23033003838) | [politico.eu](https://www.english-corpora.org/corona/x3.asp?node=&p=3&w10=coronavirus&w11=vaccination&r=) |  |  |  | coronavirus **vaccination** mandatory from February, the government confirmed Sunday. # Presenting the final version of |
| [257](https://www.english-corpora.org/corona/x4.asp?rs=&t=43861215&ID=23076383147) | [22-01-19 GB](https://www.english-corpora.org/corona/x4.asp?rs=&t=43861215&ID=23076383147) | [theguardian.com](https://www.english-corpora.org/corona/x3.asp?node=&p=3&w10=coronavirus&w11=vaccination&r=) |  |  |  | instead stressed the accomplishments of Biden's first year in office, including boosting **coronavirus** **vaccination** rates and getting the bipartisan infrastructure bill signed into law. # Jen Psaki ( |
| [258](https://www.english-corpora.org/corona/x4.asp?rs=&t=73899934&ID=22959111643) | [22-01-11 AU](https://www.english-corpora.org/corona/x4.asp?rs=&t=73899934&ID=22959111643) | [thecourier.com.au](https://www.english-corpora.org/corona/x3.asp?node=&p=3&w10=coronavirus&w11=vaccination&r=) |  |  |  | . This is how you can continue to access our trusted content: **Coronavirus** **vaccination** in Ballarat: school clinics to help get kids jabs done # JABBED: Audrey |
| [259](https://www.english-corpora.org/corona/x4.asp?rs=&t=73904628&ID=22972741620) | [22-01-12 AU](https://www.english-corpora.org/corona/x4.asp?rs=&t=73904628&ID=22972741620) | [thewest.com.au](https://www.english-corpora.org/corona/x3.asp?node=&p=3&w10=coronavirus&w11=vaccination&r=) |  |  |  | opponents said he should resign. # * Denmark is to offer a fourth **coronavirus** **vaccination** to vulnerable citizens and will ease restrictions at the end of the week, while |
| [260](https://www.english-corpora.org/corona/x4.asp?rs=&t=73937370&ID=23085796075) | [22-01-19 AU](https://www.english-corpora.org/corona/x4.asp?rs=&t=73937370&ID=23085796075) | [thenewdaily.com.au](https://www.english-corpora.org/corona/x3.asp?node=&p=3&w10=coronavirus&w11=vaccination&r=) |  |  |  | Novak Djokovic walks with coach Goran Ivanisevic in Dubai after being deported over his **coronavirus** **vaccination** status. Photo: AFPTV/AFP/Getty ' Could have been prevented from the beginning' |
| [261](https://www.english-corpora.org/corona/x4.asp?rs=&t=53529666&ID=23006552924) | [22-01-14 IN](https://www.english-corpora.org/corona/x4.asp?rs=&t=53529666&ID=23006552924) | [newkerala.com](https://www.english-corpora.org/corona/x3.asp?node=&p=3&w10=coronavirus&w11=vaccination&r=) |  |  |  | people in Germany are still unvaccinated. # Lauterbach reiterated his support for compulsory **coronavirus** **vaccination** in Germany, which he described as " the safest and fastest way out of |
| [262](https://www.english-corpora.org/corona/x4.asp?rs=&t=63738167&ID=22945072026) | [22-01-10 IN](https://www.english-corpora.org/corona/x4.asp?rs=&t=63738167&ID=22945072026) | [rediff.com](https://www.english-corpora.org/corona/x3.asp?node=&p=3&w10=coronavirus&w11=vaccination&r=) |  |  |  | cancelled because of problems with the medical exemption from Australia's immigration requirement for **coronavirus** **vaccination** that he presented. # He was released on Monday, after a court quashed |
| [263](https://www.english-corpora.org/corona/x4.asp?rs=&t=73890067&ID=22927374789) | [22-01-09 IN](https://www.english-corpora.org/corona/x4.asp?rs=&t=73890067&ID=22927374789) | [thestatesman.com](https://www.english-corpora.org/corona/x3.asp?node=&p=3&w10=coronavirus&w11=vaccination&r=) |  |  |  | total of 151.58 crore vaccine doses so far under Nationwide Vaccination Drive. The **coronavirus** **vaccination** drive began on January 16, 2021. 1,51,57,60.645 people have been vaccinated in the country |
| [264](https://www.english-corpora.org/corona/x4.asp?rs=&t=88625061&ID=23012767754) | [22-01-14 IN](https://www.english-corpora.org/corona/x4.asp?rs=&t=88625061&ID=23012767754) | [The Financial Express](https://www.english-corpora.org/corona/x3.asp?node=&p=3&w10=coronavirus&w11=vaccination&r=) |  |  |  | that could require intensive care or leave long-lasting symptoms. (Reuters) # **Coronavirus** **Vaccination** for Children in India: Prime Minister Narendra Modi, in a surprise move, |
| [265](https://www.english-corpora.org/corona/x4.asp?rs=&t=53519845&ID=22957747225) | [22-01-11 PK](https://www.english-corpora.org/corona/x4.asp?rs=&t=53519845&ID=22957747225) | [dnd.com.pk](https://www.english-corpora.org/corona/x3.asp?node=&p=3&w10=coronavirus&w11=vaccination&r=) |  |  |  | ) on Monday to recognize the services of all workers who actively participated in **Coronavirus** **Vaccination** Campaign, Asad Umar once again advised people to follow Standard Operating Procedures (SOPs |
| [266](https://www.english-corpora.org/corona/x4.asp?rs=&t=43852682&ID=23051169380) | [22-01-17 MY](https://www.english-corpora.org/corona/x4.asp?rs=&t=43852682&ID=23051169380) | [malaysiakini.com](https://www.english-corpora.org/corona/x3.asp?node=&p=3&w10=coronavirus&w11=vaccination&r=) |  |  |  | the Covid-19 epidemic in war-torn countries, where access to primary health care and **coronavirus** **vaccination** is severely hampered. # Peter Maurer, president of the International Committee of the |
| [267](https://www.english-corpora.org/corona/x4.asp?rs=&t=43825956&ID=22938496425) | [22-01-10 SG](https://www.english-corpora.org/corona/x4.asp?rs=&t=43825956&ID=22938496425) | [straitstimes.com](https://www.english-corpora.org/corona/x3.asp?node=&p=3&w10=coronavirus&w11=vaccination&r=) |  |  |  | Grichka Bogdanoff died aged 72 over the new year, having earlier refused the **coronavirus** **vaccination** and fallen ill around December 15. # Hundreds of people attended the service in |
| [268](https://www.english-corpora.org/corona/x4.asp?rs=&t=73926789&ID=23053181015) | [22-01-17 PH](https://www.english-corpora.org/corona/x4.asp?rs=&t=73926789&ID=23053181015) | [ph.news.yahoo.com](https://www.english-corpora.org/corona/x3.asp?node=&p=3&w10=coronavirus&w11=vaccination&r=) |  |  |  | Djokovic landed in Dubai on Monday after his sensational deportation from Australia over his **coronavirus** **vaccination** status put his bid for a record 21st Grand Slam title on hold. # |
| [269](https://www.english-corpora.org/corona/x4.asp?rs=&t=73937894&ID=23085896500) | [22-01-19 PH](https://www.english-corpora.org/corona/x4.asp?rs=&t=73937894&ID=23085896500) | [pageone.ph](https://www.english-corpora.org/corona/x3.asp?node=&p=3&w10=coronavirus&w11=vaccination&r=) |  |  |  | the country's rice and food production. # People in countries with low **coronavirus** **vaccination** rates are at risk of severe illness and death, as Covid-19's Omicron variant |
| [274](https://www.english-corpora.org/corona/x4.asp?rs=&t=73939605&ID=23078948375) | [22-01-19 PH](https://www.english-corpora.org/corona/x4.asp?rs=&t=73939605&ID=23078948375) | [pna.gov.ph](https://www.english-corpora.org/corona/x3.asp?node=&p=3&w10=coronavirus&w11=vaccination&r=)[(4)](https://www.english-corpora.org/corona/duplicates1.asp?xx=273&n=4) |  |  |  | -- The Department of Education (DepEd) said Thursday its requirement of **coronavirus** **vaccination** for teaching and non-teaching personnel who will participate in limited face-to-face classes is to prevent |
| [275](https://www.english-corpora.org/corona/x4.asp?rs=&t=73939618&ID=23078955638) | [22-01-19 PH](https://www.english-corpora.org/corona/x4.asp?rs=&t=73939618&ID=23078955638) | [pageone.ph](https://www.english-corpora.org/corona/x3.asp?node=&p=3&w10=coronavirus&w11=vaccination&r=) |  |  |  | the country's rice and food production. # People in countries with low **coronavirus** **vaccination** rates are at risk of severe illness and death, as Covid-19's Omicron variant |
| [285](https://www.english-corpora.org/corona/x4.asp?rs=&t=73944147&ID=23101364187) | [22-01-20 PH](https://www.english-corpora.org/corona/x4.asp?rs=&t=73944147&ID=23101364187) | [pageone.ph](https://www.english-corpora.org/corona/x3.asp?node=&p=3&w10=coronavirus&w11=vaccination&r=)[(9)](https://www.english-corpora.org/corona/duplicates1.asp?xx=284&n=9) |  |  |  | the province. # The Department of Education said Thursday its requirement of **coronavirus** **vaccination** for teaching and non-teaching personnel who will participate in limited face-to-face classes is to prevent |
| [296](https://www.english-corpora.org/corona/x4.asp?rs=&t=63764879&ID=23052500329) | [22-01-17 ZA](https://www.english-corpora.org/corona/x4.asp?rs=&t=63764879&ID=23052500329) | [ewn.co.za](https://www.english-corpora.org/corona/x3.asp?node=&p=3&w10=coronavirus&w11=vaccination&r=)[(10)](https://www.english-corpora.org/corona/duplicates1.asp?xx=295&n=10) |  |  |  | landed in Dubai on Monday after his sensational deportation from Australia over his **coronavirus** **vaccination** status put his bid for a record 21st Grand Slam title on hold. # |
| [297](https://www.english-corpora.org/corona/x4.asp?rs=&t=53537905&ID=23031863060) | [22-01-16 NG](https://www.english-corpora.org/corona/x4.asp?rs=&t=53537905&ID=23031863060) | [sundiatapost.com](https://www.english-corpora.org/corona/x3.asp?node=&p=3&w10=coronavirus&w11=vaccination&r=) |  |  |  | Djokovic was deported from Australia Sunday having lost a high-stakes legal battle over his **coronavirus** **vaccination** status and with his dream of clinching a record 21st Grand Slam in tatters. |
| [298](https://www.english-corpora.org/corona/x4.asp?rs=&t=63759306&ID=23031990371) | [22-01-16 KE](https://www.english-corpora.org/corona/x4.asp?rs=&t=63759306&ID=23031990371) | [capitalfm.co.ke](https://www.english-corpora.org/corona/x3.asp?node=&p=3&w10=coronavirus&w11=vaccination&r=) |  |  |  | Djokovic landed in Dubai on Monday after his sensational deportation from Australia over his **coronavirus** **vaccination** status shattered his dream of scoring a record 21st Grand Slam title in Melbourne. |
| [299](https://www.english-corpora.org/corona/x4.asp?rs=&t=63778800&ID=23095669246) | [22-01-20 JM](https://www.english-corpora.org/corona/x4.asp?rs=&t=63778800&ID=23095669246) | [jamaicaobserver.com](https://www.english-corpora.org/corona/x3.asp?node=&p=3&w10=coronavirus&w11=vaccination&r=) |  |  |  | ? from? Australia Sunday having? lost a sensational legal battle over his **coronavirus** **vaccination** status and with his dream of clinching a record 21st Grand Slam in tatters. |
| [300](https://www.english-corpora.org/corona/x4.asp?rs=&t=88648569&ID=23075445963) |  | [LinkedIn](https://www.english-corpora.org/corona/x3.asp?node=&p=3&w10=coronavirus&w11=vaccination&r=) |  |  |  | of the highest vaccine hesitancy rates in industrialised countries -- the United Airlines mandated **coronavirus** **vaccination** for its employees and reported almost 100 per cent compliance for its 67,000 customer-facing employees |

| PAGE:   [**<<**](https://www.english-corpora.org/corona/x3.asp?node=&p=1&w10=coronavirus&w11=vaccination&r=) [**<**](https://www.english-corpora.org/corona/x3.asp?node=&p=2&w10=coronavirus&w11=vaccination&r=)   3 / 42  [**>**](https://www.english-corpora.org/corona/x3.asp?node=&p=4&w10=coronavirus&w11=vaccination&r=) [**>>**](https://www.english-corpora.org/corona/x3.asp?node=&p=42&w10=coronavirus&w11=vaccination&r=) |
| --- |

Конец формы

| FIND SAMPLE:  [100](https://www.english-corpora.org/corona/x3.asp?node=&sample=100&w10=coronavirus&w11=vaccination&r=)  [200](https://www.english-corpora.org/corona/x3.asp?node=&sample=200&w10=coronavirus&w11=vaccination&r=)  [500](https://www.english-corpora.org/corona/x3.asp?node=&sample=500&w10=coronavirus&w11=vaccination&r=)  [1000](https://www.english-corpora.org/corona/x3.asp?node=&sample=1000&w10=coronavirus&w11=vaccination&r=) PAGE:   [**<<**](https://www.english-corpora.org/corona/x3.asp?node=&p=1&w10=coronavirus&w11=vaccination&r=) [**<**](https://www.english-corpora.org/corona/x3.asp?node=&p=3&w10=coronavirus&w11=vaccination&r=)   4 / 42  [**>**](https://www.english-corpora.org/corona/x3.asp?node=&p=5&w10=coronavirus&w11=vaccination&r=) [**>>**](https://www.english-corpora.org/corona/x3.asp?node=&p=42&w10=coronavirus&w11=vaccination&r=) |  |
| --- | --- |

Начало формы

| CLICK FOR MORE CONTEXT | [**HELP**](javascript:newFeatures()) | [SAVE](javascript:chooser('s'))    [TRANSLATE](javascript:chooser('t'))    [ANALYZE](javascript:chooser('p')) |
| --- | --- | --- |

| [301](https://www.english-corpora.org/corona/x4.asp?rs=&t=33547777&ID=22739213804) | [21-12-24 US](https://www.english-corpora.org/corona/x4.asp?rs=&t=33547777&ID=22739213804) | [aljazeera.com](https://www.english-corpora.org/corona/x3.asp?node=&p=4&w10=coronavirus&w11=vaccination&r=) |  |  |  | can be no talks as long as such protests are carried out. # **Coronavirus** **vaccination** rates in France's Caribbean territories are far lower than those in mainland France, |
| --- | --- | --- | --- | --- | --- | --- |
| [302](https://www.english-corpora.org/corona/x4.asp?rs=&t=33568285&ID=22809281809) | [21-12-31 US](https://www.english-corpora.org/corona/x4.asp?rs=&t=33568285&ID=22809281809) | [sunjournal.com](https://www.english-corpora.org/corona/x3.asp?node=&p=4&w10=coronavirus&w11=vaccination&r=) |  |  |  | Steve Ewing, left, and John Tarbox greet and assist people toward the **coronavirus** **vaccination** line at Franklin Memorial Hospital in Farmington on Monday, March 23. Ewing and |
| [303](https://www.english-corpora.org/corona/x4.asp?rs=&t=43786084&ID=22789493414) | [21-12-29 US](https://www.english-corpora.org/corona/x4.asp?rs=&t=43786084&ID=22789493414) | [news4jax.com](https://www.english-corpora.org/corona/x3.asp?node=&p=4&w10=coronavirus&w11=vaccination&r=) |  |  |  | unemployment benefits if they are let go from jobs for refusing to comply with **coronavirus** **vaccination** mandates. # A notice of the rule, published in the Florida Administrative Register |
| [304](https://www.english-corpora.org/corona/x4.asp?rs=&t=53461411&ID=22727841717) | [21-12-23 US](https://www.english-corpora.org/corona/x4.asp?rs=&t=53461411&ID=22727841717) | [pix11.com](https://www.english-corpora.org/corona/x3.asp?node=&p=4&w10=coronavirus&w11=vaccination&r=) |  |  |  | vaccination mandatory for most citizens # A woman gets her shot of the COVID-19 vaccine, |
| [305](https://www.english-corpora.org/corona/x4.asp?rs=&t=63700434&ID=22808664659) | [21-12-31 US](https://www.english-corpora.org/corona/x4.asp?rs=&t=63700434&ID=22808664659) | [perezhilton.com](https://www.english-corpora.org/corona/x3.asp?node=&p=4&w10=coronavirus&w11=vaccination&r=) |  |  |  | would YOU have done?? 63700444 # **Coronavirus** **vaccination** teams |
| [306](https://www.english-corpora.org/corona/x4.asp?rs=&t=73827414&ID=22716432059) | [21-12-22 US](https://www.english-corpora.org/corona/x4.asp?rs=&t=73827414&ID=22716432059) | [yorkdispatch.com](https://www.english-corpora.org/corona/x3.asp?node=&p=4&w10=coronavirus&w11=vaccination&r=) |  |  |  | and Safety Administration said Saturday that it would not issue citations tied to its **coronavirus** **vaccination** mandate before Jan. 10, so that companies have time to adjust to and implement |
| [307](https://www.english-corpora.org/corona/x4.asp?rs=&t=88492797&ID=22725847268) | [21-12-22 US](https://www.english-corpora.org/corona/x4.asp?rs=&t=88492797&ID=22725847268) | [The Boston Globe](https://www.english-corpora.org/corona/x3.asp?node=&p=4&w10=coronavirus&w11=vaccination&r=) |  |  |  | to help control COVID-19 surge # Nurse Jill Marshall prepared a dose during a **coronavirus** **vaccination** clinic at the Museum of Science in Boston on Dec. 3. Craig F. Walker/Globe |
| [308](https://www.english-corpora.org/corona/x4.asp?rs=&t=88496747&ID=22724473062) | [21-12-22 US](https://www.english-corpora.org/corona/x4.asp?rs=&t=88496747&ID=22724473062) | [The Guardian on MSN.com](https://www.english-corpora.org/corona/x3.asp?node=&p=4&w10=coronavirus&w11=vaccination&r=) |  |  |  | # Thousands of new road signs are to be installed to guide people to **coronavirus** **vaccination** centres following an agreement between the AA and the government, PA Media reports. |
| [310](https://www.english-corpora.org/corona/x4.asp?rs=&t=88499295&ID=22737164171) | [21-12-23 US](https://www.english-corpora.org/corona/x4.asp?rs=&t=88499295&ID=22737164171) | [Fox Business](https://www.english-corpora.org/corona/x3.asp?node=&p=4&w10=coronavirus&w11=vaccination&r=)[(1)](https://www.english-corpora.org/corona/duplicates1.asp?xx=309&n=1) |  |  |  | . # The Food and Drug Administration is encouraging Americans to receive the **coronavirus** **vaccination** if eligible, and it states that it prevents COVID-19 and serious illness from infection |
| [311](https://www.english-corpora.org/corona/x4.asp?rs=&t=88500878&ID=22737879755) | [21-12-23 US](https://www.english-corpora.org/corona/x4.asp?rs=&t=88500878&ID=22737879755) | [Washington Post](https://www.english-corpora.org/corona/x3.asp?node=&p=4&w10=coronavirus&w11=vaccination&r=) |  |  |  | in a summit during which an emergency-room doctor gave participants a script for resisting **coronavirus** **vaccination**. # " If anywhere my client is represented as anti-vaccine that will be taken |
| [312](https://www.english-corpora.org/corona/x4.asp?rs=&t=88510312&ID=22749938448) | [21-12-25 US](https://www.english-corpora.org/corona/x4.asp?rs=&t=88510312&ID=22749938448) | [Washington Post](https://www.english-corpora.org/corona/x3.asp?node=&p=4&w10=coronavirus&w11=vaccination&r=) |  |  |  | funerals and evenings together despite fuel and electricity shortages. Face mask wearing and **coronavirus** **vaccination** rates remain low, while the overshadowing prospect of another conflict between Hamas and Israel |
| [313](https://www.english-corpora.org/corona/x4.asp?rs=&t=88510312&ID=22749938617) | [21-12-25 US](https://www.english-corpora.org/corona/x4.asp?rs=&t=88510312&ID=22749938617) | [Washington Post](https://www.english-corpora.org/corona/x3.asp?node=&p=4&w10=coronavirus&w11=vaccination&r=) |  |  |  | be wounded or killed by the Israeli army during border protests and clashes. **Coronavirus** **vaccination** rates have also remained low, despite efforts by Gaza's beleaguered and aid-dependent health |
| [314](https://www.english-corpora.org/corona/x4.asp?rs=&t=88513921&ID=22760227467) | [21-12-26 US](https://www.english-corpora.org/corona/x4.asp?rs=&t=88513921&ID=22760227467) | [Arkansas Democrat-Gazette](https://www.english-corpora.org/corona/x3.asp?node=&p=4&w10=coronavirus&w11=vaccination&r=) |  |  |  | teaches Army medics in Los Angeles about the variety of syringes used for a **coronavirus** **vaccination**. MUST CREDIT: Pfc. Garrison Waites/U.S. Army. # WASHINGTON -- The |
| [315](https://www.english-corpora.org/corona/x4.asp?rs=&t=88515268&ID=22764073191) | [21-12-26 US](https://www.english-corpora.org/corona/x4.asp?rs=&t=88515268&ID=22764073191) | [The Guardian on MSN.com](https://www.english-corpora.org/corona/x3.asp?node=&p=4&w10=coronavirus&w11=vaccination&r=) |  |  |  | quarter of the population have had one vaccine dose amid vaccine hesitancy. # **Coronavirus** **vaccination** teams could go door-to-door in the UK to reach those yet to have their jabs |
| [316](https://www.english-corpora.org/corona/x4.asp?rs=&t=88515268&ID=22764073648) | [21-12-26 US](https://www.english-corpora.org/corona/x4.asp?rs=&t=88515268&ID=22764073648) | [The Guardian on MSN.com](https://www.english-corpora.org/corona/x3.asp?node=&p=4&w10=coronavirus&w11=vaccination&r=) |  |  |  | quarantine. UK considering door-to-door vaccination teams, reports say # 14:52 # **Coronavirus** **vaccination** teams could go door-to-door in the UK to reach those yet to have their jabs |
| [317](https://www.english-corpora.org/corona/x4.asp?rs=&t=88518123&ID=22769697510) | [21-12-27 US](https://www.english-corpora.org/corona/x4.asp?rs=&t=88518123&ID=22769697510) | [Reuters on MSN.com](https://www.english-corpora.org/corona/x3.asp?node=&p=4&w10=coronavirus&w11=vaccination&r=) |  |  |  | will divulge on Jan. 5 the manner in which Brazil will carry out its **coronavirus** **vaccination** campaign for 5 to 11-year-olds, which was approved earlier this month. # " |
| [318](https://www.english-corpora.org/corona/x4.asp?rs=&t=88518954&ID=22771511037) | [21-12-27 US](https://www.english-corpora.org/corona/x4.asp?rs=&t=88518954&ID=22771511037) | [The Washington Post on MSN.com](https://www.english-corpora.org/corona/x3.asp?node=&p=4&w10=coronavirus&w11=vaccination&r=) |  |  |  | health guidelines, the agency said. # The South Korean pop group endorsed **coronavirus** **vaccination** during a speech in September at the United Nations. In November, the group |
| [320](https://www.english-corpora.org/corona/x4.asp?rs=&t=88523463&ID=22781723564) | [21-12-28 US](https://www.english-corpora.org/corona/x4.asp?rs=&t=88523463&ID=22781723564) | [YAHOO!News](https://www.english-corpora.org/corona/x3.asp?node=&p=4&w10=coronavirus&w11=vaccination&r=)[(1)](https://www.english-corpora.org/corona/duplicates1.asp?xx=319&n=1) |  |  |  | divulge on Jan. 5 the manner in which Brazil will carry out its **coronavirus** **vaccination** campaign for 5 to 11-year-olds, which was approved earlier this month. " Children |
| [322](https://www.english-corpora.org/corona/x4.asp?rs=&t=88527638&ID=22782899488) | [21-12-28 US](https://www.english-corpora.org/corona/x4.asp?rs=&t=88527638&ID=22782899488) | [Washington Post](https://www.english-corpora.org/corona/x3.asp?node=&p=4&w10=coronavirus&w11=vaccination&r=)[(1)](https://www.english-corpora.org/corona/duplicates1.asp?xx=321&n=1) |  |  |  | ) # The Supreme Court on Monday declined to stop New York's **coronavirus** **vaccination** mandate for health care workers that does not include an exception for religious objectors. |
| [323](https://www.english-corpora.org/corona/x4.asp?rs=&t=88531426&ID=22792061156) | [21-12-29 US](https://www.english-corpora.org/corona/x4.asp?rs=&t=88531426&ID=22792061156) | [The Boston Globe](https://www.english-corpora.org/corona/x3.asp?node=&p=4&w10=coronavirus&w11=vaccination&r=) |  |  |  | governor unless activated by the president. This means, they said, that **coronavirus** **vaccination** requirements can not be imposed by the defense secretary on their states' National Guard |
| [324](https://www.english-corpora.org/corona/x4.asp?rs=&t=88534535&ID=22793402722) | [21-12-29 US](https://www.english-corpora.org/corona/x4.asp?rs=&t=88534535&ID=22793402722) | [Washington Post](https://www.english-corpora.org/corona/x3.asp?node=&p=4&w10=coronavirus&w11=vaccination&r=) |  |  |  | more omicron outbreaks, despite having had one of the most rapid and wide-reaching **coronavirus** **vaccination** campaigns. # Advertisement # Story continues below advertisement # Israel on Monday began a |
| [326](https://www.english-corpora.org/corona/x4.asp?rs=&t=88538083&ID=22803939435) | [21-12-30 US](https://www.english-corpora.org/corona/x4.asp?rs=&t=88538083&ID=22803939435) | [YAHOO!News](https://www.english-corpora.org/corona/x3.asp?node=&p=4&w10=coronavirus&w11=vaccination&r=)[(1)](https://www.english-corpora.org/corona/duplicates1.asp?xx=325&n=1) |  |  |  | add a further 4,000 " super surge " beds. # A pop-up **coronavirus** **vaccination** centre at the Redbridge Town Hall, east London. (Getty) # The |
| [327](https://www.english-corpora.org/corona/x4.asp?rs=&t=88542309&ID=22812242315) | [21-12-31 US](https://www.english-corpora.org/corona/x4.asp?rs=&t=88542309&ID=22812242315) | [Fox News](https://www.english-corpora.org/corona/x3.asp?node=&p=4&w10=coronavirus&w11=vaccination&r=) |  |  |  | " system used by the CDC to track patients' health after receiving the **coronavirus** **vaccination**. The group says the CDC has already provided Oracle, a government contractor, |
| [328](https://www.english-corpora.org/corona/x4.asp?rs=&t=88544976&ID=22814838145) | [21-12-31 US](https://www.english-corpora.org/corona/x4.asp?rs=&t=88544976&ID=22814838145) | [Reuters](https://www.english-corpora.org/corona/x3.asp?node=&p=4&w10=coronavirus&w11=vaccination&r=) |  |  |  | , Dec 17 (Reuters) - Boeing Co (BA.N) suspended its **coronavirus** **vaccination** requirement for U.S.-based employees, the U.S. planemaker said on Friday, capping weeks of |
| [329](https://www.english-corpora.org/corona/x4.asp?rs=&t=43767125&ID=22727127513) | [21-12-23 GB](https://www.english-corpora.org/corona/x4.asp?rs=&t=43767125&ID=22727127513) | [countypress.co.uk](https://www.english-corpora.org/corona/x3.asp?node=&p=4&w10=coronavirus&w11=vaccination&r=) |  |  |  | from **coronavirus** **vaccination** centre Power cables stolen from coronavirus vaccination centre # Power cables are believed to |
| [330](https://www.english-corpora.org/corona/x4.asp?rs=&t=43767125&ID=22727127521) | [21-12-23 GB](https://www.english-corpora.org/corona/x4.asp?rs=&t=43767125&ID=22727127521) | [countypress.co.uk](https://www.english-corpora.org/corona/x3.asp?node=&p=4&w10=coronavirus&w11=vaccination&r=) |  |  |  | from coronavirus vaccination centre Power cables stolen from **coronavirus** **vaccination** centre # Power cables are believed to have been stolen from a vaccination centre in |
| [331](https://www.english-corpora.org/corona/x4.asp?rs=&t=43772500&ID=22753351457) | [21-12-26 GB](https://www.english-corpora.org/corona/x4.asp?rs=&t=43772500&ID=22753351457) | [walesonline.co.uk](https://www.english-corpora.org/corona/x3.asp?node=&p=4&w10=coronavirus&w11=vaccination&r=) |  |  |  | on our understanding. You can unsubscribe at any time. More info # **Coronavirus** **vaccination** teams could go door-to-door to reach those yet to have their jabs, reports have |
| [332](https://www.english-corpora.org/corona/x4.asp?rs=&t=43776216&ID=22764646756) | [21-12-27 GB](https://www.english-corpora.org/corona/x4.asp?rs=&t=43776216&ID=22764646756) | [independent.co.uk](https://www.english-corpora.org/corona/x3.asp?node=&p=4&w10=coronavirus&w11=vaccination&r=) |  |  |  | will divulge on 5 January the manner in which Brazil will carry out its **coronavirus** **vaccination** campaign for 5 to 11-year-olds, which was approved earlier this month. # " |
| [333](https://www.english-corpora.org/corona/x4.asp?rs=&t=43781911&ID=22784705176) | [21-12-29 GB](https://www.english-corpora.org/corona/x4.asp?rs=&t=43781911&ID=22784705176) | [lancashiretelegraph.co.uk](https://www.english-corpora.org/corona/x3.asp?node=&p=4&w10=coronavirus&w11=vaccination&r=) |  |  |  | Wales's highest mountain, in snowy conditions (Ben Birchall/PA) # A **coronavirus** **vaccination** centre opened at Cwmbran Stadium in South Wales as the rollout gathered pace. # |
| [334](https://www.english-corpora.org/corona/x4.asp?rs=&t=43790165&ID=22807773114) | [21-12-31 GB](https://www.english-corpora.org/corona/x4.asp?rs=&t=43790165&ID=22807773114) | [expressandstar.com](https://www.english-corpora.org/corona/x3.asp?node=&p=4&w10=coronavirus&w11=vaccination&r=) |  |  |  | Families with secondary school age children are being reminded to take them to walk-in **coronavirus** **vaccination** centres for second doses. # Subscribe to our daily newsletter! # Parents and |
| [335](https://www.english-corpora.org/corona/x4.asp?rs=&t=53462561&ID=22730262224) | [21-12-23 GB](https://www.english-corpora.org/corona/x4.asp?rs=&t=53462561&ID=22730262224) | [news.sky.com](https://www.english-corpora.org/corona/x3.asp?node=&p=4&w10=coronavirus&w11=vaccination&r=) |  |  |  | gives out 500m jabs # The United States has now given out 500 million **coronavirus** **vaccination** shots. # In total, 500,222,330 doses have been put into arms in the |
| [336](https://www.english-corpora.org/corona/x4.asp?rs=&t=63689717&ID=22765591623) | [21-12-27 GB](https://www.english-corpora.org/corona/x4.asp?rs=&t=63689717&ID=22765591623) | [aol.co.uk](https://www.english-corpora.org/corona/x3.asp?node=&p=4&w10=coronavirus&w11=vaccination&r=) |  |  |  | # Thousands of new road signs are to be installed to guide people to **coronavirus** **vaccination** centres following an agreement between the AA and the Government. # This follows requests |
| [337](https://www.english-corpora.org/corona/x4.asp?rs=&t=88494040&ID=22723265973) | [21-12-22 GB](https://www.english-corpora.org/corona/x4.asp?rs=&t=88494040&ID=22723265973) | [The Guardian](https://www.english-corpora.org/corona/x3.asp?node=&p=4&w10=coronavirus&w11=vaccination&r=) |  |  |  | a first dose of a Covid vaccine this month, three weeks after the **coronavirus** **vaccination** programme was opened up to those in their 20s. # Vaccines minister, Nadhim |
| [338](https://www.english-corpora.org/corona/x4.asp?rs=&t=63691999&ID=22790112248) | [21-12-29 IE](https://www.english-corpora.org/corona/x4.asp?rs=&t=63691999&ID=22790112248) | [trtworld.com](https://www.english-corpora.org/corona/x3.asp?node=&p=4&w10=coronavirus&w11=vaccination&r=) |  |  |  | will divulge on January 5 the manner in which Brazil will carry out its **coronavirus** **vaccination** campaign for 5 to 11-year-olds, which was approved earlier this month. # Vaccination |
| [339](https://www.english-corpora.org/corona/x4.asp?rs=&t=33565449&ID=22797518660) | [21-12-30 AU](https://www.english-corpora.org/corona/x4.asp?rs=&t=33565449&ID=22797518660) | [riverineherald.com.au](https://www.english-corpora.org/corona/x3.asp?node=&p=4&w10=coronavirus&w11=vaccination&r=) |  |  |  | given a fifth reason to leave home during this lockdown -- to get a **coronavirus** **vaccination** -- but the lockdown was to cope with the new Delta strain, which was |
| [340](https://www.english-corpora.org/corona/x4.asp?rs=&t=73841878&ID=22779241079) | [21-12-28 AU](https://www.english-corpora.org/corona/x4.asp?rs=&t=73841878&ID=22779241079) | [au.finance.yahoo.com](https://www.english-corpora.org/corona/x3.asp?node=&p=4&w10=coronavirus&w11=vaccination&r=) |  |  |  | will divulge on Jan. 5 the manner in which Brazil will carry out its **coronavirus** **vaccination** campaign for 5 to 11-year-olds, which was approved earlier this month. " Children |
| [341](https://www.english-corpora.org/corona/x4.asp?rs=&t=33558169&ID=22777721369) | [21-12-28 NZ](https://www.english-corpora.org/corona/x4.asp?rs=&t=33558169&ID=22777721369) | [radionz.co.nz](https://www.english-corpora.org/corona/x3.asp?node=&p=4&w10=coronavirus&w11=vaccination&r=) |  |  |  | will divulge on 5 January the manner in which Brazil will carry out its **coronavirus** **vaccination** campaign for five to 11 year olds, which was approved earlier this month. |
| [342](https://www.english-corpora.org/corona/x4.asp?rs=&t=63698799&ID=22799741771) | [21-12-30 IN](https://www.english-corpora.org/corona/x4.asp?rs=&t=63698799&ID=22799741771) | [ptinews.com](https://www.english-corpora.org/corona/x3.asp?node=&p=4&w10=coronavirus&w11=vaccination&r=) |  |  |  | ensuring Covid protocol. # The CEC also asked the state to step up **coronavirus** **vaccination**. # The number of polling booths will be increased and the voting time enhanced |
| [343](https://www.english-corpora.org/corona/x4.asp?rs=&t=73838373&ID=22759830491) | [21-12-26 IN](https://www.english-corpora.org/corona/x4.asp?rs=&t=73838373&ID=22759830491) | [outlookindia.com](https://www.english-corpora.org/corona/x3.asp?node=&p=4&w10=coronavirus&w11=vaccination&r=) |  |  |  | experts. # 2. Modi announced that the country will start administering the **coronavirus** **vaccination** for the children belonging to the age group 15-18 from January 3, 2022. |
| [344](https://www.english-corpora.org/corona/x4.asp?rs=&t=73847794&ID=22790891967) | [21-12-29 IN](https://www.english-corpora.org/corona/x4.asp?rs=&t=73847794&ID=22790891967) | [businesstoday.in](https://www.english-corpora.org/corona/x3.asp?node=&p=4&w10=coronavirus&w11=vaccination&r=) |  |  |  | vaccination coverage has exceeded 143.15 crore as of 7 am today with the administration of 64.61 |
| [345](https://www.english-corpora.org/corona/x4.asp?rs=&t=73847794&ID=22790892047) | [21-12-29 IN](https://www.english-corpora.org/corona/x4.asp?rs=&t=73847794&ID=22790892047) | [businesstoday.in](https://www.english-corpora.org/corona/x3.asp?node=&p=4&w10=coronavirus&w11=vaccination&r=) |  |  |  | , # Updated Dec 29, 2021, 11:14 AM IST # India's **coronavirus** **vaccination** coverage has exceeded 143.15 crore as of 7 am today with the administration of 64.61 |
| [346](https://www.english-corpora.org/corona/x4.asp?rs=&t=43765816&ID=22726983830) | [21-12-23 HK](https://www.english-corpora.org/corona/x4.asp?rs=&t=43765816&ID=22726983830) | [hongkongfp.com](https://www.english-corpora.org/corona/x3.asp?node=&p=4&w10=coronavirus&w11=vaccination&r=) |  |  |  | nasal and throat swabs -- will be valid for three days only. # **Coronavirus** **vaccination** in Hong Kong. Photo: GovHK. # " In view of the increasing |
| [347](https://www.english-corpora.org/corona/x4.asp?rs=&t=63684099&ID=22759317209) | [21-12-26 ZA](https://www.english-corpora.org/corona/x4.asp?rs=&t=63684099&ID=22759317209) | [citizen.co.za](https://www.english-corpora.org/corona/x3.asp?node=&p=4&w10=coronavirus&w11=vaccination&r=) |  |  |  | # A health worker prepares a dose of the Covid-19 vaccine at a pop-up **coronavirus** **vaccination** centre at the Redbridge Town Hall, east London on December 25, 2021. - |
| [348](https://www.english-corpora.org/corona/x4.asp?rs=&t=53467384&ID=22758729214) | [21-12-26 NG](https://www.english-corpora.org/corona/x4.asp?rs=&t=53467384&ID=22758729214) | [leadership.ng](https://www.english-corpora.org/corona/x3.asp?node=&p=4&w10=coronavirus&w11=vaccination&r=) |  |  |  | virus, considering her weak health and government systems and the general apathy to **coronavirus** **vaccination** and poor observance of social and physical distancing protocols. # This year's Christmas |
| [349](https://www.english-corpora.org/corona/x4.asp?rs=&t=33570746&ID=22817250871) | [22-01-01 US](https://www.english-corpora.org/corona/x4.asp?rs=&t=33570746&ID=22817250871) | [wtop.com](https://www.english-corpora.org/corona/x3.asp?node=&p=4&w10=coronavirus&w11=vaccination&r=) |  |  |  | # In late 2020, the Centers for Disease Control and Prevention began reporting **coronavirus** **vaccination** data for all 50 states, various U.S. territories and federal agencies. Updated every |
| [350](https://www.english-corpora.org/corona/x4.asp?rs=&t=33589592&ID=22882355848) | [22-01-06 US](https://www.english-corpora.org/corona/x4.asp?rs=&t=33589592&ID=22882355848) | [washingtonexaminer.com](https://www.english-corpora.org/corona/x3.asp?node=&p=4&w10=coronavirus&w11=vaccination&r=) |  |  |  | sailors and more than 250 Marines have been discharged for refusing to receive the **coronavirus** **vaccination**. # The Navy announced its first round of discharges on Wednesday, while the |
| [351](https://www.english-corpora.org/corona/x4.asp?rs=&t=33596623&ID=22890259875) | [22-01-07 US](https://www.english-corpora.org/corona/x4.asp?rs=&t=33596623&ID=22890259875) | [aljazeera.com](https://www.english-corpora.org/corona/x3.asp?node=&p=4&w10=coronavirus&w11=vaccination&r=) |  |  |  | taken up challenges to efforts by the administration of President Joe Biden to improve **coronavirus** **vaccination** rates as the Omicron variant spreads widely Evan Vucci/AP Photo # The United States Supreme |
| [352](https://www.english-corpora.org/corona/x4.asp?rs=&t=33598770&ID=22915857407) | [22-01-08 US](https://www.english-corpora.org/corona/x4.asp?rs=&t=33598770&ID=22915857407) | [arkansasonline.com](https://www.english-corpora.org/corona/x3.asp?node=&p=4&w10=coronavirus&w11=vaccination&r=) |  |  |  | * David Hodges of Lewes, Del., a paramedic who worked at a **coronavirus** **vaccination** site, was charged with pocketing nearly $1,300 after selling copied or stolen blank vaccination |
| [353](https://www.english-corpora.org/corona/x4.asp?rs=&t=73855188&ID=22820233280) | [22-01-01 US](https://www.english-corpora.org/corona/x4.asp?rs=&t=73855188&ID=22820233280) | [heavy.com](https://www.english-corpora.org/corona/x3.asp?node=&p=4&w10=coronavirus&w11=vaccination&r=) |  |  |  | , the Vikings quarterback was never dishonest or ambiguous about his misgivings around the **coronavirus** **vaccination**. Before the season, Cousins said he would not be vaccinated. He added |
| [354](https://www.english-corpora.org/corona/x4.asp?rs=&t=73865387&ID=22841933936) | [22-01-03 US](https://www.english-corpora.org/corona/x4.asp?rs=&t=73865387&ID=22841933936) | [forward.com](https://www.english-corpora.org/corona/x3.asp?node=&p=4&w10=coronavirus&w11=vaccination&r=) |  |  |  | dose of the coronavirus vaccine Sunday. # While the ability of a fourth **coronavirus** **vaccination** to boost immunity has not been proven, the question is currently being studied at |
| [355](https://www.english-corpora.org/corona/x4.asp?rs=&t=73873641&ID=22867846093) | [22-01-05 US](https://www.english-corpora.org/corona/x4.asp?rs=&t=73873641&ID=22867846093) | [gazette.com](https://www.english-corpora.org/corona/x3.asp?node=&p=4&w10=coronavirus&w11=vaccination&r=) |  |  |  | service branch that are discharged from their positions over their refusal to get the **coronavirus** **vaccination** can not be discharged under less than the general designation as a result of a |
| [357](https://www.english-corpora.org/corona/x4.asp?rs=&t=73875766&ID=22868224939) | [22-01-05 US](https://www.english-corpora.org/corona/x4.asp?rs=&t=73875766&ID=22868224939) | [gazette.com](https://www.english-corpora.org/corona/x3.asp?node=&p=4&w10=coronavirus&w11=vaccination&r=)[(1)](https://www.english-corpora.org/corona/duplicates1.asp?xx=356&n=1) |  |  |  | with the implicit design to keep whites ahead of minorities, to the **coronavirus** **vaccination** mandate. # The crescendo of the conversation regarding critical race theory in the military |
| [358](https://www.english-corpora.org/corona/x4.asp?rs=&t=73877223&ID=22880630924) | [22-01-06 US](https://www.english-corpora.org/corona/x4.asp?rs=&t=73877223&ID=22880630924) | [sports.yahoo.com](https://www.english-corpora.org/corona/x3.asp?node=&p=4&w10=coronavirus&w11=vaccination&r=) |  |  |  | he only had himself to blame for being denied entry to Australia over his **coronavirus** **vaccination** status, as the tennis world was divided over whether to sympathise with the world |
| [359](https://www.english-corpora.org/corona/x4.asp?rs=&t=88549003&ID=22820770865) | [22-01-01 US](https://www.english-corpora.org/corona/x4.asp?rs=&t=88549003&ID=22820770865) | [YAHOO!News](https://www.english-corpora.org/corona/x3.asp?node=&p=4&w10=coronavirus&w11=vaccination&r=) |  |  |  | fan12 and older at Wolverines home games will need proof of a **coronavirus** **vaccination** or a negative test result within 72 hours of the event. Those without either |
| [360](https://www.english-corpora.org/corona/x4.asp?rs=&t=88549132&ID=22820790764) | [22-01-01 US](https://www.english-corpora.org/corona/x4.asp?rs=&t=88549132&ID=22820790764) | [New York Times](https://www.english-corpora.org/corona/x3.asp?node=&p=4&w10=coronavirus&w11=vaccination&r=) |  |  |  | Prime Minister Boris Johnson of Britain during a visit to a National Health Service **coronavirus** **vaccination** center near Ramsgate, England, on Thursday.Credit... Pool photo by Leon Neal # |
| [361](https://www.english-corpora.org/corona/x4.asp?rs=&t=88556035&ID=22832418980) | [22-01-02 US](https://www.english-corpora.org/corona/x4.asp?rs=&t=88556035&ID=22832418980) | [Detroit Free Press on MSN.com](https://www.english-corpora.org/corona/x3.asp?node=&p=4&w10=coronavirus&w11=vaccination&r=) |  |  |  | and fans 12 and older at Wolverines home games will need proof of a **coronavirus** **vaccination** or a negative test result within 72 hours of the event. Those without either |
| [362](https://www.english-corpora.org/corona/x4.asp?rs=&t=88558693&ID=22845807904) | [22-01-03 US](https://www.english-corpora.org/corona/x4.asp?rs=&t=88558693&ID=22845807904) | [Patch](https://www.english-corpora.org/corona/x3.asp?node=&p=4&w10=coronavirus&w11=vaccination&r=) |  |  |  | Thursday, which is walk-in only. # DELAWARE COUNTY, PA -- Several **coronavirus** **vaccination** clinics are being held in Delaware County this week as the coronavirus and its variants |
| [363](https://www.english-corpora.org/corona/x4.asp?rs=&t=88559616&ID=22843333652) | [22-01-03 US](https://www.english-corpora.org/corona/x4.asp?rs=&t=88559616&ID=22843333652) | [Houston Chronicle](https://www.english-corpora.org/corona/x3.asp?node=&p=4&w10=coronavirus&w11=vaccination&r=) |  |  |  | the Capitol calling for lawmakers to let children have a say in the upcoming **coronavirus** **vaccination** mandate for schoolchildren, at a demonstration held in Sacramento, Calif., Monday, |
| [364](https://www.english-corpora.org/corona/x4.asp?rs=&t=88559616&ID=22843333720) | [22-01-03 US](https://www.english-corpora.org/corona/x4.asp?rs=&t=88559616&ID=22843333720) | [Houston Chronicle](https://www.english-corpora.org/corona/x3.asp?node=&p=4&w10=coronavirus&w11=vaccination&r=) |  |  |  | , joins her mother in traveling from Visalia to protest the state's upcoming **coronavirus** **vaccination** mandate for schoolchildren, at a demonstration held at the Capitol in Sacramento, Calif. |
| [365](https://www.english-corpora.org/corona/x4.asp?rs=&t=88562308&ID=22843722903) | [22-01-03 US](https://www.english-corpora.org/corona/x4.asp?rs=&t=88562308&ID=22843722903) | [The Motley Fool](https://www.english-corpora.org/corona/x3.asp?node=&p=4&w10=coronavirus&w11=vaccination&r=) |  |  |  | monthly active users (MAUs). This decline was the expected reaction as **coronavirus** **vaccination** rates kept ticking higher and people returned to some activities outside their homes. But |
| [366](https://www.english-corpora.org/corona/x4.asp?rs=&t=88563187&ID=22846660973) | [22-01-03 US](https://www.english-corpora.org/corona/x4.asp?rs=&t=88563187&ID=22846660973) | [New York Times](https://www.english-corpora.org/corona/x3.asp?node=&p=4&w10=coronavirus&w11=vaccination&r=) |  |  |  | port neighborhood, La Goulette, citing the president's troubled rollout of a **coronavirus** **vaccination** registry and plans to freeze public sector salaries. " He doesn't live in |
| [367](https://www.english-corpora.org/corona/x4.asp?rs=&t=88569014&ID=22853897290) | [22-01-04 US](https://www.english-corpora.org/corona/x4.asp?rs=&t=88569014&ID=22853897290) | [NPR](https://www.english-corpora.org/corona/x3.asp?node=&p=4&w10=coronavirus&w11=vaccination&r=) |  |  |  | Djokovic, the top-ranked men's tennis player who is also famously skeptical about **coronavirus** **vaccination**, will play in the Australian Open later this month, after receiving a special |
| [369](https://www.english-corpora.org/corona/x4.asp?rs=&t=88571828&ID=22875595345) | [22-01-05 US](https://www.english-corpora.org/corona/x4.asp?rs=&t=88571828&ID=22875595345) | [Japan Today](https://www.english-corpora.org/corona/x3.asp?node=&p=4&w10=coronavirus&w11=vaccination&r=)[(1)](https://www.english-corpora.org/corona/duplicates1.asp?xx=368&n=1) |  |  |  | work in its New York City headquarters offices to receive at least one **coronavirus** **vaccination** shot by Dec. 27, and is scrapping the option of regular testing for employees |
| [370](https://www.english-corpora.org/corona/x4.asp?rs=&t=88573094&ID=22878098798) | [22-01-05 US](https://www.english-corpora.org/corona/x4.asp?rs=&t=88573094&ID=22878098798) | [CNN on MSN.com](https://www.english-corpora.org/corona/x3.asp?node=&p=4&w10=coronavirus&w11=vaccination&r=) |  |  |  | following an outcry over his controversial " medical exemption " from the country's **coronavirus** **vaccination** rules granted by the competition's organizers. # Djokovic, the men's tennis |
| [371](https://www.english-corpora.org/corona/x4.asp?rs=&t=88573860&ID=22875946820) | [22-01-05 US](https://www.english-corpora.org/corona/x4.asp?rs=&t=88573860&ID=22875946820) | [The Washington Post on MSN.com](https://www.english-corpora.org/corona/x3.asp?node=&p=4&w10=coronavirus&w11=vaccination&r=) |  |  |  | fortune. Although most of today's travelers are obsessed with carrying proof of **coronavirus** **vaccination** or negative test results, few take any other health information. That's a |
| [372](https://www.english-corpora.org/corona/x4.asp?rs=&t=88574803&ID=22874818904) | [22-01-05 US](https://www.english-corpora.org/corona/x4.asp?rs=&t=88574803&ID=22874818904) | [San Francisco Chronicle](https://www.english-corpora.org/corona/x3.asp?node=&p=4&w10=coronavirus&w11=vaccination&r=) |  |  |  | canceled his visa because he failed to meet the requirements for an exemption to **coronavirus** **vaccination** rules. Health Minister Greg Hunt said the visa cancellation followed a review of Djokovic |
| [373](https://www.english-corpora.org/corona/x4.asp?rs=&t=88574921&ID=22874419789) | [22-01-05 US](https://www.english-corpora.org/corona/x4.asp?rs=&t=88574921&ID=22874419789) | [New York Daily News](https://www.english-corpora.org/corona/x3.asp?node=&p=4&w10=coronavirus&w11=vaccination&r=) |  |  |  | a fake **coronavirus** **vaccination** card has become a state crime in New York thanks to a bill signed into |
| [374](https://www.english-corpora.org/corona/x4.asp?rs=&t=88574921&ID=22874419836) | [22-01-05 US](https://www.english-corpora.org/corona/x4.asp?rs=&t=88574921&ID=22874419836) | [New York Daily News](https://www.english-corpora.org/corona/x3.asp?node=&p=4&w10=coronavirus&w11=vaccination&r=) |  |  |  | with a package of other pandemic response-related measures, makes the falsification of a **coronavirus** **vaccination** card a class A misdemeanor. It also establishes a new felony computer-tampering charge for |
| [375](https://www.english-corpora.org/corona/x4.asp?rs=&t=88574921&ID=22874419871) | [22-01-05 US](https://www.english-corpora.org/corona/x4.asp?rs=&t=88574921&ID=22874419871) | [New York Daily News](https://www.english-corpora.org/corona/x3.asp?node=&p=4&w10=coronavirus&w11=vaccination&r=) |  |  |  | pertaining to COVID-19 vaccine records. # Advertisement # Producing or using a fake **coronavirus** **vaccination** card has become a state crime in New York thanks to a bill signed into |
| [376](https://www.english-corpora.org/corona/x4.asp?rs=&t=88576630&ID=22883974360) | [22-01-06 US](https://www.english-corpora.org/corona/x4.asp?rs=&t=88576630&ID=22883974360) | [YAHOO!News](https://www.english-corpora.org/corona/x3.asp?node=&p=4&w10=coronavirus&w11=vaccination&r=) |  |  |  | he only had himself to blame for being denied entry to Australia over his **coronavirus** **vaccination** status, as the tennis world was divided over whether to sympathise with the world |
| [378](https://www.english-corpora.org/corona/x4.asp?rs=&t=88577261&ID=22885641309) | [22-01-06 US](https://www.english-corpora.org/corona/x4.asp?rs=&t=88577261&ID=22885641309) | [Washington Post](https://www.english-corpora.org/corona/x3.asp?node=&p=4&w10=coronavirus&w11=vaccination&r=)[(1)](https://www.english-corpora.org/corona/duplicates1.asp?xx=377&n=1) |  |  |  | rival Novak Djokovic's visa to Australia was canceled after uproar about his **coronavirus** **vaccination** status. Djokovic is preparing an appeal on Monday as the world No. 1 is |
| [380](https://www.english-corpora.org/corona/x4.asp?rs=&t=88579777&ID=22888423809) | [22-01-06 US](https://www.english-corpora.org/corona/x4.asp?rs=&t=88579777&ID=22888423809) | [Yahoo! Sports](https://www.english-corpora.org/corona/x3.asp?node=&p=4&w10=coronavirus&w11=vaccination&r=)[(1)](https://www.english-corpora.org/corona/duplicates1.asp?xx=379&n=1) |  |  |  | only had himself to blame for being denied entry to Australia over his **coronavirus** **vaccination** status, as the tennis world was divided over whether to sympathise with the world |
| [382](https://www.english-corpora.org/corona/x4.asp?rs=&t=88585109&ID=22906820451) | [22-01-07 US](https://www.english-corpora.org/corona/x4.asp?rs=&t=88585109&ID=22906820451) | [Houston Public Media](https://www.english-corpora.org/corona/x3.asp?node=&p=4&w10=coronavirus&w11=vaccination&r=)[(1)](https://www.english-corpora.org/corona/duplicates1.asp?xx=381&n=1) |  |  |  | rise in cases, he said, due to the state's lower **coronavirus** **vaccination** rate. According to the CDC, nearly 62% of the U.S. population is considered |
| [383](https://www.english-corpora.org/corona/x4.asp?rs=&t=88585141&ID=22907081292) | [22-01-07 US](https://www.english-corpora.org/corona/x4.asp?rs=&t=88585141&ID=22907081292) | [New York Times](https://www.english-corpora.org/corona/x3.asp?node=&p=4&w10=coronavirus&w11=vaccination&r=) |  |  |  | oral arguments over efforts to overturn two major Biden Administration policies designed to raise **coronavirus** **vaccination** rates: its vaccine-or-testing mandate aimed at large employers and a vaccination requirement for some |
| [384](https://www.english-corpora.org/corona/x4.asp?rs=&t=53508670&ID=22917276386) | [22-01-08 CA](https://www.english-corpora.org/corona/x4.asp?rs=&t=53508670&ID=22917276386) | [ca.news.yahoo.com](https://www.english-corpora.org/corona/x3.asp?node=&p=4&w10=coronavirus&w11=vaccination&r=) |  |  |  | # A Southern California man was arrested after he recently attacked workers at a **coronavirus** **vaccination** clinic, allegedly calling them " murderers " and falsely accusing the staffers of causing |
| [385](https://www.english-corpora.org/corona/x4.asp?rs=&t=53508670&ID=22917276843) | [22-01-08 CA](https://www.english-corpora.org/corona/x4.asp?rs=&t=53508670&ID=22917276843) | [ca.news.yahoo.com](https://www.english-corpora.org/corona/x3.asp?node=&p=4&w10=coronavirus&w11=vaccination&r=) |  |  |  | . # The United States continues to deal with a persistent partisan divide over **coronavirus** **vaccination**, even as the highly transmissible omicron variant causes case numbers to surge nationwide. |
| [386](https://www.english-corpora.org/corona/x4.asp?rs=&t=53509758&ID=22914714551) | [22-01-08 CA](https://www.english-corpora.org/corona/x4.asp?rs=&t=53509758&ID=22914714551) | [ca.news.yahoo.com](https://www.english-corpora.org/corona/x3.asp?node=&p=4&w10=coronavirus&w11=vaccination&r=) |  |  |  | May Be Late After **Coronavirus** **Vaccination**, Study Suggests # Roni Caryn Rabin # January 7, 2022, 6:14 a.m. |
| [387](https://www.english-corpora.org/corona/x4.asp?rs=&t=43795734&ID=22837576563) | [22-01-03 GB](https://www.english-corpora.org/corona/x4.asp?rs=&t=43795734&ID=22837576563) | [dailyrecord.co.uk](https://www.english-corpora.org/corona/x3.asp?node=&p=4&w10=coronavirus&w11=vaccination&r=) |  |  |  | ask for your banking details or identity documents, email you to arrange your **coronavirus** **vaccination**, or turn up at your residence unannounced. " |
| [388](https://www.english-corpora.org/corona/x4.asp?rs=&t=43812267&ID=22901679153) | [22-01-07 GB](https://www.english-corpora.org/corona/x4.asp?rs=&t=43812267&ID=22901679153) | [politico.eu](https://www.english-corpora.org/corona/x3.asp?node=&p=4&w10=coronavirus&w11=vaccination&r=) |  |  |  | JABS FOR PEOPLE OVER 50: Italian ministers have passed a decree making a **coronavirus** **vaccination** mandatory for everybody over the age of 50, with Ansa reporting that people who |
| [389](https://www.english-corpora.org/corona/x4.asp?rs=&t=43813030&ID=22890556451) | [22-01-07 GB](https://www.english-corpora.org/corona/x4.asp?rs=&t=43813030&ID=22890556451) | [dailyrecord.co.uk](https://www.english-corpora.org/corona/x3.asp?node=&p=4&w10=coronavirus&w11=vaccination&r=) |  |  |  | The latest local vaccination data shows that 332,875 people have had at least one **coronavirus** **vaccination**, 307,664 have received at least two, while 237,661 have also received a third |
| [390](https://www.english-corpora.org/corona/x4.asp?rs=&t=43813388&ID=22901849648) | [22-01-07 GB](https://www.english-corpora.org/corona/x4.asp?rs=&t=43813388&ID=22901849648) | [shropshirestar.com](https://www.english-corpora.org/corona/x3.asp?node=&p=4&w10=coronavirus&w11=vaccination&r=) |  |  |  | over-65s had received the booster. # Subscribe to our daily newsletter! # **Coronavirus** **vaccination** # Vaccine boosters are 90% effective against admission to hospital from the Omicron variant for |
| [391](https://www.english-corpora.org/corona/x4.asp?rs=&t=53499942&ID=22865960800) | [22-01-05 LK](https://www.english-corpora.org/corona/x4.asp?rs=&t=53499942&ID=22865960800) | [nation.lk](https://www.english-corpora.org/corona/x3.asp?node=&p=4&w10=coronavirus&w11=vaccination&r=) |  |  |  | organize a rescue more promptly. # Executives warned Congress last month that a **coronavirus** **vaccination** mandate threatened to leave it short-staffed, and the mandate was suspended on December 14 |
| [392](https://www.english-corpora.org/corona/x4.asp?rs=&t=53505774&ID=22903052971) | [22-01-07 PK](https://www.english-corpora.org/corona/x4.asp?rs=&t=53505774&ID=22903052971) | [samaa.tv](https://www.english-corpora.org/corona/x3.asp?node=&p=4&w10=coronavirus&w11=vaccination&r=) |  |  |  | of the population. In one such move, the Sindh government has made **coronavirus** **vaccination** mandatory for students above and of the age of 12 years. According to a |
| [394](https://www.english-corpora.org/corona/x4.asp?rs=&t=53507610&ID=22892215440) | [22-01-07 PK](https://www.english-corpora.org/corona/x4.asp?rs=&t=53507610&ID=22892215440) | [pakistantoday.com.pk](https://www.english-corpora.org/corona/x3.asp?node=&p=4&w10=coronavirus&w11=vaccination&r=)[(1)](https://www.english-corpora.org/corona/duplicates1.asp?xx=393&n=1) |  |  |  | in Sindh, the Sindh government has further decided to launch a door-to-door **coronavirus** **vaccination** campaign for women amid rising cases of the Omicron variant in Karachi and other major |
| [395](https://www.english-corpora.org/corona/x4.asp?rs=&t=53510465&ID=22914842249) | [22-01-08 PK](https://www.english-corpora.org/corona/x4.asp?rs=&t=53510465&ID=22914842249) | [samaa.tv](https://www.english-corpora.org/corona/x3.asp?node=&p=4&w10=coronavirus&w11=vaccination&r=) |  |  |  | It was 4.74% on December 31, 2021. The provincial government has made **coronavirus** **vaccination** mandatory for students above and of the age of 12 years. Farmers are complaining |
| [396](https://www.english-corpora.org/corona/x4.asp?rs=&t=53488420&ID=22824926750) | [22-01-02 BD](https://www.english-corpora.org/corona/x4.asp?rs=&t=53488420&ID=22824926750) | [theindependentbd.com](https://www.english-corpora.org/corona/x3.asp?node=&p=4&w10=coronavirus&w11=vaccination&r=) |  |  |  | and about four and a half crore people have received double dose. The **coronavirus** **vaccination** programme in the country has been going on for 10 months and as of December |
| [397](https://www.english-corpora.org/corona/x4.asp?rs=&t=53488420&ID=22824927907) | [22-01-02 BD](https://www.english-corpora.org/corona/x4.asp?rs=&t=53488420&ID=22824927907) | [theindependentbd.com](https://www.english-corpora.org/corona/x3.asp?node=&p=4&w10=coronavirus&w11=vaccination&r=) |  |  |  | registration of vaccination and about four and a half crore people have receiveddouble dose.The **coronavirus** **vaccination** programme in the country has been going on for 10 months and as of December |
| [398](https://www.english-corpora.org/corona/x4.asp?rs=&t=43804526&ID=22859412147) | [22-01-05 SG](https://www.english-corpora.org/corona/x4.asp?rs=&t=43804526&ID=22859412147) | [straitstimes.com](https://www.english-corpora.org/corona/x3.asp?node=&p=4&w10=coronavirus&w11=vaccination&r=) |  |  |  | BLOOMBERG) - A year after rolling out the world's fastest **coronavirus** **vaccination** programme, Israel again finds itself a global test case as it begins widely delivering |
| [399](https://www.english-corpora.org/corona/x4.asp?rs=&t=43799845&ID=22849558563) | [22-01-04 HK](https://www.english-corpora.org/corona/x4.asp?rs=&t=43799845&ID=22849558563) | [hongkongfp.com](https://www.english-corpora.org/corona/x3.asp?node=&p=4&w10=coronavirus&w11=vaccination&r=) |  |  |  | teaching has huge impacts on students, especially pupils from lower-income backgrounds. # **Coronavirus** **vaccination** in Hong Kong. Photo: GovHK. # Ramon Yuen, the Democratic Party |
| [400](https://www.english-corpora.org/corona/x4.asp?rs=&t=63717359&ID=22866429936) | [22-01-05 ZA](https://www.english-corpora.org/corona/x4.asp?rs=&t=63717359&ID=22866429936) | [ewn.co.za](https://www.english-corpora.org/corona/x3.asp?node=&p=4&w10=coronavirus&w11=vaccination&r=) |  |  |  | . # A health worker administers a dose of the AstraZeneca/Oxford vaccine at a **coronavirus** **vaccination** centre at the Fazl Mosque in southwest London on March 23, 2021, on |

| PAGE:   [**<<**](https://www.english-corpora.org/corona/x3.asp?node=&p=1&w10=coronavirus&w11=vaccination&r=) [**<**](https://www.english-corpora.org/corona/x3.asp?node=&p=3&w10=coronavirus&w11=vaccination&r=)   4 / 42  [**>**](https://www.english-corpora.org/corona/x3.asp?node=&p=5&w10=coronavirus&w11=vaccination&r=) [**>>**](https://www.english-corpora.org/corona/x3.asp?node=&p=42&w10=coronavirus&w11=vaccination&r=) |
| --- |

Конец формы

| FIND SAMPLE:  [100](https://www.english-corpora.org/corona/x3.asp?node=&sample=100&w10=coronavirus&w11=vaccination&r=)  [200](https://www.english-corpora.org/corona/x3.asp?node=&sample=200&w10=coronavirus&w11=vaccination&r=)  [500](https://www.english-corpora.org/corona/x3.asp?node=&sample=500&w10=coronavirus&w11=vaccination&r=)  [1000](https://www.english-corpora.org/corona/x3.asp?node=&sample=1000&w10=coronavirus&w11=vaccination&r=) PAGE:   [**<<**](https://www.english-corpora.org/corona/x3.asp?node=&p=1&w10=coronavirus&w11=vaccination&r=) [**<**](https://www.english-corpora.org/corona/x3.asp?node=&p=4&w10=coronavirus&w11=vaccination&r=)   5 / 42  [**>**](https://www.english-corpora.org/corona/x3.asp?node=&p=6&w10=coronavirus&w11=vaccination&r=) [**>>**](https://www.english-corpora.org/corona/x3.asp?node=&p=42&w10=coronavirus&w11=vaccination&r=) |  |
| --- | --- |

Начало формы

| CLICK FOR MORE CONTEXT | [**HELP**](javascript:newFeatures()) | [SAVE](javascript:chooser('s'))    [TRANSLATE](javascript:chooser('t'))    [ANALYZE](javascript:chooser('p')) |
| --- | --- | --- |

| [401](https://www.english-corpora.org/corona/x4.asp?rs=&t=73722215&ID=22407878535) | [21-11-24 US](https://www.english-corpora.org/corona/x4.asp?rs=&t=73722215&ID=22407878535) | [theweek.com](https://www.english-corpora.org/corona/x3.asp?node=&p=5&w10=coronavirus&w11=vaccination&r=) |  |  |  | -- who is Jewish, incidentally -- to whine about his proposal to mandate **coronavirus** **vaccination** to attend public school. # Even for American conservatives, whose victim complex runs |
| --- | --- | --- | --- | --- | --- | --- |
| [402](https://www.english-corpora.org/corona/x4.asp?rs=&t=73726043&ID=22418906353) | [21-11-25 US](https://www.english-corpora.org/corona/x4.asp?rs=&t=73726043&ID=22418906353) | [apnews.com](https://www.english-corpora.org/corona/x3.asp?node=&p=5&w10=coronavirus&w11=vaccination&r=) |  |  |  | . John Bel Edwards' administration to start requiring some students to get the **coronavirus** **vaccination** or submit a written dissent to attend school. # The Advocate reports the mandate |
| [403](https://www.english-corpora.org/corona/x4.asp?rs=&t=73736672&ID=22451946751) | [21-11-28 US](https://www.english-corpora.org/corona/x4.asp?rs=&t=73736672&ID=22451946751) | [nationalreview.com](https://www.english-corpora.org/corona/x3.asp?node=&p=5&w10=coronavirus&w11=vaccination&r=) |  |  |  | the BioNTech and Pfizer **coronavirus** **vaccination** is given in Mainz, Germany. (BioNTech SE 2020/Handout via Reuters) # |
| [404](https://www.english-corpora.org/corona/x4.asp?rs=&t=88328057&ID=22404556273) | [21-11-23 US](https://www.english-corpora.org/corona/x4.asp?rs=&t=88328057&ID=22404556273) | [MSN](https://www.english-corpora.org/corona/x3.asp?node=&p=5&w10=coronavirus&w11=vaccination&r=) |  |  |  | The Biden administration has asked a federal court to lift the stay on a **coronavirus** **vaccination** rule for large businesses. The rule, which was set to go into effect |
| [406](https://www.english-corpora.org/corona/x4.asp?rs=&t=88336226&ID=22421545619) | [21-11-25 US](https://www.english-corpora.org/corona/x4.asp?rs=&t=88336226&ID=22421545619) | [Houston Chronicle](https://www.english-corpora.org/corona/x3.asp?node=&p=5&w10=coronavirus&w11=vaccination&r=)[(1)](https://www.english-corpora.org/corona/duplicates1.asp?xx=405&n=1) |  |  |  | (AP) -- Trying to boost one of the nation's lowest **coronavirus** **vaccination** rates, Louisiana Gov. John Bel Edwards' administration has spent $27 million and |
| [407](https://www.english-corpora.org/corona/x4.asp?rs=&t=88340530&ID=22429926458) | [21-11-26 US](https://www.english-corpora.org/corona/x4.asp?rs=&t=88340530&ID=22429926458) | [YAHOO!News](https://www.english-corpora.org/corona/x3.asp?node=&p=5&w10=coronavirus&w11=vaccination&r=) |  |  |  | the Washington, D.C. region will soon face repercussions after failing to comply with **coronavirus** **vaccination** deadlines. While the vast majority of staffers at these schools are already vaccinated, |
| [409](https://www.english-corpora.org/corona/x4.asp?rs=&t=88356186&ID=22455944273) | [21-11-28 US](https://www.english-corpora.org/corona/x4.asp?rs=&t=88356186&ID=22455944273) | [WBUR](https://www.english-corpora.org/corona/x3.asp?node=&p=5&w10=coronavirus&w11=vaccination&r=)[(1)](https://www.english-corpora.org/corona/duplicates1.asp?xx=408&n=1) |  |  |  | will require employee vaccinations # The Essex County sheriff is requiring proof of **coronavirus** **vaccination** for all employees, vendors and contractors at the jail his office oversees. # |
| [411](https://www.english-corpora.org/corona/x4.asp?rs=&t=43684560&ID=22408139918) | [21-11-24 GB](https://www.english-corpora.org/corona/x4.asp?rs=&t=43684560&ID=22408139918) | [politico.eu](https://www.english-corpora.org/corona/x3.asp?node=&p=5&w10=coronavirus&w11=vaccination&r=)[(1)](https://www.english-corpora.org/corona/duplicates1.asp?xx=410&n=1) |  |  |  | reporters. # Scholz said the new government would ramp up the national **coronavirus** **vaccination** campaign and consider making shots compulsory for health staff and other essential workers. # |
| [412](https://www.english-corpora.org/corona/x4.asp?rs=&t=43692224&ID=22435259078) | [21-11-27 GB](https://www.english-corpora.org/corona/x4.asp?rs=&t=43692224&ID=22435259078) | [poundsterlinglive.com](https://www.english-corpora.org/corona/x3.asp?node=&p=5&w10=coronavirus&w11=vaccination&r=) |  |  |  | nature of this market. 43692229 Third **coronavirus** **vaccination** centre for Dover district in Whitfield # People in Dover district are being urged to |
| [413](https://www.english-corpora.org/corona/x4.asp?rs=&t=43700181&ID=22472054498) | [21-11-30 GB](https://www.english-corpora.org/corona/x4.asp?rs=&t=43700181&ID=22472054498) | [politico.eu](https://www.english-corpora.org/corona/x3.asp?node=&p=5&w10=coronavirus&w11=vaccination&r=) |  |  |  | Chancellor Olaf Scholz is in favor of introducing mandatory **coronavirus** **vaccination** for all Germans as early as February, an official close to Scholz said. |
| [414](https://www.english-corpora.org/corona/x4.asp?rs=&t=43700181&ID=22472054791) | [21-11-30 GB](https://www.english-corpora.org/corona/x4.asp?rs=&t=43700181&ID=22472054791) | [politico.eu](https://www.english-corpora.org/corona/x3.asp?node=&p=5&w10=coronavirus&w11=vaccination&r=) |  |  |  | next winter. " # Austria was the first Western country to announce mandatory **coronavirus** **vaccination**, which is planned to enter into force in February. |
| [415](https://www.english-corpora.org/corona/x4.asp?rs=&t=43701224&ID=22468489716) | [21-11-30 GB](https://www.english-corpora.org/corona/x4.asp?rs=&t=43701224&ID=22468489716) | [plymouthherald.co.uk](https://www.english-corpora.org/corona/x3.asp?node=&p=5&w10=coronavirus&w11=vaccination&r=) |  |  |  | understanding. You can unsubscribe at any time. More info # The Covid-19 **coronavirus** **vaccination** programme " is all about speed ", a member of the Joint Committee on |
| [416](https://www.english-corpora.org/corona/x4.asp?rs=&t=53377183&ID=22450678897) | [21-11-28 PK](https://www.english-corpora.org/corona/x4.asp?rs=&t=53377183&ID=22450678897) | [nation.com.pk](https://www.english-corpora.org/corona/x3.asp?node=&p=5&w10=coronavirus&w11=vaccination&r=) |  |  |  | compulsory to offer prayers in Sindh mosques # KARACHI - The Sindh government made **coronavirus** **vaccination** compulsory for people offering prayers in mosques, in a bid to keep the virus |
| [417](https://www.english-corpora.org/corona/x4.asp?rs=&t=33480562&ID=22480430867) | [21-12-01 US](https://www.english-corpora.org/corona/x4.asp?rs=&t=33480562&ID=22480430867) | [staradvertiser.com](https://www.english-corpora.org/corona/x3.asp?node=&p=5&w10=coronavirus&w11=vaccination&r=) |  |  |  | coronavirus **vaccination** for those over 60 years old # By Associated Press # Today # ASSOCIATED PRESS |
| [418](https://www.english-corpora.org/corona/x4.asp?rs=&t=33496139&ID=22549482944) | [21-12-07 US](https://www.english-corpora.org/corona/x4.asp?rs=&t=33496139&ID=22549482944) | [nytimes.com](https://www.english-corpora.org/corona/x3.asp?node=&p=5&w10=coronavirus&w11=vaccination&r=) |  |  |  | at the Center for American Progress. # At least 22 states now require **coronavirus** **vaccination** for some categories of workers, like those employed by the state or in health |
| [419](https://www.english-corpora.org/corona/x4.asp?rs=&t=33496170&ID=22552455841) | [21-12-07 US](https://www.english-corpora.org/corona/x4.asp?rs=&t=33496170&ID=22552455841) | [nytimes.com](https://www.english-corpora.org/corona/x3.asp?node=&p=5&w10=coronavirus&w11=vaccination&r=) |  |  |  | the State House chamber on Thursday because they did not show proof of a **coronavirus** **vaccination** or a negative Covid-19 test.Credit... Mike Catalini/Associated Press # The speaker of the New |
| [420](https://www.english-corpora.org/corona/x4.asp?rs=&t=33517158&ID=22614802744) | [21-12-13 US](https://www.english-corpora.org/corona/x4.asp?rs=&t=33517158&ID=22614802744) | [aljazeera.com](https://www.english-corpora.org/corona/x3.asp?node=&p=5&w10=coronavirus&w11=vaccination&r=) |  |  |  | people to get vaccinated against COVID-19. # DRC has the world's lowest **coronavirus** **vaccination** rate, with roughly 0.1 percent of its 90 million population inoculated. # " |
| [421](https://www.english-corpora.org/corona/x4.asp?rs=&t=33517344&ID=22614836283) | [21-12-13 US](https://www.english-corpora.org/corona/x4.asp?rs=&t=33517344&ID=22614836283) | [aljazeera.com](https://www.english-corpora.org/corona/x3.asp?node=&p=5&w10=coronavirus&w11=vaccination&r=) |  |  |  | was selected to host the contest earlier this year because of Israel's successful **coronavirus** **vaccination** programme. # South Africa's Lalela Mswane, from left, India's Harnaaz |
| [422](https://www.english-corpora.org/corona/x4.asp?rs=&t=33521524&ID=22637832250) | [21-12-15 US](https://www.english-corpora.org/corona/x4.asp?rs=&t=33521524&ID=22637832250) | [nytimes.com](https://www.english-corpora.org/corona/x3.asp?node=&p=5&w10=coronavirus&w11=vaccination&r=) |  |  |  | against the coronavirus, already requires proof of at least one dose of a **coronavirus** **vaccination** to eat indoors at a restaurant or see a movie. But now masks will |
| [423](https://www.english-corpora.org/corona/x4.asp?rs=&t=33532077&ID=22679255467) | [21-12-18 US](https://www.english-corpora.org/corona/x4.asp?rs=&t=33532077&ID=22679255467) | [chicago.suntimes.com](https://www.english-corpora.org/corona/x3.asp?node=&p=5&w10=coronavirus&w11=vaccination&r=) |  |  |  | and Safety Administration said Saturday that it would not issue citations tied to its **coronavirus** **vaccination** mandate before Jan. 10, so that companies have time to adjust to and implement |
| [427](https://www.english-corpora.org/corona/x4.asp?rs=&t=33536524&ID=22693593087) | [21-12-20 US](https://www.english-corpora.org/corona/x4.asp?rs=&t=33536524&ID=22693593087) | [nytimes.com](https://www.english-corpora.org/corona/x3.asp?node=&p=5&w10=coronavirus&w11=vaccination&r=)[(3)](https://www.english-corpora.org/corona/duplicates1.asp?xx=426&n=3) |  |  |  | Minister Boris Johnson of Britain during a visit to a National Health Service **coronavirus** **vaccination** center near Ramsgate, England, on Thursday.Credit... Pool photo by Leon Neal # |
| [428](https://www.english-corpora.org/corona/x4.asp?rs=&t=33537793&ID=22699137418) | [21-12-20 US](https://www.english-corpora.org/corona/x4.asp?rs=&t=33537793&ID=22699137418) | [pilotonline.com](https://www.english-corpora.org/corona/x3.asp?node=&p=5&w10=coronavirus&w11=vaccination&r=) |  |  |  | and Safety Administration said Saturday that it would not issue citations tied to its **coronavirus** **vaccination** mandate before Jan. 10, so that companies have time to adjust to and implement |
| [429](https://www.english-corpora.org/corona/x4.asp?rs=&t=43735883&ID=22612535560) | [21-12-13 US](https://www.english-corpora.org/corona/x4.asp?rs=&t=43735883&ID=22612535560) | [ktar.com](https://www.english-corpora.org/corona/x3.asp?node=&p=5&w10=coronavirus&w11=vaccination&r=) |  |  |  | was selected to host the contest earlier this year because of Israel's successful **coronavirus** **vaccination** program. # The contest suffered a last-minute hiccup with the arrival of the omicron |
| [430](https://www.english-corpora.org/corona/x4.asp?rs=&t=43742239&ID=22640971810) | [21-12-15 US](https://www.english-corpora.org/corona/x4.asp?rs=&t=43742239&ID=22640971810) | [ktar.com](https://www.english-corpora.org/corona/x3.asp?node=&p=5&w10=coronavirus&w11=vaccination&r=) |  |  |  | vaccination of migrants in Yemen # CAIRO (AP) -- The United Nations has launched |
| [431](https://www.english-corpora.org/corona/x4.asp?rs=&t=43742239&ID=22640971828) | [21-12-15 US](https://www.english-corpora.org/corona/x4.asp?rs=&t=43742239&ID=22640971828) | [ktar.com](https://www.english-corpora.org/corona/x3.asp?node=&p=5&w10=coronavirus&w11=vaccination&r=) |  |  |  | in Yemen # CAIRO (AP) -- The United Nations has launched a **coronavirus** **vaccination** campaign for stranded migrants in war-torn Yemen, the U.N. migration agency said Wednesday |
| [432](https://www.english-corpora.org/corona/x4.asp?rs=&t=43752509&ID=22675833085) | [21-12-18 US](https://www.english-corpora.org/corona/x4.asp?rs=&t=43752509&ID=22675833085) | [news4jax.com](https://www.english-corpora.org/corona/x3.asp?node=&p=5&w10=coronavirus&w11=vaccination&r=) |  |  |  | and Safety Administration said Saturday that it would not issue citations tied to its **coronavirus** **vaccination** mandate before Jan. 10, so that companies have time to adjust to and implement |
| [436](https://www.english-corpora.org/corona/x4.asp?rs=&t=63626328&ID=22548631378) | [21-12-07 US](https://www.english-corpora.org/corona/x4.asp?rs=&t=63626328&ID=22548631378) | [nwitimes.com](https://www.english-corpora.org/corona/x3.asp?node=&p=5&w10=coronavirus&w11=vaccination&r=)[(3)](https://www.english-corpora.org/corona/duplicates1.asp?xx=435&n=3) |  |  |  | vaccine at Community Hospital in Munster. Nurse Barbara Pennington administers the first **coronavirus** **vaccination** in Lake County. # Provided by Community Hospital Region's first COVID vaccine |
| [437](https://www.english-corpora.org/corona/x4.asp?rs=&t=63669503&ID=22706886607) | [21-12-21 US](https://www.english-corpora.org/corona/x4.asp?rs=&t=63669503&ID=22706886607) | [mdjonline.com](https://www.english-corpora.org/corona/x3.asp?node=&p=5&w10=coronavirus&w11=vaccination&r=) |  |  |  | COVID Study -- an app some U.K. residents use to self-report information about their **coronavirus** **vaccination** and infections. # A comparison of data from thousands of people who tested positive |
| [438](https://www.english-corpora.org/corona/x4.asp?rs=&t=63670359&ID=22708257284) | [21-12-21 US](https://www.english-corpora.org/corona/x4.asp?rs=&t=63670359&ID=22708257284) | [whsv.com](https://www.english-corpora.org/corona/x3.asp?node=&p=5&w10=coronavirus&w11=vaccination&r=) |  |  |  | . (WVIR) - The University of Virginia is updating its **coronavirus** **vaccination** policies for the spring semester. # " After careful consideration of current and projected |
| [439](https://www.english-corpora.org/corona/x4.asp?rs=&t=73746164&ID=22482646912) | [21-12-01 US](https://www.english-corpora.org/corona/x4.asp?rs=&t=73746164&ID=22482646912) | [nationalreview.com](https://www.english-corpora.org/corona/x3.asp?node=&p=5&w10=coronavirus&w11=vaccination&r=) |  |  |  | # Dr. Anthony Fauci stands by during an event to commemorate the 50 millionth **coronavirus** **vaccination** in the South Court Auditorium at the White House, February 25, 2021. |
| [440](https://www.english-corpora.org/corona/x4.asp?rs=&t=73788805&ID=22614128755) | [21-12-13 US](https://www.english-corpora.org/corona/x4.asp?rs=&t=73788805&ID=22614128755) | [pe.com](https://www.english-corpora.org/corona/x3.asp?node=&p=5&w10=coronavirus&w11=vaccination&r=) |  |  |  | for vaccinations. Across California, 71.5% of residents have had at least one **coronavirus** **vaccination**. In Riverside County, 60.5% of residents have had at least one shot. |
| [442](https://www.english-corpora.org/corona/x4.asp?rs=&t=73805164&ID=22655546357) | [21-12-16 US](https://www.english-corpora.org/corona/x4.asp?rs=&t=73805164&ID=22655546357) | [adn.com](https://www.english-corpora.org/corona/x3.asp?node=&p=5&w10=coronavirus&w11=vaccination&r=)[(1)](https://www.english-corpora.org/corona/duplicates1.asp?xx=441&n=1) |  |  |  | Army medics in Los Angeles about the variety of syringes used for a **coronavirus** **vaccination**. (Pfc. Garrison Waites/U.S. Army.) # WASHINGTON -- The number |
| [443](https://www.english-corpora.org/corona/x4.asp?rs=&t=73818694&ID=22689462023) | [21-12-19 US](https://www.english-corpora.org/corona/x4.asp?rs=&t=73818694&ID=22689462023) | [wtae.com](https://www.english-corpora.org/corona/x3.asp?node=&p=5&w10=coronavirus&w11=vaccination&r=) |  |  |  | and Safety Administration said Saturday that it would not issue citations tied to its **coronavirus** **vaccination** mandate before Jan. 10, so that companies have time to adjust to and implement |
| [445](https://www.english-corpora.org/corona/x4.asp?rs=&t=73824057&ID=22708465073) | [21-12-21 US](https://www.english-corpora.org/corona/x4.asp?rs=&t=73824057&ID=22708465073) | [wbal.com](https://www.english-corpora.org/corona/x3.asp?node=&p=5&w10=coronavirus&w11=vaccination&r=)[(1)](https://www.english-corpora.org/corona/duplicates1.asp?xx=444&n=1) |  |  |  | Lori Lightfoot said Tuesday the nation's third-largest city will require proof of **coronavirus** **vaccination** at restaurants, bars, gyms and other indoor venues, as the rapidly spreading |
| [446](https://www.english-corpora.org/corona/x4.asp?rs=&t=88378949&ID=22502426027) | [21-12-02 US](https://www.english-corpora.org/corona/x4.asp?rs=&t=88378949&ID=22502426027) | [San Diego Union-Tribune](https://www.english-corpora.org/corona/x3.asp?node=&p=5&w10=coronavirus&w11=vaccination&r=) |  |  |  | doses together. # Look closely at what's happening in the very busy **coronavirus** **vaccination** clinic at Rady Children's Hospital in San Diego, and you can see true |
| [447](https://www.english-corpora.org/corona/x4.asp?rs=&t=88387649&ID=22524437346) | [21-12-04 US](https://www.english-corpora.org/corona/x4.asp?rs=&t=88387649&ID=22524437346) | [The National Interest](https://www.english-corpora.org/corona/x3.asp?node=&p=5&w10=coronavirus&w11=vaccination&r=) |  |  |  | the media's reporting of the Omicron's rapid spread across the world, **coronavirus** **vaccination** rates in the United States spiked on both Wednesday and Thursday, with data from |
| [448](https://www.english-corpora.org/corona/x4.asp?rs=&t=88392575&ID=22532548556) | [21-12-05 US](https://www.english-corpora.org/corona/x4.asp?rs=&t=88392575&ID=22532548556) | [Patch](https://www.english-corpora.org/corona/x3.asp?node=&p=5&w10=coronavirus&w11=vaccination&r=) |  |  |  | with the Essex County Sheriff's Department will need to provide proof of a **coronavirus** **vaccination** as of Jan. 4, 2022, as part of a new order from Sheriff |
| [449](https://www.english-corpora.org/corona/x4.asp?rs=&t=88403332&ID=22555726279) | [21-12-07 US](https://www.english-corpora.org/corona/x4.asp?rs=&t=88403332&ID=22555726279) | [ABC](https://www.english-corpora.org/corona/x3.asp?node=&p=5&w10=coronavirus&w11=vaccination&r=) |  |  |  | 2 min read # The Associated Press # FILE - A woman receives a **coronavirus** **vaccination** at the Kololo airstrip in Kampala, Uganda on May 31, 2021. Uganda |
| [452](https://www.english-corpora.org/corona/x4.asp?rs=&t=88421662&ID=22592010216) | [21-12-10 US](https://www.english-corpora.org/corona/x4.asp?rs=&t=88421662&ID=22592010216) | [Fox Business](https://www.english-corpora.org/corona/x3.asp?node=&p=5&w10=coronavirus&w11=vaccination&r=)[(2)](https://www.english-corpora.org/corona/duplicates1.asp?xx=451&n=2) |  |  |  | adequate amount of employees to meet the Biden administration's Jan. 4 federal **coronavirus** **vaccination** deadline, necessitating service reductions, according to the Wall Street Journal. # He |
| [453](https://www.english-corpora.org/corona/x4.asp?rs=&t=88431534&ID=22602024627) | [21-12-11 US](https://www.english-corpora.org/corona/x4.asp?rs=&t=88431534&ID=22602024627) | [The Boston Globe on MSN.com](https://www.english-corpora.org/corona/x3.asp?node=&p=5&w10=coronavirus&w11=vaccination&r=) |  |  |  | , state health officials said Rhode Island is winding down operations at its large **coronavirus** **vaccination** sites in favor of more community-based vaccination clinics, the Associated Press reported. # |
| [454](https://www.english-corpora.org/corona/x4.asp?rs=&t=88450041&ID=22647154999) | [21-12-15 US](https://www.english-corpora.org/corona/x4.asp?rs=&t=88450041&ID=22647154999) | [ABC](https://www.english-corpora.org/corona/x3.asp?node=&p=5&w10=coronavirus&w11=vaccination&r=) |  |  |  | the same thing. 88450041 UN launches **coronavirus** **vaccination** of migrants in Yemen # The U.N. migration agency says it has launched a |
| [455](https://www.english-corpora.org/corona/x4.asp?rs=&t=88450041&ID=22647155016) | [21-12-15 US](https://www.english-corpora.org/corona/x4.asp?rs=&t=88450041&ID=22647155016) | [ABC](https://www.english-corpora.org/corona/x3.asp?node=&p=5&w10=coronavirus&w11=vaccination&r=) |  |  |  | migrants in Yemen # The U.N. migration agency says it has launched a **coronavirus** **vaccination** campaign for stranded migrants in war-torn Yemen # ByThe Associated Press # December 15, |
| [456](https://www.english-corpora.org/corona/x4.asp?rs=&t=88450041&ID=22647155051) | [21-12-15 US](https://www.english-corpora.org/corona/x4.asp?rs=&t=88450041&ID=22647155051) | [ABC](https://www.english-corpora.org/corona/x3.asp?node=&p=5&w10=coronavirus&w11=vaccination&r=) |  |  |  | # * 2 min read # CAIRO -- The United Nations has launched a **coronavirus** **vaccination** campaign for stranded migrants in war-torn Yemen, the U.N. migration agency said Wednesday |
| [457](https://www.english-corpora.org/corona/x4.asp?rs=&t=88455930&ID=22645635694) | [21-12-15 US](https://www.english-corpora.org/corona/x4.asp?rs=&t=88455930&ID=22645635694) | [NJ.com](https://www.english-corpora.org/corona/x3.asp?node=&p=5&w10=coronavirus&w11=vaccination&r=) |  |  |  | Phil Murphy visited the hospital to mark the one-year anniversary of New Jersey's **coronavirus** **vaccination** program -- and to call for more fully vaccinated people to receive booster shots as |
| [458](https://www.english-corpora.org/corona/x4.asp?rs=&t=88458129&ID=22659607163) | [21-12-16 US](https://www.english-corpora.org/corona/x4.asp?rs=&t=88458129&ID=22659607163) | [CNN](https://www.english-corpora.org/corona/x3.asp?node=&p=5&w10=coronavirus&w11=vaccination&r=) |  |  |  | of Republicans don't want a booster # A nurse marks a **coronavirus** **vaccination** card with a third " booster " dose of Pfizer, at a vaccine clinic |
| [459](https://www.english-corpora.org/corona/x4.asp?rs=&t=88468141&ID=22672364953) | [21-12-17 US](https://www.english-corpora.org/corona/x4.asp?rs=&t=88468141&ID=22672364953) | [The Washington Post on MSN.com](https://www.english-corpora.org/corona/x3.asp?node=&p=5&w10=coronavirus&w11=vaccination&r=) |  |  |  | . # The Nets will bring Irving back for road games in cities without **coronavirus** **vaccination** mandates. New York City's mandate will continue to keep Irving from playing at |
| [460](https://www.english-corpora.org/corona/x4.asp?rs=&t=88468141&ID=22672365172) | [21-12-17 US](https://www.english-corpora.org/corona/x4.asp?rs=&t=88468141&ID=22672365172) | [The Washington Post on MSN.com](https://www.english-corpora.org/corona/x3.asp?node=&p=5&w10=coronavirus&w11=vaccination&r=) |  |  |  | in October to get vaccinated, per the terms of New York City's **coronavirus** **vaccination** mandate. That month, he confirmed that he was unvaccinated and said he was |
| [461](https://www.english-corpora.org/corona/x4.asp?rs=&t=88469776&ID=22684328029) | [21-12-18 US](https://www.english-corpora.org/corona/x4.asp?rs=&t=88469776&ID=22684328029) | [YAHOO!Finance](https://www.english-corpora.org/corona/x3.asp?node=&p=5&w10=coronavirus&w11=vaccination&r=) |  |  |  | and Safety Administration said Saturday that it would not issue citations tied to its **coronavirus** **vaccination** mandate before Jan. 10, so that companies have time to adjust to and implement |
| [464](https://www.english-corpora.org/corona/x4.asp?rs=&t=88476070&ID=22692195886) | [21-12-19 US](https://www.english-corpora.org/corona/x4.asp?rs=&t=88476070&ID=22692195886) | [YAHOO!News](https://www.english-corpora.org/corona/x3.asp?node=&p=5&w10=coronavirus&w11=vaccination&r=)[(2)](https://www.english-corpora.org/corona/duplicates1.asp?xx=463&n=2) |  |  |  | Safety Administration said Saturday that it would not issue citations tied to its **coronavirus** **vaccination** mandate for large employers before Jan. 10, so that companies have time to adjust |
| [465](https://www.english-corpora.org/corona/x4.asp?rs=&t=88481346&ID=22703921996) | [21-12-20 US](https://www.english-corpora.org/corona/x4.asp?rs=&t=88481346&ID=22703921996) | [...ciated Press - Sports on MSN.com](https://www.english-corpora.org/corona/x3.asp?node=&p=5&w10=coronavirus&w11=vaccination&r=) |  |  |  | (AP) -- The University of New Mexico said Monday it will add **coronavirus** **vaccination** rules to a mask requirement for fans entering the Pit arena in Albuquerque starting after |
| [467](https://www.english-corpora.org/corona/x4.asp?rs=&t=88485624&ID=22710527805) | [21-12-21 US](https://www.english-corpora.org/corona/x4.asp?rs=&t=88485624&ID=22710527805) | [Yahoo! Sports](https://www.english-corpora.org/corona/x3.asp?node=&p=5&w10=coronavirus&w11=vaccination&r=)[(1)](https://www.english-corpora.org/corona/duplicates1.asp?xx=466&n=1) |  |  |  | Association of Foodbank. Recommended Stories # Chicago will require proof of **coronavirus** **vaccination** at restaurants, bars, gyms and other indoor venues, as the rapidly spreading |
| [468](https://www.english-corpora.org/corona/x4.asp?rs=&t=53399032&ID=22504532930) | [21-12-03 CA](https://www.english-corpora.org/corona/x4.asp?rs=&t=53399032&ID=22504532930) | [thestar.com](https://www.english-corpora.org/corona/x3.asp?node=&p=5&w10=coronavirus&w11=vaccination&r=) |  |  |  | kick in Wednesday. # Face masks are again required in enclosed spaces and **coronavirus** **vaccination** or COVID-19 recovery tests are required to enter restaurants, cinemas, gyms and hotels |
| [469](https://www.english-corpora.org/corona/x4.asp?rs=&t=53450674&ID=22700089086) | [21-12-20 CA](https://www.english-corpora.org/corona/x4.asp?rs=&t=53450674&ID=22700089086) | [kelownadailycourier.ca](https://www.english-corpora.org/corona/x3.asp?node=&p=5&w10=coronavirus&w11=vaccination&r=) |  |  |  | and many other indoor businesses in Boston will be required to show proof of **coronavirus** **vaccination** starting in mid-January in an effort to curb a rise in new cases across the |
| [470](https://www.english-corpora.org/corona/x4.asp?rs=&t=53451137&ID=22697534501) | [21-12-20 CA](https://www.english-corpora.org/corona/x4.asp?rs=&t=53451137&ID=22697534501) | [chroniclejournal.com](https://www.english-corpora.org/corona/x3.asp?node=&p=5&w10=coronavirus&w11=vaccination&r=) |  |  |  | and Safety Administration said Saturday that it would not issue citations tied to its **coronavirus** **vaccination** mandate before Jan. 10, so that companies have time to adjust to and implement |
| [471](https://www.english-corpora.org/corona/x4.asp?rs=&t=33485046&ID=22493856231) | [21-12-02 GB](https://www.english-corpora.org/corona/x4.asp?rs=&t=33485046&ID=22493856231) | [edition.cnn.com](https://www.english-corpora.org/corona/x3.asp?node=&p=5&w10=coronavirus&w11=vaccination&r=) |  |  |  | of Republicans don't want a booster # A nurse marks a **coronavirus** **vaccination** card with a third " booster " dose of Pfizer, at a vaccine clinic |
| [472](https://www.english-corpora.org/corona/x4.asp?rs=&t=43714735&ID=22550271679) | [21-12-07 GB](https://www.english-corpora.org/corona/x4.asp?rs=&t=43714735&ID=22550271679) | [dailyrecord.co.uk](https://www.english-corpora.org/corona/x3.asp?node=&p=5&w10=coronavirus&w11=vaccination&r=) |  |  |  | centre in Kilmarnock is being used as a **Coronavirus** **vaccination** centre (Image: Submitted/Kilmarnock Standard) # Covid-19 vaccinations are available on a drop-in |
| [473](https://www.english-corpora.org/corona/x4.asp?rs=&t=43716463&ID=22563073574) | [21-12-08 GB](https://www.english-corpora.org/corona/x4.asp?rs=&t=43716463&ID=22563073574) | [express.co.uk](https://www.english-corpora.org/corona/x3.asp?node=&p=5&w10=coronavirus&w11=vaccination&r=) |  |  |  | How to get a Covid pass # An NHS COVID Pass will show your **coronavirus** **vaccination** details or test results. This is also known as your COVID-19 status. # |
| [474](https://www.english-corpora.org/corona/x4.asp?rs=&t=43718727&ID=22560689078) | [21-12-08 GB](https://www.english-corpora.org/corona/x4.asp?rs=&t=43718727&ID=22560689078) | [theweek.co.uk](https://www.english-corpora.org/corona/x3.asp?node=&p=5&w10=coronavirus&w11=vaccination&r=) |  |  |  | Agency, making the UK the first country in the world to approve a **coronavirus** **vaccination.** 4 January 2021 # Johnson announces new national lockdown measures for England due to |
| [475](https://www.english-corpora.org/corona/x4.asp?rs=&t=43728532&ID=22595073017) | [21-12-11 GB](https://www.english-corpora.org/corona/x4.asp?rs=&t=43728532&ID=22595073017) | [thenational.scot](https://www.english-corpora.org/corona/x3.asp?node=&p=5&w10=coronavirus&w11=vaccination&r=) |  |  |  | to comment on how many of his squad have received both doses of the **coronavirus** **vaccination** when quizzed on the fact NHS Covid passes will be required for people to attend |
| [476](https://www.english-corpora.org/corona/x4.asp?rs=&t=43737904&ID=22625096382) | [21-12-14 GB](https://www.english-corpora.org/corona/x4.asp?rs=&t=43737904&ID=22625096382) | [bristolpost.co.uk](https://www.english-corpora.org/corona/x3.asp?node=&p=5&w10=coronavirus&w11=vaccination&r=) |  |  |  | nightclubs # We've outlined how you can get your hands on your third **coronavirus** **vaccination** and also when you'll be able to get it if you're not already |
| [477](https://www.english-corpora.org/corona/x4.asp?rs=&t=43740575&ID=22643253415) | [21-12-15 GB](https://www.english-corpora.org/corona/x4.asp?rs=&t=43740575&ID=22643253415) | [chroniclelive.co.uk](https://www.english-corpora.org/corona/x3.asp?node=&p=5&w10=coronavirus&w11=vaccination&r=) |  |  |  | : (a) a person who has received a complete course of the **coronavirus** **vaccination**, provided that the contact takes place more than 14 days after they have completed |
| [478](https://www.english-corpora.org/corona/x4.asp?rs=&t=43747874&ID=22668647458) | [21-12-17 GB](https://www.english-corpora.org/corona/x4.asp?rs=&t=43747874&ID=22668647458) | [politico.eu](https://www.english-corpora.org/corona/x3.asp?node=&p=5&w10=coronavirus&w11=vaccination&r=) |  |  |  | risks fueling distrust in political authority, a key driver of Romania's low **coronavirus** **vaccination** rate. It may boost the vaccine-skeptical far right, which has grown in strength |
| [479](https://www.english-corpora.org/corona/x4.asp?rs=&t=43751341&ID=22677556269) | [21-12-18 GB](https://www.english-corpora.org/corona/x4.asp?rs=&t=43751341&ID=22677556269) | [bristolpost.co.uk](https://www.english-corpora.org/corona/x3.asp?node=&p=5&w10=coronavirus&w11=vaccination&r=) |  |  |  | as last winter. # Reduce hospitalisations this winter are linked to the successful **coronavirus** **vaccination** programme which has now 89.4 per cent of people with the first jab, 81.6 |
| [480](https://www.english-corpora.org/corona/x4.asp?rs=&t=43755336&ID=22696781681) | [21-12-20 GB](https://www.english-corpora.org/corona/x4.asp?rs=&t=43755336&ID=22696781681) | [eadt.co.uk](https://www.english-corpora.org/corona/x3.asp?node=&p=5&w10=coronavirus&w11=vaccination&r=) |  |  |  | stop the Omicron variant. Pictured: Mr Javid during a visit to a **coronavirus** **vaccination** in London. - Credit: PA # The health secretary says the government will " |
| [481](https://www.english-corpora.org/corona/x4.asp?rs=&t=43761763&ID=22717183234) | [21-12-22 GB](https://www.english-corpora.org/corona/x4.asp?rs=&t=43761763&ID=22717183234) | [shropshirestar.com](https://www.english-corpora.org/corona/x3.asp?node=&p=5&w10=coronavirus&w11=vaccination&r=) |  |  |  | Subscribe to our daily newsletter! # Anna Garside, 28. receives her second **coronavirus** **vaccination** at the UK's first nightclub vaccine centre which has been set up at Birmingham |
| [482](https://www.english-corpora.org/corona/x4.asp?rs=&t=53398330&ID=22492103928) | [21-12-02 GB](https://www.english-corpora.org/corona/x4.asp?rs=&t=53398330&ID=22492103928) | [thescottishsun.co.uk](https://www.english-corpora.org/corona/x3.asp?node=&p=5&w10=coronavirus&w11=vaccination&r=) |  |  |  | of the Joint Committee on Vaccination and Immunisation (JCVI), said the **coronavirus** **vaccination** programme " is all about speed ". # He told BBC Breakfast: " |
| [483](https://www.english-corpora.org/corona/x4.asp?rs=&t=63626267&ID=22554568395) | [21-12-07 GB](https://www.english-corpora.org/corona/x4.asp?rs=&t=63626267&ID=22554568395) | [thewestmorlandgazette.co.uk](https://www.english-corpora.org/corona/x3.asp?node=&p=5&w10=coronavirus&w11=vaccination&r=) |  |  |  | 11% pf the population -- across the UK are yet to have a single **coronavirus** **vaccination**. # Nurse Lady T has urged people to come forward and get their Covid-19 |
| [484](https://www.english-corpora.org/corona/x4.asp?rs=&t=73808087&ID=22670235483) | [21-12-17 GB](https://www.english-corpora.org/corona/x4.asp?rs=&t=73808087&ID=22670235483) | [echo-news.co.uk](https://www.english-corpora.org/corona/x3.asp?node=&p=5&w10=coronavirus&w11=vaccination&r=) |  |  |  | from moral point of view' # Liverpool manager Jurgen Klopp believes getting a **coronavirus** **vaccination** should be mandatory " from a moral point of view ". # While the |
| [485](https://www.english-corpora.org/corona/x4.asp?rs=&t=63619752&ID=22537553396) | [21-12-06 IE](https://www.english-corpora.org/corona/x4.asp?rs=&t=63619752&ID=22537553396) | [intellinews.com](https://www.english-corpora.org/corona/x3.asp?node=&p=5&w10=coronavirus&w11=vaccination&r=) |  |  |  | more uncertain than usual, the Fund acknowledged, urging Georgia to increase its **coronavirus** **vaccination** rate. # Georgian health officials are alarmed and concerned by the recent low vaccination |
| [486](https://www.english-corpora.org/corona/x4.asp?rs=&t=63627138&ID=22564253166) | [21-12-08 IE](https://www.english-corpora.org/corona/x4.asp?rs=&t=63627138&ID=22564253166) | [the42.ie](https://www.english-corpora.org/corona/x3.asp?node=&p=5&w10=coronavirus&w11=vaccination&r=) |  |  |  | League match at Anfield, Liverpool. # Image: PA # PROOF OF **CORONAVIRUS** **vaccination** or a negative lateral flow test will be required for spectators to attend sporting events |
| [487](https://www.english-corpora.org/corona/x4.asp?rs=&t=73829223&ID=22716647425) | [21-12-22 AU](https://www.english-corpora.org/corona/x4.asp?rs=&t=73829223&ID=22716647425) | [watoday.com.au](https://www.english-corpora.org/corona/x3.asp?node=&p=5&w10=coronavirus&w11=vaccination&r=) |  |  |  | By Michaela Whitbourn # NSW Premier Dominic Perrottet says his state will push for **coronavirus** **vaccination** booster shots to be brought forward from five months to four months after a second |
| [488](https://www.english-corpora.org/corona/x4.asp?rs=&t=73769721&ID=22562571482) | [21-12-08 IN](https://www.english-corpora.org/corona/x4.asp?rs=&t=73769721&ID=22562571482) | [scroll.in](https://www.english-corpora.org/corona/x3.asp?node=&p=5&w10=coronavirus&w11=vaccination&r=) |  |  |  | made on the facade of the Tambaram railway station, to create awareness about **coronavirus** **vaccination** in Chennai. Arun Sankar / AFP # The Centre told the states on Wednesday |
| [489](https://www.english-corpora.org/corona/x4.asp?rs=&t=73806826&ID=22665325367) | [21-12-17 IN](https://www.english-corpora.org/corona/x4.asp?rs=&t=73806826&ID=22665325367) | [devdiscourse.com](https://www.english-corpora.org/corona/x3.asp?node=&p=5&w10=coronavirus&w11=vaccination&r=) |  |  |  | mandate for U.S. employees Boeing Co suspended its **coronavirus** **vaccination** requirement for U.S.-based employees, the U.S. planemaker said on Friday, capping weeks of |
| [491](https://www.english-corpora.org/corona/x4.asp?rs=&t=73823256&ID=22708352742) | [21-12-21 IN](https://www.english-corpora.org/corona/x4.asp?rs=&t=73823256&ID=22708352742) | [timesnownews.com](https://www.english-corpora.org/corona/x3.asp?node=&p=5&w10=coronavirus&w11=vaccination&r=)[(1)](https://www.english-corpora.org/corona/duplicates1.asp?xx=490&n=1) |  |  |  | called the country's Pandemic Expert Committee's recommendation to administer a fourth **coronavirus** **vaccination** to Israelis over 60 and medical teams " wonderful news ". # " ( |
| [492](https://www.english-corpora.org/corona/x4.asp?rs=&t=53396419&ID=22491674837) | [21-12-02 PK](https://www.english-corpora.org/corona/x4.asp?rs=&t=53396419&ID=22491674837) | [suchtv.pk](https://www.english-corpora.org/corona/x3.asp?node=&p=5&w10=coronavirus&w11=vaccination&r=) |  |  |  | NCOC) urged provinces in a morning session on Thursday to speed up the **coronavirus** **vaccination** process in light of the rising trend of the Omicron variant around the world and |
| [493](https://www.english-corpora.org/corona/x4.asp?rs=&t=53422419&ID=22573881496) | [21-12-09 PK](https://www.english-corpora.org/corona/x4.asp?rs=&t=53422419&ID=22573881496) | [thenews.com.pk](https://www.english-corpora.org/corona/x3.asp?node=&p=5&w10=coronavirus&w11=vaccination&r=) |  |  |  | that so far 16,175,000 people in Sindh had received the first dose of the **coronavirus** **vaccination** that accounted for 46 per cent portion of the population of Sindh. Some, |
| [495](https://www.english-corpora.org/corona/x4.asp?rs=&t=33518347&ID=22614945623) | [21-12-13 SG](https://www.english-corpora.org/corona/x4.asp?rs=&t=33518347&ID=22614945623) | [fourfourtwo.com](https://www.english-corpora.org/corona/x3.asp?node=&p=5&w10=coronavirus&w11=vaccination&r=)[(1)](https://www.english-corpora.org/corona/duplicates1.asp?xx=494&n=1) |  |  |  | , has urged his players and staff to make sure they get their **coronavirus** **vaccination** booster as soon as they become eligible. # Covid-19 restrictions have been tightened in |
| [496](https://www.english-corpora.org/corona/x4.asp?rs=&t=43738250&ID=22627648312) | [21-12-14 HK](https://www.english-corpora.org/corona/x4.asp?rs=&t=43738250&ID=22627648312) | [hongkongfp.com](https://www.english-corpora.org/corona/x3.asp?node=&p=5&w10=coronavirus&w11=vaccination&r=) |  |  |  | Hong Kong but authorities " can not let down their guard. " # **Coronavirus** **vaccination** in Hong Kong. Photo: GovHK. # In an interview with RTHK on |
| [497](https://www.english-corpora.org/corona/x4.asp?rs=&t=43751543&ID=22677577513) | [21-12-18 HK](https://www.english-corpora.org/corona/x4.asp?rs=&t=43751543&ID=22677577513) | [hongkongfp.com](https://www.english-corpora.org/corona/x3.asp?node=&p=5&w10=coronavirus&w11=vaccination&r=) |  |  |  | microbiology researchers at the University of Hong Kong and published on Tuesday. # **Coronavirus** **vaccination** in Hong Kong. Photo: GovHK. # Between the two vaccines, BioNTech |
| [498](https://www.english-corpora.org/corona/x4.asp?rs=&t=63638946&ID=22597545976) | [21-12-11 ZA](https://www.english-corpora.org/corona/x4.asp?rs=&t=63638946&ID=22597545976) | [heraldlive.co.za](https://www.english-corpora.org/corona/x3.asp?node=&p=5&w10=coronavirus&w11=vaccination&r=) |  |  |  | , mandated in July that its adult population be inoculated. # TAJIKISTAN made **coronavirus** **vaccination** mandatory for citizens above 18 years in July. # TURKMENISTAN has made vaccination mandatory |
| [499](https://www.english-corpora.org/corona/x4.asp?rs=&t=53389947&ID=22481584666) | [21-12-01 NG](https://www.english-corpora.org/corona/x4.asp?rs=&t=53389947&ID=22481584666) | [independent.ng](https://www.english-corpora.org/corona/x3.asp?node=&p=5&w10=coronavirus&w11=vaccination&r=) |  |  |  | Southern African coun? tries. # Much as we agree that evading the **Coronavirus** **vaccination** may be putting other people at risk and spreading the virus, compelling people to |
| [500](https://www.english-corpora.org/corona/x4.asp?rs=&t=53432068&ID=22618545392) | [21-12-13 NG](https://www.english-corpora.org/corona/x4.asp?rs=&t=53432068&ID=22618545392) | [pmnewsnigeria.com](https://www.english-corpora.org/corona/x3.asp?node=&p=5&w10=coronavirus&w11=vaccination&r=) |  |  |  | , Israel was chosen to host the pageant because of the country's successful **coronavirus** **vaccination** program. # The emergence of the omicron variant forced Israel to close its borders |

| PAGE:   [**<<**](https://www.english-corpora.org/corona/x3.asp?node=&p=1&w10=coronavirus&w11=vaccination&r=) [**<**](https://www.english-corpora.org/corona/x3.asp?node=&p=4&w10=coronavirus&w11=vaccination&r=)   5 / 42  [**>**](https://www.english-corpora.org/corona/x3.asp?node=&p=6&w10=coronavirus&w11=vaccination&r=) [**>>**](https://www.english-corpora.org/corona/x3.asp?node=&p=42&w10=coronavirus&w11=vaccination&r=) |
| --- |

Конец формы

| FIND SAMPLE:  [100](https://www.english-corpora.org/corona/x3.asp?node=&sample=100&w10=coronavirus&w11=vaccination&r=)  [200](https://www.english-corpora.org/corona/x3.asp?node=&sample=200&w10=coronavirus&w11=vaccination&r=)  [500](https://www.english-corpora.org/corona/x3.asp?node=&sample=500&w10=coronavirus&w11=vaccination&r=)  [1000](https://www.english-corpora.org/corona/x3.asp?node=&sample=1000&w10=coronavirus&w11=vaccination&r=) PAGE:   [**<<**](https://www.english-corpora.org/corona/x3.asp?node=&p=1&w10=coronavirus&w11=vaccination&r=) [**<**](https://www.english-corpora.org/corona/x3.asp?node=&p=5&w10=coronavirus&w11=vaccination&r=)   6 / 42  [**>**](https://www.english-corpora.org/corona/x3.asp?node=&p=7&w10=coronavirus&w11=vaccination&r=) [**>>**](https://www.english-corpora.org/corona/x3.asp?node=&p=42&w10=coronavirus&w11=vaccination&r=) |  |
| --- | --- |

Начало формы

| CLICK FOR MORE CONTEXT | [**HELP**](javascript:newFeatures()) | [SAVE](javascript:chooser('s'))    [TRANSLATE](javascript:chooser('t'))    [ANALYZE](javascript:chooser('p')) |
| --- | --- | --- |

| [501](https://www.english-corpora.org/corona/x4.asp?rs=&t=88289519&ID=22330508001) | [21-11-17 US](https://www.english-corpora.org/corona/x4.asp?rs=&t=88289519&ID=22330508001) | [Yahoo! Sports](https://www.english-corpora.org/corona/x3.asp?node=&p=6&w10=coronavirus&w11=vaccination&r=) |  |  |  | it will happen. " Recommended Stories # The Department of Correction's **coronavirus** **vaccination** rate hasn't improved in more than two weeks -- with nearly half of the |
| --- | --- | --- | --- | --- | --- | --- |
| [502](https://www.english-corpora.org/corona/x4.asp?rs=&t=73691112&ID=22324338452) | [21-11-17 PH](https://www.english-corpora.org/corona/x4.asp?rs=&t=73691112&ID=22324338452) | [bworldonline.com](https://www.english-corpora.org/corona/x3.asp?node=&p=6&w10=coronavirus&w11=vaccination&r=) |  |  |  | for the general population. The Philippines has been prioritizing health workers in its **coronavirus** **vaccination** program. # Health authorities earlier said seniors may also receive booster shots before the |
| [503](https://www.english-corpora.org/corona/x4.asp?rs=&t=43660635&ID=22324839692) | [21-11-17 HK](https://www.english-corpora.org/corona/x4.asp?rs=&t=43660635&ID=22324839692) | [hongkongfp.com](https://www.english-corpora.org/corona/x3.asp?node=&p=6&w10=coronavirus&w11=vaccination&r=) |  |  |  | year as Hong Kong was struggling to curb the spread of Covid-19. # **Coronavirus** **vaccination** in Hong Kong. Photo: GovHK. # As of Tuesday, Hong Kong |
| [504](https://www.english-corpora.org/corona/x4.asp?rs=&t=88184490&ID=22130119520) | [21-10-29 US](https://www.english-corpora.org/corona/x4.asp?rs=&t=88184490&ID=22130119520) | [Deadline.com](https://www.english-corpora.org/corona/x3.asp?node=&p=6&w10=coronavirus&w11=vaccination&r=) |  |  |  | of Californians and 3.5 million residents ages 5-11 might become eligible for their first **coronavirus** **vaccination** as soon as next week. The hope is that the additional vaccinations among those |
| [505](https://www.english-corpora.org/corona/x4.asp?rs=&t=88185321&ID=22127911962) | [21-10-29 US](https://www.english-corpora.org/corona/x4.asp?rs=&t=88185321&ID=22127911962) | [Black Enterprise](https://www.english-corpora.org/corona/x3.asp?node=&p=6&w10=coronavirus&w11=vaccination&r=) |  |  |  | ESPN, Irving spoke out about the consequences associated with him not getting a **coronavirus** **vaccination** and how it's about his life. # " The financial consequences, I |
| [506](https://www.english-corpora.org/corona/x4.asp?rs=&t=88187132&ID=22128291036) | [21-10-29 US](https://www.english-corpora.org/corona/x4.asp?rs=&t=88187132&ID=22128291036) | [Al Jazeera on MSN.com](https://www.english-corpora.org/corona/x3.asp?node=&p=6&w10=coronavirus&w11=vaccination&r=) |  |  |  | " said Patrick. The jab divide # Addressing the growing gap in **coronavirus** **vaccination** rates between rich and poor countries is also exacerbating inequalities between nations. # On |
| [507](https://www.english-corpora.org/corona/x4.asp?rs=&t=88187476&ID=22130836267) | [21-10-29 US](https://www.english-corpora.org/corona/x4.asp?rs=&t=88187476&ID=22130836267) | [The Telegraph](https://www.english-corpora.org/corona/x3.asp?node=&p=6&w10=coronavirus&w11=vaccination&r=) |  |  |  | # The lending agency called Thursday for greater efforts from wealthy nations to boost **coronavirus** **vaccination** rates in poorer countries, while also urging the Federal Reserve and other central banks |
| [508](https://www.english-corpora.org/corona/x4.asp?rs=&t=88193413&ID=22143026338) | [21-10-31 US](https://www.english-corpora.org/corona/x4.asp?rs=&t=88193413&ID=22143026338) | [Deadline.com](https://www.english-corpora.org/corona/x3.asp?node=&p=6&w10=coronavirus&w11=vaccination&r=) |  |  |  | of Californians and 3.5 million residents ages 5-11 might become eligible for their first **coronavirus** **vaccination** as soon as next week. The hope is that the additional vaccinations among those |
| [509](https://www.english-corpora.org/corona/x4.asp?rs=&t=73615937&ID=22124071341) | [21-10-29 AU](https://www.english-corpora.org/corona/x4.asp?rs=&t=73615937&ID=22124071341) | [perthnow.com.au](https://www.english-corpora.org/corona/x3.asp?node=&p=6&w10=coronavirus&w11=vaccination&r=) |  |  |  | trying to firebomb a police station amid growing volatility over Western Australia's mandatory **coronavirus** **vaccination** plans. # * Premier Mark McGowan is staying tight-lipped about Western Australia's border |
| [510](https://www.english-corpora.org/corona/x4.asp?rs=&t=73622080&ID=22142343700) | [21-10-31 IN](https://www.english-corpora.org/corona/x4.asp?rs=&t=73622080&ID=22142343700) | [cnbctv18.com](https://www.english-corpora.org/corona/x3.asp?node=&p=6&w10=coronavirus&w11=vaccination&r=) |  |  |  | Uddhav Thackeray on Sunday expressed concern over what said was a slow pace of **coronavirus** **vaccination** in the state, adding he will take up the issue during an interaction with |
| [511](https://www.english-corpora.org/corona/x4.asp?rs=&t=73622080&ID=22142343970) | [21-10-31 IN](https://www.english-corpora.org/corona/x4.asp?rs=&t=73622080&ID=22142343970) | [cnbctv18.com](https://www.english-corpora.org/corona/x3.asp?node=&p=6&w10=coronavirus&w11=vaccination&r=) |  |  |  | video conferencing on November 3 with district magistrates of over 40 districts having low **coronavirus** **vaccination** coverage. The meeting will include districts with less than 50 per cent coverage of |
| [512](https://www.english-corpora.org/corona/x4.asp?rs=&t=33394587&ID=22186474840) | [21-11-04 US](https://www.english-corpora.org/corona/x4.asp?rs=&t=33394587&ID=22186474840) | [nytimes.com](https://www.english-corpora.org/corona/x3.asp?node=&p=6&w10=coronavirus&w11=vaccination&r=) |  |  |  | those guys. " Advertisement # U.S. Air Force troops helping run a **coronavirus** **vaccination** site in Houston in February. The vaccine deadline for that branch of service arrived |
| [513](https://www.english-corpora.org/corona/x4.asp?rs=&t=33394587&ID=22186476768) | [21-11-04 US](https://www.english-corpora.org/corona/x4.asp?rs=&t=33394587&ID=22186476768) | [nytimes.com](https://www.english-corpora.org/corona/x3.asp?node=&p=6&w10=coronavirus&w11=vaccination&r=) |  |  |  | education committee. # The first child under 12 years old to receive a **coronavirus** **vaccination** at Texas Children's Hospital was Paxton Bowers, 5, a leukemia patient at |
| [514](https://www.english-corpora.org/corona/x4.asp?rs=&t=33397592&ID=22208312897) | [21-11-06 US](https://www.english-corpora.org/corona/x4.asp?rs=&t=33397592&ID=22208312897) | [inquisitr.com](https://www.english-corpora.org/corona/x3.asp?node=&p=6&w10=coronavirus&w11=vaccination&r=) |  |  |  | that some of these cuts, which included main roster superstars are due to **Coronavirus** **vaccination** statuses. Also referenced were the usual " budget cuts " the company has made |
| [515](https://www.english-corpora.org/corona/x4.asp?rs=&t=33408720&ID=22236141956) | [21-11-09 US](https://www.english-corpora.org/corona/x4.asp?rs=&t=33408720&ID=22236141956) | [jpost.com](https://www.english-corpora.org/corona/x3.asp?node=&p=6&w10=coronavirus&w11=vaccination&r=) |  |  |  | # The damage caused to the heart muscle in rare occurrences of myocarditis after **coronavirus** **vaccination** is mild and unlikely to affect patients' long-term health, according to a new |
| [516](https://www.english-corpora.org/corona/x4.asp?rs=&t=33419290&ID=22278876232) | [21-11-12 US](https://www.english-corpora.org/corona/x4.asp?rs=&t=33419290&ID=22278876232) | [yahoo.com](https://www.english-corpora.org/corona/x3.asp?node=&p=6&w10=coronavirus&w11=vaccination&r=) |  |  |  | increase their dividend each year and have a payout ratio below 50%. # **Coronavirus** **vaccination** clinics for Virginia Beach city employees were canceled through the rest of the year due |
| [517](https://www.english-corpora.org/corona/x4.asp?rs=&t=33420863&ID=22279042459) | [21-11-12 US](https://www.english-corpora.org/corona/x4.asp?rs=&t=33420863&ID=22279042459) | [news.yahoo.com](https://www.english-corpora.org/corona/x3.asp?node=&p=6&w10=coronavirus&w11=vaccination&r=) |  |  |  | Coronavirus **vaccination** clinics for Virginia Beach city employees were canceled through the rest of the year due |
| [518](https://www.english-corpora.org/corona/x4.asp?rs=&t=33423416&ID=22297618166) | [21-11-14 US](https://www.english-corpora.org/corona/x4.asp?rs=&t=33423416&ID=22297618166) | [nbcnews.com](https://www.english-corpora.org/corona/x3.asp?node=&p=6&w10=coronavirus&w11=vaccination&r=) |  |  |  | now littered with users caving to societal pressure or work mandates and receiving a **coronavirus** **vaccination**. # " Once you're injected, the lifesaving vaccination process has already begun |
| [519](https://www.english-corpora.org/corona/x4.asp?rs=&t=33454620&ID=22380603192) | [21-11-22 US](https://www.english-corpora.org/corona/x4.asp?rs=&t=33454620&ID=22380603192) | [ktla.com](https://www.english-corpora.org/corona/x3.asp?node=&p=6&w10=coronavirus&w11=vaccination&r=) |  |  |  | another winter spike that could overwhelm hospitals in some areas. # Visiting a **coronavirus** **vaccination** clinic in San Francisco, Newsom urged people to wear masks and get COVID shots |
| [520](https://www.english-corpora.org/corona/x4.asp?rs=&t=33454795&ID=22386321197) | [21-11-22 US](https://www.english-corpora.org/corona/x4.asp?rs=&t=33454795&ID=22386321197) | [nytimes.com](https://www.english-corpora.org/corona/x3.asp?node=&p=6&w10=coronavirus&w11=vaccination&r=) |  |  |  | Press # NAIROBI, Kenya -- Kenya will require people to show proof of **coronavirus** **vaccination** to enter many businesses, restaurants and government offices starting next month, a major |
| [521](https://www.english-corpora.org/corona/x4.asp?rs=&t=43599012&ID=22147101826) | [21-11-01 US](https://www.english-corpora.org/corona/x4.asp?rs=&t=43599012&ID=22147101826) | [wsbtv.com](https://www.english-corpora.org/corona/x3.asp?node=&p=6&w10=coronavirus&w11=vaccination&r=) |  |  |  | has thrown out California's new 30-foot buffer zone designed to restrict protests at **coronavirus** **vaccination** sites, though his ruling left in place other parts of a new state law |
| [522](https://www.english-corpora.org/corona/x4.asp?rs=&t=43647045&ID=22290998962) | [21-11-13 US](https://www.english-corpora.org/corona/x4.asp?rs=&t=43647045&ID=22290998962) | [fox6now.com](https://www.english-corpora.org/corona/x3.asp?node=&p=6&w10=coronavirus&w11=vaccination&r=) |  |  |  | # Meanwhile, the Madison diocese has instructed its 102 parishes not to host **coronavirus** **vaccination** clinics in their schools and churches. The diocese directive follows federal action which recently |
| [523](https://www.english-corpora.org/corona/x4.asp?rs=&t=53294292&ID=22176635996) | [21-11-03 US](https://www.english-corpora.org/corona/x4.asp?rs=&t=53294292&ID=22176635996) | [ksby.com](https://www.english-corpora.org/corona/x3.asp?node=&p=6&w10=coronavirus&w11=vaccination&r=) |  |  |  | has thrown out California's new 30-foot buffer zone designed to restrict protests at **coronavirus** **vaccination** sites. But U.S. District Judge Dale Drozd's ruling Saturday left in place other |
| [524](https://www.english-corpora.org/corona/x4.asp?rs=&t=73637989&ID=22187664795) | [21-11-04 US](https://www.english-corpora.org/corona/x4.asp?rs=&t=73637989&ID=22187664795) | [news.trust.org](https://www.english-corpora.org/corona/x3.asp?node=&p=6&w10=coronavirus&w11=vaccination&r=) |  |  |  | city workers under the mandate are vaccinated. # De Blasio had declared his **coronavirus** **vaccination** order for emergency responders a success on Monday, with no disruption to city services |
| [525](https://www.english-corpora.org/corona/x4.asp?rs=&t=88201200&ID=22153528453) | [21-11-01 US](https://www.english-corpora.org/corona/x4.asp?rs=&t=88201200&ID=22153528453) | [New York Daily News](https://www.english-corpora.org/corona/x3.asp?node=&p=6&w10=coronavirus&w11=vaccination&r=) |  |  |  | gear, march over the Brooklyn Bridge roadway to protest Mayor de Blasio's **coronavirus** **vaccination** mandate for all municipal workers. (Luiz C. Ribeiro/for New York Daily News) |
| [526](https://www.english-corpora.org/corona/x4.asp?rs=&t=88206903&ID=22166425023) | [21-11-02 US](https://www.english-corpora.org/corona/x4.asp?rs=&t=88206903&ID=22166425023) | [Washington Post](https://www.english-corpora.org/corona/x3.asp?node=&p=6&w10=coronavirus&w11=vaccination&r=) |  |  |  | Advertisement Vaccine mandates # A slight majority of Virginia voters favored employers requiring **coronavirus** **vaccination** for their employees, while just over 4 in 10 opposed such a measure, |
| [527](https://www.english-corpora.org/corona/x4.asp?rs=&t=88212766&ID=22180339551) | [21-11-03 US](https://www.english-corpora.org/corona/x4.asp?rs=&t=88212766&ID=22180339551) | [The Hill](https://www.english-corpora.org/corona/x3.asp?node=&p=6&w10=coronavirus&w11=vaccination&r=) |  |  |  | the nation's largest pharmacy chains -- CVS Health and Walgreens -- are accepting **coronavirus** **vaccination** appointments for children ages 5-11. # CVS Health announced Wednesday that it will be |
| [528](https://www.english-corpora.org/corona/x4.asp?rs=&t=88213154&ID=22179985780) | [21-11-03 US](https://www.english-corpora.org/corona/x4.asp?rs=&t=88213154&ID=22179985780) | [New York Times](https://www.english-corpora.org/corona/x3.asp?node=&p=6&w10=coronavirus&w11=vaccination&r=) |  |  |  | with nearly all troops vaccinated. # U.S. Air Force troops helping run a **coronavirus** **vaccination** site in Houston in February. The vaccine deadline for that branch of service arrived |
| [529](https://www.english-corpora.org/corona/x4.asp?rs=&t=88213502&ID=22178008029) | [21-11-03 US](https://www.english-corpora.org/corona/x4.asp?rs=&t=88213502&ID=22178008029) | [Fox News](https://www.english-corpora.org/corona/x3.asp?node=&p=6&w10=coronavirus&w11=vaccination&r=) |  |  |  | revoking medical licenses of firefighters and EMS workers who don't submit to a **coronavirus** **vaccination**. # In the letter, Republican Reps. Andy Harris, Madison Cawthorn, |
| [531](https://www.english-corpora.org/corona/x4.asp?rs=&t=88213904&ID=22178380213) | [21-11-03 US](https://www.english-corpora.org/corona/x4.asp?rs=&t=88213904&ID=22178380213) | [Roll Call](https://www.english-corpora.org/corona/x3.asp?node=&p=6&w10=coronavirus&w11=vaccination&r=)[(1)](https://www.english-corpora.org/corona/duplicates1.asp?xx=530&n=1) |  |  |  | for debate # Under new Defense Department and federal government directives, a **coronavirus** **vaccination** is mandatory for not only servicemembers but also Defense Department civilians and contractors, except |
| [533](https://www.english-corpora.org/corona/x4.asp?rs=&t=88214431&ID=22177675253) | [21-11-03 US](https://www.english-corpora.org/corona/x4.asp?rs=&t=88214431&ID=22177675253) | [Forbes](https://www.english-corpora.org/corona/x3.asp?node=&p=6&w10=coronavirus&w11=vaccination&r=)[(1)](https://www.english-corpora.org/corona/duplicates1.asp?xx=532&n=1) |  |  |  | " homeopathic treatment " he received from his doctor to count as a **coronavirus** **vaccination**. The treatment involved Rodgers' personal doctor raising his antibody levels, according to |
| [534](https://www.english-corpora.org/corona/x4.asp?rs=&t=88215648&ID=22190847132) | [21-11-04 US](https://www.english-corpora.org/corona/x4.asp?rs=&t=88215648&ID=22190847132) | [Associated Press](https://www.english-corpora.org/corona/x3.asp?node=&p=6&w10=coronavirus&w11=vaccination&r=) |  |  |  | 's ability to maintain safe and efficient operations; the impact and timing of **coronavirus** **vaccination** programs; issues with vessel operations; higher than expected costs and expenses, off-hire |
| [535](https://www.english-corpora.org/corona/x4.asp?rs=&t=88216981&ID=22191197519) | [21-11-04 US](https://www.english-corpora.org/corona/x4.asp?rs=&t=88216981&ID=22191197519) | [Associated Press](https://www.english-corpora.org/corona/x3.asp?node=&p=6&w10=coronavirus&w11=vaccination&r=) |  |  |  | 's ability to maintain safe and efficient operations; the impact and timing of **coronavirus** **vaccination** programs; the impact of geopolitical tensions and changes in global economic conditions; greater |
| [536](https://www.english-corpora.org/corona/x4.asp?rs=&t=88218491&ID=22191286484) | [21-11-04 US](https://www.english-corpora.org/corona/x4.asp?rs=&t=88218491&ID=22191286484) | [SFGate](https://www.english-corpora.org/corona/x3.asp?node=&p=6&w10=coronavirus&w11=vaccination&r=) |  |  |  | general pushed back Thursday, Nov. 4, 2021 against President Joe Biden's **coronavirus** **vaccination** mandate for private employers, filing a lawsuit claiming the requirement amounts to government overreach |
| [538](https://www.english-corpora.org/corona/x4.asp?rs=&t=88219744&ID=22190364673) | [21-11-04 US](https://www.english-corpora.org/corona/x4.asp?rs=&t=88219744&ID=22190364673) | [CNBC](https://www.english-corpora.org/corona/x3.asp?node=&p=6&w10=coronavirus&w11=vaccination&r=)[(1)](https://www.english-corpora.org/corona/duplicates1.asp?xx=537&n=1) |  |  |  | is now 92%. # The New York City mayor had declared his **coronavirus** **vaccination** order for emergency responders a success on Monday, with no disruption to city services |
| [539](https://www.english-corpora.org/corona/x4.asp?rs=&t=88220417&ID=22189089935) | [21-11-04 US](https://www.english-corpora.org/corona/x4.asp?rs=&t=88220417&ID=22189089935) | [The Hill](https://www.english-corpora.org/corona/x3.asp?node=&p=6&w10=coronavirus&w11=vaccination&r=) |  |  |  | multiple violations in a workplace. # President Biden stood by his administration's **coronavirus** **vaccination** mandate for businesses, arguing on Thursday it will not lead to worker shortages amid |
| [540](https://www.english-corpora.org/corona/x4.asp?rs=&t=88228217&ID=22211174330) | [21-11-06 US](https://www.english-corpora.org/corona/x4.asp?rs=&t=88228217&ID=22211174330) | [New York Post](https://www.english-corpora.org/corona/x3.asp?node=&p=6&w10=coronavirus&w11=vaccination&r=) |  |  |  | In all, six city agencies still lag behind the Big Apple's overall **coronavirus** **vaccination** rate for adults, and about 9,000 city workers are on unpaid leave for refusing |
| [542](https://www.english-corpora.org/corona/x4.asp?rs=&t=88232015&ID=22218878160) | [21-11-07 US](https://www.english-corpora.org/corona/x4.asp?rs=&t=88232015&ID=22218878160) | [YAHOO!News](https://www.english-corpora.org/corona/x3.asp?node=&p=6&w10=coronavirus&w11=vaccination&r=)[(1)](https://www.english-corpora.org/corona/duplicates1.asp?xx=541&n=1) |  |  |  | puppet Big Bird announced his **coronavirus** **vaccination** on Twitter -- and drew fire from conservatives such as Ted Cruz, who dismissed |
| [543](https://www.english-corpora.org/corona/x4.asp?rs=&t=88236122&ID=22231818586) | [21-11-08 US](https://www.english-corpora.org/corona/x4.asp?rs=&t=88236122&ID=22231818586) | [Associated Press](https://www.english-corpora.org/corona/x3.asp?node=&p=6&w10=coronavirus&w11=vaccination&r=) |  |  |  | troubled by a provision empowering citizens to sue businesses they believe slighted them with **coronavirus** **vaccination** requirements, which the bill largely bans for companies and government entities. # So |
| [544](https://www.english-corpora.org/corona/x4.asp?rs=&t=88241115&ID=22232406697) | [21-11-08 US](https://www.english-corpora.org/corona/x4.asp?rs=&t=88241115&ID=22232406697) | [New York Times](https://www.english-corpora.org/corona/x3.asp?node=&p=6&w10=coronavirus&w11=vaccination&r=) |  |  |  | Mexico, in the predawn hours on Monday, as tourists with proof of **coronavirus** **vaccination** joined the mix of students, essential workers and returning Americans entering the United States |
| [545](https://www.english-corpora.org/corona/x4.asp?rs=&t=88245446&ID=22247767322) | [21-11-09 US](https://www.english-corpora.org/corona/x4.asp?rs=&t=88245446&ID=22247767322) | [New York Daily News](https://www.english-corpora.org/corona/x3.asp?node=&p=6&w10=coronavirus&w11=vaccination&r=) |  |  |  | Monday, Oct. 25, 2021, to protest Mayor de Blasio's looming **coronavirus** **vaccination** mandate for all municipal workers in the city. (Luiz C. Ribeiro for New |
| [552](https://www.english-corpora.org/corona/x4.asp?rs=&t=88247559&ID=22259846146) | [21-11-10 US](https://www.english-corpora.org/corona/x4.asp?rs=&t=88247559&ID=22259846146) | [ABC](https://www.english-corpora.org/corona/x3.asp?node=&p=6&w10=coronavirus&w11=vaccination&r=)[(6)](https://www.english-corpora.org/corona/duplicates1.asp?xx=551&n=6) |  |  |  | was sort of bewildered, " he said during a visit to a **coronavirus** **vaccination** clinic in Los Angeles. " I drove into the office every single day, |
| [553](https://www.english-corpora.org/corona/x4.asp?rs=&t=88249644&ID=22259233437) | [21-11-10 US](https://www.english-corpora.org/corona/x4.asp?rs=&t=88249644&ID=22259233437) | [The Guardian on MSN.com](https://www.english-corpora.org/corona/x3.asp?node=&p=6&w10=coronavirus&w11=vaccination&r=) |  |  |  | and Biden had a " very productive " discussion about the need to increase **coronavirus** **vaccination** rates. # Biden is now en route to Baltimore, Maryland, where he |
| [554](https://www.english-corpora.org/corona/x4.asp?rs=&t=88252210&ID=22257054956) | [21-11-10 US](https://www.english-corpora.org/corona/x4.asp?rs=&t=88252210&ID=22257054956) | [Fox News](https://www.english-corpora.org/corona/x3.asp?node=&p=6&w10=coronavirus&w11=vaccination&r=) |  |  |  | In June, the liberal news outlet published a report that boasted China's **coronavirus** **vaccination** rates... according to data provided by China. # A CNN reporter was accused |
| [556](https://www.english-corpora.org/corona/x4.asp?rs=&t=88254183&ID=22274456652) | [21-11-11 US](https://www.english-corpora.org/corona/x4.asp?rs=&t=88254183&ID=22274456652) | [YAHOO!News](https://www.english-corpora.org/corona/x3.asp?node=&p=6&w10=coronavirus&w11=vaccination&r=)[(1)](https://www.english-corpora.org/corona/duplicates1.asp?xx=555&n=1) |  |  |  | a year, will be reviewed: MOH # People enter a Covid-19 **coronavirus** **vaccination** centre set up at a community centre in Singapore on October 7, 2021. |
| [557](https://www.english-corpora.org/corona/x4.asp?rs=&t=88260919&ID=22285225273) | [21-11-12 US](https://www.english-corpora.org/corona/x4.asp?rs=&t=88260919&ID=22285225273) | [YAHOO!News](https://www.english-corpora.org/corona/x3.asp?node=&p=6&w10=coronavirus&w11=vaccination&r=) |  |  |  | Parts of Eastern Europe have also seen a sharp rise in cases. # **Coronavirus** **vaccination** clinics for Virginia Beach city employees were canceled through the rest of the year due |
| [558](https://www.english-corpora.org/corona/x4.asp?rs=&t=88261396&ID=22288180237) | [21-11-12 US](https://www.english-corpora.org/corona/x4.asp?rs=&t=88261396&ID=22288180237) | [The Washington Post on MSN.com](https://www.english-corpora.org/corona/x3.asp?node=&p=6&w10=coronavirus&w11=vaccination&r=) |  |  |  | of Inc.com. 88261396 # Nearly a year after **coronavirus** **vaccination** campaigns began in earnest around the globe, a number of mainly Western countries with |
| [559](https://www.english-corpora.org/corona/x4.asp?rs=&t=88262164&ID=22286252931) | [21-11-12 US](https://www.english-corpora.org/corona/x4.asp?rs=&t=88262164&ID=22286252931) | [Chicago Tribune](https://www.english-corpora.org/corona/x3.asp?node=&p=6&w10=coronavirus&w11=vaccination&r=) |  |  |  | a measure intended to prevent a decades-old state law from being used to skirt **coronavirus** **vaccination** requirements. # While the change to the state's Health Care Right of Conscience |
| [560](https://www.english-corpora.org/corona/x4.asp?rs=&t=88264894&ID=22288017638) | [21-11-12 US](https://www.english-corpora.org/corona/x4.asp?rs=&t=88264894&ID=22288017638) | [SFGate](https://www.english-corpora.org/corona/x3.asp?node=&p=6&w10=coronavirus&w11=vaccination&r=) |  |  |  | ) -- The Madison Catholic Diocese has instructed its 102 parishes not to host **coronavirus** **vaccination** clinics in their schools and churches. # The diocese directive follows federal action which |
| [561](https://www.english-corpora.org/corona/x4.asp?rs=&t=88266290&ID=22296492983) | [21-11-13 US](https://www.english-corpora.org/corona/x4.asp?rs=&t=88266290&ID=22296492983) | [YAHOO!Finance](https://www.english-corpora.org/corona/x3.asp?node=&p=6&w10=coronavirus&w11=vaccination&r=) |  |  |  | patients with solid tumors. Oncorus reported that ONCR-177 was well tolerated. # **Coronavirus** **vaccination** clinics for Virginia Beach city employees were canceled through the rest of the year due |
| [562](https://www.english-corpora.org/corona/x4.asp?rs=&t=88267310&ID=22294340598) | [21-11-13 US](https://www.english-corpora.org/corona/x4.asp?rs=&t=88267310&ID=22294340598) | [Associated Press](https://www.english-corpora.org/corona/x3.asp?node=&p=6&w10=coronavirus&w11=vaccination&r=) |  |  |  | ALBANY, N.Y. (AP) -- Ten of New York's mass **coronavirus** **vaccination** sites are ready to give shots to children ages 5 to 11 who recently became |
| [563](https://www.english-corpora.org/corona/x4.asp?rs=&t=88268230&ID=22294390556) | [21-11-13 US](https://www.english-corpora.org/corona/x4.asp?rs=&t=88268230&ID=22294390556) | [YAHOO!News](https://www.english-corpora.org/corona/x3.asp?node=&p=6&w10=coronavirus&w11=vaccination&r=) |  |  |  | and that of other Daily Herald journalists by subscribing to the publication. # **Coronavirus** **vaccination** clinics for Virginia Beach city employees were canceled through the rest of the year due |
| [564](https://www.english-corpora.org/corona/x4.asp?rs=&t=88268811&ID=22294427035) | [21-11-13 US](https://www.english-corpora.org/corona/x4.asp?rs=&t=88268811&ID=22294427035) | [YAHOO!News](https://www.english-corpora.org/corona/x3.asp?node=&p=6&w10=coronavirus&w11=vaccination&r=) |  |  |  | station spans 150,000 square feet and is located at 14601 Sweitzer Lane. # **Coronavirus** **vaccination** clinics for Virginia Beach city employees were canceled through the rest of the year due |
| [565](https://www.english-corpora.org/corona/x4.asp?rs=&t=88268820&ID=22294427875) | [21-11-13 US](https://www.english-corpora.org/corona/x4.asp?rs=&t=88268820&ID=22294427875) | [Yahoo! Sports](https://www.english-corpora.org/corona/x3.asp?node=&p=6&w10=coronavirus&w11=vaccination&r=) |  |  |  | returned to work, told Self for a cover story published Friday. # **Coronavirus** **vaccination** clinics for Virginia Beach city employees were canceled through the rest of the year due |
| [566](https://www.english-corpora.org/corona/x4.asp?rs=&t=88270441&ID=22303303603) | [21-11-14 US](https://www.english-corpora.org/corona/x4.asp?rs=&t=88270441&ID=22303303603) | [YAHOO!Finance](https://www.english-corpora.org/corona/x3.asp?node=&p=6&w10=coronavirus&w11=vaccination&r=) |  |  |  | longer. Read on to find out more -- and to ensure you # **Coronavirus** **vaccination** clinics for Virginia Beach city employees were canceled through the rest of the year due |
| [567](https://www.english-corpora.org/corona/x4.asp?rs=&t=88274074&ID=22305218240) | [21-11-14 US](https://www.english-corpora.org/corona/x4.asp?rs=&t=88274074&ID=22305218240) | [Radio Free Europe/Radio Liberty](https://www.english-corpora.org/corona/x3.asp?node=&p=6&w10=coronavirus&w11=vaccination&r=) |  |  |  | POZh-70, Voronkin and other soldiers were sent by truck to receive their second **coronavirus** **vaccination** shots. Neither the soldiers with him nor the doctors who oversaw the vaccinations noted |
| [568](https://www.english-corpora.org/corona/x4.asp?rs=&t=88284921&ID=22332523148) | [21-11-16 US](https://www.english-corpora.org/corona/x4.asp?rs=&t=88284921&ID=22332523148) | [New York Times](https://www.english-corpora.org/corona/x3.asp?node=&p=6&w10=coronavirus&w11=vaccination&r=) |  |  |  | be eased on Nov. 22, reflecting a shift away from mask requirements as **coronavirus** **vaccination** rates continue to rise and infections fueled by the Delta variant subside in the city |
| [569](https://www.english-corpora.org/corona/x4.asp?rs=&t=88284921&ID=22332527817) | [21-11-16 US](https://www.english-corpora.org/corona/x4.asp?rs=&t=88284921&ID=22332527817) | [New York Times](https://www.english-corpora.org/corona/x3.asp?node=&p=6&w10=coronavirus&w11=vaccination&r=) |  |  |  | discretion over whether students should get vaccinated or wear masks. # Administering a **coronavirus** **vaccination** at a hub in Sydney, Australia, in October.Credit... Lisa Maree Williams/Getty Images |
| [570](https://www.english-corpora.org/corona/x4.asp?rs=&t=88297362&ID=22345628648) | [21-11-18 US](https://www.english-corpora.org/corona/x4.asp?rs=&t=88297362&ID=22345628648) | [Boston Herald](https://www.english-corpora.org/corona/x3.asp?node=&p=6&w10=coronavirus&w11=vaccination&r=) |  |  |  | , deaths and disrupted education. (AP Photo/Carolyn Kaster) # Hundreds of **coronavirus** **vaccination** sites for kids will come online in Massachusetts starting Thursday in an effort to get |
| [571](https://www.english-corpora.org/corona/x4.asp?rs=&t=88306628&ID=22356554103) | [21-11-19 US](https://www.english-corpora.org/corona/x4.asp?rs=&t=88306628&ID=22356554103) | [New York Times](https://www.english-corpora.org/corona/x3.asp?node=&p=6&w10=coronavirus&w11=vaccination&r=) |  |  |  | Thursday, Austria will go into a nationwide lockdown on Monday and impose a **coronavirus** **vaccination** mandate in February, the first such mandate in a Western democracy. # Health |
| [572](https://www.english-corpora.org/corona/x4.asp?rs=&t=88309685&ID=22367612708) | [21-11-20 US](https://www.english-corpora.org/corona/x4.asp?rs=&t=88309685&ID=22367612708) | [NJ.com](https://www.english-corpora.org/corona/x3.asp?node=&p=6&w10=coronavirus&w11=vaccination&r=) |  |  |  | 11th day in a row and as Phil Murphy encouraged adult residents to get **coronavirus** **vaccination** booster shots a day after federal officials expanded eligibility to anyone 18 and older. |
| [573](https://www.english-corpora.org/corona/x4.asp?rs=&t=88313134&ID=22377965662) | [21-11-21 US](https://www.english-corpora.org/corona/x4.asp?rs=&t=88313134&ID=22377965662) | [The Independent on MSN.com](https://www.english-corpora.org/corona/x3.asp?node=&p=6&w10=coronavirus&w11=vaccination&r=) |  |  |  | 17:00, Emily Atkinson # Health secretary Sajid Javid has ruled out mass mandatory **coronavirus** **vaccination**, saying that it is not something that the government would ever " look at |
| [574](https://www.english-corpora.org/corona/x4.asp?rs=&t=88313416&ID=22376895440) | [21-11-21 US](https://www.english-corpora.org/corona/x4.asp?rs=&t=88313416&ID=22376895440) | [Daily Mail on MSN.com](https://www.english-corpora.org/corona/x3.asp?node=&p=6&w10=coronavirus&w11=vaccination&r=) |  |  |  | ' # People aged 40 and over will be able to book their third **coronavirus** **vaccination** as of tomorrow. Sixteen and 17-year-olds will also be able to book in for |
| [575](https://www.english-corpora.org/corona/x4.asp?rs=&t=88316665&ID=22378172609) | [21-11-21 US](https://www.english-corpora.org/corona/x4.asp?rs=&t=88316665&ID=22378172609) | [Washington Post](https://www.english-corpora.org/corona/x3.asp?node=&p=6&w10=coronavirus&w11=vaccination&r=) |  |  |  | had demanded all week after he was exposed for misleading the public about his **coronavirus** **vaccination** status: an explanation. # Advertisement # Story continues below advertisement # He should |
| [576](https://www.english-corpora.org/corona/x4.asp?rs=&t=88319183&ID=22392526846) | [21-11-22 US](https://www.english-corpora.org/corona/x4.asp?rs=&t=88319183&ID=22392526846) | [Radio Free Europe/Radio Liberty](https://www.english-corpora.org/corona/x3.asp?node=&p=6&w10=coronavirus&w11=vaccination&r=) |  |  |  | rumors that a 21-year-old man had died in Romania six days after receiving a **coronavirus** **vaccination**. That broadcast got some 1 million views and was shared 17,800 times. # |
| [577](https://www.english-corpora.org/corona/x4.asp?rs=&t=43597437&ID=22149197053) | [21-11-01 GB](https://www.english-corpora.org/corona/x4.asp?rs=&t=43597437&ID=22149197053) | [joe.co.uk](https://www.english-corpora.org/corona/x3.asp?node=&p=6&w10=coronavirus&w11=vaccination&r=) |  |  |  | aged between 12 and 15 will now be offered a first dose of the **coronavirus** **vaccination**, with the plan rolling out in more than 800 schools across the UK. |
| [578](https://www.english-corpora.org/corona/x4.asp?rs=&t=43666916&ID=22348638251) | [21-11-19 GB](https://www.english-corpora.org/corona/x4.asp?rs=&t=43666916&ID=22348638251) | [politico.eu](https://www.english-corpora.org/corona/x3.asp?node=&p=6&w10=coronavirus&w11=vaccination&r=) |  |  |  | country -- and one of the first in the world -- to impose compulsory **coronavirus** **vaccination**. # " We have not managed to get enough unvaccinated people to get vaccinated |
| [579](https://www.english-corpora.org/corona/x4.asp?rs=&t=43666916&ID=22348638481) | [21-11-19 GB](https://www.english-corpora.org/corona/x4.asp?rs=&t=43666916&ID=22348638481) | [politico.eu](https://www.english-corpora.org/corona/x3.asp?node=&p=6&w10=coronavirus&w11=vaccination&r=) |  |  |  | care -- and which will not. Skeptical and vulnerable # Takeup of **coronavirus** **vaccination** by Austria's nine million people has been relatively low by European standards, at |
| [580](https://www.english-corpora.org/corona/x4.asp?rs=&t=43671118&ID=22368945688) | [21-11-21 GB](https://www.english-corpora.org/corona/x4.asp?rs=&t=43671118&ID=22368945688) | [politico.eu](https://www.english-corpora.org/corona/x3.asp?node=&p=6&w10=coronavirus&w11=vaccination&r=) |  |  |  | Vienna against lockdown and mandatory **coronavirus** **vaccination** # Thousands protested in Austria against government measures. # A demonstrator holds up a |
| [581](https://www.english-corpora.org/corona/x4.asp?rs=&t=43671118&ID=22368945850) | [21-11-21 GB](https://www.english-corpora.org/corona/x4.asp?rs=&t=43671118&ID=22368945850) | [politico.eu](https://www.english-corpora.org/corona/x3.asp?node=&p=6&w10=coronavirus&w11=vaccination&r=) |  |  |  | were also penalized. # Following Friday's announcement by Chancellor Alexander Schallenberg that **coronavirus** **vaccination** would become mandatory as of February, the authorities had expected a large protest and |
| [582](https://www.english-corpora.org/corona/x4.asp?rs=&t=43680278&ID=22399578251) | [21-11-23 GB](https://www.english-corpora.org/corona/x4.asp?rs=&t=43680278&ID=22399578251) | [politico.eu](https://www.english-corpora.org/corona/x3.asp?node=&p=6&w10=coronavirus&w11=vaccination&r=) |  |  |  | is believed to have sold more than 2,000 fake **coronavirus** **vaccination** certificates, authorities said Tuesday. # Doctors in Belgium have access to a system |
| [583](https://www.english-corpora.org/corona/x4.asp?rs=&t=73682323&ID=22309556794) | [21-11-15 GB](https://www.english-corpora.org/corona/x4.asp?rs=&t=73682323&ID=22309556794) | [echo-news.co.uk](https://www.english-corpora.org/corona/x3.asp?node=&p=6&w10=coronavirus&w11=vaccination&r=) |  |  |  | has urged women who are or are intending to become pregnant to get their **coronavirus** **vaccination** as he set out some " stark facts " showing the majority of expectant mothers |
| [584](https://www.english-corpora.org/corona/x4.asp?rs=&t=63498583&ID=22152540359) | [21-11-01 IE](https://www.english-corpora.org/corona/x4.asp?rs=&t=63498583&ID=22152540359) | [extra.ie](https://www.english-corpora.org/corona/x3.asp?node=&p=6&w10=coronavirus&w11=vaccination&r=) |  |  |  | that there is a' steady number of people' coming forward for their **coronavirus** **vaccination** as of late. # Sharing the update to Twitter on Monday morning, Mr |
| [585](https://www.english-corpora.org/corona/x4.asp?rs=&t=63498583&ID=22152540442) | [21-11-01 IE](https://www.english-corpora.org/corona/x4.asp?rs=&t=63498583&ID=22152540442) | [extra.ie](https://www.english-corpora.org/corona/x3.asp?node=&p=6&w10=coronavirus&w11=vaccination&r=) |  |  |  | that there is a' steady number of people' coming forward for their **coronavirus** **vaccination** as of late. Pic: Sam Boal/Photocall Ireland # Positively, we continue to |
| [586](https://www.english-corpora.org/corona/x4.asp?rs=&t=63516049&ID=22207258970) | [21-11-06 IE](https://www.english-corpora.org/corona/x4.asp?rs=&t=63516049&ID=22207258970) | [intellinews.com](https://www.english-corpora.org/corona/x3.asp?node=&p=6&w10=coronavirus&w11=vaccination&r=) |  |  |  | more uncertain than usual, the Fund acknowledged, urging Georgia to increase its **coronavirus** **vaccination** rate. # Georgian health officials are alarmed and concerned by the recent low vaccination |
| [587](https://www.english-corpora.org/corona/x4.asp?rs=&t=73626472&ID=22165115149) | [21-11-02 AU](https://www.english-corpora.org/corona/x4.asp?rs=&t=73626472&ID=22165115149) | [perthnow.com.au](https://www.english-corpora.org/corona/x3.asp?node=&p=6&w10=coronavirus&w11=vaccination&r=) |  |  |  | . # AMERICAS # * New York City Mayor Bill de Blasio declared his **coronavirus** **vaccination** order for emergency responders a success, with no disruption to city services, despite |
| [588](https://www.english-corpora.org/corona/x4.asp?rs=&t=73627102&ID=22160139197) | [21-11-02 AU](https://www.english-corpora.org/corona/x4.asp?rs=&t=73627102&ID=22160139197) | [themandarin.com.au](https://www.english-corpora.org/corona/x3.asp?node=&p=6&w10=coronavirus&w11=vaccination&r=) |  |  |  | having the vaccine. # Price calls any attempt by people to avoid the **coronavirus** **vaccination** as being a " cynical ploy ". # " Anxiety is taken extremely seriously |
| [589](https://www.english-corpora.org/corona/x4.asp?rs=&t=73677973&ID=22316211132) | [21-11-15 AU](https://www.english-corpora.org/corona/x4.asp?rs=&t=73677973&ID=22316211132) | [watoday.com.au](https://www.english-corpora.org/corona/x3.asp?node=&p=6&w10=coronavirus&w11=vaccination&r=) |  |  |  | where he likely infected a 30-year-old woman, who wasn't immunised. # **Coronavirus** **vaccination** rates in Katherine hover about the 80 per cent mark, but the government is |
| [590](https://www.english-corpora.org/corona/x4.asp?rs=&t=73663272&ID=22269986140) | [21-11-11 IN](https://www.english-corpora.org/corona/x4.asp?rs=&t=73663272&ID=22269986140) | [timesnownews.com](https://www.english-corpora.org/corona/x3.asp?node=&p=6&w10=coronavirus&w11=vaccination&r=) |  |  |  | been recognised by 96 countries, and they have agreed to mutually accept the **coronavirus** **vaccination** certificates from India. # " The World Health Organization has included 8 vaccines in |
| [591](https://www.english-corpora.org/corona/x4.asp?rs=&t=73693210&ID=22336519685) | [21-11-18 IN](https://www.english-corpora.org/corona/x4.asp?rs=&t=73693210&ID=22336519685) | [indiatoday.in](https://www.english-corpora.org/corona/x3.asp?node=&p=6&w10=coronavirus&w11=vaccination&r=) |  |  |  | or less will need to submit evidence of their injury and its relationship to **coronavirus** **vaccination**, medical costs and lost wages. # The Australian government is yet to release |
| [592](https://www.english-corpora.org/corona/x4.asp?rs=&t=53298728&ID=22194013150) | [21-11-05 PK](https://www.english-corpora.org/corona/x4.asp?rs=&t=53298728&ID=22194013150) | [samaa.tv](https://www.english-corpora.org/corona/x3.asp?node=&p=6&w10=coronavirus&w11=vaccination&r=) |  |  |  | report is, on the other hand, mandatory for all passengers. Complete **coronavirus** **vaccination** too is mandatory for travelers. All passengers of and above six years, whether |
| [593](https://www.english-corpora.org/corona/x4.asp?rs=&t=53303767&ID=22209267766) | [21-11-06 PK](https://www.english-corpora.org/corona/x4.asp?rs=&t=53303767&ID=22209267766) | [dailytimes.com.pk](https://www.english-corpora.org/corona/x3.asp?node=&p=6&w10=coronavirus&w11=vaccination&r=) |  |  |  | National Health Services, Dr Nausheen Hamid Monday said that the government has kick-started **coronavirus** **vaccination** for youngsters age group (15-18) years from September 13. " Teens below |
| [594](https://www.english-corpora.org/corona/x4.asp?rs=&t=73629401&ID=22160422590) | [21-11-02 PH](https://www.english-corpora.org/corona/x4.asp?rs=&t=73629401&ID=22160422590) | [pageone.ph](https://www.english-corpora.org/corona/x3.asp?node=&p=6&w10=coronavirus&w11=vaccination&r=) |  |  |  | the anti-coronavirus crisis center, in Russia, over 50.9 million full cycles of **coronavirus** **vaccination** have already been carried out, and the first component was vaccinated 56.8 million times |
| [595](https://www.english-corpora.org/corona/x4.asp?rs=&t=73629510&ID=22160443586) | [21-11-02 PH](https://www.english-corpora.org/corona/x4.asp?rs=&t=73629510&ID=22160443586) | [pna.gov.ph](https://www.english-corpora.org/corona/x3.asp?node=&p=6&w10=coronavirus&w11=vaccination&r=) |  |  |  | vax program to minors # MANILA -- The **coronavirus** **vaccination** rollout to the rest of the pediatric population aged 12 to 17 years in Taguig |
| [596](https://www.english-corpora.org/corona/x4.asp?rs=&t=73683393&ID=22317189144) | [21-11-15 PH](https://www.english-corpora.org/corona/x4.asp?rs=&t=73683393&ID=22317189144) | [rappler.com](https://www.english-corpora.org/corona/x3.asp?node=&p=6&w10=coronavirus&w11=vaccination&r=) |  |  |  | vaccination site at bus terminal # The Zamboanga City Health Office opened its biggest **coronavirus** **vaccination** site at the city's Integrated Bus Terminal as it stepped up its campaign to |
| [597](https://www.english-corpora.org/corona/x4.asp?rs=&t=43617607&ID=22204083854) | [21-11-06 HK](https://www.english-corpora.org/corona/x4.asp?rs=&t=43617607&ID=22204083854) | [hongkongfp.com](https://www.english-corpora.org/corona/x3.asp?node=&p=6&w10=coronavirus&w11=vaccination&r=) |  |  |  | Friday, the relevant groups may book their third jabs on the government's **coronavirus** **vaccination** website, civil service chief Patrick Nip said. They will also be able to |
| [598](https://www.english-corpora.org/corona/x4.asp?rs=&t=43617607&ID=22204083937) | [21-11-06 HK](https://www.english-corpora.org/corona/x4.asp?rs=&t=43617607&ID=22204083937) | [hongkongfp.com](https://www.english-corpora.org/corona/x3.asp?node=&p=6&w10=coronavirus&w11=vaccination&r=) |  |  |  | or documents proving that they work in high-exposure professions, Nip said. # **Coronavirus** **vaccination** in Hong Kong. Photo: GovHK. # Those who received two Sinovac jabs |
| [599](https://www.english-corpora.org/corona/x4.asp?rs=&t=63556410&ID=22324531834) | [21-11-16 ZA](https://www.english-corpora.org/corona/x4.asp?rs=&t=63556410&ID=22324531834) | [ecr.co.za](https://www.english-corpora.org/corona/x3.asp?node=&p=6&w10=coronavirus&w11=vaccination&r=) |  |  |  | " difficult " lockdown imposed on Monday on unvaccinated people had led to increased **coronavirus** **vaccination** rates. # aschallenberg # " The developments in recent days, the huge increases |
| [600](https://www.english-corpora.org/corona/x4.asp?rs=&t=88260313&ID=22284241780) | [21-11-12 --](https://www.english-corpora.org/corona/x4.asp?rs=&t=88260313&ID=22284241780) | [Rferl.org](https://www.english-corpora.org/corona/x3.asp?node=&p=6&w10=coronavirus&w11=vaccination&r=) |  |  |  | reporting by RFE/RL's Radio Azadi. 88260322 # **Coronavirus** **vaccination** clinics |

| PAGE:   [**<<**](https://www.english-corpora.org/corona/x3.asp?node=&p=1&w10=coronavirus&w11=vaccination&r=) [**<**](https://www.english-corpora.org/corona/x3.asp?node=&p=5&w10=coronavirus&w11=vaccination&r=)   6 / 42  [**>**](https://www.english-corpora.org/corona/x3.asp?node=&p=7&w10=coronavirus&w11=vaccination&r=) [**>>**](https://www.english-corpora.org/corona/x3.asp?node=&p=42&w10=coronavirus&w11=vaccination&r=) |
| --- |

Конец формы

| FIND SAMPLE:  [100](https://www.english-corpora.org/corona/x3.asp?node=&sample=100&w10=coronavirus&w11=vaccination&r=)  [200](https://www.english-corpora.org/corona/x3.asp?node=&sample=200&w10=coronavirus&w11=vaccination&r=)  [500](https://www.english-corpora.org/corona/x3.asp?node=&sample=500&w10=coronavirus&w11=vaccination&r=)  [1000](https://www.english-corpora.org/corona/x3.asp?node=&sample=1000&w10=coronavirus&w11=vaccination&r=) PAGE:   [**<<**](https://www.english-corpora.org/corona/x3.asp?node=&p=1&w10=coronavirus&w11=vaccination&r=) [**<**](https://www.english-corpora.org/corona/x3.asp?node=&p=6&w10=coronavirus&w11=vaccination&r=)   7 / 42  [**>**](https://www.english-corpora.org/corona/x3.asp?node=&p=8&w10=coronavirus&w11=vaccination&r=) [**>>**](https://www.english-corpora.org/corona/x3.asp?node=&p=42&w10=coronavirus&w11=vaccination&r=) |  |
| --- | --- |

Начало формы

| CLICK FOR MORE CONTEXT | [**HELP**](javascript:newFeatures()) | [SAVE](javascript:chooser('s'))    [TRANSLATE](javascript:chooser('t'))    [ANALYZE](javascript:chooser('p')) |
| --- | --- | --- |

| [601](https://www.english-corpora.org/corona/x4.asp?rs=&t=33295582&ID=21884962386) | [21-10-06 US](https://www.english-corpora.org/corona/x4.asp?rs=&t=33295582&ID=21884962386) | [nytimes.com](https://www.english-corpora.org/corona/x3.asp?node=&p=7&w10=coronavirus&w11=vaccination&r=) |  |  |  | require most people to provide proof of full **coronavirus** **vaccination** to enter a range of indoor businesses, including restaurants, gyms, museums, |
| --- | --- | --- | --- | --- | --- | --- |
| [602](https://www.english-corpora.org/corona/x4.asp?rs=&t=33297181&ID=21885211938) | [21-10-06 US](https://www.english-corpora.org/corona/x4.asp?rs=&t=33297181&ID=21885211938) | [nytimes.com](https://www.english-corpora.org/corona/x3.asp?node=&p=7&w10=coronavirus&w11=vaccination&r=) |  |  |  | is an explicit insularity and parochialism that now dictates debate. " # A **coronavirus** **vaccination** clinic at Shorter Community AME Church in Denver in February.Credit... Kevin Mohatt for The |
| [603](https://www.english-corpora.org/corona/x4.asp?rs=&t=33311760&ID=21925799324) | [21-10-10 US](https://www.english-corpora.org/corona/x4.asp?rs=&t=33311760&ID=21925799324) | [nytimes.com](https://www.english-corpora.org/corona/x3.asp?node=&p=7&w10=coronavirus&w11=vaccination&r=) |  |  |  | an executive order in August directing all county employees to show proof of a **coronavirus** **vaccination** by Oct. 1. The order did not provide for a testing option as some |
| [604](https://www.english-corpora.org/corona/x4.asp?rs=&t=33318064&ID=21947026399) | [21-10-12 US](https://www.english-corpora.org/corona/x4.asp?rs=&t=33318064&ID=21947026399) | [thehill.com](https://www.english-corpora.org/corona/x3.asp?node=&p=7&w10=coronavirus&w11=vaccination&r=) |  |  |  | show host says he was fired after refusing to comply with the company's **coronavirus** **vaccination** policy. # Tim Hill, a host of " The Game " show on |
| [605](https://www.english-corpora.org/corona/x4.asp?rs=&t=33337644&ID=22016195322) | [21-10-18 US](https://www.english-corpora.org/corona/x4.asp?rs=&t=33337644&ID=22016195322) | [politico.com](https://www.english-corpora.org/corona/x3.asp?node=&p=7&w10=coronavirus&w11=vaccination&r=) |  |  |  | surcharge. # States spent at least $89 million on lotteries aimed at boosting **coronavirus** **vaccination** rates -- and it might not have made a difference, reports Insider's Erin |
| [606](https://www.english-corpora.org/corona/x4.asp?rs=&t=33352968&ID=22066493340) | [21-10-23 US](https://www.english-corpora.org/corona/x4.asp?rs=&t=33352968&ID=22066493340) | [politico.com](https://www.english-corpora.org/corona/x3.asp?node=&p=7&w10=coronavirus&w11=vaccination&r=) |  |  |  | of students needing to quarantine. And, most people eligible to receive a **coronavirus** **vaccination** in the district have received at least one dose, he said. " |
| [607](https://www.english-corpora.org/corona/x4.asp?rs=&t=33353514&ID=22063674995) | [21-10-23 US](https://www.english-corpora.org/corona/x4.asp?rs=&t=33353514&ID=22063674995) | [wbur.org](https://www.english-corpora.org/corona/x3.asp?node=&p=7&w10=coronavirus&w11=vaccination&r=) |  |  |  | first school in Massachusetts to make face coverings optional after reaching the state-set 80% **coronavirus** **vaccination** threshold. # The Hopkinton School Committee voted 3-2 on Thursday night to give vaccinated |
| [608](https://www.english-corpora.org/corona/x4.asp?rs=&t=33364321&ID=22088563387) | [21-10-26 US](https://www.english-corpora.org/corona/x4.asp?rs=&t=33364321&ID=22088563387) | [ndtv.com](https://www.english-corpora.org/corona/x3.asp?node=&p=7&w10=coronavirus&w11=vaccination&r=) |  |  |  | , India's cumulative COVID-19 vaccination coverage crossed 100 crore doses today. The **coronavirus** **vaccination** drive was launched on January 16, 2021 and the government wants all of India |
| [609](https://www.english-corpora.org/corona/x4.asp?rs=&t=33369022&ID=22108070816) | [21-10-28 US](https://www.english-corpora.org/corona/x4.asp?rs=&t=33369022&ID=22108070816) | [chicagotribune.com](https://www.english-corpora.org/corona/x3.asp?node=&p=7&w10=coronavirus&w11=vaccination&r=) |  |  |  | polarizing proposal aimed at preventing people from using a decades-old state law to skirt **coronavirus** **vaccination** mandates by citing moral or religious objections. # Before adjourning for the year, |
| [610](https://www.english-corpora.org/corona/x4.asp?rs=&t=43540413&ID=21970337584) | [21-10-14 US](https://www.english-corpora.org/corona/x4.asp?rs=&t=43540413&ID=21970337584) | [news4jax.com](https://www.english-corpora.org/corona/x3.asp?node=&p=7&w10=coronavirus&w11=vaccination&r=) |  |  |  | # The lending agency called Thursday for greater efforts from wealthy nations to boost **coronavirus** **vaccination** rates in poorer countries, while also urging the Federal Reserve and other central banks |
| [611](https://www.english-corpora.org/corona/x4.asp?rs=&t=63424427&ID=21903196067) | [21-10-08 US](https://www.english-corpora.org/corona/x4.asp?rs=&t=63424427&ID=21903196067) | [witn.com](https://www.english-corpora.org/corona/x3.asp?node=&p=7&w10=coronavirus&w11=vaccination&r=) |  |  |  | 2021, three vials of the Moderna COVID-19 Vaccine are pictured in a new **coronavirus** **vaccination** center at the' Velodrom' in Berlin, Germany. (AP Photo/Michael Sohn |
| [613](https://www.english-corpora.org/corona/x4.asp?rs=&t=73540826&ID=21916090830) | [21-10-09 US](https://www.english-corpora.org/corona/x4.asp?rs=&t=73540826&ID=21916090830) | [news.trust.org](https://www.english-corpora.org/corona/x3.asp?node=&p=7&w10=coronavirus&w11=vaccination&r=)[(1)](https://www.english-corpora.org/corona/duplicates1.asp?xx=612&n=1) |  |  |  | used water cannon to push back hundreds of people demonstrating in Rome against **coronavirus** **vaccination**, seeking to keep them from the prime minister's office. # Around 10,000 |
| [614](https://www.english-corpora.org/corona/x4.asp?rs=&t=73563822&ID=21984342263) | [21-10-15 US](https://www.english-corpora.org/corona/x4.asp?rs=&t=73563822&ID=21984342263) | [theweek.com](https://www.english-corpora.org/corona/x3.asp?node=&p=7&w10=coronavirus&w11=vaccination&r=) |  |  |  | made by companies that didn't make their original vaccine. Administration says **coronavirus** **vaccination** rates rose after mandates # U.S. coronavirus vaccination rates have jumped by more than 20 |
| [615](https://www.english-corpora.org/corona/x4.asp?rs=&t=73563822&ID=21984342271) | [21-10-15 US](https://www.english-corpora.org/corona/x4.asp?rs=&t=73563822&ID=21984342271) | [theweek.com](https://www.english-corpora.org/corona/x3.asp?node=&p=7&w10=coronavirus&w11=vaccination&r=) |  |  |  | original vaccine. Administration says coronavirus vaccination rates rose after mandates # U.S. **coronavirus** **vaccination** rates have jumped by more than 20 percentage points since numerous businesses, hospital systems |
| [616](https://www.english-corpora.org/corona/x4.asp?rs=&t=88063415&ID=21889536439) | [21-10-06 US](https://www.english-corpora.org/corona/x4.asp?rs=&t=88063415&ID=21889536439) | [New York Times](https://www.english-corpora.org/corona/x3.asp?node=&p=7&w10=coronavirus&w11=vaccination&r=) |  |  |  | in a blog post and pointed out that the White House had missed its **coronavirus** **vaccination** goals. # " Facebook is not the reason this goal was missed, " |
| [618](https://www.english-corpora.org/corona/x4.asp?rs=&t=88067629&ID=21898630436) | [21-10-07 US](https://www.english-corpora.org/corona/x4.asp?rs=&t=88067629&ID=21898630436) | [Bloomberg on MSN.com](https://www.english-corpora.org/corona/x3.asp?node=&p=7&w10=coronavirus&w11=vaccination&r=)[(1)](https://www.english-corpora.org/corona/duplicates1.asp?xx=617&n=1) |  |  |  | -- President Joe Biden escalated his campaign to pressure private employers into imposing **coronavirus** **vaccination** mandates in a trip to a Chicago suburb, saying shots would save lives and |
| [619](https://www.english-corpora.org/corona/x4.asp?rs=&t=88070610&ID=21897650380) | [21-10-07 US](https://www.english-corpora.org/corona/x4.asp?rs=&t=88070610&ID=21897650380) | [The Guardian on MSN.com](https://www.english-corpora.org/corona/x3.asp?node=&p=7&w10=coronavirus&w11=vaccination&r=) |  |  |  | of vaccinated adults. # Anybody aged 12 and older is eligible for a **coronavirus** **vaccination** in Germany. # 14:29 # Hungary has offered its help to neighbouring Romania in |
| [620](https://www.english-corpora.org/corona/x4.asp?rs=&t=88071417&ID=21898542290) | [21-10-07 US](https://www.english-corpora.org/corona/x4.asp?rs=&t=88071417&ID=21898542290) | [Washington Post](https://www.english-corpora.org/corona/x3.asp?node=&p=7&w10=coronavirus&w11=vaccination&r=) |  |  |  | in Norfolk, has repeatedly refused to say publicly whether she has gotten a **coronavirus** **vaccination**. She did not respond to requests for comment from The Washington Post, but |
| [621](https://www.english-corpora.org/corona/x4.asp?rs=&t=88077821&ID=21920646066) | [21-10-09 US](https://www.english-corpora.org/corona/x4.asp?rs=&t=88077821&ID=21920646066) | [YAHOO!News](https://www.english-corpora.org/corona/x3.asp?node=&p=7&w10=coronavirus&w11=vaccination&r=) |  |  |  | recovery extended to all cases aged 5 to 49 # People enter a Covid-19 **coronavirus** **vaccination** centre set up at a community centre in Singapore on October 7, 2021. / |
| [622](https://www.english-corpora.org/corona/x4.asp?rs=&t=88078316&ID=21921509976) | [21-10-09 US](https://www.english-corpora.org/corona/x4.asp?rs=&t=88078316&ID=21921509976) | [The Guardian on MSN.com](https://www.english-corpora.org/corona/x3.asp?node=&p=7&w10=coronavirus&w11=vaccination&r=) |  |  |  | police used water cannon to push back hundreds of people demonstrating in Rome against **coronavirus** **vaccination** rules, seeking to keep them from the prime minister's office, Reuters reports |
| [624](https://www.english-corpora.org/corona/x4.asp?rs=&t=88080367&ID=21923899889) | [21-10-09 US](https://www.english-corpora.org/corona/x4.asp?rs=&t=88080367&ID=21923899889) | [The Motley Fool](https://www.english-corpora.org/corona/x3.asp?node=&p=7&w10=coronavirus&w11=vaccination&r=)[(1)](https://www.english-corpora.org/corona/duplicates1.asp?xx=623&n=1) |  |  |  | jobs on the line. # A number of major employers are imposing **coronavirus** **vaccination** requirements, and workers who don't comply -- and also don't qualify for |
| [625](https://www.english-corpora.org/corona/x4.asp?rs=&t=88086141&ID=21929637391) | [21-10-10 US](https://www.english-corpora.org/corona/x4.asp?rs=&t=88086141&ID=21929637391) | [SFGate](https://www.english-corpora.org/corona/x3.asp?node=&p=7&w10=coronavirus&w11=vaccination&r=) |  |  |  | West said on Twitter. The Republican candidate said he has not gotten a **coronavirus** **vaccination** and that doctors were worried Saturday about the lowered level of oxygen saturation in his |
| [626](https://www.english-corpora.org/corona/x4.asp?rs=&t=88101626&ID=21966664778) | [21-10-13 US](https://www.english-corpora.org/corona/x4.asp?rs=&t=88101626&ID=21966664778) | [New York Times](https://www.english-corpora.org/corona/x3.asp?node=&p=7&w10=coronavirus&w11=vaccination&r=) |  |  |  | period of rapid inflation than they had expected. # A Johnson & Johnson **coronavirus** **vaccination** site in Newark in March.Credit... James Estrin/The New York Times # 2. People |
| [627](https://www.english-corpora.org/corona/x4.asp?rs=&t=88105037&ID=21979365455) | [21-10-14 US](https://www.english-corpora.org/corona/x4.asp?rs=&t=88105037&ID=21979365455) | [Kaiser Health News](https://www.english-corpora.org/corona/x3.asp?node=&p=7&w10=coronavirus&w11=vaccination&r=) |  |  |  | are well on their way to meeting Thursday's deadline for the state's **coronavirus** **vaccination** mandate, with several citing vaccination rates of 90% or higher. California was the |
| [628](https://www.english-corpora.org/corona/x4.asp?rs=&t=88105196&ID=21980860362) | [21-10-14 US](https://www.english-corpora.org/corona/x4.asp?rs=&t=88105196&ID=21980860362) | [Patch](https://www.english-corpora.org/corona/x3.asp?node=&p=7&w10=coronavirus&w11=vaccination&r=) |  |  |  | on the map below and click on a pin to see that community's **coronavirus** **vaccination** rates. You can also view the town-by-town coronavirus vaccination data in the spreadsheet we |
| [629](https://www.english-corpora.org/corona/x4.asp?rs=&t=88105196&ID=21980860372) | [21-10-14 US](https://www.english-corpora.org/corona/x4.asp?rs=&t=88105196&ID=21980860372) | [Patch](https://www.english-corpora.org/corona/x3.asp?node=&p=7&w10=coronavirus&w11=vaccination&r=) |  |  |  | see that community's coronavirus vaccination rates. You can also view the town-by-town **coronavirus** **vaccination** data in the spreadsheet we used to create this map. # Colors reflect the |
| [630](https://www.english-corpora.org/corona/x4.asp?rs=&t=88110090&ID=21980220181) | [21-10-14 US](https://www.english-corpora.org/corona/x4.asp?rs=&t=88110090&ID=21980220181) | [The Hill](https://www.english-corpora.org/corona/x3.asp?node=&p=7&w10=coronavirus&w11=vaccination&r=) |  |  |  | disease. # United in recent months has been considered an industry leader in **coronavirus** **vaccination** mandates. It was the first airline company to mandate that its employees get vaccinated |
| [631](https://www.english-corpora.org/corona/x4.asp?rs=&t=88113672&ID=21993478992) | [21-10-15 US](https://www.english-corpora.org/corona/x4.asp?rs=&t=88113672&ID=21993478992) | [The Washington Post on MSN.com](https://www.english-corpora.org/corona/x3.asp?node=&p=7&w10=coronavirus&w11=vaccination&r=) |  |  |  | " Galassetti said. # But Peron -- who said he was suspicious of **coronavirus** **vaccination** and did not trust the mainstream media's information -- said he didn't know |
| [632](https://www.english-corpora.org/corona/x4.asp?rs=&t=88125590&ID=22012404469) | [21-10-17 US](https://www.english-corpora.org/corona/x4.asp?rs=&t=88125590&ID=22012404469) | [Yahoo](https://www.english-corpora.org/corona/x3.asp?node=&p=7&w10=coronavirus&w11=vaccination&r=) |  |  |  | to enjoy. " Dr. Fauci Thinks Mask Mandates Work # Check-in for **coronavirus** **vaccination** against Covid-19 with doctor in the background. # " Texas Governor Greg Abbott, |
| [633](https://www.english-corpora.org/corona/x4.asp?rs=&t=88131316&ID=22022321068) | [21-10-18 US](https://www.english-corpora.org/corona/x4.asp?rs=&t=88131316&ID=22022321068) | [Washington Post](https://www.english-corpora.org/corona/x3.asp?node=&p=7&w10=coronavirus&w11=vaccination&r=) |  |  |  | and deaths from covid-19 in the city. The wards also have the lowest **coronavirus** **vaccination** rates. # Advertisement # Story continues below advertisement # " I'm really hopeful |
| [634](https://www.english-corpora.org/corona/x4.asp?rs=&t=88134743&ID=22033734557) | [21-10-19 US](https://www.english-corpora.org/corona/x4.asp?rs=&t=88134743&ID=22033734557) | [The Guardian on MSN.com](https://www.english-corpora.org/corona/x3.asp?node=&p=7&w10=coronavirus&w11=vaccination&r=) |  |  |  | their jobs as increasing numbers of states, cities and companies bring in mandatory **coronavirus** **vaccination** mandates. # The latest high profile example, reports Reuters, came on Monday |
| [635](https://www.english-corpora.org/corona/x4.asp?rs=&t=88134770&ID=22035009641) | [21-10-19 US](https://www.english-corpora.org/corona/x4.asp?rs=&t=88134770&ID=22035009641) | [New York Times](https://www.english-corpora.org/corona/x3.asp?node=&p=7&w10=coronavirus&w11=vaccination&r=) |  |  |  | The Ministry of Health in Ukraine, the country with the lowest rate of **coronavirus** **vaccination** in Europe, reported on Tuesday that 538 people had died of Covid-19 in the |
| [636](https://www.english-corpora.org/corona/x4.asp?rs=&t=88134981&ID=22035023577) | [21-10-19 US](https://www.english-corpora.org/corona/x4.asp?rs=&t=88134981&ID=22035023577) | [Washington Post](https://www.english-corpora.org/corona/x3.asp?node=&p=7&w10=coronavirus&w11=vaccination&r=) |  |  |  | health-care system is giving its employees a choice as it seeks to boost **coronavirus** **vaccination** numbers: ensure your spouse has received a shot or face higher health insurance costs |
| [637](https://www.english-corpora.org/corona/x4.asp?rs=&t=88134981&ID=22035023757) | [21-10-19 US](https://www.english-corpora.org/corona/x4.asp?rs=&t=88134981&ID=22035023757) | [Washington Post](https://www.english-corpora.org/corona/x3.asp?node=&p=7&w10=coronavirus&w11=vaccination&r=) |  |  |  | large companies in the United States to include dependent spouses and partners in a **coronavirus** **vaccination** surcharge policy. With doses widely available in the United States and the Pfizer-BioNTech vaccine |
| [638](https://www.english-corpora.org/corona/x4.asp?rs=&t=88140310&ID=22043044998) | [21-10-20 US](https://www.english-corpora.org/corona/x4.asp?rs=&t=88140310&ID=22043044998) | [Miami Herald on MSN.com](https://www.english-corpora.org/corona/x3.asp?node=&p=7&w10=coronavirus&w11=vaccination&r=) |  |  |  | students needing to quarantine. # And, most people eligible to receive a **coronavirus** **vaccination** in the district have received at least one dose, he said. # Speaking |
| [639](https://www.english-corpora.org/corona/x4.asp?rs=&t=88142359&ID=22045902365) | [21-10-20 US](https://www.english-corpora.org/corona/x4.asp?rs=&t=88142359&ID=22045902365) | [New York Daily News](https://www.english-corpora.org/corona/x3.asp?node=&p=7&w10=coronavirus&w11=vaccination&r=) |  |  |  | COVID spikes # A week after Russian President Vladimir Putin called for upping the **coronavirus** **vaccination** rate, he has ordered most of his fellow citizens to stay home from work |
| [640](https://www.english-corpora.org/corona/x4.asp?rs=&t=88142910&ID=22043197419) | [21-10-20 US](https://www.english-corpora.org/corona/x4.asp?rs=&t=88142910&ID=22043197419) | [The Washington Post on MSN.com](https://www.english-corpora.org/corona/x3.asp?node=&p=7&w10=coronavirus&w11=vaccination&r=) |  |  |  | over the summer that he would not perform at venues that require proof of **coronavirus** **vaccination** for people to attend. Clapton went back on that promise last month when he |
| [641](https://www.english-corpora.org/corona/x4.asp?rs=&t=88142926&ID=22044081053) | [21-10-20 US](https://www.english-corpora.org/corona/x4.asp?rs=&t=88142926&ID=22044081053) | [New York Times](https://www.english-corpora.org/corona/x3.asp?node=&p=7&w10=coronavirus&w11=vaccination&r=) |  |  |  | coronavirus **vaccination** card in Jackson, Ala., earlier this month.Credit... Charity Rachelle for The New |
| [642](https://www.english-corpora.org/corona/x4.asp?rs=&t=88142926&ID=22044081053) | [21-10-20 US](https://www.english-corpora.org/corona/x4.asp?rs=&t=88142926&ID=22044081053) | [New York Times](https://www.english-corpora.org/corona/x3.asp?node=&p=7&w10=coronavirus&w11=vaccination&r=) |  |  |  | coronavirus **vaccination** card in Jackson, Ala., earlier this month.Credit... Charity Rachelle for The New |
| [643](https://www.english-corpora.org/corona/x4.asp?rs=&t=88144138&ID=22055364375) | [21-10-21 US](https://www.english-corpora.org/corona/x4.asp?rs=&t=88144138&ID=22055364375) | [The Baltimore Sun](https://www.english-corpora.org/corona/x3.asp?node=&p=7&w10=coronavirus&w11=vaccination&r=) |  |  |  | before the event, audience members waited in lines to have their identification and **coronavirus** **vaccination** cards checked. # The network solicited questions from the community through institutions such as |
| [645](https://www.english-corpora.org/corona/x4.asp?rs=&t=88146818&ID=22053921838) | [21-10-21 US](https://www.english-corpora.org/corona/x4.asp?rs=&t=88146818&ID=22053921838) | [Yahoo](https://www.english-corpora.org/corona/x3.asp?node=&p=7&w10=coronavirus&w11=vaccination&r=)[(1)](https://www.english-corpora.org/corona/duplicates1.asp?xx=644&n=1) |  |  |  | and concerns about the coming rule that will require many companies to implement **coronavirus** **vaccination** or testing protocols for their workers, according to records posted on a government website |
| [646](https://www.english-corpora.org/corona/x4.asp?rs=&t=88150981&ID=22062402085) | [21-10-22 US](https://www.english-corpora.org/corona/x4.asp?rs=&t=88150981&ID=22062402085) | [The Baltimore Sun](https://www.english-corpora.org/corona/x3.asp?node=&p=7&w10=coronavirus&w11=vaccination&r=) |  |  |  | militarily with China. The president has little patience for anti-vaxxers # Addressing **coronavirus** **vaccination** mandates, Biden said he thinks they're working. He said people have not |
| [647](https://www.english-corpora.org/corona/x4.asp?rs=&t=88153126&ID=22061058548) | [21-10-22 US](https://www.english-corpora.org/corona/x4.asp?rs=&t=88153126&ID=22061058548) | [AFP on MSN.com](https://www.english-corpora.org/corona/x3.asp?node=&p=7&w10=coronavirus&w11=vaccination&r=) |  |  |  | over slow jab drive as deaths rise # The Kremlin conceded Friday its **coronavirus** **vaccination** drive was struggling and blamed Russians for not getting inoculated as deaths and cases hit |
| [648](https://www.english-corpora.org/corona/x4.asp?rs=&t=88156020&ID=22067788680) | [21-10-23 US](https://www.english-corpora.org/corona/x4.asp?rs=&t=88156020&ID=22067788680) | [The Guardian on MSN.com](https://www.english-corpora.org/corona/x3.asp?node=&p=7&w10=coronavirus&w11=vaccination&r=) |  |  |  | hospital system is stretched to breaking point. # The country has the second-lowest **coronavirus** **vaccination** rate in the EU. Record Covid deaths in Russia for fifth straight day |
| [649](https://www.english-corpora.org/corona/x4.asp?rs=&t=88156547&ID=22069615032) | [21-10-23 US](https://www.english-corpora.org/corona/x4.asp?rs=&t=88156547&ID=22069615032) | [AOL](https://www.english-corpora.org/corona/x3.asp?node=&p=7&w10=coronavirus&w11=vaccination&r=) |  |  |  | social media users are falsely claiming it shows Indigenous people in Australia staving off **coronavirus** **vaccination** efforts. " The Australian Aborigines are defending themselves with bow and arrow against the |
| [650](https://www.english-corpora.org/corona/x4.asp?rs=&t=88157128&ID=22068790713) | [21-10-23 US](https://www.english-corpora.org/corona/x4.asp?rs=&t=88157128&ID=22068790713) | [Washington Post](https://www.english-corpora.org/corona/x3.asp?node=&p=7&w10=coronavirus&w11=vaccination&r=) |  |  |  | cancel its plans to turn 2,000 adult doses into 6,000 pediatric doses for a **coronavirus** **vaccination** clinic if it can not use existing vaccine doses and is unable to get the |
| [651](https://www.english-corpora.org/corona/x4.asp?rs=&t=88157128&ID=22068790869) | [21-10-23 US](https://www.english-corpora.org/corona/x4.asp?rs=&t=88157128&ID=22068790869) | [Washington Post](https://www.english-corpora.org/corona/x3.asp?node=&p=7&w10=coronavirus&w11=vaccination&r=) |  |  |  | federally funded program that provides vaccines at no cost to children -- into the **coronavirus** **vaccination** program. # The U.S. Health and Human Services and the Education departments also plan |
| [652](https://www.english-corpora.org/corona/x4.asp?rs=&t=88160656&ID=22076662540) | [21-10-24 US](https://www.english-corpora.org/corona/x4.asp?rs=&t=88160656&ID=22076662540) | [The Hill on MSN.com](https://www.english-corpora.org/corona/x3.asp?node=&p=7&w10=coronavirus&w11=vaccination&r=) |  |  |  | announcing he had tested positive for COVID-19, urged all Americans to receive a **coronavirus** **vaccination** in order to keep themselves and others safe. # " For god's sake |
| [654](https://www.english-corpora.org/corona/x4.asp?rs=&t=88163805&ID=22075560737) | [21-10-24 US](https://www.english-corpora.org/corona/x4.asp?rs=&t=88163805&ID=22075560737) | [The Independent on MSN.com](https://www.english-corpora.org/corona/x3.asp?node=&p=7&w10=coronavirus&w11=vaccination&r=)[(1)](https://www.english-corpora.org/corona/duplicates1.asp?xx=653&n=1) |  |  |  | Sabin # About 75.6 per cent of people in China have completed a **coronavirus** **vaccination** programme, the country's health authorities have said today. # This is the |
| [655](https://www.english-corpora.org/corona/x4.asp?rs=&t=88164020&ID=22075127659) | [21-10-24 US](https://www.english-corpora.org/corona/x4.asp?rs=&t=88164020&ID=22075127659) | [The Guardian on MSN.com](https://www.english-corpora.org/corona/x3.asp?node=&p=7&w10=coronavirus&w11=vaccination&r=) |  |  |  | 17:27 # In Portugal, where it has one of the world's highest **coronavirus** **vaccination** rates, it is learning to live with Covid-19 while easing its restrictions. # |
| [656](https://www.english-corpora.org/corona/x4.asp?rs=&t=88165734&ID=22086419942) | [21-10-25 US](https://www.english-corpora.org/corona/x4.asp?rs=&t=88165734&ID=22086419942) | [Yahoo! Sports](https://www.english-corpora.org/corona/x3.asp?node=&p=7&w10=coronavirus&w11=vaccination&r=) |  |  |  | Brooklyn Bridge roadway Monday in their fight against Mayor Bill de Blasio's looming **coronavirus** **vaccination** mandate for all municipal workers in the city. The mandate, which takes effect |
| [657](https://www.english-corpora.org/corona/x4.asp?rs=&t=88171040&ID=22092206991) | [21-10-26 US](https://www.english-corpora.org/corona/x4.asp?rs=&t=88171040&ID=22092206991) | [Associated Press](https://www.english-corpora.org/corona/x3.asp?node=&p=7&w10=coronavirus&w11=vaccination&r=) |  |  |  | two months, falling below 3% statewide. # Though the last surge boosted **coronavirus** **vaccination** interest, Louisiana continues to have one of the country's lowest immunization rates -- |
| [658](https://www.english-corpora.org/corona/x4.asp?rs=&t=88171446&ID=22093027835) | [21-10-26 US](https://www.english-corpora.org/corona/x4.asp?rs=&t=88171446&ID=22093027835) | [Chicago Tribune on MSN.com](https://www.english-corpora.org/corona/x3.asp?node=&p=7&w10=coronavirus&w11=vaccination&r=) |  |  |  | a proposal seeking to prevent people from using another decades-old state law to skirt **coronavirus** **vaccination** requirements by citing moral or religious objections. # And with a new Illinois congressional |
| [659](https://www.english-corpora.org/corona/x4.asp?rs=&t=88172191&ID=22093599811) | [21-10-26 US](https://www.english-corpora.org/corona/x4.asp?rs=&t=88172191&ID=22093599811) | [YAHOO!News](https://www.english-corpora.org/corona/x3.asp?node=&p=7&w10=coronavirus&w11=vaccination&r=) |  |  |  | Brooklyn Bridge roadway Monday in their fight against Mayor Bill de Blasio's looming **coronavirus** **vaccination** mandate for all municipal workers in the city. The mandate, which takes effect |
| [661](https://www.english-corpora.org/corona/x4.asp?rs=&t=88183995&ID=22117775273) | [21-10-28 US](https://www.english-corpora.org/corona/x4.asp?rs=&t=88183995&ID=22117775273) | [New York Daily News](https://www.english-corpora.org/corona/x3.asp?node=&p=7&w10=coronavirus&w11=vaccination&r=)[(1)](https://www.english-corpora.org/corona/duplicates1.asp?xx=660&n=1) |  |  |  | amid backlash # Mayor de Blasio didn't bat an eye at flagging **coronavirus** **vaccination** rates for cops, firefighters and garbage collectors on Thursday -- even as the deadline |
| [662](https://www.english-corpora.org/corona/x4.asp?rs=&t=53221468&ID=21896479997) | [21-10-07 CA](https://www.english-corpora.org/corona/x4.asp?rs=&t=53221468&ID=21896479997) | [thompsoncitizen.net](https://www.english-corpora.org/corona/x3.asp?node=&p=7&w10=coronavirus&w11=vaccination&r=) |  |  |  | number of vaccinated adults. Anybody aged 12 and older is eligible for a **coronavirus** **vaccination** in Germany. # Spahn said the higher-than-thought number of fully vaccinated people in Germany |
| [663](https://www.english-corpora.org/corona/x4.asp?rs=&t=53228459&ID=21917243900) | [21-10-09 CA](https://www.english-corpora.org/corona/x4.asp?rs=&t=53228459&ID=21917243900) | [toronto.citynews.ca](https://www.english-corpora.org/corona/x3.asp?node=&p=7&w10=coronavirus&w11=vaccination&r=) |  |  |  | 2021, three vials of the Moderna COVID-19 Vaccine are pictured in a new **coronavirus** **vaccination** center at the' Velodrom' in Berlin, Germany. (AP Photo/Michael Sohn |
| [664](https://www.english-corpora.org/corona/x4.asp?rs=&t=53231233&ID=21925221908) | [21-10-10 CA](https://www.english-corpora.org/corona/x4.asp?rs=&t=53231233&ID=21925221908) | [570news.com](https://www.english-corpora.org/corona/x3.asp?node=&p=7&w10=coronavirus&w11=vaccination&r=) |  |  |  | West said on Twitter. The Republican candidate said he has not gotten a **coronavirus** **vaccination** and that doctors were worried Saturday about the lowered level of oxygen saturation in his |
| [665](https://www.english-corpora.org/corona/x4.asp?rs=&t=63414052&ID=21883850412) | [21-10-06 IE](https://www.english-corpora.org/corona/x4.asp?rs=&t=63414052&ID=21883850412) | [intellinews.com](https://www.english-corpora.org/corona/x3.asp?node=&p=7&w10=coronavirus&w11=vaccination&r=) |  |  |  | more uncertain than usual, the Fund acknowledged, urging Georgia to increase its **coronavirus** **vaccination** rate. # Georgian health officials are alarmed and concerned by the recent low vaccination |
| [666](https://www.english-corpora.org/corona/x4.asp?rs=&t=73526736&ID=21884320704) | [21-10-06 AU](https://www.english-corpora.org/corona/x4.asp?rs=&t=73526736&ID=21884320704) | [theage.com.au](https://www.english-corpora.org/corona/x3.asp?node=&p=7&w10=coronavirus&w11=vaccination&r=) |  |  |  | symptoms and come forward for vaccination. # After climbing rapidly last week, **coronavirus** **vaccination** rates in NSW have felt the impact of the state's public holiday weekend. |
| [667](https://www.english-corpora.org/corona/x4.asp?rs=&t=73531247&ID=21896113678) | [21-10-07 AU](https://www.english-corpora.org/corona/x4.asp?rs=&t=73531247&ID=21896113678) | [watoday.com.au](https://www.english-corpora.org/corona/x3.asp?node=&p=7&w10=coronavirus&w11=vaccination&r=) |  |  |  | federal Liberal MP Russell Broadbent announced last week that he would not receive a **coronavirus** **vaccination**. # Several Liberal Party sources confirmed a motion was put before the Warragul branch |
| [668](https://www.english-corpora.org/corona/x4.asp?rs=&t=73547344&ID=21948579447) | [21-10-12 AU](https://www.english-corpora.org/corona/x4.asp?rs=&t=73547344&ID=21948579447) | [businessinsider.com.au](https://www.english-corpora.org/corona/x3.asp?node=&p=7&w10=coronavirus&w11=vaccination&r=) |  |  |  | about that, " Sutton said during yesterday's coronavirus update. # As **coronavirus** **vaccination** rates rise in NSW and Victoria, it's possible both states might move along |
| [669](https://www.english-corpora.org/corona/x4.asp?rs=&t=73547785&ID=21951303170) | [21-10-12 AU](https://www.english-corpora.org/corona/x4.asp?rs=&t=73547785&ID=21951303170) | [perthnow.com.au](https://www.english-corpora.org/corona/x3.asp?node=&p=7&w10=coronavirus&w11=vaccination&r=) |  |  |  | lockdown has slowed the potential growth. # In an effort to drive up **coronavirus** **vaccination** faster, the state has launched neighbourhood pop-up clinics in suburban cafes, shops and |
| [670](https://www.english-corpora.org/corona/x4.asp?rs=&t=73547979&ID=21951333189) | [21-10-12 AU](https://www.english-corpora.org/corona/x4.asp?rs=&t=73547979&ID=21951333189) | [thecourier.com.au](https://www.english-corpora.org/corona/x3.asp?node=&p=7&w10=coronavirus&w11=vaccination&r=) |  |  |  | registered 1612 infections and eight fatalities. * Western Australia is looking to make **coronavirus** **vaccination** mandatory in some other sectors, on top of the already announced port, transport |
| [678](https://www.english-corpora.org/corona/x4.asp?rs=&t=73553268&ID=21965146851) | [21-10-13 AU](https://www.english-corpora.org/corona/x4.asp?rs=&t=73553268&ID=21965146851) | [westernadvocate.com.au](https://www.english-corpora.org/corona/x3.asp?node=&p=7&w10=coronavirus&w11=vaccination&r=)[(7)](https://www.english-corpora.org/corona/duplicates1.asp?xx=677&n=7) |  |  |  | the 70 per cent mark. Australians are facing an increasing patchwork of **coronavirus** **vaccination** mandates with different rules across state borders and industries. Vaccination requirements are now in |
| [679](https://www.english-corpora.org/corona/x4.asp?rs=&t=73557196&ID=21976475209) | [21-10-14 AU](https://www.english-corpora.org/corona/x4.asp?rs=&t=73557196&ID=21976475209) | [mamamia.com.au](https://www.english-corpora.org/corona/x3.asp?node=&p=7&w10=coronavirus&w11=vaccination&r=) |  |  |  | state's 1.25 million authorised workers a fortnight to get at least their first **coronavirus** **vaccination** by Friday - or show proof of a booking within the next week - otherwise |
| [680](https://www.english-corpora.org/corona/x4.asp?rs=&t=73557395&ID=21976517214) | [21-10-14 AU](https://www.english-corpora.org/corona/x4.asp?rs=&t=73557395&ID=21976517214) | [indaily.com.au](https://www.english-corpora.org/corona/x3.asp?node=&p=7&w10=coronavirus&w11=vaccination&r=) |  |  |  | state's 1.25 million authorised workers a fortnight to get at least their first **coronavirus** **vaccination** by Friday -- or show proof of a booking within the next week -- otherwise |
| [682](https://www.english-corpora.org/corona/x4.asp?rs=&t=73596940&ID=22073289720) | [21-10-24 AU](https://www.english-corpora.org/corona/x4.asp?rs=&t=73596940&ID=22073289720) | [themandarin.com.au](https://www.english-corpora.org/corona/x3.asp?node=&p=7&w10=coronavirus&w11=vaccination&r=)[(1)](https://www.english-corpora.org/corona/duplicates1.asp?xx=681&n=1) |  |  |  | the people who might otherwise be attacked. " # With mandates for **coronavirus** **vaccination** applying to some areas of public service, Murphy said he was not supportive of |
| [683](https://www.english-corpora.org/corona/x4.asp?rs=&t=73611717&ID=22109915975) | [21-10-28 AU](https://www.english-corpora.org/corona/x4.asp?rs=&t=73611717&ID=22109915975) | [examiner.com.au](https://www.english-corpora.org/corona/x3.asp?node=&p=7&w10=coronavirus&w11=vaccination&r=) |  |  |  | trying to firebomb a police station amid growing volatility over Western Australia's mandatory **coronavirus** **vaccination** plans. Police allege the 32-year-old man on Wednesday ignited a water bottle containing an |
| [684](https://www.english-corpora.org/corona/x4.asp?rs=&t=73611717&ID=22109916581) | [21-10-28 AU](https://www.english-corpora.org/corona/x4.asp?rs=&t=73611717&ID=22109916581) | [examiner.com.au](https://www.english-corpora.org/corona/x3.asp?node=&p=7&w10=coronavirus&w11=vaccination&r=) |  |  |  | trying to firebomb a police station amid growing volatility over Western Australia's mandatory **coronavirus** **vaccination** plans. # Police allege the 32-year-old man on Wednesday ignited a water bottle containing |
| [685](https://www.english-corpora.org/corona/x4.asp?rs=&t=73529753&ID=21884745271) | [21-10-06 IN](https://www.english-corpora.org/corona/x4.asp?rs=&t=73529753&ID=21884745271) | [india.com](https://www.english-corpora.org/corona/x3.asp?node=&p=7&w10=coronavirus&w11=vaccination&r=) |  |  |  | German government such as; travellers will still have to provide a proof of **coronavirus** **vaccination**, or a negative test result, or a proof of recovery from COVID-19. |
| [686](https://www.english-corpora.org/corona/x4.asp?rs=&t=73553821&ID=21958884474) | [21-10-13 IN](https://www.english-corpora.org/corona/x4.asp?rs=&t=73553821&ID=21958884474) | [bloombergquint.com](https://www.english-corpora.org/corona/x3.asp?node=&p=7&w10=coronavirus&w11=vaccination&r=) |  |  |  | order issued Monday barring any entity, including an employer, from requiring a **coronavirus** **vaccination** puts him squarely in opposition to the plans of many Texas businesses. Florida Governor |
| [687](https://www.english-corpora.org/corona/x4.asp?rs=&t=73577749&ID=22029426636) | [21-10-19 IN](https://www.english-corpora.org/corona/x4.asp?rs=&t=73577749&ID=22029426636) | [economictimes.indiatimes.com](https://www.english-corpora.org/corona/x3.asp?node=&p=7&w10=coronavirus&w11=vaccination&r=) |  |  |  | , are being jabbed with needles rather than vampiric fangs this weekend in a **coronavirus** **vaccination** drive. # Everyone ushered 2021 on a hopeful note. Businesses had just started |
| [688](https://www.english-corpora.org/corona/x4.asp?rs=&t=73593591&ID=22067485270) | [21-10-23 IN](https://www.english-corpora.org/corona/x4.asp?rs=&t=73593591&ID=22067485270) | [timesnownews.com](https://www.english-corpora.org/corona/x3.asp?node=&p=7&w10=coronavirus&w11=vaccination&r=) |  |  |  | like its other schemes, there is no " VIP culture " in the **coronavirus** **vaccination** drive either. # PM Modi expressed gratitude to health workers, including doctors, |
| [689](https://www.english-corpora.org/corona/x4.asp?rs=&t=73593649&ID=22066091236) | [21-10-23 IN](https://www.english-corpora.org/corona/x4.asp?rs=&t=73593649&ID=22066091236) | [economictimes.indiatimes.com](https://www.english-corpora.org/corona/x3.asp?node=&p=7&w10=coronavirus&w11=vaccination&r=) |  |  |  | tour agencies comes amid growing demand for overseas trips on the back of fast-rising **Coronavirus** **vaccination** rates and Seoul's " travel bubble " pacts with other countries. # The |
| [690](https://www.english-corpora.org/corona/x4.asp?rs=&t=53226394&ID=21907892767) | [21-10-08 PK](https://www.english-corpora.org/corona/x4.asp?rs=&t=53226394&ID=21907892767) | [suchtv.pk](https://www.english-corpora.org/corona/x3.asp?node=&p=7&w10=coronavirus&w11=vaccination&r=) |  |  |  | vaccinated before november 30: Dr Faisal Sultan # The government has launched a **coronavirus** **vaccination** campaign for school children under which it is mandatory to get them fully vaccinated by |
| [691](https://www.english-corpora.org/corona/x4.asp?rs=&t=53250389&ID=21988973156) | [21-10-15 PK](https://www.english-corpora.org/corona/x4.asp?rs=&t=53250389&ID=21988973156) | [samaa.tv](https://www.english-corpora.org/corona/x3.asp?node=&p=7&w10=coronavirus&w11=vaccination&r=) |  |  |  | On October 11, educational institutions across Pakistan reopened. The government has made **coronavirus** **vaccination** mandatory for students of and above the age of 12 years. According to SAPM |
| [693](https://www.english-corpora.org/corona/x4.asp?rs=&t=53242924&ID=21970540527) | [21-10-14 BD](https://www.english-corpora.org/corona/x4.asp?rs=&t=53242924&ID=21970540527) | [bdnews24.com](https://www.english-corpora.org/corona/x3.asp?node=&p=7&w10=coronavirus&w11=vaccination&r=)[(1)](https://www.english-corpora.org/corona/duplicates1.asp?xx=692&n=1) |  |  |  | COVID vaccines for children this week The government will roll out a **coronavirus** **vaccination** programme for children aged between 12 to 17 years this week. # Children will |
| [694](https://www.english-corpora.org/corona/x4.asp?rs=&t=43525853&ID=21934825693) | [21-10-11 SG](https://www.english-corpora.org/corona/x4.asp?rs=&t=43525853&ID=21934825693) | [sg.news.yahoo.com](https://www.english-corpora.org/corona/x3.asp?node=&p=7&w10=coronavirus&w11=vaccination&r=) |  |  |  | not restaurants, bars or shopping malls. Brazil had a late start with **coronavirus** **vaccination** but is now the country with the fourth-most doses administered. # Israeli archaeologists uncover |
| [695](https://www.english-corpora.org/corona/x4.asp?rs=&t=73535196&ID=21908943681) | [21-10-08 PH](https://www.english-corpora.org/corona/x4.asp?rs=&t=73535196&ID=21908943681) | [spin.ph](https://www.english-corpora.org/corona/x3.asp?node=&p=7&w10=coronavirus&w11=vaccination&r=) |  |  |  | after a ruling that their practice facility doesn't fall under a New York **coronavirus** **vaccination** mandate. # The Nets were told Friday (Saturday, Manila time) that |
| [696](https://www.english-corpora.org/corona/x4.asp?rs=&t=73562438&ID=21987040097) | [21-10-15 PH](https://www.english-corpora.org/corona/x4.asp?rs=&t=73562438&ID=21987040097) | [bworldonline.com](https://www.english-corpora.org/corona/x3.asp?node=&p=7&w10=coronavirus&w11=vaccination&r=) |  |  |  | has set aside? 800,000 (over P47 million) to fund local government **coronavirus** **vaccination** programs in Mindanao. # The humanitarian aid will support organizations on the ground start |
| [697](https://www.english-corpora.org/corona/x4.asp?rs=&t=73566476&ID=21999465437) | [21-10-16 PH](https://www.english-corpora.org/corona/x4.asp?rs=&t=73566476&ID=21999465437) | [sunstar.com.ph](https://www.english-corpora.org/corona/x3.asp?node=&p=7&w10=coronavirus&w11=vaccination&r=) |  |  |  | . # The lending agency called for greater efforts from wealthy nations to boost **coronavirus** **vaccination** rates in poorer countries, while also urging the Federal Reserve and other central banks |
| [698](https://www.english-corpora.org/corona/x4.asp?rs=&t=73578511&ID=22027407674) | [21-10-19 PH](https://www.english-corpora.org/corona/x4.asp?rs=&t=73578511&ID=22027407674) | [news.abs-cbn.com](https://www.english-corpora.org/corona/x3.asp?node=&p=7&w10=coronavirus&w11=vaccination&r=) |  |  |  | refusing to show her " Green Pass ", which offers proof of a **coronavirus** **vaccination**, negative test or recent recovery from Covid-19. # She had been due to |
| [699](https://www.english-corpora.org/corona/x4.asp?rs=&t=53249628&ID=21983049465) | [21-10-15 NG](https://www.english-corpora.org/corona/x4.asp?rs=&t=53249628&ID=21983049465) | [signalng.com](https://www.english-corpora.org/corona/x3.asp?node=&p=7&w10=coronavirus&w11=vaccination&r=) |  |  |  | the world. " # This comes two days after the Federal Government mandated **coronavirus** **vaccination** for civil servants. # The Chairman of the Presidential Steering Committee on COVID-19, |
| [700](https://www.english-corpora.org/corona/x4.asp?rs=&t=88185632&ID=22128185241) | [21-10-29 --](https://www.english-corpora.org/corona/x4.asp?rs=&t=88185632&ID=22128185241) | [The Punch Newspapers](https://www.english-corpora.org/corona/x3.asp?node=&p=7&w10=coronavirus&w11=vaccination&r=) |  |  |  | " Read Also # This comes two days after the Federal Government mandated **coronavirus** **vaccination** for civil servants. # The Chairman of the Presidential Steering Committee on COVID-19, |

| PAGE:   [**<<**](https://www.english-corpora.org/corona/x3.asp?node=&p=1&w10=coronavirus&w11=vaccination&r=) [**<**](https://www.english-corpora.org/corona/x3.asp?node=&p=6&w10=coronavirus&w11=vaccination&r=)   7 / 42  [**>**](https://www.english-corpora.org/corona/x3.asp?node=&p=8&w10=coronavirus&w11=vaccination&r=) [**>>**](https://www.english-corpora.org/corona/x3.asp?node=&p=42&w10=coronavirus&w11=vaccination&r=) |
| --- |

Конец формы

| FIND SAMPLE:  [100](https://www.english-corpora.org/corona/x3.asp?node=&sample=100&w10=coronavirus&w11=vaccination&r=)  [200](https://www.english-corpora.org/corona/x3.asp?node=&sample=200&w10=coronavirus&w11=vaccination&r=)  [500](https://www.english-corpora.org/corona/x3.asp?node=&sample=500&w10=coronavirus&w11=vaccination&r=)  [1000](https://www.english-corpora.org/corona/x3.asp?node=&sample=1000&w10=coronavirus&w11=vaccination&r=) PAGE:   [**<<**](https://www.english-corpora.org/corona/x3.asp?node=&p=1&w10=coronavirus&w11=vaccination&r=) [**<**](https://www.english-corpora.org/corona/x3.asp?node=&p=7&w10=coronavirus&w11=vaccination&r=)   8 / 42  [**>**](https://www.english-corpora.org/corona/x3.asp?node=&p=9&w10=coronavirus&w11=vaccination&r=) [**>>**](https://www.english-corpora.org/corona/x3.asp?node=&p=42&w10=coronavirus&w11=vaccination&r=) |  |
| --- | --- |

Начало формы

| CLICK FOR MORE CONTEXT | [**HELP**](javascript:newFeatures()) | [SAVE](javascript:chooser('s'))    [TRANSLATE](javascript:chooser('t'))    [ANALYZE](javascript:chooser('p')) |
| --- | --- | --- |

| [701](https://www.english-corpora.org/corona/x4.asp?rs=&t=33259108&ID=21741232302) | [21-09-23 US](https://www.english-corpora.org/corona/x4.asp?rs=&t=33259108&ID=21741232302) | [sfgate.com](https://www.english-corpora.org/corona/x3.asp?node=&p=8&w10=coronavirus&w11=vaccination&r=) |  |  |  | to discriminate based on race or ethnicity. # The idea of factoring in **coronavirus** **vaccination** status has drawn particular backlash from the public. A critical care task force in |
| --- | --- | --- | --- | --- | --- | --- |
| [702](https://www.english-corpora.org/corona/x4.asp?rs=&t=43465189&ID=21758364585) | [21-09-24 US](https://www.english-corpora.org/corona/x4.asp?rs=&t=43465189&ID=21758364585) | [wsbtv.com](https://www.english-corpora.org/corona/x3.asp?node=&p=8&w10=coronavirus&w11=vaccination&r=) |  |  |  | continued to surge, leading to record-high hospitalization rates. And the state's **coronavirus** **vaccination** rates remain among the lowest in the nation, with only about 51% of eligible |
| [703](https://www.english-corpora.org/corona/x4.asp?rs=&t=73507763&ID=21820521045) | [21-09-30 US](https://www.english-corpora.org/corona/x4.asp?rs=&t=73507763&ID=21820521045) | [theweek.com](https://www.english-corpora.org/corona/x3.asp?node=&p=8&w10=coronavirus&w11=vaccination&r=) |  |  |  | by 3.6 percent in July compared to a year earlier, driven by rising **coronavirus** **vaccination** rates and nearly $2.8 trillion in federal spending approved since December. The price increases |
| [704](https://www.english-corpora.org/corona/x4.asp?rs=&t=87983425&ID=21736898472) | [21-09-22 US](https://www.english-corpora.org/corona/x4.asp?rs=&t=87983425&ID=21736898472) | [SFGate](https://www.english-corpora.org/corona/x3.asp?node=&p=8&w10=coronavirus&w11=vaccination&r=) |  |  |  | to meet again Tuesday. # # CHARLESTON, W.Va. -- West Virginia's **coronavirus** **vaccination** figures have been adjusted sharply lower after officials discovered that numbers from a federal contractor |
| [705](https://www.english-corpora.org/corona/x4.asp?rs=&t=87983425&ID=21736898803) | [21-09-22 US](https://www.english-corpora.org/corona/x4.asp?rs=&t=87983425&ID=21736898803) | [SFGate](https://www.english-corpora.org/corona/x3.asp?node=&p=8&w10=coronavirus&w11=vaccination&r=) |  |  |  | appear Wednesday for a virtual court hearing over her arrest for an alleged fake **coronavirus** **vaccination** card that misspelled Moderna as " Maderna. " # The 24-year-old woman was released |
| [706](https://www.english-corpora.org/corona/x4.asp?rs=&t=87985139&ID=21739006769) | [21-09-22 US](https://www.english-corpora.org/corona/x4.asp?rs=&t=87985139&ID=21739006769) | [Chicago Tribune](https://www.english-corpora.org/corona/x3.asp?node=&p=8&w10=coronavirus&w11=vaccination&r=) |  |  |  | in a blog post and pointed out that the White House had missed its **coronavirus** **vaccination** goals. # " Facebook is not the reason this goal was missed, " |
| [707](https://www.english-corpora.org/corona/x4.asp?rs=&t=87988731&ID=21748739512) | [21-09-23 US](https://www.english-corpora.org/corona/x4.asp?rs=&t=87988731&ID=21748739512) | [Fox News](https://www.english-corpora.org/corona/x3.asp?node=&p=8&w10=coronavirus&w11=vaccination&r=) |  |  |  | the New York State Department of Health, speaks during a news conference on **coronavirus** **vaccination** at Suffolk County Community College on Monday, April 12, 2021 in Brentwood, |
| [708](https://www.english-corpora.org/corona/x4.asp?rs=&t=87990346&ID=21751418870) | [21-09-23 US](https://www.english-corpora.org/corona/x4.asp?rs=&t=87990346&ID=21751418870) | [San Francisco Chronicle](https://www.english-corpora.org/corona/x3.asp?node=&p=8&w10=coronavirus&w11=vaccination&r=) |  |  |  | but steady stream, not only at Balboa High but also at three other **coronavirus** **vaccination** clinics that opened in city schools beginning on Sept. 14, after students returned to |
| [709](https://www.english-corpora.org/corona/x4.asp?rs=&t=87993246&ID=21762024227) | [21-09-24 US](https://www.english-corpora.org/corona/x4.asp?rs=&t=87993246&ID=21762024227) | [CNBC](https://www.english-corpora.org/corona/x3.asp?node=&p=8&w10=coronavirus&w11=vaccination&r=) |  |  |  | . # Earlier this year, Biden ordered all federal employees to prove their **coronavirus** **vaccination** status or submit to a series of rigorous safety protocols. Thursday's order will |
| [710](https://www.english-corpora.org/corona/x4.asp?rs=&t=87995111&ID=21759730613) | [21-09-24 US](https://www.english-corpora.org/corona/x4.asp?rs=&t=87995111&ID=21759730613) | [SFGate](https://www.english-corpora.org/corona/x3.asp?node=&p=8&w10=coronavirus&w11=vaccination&r=) |  |  |  | continued to surge, leading to record-high hospitalization rates. And the state's **coronavirus** **vaccination** rates remain among the lowest in the nation, with only about 51% of eligible |
| [711](https://www.english-corpora.org/corona/x4.asp?rs=&t=87999901&ID=21767131156) | [21-09-25 US](https://www.english-corpora.org/corona/x4.asp?rs=&t=87999901&ID=21767131156) | [YAHOO!News](https://www.english-corpora.org/corona/x3.asp?node=&p=8&w10=coronavirus&w11=vaccination&r=) |  |  |  | fascist. " # Even in Idaho, which has one of the lowest **coronavirus** **vaccination** rates in the country, clinics have been gearing up for an onslaught of calls |
| [712](https://www.english-corpora.org/corona/x4.asp?rs=&t=88001134&ID=21768900804) | [21-09-25 US](https://www.english-corpora.org/corona/x4.asp?rs=&t=88001134&ID=21768900804) | [The Motley Fool](https://www.english-corpora.org/corona/x3.asp?node=&p=8&w10=coronavirus&w11=vaccination&r=) |  |  |  | predict exactly how long vaccine revenue growth may last. But we might imagine **coronavirus** **vaccination** becoming much like the flu shot: an annual routine. If that happens, |
| [713](https://www.english-corpora.org/corona/x4.asp?rs=&t=88003223&ID=21777483543) | [21-09-26 US](https://www.english-corpora.org/corona/x4.asp?rs=&t=88003223&ID=21777483543) | [YAHOO!Finance](https://www.english-corpora.org/corona/x3.asp?node=&p=8&w10=coronavirus&w11=vaccination&r=) |  |  |  | the limit imp # Even in Idaho, which has one of the lowest **coronavirus** **vaccination** rates in the country, clinics have been gearing up for an onslaught of calls |
| [715](https://www.english-corpora.org/corona/x4.asp?rs=&t=88005589&ID=21780711790) | [21-09-26 US](https://www.english-corpora.org/corona/x4.asp?rs=&t=88005589&ID=21780711790) | [Washington Post](https://www.english-corpora.org/corona/x3.asp?node=&p=8&w10=coronavirus&w11=vaccination&r=)[(1)](https://www.english-corpora.org/corona/duplicates1.asp?xx=714&n=1) |  |  |  | state fair over a requirement that everyone over age 12 show proof of **coronavirus** **vaccination**, test or exemption to enter. # New Mexico's state fair this year |
| [716](https://www.english-corpora.org/corona/x4.asp?rs=&t=88006865&ID=21779060399) | [21-09-26 US](https://www.english-corpora.org/corona/x4.asp?rs=&t=88006865&ID=21779060399) | [Yahoo](https://www.english-corpora.org/corona/x3.asp?node=&p=8&w10=coronavirus&w11=vaccination&r=) |  |  |  | I can b # Even in Idaho, which has one of the lowest **coronavirus** **vaccination** rates in the country, clinics have been gearing up for an onslaught of calls |
| [719](https://www.english-corpora.org/corona/x4.asp?rs=&t=88012862&ID=21790107788) | [21-09-27 US](https://www.english-corpora.org/corona/x4.asp?rs=&t=88012862&ID=21790107788) | [Forbes](https://www.english-corpora.org/corona/x3.asp?node=&p=8&w10=coronavirus&w11=vaccination&r=)[(2)](https://www.english-corpora.org/corona/duplicates1.asp?xx=718&n=2) |  |  |  | the country have begun firing healthcare workers who refuse to comply with **coronavirus** **vaccination** requirements, triggering concerns among some about possible staffing shortages that could arise as mandates |
| [720](https://www.english-corpora.org/corona/x4.asp?rs=&t=88013702&ID=21798784220) | [21-09-28 US](https://www.english-corpora.org/corona/x4.asp?rs=&t=88013702&ID=21798784220) | [YAHOO!News](https://www.english-corpora.org/corona/x3.asp?node=&p=8&w10=coronavirus&w11=vaccination&r=) |  |  |  | officials to reach African Americans and Latinos is showing the intended results, with **coronavirus** **vaccination** rates for those groups on par with that for white Americans. # North Korea |
| [721](https://www.english-corpora.org/corona/x4.asp?rs=&t=88013978&ID=21802705467) | [21-09-28 US](https://www.english-corpora.org/corona/x4.asp?rs=&t=88013978&ID=21802705467) | [New York Post](https://www.english-corpora.org/corona/x3.asp?node=&p=8&w10=coronavirus&w11=vaccination&r=) |  |  |  | which required all hospital and nursing staff to get their first shot of the **coronavirus** **vaccination** by Sept 27. # Governor Kathy Hochul had mandated hospital staff to get their |
| [722](https://www.english-corpora.org/corona/x4.asp?rs=&t=88017893&ID=21801996573) | [21-09-28 US](https://www.english-corpora.org/corona/x4.asp?rs=&t=88017893&ID=21801996573) | [The Washington Post on MSN.com](https://www.english-corpora.org/corona/x3.asp?node=&p=8&w10=coronavirus&w11=vaccination&r=) |  |  |  | 175 unvaccinated employees were fired for failing to comply with the organization's mandatory **coronavirus** **vaccination** policy, the latest in a series of health-care dismissals over coronavirus immunization. # |
| [723](https://www.english-corpora.org/corona/x4.asp?rs=&t=88021650&ID=21814210216) | [21-09-29 US](https://www.english-corpora.org/corona/x4.asp?rs=&t=88021650&ID=21814210216) | [New York Times](https://www.english-corpora.org/corona/x3.asp?node=&p=8&w10=coronavirus&w11=vaccination&r=) |  |  |  | . United Airlines is firing about 600 employees for refusing to comply with its **coronavirus** **vaccination** requirement. Nearly all -- 99 percent -- of its U.S. work force has been |
| [724](https://www.english-corpora.org/corona/x4.asp?rs=&t=88021999&ID=21816804117) | [21-09-29 US](https://www.english-corpora.org/corona/x4.asp?rs=&t=88021999&ID=21816804117) | [YAHOO!News](https://www.english-corpora.org/corona/x3.asp?node=&p=8&w10=coronavirus&w11=vaccination&r=) |  |  |  | officials to reach African Americans and Latinos is showing the intended results, with **coronavirus** **vaccination** rates for those groups on par with that for white Americans. # In Alaska |
| [726](https://www.english-corpora.org/corona/x4.asp?rs=&t=88022997&ID=21816518793) | [21-09-29 US](https://www.english-corpora.org/corona/x4.asp?rs=&t=88022997&ID=21816518793) | [SFGate](https://www.english-corpora.org/corona/x3.asp?node=&p=8&w10=coronavirus&w11=vaccination&r=)[(1)](https://www.english-corpora.org/corona/duplicates1.asp?xx=725&n=1) |  |  |  | suit challenging denials of their requests for religious exemptions from the school's **coronavirus** **vaccination** mandate. # The conservative public interest law firm the Thomas More Society filed the |
| [727](https://www.english-corpora.org/corona/x4.asp?rs=&t=88023557&ID=21815212529) | [21-09-29 US](https://www.english-corpora.org/corona/x4.asp?rs=&t=88023557&ID=21815212529) | [Yahoo](https://www.english-corpora.org/corona/x3.asp?node=&p=8&w10=coronavirus&w11=vaccination&r=) |  |  |  | , making it one of the first countries in Latin America to impose a **coronavirus** **vaccination** mandate. Private companies across the country will also be able to mandate vaccination for |
| [728](https://www.english-corpora.org/corona/x4.asp?rs=&t=88024515&ID=21813559728) | [21-09-29 US](https://www.english-corpora.org/corona/x4.asp?rs=&t=88024515&ID=21813559728) | [Detroit Free Press](https://www.english-corpora.org/corona/x3.asp?node=&p=8&w10=coronavirus&w11=vaccination&r=) |  |  |  | Add colds and breakthrough infections, which occur in patients who have had the **coronavirus** **vaccination** and still get sick with the virus, to the difficulty of making a diagnosis |
| [729](https://www.english-corpora.org/corona/x4.asp?rs=&t=88025094&ID=21816329184) | [21-09-29 US](https://www.english-corpora.org/corona/x4.asp?rs=&t=88025094&ID=21816329184) | [New Scientist](https://www.english-corpora.org/corona/x3.asp?node=&p=8&w10=coronavirus&w11=vaccination&r=) |  |  |  | England. Overall, half the respondents said they were willing to have a **coronavirus** **vaccination**, 37 per cent said they were undecided and 13 per cent said they wanted |
| [730](https://www.english-corpora.org/corona/x4.asp?rs=&t=53186099&ID=21797069617) | [21-09-28 CA](https://www.english-corpora.org/corona/x4.asp?rs=&t=53186099&ID=21797069617) | [ca.news.yahoo.com](https://www.english-corpora.org/corona/x3.asp?node=&p=8&w10=coronavirus&w11=vaccination&r=) |  |  |  | also repor # General Motors warned that salaried workers who have not reported their **coronavirus** **vaccination** status by this Friday, October 1, will receive a letter of safety violation |
| [731](https://www.english-corpora.org/corona/x4.asp?rs=&t=73502807&ID=21805957918) | [21-09-29 CA](https://www.english-corpora.org/corona/x4.asp?rs=&t=73502807&ID=21805957918) | [windsorstar.com](https://www.english-corpora.org/corona/x3.asp?node=&p=8&w10=coronavirus&w11=vaccination&r=) |  |  |  | 175 unvaccinated employees were fired for failing to comply with the organization's mandatory **coronavirus** **vaccination** policy, the latest in a series of health care dismissals over coronavirus immunization. |
| [732](https://www.english-corpora.org/corona/x4.asp?rs=&t=43481157&ID=21807118418) | [21-09-29 GB](https://www.english-corpora.org/corona/x4.asp?rs=&t=43481157&ID=21807118418) | [expressandstar.com](https://www.english-corpora.org/corona/x3.asp?node=&p=8&w10=coronavirus&w11=vaccination&r=) |  |  |  | these are not real either. " # A Covid pass shows people's **coronavirus** **vaccination** details, which they may be asked to show if they travel abroad -- or |
| [733](https://www.english-corpora.org/corona/x4.asp?rs=&t=63394312&ID=21804972447) | [21-09-29 IE](https://www.english-corpora.org/corona/x4.asp?rs=&t=63394312&ID=21804972447) | [buzz.ie](https://www.english-corpora.org/corona/x3.asp?node=&p=8&w10=coronavirus&w11=vaccination&r=) |  |  |  | . # The Premier League is considering whether to " reward " clubs whose **coronavirus** **vaccination** rates are high # " According to the data we have, only seven clubs |
| [734](https://www.english-corpora.org/corona/x4.asp?rs=&t=73484686&ID=21759198768) | [21-09-24 AU](https://www.english-corpora.org/corona/x4.asp?rs=&t=73484686&ID=21759198768) | [watoday.com.au](https://www.english-corpora.org/corona/x3.asp?node=&p=8&w10=coronavirus&w11=vaccination&r=) |  |  |  | fail to meet its first dose **coronavirus** **vaccination** target of 80 per cent by this Sunday, as was initially projected, but |
| [735](https://www.english-corpora.org/corona/x4.asp?rs=&t=73495644&ID=21796001670) | [21-09-28 AU](https://www.english-corpora.org/corona/x4.asp?rs=&t=73495644&ID=21796001670) | [bendigoadvertiser.com.au](https://www.english-corpora.org/corona/x3.asp?node=&p=8&w10=coronavirus&w11=vaccination&r=) |  |  |  | Mr Birks said waiting the extra months would also allow time for Victoria's **coronavirus** **vaccination** rate to rise. " Wearing masks between venues is fine but it is worth |
| [736](https://www.english-corpora.org/corona/x4.asp?rs=&t=73495644&ID=21796002104) | [21-09-28 AU](https://www.english-corpora.org/corona/x4.asp?rs=&t=73495644&ID=21796002104) | [bendigoadvertiser.com.au](https://www.english-corpora.org/corona/x3.asp?node=&p=8&w10=coronavirus&w11=vaccination&r=) |  |  |  | Mr Birks said waiting the extra months would also allow time for Victoria's **coronavirus** **vaccination** rate to rise. # " Wearing masks between venues is fine but it is |
| [737](https://www.english-corpora.org/corona/x4.asp?rs=&t=73498341&ID=21796322745) | [21-09-28 AU](https://www.english-corpora.org/corona/x4.asp?rs=&t=73498341&ID=21796322745) | [examiner.com.au](https://www.english-corpora.org/corona/x3.asp?node=&p=8&w10=coronavirus&w11=vaccination&r=) |  |  |  | department boss has defended making nursing home residents a higher **coronavirus** **vaccination** priority than people living in disability care. A royal commission draft report found the |
| [738](https://www.english-corpora.org/corona/x4.asp?rs=&t=73498720&ID=21805358476) | [21-09-29 AU](https://www.english-corpora.org/corona/x4.asp?rs=&t=73498720&ID=21805358476) | [au.news.yahoo.com](https://www.english-corpora.org/corona/x3.asp?node=&p=8&w10=coronavirus&w11=vaccination&r=) |  |  |  | raised after the federal government revealed work was not being done to counter faked **coronavirus** **vaccination** certificates.Top-ranking Services Australia bureaucrats were grilled about security measures for paper and digital proof of |
| [739](https://www.english-corpora.org/corona/x4.asp?rs=&t=73498879&ID=21811961795) | [21-09-29 AU](https://www.english-corpora.org/corona/x4.asp?rs=&t=73498879&ID=21811961795) | [au.news.yahoo.com](https://www.english-corpora.org/corona/x3.asp?node=&p=8&w10=coronavirus&w11=vaccination&r=) |  |  |  | to shore up its quarantine system ahead of international travel resuming when a key **coronavirus** **vaccination** milestone is reached.The Morrison government is planning to lift outbound travel restrictions and has encouraged |
| [740](https://www.english-corpora.org/corona/x4.asp?rs=&t=73504487&ID=21821813335) | [21-09-30 AU](https://www.english-corpora.org/corona/x4.asp?rs=&t=73504487&ID=21821813335) | [brisbanetimes.com.au](https://www.english-corpora.org/corona/x3.asp?node=&p=8&w10=coronavirus&w11=vaccination&r=) |  |  |  | Mr McGuire refused to say whether the Red Union associations were for or against **coronavirus** **vaccination**, instead arguing the associations represented their members' views. # " We have |
| [741](https://www.english-corpora.org/corona/x4.asp?rs=&t=33254989&ID=21746026924) | [21-09-23 NZ](https://www.english-corpora.org/corona/x4.asp?rs=&t=33254989&ID=21746026924) | [radionz.co.nz](https://www.english-corpora.org/corona/x3.asp?node=&p=8&w10=coronavirus&w11=vaccination&r=) |  |  |  | percent are fully protected. # People wait in a queue for their Covid-19 **coronavirus** **vaccination** in Sydney. Photo: AFP # Almost a third of young people aged between |
| [742](https://www.english-corpora.org/corona/x4.asp?rs=&t=33277342&ID=21806896845) | [21-09-29 NZ](https://www.english-corpora.org/corona/x4.asp?rs=&t=33277342&ID=21806896845) | [home.nzcity.co.nz](https://www.english-corpora.org/corona/x3.asp?node=&p=8&w10=coronavirus&w11=vaccination&r=) |  |  |  | to shore up its quarantine system ahead of international travel resuming when a key **coronavirus** **vaccination** milestone is reached. The Morrison government is planning to lift outbound travel restrictions and |
| [743](https://www.english-corpora.org/corona/x4.asp?rs=&t=73477613&ID=21730624220) | [21-09-22 IN](https://www.english-corpora.org/corona/x4.asp?rs=&t=73477613&ID=21730624220) | [economictimes.indiatimes.com](https://www.english-corpora.org/corona/x3.asp?node=&p=8&w10=coronavirus&w11=vaccination&r=) |  |  |  | will seek an emergency loan of $100 million from the World Bank for a **coronavirus** **vaccination** drive, officials said Wednesday, as the country struggles with an acute currency crisis |
| [744](https://www.english-corpora.org/corona/x4.asp?rs=&t=73496230&ID=21794152844) | [21-09-28 IN](https://www.english-corpora.org/corona/x4.asp?rs=&t=73496230&ID=21794152844) | [devdiscourse.com](https://www.english-corpora.org/corona/x3.asp?node=&p=8&w10=coronavirus&w11=vaccination&r=) |  |  |  | , making it one of the first countries in Latin America to impose a **coronavirus** **vaccination** mandate. ASIA-PACIFIC # * China administered about 3.0 million doses of COVID-19 vaccines on |
| [745](https://www.english-corpora.org/corona/x4.asp?rs=&t=53173738&ID=21756428821) | [21-09-24 PK](https://www.english-corpora.org/corona/x4.asp?rs=&t=53173738&ID=21756428821) | [en.dailypakistan.com.pk](https://www.english-corpora.org/corona/x3.asp?node=&p=8&w10=coronavirus&w11=vaccination&r=) |  |  |  | registered against three employees of the hospital involved in the fake entry of the **coronavirus** **vaccination**. Two of those nominated in the FIR have been arrested. # The Federal |
| [746](https://www.english-corpora.org/corona/x4.asp?rs=&t=53180630&ID=21771931171) | [21-09-26 PK](https://www.english-corpora.org/corona/x4.asp?rs=&t=53180630&ID=21771931171) | [tribune.com.pk](https://www.english-corpora.org/corona/x3.asp?node=&p=8&w10=coronavirus&w11=vaccination&r=) |  |  |  | Complaints were being received that the police were arresting passers-by for not having the **coronavirus** **vaccination** card, which was a completely wrong practice, " the spokesperson said, adding |
| [747](https://www.english-corpora.org/corona/x4.asp?rs=&t=53188555&ID=21795421843) | [21-09-28 PK](https://www.english-corpora.org/corona/x4.asp?rs=&t=53188555&ID=21795421843) | [samaa.tv](https://www.english-corpora.org/corona/x3.asp?node=&p=8&w10=coronavirus&w11=vaccination&r=) |  |  |  | can just walk into any centre. Earlier this week, the government made **coronavirus** **vaccination** compulsory for students between the ages 15 and 18. They will be administered the |
| [749](https://www.english-corpora.org/corona/x4.asp?rs=&t=53191630&ID=21804661982) | [21-09-29 PK](https://www.english-corpora.org/corona/x4.asp?rs=&t=53191630&ID=21804661982) | [samaa.tv](https://www.english-corpora.org/corona/x3.asp?node=&p=8&w10=coronavirus&w11=vaccination&r=)[(1)](https://www.english-corpora.org/corona/duplicates1.asp?xx=748&n=1) |  |  |  | Operation Centre has decided to increase restrictions in cities with low levels of **coronavirus** **vaccination**, Federal Planning Minister Asad Umar has announced. " The only way out of |
| [751](https://www.english-corpora.org/corona/x4.asp?rs=&t=53188976&ID=21810819953) | [21-09-29 BD](https://www.english-corpora.org/corona/x4.asp?rs=&t=53188976&ID=21810819953) | [bdnews24.com](https://www.english-corpora.org/corona/x3.asp?node=&p=8&w10=coronavirus&w11=vaccination&r=)[(1)](https://www.english-corpora.org/corona/duplicates1.asp?xx=750&n=1) |  |  |  | beards' and' men's voices are changing' due to the **coronavirus** **vaccination** have made waves on social media. # In one of his lectures, he |
| [752](https://www.english-corpora.org/corona/x4.asp?rs=&t=43477811&ID=21792874115) | [21-09-28 SG](https://www.english-corpora.org/corona/x4.asp?rs=&t=43477811&ID=21792874115) | [sg.news.yahoo.com](https://www.english-corpora.org/corona/x3.asp?node=&p=8&w10=coronavirus&w11=vaccination&r=) |  |  |  | officials to reach African Americans and Latinos is showing the intended results, with **coronavirus** **vaccination** rates for those groups on par with that for white Americans. # Tom McAlpin |
| [753](https://www.english-corpora.org/corona/x4.asp?rs=&t=73485792&ID=21759347225) | [21-09-24 PH](https://www.english-corpora.org/corona/x4.asp?rs=&t=73485792&ID=21759347225) | [bworldonline.com](https://www.english-corpora.org/corona/x3.asp?node=&p=8&w10=coronavirus&w11=vaccination&r=) |  |  |  | as malls, local governments have been tapping various venues and schemes for the **coronavirus** **vaccination** program, including drive-thru, house-to-house service for the elderly, and village-level delivery. |
| [754](https://www.english-corpora.org/corona/x4.asp?rs=&t=63376204&ID=21742876639) | [21-09-23 ZA](https://www.english-corpora.org/corona/x4.asp?rs=&t=63376204&ID=21742876639) | [briefly.co.za](https://www.english-corpora.org/corona/x3.asp?node=&p=8&w10=coronavirus&w11=vaccination&r=) |  |  |  | the Western Cape # In related news, Briefly News previously reported that since **coronavirus** **vaccination** for all adults opened up on Friday, 20 August, health officials have come |
| [755](https://www.english-corpora.org/corona/x4.asp?rs=&t=33284235&ID=21840275690) | [21-10-02 US](https://www.english-corpora.org/corona/x4.asp?rs=&t=33284235&ID=21840275690) | [nytimes.com](https://www.english-corpora.org/corona/x3.asp?node=&p=8&w10=coronavirus&w11=vaccination&r=) |  |  |  | State and federal officials said the booster program would look much different than earlier **coronavirus** **vaccination** drives, which relied heavily on mass inoculation sites at sports stadiums and convention centers |
| [756](https://www.english-corpora.org/corona/x4.asp?rs=&t=33285572&ID=21841894738) | [21-10-02 US](https://www.english-corpora.org/corona/x4.asp?rs=&t=33285572&ID=21841894738) | [ktla.com](https://www.english-corpora.org/corona/x3.asp?node=&p=8&w10=coronavirus&w11=vaccination&r=) |  |  |  | students # California Gov. Gavin Newsom on Friday imposed the nation's first **coronavirus** **vaccination** mandate for schoolchildren. But the mandate won't take effect immediately and won't |
| [757](https://www.english-corpora.org/corona/x4.asp?rs=&t=33286211&ID=21838974000) | [21-10-02 US](https://www.english-corpora.org/corona/x4.asp?rs=&t=33286211&ID=21838974000) | [medicalxpress.com](https://www.english-corpora.org/corona/x3.asp?node=&p=8&w10=coronavirus&w11=vaccination&r=) |  |  |  | Cancer patients are at increased risk of SARS-CoV-2 infection and severe COVID-19 courses. **Coronavirus** **vaccination** is generally recommended for cancer patients, but so far little is known about how |
| [758](https://www.english-corpora.org/corona/x4.asp?rs=&t=43493185&ID=21832411297) | [21-10-01 US](https://www.english-corpora.org/corona/x4.asp?rs=&t=43493185&ID=21832411297) | [sacramento.cbslocal.com](https://www.english-corpora.org/corona/x3.asp?node=&p=8&w10=coronavirus&w11=vaccination&r=) |  |  |  | ) -- California Gov. Gavin Newsom on Friday announced the nation's first **coronavirus** **vaccination** mandate for schoolchildren, a plan that will have all elementary through high school students |
| [759](https://www.english-corpora.org/corona/x4.asp?rs=&t=53204712&ID=21839538290) | [21-10-02 US](https://www.english-corpora.org/corona/x4.asp?rs=&t=53204712&ID=21839538290) | [ksby.com](https://www.english-corpora.org/corona/x3.asp?node=&p=8&w10=coronavirus&w11=vaccination&r=) |  |  |  | updated2021-10-02 11:31:10-04 # California Gov. Gavin Newsom is imposing the nation's first **coronavirus** **vaccination** mandate for schoolchildren. But the mandate he announced Friday won't take effect immediately |
| [760](https://www.english-corpora.org/corona/x4.asp?rs=&t=88032021&ID=21833787157) | [21-10-01 US](https://www.english-corpora.org/corona/x4.asp?rs=&t=88032021&ID=21833787157) | [YAHOO!News](https://www.english-corpora.org/corona/x3.asp?node=&p=8&w10=coronavirus&w11=vaccination&r=) |  |  |  | . # California Gov. Gavin Newsom on Friday announced the nation's first **coronavirus** **vaccination** mandate for schoolchildren, a plan that will have all elementary through high school students |
| [762](https://www.english-corpora.org/corona/x4.asp?rs=&t=88033530&ID=21833873627) | [21-10-01 US](https://www.english-corpora.org/corona/x4.asp?rs=&t=88033530&ID=21833873627) | [The Guardian on MSN.com](https://www.english-corpora.org/corona/x3.asp?node=&p=8&w10=coronavirus&w11=vaccination&r=)[(1)](https://www.english-corpora.org/corona/duplicates1.asp?xx=761&n=1) |  |  |  | . # In the US, California has announced the nation's first **coronavirus** **vaccination** mandate for schoolchildren. The plan, announced by Governor Gavin Newsom, will see |
| [764](https://www.english-corpora.org/corona/x4.asp?rs=&t=88033530&ID=21833875900) | [21-10-01 US](https://www.english-corpora.org/corona/x4.asp?rs=&t=88033530&ID=21833875900) | [The Guardian on MSN.com](https://www.english-corpora.org/corona/x3.asp?node=&p=8&w10=coronavirus&w11=vaccination&r=)[(1)](https://www.english-corpora.org/corona/duplicates1.asp?xx=763&n=1) |  |  |  | # In the United States, California has announced the nation's first **coronavirus** **vaccination** mandate for schoolchildren. # The plan, announced by Governor Gavin Newsom, will |
| [765](https://www.english-corpora.org/corona/x4.asp?rs=&t=88033652&ID=21835665414) | [21-10-01 US](https://www.english-corpora.org/corona/x4.asp?rs=&t=88033652&ID=21835665414) | [Associated Press](https://www.english-corpora.org/corona/x3.asp?node=&p=8&w10=coronavirus&w11=vaccination&r=) |  |  |  | ) # California Gov. Gavin Newsom on Friday imposed the nation's first **coronavirus** **vaccination** mandate for schoolchildren. But the mandate won't take effect immediately and won't |
| [766](https://www.english-corpora.org/corona/x4.asp?rs=&t=88036792&ID=21837336235) | [21-10-01 US](https://www.english-corpora.org/corona/x4.asp?rs=&t=88036792&ID=21837336235) | [The Washington Post on MSN.com](https://www.english-corpora.org/corona/x3.asp?node=&p=8&w10=coronavirus&w11=vaccination&r=) |  |  |  | attempt to block New York City's requirement that public school teachers receive a **coronavirus** **vaccination**. # Justice Sonia Sotomayor, the justice charged with handling emergency requests from that |
| [767](https://www.english-corpora.org/corona/x4.asp?rs=&t=88042006&ID=21843940033) | [21-10-02 US](https://www.english-corpora.org/corona/x4.asp?rs=&t=88042006&ID=21843940033) | [New York Times](https://www.english-corpora.org/corona/x3.asp?node=&p=8&w10=coronavirus&w11=vaccination&r=) |  |  |  | the government announced Thursday. It is the first country in Europe to require **coronavirus** **vaccination** certificates so widely. # " It's an extraordinary endeavor, " Italy's |
| [769](https://www.english-corpora.org/corona/x4.asp?rs=&t=88042006&ID=21843940732) | [21-10-02 US](https://www.english-corpora.org/corona/x4.asp?rs=&t=88042006&ID=21843940732) | [New York Times](https://www.english-corpora.org/corona/x3.asp?node=&p=8&w10=coronavirus&w11=vaccination&r=)[(1)](https://www.english-corpora.org/corona/duplicates1.asp?xx=768&n=1) |  |  |  | version of this item misstated the age group included in two statistics on **coronavirus** **vaccination** in Italy. The country reports that it has administered at least one dose of |
| [771](https://www.english-corpora.org/corona/x4.asp?rs=&t=88046227&ID=21853917849) | [21-10-03 US](https://www.english-corpora.org/corona/x4.asp?rs=&t=88046227&ID=21853917849) | [New York Times](https://www.english-corpora.org/corona/x3.asp?node=&p=8&w10=coronavirus&w11=vaccination&r=)[(1)](https://www.english-corpora.org/corona/duplicates1.asp?xx=770&n=1) |  |  |  | vaccinated. # JERUSALEM -- Israel on Sunday introduced new rules for determining **coronavirus** **vaccination** status, making a booster shot a requirement for full inoculation and vaccination passports. |
| [772](https://www.english-corpora.org/corona/x4.asp?rs=&t=88046365&ID=21854909503) | [21-10-03 US](https://www.english-corpora.org/corona/x4.asp?rs=&t=88046365&ID=21854909503) | [CNN](https://www.english-corpora.org/corona/x3.asp?node=&p=8&w10=coronavirus&w11=vaccination&r=) |  |  |  | foreign nationals must have **coronavirus** **vaccination** to enter country from November # (CNN) -- All foreign nationals entering New |
| [773](https://www.english-corpora.org/corona/x4.asp?rs=&t=88046365&ID=21854909503) | [21-10-03 US](https://www.english-corpora.org/corona/x4.asp?rs=&t=88046365&ID=21854909503) | [CNN](https://www.english-corpora.org/corona/x3.asp?node=&p=8&w10=coronavirus&w11=vaccination&r=) |  |  |  | foreign nationals must have **coronavirus** **vaccination** to enter country from November # (CNN) -- All foreign nationals entering New |
| [774](https://www.english-corpora.org/corona/x4.asp?rs=&t=88050934&ID=21865376107) | [21-10-04 US](https://www.english-corpora.org/corona/x4.asp?rs=&t=88050934&ID=21865376107) | [The Guardian on MSN.com](https://www.english-corpora.org/corona/x3.asp?node=&p=8&w10=coronavirus&w11=vaccination&r=) |  |  |  | as long as they meet strict safeguards and have at least had their first **coronavirus** **vaccination**. # The industry-wide mandate, and other restrictions, sparked a protest outside the |
| [775](https://www.english-corpora.org/corona/x4.asp?rs=&t=88053397&ID=21867201287) | [21-10-04 US](https://www.english-corpora.org/corona/x4.asp?rs=&t=88053397&ID=21867201287) | [Yahoo](https://www.english-corpora.org/corona/x3.asp?node=&p=8&w10=coronavirus&w11=vaccination&r=) |  |  |  | a nurse at a Michigan Veterans Affairs hospital with stealing authentic **coronavirus** **vaccination** cards, along with vaccine lot numbers required to make the cards appear legitimate. |
| [776](https://www.english-corpora.org/corona/x4.asp?rs=&t=88053397&ID=21867201494) | [21-10-04 US](https://www.english-corpora.org/corona/x4.asp?rs=&t=88053397&ID=21867201494) | [Yahoo](https://www.english-corpora.org/corona/x3.asp?node=&p=8&w10=coronavirus&w11=vaccination&r=) |  |  |  | about coronavirus booster shots # Federal authorities have since charged Kierczak with stealing authentic **coronavirus** **vaccination** cards from the VA hospital - along with vaccine lot numbers required to make the |
| [777](https://www.english-corpora.org/corona/x4.asp?rs=&t=88054443&ID=21875055664) | [21-10-05 US](https://www.english-corpora.org/corona/x4.asp?rs=&t=88054443&ID=21875055664) | [Tulsa World](https://www.english-corpora.org/corona/x3.asp?node=&p=8&w10=coronavirus&w11=vaccination&r=) |  |  |  | man rests after receiving his second dose of the Pfizer COVID-19 vaccine at a **coronavirus** **vaccination** center set up at a synagogue in Bnei Brak, Israel, Sunday, March |
| [779](https://www.english-corpora.org/corona/x4.asp?rs=&t=88054725&ID=21874036413) | [21-10-05 US](https://www.english-corpora.org/corona/x4.asp?rs=&t=88054725&ID=21874036413) | [Yahoo! Sports](https://www.english-corpora.org/corona/x3.asp?node=&p=8&w10=coronavirus&w11=vaccination&r=)[(1)](https://www.english-corpora.org/corona/duplicates1.asp?xx=778&n=1) |  |  |  | year to year. But ever since Conway Regional Health System instituted a **coronavirus** **vaccination** mandate for its employees, he's been devoting a chunk of his workday to |
| [781](https://www.english-corpora.org/corona/x4.asp?rs=&t=53198623&ID=21828127010) | [21-10-01 CA](https://www.english-corpora.org/corona/x4.asp?rs=&t=53198623&ID=21828127010) | [kelownadailycourier.ca](https://www.english-corpora.org/corona/x3.asp?node=&p=8&w10=coronavirus&w11=vaccination&r=)[(1)](https://www.english-corpora.org/corona/duplicates1.asp?xx=780&n=1) |  |  |  | -- California Gov. Gavin Newsom on Friday announced the nation's first **coronavirus** **vaccination** mandate for schoolchildren, requiring that all elementary through high school students get the shots |
| [782](https://www.english-corpora.org/corona/x4.asp?rs=&t=73518460&ID=21860874549) | [21-10-04 AU](https://www.english-corpora.org/corona/x4.asp?rs=&t=73518460&ID=21860874549) | [thenewdaily.com.au](https://www.english-corpora.org/corona/x3.asp?node=&p=8&w10=coronavirus&w11=vaccination&r=) |  |  |  | as long as they meet strict safeguards and have at least had their first **coronavirus** **vaccination**. # The industry-wide mandate, and other restrictions brought in to curb transmission, |
| [783](https://www.english-corpora.org/corona/x4.asp?rs=&t=73518987&ID=21872361578) | [21-10-05 AU](https://www.english-corpora.org/corona/x4.asp?rs=&t=73518987&ID=21872361578) | [canberratimes.com.au](https://www.english-corpora.org/corona/x3.asp?node=&p=8&w10=coronavirus&w11=vaccination&r=) |  |  |  | : * Australia has broken through 80 per cent first-dose **coronavirus** **vaccination** coverage, with eight in 10 Australians aged 16 and above having received at least |
| [785](https://www.english-corpora.org/corona/x4.asp?rs=&t=73519088&ID=21872374640) | [21-10-05 AU](https://www.english-corpora.org/corona/x4.asp?rs=&t=73519088&ID=21872374640) | [thenewdaily.com.au](https://www.english-corpora.org/corona/x3.asp?node=&p=8&w10=coronavirus&w11=vaccination&r=)[(1)](https://www.english-corpora.org/corona/duplicates1.asp?xx=784&n=1) |  |  |  | 19-31. # Elsewhere, Australia has broken through 80 per cent first-dose **coronavirus** **vaccination** coverage. More than 57 per cent of over-16s have received both doses. |
| [786](https://www.english-corpora.org/corona/x4.asp?rs=&t=73519806&ID=21868716962) | [21-10-05 AU](https://www.english-corpora.org/corona/x4.asp?rs=&t=73519806&ID=21868716962) | [mamamia.com.au](https://www.english-corpora.org/corona/x3.asp?node=&p=8&w10=coronavirus&w11=vaccination&r=) |  |  |  | as long as they meet strict safeguards and have at least had their first **coronavirus** **vaccination**. # The industry-wide mandate, and other restrictions brought in to curb transmission, |
| [787](https://www.english-corpora.org/corona/x4.asp?rs=&t=33288325&ID=21846575952) | [21-10-03 NZ](https://www.english-corpora.org/corona/x4.asp?rs=&t=33288325&ID=21846575952) | [sunlive.co.nz](https://www.english-corpora.org/corona/x3.asp?node=&p=8&w10=coronavirus&w11=vaccination&r=) |  |  |  | citizens to come and go without seeking permission as states close in on key **coronavirus** **vaccination** targets. # Drugmaker Merck says its experimental Covid-19 pill reduced hospitalisations and deaths by |
| [788](https://www.english-corpora.org/corona/x4.asp?rs=&t=33288762&ID=21858800808) | [21-10-04 NZ](https://www.english-corpora.org/corona/x4.asp?rs=&t=33288762&ID=21858800808) | [nz.news.yahoo.com](https://www.english-corpora.org/corona/x3.asp?node=&p=8&w10=coronavirus&w11=vaccination&r=) |  |  |  | # LATEST COVID-19 DEVELOPMENTS: * Australia has broken through 80 per cent first-dose **coronavirus** **vaccination** coverage, with eight in 10 Australians aged 16 and above having received at least |
| [792](https://www.english-corpora.org/corona/x4.asp?rs=&t=73520653&ID=21870782741) | [21-10-05 IN](https://www.english-corpora.org/corona/x4.asp?rs=&t=73520653&ID=21870782741) | [economictimes.indiatimes.com](https://www.english-corpora.org/corona/x3.asp?node=&p=8&w10=coronavirus&w11=vaccination&r=)[(3)](https://www.english-corpora.org/corona/duplicates1.asp?xx=791&n=3) |  |  |  | . # According to a report by TOI, dry runs of the **coronavirus** **vaccination** programme started on Monday in four states, a day after India recorded its lowest |
| [793](https://www.english-corpora.org/corona/x4.asp?rs=&t=53211148&ID=21860341345) | [21-10-04 PK](https://www.english-corpora.org/corona/x4.asp?rs=&t=53211148&ID=21860341345) | [samaa.tv](https://www.english-corpora.org/corona/x3.asp?node=&p=8&w10=coronavirus&w11=vaccination&r=) |  |  |  | has decided to relax restrictions in eight cities across Pakistan with high rates of **coronavirus** **vaccination**. These include Islamabad, Peshawar, Skardu, Gilgit, Rawalpindi, Quetta, |
| [794](https://www.english-corpora.org/corona/x4.asp?rs=&t=53211148&ID=21860341554) | [21-10-04 PK](https://www.english-corpora.org/corona/x4.asp?rs=&t=53211148&ID=21860341554) | [samaa.tv](https://www.english-corpora.org/corona/x3.asp?node=&p=8&w10=coronavirus&w11=vaccination&r=) |  |  |  | has decided to relax restrictions in eight cities across Pakistan with high rates of **coronavirus** **vaccination**. # These include Islamabad, Peshawar, Skardu, Gilgit, Rawalpindi, Quetta |
| [795](https://www.english-corpora.org/corona/x4.asp?rs=&t=88037350&ID=21835547700) | [21-10-01 SG](https://www.english-corpora.org/corona/x4.asp?rs=&t=88037350&ID=21835547700) | [The Straits Times](https://www.english-corpora.org/corona/x3.asp?node=&p=8&w10=coronavirus&w11=vaccination&r=) |  |  |  | start plagued by much controversy that still haunts President Jair Bolsonaro, Brazil's **coronavirus** **vaccination** campaign is now one of the fastest-paced and farthest-reaching in the world. # While |
| [796](https://www.english-corpora.org/corona/x4.asp?rs=&t=88037350&ID=21835547807) | [21-10-01 SG](https://www.english-corpora.org/corona/x4.asp?rs=&t=88037350&ID=21835547807) | [The Straits Times](https://www.english-corpora.org/corona/x3.asp?node=&p=8&w10=coronavirus&w11=vaccination&r=) |  |  |  | death toll - more than 588,000 fatalities reported to date - has seen its **coronavirus** **vaccination** rate pick up and its death rate tumble as imported jabs started arriving and local |
| [797](https://www.english-corpora.org/corona/x4.asp?rs=&t=43501102&ID=21857730795) | [21-10-04 HK](https://www.english-corpora.org/corona/x4.asp?rs=&t=43501102&ID=21857730795) | [hongkongfp.com](https://www.english-corpora.org/corona/x3.asp?node=&p=8&w10=coronavirus&w11=vaccination&r=) |  |  |  | # Hong Kong health authorities have ejected a private clinic from the city's **coronavirus** **vaccination** programme after it reportedly recommended the German-made Pfizer/BioNTech shot to patients over the one from |
| [799](https://www.english-corpora.org/corona/x4.asp?rs=&t=88033123&ID=21834179867) | [21-10-01 --](https://www.english-corpora.org/corona/x4.asp?rs=&t=88033123&ID=21834179867) | [France 24](https://www.english-corpora.org/corona/x3.asp?node=&p=8&w10=coronavirus&w11=vaccination&r=)[(1)](https://www.english-corpora.org/corona/duplicates1.asp?xx=798&n=1) |  |  |  | plagued by much controversy that still haunts President Jair Bolsonaro, Brazil's **coronavirus** **vaccination** campaign is now one of the fastest-paced and farthest-reaching in the world. # While |
| [800](https://www.english-corpora.org/corona/x4.asp?rs=&t=88033123&ID=21834179974) | [21-10-01 --](https://www.english-corpora.org/corona/x4.asp?rs=&t=88033123&ID=21834179974) | [France 24](https://www.english-corpora.org/corona/x3.asp?node=&p=8&w10=coronavirus&w11=vaccination&r=) |  |  |  | death toll -- more than 588,000 fatalities reported to date -- has seen its **coronavirus** **vaccination** rate pick up and its death rate tumble as imported jabs started arriving and local |

| PAGE:   [**<<**](https://www.english-corpora.org/corona/x3.asp?node=&p=1&w10=coronavirus&w11=vaccination&r=) [**<**](https://www.english-corpora.org/corona/x3.asp?node=&p=7&w10=coronavirus&w11=vaccination&r=)   8 / 42  [**>**](https://www.english-corpora.org/corona/x3.asp?node=&p=9&w10=coronavirus&w11=vaccination&r=) [**>>**](https://www.english-corpora.org/corona/x3.asp?node=&p=42&w10=coronavirus&w11=vaccination&r=) |
| --- |

Конец формы

| FIND SAMPLE:  [100](https://www.english-corpora.org/corona/x3.asp?node=&sample=100&w10=coronavirus&w11=vaccination&r=)  [200](https://www.english-corpora.org/corona/x3.asp?node=&sample=200&w10=coronavirus&w11=vaccination&r=)  [500](https://www.english-corpora.org/corona/x3.asp?node=&sample=500&w10=coronavirus&w11=vaccination&r=)  [1000](https://www.english-corpora.org/corona/x3.asp?node=&sample=1000&w10=coronavirus&w11=vaccination&r=) PAGE:   [**<<**](https://www.english-corpora.org/corona/x3.asp?node=&p=1&w10=coronavirus&w11=vaccination&r=) [**<**](https://www.english-corpora.org/corona/x3.asp?node=&p=8&w10=coronavirus&w11=vaccination&r=)   9 / 42  [**>**](https://www.english-corpora.org/corona/x3.asp?node=&p=10&w10=coronavirus&w11=vaccination&r=) [**>>**](https://www.english-corpora.org/corona/x3.asp?node=&p=42&w10=coronavirus&w11=vaccination&r=) |  |
| --- | --- |

Начало формы

| CLICK FOR MORE CONTEXT | [**HELP**](javascript:newFeatures()) | [SAVE](javascript:chooser('s'))    [TRANSLATE](javascript:chooser('t'))    [ANALYZE](javascript:chooser('p')) |
| --- | --- | --- |

| [801](https://www.english-corpora.org/corona/x4.asp?rs=&t=33209863&ID=21584211485) | [21-09-08 US](https://www.english-corpora.org/corona/x4.asp?rs=&t=33209863&ID=21584211485) | [staradvertiser.com](https://www.english-corpora.org/corona/x3.asp?node=&p=9&w10=coronavirus&w11=vaccination&r=) |  |  |  | # A man received a third Pfizer-BioNTech COVID-19 vaccine from medical staff at a **coronavirus** **vaccination** center in Tel Aviv, Israel, Aug. 10. # Israel, once a |
| --- | --- | --- | --- | --- | --- | --- |
| [802](https://www.english-corpora.org/corona/x4.asp?rs=&t=33215161&ID=21617835985) | [21-09-11 US](https://www.english-corpora.org/corona/x4.asp?rs=&t=33215161&ID=21617835985) | [cnbc.com](https://www.english-corpora.org/corona/x3.asp?node=&p=9&w10=coronavirus&w11=vaccination&r=) |  |  |  | # Long queues of people waiting to get coronavirus vaccine at new pop-up walk-in **coronavirus** **vaccination** clinic at the Peanut Farm in St Kilda, Melbourne, Victoria, Australia in |
| [803](https://www.english-corpora.org/corona/x4.asp?rs=&t=33216462&ID=21618032349) | [21-09-11 US](https://www.english-corpora.org/corona/x4.asp?rs=&t=33216462&ID=21618032349) | [aljazeera.com](https://www.english-corpora.org/corona/x3.asp?node=&p=9&w10=coronavirus&w11=vaccination&r=) |  |  |  | visitors over the course of six months, won't require visitors to present **coronavirus** **vaccination** certificates or COVID-19 tests, a spokesperson tells Bloomberg News. # Visitors to Dubai |
| [804](https://www.english-corpora.org/corona/x4.asp?rs=&t=33219829&ID=21616126804) | [21-09-11 US](https://www.english-corpora.org/corona/x4.asp?rs=&t=33219829&ID=21616126804) | [eastbaytimes.com](https://www.english-corpora.org/corona/x3.asp?node=&p=9&w10=coronavirus&w11=vaccination&r=) |  |  |  | 500,000 doses of the Pfizer vaccine from Denmark as it tries to keep its **coronavirus** **vaccination** program running at full speed, the government said. # The doses are on |
| [805](https://www.english-corpora.org/corona/x4.asp?rs=&t=33226319&ID=21644327792) | [21-09-14 US](https://www.english-corpora.org/corona/x4.asp?rs=&t=33226319&ID=21644327792) | [nytimes.com](https://www.english-corpora.org/corona/x3.asp?node=&p=9&w10=coronavirus&w11=vaccination&r=) |  |  |  | -- As recently as late August, Hai Shoulian, a prominent opponent of **coronavirus** **vaccination** policies in Israel, was demonstrating in Jerusalem and describing the country's electronic pass |
| [806](https://www.english-corpora.org/corona/x4.asp?rs=&t=33230854&ID=21657629127) | [21-09-15 US](https://www.english-corpora.org/corona/x4.asp?rs=&t=33230854&ID=21657629127) | [thehill.com](https://www.english-corpora.org/corona/x3.asp?node=&p=9&w10=coronavirus&w11=vaccination&r=) |  |  |  | . # The poll comes as more venues are starting to require proof of **coronavirus** **vaccination** in order to enter. # Two NFL teams have said fans must be vaccinated |
| [807](https://www.english-corpora.org/corona/x4.asp?rs=&t=33231192&ID=21659927626) | [21-09-15 US](https://www.english-corpora.org/corona/x4.asp?rs=&t=33231192&ID=21659927626) | [gizmodo.com](https://www.english-corpora.org/corona/x3.asp?node=&p=9&w10=coronavirus&w11=vaccination&r=) |  |  |  | weird for several reasons. First, on Tuesday, the Biden administration issued **coronavirus** **vaccination** requirements for immigrants seeking to become " lawful permanent residents, " joining a long |
| [808](https://www.english-corpora.org/corona/x4.asp?rs=&t=33233654&ID=21668383804) | [21-09-16 US](https://www.english-corpora.org/corona/x4.asp?rs=&t=33233654&ID=21668383804) | [nytimes.com](https://www.english-corpora.org/corona/x3.asp?node=&p=9&w10=coronavirus&w11=vaccination&r=) |  |  |  | ... Damian Dovarganes/Associated Press # Los Angeles County next month will require proof of **coronavirus** **vaccination** to enter bars, nightclubs and other drinking establishments, as it joins the list |
| [809](https://www.english-corpora.org/corona/x4.asp?rs=&t=33233654&ID=21668388601) | [21-09-16 US](https://www.english-corpora.org/corona/x4.asp?rs=&t=33233654&ID=21668388601) | [nytimes.com](https://www.english-corpora.org/corona/x3.asp?node=&p=9&w10=coronavirus&w11=vaccination&r=) |  |  |  | France, last week.Credit... Jeff Pachoud/Agence France-Presse -- Getty Images # As a **coronavirus** **vaccination** mandate for health workers went into effect in France on Wednesday, government officials said |
| [810](https://www.english-corpora.org/corona/x4.asp?rs=&t=33234302&ID=21666436404) | [21-09-16 US](https://www.english-corpora.org/corona/x4.asp?rs=&t=33234302&ID=21666436404) | [pilotonline.com](https://www.english-corpora.org/corona/x3.asp?node=&p=9&w10=coronavirus&w11=vaccination&r=) |  |  |  | incorporating **coronavirus** **vaccination** data of Virginians who received their shots in Maryland into its records Thursday. # |
| [811](https://www.english-corpora.org/corona/x4.asp?rs=&t=33235711&ID=21666650628) | [21-09-16 US](https://www.english-corpora.org/corona/x4.asp?rs=&t=33235711&ID=21666650628) | [wbur.org](https://www.english-corpora.org/corona/x3.asp?node=&p=9&w10=coronavirus&w11=vaccination&r=) |  |  |  | ' Stateline publication. " Our expectation is that the black market for fake **coronavirus** **vaccination** cards will continue to thrive as more policy requiring vaccination proof gets rolled out. |
| [812](https://www.english-corpora.org/corona/x4.asp?rs=&t=33237086&ID=21682778112) | [21-09-17 US](https://www.english-corpora.org/corona/x4.asp?rs=&t=33237086&ID=21682778112) | [tampabay.com](https://www.english-corpora.org/corona/x3.asp?node=&p=9&w10=coronavirus&w11=vaccination&r=) |  |  |  | also offering $500 and two extra days off to employees who submit proof of **coronavirus** **vaccination**, and is requiring all new hires to be vaccinated. # It's not |
| [813](https://www.english-corpora.org/corona/x4.asp?rs=&t=53117766&ID=21571302685) | [21-09-07 US](https://www.english-corpora.org/corona/x4.asp?rs=&t=53117766&ID=21571302685) | [abc7news.com](https://www.english-corpora.org/corona/x3.asp?node=&p=9&w10=coronavirus&w11=vaccination&r=) |  |  |  | , gyms, more # San Francisco has one of the nation's highest **coronavirus** **vaccination** rates at nearly 80-percent, but with the Delta variant, it wants more. |
| [814](https://www.english-corpora.org/corona/x4.asp?rs=&t=53147962&ID=21669463366) | [21-09-16 US](https://www.english-corpora.org/corona/x4.asp?rs=&t=53147962&ID=21669463366) | [boston.com](https://www.english-corpora.org/corona/x3.asp?node=&p=9&w10=coronavirus&w11=vaccination&r=) |  |  |  | are criticizing Gov. Charlie Baker over the state's lack of a statewide **coronavirus** **vaccination** policy for schools. # " A statewide mandate requiring educators to be vaccinated, |
| [815](https://www.english-corpora.org/corona/x4.asp?rs=&t=73442976&ID=21622121604) | [21-09-11 US](https://www.english-corpora.org/corona/x4.asp?rs=&t=73442976&ID=21622121604) | [theweek.com](https://www.english-corpora.org/corona/x3.asp?node=&p=9&w10=coronavirus&w11=vaccination&r=) |  |  |  | and up # The Los Angeles Board of Education on Thursday voted to make **coronavirus** **vaccination** mandatory for all children 12 and older in Los Angeles public schools. The decision |
| [816](https://www.english-corpora.org/corona/x4.asp?rs=&t=87889040&ID=21564304705) | [21-09-06 US](https://www.english-corpora.org/corona/x4.asp?rs=&t=87889040&ID=21564304705) | [New York Times](https://www.english-corpora.org/corona/x3.asp?node=&p=9&w10=coronavirus&w11=vaccination&r=) |  |  |  | individual Christians sincerely (though mistakenly) believe that their religious faith prohibits a **coronavirus** **vaccination**, that still does not justify an exemption. " Sincerity " does not justify |
| [817](https://www.english-corpora.org/corona/x4.asp?rs=&t=87890705&ID=21563612429) | [21-09-06 US](https://www.english-corpora.org/corona/x4.asp?rs=&t=87890705&ID=21563612429) | [Reuters](https://www.english-corpora.org/corona/x3.asp?node=&p=9&w10=coronavirus&w11=vaccination&r=) |  |  |  | one dose by Sept. 15. read more # Vietnam has one of the lowest **coronavirus** **vaccination** rates in the region, with only 3.4% of its 98 million people fully vaccinated |
| [818](https://www.english-corpora.org/corona/x4.asp?rs=&t=87891635&ID=21565013347) | [21-09-06 US](https://www.english-corpora.org/corona/x4.asp?rs=&t=87891635&ID=21565013347) | [St. Louis Post-Dispatch](https://www.english-corpora.org/corona/x3.asp?node=&p=9&w10=coronavirus&w11=vaccination&r=) |  |  |  | man rests after receiving his second dose of the Pfizer COVID-19 vaccine at a **coronavirus** **vaccination** center set up at a synagogue in Bnei Brak, Israel, Sunday, March |
| [819](https://www.english-corpora.org/corona/x4.asp?rs=&t=87897668&ID=21580393883) | [21-09-07 US](https://www.english-corpora.org/corona/x4.asp?rs=&t=87897668&ID=21580393883) | [Yahoo](https://www.english-corpora.org/corona/x3.asp?node=&p=9&w10=coronavirus&w11=vaccination&r=) |  |  |  | that sick if she caught the virus. Eliza is old enough for a **coronavirus** **vaccination**, but her mother, Christie Datko, said she is opposed to it, |
| [820](https://www.english-corpora.org/corona/x4.asp?rs=&t=87898249&ID=21592192944) | [21-09-08 US](https://www.english-corpora.org/corona/x4.asp?rs=&t=87898249&ID=21592192944) | [Yahoo! Sports](https://www.english-corpora.org/corona/x3.asp?node=&p=9&w10=coronavirus&w11=vaccination&r=) |  |  |  | resigned after they were accused of being involved in a scheme to create fake **coronavirus** **vaccination** cards in the nation's most vaccinated state, the state police announced Tuesday. |
| [822](https://www.english-corpora.org/corona/x4.asp?rs=&t=87899631&ID=21589588912) | [21-09-08 US](https://www.english-corpora.org/corona/x4.asp?rs=&t=87899631&ID=21589588912) | [Associated Press](https://www.english-corpora.org/corona/x3.asp?node=&p=9&w10=coronavirus&w11=vaccination&r=)[(1)](https://www.english-corpora.org/corona/duplicates1.asp?xx=821&n=1) |  |  |  | -- The Massachusetts Public Health Council on Wednesday approved a plan to require **coronavirus** **vaccination** for all employees at rest homes, assisted living residences and hospice programs, along |
| [823](https://www.english-corpora.org/corona/x4.asp?rs=&t=87900103&ID=21589971457) | [21-09-08 US](https://www.english-corpora.org/corona/x4.asp?rs=&t=87900103&ID=21589971457) | [YAHOO!News](https://www.english-corpora.org/corona/x3.asp?node=&p=9&w10=coronavirus&w11=vaccination&r=) |  |  |  | resigned after they were accused of being involved in a scheme to create fake **coronavirus** **vaccination** cards in the nation's most vaccinated state, the state police announced Tuesday. |
| [824](https://www.english-corpora.org/corona/x4.asp?rs=&t=87900292&ID=21592829121) | [21-09-08 US](https://www.english-corpora.org/corona/x4.asp?rs=&t=87900292&ID=21592829121) | [Reuters](https://www.english-corpora.org/corona/x3.asp?node=&p=9&w10=coronavirus&w11=vaccination&r=) |  |  |  | the end of September. # " I've done my utmost to promote **coronavirus** **vaccination**, " he said. # Still, months of emergency curbs in Tokyo and |
| [825](https://www.english-corpora.org/corona/x4.asp?rs=&t=87901790&ID=21592607696) | [21-09-08 US](https://www.english-corpora.org/corona/x4.asp?rs=&t=87901790&ID=21592607696) | [Yahoo](https://www.english-corpora.org/corona/x3.asp?node=&p=9&w10=coronavirus&w11=vaccination&r=) |  |  |  | resigned after they were accused of being involved in a scheme to create fake **coronavirus** **vaccination** cards in the nation's most vaccinated state, the state police announced Tuesday. |
| [826](https://www.english-corpora.org/corona/x4.asp?rs=&t=87904512&ID=21601917629) | [21-09-09 US](https://www.english-corpora.org/corona/x4.asp?rs=&t=87904512&ID=21601917629) | [Yahoo! Sports](https://www.english-corpora.org/corona/x3.asp?node=&p=9&w10=coronavirus&w11=vaccination&r=) |  |  |  | one day take its place. # TOKYO (Reuters) -Japan's popular **coronavirus** **vaccination** minister, Taro Kono, is set to announce his candidacy on Friday to lead |
| [827](https://www.english-corpora.org/corona/x4.asp?rs=&t=87905038&ID=21604343069) | [21-09-09 US](https://www.english-corpora.org/corona/x4.asp?rs=&t=87905038&ID=21604343069) | [Houston Chronicle](https://www.english-corpora.org/corona/x3.asp?node=&p=9&w10=coronavirus&w11=vaccination&r=) |  |  |  | AP) -- Montana Gov. Greg Gianforte on Thursday said a new federal **coronavirus** **vaccination** mandate that could affect as many as 100 million Americans is " unlawful and un-American |
| [828](https://www.english-corpora.org/corona/x4.asp?rs=&t=87908241&ID=21603287177) | [21-09-09 US](https://www.english-corpora.org/corona/x4.asp?rs=&t=87908241&ID=21603287177) | [TheStreet.com](https://www.english-corpora.org/corona/x3.asp?node=&p=9&w10=coronavirus&w11=vaccination&r=) |  |  |  | thing. " # Biden had earlier asked all federal employees to prove their **coronavirus** **vaccination** status or submit to a series of rigorous safety protocols. Federal employees who were |
| [829](https://www.english-corpora.org/corona/x4.asp?rs=&t=87908668&ID=21605970346) | [21-09-09 US](https://www.english-corpora.org/corona/x4.asp?rs=&t=87908668&ID=21605970346) | [New York Daily News](https://www.english-corpora.org/corona/x3.asp?node=&p=9&w10=coronavirus&w11=vaccination&r=) |  |  |  | hit the streets Monday to start enforcing an indoor **coronavirus** **vaccination** mandate for restaurants, bars and other businesses -- but priority won't be placed |
| [830](https://www.english-corpora.org/corona/x4.asp?rs=&t=87909298&ID=21606004350) | [21-09-09 US](https://www.english-corpora.org/corona/x4.asp?rs=&t=87909298&ID=21606004350) | [Forbes](https://www.english-corpora.org/corona/x3.asp?node=&p=9&w10=coronavirus&w11=vaccination&r=) |  |  |  | # Students who are 11 years old and will become eligible to receive a **coronavirus** **vaccination** during the school year will need to get their first shot within 30 days of |
| [831](https://www.english-corpora.org/corona/x4.asp?rs=&t=87911397&ID=21614769644) | [21-09-10 US](https://www.english-corpora.org/corona/x4.asp?rs=&t=87911397&ID=21614769644) | [YAHOO!News](https://www.english-corpora.org/corona/x3.asp?node=&p=9&w10=coronavirus&w11=vaccination&r=) |  |  |  | ago, experts have warned. # TOKYO (Reuters) -Japan's popular **coronavirus** **vaccination** minister, Taro Kono, announced his candidacy on Friday to lead the ruling party |
| [832](https://www.english-corpora.org/corona/x4.asp?rs=&t=87912363&ID=21612637568) | [21-09-10 US](https://www.english-corpora.org/corona/x4.asp?rs=&t=87912363&ID=21612637568) | [New York Post](https://www.english-corpora.org/corona/x3.asp?node=&p=9&w10=coronavirus&w11=vaccination&r=) |  |  |  | Photo/Charlie Neibergall # In the public sector, all federal employees must receive a **coronavirus** **vaccination**, with extremely narrow exceptions. Federal employees will not have a weekly testing option |
| [833](https://www.english-corpora.org/corona/x4.asp?rs=&t=87912634&ID=21612948856) | [21-09-10 US](https://www.english-corpora.org/corona/x4.asp?rs=&t=87912634&ID=21612948856) | [The Motley Fool](https://www.english-corpora.org/corona/x3.asp?node=&p=9&w10=coronavirus&w11=vaccination&r=) |  |  |  | once again from U.S. ports? As more and more people lined up for **coronavirus** **vaccination**, there was reason for optimism. Vaccinations would help stop the pandemic -- and |
| [834](https://www.english-corpora.org/corona/x4.asp?rs=&t=87917297&ID=21615069808) | [21-09-10 US](https://www.english-corpora.org/corona/x4.asp?rs=&t=87917297&ID=21615069808) | [The New York Times on MSN.com](https://www.english-corpora.org/corona/x3.asp?node=&p=9&w10=coronavirus&w11=vaccination&r=) |  |  |  | Biden's new **coronavirus** **vaccination** mandates have prompted some backlash, but the two federal departments that already require vaccinations |
| [835](https://www.english-corpora.org/corona/x4.asp?rs=&t=87918982&ID=21625026342) | [21-09-11 US](https://www.english-corpora.org/corona/x4.asp?rs=&t=87918982&ID=21625026342) | [Yahoo! Sports](https://www.english-corpora.org/corona/x3.asp?node=&p=9&w10=coronavirus&w11=vaccination&r=) |  |  |  | money burning holes in... # As more and more people lined up for **coronavirus** **vaccination**, there was reason for optimism. As a result, Carnival (NYSE: |
| [836](https://www.english-corpora.org/corona/x4.asp?rs=&t=87921732&ID=21622746568) | [21-09-11 US](https://www.english-corpora.org/corona/x4.asp?rs=&t=87921732&ID=21622746568) | [Washington Post](https://www.english-corpora.org/corona/x3.asp?node=&p=9&w10=coronavirus&w11=vaccination&r=) |  |  |  | , Uganda takes vaccination drive to markets # A nurse administers a **coronavirus** **vaccination** at Kisenyi Health Center in downtown Kampala, Uganda Wednesday, Sept. 8, 2021 |
| [837](https://www.english-corpora.org/corona/x4.asp?rs=&t=87924932&ID=21628576652) | [21-09-12 US](https://www.english-corpora.org/corona/x4.asp?rs=&t=87924932&ID=21628576652) | [San Francisco Chronicle](https://www.english-corpora.org/corona/x3.asp?node=&p=9&w10=coronavirus&w11=vaccination&r=) |  |  |  | 500,000 doses of the Pfizer vaccine from Denmark as it tries to keep its **coronavirus** **vaccination** program running at full speed, the government said. # The doses are on |
| [838](https://www.english-corpora.org/corona/x4.asp?rs=&t=87925272&ID=21630907099) | [21-09-12 US](https://www.english-corpora.org/corona/x4.asp?rs=&t=87925272&ID=21630907099) | [New York Times](https://www.english-corpora.org/corona/x3.asp?node=&p=9&w10=coronavirus&w11=vaccination&r=) |  |  |  | for The New York Times # Mississippi, which has one of the lowest **coronavirus** **vaccination** rates in the nation, has consistently led the United States in childhood vaccinations -- |
| [839](https://www.english-corpora.org/corona/x4.asp?rs=&t=87928263&ID=21641645954) | [21-09-13 US](https://www.english-corpora.org/corona/x4.asp?rs=&t=87928263&ID=21641645954) | [Forbes](https://www.english-corpora.org/corona/x3.asp?node=&p=9&w10=coronavirus&w11=vaccination&r=) |  |  |  | # What Biden's New Coronavirus Plan Requires of Workers # Biden's new **coronavirus** **vaccination** requirements focus on four main groups of workers and employers. # First, there |
| [840](https://www.english-corpora.org/corona/x4.asp?rs=&t=87932626&ID=21641028527) | [21-09-13 US](https://www.english-corpora.org/corona/x4.asp?rs=&t=87932626&ID=21641028527) | [The Boston Globe on MSN.com](https://www.english-corpora.org/corona/x3.asp?node=&p=9&w10=coronavirus&w11=vaccination&r=) |  |  |  | 3:54 p.m. # By The Washington Post # As more countries turn to **coronavirus** **vaccination** requirements in a bid to bring the pandemic to heel, England is moving in |
| [841](https://www.english-corpora.org/corona/x4.asp?rs=&t=87932626&ID=21641031132) | [21-09-13 US](https://www.english-corpora.org/corona/x4.asp?rs=&t=87932626&ID=21641031132) | [The Boston Globe on MSN.com](https://www.english-corpora.org/corona/x3.asp?node=&p=9&w10=coronavirus&w11=vaccination&r=) |  |  |  | 500,000 doses of the Pfizer vaccine from Denmark as it tries to keep its **coronavirus** **vaccination** program running at full speed, the government said. # The doses are on |
| [842](https://www.english-corpora.org/corona/x4.asp?rs=&t=87935440&ID=21652101138) | [21-09-14 US](https://www.english-corpora.org/corona/x4.asp?rs=&t=87935440&ID=21652101138) | [Business Insider](https://www.english-corpora.org/corona/x3.asp?node=&p=9&w10=coronavirus&w11=vaccination&r=) |  |  |  | reasons. " # The lawsuit also claimed that people who are exempt from **coronavirus** **vaccination** are punished with " invasive testing " that violates their privacy rights. # Of |
| [843](https://www.english-corpora.org/corona/x4.asp?rs=&t=87935730&ID=21650586855) | [21-09-14 US](https://www.english-corpora.org/corona/x4.asp?rs=&t=87935730&ID=21650586855) | [The Washington Post on MSN.com](https://www.english-corpora.org/corona/x3.asp?node=&p=9&w10=coronavirus&w11=vaccination&r=) |  |  |  | any and all vaccines they are eligible for -- whether it's their first **coronavirus** **vaccination**, a booster vaccine dose to combat waning immunity or a flu shot. # |
| [844](https://www.english-corpora.org/corona/x4.asp?rs=&t=87937281&ID=21653722053) | [21-09-14 US](https://www.english-corpora.org/corona/x4.asp?rs=&t=87937281&ID=21653722053) | [Forbes](https://www.english-corpora.org/corona/x3.asp?node=&p=9&w10=coronavirus&w11=vaccination&r=) |  |  |  | shortage " that has been driven by surges in demand in states with low **coronavirus** **vaccination** rates. # Kentucky Governor Andy Beshear (R) speaks to the media as |
| [845](https://www.english-corpora.org/corona/x4.asp?rs=&t=87939377&ID=21654642423) | [21-09-14 US](https://www.english-corpora.org/corona/x4.asp?rs=&t=87939377&ID=21654642423) | [New York Times](https://www.english-corpora.org/corona/x3.asp?node=&p=9&w10=coronavirus&w11=vaccination&r=) |  |  |  | the Instagram handle AntiVaxMomma was charged in a conspiracy to sell hundreds of fake **coronavirus** **vaccination** cards over the social media platform, Manhattan prosecutors said on Tuesday. # The |
| [846](https://www.english-corpora.org/corona/x4.asp?rs=&t=87940022&ID=21662252729) | [21-09-15 US](https://www.english-corpora.org/corona/x4.asp?rs=&t=87940022&ID=21662252729) | [ESPN](https://www.english-corpora.org/corona/x3.asp?node=&p=9&w10=coronavirus&w11=vaccination&r=) |  |  |  | players to get vaccinated as Premiership Rugby introduced relaxed rules for teams with high **coronavirus** **vaccination** rates. # On Monday, Premiership Rugby said in a statement that clubs would |
| [848](https://www.english-corpora.org/corona/x4.asp?rs=&t=87942933&ID=21662437006) | [21-09-15 US](https://www.english-corpora.org/corona/x4.asp?rs=&t=87942933&ID=21662437006) | [The Washington Post on MSN.com](https://www.english-corpora.org/corona/x3.asp?node=&p=9&w10=coronavirus&w11=vaccination&r=)[(1)](https://www.english-corpora.org/corona/duplicates1.asp?xx=847&n=1) |  |  |  | applicants, including measles, polio, influenza and tetanus. # The **coronavirus** **vaccination** requirement follows updated guidance from the Centers for Disease Control and Prevention, USCIS said |
| [850](https://www.english-corpora.org/corona/x4.asp?rs=&t=87950439&ID=21675392107) | [21-09-16 US](https://www.english-corpora.org/corona/x4.asp?rs=&t=87950439&ID=21675392107) | [Washington Post](https://www.english-corpora.org/corona/x3.asp?node=&p=9&w10=coronavirus&w11=vaccination&r=)[(1)](https://www.english-corpora.org/corona/duplicates1.asp?xx=849&n=1) |  |  |  | state law says businesses can't require customers to show proof of a **coronavirus** **vaccination**. (Chuck Burton/AP) # By Nicholas Barry Creel # and # Jehan El-Jourbagy |
| [851](https://www.english-corpora.org/corona/x4.asp?rs=&t=87952642&ID=21687209932) | [21-09-17 US](https://www.english-corpora.org/corona/x4.asp?rs=&t=87952642&ID=21687209932) | [ABC](https://www.english-corpora.org/corona/x3.asp?node=&p=9&w10=coronavirus&w11=vaccination&r=) |  |  |  | living. " # Robyn Beck/AFP via Getty Images # A nurse marks a **coronavirus** **vaccination** card with a third &quot; booster&quot; dose of the Pfizer COVID-19 vaccine at |
| [852](https://www.english-corpora.org/corona/x4.asp?rs=&t=87954320&ID=21685602094) | [21-09-17 US](https://www.english-corpora.org/corona/x4.asp?rs=&t=87954320&ID=21685602094) | [The Hill](https://www.english-corpora.org/corona/x3.asp?node=&p=9&w10=coronavirus&w11=vaccination&r=) |  |  |  | both public and private. It is the first country in Europe to require **coronavirus** **vaccination** certificates so widely. " It's an extraordinary endeavor, " Italy's public |
| [853](https://www.english-corpora.org/corona/x4.asp?rs=&t=87960119&ID=21696900666) | [21-09-18 US](https://www.english-corpora.org/corona/x4.asp?rs=&t=87960119&ID=21696900666) | [YAHOO!News](https://www.english-corpora.org/corona/x3.asp?node=&p=9&w10=coronavirus&w11=vaccination&r=) |  |  |  | in a statement. # In January, Alaska had the highest per capita **coronavirus** **vaccination** rate in the nation. Providence Alaska Medical Center, the state's largest hospital |
| [855](https://www.english-corpora.org/corona/x4.asp?rs=&t=87969325&ID=21713746712) | [21-09-20 US](https://www.english-corpora.org/corona/x4.asp?rs=&t=87969325&ID=21713746712) | [The Washington Post on MSN.com](https://www.english-corpora.org/corona/x3.asp?node=&p=9&w10=coronavirus&w11=vaccination&r=)[(1)](https://www.english-corpora.org/corona/duplicates1.asp?xx=854&n=1) |  |  |  | platforms. # 10:59 PM: Doubters' push for religious exemptions from **coronavirus** **vaccination** may not work # Compulsory coronavirus vaccination has been a specter hovering over vaccine skeptics |
| [856](https://www.english-corpora.org/corona/x4.asp?rs=&t=87969325&ID=21713746719) | [21-09-20 US](https://www.english-corpora.org/corona/x4.asp?rs=&t=87969325&ID=21713746719) | [The Washington Post on MSN.com](https://www.english-corpora.org/corona/x3.asp?node=&p=9&w10=coronavirus&w11=vaccination&r=) |  |  |  | Doubters' push for religious exemptions from coronavirus vaccination may not work # Compulsory **coronavirus** **vaccination** has been a specter hovering over vaccine skeptics throughout the pandemic, but the issue |
| [857](https://www.english-corpora.org/corona/x4.asp?rs=&t=87969325&ID=21713746814) | [21-09-20 US](https://www.english-corpora.org/corona/x4.asp?rs=&t=87969325&ID=21713746814) | [The Washington Post on MSN.com](https://www.english-corpora.org/corona/x3.asp?node=&p=9&w10=coronavirus&w11=vaccination&r=) |  |  |  | process for gaining such an exemption suggests that those seeking religious exemptions to the **coronavirus** **vaccination** mandates will not be widely successful. In recent years, many states, including |
| [858](https://www.english-corpora.org/corona/x4.asp?rs=&t=87971081&ID=21715899096) | [21-09-20 US](https://www.english-corpora.org/corona/x4.asp?rs=&t=87971081&ID=21715899096) | [seattlepi.com](https://www.english-corpora.org/corona/x3.asp?node=&p=9&w10=coronavirus&w11=vaccination&r=) |  |  |  | at the Putnam Clubhouse at Gillette Stadium in Foxboro, Mass. to receive his **coronavirus** **vaccination**. The resurgence of COVID-19 this summer and the national debate over vaccine requirements have |
| [859](https://www.english-corpora.org/corona/x4.asp?rs=&t=87971536&ID=21715400644) | [21-09-20 US](https://www.english-corpora.org/corona/x4.asp?rs=&t=87971536&ID=21715400644) | [NJ.com on MSN.com](https://www.english-corpora.org/corona/x3.asp?node=&p=9&w10=coronavirus&w11=vaccination&r=) |  |  |  | Monmouth County nurse supervisor would be granted a religious exemption from her hospital's **coronavirus** **vaccination** mandate, she was ready to quit after 23 years. # And nothing was |
| [860](https://www.english-corpora.org/corona/x4.asp?rs=&t=87973869&ID=21728305714) | [21-09-21 US](https://www.english-corpora.org/corona/x4.asp?rs=&t=87973869&ID=21728305714) | [New York Post](https://www.english-corpora.org/corona/x3.asp?node=&p=9&w10=coronavirus&w11=vaccination&r=) |  |  |  | the Brazilian president. # In August, New York City mandated proof of **coronavirus** **vaccination** for people to enter certain indoor businesses including all indoor restaurants, entertainment venues and |
| [861](https://www.english-corpora.org/corona/x4.asp?rs=&t=87976877&ID=21728154263) | [21-09-21 US](https://www.english-corpora.org/corona/x4.asp?rs=&t=87976877&ID=21728154263) | [New York Daily News](https://www.english-corpora.org/corona/x3.asp?node=&p=9&w10=coronavirus&w11=vaccination&r=) |  |  |  | . Hochul said Tuesday that 120 new popup **coronavirus** **vaccination** sites will crop up across New York State, part of a " VaxtoSchool " |
| [862](https://www.english-corpora.org/corona/x4.asp?rs=&t=53138319&ID=21645116026) | [21-09-14 CA](https://www.english-corpora.org/corona/x4.asp?rs=&t=53138319&ID=21645116026) | [prpeak.com](https://www.english-corpora.org/corona/x3.asp?node=&p=9&w10=coronavirus&w11=vaccination&r=) |  |  |  | -- - # SYDNEY -- Australia's New South Wales state has hit a **coronavirus** **vaccination** milestone, with 80% of the target population receiving a shot, and the government |
| [863](https://www.english-corpora.org/corona/x4.asp?rs=&t=53153298&ID=21695051299) | [21-09-18 CA](https://www.english-corpora.org/corona/x4.asp?rs=&t=53153298&ID=21695051299) | [arabtimesonline.com](https://www.english-corpora.org/corona/x3.asp?node=&p=9&w10=coronavirus&w11=vaccination&r=) |  |  |  | latest. Sources told the daily that the ministry is planning to allocate some **coronavirus** **vaccination** centers for seasonal influenza and pneumonia vaccination in order to prevent crowding or long queues |
| [864](https://www.english-corpora.org/corona/x4.asp?rs=&t=43419331&ID=21606120742) | [21-09-10 GB](https://www.english-corpora.org/corona/x4.asp?rs=&t=43419331&ID=21606120742) | [uk.news.yahoo.com](https://www.english-corpora.org/corona/x3.asp?node=&p=9&w10=coronavirus&w11=vaccination&r=) |  |  |  | his political ambitions above governing. # TOKYO (Reuters) -Japan's popular **coronavirus** **vaccination** minister, Taro Kono, announced his candidacy on Friday to lead the ruling party |
| [866](https://www.english-corpora.org/corona/x4.asp?rs=&t=43425959&ID=21646761628) | [21-09-14 GB](https://www.english-corpora.org/corona/x4.asp?rs=&t=43425959&ID=21646761628) | [expressandstar.com](https://www.english-corpora.org/corona/x3.asp?node=&p=9&w10=coronavirus&w11=vaccination&r=)[(1)](https://www.english-corpora.org/corona/duplicates1.asp?xx=865&n=1) |  |  |  | the Commons on Tuesday. # Subscribe to our daily newsletter! # **Coronavirus** **vaccination** # Boris Johnson will put his trust in a " massive " booster vaccination campaign |
| [867](https://www.english-corpora.org/corona/x4.asp?rs=&t=43428688&ID=21670680208) | [21-09-16 GB](https://www.english-corpora.org/corona/x4.asp?rs=&t=43428688&ID=21670680208) | [yorkshirepost.co.uk](https://www.english-corpora.org/corona/x3.asp?node=&p=9&w10=coronavirus&w11=vaccination&r=) |  |  |  | the cases are still too low to be added as a common symptom of **coronavirus** **vaccination**. # Dr Male added: " MHRA states that evaluation of yellow card reports |
| [868](https://www.english-corpora.org/corona/x4.asp?rs=&t=43441940&ID=21708591691) | [21-09-20 GB](https://www.english-corpora.org/corona/x4.asp?rs=&t=43441940&ID=21708591691) | [chroniclelive.co.uk](https://www.english-corpora.org/corona/x3.asp?node=&p=9&w10=coronavirus&w11=vaccination&r=) |  |  |  | in patients being admitted with serious illness. # However the success of the **coronavirus** **vaccination** programme means the trust has been able to start re-introducing some visiting at its three |
| [869](https://www.english-corpora.org/corona/x4.asp?rs=&t=87920687&ID=21625551463) | [21-09-11 GB](https://www.english-corpora.org/corona/x4.asp?rs=&t=87920687&ID=21625551463) | [ITV](https://www.english-corpora.org/corona/x3.asp?node=&p=9&w10=coronavirus&w11=vaccination&r=) |  |  |  | to this group. # Health Secretary Sajid Javid # The DHSC said the **coronavirus** **vaccination** programme has been so successful because plans were put in place before the medicines regulator |
| [870](https://www.english-corpora.org/corona/x4.asp?rs=&t=87941266&ID=21664404180) | [21-09-15 GB](https://www.english-corpora.org/corona/x4.asp?rs=&t=87941266&ID=21664404180) | [Sky](https://www.english-corpora.org/corona/x3.asp?node=&p=9&w10=coronavirus&w11=vaccination&r=) |  |  |  | many of those who suffer from it also have lower levels of antibodies after **coronavirus** **vaccination** than the general population. Related Topics: # Data from the so-called OCTAVE |
| [871](https://www.english-corpora.org/corona/x4.asp?rs=&t=63323385&ID=21600594667) | [21-09-09 IE](https://www.english-corpora.org/corona/x4.asp?rs=&t=63323385&ID=21600594667) | [flightglobal.com](https://www.english-corpora.org/corona/x3.asp?node=&p=9&w10=coronavirus&w11=vaccination&r=) |  |  |  | his country will continue to look at ways to restore international air travel as **coronavirus** **vaccination** rates rise globally, including discussions with the USA. # Singapore has announced a |
| [872](https://www.english-corpora.org/corona/x4.asp?rs=&t=73434348&ID=21598702871) | [21-09-09 AU](https://www.english-corpora.org/corona/x4.asp?rs=&t=73434348&ID=21598702871) | [examiner.com.au](https://www.english-corpora.org/corona/x3.asp?node=&p=9&w10=coronavirus&w11=vaccination&r=) |  |  |  | with forecasts that 80 per cent of eligible Australians will have had their second **coronavirus** **vaccination** by mid-November. " When we can safely move from restrictions to freedoms, I |
| [873](https://www.english-corpora.org/corona/x4.asp?rs=&t=73434348&ID=21598703511) | [21-09-09 AU](https://www.english-corpora.org/corona/x4.asp?rs=&t=73434348&ID=21598703511) | [examiner.com.au](https://www.english-corpora.org/corona/x3.asp?node=&p=9&w10=coronavirus&w11=vaccination&r=) |  |  |  | with forecasts that 80 per cent of eligible Australians will have had their second **coronavirus** **vaccination** by mid-November. # " When we can safely move from restrictions to freedoms, |
| [874](https://www.english-corpora.org/corona/x4.asp?rs=&t=73438053&ID=21608862677) | [21-09-10 AU](https://www.english-corpora.org/corona/x4.asp?rs=&t=73438053&ID=21608862677) | [perthnow.com.au](https://www.english-corpora.org/corona/x3.asp?node=&p=9&w10=coronavirus&w11=vaccination&r=) |  |  |  | enters leader race Topics # Japan's popular **coronavirus** **vaccination** minister, Taro Kono, has announced his candidacy to lead the ruling party and |
| [875](https://www.english-corpora.org/corona/x4.asp?rs=&t=73455925&ID=21668011215) | [21-09-16 AU](https://www.english-corpora.org/corona/x4.asp?rs=&t=73455925&ID=21668011215) | [broadsheet.com.au](https://www.english-corpora.org/corona/x3.asp?node=&p=9&w10=coronavirus&w11=vaccination&r=) |  |  |  | September 2021 # by Nick Connellan # Share # Victoria is fast approaching a **coronavirus** **vaccination** milestone -- 69.2 per cent of people over the age of 16 have now received |
| [876](https://www.english-corpora.org/corona/x4.asp?rs=&t=73456102&ID=21668032266) | [21-09-16 AU](https://www.english-corpora.org/corona/x4.asp?rs=&t=73456102&ID=21668032266) | [indaily.com.au](https://www.english-corpora.org/corona/x3.asp?node=&p=9&w10=coronavirus&w11=vaccination&r=) |  |  |  | impose an obligation on private and public sector employees to show evidence of a **coronavirus** **vaccination** or negative test results or be banned from working in offices, shops and restaurants |
| [877](https://www.english-corpora.org/corona/x4.asp?rs=&t=73458627&ID=21679057173) | [21-09-17 AU](https://www.english-corpora.org/corona/x4.asp?rs=&t=73458627&ID=21679057173) | [canberratimes.com.au](https://www.english-corpora.org/corona/x3.asp?node=&p=9&w10=coronavirus&w11=vaccination&r=) |  |  |  | 73458627 # Western Australia has tens of thousands of empty **coronavirus** **vaccination** slots as authorities prepare to offer jabs at selected secondary schools. Vaccine Commander Chris |
| [878](https://www.english-corpora.org/corona/x4.asp?rs=&t=73458627&ID=21679057664) | [21-09-17 AU](https://www.english-corpora.org/corona/x4.asp?rs=&t=73458627&ID=21679057664) | [canberratimes.com.au](https://www.english-corpora.org/corona/x3.asp?node=&p=9&w10=coronavirus&w11=vaccination&r=) |  |  |  | to fill empty vaccination slots # Western Australia has tens of thousands of empty **coronavirus** **vaccination** slots as authorities prepare to offer jabs at selected secondary schools. # Vaccine Commander |
| [879](https://www.english-corpora.org/corona/x4.asp?rs=&t=73459395&ID=21684934122) | [21-09-17 AU](https://www.english-corpora.org/corona/x4.asp?rs=&t=73459395&ID=21684934122) | [examiner.com.au](https://www.english-corpora.org/corona/x3.asp?node=&p=9&w10=coronavirus&w11=vaccination&r=) |  |  |  | a **coronavirus** **vaccination** milestone, with more than half of the island state's population aged over 16 |
| [880](https://www.english-corpora.org/corona/x4.asp?rs=&t=73466480&ID=21701334061) | [21-09-19 AU](https://www.english-corpora.org/corona/x4.asp?rs=&t=73466480&ID=21701334061) | [perthnow.com.au](https://www.english-corpora.org/corona/x3.asp?node=&p=9&w10=coronavirus&w11=vaccination&r=) |  |  |  | each week from October. # * Tasmania is targeting a 90 per cent **coronavirus** **vaccination** rate by December so the island can " open with confidence " for summer and |
| [881](https://www.english-corpora.org/corona/x4.asp?rs=&t=73466480&ID=21701334090) | [21-09-19 AU](https://www.english-corpora.org/corona/x4.asp?rs=&t=73466480&ID=21701334090) | [perthnow.com.au](https://www.english-corpora.org/corona/x3.asp?node=&p=9&w10=coronavirus&w11=vaccination&r=) |  |  |  | summer and Christmas. # * Western Australia has tens of thousands of empty **coronavirus** **vaccination** slots as authorities prepare to offer jabs at selected secondary schools. # * Australia |
| [882](https://www.english-corpora.org/corona/x4.asp?rs=&t=73469596&ID=21701765932) | [21-09-19 AU](https://www.english-corpora.org/corona/x4.asp?rs=&t=73469596&ID=21701765932) | [indaily.com.au](https://www.english-corpora.org/corona/x3.asp?node=&p=9&w10=coronavirus&w11=vaccination&r=) |  |  |  | completion of the terminal expansion was expected to coincide with increased visitor numbers as **coronavirus** **vaccination** rates climbed and border restrictions eased. # " It's great to see the |
| [883](https://www.english-corpora.org/corona/x4.asp?rs=&t=73472912&ID=21718909915) | [21-09-21 AU](https://www.english-corpora.org/corona/x4.asp?rs=&t=73472912&ID=21718909915) | [brisbanetimes.com.au](https://www.english-corpora.org/corona/x3.asp?node=&p=9&w10=coronavirus&w11=vaccination&r=) |  |  |  | " # Twelve-year-old Eve, accompanied by her mum Narelle, having his first **coronavirus** **vaccination** at the Heidelberg Repatriation HospitalCredit:Eddie Jim # Q. The Doherty report talks about 70 per |
| [884](https://www.english-corpora.org/corona/x4.asp?rs=&t=53125069&ID=21600263344) | [21-09-09 PK](https://www.english-corpora.org/corona/x4.asp?rs=&t=53125069&ID=21600263344) | [samaa.tv](https://www.english-corpora.org/corona/x3.asp?node=&p=9&w10=coronavirus&w11=vaccination&r=) |  |  |  | the Sindh Education Department announced that **coronavirus** **vaccination** drives across educational institutions in the province will commence from September 6. The drive |
| [885](https://www.english-corpora.org/corona/x4.asp?rs=&t=53125069&ID=21600263505) | [21-09-09 PK](https://www.english-corpora.org/corona/x4.asp?rs=&t=53125069&ID=21600263505) | [samaa.tv](https://www.english-corpora.org/corona/x3.asp?node=&p=9&w10=coronavirus&w11=vaccination&r=) |  |  |  | on several indicators to devise a Covid prevention mechanism. The government recently made **coronavirus** **vaccination** mandatory for students across the country. Students won't be allowed on campuses if |
| [886](https://www.english-corpora.org/corona/x4.asp?rs=&t=53125069&ID=21600263663) | [21-09-09 PK](https://www.english-corpora.org/corona/x4.asp?rs=&t=53125069&ID=21600263663) | [samaa.tv](https://www.english-corpora.org/corona/x3.asp?node=&p=9&w10=coronavirus&w11=vaccination&r=) |  |  |  | by September 30. # On Sunday, the Sindh Education Department announced that **coronavirus** **vaccination** drives across educational institutions in the province will commence from September 6. The drive |
| [887](https://www.english-corpora.org/corona/x4.asp?rs=&t=53134943&ID=21627203228) | [21-09-12 PK](https://www.english-corpora.org/corona/x4.asp?rs=&t=53134943&ID=21627203228) | [samaa.tv](https://www.english-corpora.org/corona/x3.asp?node=&p=9&w10=coronavirus&w11=vaccination&r=) |  |  |  | . # Umar has also raised objections at posters showing key PPP figures at **coronavirus** **vaccination** centres and said that not even a single dose of vaccine had been brought by |
| [888](https://www.english-corpora.org/corona/x4.asp?rs=&t=53162749&ID=21720463438) | [21-09-21 PK](https://www.english-corpora.org/corona/x4.asp?rs=&t=53162749&ID=21720463438) | [oyeyeah.com](https://www.english-corpora.org/corona/x3.asp?node=&p=9&w10=coronavirus&w11=vaccination&r=) |  |  |  | dose by September 15 # Earlier in the day, the Balochistan government made **coronavirus** **vaccination** mandatory for people travelling inside and outside the province by bus. # According to |
| [889](https://www.english-corpora.org/corona/x4.asp?rs=&t=53118338&ID=21584965242) | [21-09-08 BD](https://www.english-corpora.org/corona/x4.asp?rs=&t=53118338&ID=21584965242) | [theindependentbd.com](https://www.english-corpora.org/corona/x3.asp?node=&p=9&w10=coronavirus&w11=vaccination&r=) |  |  |  | how vulnerable school systems were as a result of a range of factors including **coronavirus** **vaccination** coverage, the climate crisis, physical attacks, and the proportion of school-age children |
| [890](https://www.english-corpora.org/corona/x4.asp?rs=&t=53127194&ID=21609486173) | [21-09-10 BD](https://www.english-corpora.org/corona/x4.asp?rs=&t=53127194&ID=21609486173) | [thedailystar.net](https://www.english-corpora.org/corona/x3.asp?node=&p=9&w10=coronavirus&w11=vaccination&r=) |  |  |  | coronavirus **vaccination** minister, Taro Kono, announced his candidacy on Friday to lead the ruling party |
| [891](https://www.english-corpora.org/corona/x4.asp?rs=&t=53130708&ID=21616332990) | [21-09-11 BD](https://www.english-corpora.org/corona/x4.asp?rs=&t=53130708&ID=21616332990) | [bdnews24.com](https://www.english-corpora.org/corona/x3.asp?node=&p=9&w10=coronavirus&w11=vaccination&r=) |  |  |  | 19, 2021, the first day of the second phase of Bangladesh's **coronavirus** **vaccination** campaign. Photo: Mahmud Zaman Ovi A new shipment of 5.4 million doses |
| [893](https://www.english-corpora.org/corona/x4.asp?rs=&t=43414984&ID=21587676914) | [21-09-08 SG](https://www.english-corpora.org/corona/x4.asp?rs=&t=43414984&ID=21587676914) | [sbr.com.sg](https://www.english-corpora.org/corona/x3.asp?node=&p=9&w10=coronavirus&w11=vaccination&r=) [(1)](https://www.english-corpora.org/corona/duplicates1.asp?xx=892&n=1) |  |  |  | fleet of text messages inviting recipients to set up an appointment for a **coronavirus** **vaccination**, or urgent offers of " leftover " vaccine supposedly up for grabs. # |
| [894](https://www.english-corpora.org/corona/x4.asp?rs=&t=73425948&ID=21572579063) | [21-09-07 PH](https://www.english-corpora.org/corona/x4.asp?rs=&t=73425948&ID=21572579063) | [rappler.com](https://www.english-corpora.org/corona/x3.asp?node=&p=9&w10=coronavirus&w11=vaccination&r=) |  |  |  | said on Sunday, September 5. # Vietnam has one of the lowest **coronavirus** **vaccination** rates in the region, with only 3.3% of the country's 98 million people |
| [895](https://www.english-corpora.org/corona/x4.asp?rs=&t=43432840&ID=21680455035) | [21-09-17 HK](https://www.english-corpora.org/corona/x4.asp?rs=&t=43432840&ID=21680455035) | [hongkongfp.com](https://www.english-corpora.org/corona/x3.asp?node=&p=9&w10=coronavirus&w11=vaccination&r=) |  |  |  | . HKFP has reached out to the hospital for confirmation and comment. # **Coronavirus** **vaccination** in Hong Kong. Photo: GovHK. # The Hong Kong government's free |
| [896](https://www.english-corpora.org/corona/x4.asp?rs=&t=63316785&ID=21574592206) | [21-09-07 ZA](https://www.english-corpora.org/corona/x4.asp?rs=&t=63316785&ID=21574592206) | [businesslive.co.za](https://www.english-corpora.org/corona/x3.asp?node=&p=9&w10=coronavirus&w11=vaccination&r=) |  |  |  | Kahn # The deans of SA's medical and dental schools have recommended compulsory **coronavirus** **vaccination** for their students and the general healthcare workforce, arguing it will protect individuals from |
| [897](https://www.english-corpora.org/corona/x4.asp?rs=&t=63359229&ID=21691549838) | [21-09-18 ZA](https://www.english-corpora.org/corona/x4.asp?rs=&t=63359229&ID=21691549838) | [ewn.co.za](https://www.english-corpora.org/corona/x3.asp?node=&p=9&w10=coronavirus&w11=vaccination&r=) |  |  |  | certificate was required for ticket purchases. # Brazil had a late start with **coronavirus** **vaccination** but is now the country with the fourth-most doses administered. # Nearly two-thirds of |
| [898](https://www.english-corpora.org/corona/x4.asp?rs=&t=63360141&ID=21700300922) | [21-09-19 ZA](https://www.english-corpora.org/corona/x4.asp?rs=&t=63360141&ID=21700300922) | [it-online.co.za](https://www.english-corpora.org/corona/x3.asp?node=&p=9&w10=coronavirus&w11=vaccination&r=) |  |  |  | of 12. # " Our expectation is that the black market for fake **coronavirus** **vaccination** cards will continue to thrive as more policy requiring vaccination proof gets rolled out. |
| [899](https://www.english-corpora.org/corona/x4.asp?rs=&t=53131636&ID=21618762034) | [21-09-11 NG](https://www.english-corpora.org/corona/x4.asp?rs=&t=53131636&ID=21618762034) | [thestreetjournal.org](https://www.english-corpora.org/corona/x3.asp?node=&p=9&w10=coronavirus&w11=vaccination&r=) |  |  |  | off **coronavirus** **vaccination** campaign # This picture taken on December 17, 2020 shows Saudi Arabia's Health |
| [900](https://www.english-corpora.org/corona/x4.asp?rs=&t=87912597&ID=21614835654) | [21-09-10 --](https://www.english-corpora.org/corona/x4.asp?rs=&t=87912597&ID=21614835654) | [Express & Star](https://www.english-corpora.org/corona/x3.asp?node=&p=9&w10=coronavirus&w11=vaccination&r=) |  |  |  | be ready to hit the ground running ". # The DHSC said the **coronavirus** **vaccination** programme has been so successful because plans were put in place before the medicines regulator |

PAGE:   [**<<**](https://www.english-corpora.org/corona/x3.asp?node=&p=1&w10=coronavirus&w11=vaccination&r=)  [**<**](https://www.english-corpora.org/corona/x3.asp?node=&p=8&w10=coronavirus&w11=vaccination&r=)   9 / 42  [**>**](https://www.english-corpora.org/corona/x3.asp?node=&p=10&w10=coronavirus&w11=vaccination&r=)   [**>>**](https://www.english-corpora.org/corona/x3.asp?node=&p=42&w10=coronavirus&w11=vaccination&r=)

Конец формы

| FIND SAMPLE:   [100](https://www.english-corpora.org/corona/x3.asp?node=&sample=100&w10=coronavirus&w11=vaccination&r=)  [200](https://www.english-corpora.org/corona/x3.asp?node=&sample=200&w10=coronavirus&w11=vaccination&r=)  [500](https://www.english-corpora.org/corona/x3.asp?node=&sample=500&w10=coronavirus&w11=vaccination&r=)  [1000](https://www.english-corpora.org/corona/x3.asp?node=&sample=1000&w10=coronavirus&w11=vaccination&r=) PAGE:   [**<<**](https://www.english-corpora.org/corona/x3.asp?node=&p=1&w10=coronavirus&w11=vaccination&r=)  [**<**](https://www.english-corpora.org/corona/x3.asp?node=&p=9&w10=coronavirus&w11=vaccination&r=)   10 / 42  [**>**](https://www.english-corpora.org/corona/x3.asp?node=&p=11&w10=coronavirus&w11=vaccination&r=)   [**>>**](https://www.english-corpora.org/corona/x3.asp?node=&p=42&w10=coronavirus&w11=vaccination&r=) |  |
| --- | --- |

Начало формы

| CLICK FOR MORE CONTEXT | [**HELP**](javascript:newFeatures()) | [SAVE](javascript:chooser('s'))    [TRANSLATE](javascript:chooser('t'))    [ANALYZE](javascript:chooser('p')) |
| --- | --- | --- |

| [901](https://www.english-corpora.org/corona/x4.asp?rs=&t=33176657&ID=21481218259) | [21-08-29 US](https://www.english-corpora.org/corona/x4.asp?rs=&t=33176657&ID=21481218259) | [chicago.suntimes.com](https://www.english-corpora.org/corona/x3.asp?node=&p=10&w10=coronavirus&w11=vaccination&r=) |  |  |  | a guardian ad litem in both DuPage and Cook counties. She said the **coronavirus** **vaccination** issue is coming up more and more, and judges are looking to science for |
| --- | --- | --- | --- | --- | --- | --- |
| [902](https://www.english-corpora.org/corona/x4.asp?rs=&t=43356999&ID=21421250959) | [21-08-23 US](https://www.english-corpora.org/corona/x4.asp?rs=&t=43356999&ID=21421250959) | [baxterbulletin.com](https://www.english-corpora.org/corona/x3.asp?node=&p=10&w10=coronavirus&w11=vaccination&r=) |  |  |  | at home or onsite. # ? The grand prize winner of Michigan's **coronavirus** **vaccination** sweepstakes said Monday that she and her husband will save money for their three kids |
| [903](https://www.english-corpora.org/corona/x4.asp?rs=&t=43381530&ID=21488525630) | [21-08-30 US](https://www.english-corpora.org/corona/x4.asp?rs=&t=43381530&ID=21488525630) | [wsbtv.com](https://www.english-corpora.org/corona/x3.asp?node=&p=10&w10=coronavirus&w11=vaccination&r=) |  |  |  | a selfie while receiving the third Pfizer-BioNTech COVID-19 vaccine from medical staff at a **coronavirus** **vaccination** center in Ramat Gan, Israel, Monday, Aug. 30, 2021. Israel |
| [904](https://www.english-corpora.org/corona/x4.asp?rs=&t=53087954&ID=21473560722) | [21-08-28 US](https://www.english-corpora.org/corona/x4.asp?rs=&t=53087954&ID=21473560722) | [abc7news.com](https://www.english-corpora.org/corona/x3.asp?node=&p=10&w10=coronavirus&w11=vaccination&r=) |  |  |  | venues and more. # The city has one of the nation's highest **coronavirus** **vaccination** rates at nearly 80-percent, but with the Delta variant, it wants more, |
| [905](https://www.english-corpora.org/corona/x4.asp?rs=&t=63285723&ID=21461030861) | [21-08-27 US](https://www.english-corpora.org/corona/x4.asp?rs=&t=63285723&ID=21461030861) | [axios.com](https://www.english-corpora.org/corona/x3.asp?node=&p=10&w10=coronavirus&w11=vaccination&r=) |  |  |  | Kabul. Photo: Chip Somodevilla/Getty Images # The U.S. has set up a **coronavirus** **vaccination** site for Afghans arriving at the Dulles International Airport in Virginia, a White House |
| [907](https://www.english-corpora.org/corona/x4.asp?rs=&t=63290197&ID=21484781699) | [21-08-29 US](https://www.english-corpora.org/corona/x4.asp?rs=&t=63290197&ID=21484781699) | [axios.com](https://www.english-corpora.org/corona/x3.asp?node=&p=10&w10=coronavirus&w11=vaccination&r=) [(1)](https://www.english-corpora.org/corona/duplicates1.asp?xx=906&n=1) |  |  |  | # Why it matters: Singapore has one of the world's highest **coronavirus** **vaccination** rates, and the milestone means the government can continue loosening restrictions and prepare to |
| [908](https://www.english-corpora.org/corona/x4.asp?rs=&t=63291691&ID=21492833087) | [21-08-30 US](https://www.english-corpora.org/corona/x4.asp?rs=&t=63291691&ID=21492833087) | [q13fox.com](https://www.english-corpora.org/corona/x3.asp?node=&p=10&w10=coronavirus&w11=vaccination&r=) |  |  |  | to 17 in the United States have received at least one dose of a **coronavirus** **vaccination**, the White House confirmed Friday. # " We have now hit a major |
| [909](https://www.english-corpora.org/corona/x4.asp?rs=&t=63293790&ID=21500971824) | [21-08-31 US](https://www.english-corpora.org/corona/x4.asp?rs=&t=63293790&ID=21500971824) | [reporternews.com](https://www.english-corpora.org/corona/x3.asp?node=&p=10&w10=coronavirus&w11=vaccination&r=) |  |  |  | Considering Taylor County's dismal vaccination rate, they should not wonder. # **Coronavirus** **vaccination** seems to be a bizarrely partisan issue. Blue states, where smart Democrats live |
| [910](https://www.english-corpora.org/corona/x4.asp?rs=&t=73384429&ID=21452039750) | [21-08-26 US](https://www.english-corpora.org/corona/x4.asp?rs=&t=73384429&ID=21452039750) | [wfmz.com](https://www.english-corpora.org/corona/x3.asp?node=&p=10&w10=coronavirus&w11=vaccination&r=) |  |  |  | workers in the public and private sector. # A woman receives her second **coronavirus** **vaccination**, the Moderna vaccine, at the health center in Lagos, Nigeria Wednesday, |
| [911](https://www.english-corpora.org/corona/x4.asp?rs=&t=73384429&ID=21452039869) | [21-08-26 US](https://www.english-corpora.org/corona/x4.asp?rs=&t=73384429&ID=21452039869) | [wfmz.com](https://www.english-corpora.org/corona/x3.asp?node=&p=10&w10=coronavirus&w11=vaccination&r=) |  |  |  | in a third wave of the pandemic. # A man receives his second **coronavirus** **vaccination**, the Moderna vaccine, at the health center in Lagos, Nigeria Wednesday, |
| [912](https://www.english-corpora.org/corona/x4.asp?rs=&t=73384429&ID=21452042395) | [21-08-26 US](https://www.english-corpora.org/corona/x4.asp?rs=&t=73384429&ID=21452042395) | [wfmz.com](https://www.english-corpora.org/corona/x3.asp?node=&p=10&w10=coronavirus&w11=vaccination&r=) |  |  |  | . # Kemal Softic - stringer, AP # A woman receives her second **coronavirus** **vaccination**, the Moderna vaccine, at the health center in Lagos, Nigeria Wednesday, |
| [913](https://www.english-corpora.org/corona/x4.asp?rs=&t=73384429&ID=21452042528) | [21-08-26 US](https://www.english-corpora.org/corona/x4.asp?rs=&t=73384429&ID=21452042528) | [wfmz.com](https://www.english-corpora.org/corona/x3.asp?node=&p=10&w10=coronavirus&w11=vaccination&r=) |  |  |  | . # Sunday Alamba - staff, AP # A man receives his second **coronavirus** **vaccination**, the Moderna vaccine, at the health center in Lagos, Nigeria Wednesday, |
| [914](https://www.english-corpora.org/corona/x4.asp?rs=&t=73384429&ID=21452044057) | [21-08-26 US](https://www.english-corpora.org/corona/x4.asp?rs=&t=73384429&ID=21452044057) | [wfmz.com](https://www.english-corpora.org/corona/x3.asp?node=&p=10&w10=coronavirus&w11=vaccination&r=) |  |  |  | Las Vegas area won the $1 million grand prize Thursday to cap an eight-week **coronavirus** **vaccination** jackpot program. # Nevada Gov. Steve Sisolak created the program to boost enthusiasm |
| [915](https://www.english-corpora.org/corona/x4.asp?rs=&t=73390852&ID=21464564878) | [21-08-27 US](https://www.english-corpora.org/corona/x4.asp?rs=&t=73390852&ID=21464564878) | [washingtontimes.com](https://www.english-corpora.org/corona/x3.asp?node=&p=10&w10=coronavirus&w11=vaccination&r=) |  |  |  | to set off what they described as a " firework bomb " at a **coronavirus** **vaccination** center, prosecutors said Thursday. </p> <p>The man, whose identity was not... |
| [916](https://www.english-corpora.org/corona/x4.asp?rs=&t=87807031&ID=21422460983) | [21-08-23 US](https://www.english-corpora.org/corona/x4.asp?rs=&t=87807031&ID=21422460983) | [Associated Press](https://www.english-corpora.org/corona/x3.asp?node=&p=10&w10=coronavirus&w11=vaccination&r=) |  |  |  | The Oakland County woman has won the grand $2 million prize in Michigan's **coronavirus** **vaccination** sweepstakes. Christine Duval was announced as the winner Monday, Aug. 23, 2021 |
| [920](https://www.english-corpora.org/corona/x4.asp?rs=&t=87807031&ID=21422461093) | [21-08-23 US](https://www.english-corpora.org/corona/x4.asp?rs=&t=87807031&ID=21422461093) | [Associated Press](https://www.english-corpora.org/corona/x3.asp?node=&p=10&w10=coronavirus&w11=vaccination&r=) [(3)](https://www.english-corpora.org/corona/duplicates1.asp?xx=919&n=3) |  |  |  | Bloomfield Township woman who won the grand $2 million prize in Michigan's **coronavirus** **vaccination** sweepstakes said Monday that she and her husband will save money for their three kids |
| [922](https://www.english-corpora.org/corona/x4.asp?rs=&t=87815622&ID=21433806240) | [21-08-24 US](https://www.english-corpora.org/corona/x4.asp?rs=&t=87815622&ID=21433806240) | [The Boston Globe on MSN.com](https://www.english-corpora.org/corona/x3.asp?node=&p=10&w10=coronavirus&w11=vaccination&r=) [(1)](https://www.english-corpora.org/corona/duplicates1.asp?xx=921&n=1) |  |  |  | an ad shown to people Facebook thinks are college-educated conservatives in Oklahoma, **coronavirus** **vaccination** is touted as " trusted by the U.S. military... and by our |
| [924](https://www.english-corpora.org/corona/x4.asp?rs=&t=87815760&ID=21434852035) | [21-08-24 US](https://www.english-corpora.org/corona/x4.asp?rs=&t=87815760&ID=21434852035) | [The Washington Post on MSN.com](https://www.english-corpora.org/corona/x3.asp?node=&p=10&w10=coronavirus&w11=vaccination&r=) [(1)](https://www.english-corpora.org/corona/duplicates1.asp?xx=923&n=1) |  |  |  | # Disney Cruise Line announced Tuesday that it will begin requiring proof of **coronavirus** **vaccination** for trips to the Bahamas. # Passengers 12 and older will have to show |
| [925](https://www.english-corpora.org/corona/x4.asp?rs=&t=87817696&ID=21450053757) | [21-08-25 US](https://www.english-corpora.org/corona/x4.asp?rs=&t=87817696&ID=21450053757) | [ABCNews](https://www.english-corpora.org/corona/x3.asp?node=&p=10&w10=coronavirus&w11=vaccination&r=) |  |  |  | Garth Brooks' concert there Saturday took advantage of a chance to get a **coronavirus** **vaccination**. # The mobile vaccination clinic operated for four hours in the Arrowhead Stadium parking |
| [926](https://www.english-corpora.org/corona/x4.asp?rs=&t=87818302&ID=21446509709) | [21-08-25 US](https://www.english-corpora.org/corona/x4.asp?rs=&t=87818302&ID=21446509709) | [CNBC](https://www.english-corpora.org/corona/x3.asp?node=&p=10&w10=coronavirus&w11=vaccination&r=) |  |  |  | support of vaccine mandates at a moment when high-profile companies have informed employees that **coronavirus** **vaccination** requirements are in the works, and some localities have adopted or are contemplating vaccine |
| [927](https://www.english-corpora.org/corona/x4.asp?rs=&t=87818640&ID=21447749360) | [21-08-25 US](https://www.english-corpora.org/corona/x4.asp?rs=&t=87818640&ID=21447749360) | [YAHOO!News](https://www.english-corpora.org/corona/x3.asp?node=&p=10&w10=coronavirus&w11=vaccination&r=) |  |  |  | In an ad shown to people Facebook thinks are college-educated conservatives in Oklahoma, **coronavirus** **vaccination** is touted as " trusted by the U.S. military... and by our |
| [928](https://www.english-corpora.org/corona/x4.asp?rs=&t=87821140&ID=21447866545) | [21-08-25 US](https://www.english-corpora.org/corona/x4.asp?rs=&t=87821140&ID=21447866545) | [The Jerusalem Post Blogs](https://www.english-corpora.org/corona/x3.asp?node=&p=10&w10=coronavirus&w11=vaccination&r=) |  |  |  | had already received it as of Tuesday afternoon. # The Health Ministry's **coronavirus** **vaccination** committee voted by an almost unanimous majority of 56-1 last week to administer the third |
| [929](https://www.english-corpora.org/corona/x4.asp?rs=&t=87822160&ID=21447909602) | [21-08-25 US](https://www.english-corpora.org/corona/x4.asp?rs=&t=87822160&ID=21447909602) | [National Review](https://www.english-corpora.org/corona/x3.asp?node=&p=10&w10=coronavirus&w11=vaccination&r=) |  |  |  | Unvaccinated # A volunteer is injected with a vaccine as he participates in a **coronavirus** **vaccination** study at the Research Centers of America in Hollywood, Fla., September 24, |
| [930](https://www.english-corpora.org/corona/x4.asp?rs=&t=87825636&ID=21457270731) | [21-08-26 US](https://www.english-corpora.org/corona/x4.asp?rs=&t=87825636&ID=21457270731) | [New York Times](https://www.english-corpora.org/corona/x3.asp?node=&p=10&w10=coronavirus&w11=vaccination&r=) |  |  |  | at least 80 percent of employees had received at least one shot of a **coronavirus** **vaccination**, a threshold some big properties achieved. Last week, MGM Resorts went further |
| [931](https://www.english-corpora.org/corona/x4.asp?rs=&t=87826113&ID=21455358591) | [21-08-26 US](https://www.english-corpora.org/corona/x4.asp?rs=&t=87826113&ID=21455358591) | [Washington Post](https://www.english-corpora.org/corona/x3.asp?node=&p=10&w10=coronavirus&w11=vaccination&r=) |  |  |  | million as of Wednesday -- are projected to attend colleges that do not require **coronavirus** **vaccination**. Many schools without vaccine mandates are located in Republican-led states, reflecting political divisions |
| [932](https://www.english-corpora.org/corona/x4.asp?rs=&t=87828171&ID=21457935690) | [21-08-26 US](https://www.english-corpora.org/corona/x4.asp?rs=&t=87828171&ID=21457935690) | [The Washington Post on MSN.com](https://www.english-corpora.org/corona/x3.asp?node=&p=10&w10=coronavirus&w11=vaccination&r=) |  |  |  | shot of the Johnson & Johnson COVID-19 Vaccine. " It will require a **coronavirus** **vaccination** record card be shown to redeem the offer. # Although the incentive has been |
| [933](https://www.english-corpora.org/corona/x4.asp?rs=&t=87830669&ID=21471653771) | [21-08-27 US](https://www.english-corpora.org/corona/x4.asp?rs=&t=87830669&ID=21471653771) | [The Baltimore Sun](https://www.english-corpora.org/corona/x3.asp?node=&p=10&w10=coronavirus&w11=vaccination&r=) |  |  |  | rates fell an astounding 76 percent. The result is that, even as **coronavirus** **vaccination** rates climb, many Maryland children may be vulnerable to other dangerous vaccine-preventable illnesses. |
| [934](https://www.english-corpora.org/corona/x4.asp?rs=&t=87833620&ID=21467568013) | [21-08-27 US](https://www.english-corpora.org/corona/x4.asp?rs=&t=87833620&ID=21467568013) | [Evening Standard on MSN.com](https://www.english-corpora.org/corona/x3.asp?node=&p=10&w10=coronavirus&w11=vaccination&r=) |  |  |  | stressed the " vast majority of patients will be absolutely fine " following their **coronavirus** **vaccination**. # It comes after a coroner concluded that award-winning BBC radio presenter Lisa Shaw |
| [935](https://www.english-corpora.org/corona/x4.asp?rs=&t=87838537&ID=21477185903) | [21-08-28 US](https://www.english-corpora.org/corona/x4.asp?rs=&t=87838537&ID=21477185903) | [Medscape](https://www.english-corpora.org/corona/x3.asp?node=&p=10&w10=coronavirus&w11=vaccination&r=) |  |  |  | hit the ground running ". Forward Planning # The DHSC said the **coronavirus** **vaccination** programme has been so successful because plans were put in place before the medicines regulator |
| [936](https://www.english-corpora.org/corona/x4.asp?rs=&t=87838891&ID=21477703769) | [21-08-28 US](https://www.english-corpora.org/corona/x4.asp?rs=&t=87838891&ID=21477703769) | [Oregonian](https://www.english-corpora.org/corona/x3.asp?node=&p=10&w10=coronavirus&w11=vaccination&r=) |  |  |  | devastating floods and hurricanes. And they assisted medical teams at walk-in and drive-through **coronavirus** **vaccination** clinics earlier this year at the Jackson County Expo in Central Point. # Vaivao |
| [937](https://www.english-corpora.org/corona/x4.asp?rs=&t=87840631&ID=21485262931) | [21-08-29 US](https://www.english-corpora.org/corona/x4.asp?rs=&t=87840631&ID=21485262931) | [YAHOO!Finance](https://www.english-corpora.org/corona/x3.asp?node=&p=10&w10=coronavirus&w11=vaccination&r=) |  |  |  | . Photograph: Anadolu Agency/Getty Images A conservative radio host from Florida who criticised **coronavirus** **vaccination** efforts -- and called himself " Mr Anti-Vax " -- before contracting Covid-19 himself has |
| [938](https://www.english-corpora.org/corona/x4.asp?rs=&t=87846914&ID=21494382317) | [21-08-30 US](https://www.english-corpora.org/corona/x4.asp?rs=&t=87846914&ID=21494382317) | [New York Times](https://www.english-corpora.org/corona/x3.asp?node=&p=10&w10=coronavirus&w11=vaccination&r=) |  |  |  | 87846914 # When the United States Tennis Association announced on Friday that proof of **coronavirus** **vaccination** would be required for all spectators 12 and older to enter the grounds of the |
| [939](https://www.english-corpora.org/corona/x4.asp?rs=&t=87847395&ID=21497173546) | [21-08-30 US](https://www.english-corpora.org/corona/x4.asp?rs=&t=87847395&ID=21497173546) | [Forbes](https://www.english-corpora.org/corona/x3.asp?node=&p=10&w10=coronavirus&w11=vaccination&r=) |  |  |  | more than 100,000, similar to the peak reached during the winter. # **Coronavirus** **vaccination** proof in passport. # getty Reciprocity counts # The European list of safe |
| [940](https://www.english-corpora.org/corona/x4.asp?rs=&t=87852838&ID=21504121395) | [21-08-31 US](https://www.english-corpora.org/corona/x4.asp?rs=&t=87852838&ID=21504121395) | [Voice of America](https://www.english-corpora.org/corona/x3.asp?node=&p=10&w10=coronavirus&w11=vaccination&r=) |  |  |  | to get more initial vaccination doses. # FILE - A woman receives a **coronavirus** **vaccination** at the Kololo airstrip in Kampala, Uganda, May 31, 2021. # |
| [941](https://www.english-corpora.org/corona/x4.asp?rs=&t=87853301&ID=21501389750) | [21-08-31 US](https://www.english-corpora.org/corona/x4.asp?rs=&t=87853301&ID=21501389750) | [San Francisco Chronicle](https://www.english-corpora.org/corona/x3.asp?node=&p=10&w10=coronavirus&w11=vaccination&r=) |  |  |  | Hanford nuclear reservation in eastern Washington state who do not provide proof of a **coronavirus** **vaccination** will be required to be tested at least weekly to be allowed on the site |
| [942](https://www.english-corpora.org/corona/x4.asp?rs=&t=87853518&ID=21502612749) | [21-08-31 US](https://www.english-corpora.org/corona/x4.asp?rs=&t=87853518&ID=21502612749) | [The Motley Fool](https://www.english-corpora.org/corona/x3.asp?node=&p=10&w10=coronavirus&w11=vaccination&r=) |  |  |  | July and expects to report data in the fourth quarter. # Experts predict **coronavirus** **vaccination** may be annual. So it's likely Pfizer will generate billions of dollars in |
| [943](https://www.english-corpora.org/corona/x4.asp?rs=&t=53071720&ID=21428085143) | [21-08-24 CA](https://www.english-corpora.org/corona/x4.asp?rs=&t=53071720&ID=21428085143) | [chroniclejournal.com](https://www.english-corpora.org/corona/x3.asp?node=&p=10&w10=coronavirus&w11=vaccination&r=) |  |  |  | State University will require all students, faculty and staff to complete the full **coronavirus** **vaccination** process by Nov. 15. # School President Kristina Johnson said Tuesday that the requirement |
| [944](https://www.english-corpora.org/corona/x4.asp?rs=&t=53078964&ID=21452632228) | [21-08-26 CA](https://www.english-corpora.org/corona/x4.asp?rs=&t=53078964&ID=21452632228) | [570news.com](https://www.english-corpora.org/corona/x3.asp?node=&p=10&w10=coronavirus&w11=vaccination&r=) |  |  |  | 2021 at 9:27 am EDT # People are been screen before taking the second **coronavirus** **vaccination**, the Moderna vaccine at the health Centre in Lagos, Nigeria Wednesday, Aug |
| [945](https://www.english-corpora.org/corona/x4.asp?rs=&t=43373364&ID=21473237450) | [21-08-28 GB](https://www.english-corpora.org/corona/x4.asp?rs=&t=43373364&ID=21473237450) | [chroniclelive.co.uk](https://www.english-corpora.org/corona/x3.asp?node=&p=10&w10=coronavirus&w11=vaccination&r=) |  |  |  | hit the ground running ", Mirror reports. # The DHSC said the **coronavirus** **vaccination** programme has been so successful because plans were put in place before the medicines regulator |
| [946](https://www.english-corpora.org/corona/x4.asp?rs=&t=87834388&ID=21471859507) | [21-08-27 GB](https://www.english-corpora.org/corona/x4.asp?rs=&t=87834388&ID=21471859507) | [The Sun](https://www.english-corpora.org/corona/x3.asp?node=&p=10&w10=coronavirus&w11=vaccination&r=) |  |  |  | stressed the " vast majority of patients will be absolutely fine " following their **coronavirus** **vaccination**. # " It's very sad, and condolences to the family of Lisa |
| [947](https://www.english-corpora.org/corona/x4.asp?rs=&t=73376967&ID=21432782924) | [21-08-24 AU](https://www.english-corpora.org/corona/x4.asp?rs=&t=73376967&ID=21432782924) | [brisbanetimes.com.au](https://www.english-corpora.org/corona/x3.asp?node=&p=10&w10=coronavirus&w11=vaccination&r=) |  |  |  | authorities. # Ms Bromley said 1.3 million people had tried to call the **coronavirus** **vaccination** booking line on Wednesday morning and the booking site was receiving 50,000 hits per minute |
| [948](https://www.english-corpora.org/corona/x4.asp?rs=&t=73386365&ID=21463948019) | [21-08-27 AU](https://www.english-corpora.org/corona/x4.asp?rs=&t=73386365&ID=21463948019) | [examiner.com.au](https://www.english-corpora.org/corona/x3.asp?node=&p=10&w10=coronavirus&w11=vaccination&r=) |  |  |  | vaccination rollout will be expanded to all children aged 12 to 15 as daily case records |
| [949](https://www.english-corpora.org/corona/x4.asp?rs=&t=53086601&ID=21472377594) | [21-08-28 IN](https://www.english-corpora.org/corona/x4.asp?rs=&t=53086601&ID=21472377594) | [english.mathrubhumi.com](https://www.english-corpora.org/corona/x3.asp?node=&p=10&w10=coronavirus&w11=vaccination&r=) |  |  |  | administered a record 1,03,35,290 COVID-19 vaccine doses on Friday. With this, the **coronavirus** **vaccination** coverage in the country surpassed 62.29 crore, as per provisional reports till 7 am |
| [950](https://www.english-corpora.org/corona/x4.asp?rs=&t=73386683&ID=21466719014) | [21-08-27 IN](https://www.english-corpora.org/corona/x4.asp?rs=&t=73386683&ID=21466719014) | [economictimes.indiatimes.com](https://www.english-corpora.org/corona/x3.asp?node=&p=10&w10=coronavirus&w11=vaccination&r=) |  |  |  | . # " International flight operations will not be feasible in the future without **coronavirus** **vaccination** for aircraft crews, " the spokesperson said. # Lufthansa's subsidiary Swiss will |
| [951](https://www.english-corpora.org/corona/x4.asp?rs=&t=73392723&ID=21472809160) | [21-08-28 IN](https://www.english-corpora.org/corona/x4.asp?rs=&t=73392723&ID=21472809160) | [millenniumpost.in](https://www.english-corpora.org/corona/x3.asp?node=&p=10&w10=coronavirus&w11=vaccination&r=) |  |  |  | administered a record 1,03,35,290 COVID-19 vaccine doses on Friday. With this, the **coronavirus** **vaccination** coverage in the country surpassed 62.29 crore, as per provisional reports till 7 am |
| [953](https://www.english-corpora.org/corona/x4.asp?rs=&t=43378283&ID=21483920084) | [21-08-29 PK](https://www.english-corpora.org/corona/x4.asp?rs=&t=43378283&ID=21483920084) | [paktribune.com](https://www.english-corpora.org/corona/x3.asp?node=&p=10&w10=coronavirus&w11=vaccination&r=) [(1)](https://www.english-corpora.org/corona/duplicates1.asp?xx=952&n=1) |  |  |  | been tasked by Chief Minister Mahmood Khan with ensuring better public awareness of **coronavirus** **vaccination**. # He said the markets would remain closed for two days a week, |
| [954](https://www.english-corpora.org/corona/x4.asp?rs=&t=53075943&ID=21441988992) | [21-08-25 PK](https://www.english-corpora.org/corona/x4.asp?rs=&t=53075943&ID=21441988992) | [profit.pakistantoday.com.pk](https://www.english-corpora.org/corona/x3.asp?node=&p=10&w10=coronavirus&w11=vaccination&r=) |  |  |  | investor hopes that higher fuel demand would follow a potential step up in US **coronavirus** **vaccination** rates. # Brent crude oil futures gained 21 cents, or 0.3%, to |
| [955](https://www.english-corpora.org/corona/x4.asp?rs=&t=53085574&ID=21474151643) | [21-08-28 BD](https://www.english-corpora.org/corona/x4.asp?rs=&t=53085574&ID=21474151643) | [unb.com.bd](https://www.english-corpora.org/corona/x3.asp?node=&p=10&w10=coronavirus&w11=vaccination&r=) |  |  |  | Your COVID-19 **Coronavirus** **Vaccination** Protection Last? # UNB News # Publish- August 28, 2021, 01:59 PM |
| [956](https://www.english-corpora.org/corona/x4.asp?rs=&t=63271690&ID=21432482471) | [21-08-24 ZA](https://www.english-corpora.org/corona/x4.asp?rs=&t=63271690&ID=21432482471) | [dailymaverick.co.za](https://www.english-corpora.org/corona/x3.asp?node=&p=10&w10=coronavirus&w11=vaccination&r=) |  |  |  | # A resident has her vaccine administered at a drive-through Covid-19 **coronavirus** **vaccination** site in Pretoria, South Africa, on 18 August 2021. (Photo: |
| [957](https://www.english-corpora.org/corona/x4.asp?rs=&t=53070694&ID=21431941800) | [21-08-24 NG](https://www.english-corpora.org/corona/x4.asp?rs=&t=53070694&ID=21431941800) | [pmnewsnigeria.com](https://www.english-corpora.org/corona/x3.asp?node=&p=10&w10=coronavirus&w11=vaccination&r=) |  |  |  | announced this on Monday in Benin, while flagging off the second phase of **Coronavirus** **vaccination** exercise, adding that this became necessary to protect residents of the State. # |
| [958](https://www.english-corpora.org/corona/x4.asp?rs=&t=33184480&ID=21513285619) | [21-09-01 US](https://www.english-corpora.org/corona/x4.asp?rs=&t=33184480&ID=21513285619) | [nytimes.com](https://www.english-corpora.org/corona/x3.asp?node=&p=10&w10=coronavirus&w11=vaccination&r=) |  |  |  | shots or anything else? We'll ask the experts. # A pop-up **coronavirus** **vaccination** site in the South Bronx.Credit... James Estrin/The New York Times # By the staff |
| [959](https://www.english-corpora.org/corona/x4.asp?rs=&t=33184481&ID=21513285848) | [21-09-01 US](https://www.english-corpora.org/corona/x4.asp?rs=&t=33184481&ID=21513285848) | [nytimes.com](https://www.english-corpora.org/corona/x3.asp?node=&p=10&w10=coronavirus&w11=vaccination&r=) |  |  |  | 's fifth-highest Covid hospitalization rate; Mississippi has the third-highest. Both states have **coronavirus** **vaccination** rates well below 50 percent, and many of their hospitals were struggling with an |
| [960](https://www.english-corpora.org/corona/x4.asp?rs=&t=33199196&ID=21554821913) | [21-09-05 US](https://www.english-corpora.org/corona/x4.asp?rs=&t=33199196&ID=21554821913) | [dailynews.com](https://www.english-corpora.org/corona/x3.asp?node=&p=10&w10=coronavirus&w11=vaccination&r=) |  |  |  | People wait in line for the vaccine and testing is also available during a **Coronavirus** **vaccination** clinic at Rio Hondo College in Whittier on Wednesday, September 1, 2021. |
| [961](https://www.english-corpora.org/corona/x4.asp?rs=&t=33199976&ID=21554935588) | [21-09-05 US](https://www.english-corpora.org/corona/x4.asp?rs=&t=33199976&ID=21554935588) | [dailynews.com](https://www.english-corpora.org/corona/x3.asp?node=&p=10&w10=coronavirus&w11=vaccination&r=) |  |  |  | died. # Nurse Anita Huang of Curative prepares the Pfizer vaccine during a **coronavirus** **vaccination** clinic at Rio Hondo College in Whittier on Sept. 1. (Photo by Keith |
| [962](https://www.english-corpora.org/corona/x4.asp?rs=&t=53104711&ID=21522173265) | [21-09-02 US](https://www.english-corpora.org/corona/x4.asp?rs=&t=53104711&ID=21522173265) | [mynorthwest.com](https://www.english-corpora.org/corona/x3.asp?node=&p=10&w10=coronavirus&w11=vaccination&r=) |  |  |  | 's infectious diseases agency on Thursday urged countries to push ahead with their primary **coronavirus** **vaccination** programs and played down the need for booster shoots to ward off the delta variant |
| [963](https://www.english-corpora.org/corona/x4.asp?rs=&t=87855909&ID=21517727737) | [21-09-01 US](https://www.english-corpora.org/corona/x4.asp?rs=&t=87855909&ID=21517727737) | [Yahoo! Sports](https://www.english-corpora.org/corona/x3.asp?node=&p=10&w10=coronavirus&w11=vaccination&r=) |  |  |  | A mass resignation of Chicago Public Schools bus drivers refusing to abide by a **coronavirus** **vaccination** mandate led to a... The post Chicago Public Schools in talks with Uber, |
| [965](https://www.english-corpora.org/corona/x4.asp?rs=&t=87856441&ID=21517995576) | [21-09-01 US](https://www.english-corpora.org/corona/x4.asp?rs=&t=87856441&ID=21517995576) | [The Hill](https://www.english-corpora.org/corona/x3.asp?node=&p=10&w10=coronavirus&w11=vaccination&r=) [(1)](https://www.english-corpora.org/corona/duplicates1.asp?xx=964&n=1) |  |  |  | Urban Meyer said a player's **coronavirus** **vaccination** status was a factor in his staff's decisionmaking process when making cuts to the |
| [966](https://www.english-corpora.org/corona/x4.asp?rs=&t=87856507&ID=21517500600) | [21-09-01 US](https://www.english-corpora.org/corona/x4.asp?rs=&t=87856507&ID=21517500600) | [CNET](https://www.english-corpora.org/corona/x3.asp?node=&p=10&w10=coronavirus&w11=vaccination&r=) |  |  |  | COVID-19 cases and hospitalizations are clear, the reasons for people opting out of **coronavirus** **vaccination** isn't. # anyaivanova/Getty What the polls say # White evangelical Christians and |
| [967](https://www.english-corpora.org/corona/x4.asp?rs=&t=87858413&ID=21517029232) | [21-09-01 US](https://www.english-corpora.org/corona/x4.asp?rs=&t=87858413&ID=21517029232) | [Yahoo! Sports](https://www.english-corpora.org/corona/x3.asp?node=&p=10&w10=coronavirus&w11=vaccination&r=) |  |  |  | A mass resignation of Chicago Public Schools bus drivers refusing to abide by a **coronavirus** **vaccination** mandate led to a... The post Chicago Public Schools in talks with Uber, |
| [968](https://www.english-corpora.org/corona/x4.asp?rs=&t=87858555&ID=21518639400) | [21-09-01 US](https://www.english-corpora.org/corona/x4.asp?rs=&t=87858555&ID=21518639400) | [The Boston Globe](https://www.english-corpora.org/corona/x3.asp?node=&p=10&w10=coronavirus&w11=vaccination&r=) |  |  |  | programs, as well as home care workers will be required to get a **coronavirus** **vaccination** by Oct. 31, the Baker administration announced Wednesday. # The plan is part |
| [969](https://www.english-corpora.org/corona/x4.asp?rs=&t=87860878&ID=21520796458) | [21-09-01 US](https://www.english-corpora.org/corona/x4.asp?rs=&t=87860878&ID=21520796458) | [New York Times](https://www.english-corpora.org/corona/x3.asp?node=&p=10&w10=coronavirus&w11=vaccination&r=) |  |  |  | States for the fall, more than 500 universities have said they will require **coronavirus** **vaccination** this year. # But at dozens of universities with less stringent health requirements, |
| [970](https://www.english-corpora.org/corona/x4.asp?rs=&t=87863283&ID=21529316803) | [21-09-02 US](https://www.english-corpora.org/corona/x4.asp?rs=&t=87863283&ID=21529316803) | [Associated Press](https://www.english-corpora.org/corona/x3.asp?node=&p=10&w10=coronavirus&w11=vaccination&r=) |  |  |  | -- City employees in Tulsa are being offered a $250 stipend for receiving a **coronavirus** **vaccination**, plus a possible additional $250 if their department reaches vaccination goals. # The |
| [971](https://www.english-corpora.org/corona/x4.asp?rs=&t=87872311&ID=21537548886) | [21-09-03 US](https://www.english-corpora.org/corona/x4.asp?rs=&t=87872311&ID=21537548886) | [The Boston Globe on MSN.com](https://www.english-corpora.org/corona/x3.asp?node=&p=10&w10=coronavirus&w11=vaccination&r=) |  |  |  | programs, as well as home care workers will be required to get a **coronavirus** **vaccination** by Oct. 31, the Baker administration announced Wednesday. # The plan is part |
| [972](https://www.english-corpora.org/corona/x4.asp?rs=&t=87881299&ID=21560165339) | [21-09-05 US](https://www.english-corpora.org/corona/x4.asp?rs=&t=87881299&ID=21560165339) | [Forbes](https://www.english-corpora.org/corona/x3.asp?node=&p=10&w10=coronavirus&w11=vaccination&r=) |  |  |  | a quick rollout whenever the shots are approved. # A nurse marks a **coronavirus** **vaccination** card with a third " booster " dose of the Pfizer Covid-19... + vaccine |
| [973](https://www.english-corpora.org/corona/x4.asp?rs=&t=87881443&ID=21558145222) | [21-09-05 US](https://www.english-corpora.org/corona/x4.asp?rs=&t=87881443&ID=21558145222) | [Associated Press](https://www.english-corpora.org/corona/x3.asp?node=&p=10&w10=coronavirus&w11=vaccination&r=) |  |  |  | restrictions that largely prevented in-person classes, followed by some opposition to IU's **coronavirus** **vaccination** requirement for all students and employees. # ADVERTISEMENT # The Indiana University Purdue University |
| [978](https://www.english-corpora.org/corona/x4.asp?rs=&t=87887538&ID=21565408568) | [21-09-06 US](https://www.english-corpora.org/corona/x4.asp?rs=&t=87887538&ID=21565408568) | [Evening Standard on MSN.com](https://www.english-corpora.org/corona/x3.asp?node=&p=10&w10=coronavirus&w11=vaccination&r=) [(4)](https://www.english-corpora.org/corona/duplicates1.asp?xx=977&n=4) |  |  |  | " no hesitation at all " in allowing his children to have a **coronavirus** **vaccination**. # The clinical lead for Covid services at the Royal Devon & Exeter NHS |
| [979](https://www.english-corpora.org/corona/x4.asp?rs=&t=43383694&ID=21513672513) | [21-09-01 GB](https://www.english-corpora.org/corona/x4.asp?rs=&t=43383694&ID=21513672513) | [scotsman.com](https://www.english-corpora.org/corona/x3.asp?node=&p=10&w10=coronavirus&w11=vaccination&r=) |  |  |  | intensive care. # To date 4,106,408 have received their first dose of the **coronavirus** **vaccination**, while 3,680,761 have received their second dose. # On Friday last week Scotland |
| [980](https://www.english-corpora.org/corona/x4.asp?rs=&t=43386533&ID=21506872251) | [21-09-01 GB](https://www.english-corpora.org/corona/x4.asp?rs=&t=43386533&ID=21506872251) | [thestar.co.uk](https://www.english-corpora.org/corona/x3.asp?node=&p=10&w10=coronavirus&w11=vaccination&r=) |  |  |  | will mean members of the public will be required to show proof of a **coronavirus** **vaccination** to gain entry to domestic venues and events. # The Prime Minister's official |
| [981](https://www.english-corpora.org/corona/x4.asp?rs=&t=43389358&ID=21510781319) | [21-09-01 GB](https://www.english-corpora.org/corona/x4.asp?rs=&t=43389358&ID=21510781319) | [bristolpost.co.uk](https://www.english-corpora.org/corona/x3.asp?node=&p=10&w10=coronavirus&w11=vaccination&r=) |  |  |  | go and get the Covid jab. # In the earlier stages of the **coronavirus** **vaccination** programme, before it had been rolled out to younger women in great numbers, |
| [982](https://www.english-corpora.org/corona/x4.asp?rs=&t=43395431&ID=21530790691) | [21-09-03 GB](https://www.english-corpora.org/corona/x4.asp?rs=&t=43395431&ID=21530790691) | [politico.eu](https://www.english-corpora.org/corona/x3.asp?node=&p=10&w10=coronavirus&w11=vaccination&r=) |  |  |  | around the world. # VACCINATION ROLLOUT: We have also launched a brand-new **coronavirus** **vaccination** coverage tracker. It includes live data on the jab rollout in EU countries and |
| [983](https://www.english-corpora.org/corona/x4.asp?rs=&t=43407470&ID=21562132119) | [21-09-06 GB](https://www.english-corpora.org/corona/x4.asp?rs=&t=43407470&ID=21562132119) | [hulldailymail.co.uk](https://www.english-corpora.org/corona/x3.asp?node=&p=10&w10=coronavirus&w11=vaccination&r=) |  |  |  | # Play now # " Everyone has now had the chance to get a **coronavirus** **vaccination** and they've made their choice and there's consequences to that either way. |
| [984](https://www.english-corpora.org/corona/x4.asp?rs=&t=63312201&ID=21550942975) | [21-09-05 GB](https://www.english-corpora.org/corona/x4.asp?rs=&t=63312201&ID=21550942975) | [somersetlive.co.uk](https://www.english-corpora.org/corona/x3.asp?node=&p=10&w10=coronavirus&w11=vaccination&r=) |  |  |  | bed. " # It comes as a Government consultation on extending the mandatory **coronavirus** **vaccination** requirement to more health and care staff is expected shortly. # The Department of |
| [985](https://www.english-corpora.org/corona/x4.asp?rs=&t=63295598&ID=21515215405) | [21-09-01 IE](https://www.english-corpora.org/corona/x4.asp?rs=&t=63295598&ID=21515215405) | [intellinews.com](https://www.english-corpora.org/corona/x3.asp?node=&p=10&w10=coronavirus&w11=vaccination&r=) |  |  |  | 10.5% economic advance. According to the bank, despite the low rate of **coronavirus** **vaccination** at the moment in the country, a strong economic recovery is expected. The |
| [986](https://www.english-corpora.org/corona/x4.asp?rs=&t=63295598&ID=21515215638) | [21-09-01 IE](https://www.english-corpora.org/corona/x4.asp?rs=&t=63295598&ID=21515215638) | [intellinews.com](https://www.english-corpora.org/corona/x3.asp?node=&p=10&w10=coronavirus&w11=vaccination&r=) |  |  |  | more uncertain than usual, the Fund acknowledged, urging Georgia to increase its **coronavirus** **vaccination** rate. # The Georgian government has announced new coronavirus restrictions that will apply between |
| [987](https://www.english-corpora.org/corona/x4.asp?rs=&t=73412894&ID=21536771919) | [21-09-03 AU](https://www.english-corpora.org/corona/x4.asp?rs=&t=73412894&ID=21536771919) | [businessinsider.com.au](https://www.english-corpora.org/corona/x3.asp?node=&p=10&w10=coronavirus&w11=vaccination&r=) |  |  |  | reported being fully vaccinated against COVID-19. # Scammers and anti-vaxxers are hawking falsified **coronavirus** **vaccination** cards on social media platforms and digital forums. Prosecutors across the country, including |
| [988](https://www.english-corpora.org/corona/x4.asp?rs=&t=73413117&ID=21531996321) | [21-09-03 AU](https://www.english-corpora.org/corona/x4.asp?rs=&t=73413117&ID=21531996321) | [au.sports.yahoo.com](https://www.english-corpora.org/corona/x3.asp?node=&p=10&w10=coronavirus&w11=vaccination&r=) |  |  |  | full crowds will return to Victoria at some point next season but recognises high **coronavirus** **vaccination** rates will be crucial for that to happen. # On Tuesday, this year |
| [989](https://www.english-corpora.org/corona/x4.asp?rs=&t=73413298&ID=21536852557) | [21-09-03 AU](https://www.english-corpora.org/corona/x4.asp?rs=&t=73413298&ID=21536852557) | [gizmodo.com.au](https://www.english-corpora.org/corona/x3.asp?node=&p=10&w10=coronavirus&w11=vaccination&r=) |  |  |  | headlines after organisers announced that attendees would be required to either show proof of **coronavirus** **vaccination** or a negative covid-19 to enter the festival grounds. The U.S. has experienced a |
| [990](https://www.english-corpora.org/corona/x4.asp?rs=&t=73417181&ID=21555894670) | [21-09-05 AU](https://www.english-corpora.org/corona/x4.asp?rs=&t=73417181&ID=21555894670) | [smh.com.au](https://www.english-corpora.org/corona/x3.asp?node=&p=10&w10=coronavirus&w11=vaccination&r=) |  |  |  | Training spokesman said all Victorian teachers and school staff are now eligible for a **coronavirus** **vaccination**, " and we encourage them all to get the jab as soon as possible |
| [991](https://www.english-corpora.org/corona/x4.asp?rs=&t=73422592&ID=21562687072) | [21-09-06 AU](https://www.english-corpora.org/corona/x4.asp?rs=&t=73422592&ID=21562687072) | [canberratimes.com.au](https://www.english-corpora.org/corona/x3.asp?node=&p=10&w10=coronavirus&w11=vaccination&r=) |  |  |  | make **coronavirus** **vaccination** mandatory for many of its workers, but will be challenged over the possibility of |
| [992](https://www.english-corpora.org/corona/x4.asp?rs=&t=73418905&ID=21551246939) | [21-09-05 IN](https://www.english-corpora.org/corona/x4.asp?rs=&t=73418905&ID=21551246939) | [devdiscourse.com](https://www.english-corpora.org/corona/x3.asp?node=&p=10&w10=coronavirus&w11=vaccination&r=) |  |  |  | the ministry of health said on Sunday. Vietnam has one of the lowest **coronavirus** **vaccination** rates in the region, with only 3.3% of the country's 98 million people |
| [993](https://www.english-corpora.org/corona/x4.asp?rs=&t=53099046&ID=21511303657) | [21-09-01 PK](https://www.english-corpora.org/corona/x4.asp?rs=&t=53099046&ID=21511303657) | [samaa.tv](https://www.english-corpora.org/corona/x3.asp?node=&p=10&w10=coronavirus&w11=vaccination&r=) |  |  |  | in the national team's selection policy. # The Sindh government has made **coronavirus** **vaccination** mandatory for matriculation and intermediate students. # The murder trial of Parveen Rehman is |
| [994](https://www.english-corpora.org/corona/x4.asp?rs=&t=53101236&ID=21511578005) | [21-09-01 PK](https://www.english-corpora.org/corona/x4.asp?rs=&t=53101236&ID=21511578005) | [tribune.com.pk](https://www.english-corpora.org/corona/x3.asp?node=&p=10&w10=coronavirus&w11=vaccination&r=) |  |  |  | that the government had decided to take strict action to ensure success of the **coronavirus** **vaccination** campaign. # The district administration has been directed to initiate action against unvaccinated employees |
| [995](https://www.english-corpora.org/corona/x4.asp?rs=&t=53109723&ID=21542840707) | [21-09-04 PK](https://www.english-corpora.org/corona/x4.asp?rs=&t=53109723&ID=21542840707) | [samaa.tv](https://www.english-corpora.org/corona/x3.asp?node=&p=10&w10=coronavirus&w11=vaccination&r=) |  |  |  | PCR test 72 hours prior to departure. On September 1, Pakistan commenced **coronavirus** **vaccination** for people above the age of 17 years. It is mandatory for students to |
| [997](https://www.english-corpora.org/corona/x4.asp?rs=&t=43404227&ID=21550052883) | [21-09-05 MY](https://www.english-corpora.org/corona/x4.asp?rs=&t=43404227&ID=21550052883) | [malaymail.com](https://www.english-corpora.org/corona/x3.asp?node=&p=10&w10=coronavirus&w11=vaccination&r=) [(1)](https://www.english-corpora.org/corona/duplicates1.asp?xx=996&n=1) |  |  |  | of health said on Sunday. # Vietnam has one of the lowest **coronavirus** **vaccination** rates in the region, with only 3.3 per cent of the country's 98 |
| [998](https://www.english-corpora.org/corona/x4.asp?rs=&t=73419844&ID=21553861573) | [21-09-05 PH](https://www.english-corpora.org/corona/x4.asp?rs=&t=73419844&ID=21553861573) | [news.abs-cbn.com](https://www.english-corpora.org/corona/x3.asp?node=&p=10&w10=coronavirus&w11=vaccination&r=) |  |  |  | Vedado neighborhood on September 3, 2021. Cuba on Friday launched a nationwide **coronavirus** **vaccination** campaign for children and adolescents aged two to 18, a condition set by the |
| [999](https://www.english-corpora.org/corona/x4.asp?rs=&t=63304097&ID=21536490279) | [21-09-03 ZA](https://www.english-corpora.org/corona/x4.asp?rs=&t=63304097&ID=21536490279) | [politicsweb.co.za](https://www.english-corpora.org/corona/x3.asp?node=&p=10&w10=coronavirus&w11=vaccination&r=) |  |  |  | Economic Transformation (RET), and reserving exclusive government control of the national **coronavirus** **vaccination** programme, among others. # The national state of disaster with its lockdowns and |
| [1000](https://www.english-corpora.org/corona/x4.asp?rs=&t=53098131&ID=21507626045) | [21-09-01 NG](https://www.english-corpora.org/corona/x4.asp?rs=&t=53098131&ID=21507626045) | [punchng.com](https://www.english-corpora.org/corona/x3.asp?node=&p=10&w10=coronavirus&w11=vaccination&r=) |  |  |  | of Astrazeneca COVID-19 vaccines, totalling 100,154 doses ahead of the second phase of **coronavirus** **vaccination** in the state. # Announcing the number of doses received by the state, |

PAGE:   [**<<**](https://www.english-corpora.org/corona/x3.asp?node=&p=1&w10=coronavirus&w11=vaccination&r=)  [**<**](https://www.english-corpora.org/corona/x3.asp?node=&p=9&w10=coronavirus&w11=vaccination&r=)   10 / 42  [**>**](https://www.english-corpora.org/corona/x3.asp?node=&p=11&w10=coronavirus&w11=vaccination&r=)   [**>>**](https://www.english-corpora.org/corona/x3.asp?node=&p=42&w10=coronavirus&w11=vaccination&r=)

Конец формы

| FIND SAMPLE:   [100](https://www.english-corpora.org/corona/x3.asp?node=&sample=100&w10=coronavirus&w11=vaccination&r=)  [200](https://www.english-corpora.org/corona/x3.asp?node=&sample=200&w10=coronavirus&w11=vaccination&r=)  [500](https://www.english-corpora.org/corona/x3.asp?node=&sample=500&w10=coronavirus&w11=vaccination&r=)  [1000](https://www.english-corpora.org/corona/x3.asp?node=&sample=1000&w10=coronavirus&w11=vaccination&r=) PAGE:   [**<<**](https://www.english-corpora.org/corona/x3.asp?node=&p=1&w10=coronavirus&w11=vaccination&r=)  [**<**](https://www.english-corpora.org/corona/x3.asp?node=&p=10&w10=coronavirus&w11=vaccination&r=)   11 / 42  [**>**](https://www.english-corpora.org/corona/x3.asp?node=&p=12&w10=coronavirus&w11=vaccination&r=)   [**>>**](https://www.english-corpora.org/corona/x3.asp?node=&p=42&w10=coronavirus&w11=vaccination&r=) |  |
| --- | --- |

Начало формы

| CLICK FOR MORE CONTEXT | [**HELP**](javascript:newFeatures()) | [SAVE](javascript:chooser('s'))    [TRANSLATE](javascript:chooser('t'))    [ANALYZE](javascript:chooser('p')) |
| --- | --- | --- |

| [1001](https://www.english-corpora.org/corona/x4.asp?rs=&t=33111278&ID=21275897110) | [21-08-09 US](https://www.english-corpora.org/corona/x4.asp?rs=&t=33111278&ID=21275897110) | [forbes.com](https://www.english-corpora.org/corona/x3.asp?node=&p=11&w10=coronavirus&w11=vaccination&r=) |  |  |  | Cruise Line Holdings (NCLH) can require passengers to show documentation confirming their **coronavirus** **vaccination** status before boarding a ship. # In a preliminary ruling, U.S. District Judge |
| --- | --- | --- | --- | --- | --- | --- |
| [1002](https://www.english-corpora.org/corona/x4.asp?rs=&t=33116854&ID=21285566045) | [21-08-10 US](https://www.english-corpora.org/corona/x4.asp?rs=&t=33116854&ID=21285566045) | [sfchronicle.com](https://www.english-corpora.org/corona/x3.asp?node=&p=11&w10=coronavirus&w11=vaccination&r=) |  |  |  | the data on risk of preterm birth could inform the public health message on **coronavirus** **vaccination**. The Centers for Disease Control and Prevention cites experts saying the vaccines are unlikely |
| [1003](https://www.english-corpora.org/corona/x4.asp?rs=&t=33120862&ID=21297538994) | [21-08-11 US](https://www.english-corpora.org/corona/x4.asp?rs=&t=33120862&ID=21297538994) | [jpost.com](https://www.english-corpora.org/corona/x3.asp?node=&p=11&w10=coronavirus&w11=vaccination&r=) |  |  |  | had already received it as of Tuesday afternoon. # The Health Ministry's **coronavirus** **vaccination** committee voted by an almost unanimous majority of 56-1 last week to administer the third |
| [1004](https://www.english-corpora.org/corona/x4.asp?rs=&t=33133189&ID=21338656287) | [21-08-15 US](https://www.english-corpora.org/corona/x4.asp?rs=&t=33133189&ID=21338656287) | [aljazeera.com](https://www.english-corpora.org/corona/x3.asp?node=&p=11&w10=coronavirus&w11=vaccination&r=) |  |  |  | independence day turban, detailed his government's achievements and hailed the country's **coronavirus** **vaccination** campaign. # " Despite all the efforts, we have not been able to |
| [1005](https://www.english-corpora.org/corona/x4.asp?rs=&t=33137468&ID=21343604649) | [21-08-16 US](https://www.english-corpora.org/corona/x4.asp?rs=&t=33137468&ID=21343604649) | [jpost.com](https://www.english-corpora.org/corona/x3.asp?node=&p=11&w10=coronavirus&w11=vaccination&r=) |  |  |  | dose of COVID vaccine # The IDF began administering the third dose of the **coronavirus** **vaccination** on Monday to medical staff above the age of 30 and soldiers above the age |
| [1007](https://www.english-corpora.org/corona/x4.asp?rs=&t=33139708&ID=21343934152) | [21-08-16 US](https://www.english-corpora.org/corona/x4.asp?rs=&t=33139708&ID=21343934152) | [dailynews.com](https://www.english-corpora.org/corona/x3.asp?node=&p=11&w10=coronavirus&w11=vaccination&r=) [(1)](https://www.english-corpora.org/corona/duplicates1.asp?xx=1006&n=1) |  |  |  | its second year in September and require attendees to show proof of full **coronavirus** **vaccination** or negative COVID-19 test results. (File photo by Axel Koester, Contributing Photographer |
| [1008](https://www.english-corpora.org/corona/x4.asp?rs=&t=33139708&ID=21343934188) | [21-08-16 US](https://www.english-corpora.org/corona/x4.asp?rs=&t=33139708&ID=21343934188) | [dailynews.com](https://www.english-corpora.org/corona/x3.asp?node=&p=11&w10=coronavirus&w11=vaccination&r=) |  |  |  | in Redondo Beach has become the latest large-scale concert in Southern California to add **coronavirus** **vaccination** and testing guidelines. # The festival, which runs Sept. 10-12 at Seaside Lagoon |
| [1009](https://www.english-corpora.org/corona/x4.asp?rs=&t=33144585&ID=21371057103) | [21-08-18 US](https://www.english-corpora.org/corona/x4.asp?rs=&t=33144585&ID=21371057103) | [nytimes.com](https://www.english-corpora.org/corona/x3.asp?node=&p=11&w10=coronavirus&w11=vaccination&r=) |  |  |  | second chances, " said Dr. Matthew Harris, the medical director of the **coronavirus** **vaccination** program at Northwell Health, New York's largest hospital system. " What keeps |
| [1010](https://www.english-corpora.org/corona/x4.asp?rs=&t=33146816&ID=21366833831) | [21-08-18 US](https://www.english-corpora.org/corona/x4.asp?rs=&t=33146816&ID=21366833831) | [medicalxpress.com](https://www.english-corpora.org/corona/x3.asp?node=&p=11&w10=coronavirus&w11=vaccination&r=) |  |  |  | to the shot. # Appointments are no longer mandatory at any of the **coronavirus** **vaccination** sites run by New York City, including its newest and maybe coolest location: |
| [1011](https://www.english-corpora.org/corona/x4.asp?rs=&t=33161277&ID=21420836811) | [21-08-23 US](https://www.english-corpora.org/corona/x4.asp?rs=&t=33161277&ID=21420836811) | [jpost.com](https://www.english-corpora.org/corona/x3.asp?node=&p=11&w10=coronavirus&w11=vaccination&r=) |  |  |  | at Jerusalem's Misgav Ladach Hospital on June 6, as Israel began its **coronavirus** **vaccination** campaign for 12-to-15 year olds (credit: MARC ISRAEL SELLEM) # Woodcock said |
| [1012](https://www.english-corpora.org/corona/x4.asp?rs=&t=43310286&ID=21289338667) | [21-08-10 US](https://www.english-corpora.org/corona/x4.asp?rs=&t=43310286&ID=21289338667) | [khou.com](https://www.english-corpora.org/corona/x3.asp?node=&p=11&w10=coronavirus&w11=vaccination&r=) |  |  |  | plan is needed to encourage more people to get vaccinated. # The Statewide **Coronavirus** **Vaccination** Community Education and Engagement Taskforce is calling for the state and White House to reopen |
| [1013](https://www.english-corpora.org/corona/x4.asp?rs=&t=43331208&ID=21344209778) | [21-08-16 US](https://www.english-corpora.org/corona/x4.asp?rs=&t=43331208&ID=21344209778) | [news4jax.com](https://www.english-corpora.org/corona/x3.asp?node=&p=11&w10=coronavirus&w11=vaccination&r=) |  |  |  | " # He said he had hoped to stay on until the country's **coronavirus** **vaccination** program is completed and the economy has recovered, but was thwarted by those " |
| [1014](https://www.english-corpora.org/corona/x4.asp?rs=&t=43331623&ID=21344270971) | [21-08-16 US](https://www.english-corpora.org/corona/x4.asp?rs=&t=43331623&ID=21344270971) | [ktar.com](https://www.english-corpora.org/corona/x3.asp?node=&p=11&w10=coronavirus&w11=vaccination&r=) |  |  |  | are calling for Prayuth's resignation over his perceived bungling of the government's **coronavirus** **vaccination** program. Infections have surged in the past few weeks while vaccination rates remain low |
| [1015](https://www.english-corpora.org/corona/x4.asp?rs=&t=53031160&ID=21298528457) | [21-08-11 US](https://www.english-corpora.org/corona/x4.asp?rs=&t=53031160&ID=21298528457) | [pix11.com](https://www.english-corpora.org/corona/x3.asp?node=&p=11&w10=coronavirus&w11=vaccination&r=) |  |  |  | # A man receives a third Pfizer-BioNTech COVID-19 vaccine from medical staff at a **coronavirus** **vaccination** center in Tel Aviv, Israel, Tuesday, Aug. 10, 2021. Israel |
| [1016](https://www.english-corpora.org/corona/x4.asp?rs=&t=63223783&ID=21290195761) | [21-08-10 US](https://www.english-corpora.org/corona/x4.asp?rs=&t=63223783&ID=21290195761) | [standardspeaker.com](https://www.english-corpora.org/corona/x3.asp?node=&p=11&w10=coronavirus&w11=vaccination&r=) |  |  |  | support of vaccine mandates at a moment when high-profile companies have informed employees that **coronavirus** **vaccination** requirements are in the works, and some localities have adopted or are contemplating vaccine |
| [1017](https://www.english-corpora.org/corona/x4.asp?rs=&t=63227589&ID=21300893169) | [21-08-11 US](https://www.english-corpora.org/corona/x4.asp?rs=&t=63227589&ID=21300893169) | [cointelegraph.com](https://www.english-corpora.org/corona/x3.asp?node=&p=11&w10=coronavirus&w11=vaccination&r=) |  |  |  | Total shares # Listen to article # 0:00 # News # With counterfeit Australian **coronavirus** **vaccination** certificates circulating online, local experts are calling for the national rollout of a blockchain-based |
| [1018](https://www.english-corpora.org/corona/x4.asp?rs=&t=87729709&ID=21279618253) | [21-08-09 US](https://www.english-corpora.org/corona/x4.asp?rs=&t=87729709&ID=21279618253) | [San Francisco Chronicle](https://www.english-corpora.org/corona/x3.asp?node=&p=11&w10=coronavirus&w11=vaccination&r=) |  |  |  | The SoMa offices of Fast are open for employees who show proof of a **coronavirus** **vaccination**. But with remote working now the norm, the space is much changed. |
| [1019](https://www.english-corpora.org/corona/x4.asp?rs=&t=87734307&ID=21291870595) | [21-08-10 US](https://www.english-corpora.org/corona/x4.asp?rs=&t=87734307&ID=21291870595) | [YAHOO!News](https://www.english-corpora.org/corona/x3.asp?node=&p=11&w10=coronavirus&w11=vaccination&r=) |  |  |  | Ron DeSantis and require that passengers leaving from the state show written proof of **coronavirus** **vaccination** before they board a ship starting on Aug. 15. # " We are pleased |
| [1020](https://www.english-corpora.org/corona/x4.asp?rs=&t=87738316&ID=21292026974) | [21-08-10 US](https://www.english-corpora.org/corona/x4.asp?rs=&t=87738316&ID=21292026974) | [SFGate](https://www.english-corpora.org/corona/x3.asp?node=&p=11&w10=coronavirus&w11=vaccination&r=) |  |  |  | # 9of1410of14A man receives a third Pfizer-BioNTech COVID-19 vaccine from medical staff at a **coronavirus** **vaccination** center in Tel Aviv, Israel, Tuesday, Aug. 10, 2021. Israel |
| [1021](https://www.english-corpora.org/corona/x4.asp?rs=&t=87738316&ID=21292028317) | [21-08-10 US](https://www.english-corpora.org/corona/x4.asp?rs=&t=87738316&ID=21292028317) | [SFGate](https://www.english-corpora.org/corona/x3.asp?node=&p=11&w10=coronavirus&w11=vaccination&r=) |  |  |  | Garth Brooks' concert there Saturday took advantage of a chance to get a **coronavirus** **vaccination**. # The mobile vaccination clinic operated for four hours in the Arrowhead Stadium parking |
| [1022](https://www.english-corpora.org/corona/x4.asp?rs=&t=87740492&ID=21304592295) | [21-08-11 US](https://www.english-corpora.org/corona/x4.asp?rs=&t=87740492&ID=21304592295) | [New York Post](https://www.english-corpora.org/corona/x3.asp?node=&p=11&w10=coronavirus&w11=vaccination&r=) |  |  |  | that interact regularly with the public have gotten at least one shot of a **coronavirus** **vaccination** -- a pace that lags far behind the Big Apple's overall average, figures |
| [1023](https://www.english-corpora.org/corona/x4.asp?rs=&t=87743831&ID=21301692051) | [21-08-11 US](https://www.english-corpora.org/corona/x4.asp?rs=&t=87743831&ID=21301692051) | [StarTribune](https://www.english-corpora.org/corona/x3.asp?node=&p=11&w10=coronavirus&w11=vaccination&r=) |  |  |  | he does not plan to require the use of masks in schools though the **coronavirus** **vaccination** rate in Mississippi remains among the lowest in the nation. Health official say they |
| [1024](https://www.english-corpora.org/corona/x4.asp?rs=&t=87745328&ID=21313376905) | [21-08-12 US](https://www.english-corpora.org/corona/x4.asp?rs=&t=87745328&ID=21313376905) | [YAHOO!Finance](https://www.english-corpora.org/corona/x3.asp?node=&p=11&w10=coronavirus&w11=vaccination&r=) |  |  |  | Follow her on Twitter aarthiswami. # Christy Foreman pulled out of the drive-through **coronavirus** **vaccination** site in March, a jab of Johnson & Johnson in her arm, and |
| [1026](https://www.english-corpora.org/corona/x4.asp?rs=&t=87745855&ID=21314211555) | [21-08-12 US](https://www.english-corpora.org/corona/x4.asp?rs=&t=87745855&ID=21314211555) | [New York Post](https://www.english-corpora.org/corona/x3.asp?node=&p=11&w10=coronavirus&w11=vaccination&r=) [(1)](https://www.english-corpora.org/corona/duplicates1.asp?xx=1025&n=1) |  |  |  | Mayor Bill de Blasio's cash-for-shots incentives program as the Big Apple's **coronavirus** **vaccination** effort picks up steam. # That means the city has already given away a |
| [1027](https://www.english-corpora.org/corona/x4.asp?rs=&t=87746393&ID=21315526013) | [21-08-12 US](https://www.english-corpora.org/corona/x4.asp?rs=&t=87746393&ID=21315526013) | [Forbes](https://www.english-corpora.org/corona/x3.asp?node=&p=11&w10=coronavirus&w11=vaccination&r=) |  |  |  | the last week. Tangent # Zients said there will be no federal **coronavirus** **vaccination** database, noting the information is stored at the state and local level. He |
| [1028](https://www.english-corpora.org/corona/x4.asp?rs=&t=87746416&ID=21313128772) | [21-08-12 US](https://www.english-corpora.org/corona/x4.asp?rs=&t=87746416&ID=21313128772) | [YAHOO!News](https://www.english-corpora.org/corona/x3.asp?node=&p=11&w10=coronavirus&w11=vaccination&r=) |  |  |  | . # A woman rests after receiving a third Pfizer-BioNTech COVID-19 vaccine at a **coronavirus** **vaccination** center in Tel Aviv, Israel, Tuesday, Aug. 10, 2021. Israel |
| [1029](https://www.english-corpora.org/corona/x4.asp?rs=&t=87747151&ID=21313703193) | [21-08-12 US](https://www.english-corpora.org/corona/x4.asp?rs=&t=87747151&ID=21313703193) | [Yahoo! Sports](https://www.english-corpora.org/corona/x3.asp?node=&p=11&w10=coronavirus&w11=vaccination&r=) |  |  |  | said at a news conference. # Christy Foreman pulled out of the drive-through **coronavirus** **vaccination** site in March, a jab of Johnson & Johnson in her arm, and |
| [1033](https://www.english-corpora.org/corona/x4.asp?rs=&t=87752589&ID=21327587060) | [21-08-13 US](https://www.english-corpora.org/corona/x4.asp?rs=&t=87752589&ID=21327587060) | [CBS News](https://www.english-corpora.org/corona/x3.asp?node=&p=11&w10=coronavirus&w11=vaccination&r=) [(3)](https://www.english-corpora.org/corona/duplicates1.asp?xx=1032&n=3) |  |  |  | to ICE, releases them or deports them, has not been offering **coronavirus** **vaccination** to people in its custody, according to the Department of Homeland Security. # |
| [1034](https://www.english-corpora.org/corona/x4.asp?rs=&t=87752589&ID=21327587213) | [21-08-13 US](https://www.english-corpora.org/corona/x4.asp?rs=&t=87752589&ID=21327587213) | [CBS News](https://www.english-corpora.org/corona/x3.asp?node=&p=11&w10=coronavirus&w11=vaccination&r=) |  |  |  | housing children who cross the southern border without their parents, has been offering **coronavirus** **vaccination** to eligible migrant teenagers in its care. # As of July 12, more |
| [1035](https://www.english-corpora.org/corona/x4.asp?rs=&t=87757097&ID=21335151884) | [21-08-14 US](https://www.english-corpora.org/corona/x4.asp?rs=&t=87757097&ID=21335151884) | [SFGate](https://www.english-corpora.org/corona/x3.asp?node=&p=11&w10=coronavirus&w11=vaccination&r=) |  |  |  | , Modi also listed his government's achievements since 2014 and hailed India's **coronavirus** **vaccination** campaign. # " We are proud that we didn't have to depend on |
| [1036](https://www.english-corpora.org/corona/x4.asp?rs=&t=87757389&ID=21335394341) | [21-08-14 US](https://www.english-corpora.org/corona/x4.asp?rs=&t=87757389&ID=21335394341) | [Yahoo! Sports](https://www.english-corpora.org/corona/x3.asp?node=&p=11&w10=coronavirus&w11=vaccination&r=) |  |  |  | New York City. Mayor Bill de Blasio announced last week that proof of **coronavirus** **vaccination** will be required to attend indoor restaurants, gyms, and entertainment venues. New |
| [1037](https://www.english-corpora.org/corona/x4.asp?rs=&t=87757980&ID=21334623326) | [21-08-14 US](https://www.english-corpora.org/corona/x4.asp?rs=&t=87757980&ID=21334623326) | [Associated Press](https://www.english-corpora.org/corona/x3.asp?node=&p=11&w10=coronavirus&w11=vaccination&r=) |  |  |  | , Modi also listed his government's achievements since 2014 and hailed India's **coronavirus** **vaccination** campaign. # ADVERTISEMENT # " We are proud that we didn't have to |
| [1038](https://www.english-corpora.org/corona/x4.asp?rs=&t=87759756&ID=21334315161) | [21-08-14 US](https://www.english-corpora.org/corona/x4.asp?rs=&t=87759756&ID=21334315161) | [The Washington Post on MSN.com](https://www.english-corpora.org/corona/x3.asp?node=&p=11&w10=coronavirus&w11=vaccination&r=) |  |  |  | by conservative advocacy organization Liberty Counsel, this week staged national walkouts to protest **coronavirus** **vaccination** requirements. The San Francisco Deputy Sheriffs' Association this month posted a message on |
| [1039](https://www.english-corpora.org/corona/x4.asp?rs=&t=87759800&ID=21332728355) | [21-08-14 US](https://www.english-corpora.org/corona/x4.asp?rs=&t=87759800&ID=21332728355) | [ABC](https://www.english-corpora.org/corona/x3.asp?node=&p=11&w10=coronavirus&w11=vaccination&r=) |  |  |  | , Modi also listed his government's achievements since 2014 and hailed India's **coronavirus** **vaccination** campaign. # " We are proud that we didn't have to depend on |
| [1040](https://www.english-corpora.org/corona/x4.asp?rs=&t=87760553&ID=21333902782) | [21-08-14 US](https://www.english-corpora.org/corona/x4.asp?rs=&t=87760553&ID=21333902782) | [The Washington Post on MSN.com](https://www.english-corpora.org/corona/x3.asp?node=&p=11&w10=coronavirus&w11=vaccination&r=) |  |  |  | small number of countries in the world that had yet to roll out a **coronavirus** **vaccination** campaign -- and the only country in the Americas. Meanwhile, coronavirus cases have |
| [1041](https://www.english-corpora.org/corona/x4.asp?rs=&t=87761133&ID=21339904544) | [21-08-15 US](https://www.english-corpora.org/corona/x4.asp?rs=&t=87761133&ID=21339904544) | [Associated Press](https://www.english-corpora.org/corona/x3.asp?node=&p=11&w10=coronavirus&w11=vaccination&r=) |  |  |  | , Modi also listed his government's achievements since 2014 and hailed India's **coronavirus** **vaccination** campaign. # ADVERTISEMENT # " We are proud that we didn't have to |
| [1042](https://www.english-corpora.org/corona/x4.asp?rs=&t=87761773&ID=21342378573) | [21-08-15 US](https://www.english-corpora.org/corona/x4.asp?rs=&t=87761773&ID=21342378573) | [ABC](https://www.english-corpora.org/corona/x3.asp?node=&p=11&w10=coronavirus&w11=vaccination&r=) |  |  |  | are calling for Prayuth's resignation over his perceived bungling of the government's **coronavirus** **vaccination** program. Thailand has seen infection rates surge in the past few weeks while vaccination |
| [1044](https://www.english-corpora.org/corona/x4.asp?rs=&t=87764011&ID=21339768436) | [21-08-15 US](https://www.english-corpora.org/corona/x4.asp?rs=&t=87764011&ID=21339768436) | [San Francisco Chronicle](https://www.english-corpora.org/corona/x3.asp?node=&p=11&w10=coronavirus&w11=vaccination&r=) [(1)](https://www.english-corpora.org/corona/duplicates1.asp?xx=1043&n=1) |  |  |  | Modi also listed his government's achievements since 2014 and hailed India's **coronavirus** **vaccination** campaign. # " We are proud that we didn't have to depend on |
| [1045](https://www.english-corpora.org/corona/x4.asp?rs=&t=87767433&ID=21350918664) | [21-08-16 US](https://www.english-corpora.org/corona/x4.asp?rs=&t=87767433&ID=21350918664) | [The Boston Globe on MSN.com](https://www.english-corpora.org/corona/x3.asp?node=&p=11&w10=coronavirus&w11=vaccination&r=) |  |  |  | are calling for Prayuth's resignation over his perceived bungling of the government's **coronavirus** **vaccination** program. Infections have surged in the past few weeks while vaccination rates remain low |
| [1046](https://www.english-corpora.org/corona/x4.asp?rs=&t=87769610&ID=21350709914) | [21-08-16 US](https://www.english-corpora.org/corona/x4.asp?rs=&t=87769610&ID=21350709914) | [Greenwich Time](https://www.english-corpora.org/corona/x3.asp?node=&p=11&w10=coronavirus&w11=vaccination&r=) |  |  |  | Zealand has first local virus case in months # 1of18A woman receives the second **coronavirus** **vaccination**, the Moderna vaccine at the Federal Medical Centre in Abuja, Nigeria Monday, |
| [1047](https://www.english-corpora.org/corona/x4.asp?rs=&t=87769610&ID=21350711746) | [21-08-16 US](https://www.english-corpora.org/corona/x4.asp?rs=&t=87769610&ID=21350711746) | [Greenwich Time](https://www.english-corpora.org/corona/x3.asp?node=&p=11&w10=coronavirus&w11=vaccination&r=) |  |  |  | six months. # # HONOLULU -- Some private schools in Hawaii are mandating **coronavirus** **vaccination** for students, faculty and staff. # The Honolulu Star-Advertiser says Parker School in |
| [1048](https://www.english-corpora.org/corona/x4.asp?rs=&t=87772043&ID=21363593111) | [21-08-17 US](https://www.english-corpora.org/corona/x4.asp?rs=&t=87772043&ID=21363593111) | [Associated Press](https://www.english-corpora.org/corona/x3.asp?node=&p=11&w10=coronavirus&w11=vaccination&r=) |  |  |  | become seriously ill. # Some primary care providers on the front lines of **coronavirus** **vaccination** and testing are also reporting serious staffing shortages. # Primary Health Medical Group's |
| [1049](https://www.english-corpora.org/corona/x4.asp?rs=&t=87773710&ID=21361779205) | [21-08-17 US](https://www.english-corpora.org/corona/x4.asp?rs=&t=87773710&ID=21361779205) | [The Washington Post on MSN.com](https://www.english-corpora.org/corona/x3.asp?node=&p=11&w10=coronavirus&w11=vaccination&r=) |  |  |  | coronavirus **vaccination** or weekly testing for employees # Metro will require employees to be vaccinated against the |
| [1050](https://www.english-corpora.org/corona/x4.asp?rs=&t=87773976&ID=21365810119) | [21-08-17 US](https://www.english-corpora.org/corona/x4.asp?rs=&t=87773976&ID=21365810119) | [The Baltimore Sun](https://www.english-corpora.org/corona/x3.asp?node=&p=11&w10=coronavirus&w11=vaccination&r=) |  |  |  | to get COVID vaccine, state health secretary says # In order to improve **coronavirus** **vaccination** rates, Maryland's top health official said Tuesday that he's hoping employers will |
| [1051](https://www.english-corpora.org/corona/x4.asp?rs=&t=87775639&ID=21364714966) | [21-08-17 US](https://www.english-corpora.org/corona/x4.asp?rs=&t=87775639&ID=21364714966) | [New York Times](https://www.english-corpora.org/corona/x3.asp?node=&p=11&w10=coronavirus&w11=vaccination&r=) |  |  |  | second chances, " said Dr. Matthew Harris, the medical director of the **coronavirus** **vaccination** program at Northwell Health, New York's largest hospital system. " What keeps |
| [1052](https://www.english-corpora.org/corona/x4.asp?rs=&t=87776285&ID=21365616035) | [21-08-17 US](https://www.english-corpora.org/corona/x4.asp?rs=&t=87776285&ID=21365616035) | [Voice of America](https://www.english-corpora.org/corona/x3.asp?node=&p=11&w10=coronavirus&w11=vaccination&r=) |  |  |  | to get more initial vaccination doses. # FILE - A woman receives a **coronavirus** **vaccination** at the Kololo airstrip in Kampala, Uganda, May 31, 2021. # |
| [1053](https://www.english-corpora.org/corona/x4.asp?rs=&t=87776641&ID=21363508546) | [21-08-17 US](https://www.english-corpora.org/corona/x4.asp?rs=&t=87776641&ID=21363508546) | [WSMV](https://www.english-corpora.org/corona/x3.asp?node=&p=11&w10=coronavirus&w11=vaccination&r=) |  |  |  | quarantined. TSU is also working with various agencies to assist in providing the **coronavirus** **vaccination**. # " We understand that some students and employees have not received a COVID-19 |
| [1054](https://www.english-corpora.org/corona/x4.asp?rs=&t=87778271&ID=21365135846) | [21-08-17 US](https://www.english-corpora.org/corona/x4.asp?rs=&t=87778271&ID=21365135846) | [New York Daily News](https://www.english-corpora.org/corona/x3.asp?node=&p=11&w10=coronavirus&w11=vaccination&r=) |  |  |  | live performance indoors in New York City will soon have to show proof of **coronavirus** **vaccination** or be denied entry, Mayor de Blasio announced Tuesday in his most aggressive push |
| [1055](https://www.english-corpora.org/corona/x4.asp?rs=&t=87786136&ID=21387294754) | [21-08-19 US](https://www.english-corpora.org/corona/x4.asp?rs=&t=87786136&ID=21387294754) | [New York Times](https://www.english-corpora.org/corona/x3.asp?node=&p=11&w10=coronavirus&w11=vaccination&r=) |  |  |  | an executive order directing the county's 110,000 employees to show proof of a **coronavirus** **vaccination** by Oct. 1. While exemptions will be made for medical and religious reasons, |
| [1056](https://www.english-corpora.org/corona/x4.asp?rs=&t=87787036&ID=21387334080) | [21-08-19 US](https://www.english-corpora.org/corona/x4.asp?rs=&t=87787036&ID=21387334080) | [New York Times](https://www.english-corpora.org/corona/x3.asp?node=&p=11&w10=coronavirus&w11=vaccination&r=) |  |  |  | effort in Russian-aligned disinformation: a campaign that taps into skepticism and fears of **coronavirus** **vaccination** to not just undermine the effort to immunize people but also try to falsely link |
| [1058](https://www.english-corpora.org/corona/x4.asp?rs=&t=87788790&ID=21387918244) | [21-08-19 US](https://www.english-corpora.org/corona/x4.asp?rs=&t=87788790&ID=21387918244) | [New York Times](https://www.english-corpora.org/corona/x3.asp?node=&p=11&w10=coronavirus&w11=vaccination&r=) [(1)](https://www.english-corpora.org/corona/duplicates1.asp?xx=1057&n=1) |  |  |  | pressing private sector leaders to adopt a new set of recommendations to maximize **coronavirus** **vaccination** among their employees. # " You have a key role to play in our |
| [1059](https://www.english-corpora.org/corona/x4.asp?rs=&t=87792281&ID=21397199013) | [21-08-20 US](https://www.english-corpora.org/corona/x4.asp?rs=&t=87792281&ID=21397199013) | [New York Times](https://www.english-corpora.org/corona/x3.asp?node=&p=11&w10=coronavirus&w11=vaccination&r=) |  |  |  | understanding. " # White House officials have also been encouraging pediatricians to incorporate **coronavirus** **vaccination** into back-to-school sports physicals. Many districts are offering the shots during sports practice, |
| [1060](https://www.english-corpora.org/corona/x4.asp?rs=&t=87795848&ID=21404289254) | [21-08-21 US](https://www.english-corpora.org/corona/x4.asp?rs=&t=87795848&ID=21404289254) | [YAHOO!News](https://www.english-corpora.org/corona/x3.asp?node=&p=11&w10=coronavirus&w11=vaccination&r=) |  |  |  | Please try again later. # WASHINGTON - The Pentagon's effort to mandate **coronavirus** **vaccination** for all 1.3 million active-duty service members will continue to face resistance from a segment |
| [1061](https://www.english-corpora.org/corona/x4.asp?rs=&t=87797153&ID=21403609935) | [21-08-21 US](https://www.english-corpora.org/corona/x4.asp?rs=&t=87797153&ID=21403609935) | [Washington Post](https://www.english-corpora.org/corona/x3.asp?node=&p=11&w10=coronavirus&w11=vaccination&r=) |  |  |  | Apley's hospitalization and death showcased the bitterness of the country's divide over **coronavirus** **vaccination**, and over how to bridge it, as the pandemic makes personal tragedy inseparable |
| [1062](https://www.english-corpora.org/corona/x4.asp?rs=&t=87800745&ID=21415120975) | [21-08-22 US](https://www.english-corpora.org/corona/x4.asp?rs=&t=87800745&ID=21415120975) | [YAHOO!News](https://www.english-corpora.org/corona/x3.asp?node=&p=11&w10=coronavirus&w11=vaccination&r=) |  |  |  | ruling United Russia party. # WASHINGTON - The Pentagon's effort to mandate **coronavirus** **vaccination** for all 1.3 million active-duty service members will continue to face resistance from a segment |
| [1063](https://www.english-corpora.org/corona/x4.asp?rs=&t=87805875&ID=21426226738) | [21-08-23 US](https://www.english-corpora.org/corona/x4.asp?rs=&t=87805875&ID=21426226738) | [YAHOO!News](https://www.english-corpora.org/corona/x3.asp?node=&p=11&w10=coronavirus&w11=vaccination&r=) |  |  |  | investor hopes that higher fuel demand would follow a potential step up in U.S. **coronavirus** **vaccination** rates. Brent crude oil futures gained 21 cents, or 0.3%, to $68.96 |
| [1066](https://www.english-corpora.org/corona/x4.asp?rs=&t=53022523&ID=21271621400) | [21-08-09 CA](https://www.english-corpora.org/corona/x4.asp?rs=&t=53022523&ID=21271621400) | [ca.news.yahoo.com](https://www.english-corpora.org/corona/x3.asp?node=&p=11&w10=coronavirus&w11=vaccination&r=) [(2)](https://www.english-corpora.org/corona/duplicates1.asp?xx=1065&n=2) |  |  |  | allowed Norwegian Cruise Line Holdings to demand that passengers show written proof of **coronavirus** **vaccination** before they board a ship, dealing a major blow to Florida Governor Ron DeSantis |
| [1067](https://www.english-corpora.org/corona/x4.asp?rs=&t=53046863&ID=21347159966) | [21-08-16 CA](https://www.english-corpora.org/corona/x4.asp?rs=&t=53046863&ID=21347159966) | [chroniclejournal.com](https://www.english-corpora.org/corona/x3.asp?node=&p=11&w10=coronavirus&w11=vaccination&r=) |  |  |  | are calling for Prayuth's resignation over his perceived bungling of the government's **coronavirus** **vaccination** program. Infections have surged in the past few weeks while vaccination rates remain low |
| [1068](https://www.english-corpora.org/corona/x4.asp?rs=&t=33154648&ID=21399911536) | [21-08-21 GB](https://www.english-corpora.org/corona/x4.asp?rs=&t=33154648&ID=21399911536) | [edition.cnn.com](https://www.english-corpora.org/corona/x3.asp?node=&p=11&w10=coronavirus&w11=vaccination&r=) |  |  |  | # (END VIDEOTAPE) # HOLMES: Canada has one of the highest **coronavirus** **vaccination** rates in the world but for the past two weeks, new cases in the |
| [1070](https://www.english-corpora.org/corona/x4.asp?rs=&t=43342116&ID=21378583955) | [21-08-19 GB](https://www.english-corpora.org/corona/x4.asp?rs=&t=43342116&ID=21378583955) | [dorsetecho.co.uk](https://www.english-corpora.org/corona/x3.asp?node=&p=11&w10=coronavirus&w11=vaccination&r=) [(1)](https://www.english-corpora.org/corona/duplicates1.asp?xx=1069&n=1) |  |  |  | certificate is used to show a person has had both doses of a **coronavirus** **vaccination** and are available to everyone in the UK through the official NHS app or website |
| [1071](https://www.english-corpora.org/corona/x4.asp?rs=&t=53041149&ID=21330128643) | [21-08-14 GB](https://www.english-corpora.org/corona/x4.asp?rs=&t=53041149&ID=21330128643) | [news.sky.com](https://www.english-corpora.org/corona/x3.asp?node=&p=11&w10=coronavirus&w11=vaccination&r=) |  |  |  | the government of failing to take advantage of the UK's " world-leading " **coronavirus** **vaccination** programme while revealing passenger numbers remained " very low ". # Gatwick said it |
| [1072](https://www.english-corpora.org/corona/x4.asp?rs=&t=87774882&ID=21365271223) | [21-08-17 GB](https://www.english-corpora.org/corona/x4.asp?rs=&t=87774882&ID=21365271223) | [ITV](https://www.english-corpora.org/corona/x3.asp?node=&p=11&w10=coronavirus&w11=vaccination&r=) |  |  |  | to be approved for 16 and 17-year-olds # The **coronavirus** **vaccination** programme looks set to be rolled out to more than a million more teenagers with |
| [1073](https://www.english-corpora.org/corona/x4.asp?rs=&t=87781861&ID=21374886019) | [21-08-18 GB](https://www.english-corpora.org/corona/x4.asp?rs=&t=87781861&ID=21374886019) | [ITV](https://www.english-corpora.org/corona/x3.asp?node=&p=11&w10=coronavirus&w11=vaccination&r=) |  |  |  | healthy 16 and 17 year olds in the UK are now eligible for a **coronavirus** **vaccination** and will soon be offered to book an appointment, the government has confirmed. |
| [1074](https://www.english-corpora.org/corona/x4.asp?rs=&t=87792746&ID=21396891659) | [21-08-20 GB](https://www.english-corpora.org/corona/x4.asp?rs=&t=87792746&ID=21396891659) | [The Sun](https://www.english-corpora.org/corona/x3.asp?node=&p=11&w10=coronavirus&w11=vaccination&r=) |  |  |  | of family member # BRIGHTON boss Graham Potter has urged everyone to accept their **coronavirus** **vaccination** invitation after the tragic loss of a family member. # The Seagulls chief, |
| [1075](https://www.english-corpora.org/corona/x4.asp?rs=&t=87795870&ID=21404512204) | [21-08-21 GB](https://www.english-corpora.org/corona/x4.asp?rs=&t=87795870&ID=21404512204) | [Sky](https://www.english-corpora.org/corona/x3.asp?node=&p=11&w10=coronavirus&w11=vaccination&r=) |  |  |  | tribes in the United States will pay its members and employees to get a **coronavirus** **vaccination**. # The Oneida and Menominee tribes in northeastern Wisconsin are offering a $500 incentive |
| [1076](https://www.english-corpora.org/corona/x4.asp?rs=&t=63250746&ID=21381466047) | [21-08-19 IE](https://www.english-corpora.org/corona/x4.asp?rs=&t=63250746&ID=21381466047) | [tradearabia.com](https://www.english-corpora.org/corona/x3.asp?node=&p=11&w10=coronavirus&w11=vaccination&r=) |  |  |  | , Modi also listed his government's achievements since 2014 and hailed India's **coronavirus** **vaccination** campaign. # " We are proud that we didn't have to depend on |
| [1077](https://www.english-corpora.org/corona/x4.asp?rs=&t=73337133&ID=21297037817) | [21-08-11 AU](https://www.english-corpora.org/corona/x4.asp?rs=&t=73337133&ID=21297037817) | [examiner.com.au](https://www.english-corpora.org/corona/x3.asp?node=&p=11&w10=coronavirus&w11=vaccination&r=) |  |  |  | a six-week **coronavirus** **vaccination** blitz to boost the island state's defence against the more infectious Delta strain. |
| [1078](https://www.english-corpora.org/corona/x4.asp?rs=&t=73340016&ID=21307584345) | [21-08-12 AU](https://www.english-corpora.org/corona/x4.asp?rs=&t=73340016&ID=21307584345) | [au.news.yahoo.com](https://www.english-corpora.org/corona/x3.asp?node=&p=11&w10=coronavirus&w11=vaccination&r=) |  |  |  | a demonstration demanding political reform and calling for a change to the country's **coronavirus** **vaccination** programme in Bangkok in August 7, 2021 # Thai police fired tear gas and |
| [1079](https://www.english-corpora.org/corona/x4.asp?rs=&t=73340016&ID=21307584383) | [21-08-12 AU](https://www.english-corpora.org/corona/x4.asp?rs=&t=73340016&ID=21307584383) | [au.news.yahoo.com](https://www.english-corpora.org/corona/x3.asp?node=&p=11&w10=coronavirus&w11=vaccination&r=) |  |  |  | in Bangkok demanding political reform and calling for a change to the country's **coronavirus** **vaccination** programme. # Demonstrators defied restrictions on public gatherings introduced as Thailand battles its worst |
| [1080](https://www.english-corpora.org/corona/x4.asp?rs=&t=73340196&ID=21311854590) | [21-08-12 AU](https://www.english-corpora.org/corona/x4.asp?rs=&t=73340196&ID=21311854590) | [watoday.com.au](https://www.english-corpora.org/corona/x3.asp?node=&p=11&w10=coronavirus&w11=vaccination&r=) |  |  |  | judge has allowed Norwegian Cruise Line to demand that passengers show written proof of **coronavirus** **vaccination** before they board a ship, dealing a major blow to Florida Governor Ron DeSantis |
| [1081](https://www.english-corpora.org/corona/x4.asp?rs=&t=73366951&ID=21392717548) | [21-08-20 AU](https://www.english-corpora.org/corona/x4.asp?rs=&t=73366951&ID=21392717548) | [brisbanetimes.com.au](https://www.english-corpora.org/corona/x3.asp?node=&p=11&w10=coronavirus&w11=vaccination&r=) |  |  |  | the national reopening plan, telling them they made a deal with Australians on **coronavirus** **vaccination** targets and have to keep up their end of the bargain. # The deal |
| [1082](https://www.english-corpora.org/corona/x4.asp?rs=&t=73367294&ID=21394902823) | [21-08-20 AU](https://www.english-corpora.org/corona/x4.asp?rs=&t=73367294&ID=21394902823) | [examiner.com.au](https://www.english-corpora.org/corona/x3.asp?node=&p=11&w10=coronavirus&w11=vaccination&r=) |  |  |  | defence troops dispatched to boost **coronavirus** **vaccination** rates in western NSW Indigenous communities will fail without trusted Aboriginal elders working alongside them |
| [1083](https://www.english-corpora.org/corona/x4.asp?rs=&t=73375058&ID=21420392024) | [21-08-23 AU](https://www.english-corpora.org/corona/x4.asp?rs=&t=73375058&ID=21420392024) | [perthnow.com.au](https://www.english-corpora.org/corona/x3.asp?node=&p=11&w10=coronavirus&w11=vaccination&r=) |  |  |  | 's tourism industry has called for a " no jab, no fly " **coronavirus** **vaccination** policy for all interstate arrivals to be implemented from December. # Tourism Council WA |
| [1084](https://www.english-corpora.org/corona/x4.asp?rs=&t=33115908&ID=21282647661) | [21-08-10 NZ](https://www.english-corpora.org/corona/x4.asp?rs=&t=33115908&ID=21282647661) | [nz.news.yahoo.com](https://www.english-corpora.org/corona/x3.asp?node=&p=11&w10=coronavirus&w11=vaccination&r=) |  |  |  | previously fallen short against Melbourne and Geelong. # Tasmania is launching a six-week **coronavirus** **vaccination** blitz to boost the island state's defence against the more infectious Delta strain.Premier Peter |
| [1085](https://www.english-corpora.org/corona/x4.asp?rs=&t=33156700&ID=21405568963) | [21-08-22 NZ](https://www.english-corpora.org/corona/x4.asp?rs=&t=33156700&ID=21405568963) | [nz.news.yahoo.com](https://www.english-corpora.org/corona/x3.asp?node=&p=11&w10=coronavirus&w11=vaccination&r=) |  |  |  | 's tourism industry has called for a " no jab, no fly " **coronavirus** **vaccination** policy for all interstate arrivals to be implemented from December. |
| [1086](https://www.english-corpora.org/corona/x4.asp?rs=&t=33160589&ID=21420722646) | [21-08-23 NZ](https://www.english-corpora.org/corona/x4.asp?rs=&t=33160589&ID=21420722646) | [radionz.co.nz](https://www.english-corpora.org/corona/x3.asp?node=&p=11&w10=coronavirus&w11=vaccination&r=) |  |  |  | hours to 8pm yesterday. # People wait in a queue for their Covid-19 **coronavirus** **vaccination** in Sydney. Photo: AFP # Three Covid-19 patients in their 80s, who |
| [1087](https://www.english-corpora.org/corona/x4.asp?rs=&t=43315371&ID=21300115602) | [21-08-11 IN](https://www.english-corpora.org/corona/x4.asp?rs=&t=43315371&ID=21300115602) | [odishatv.in](https://www.english-corpora.org/corona/x3.asp?node=&p=11&w10=coronavirus&w11=vaccination&r=) |  |  |  | # New Delhi: In a bid to speed up the pace of the **coronavirus** **vaccination** drive, Union Health Minister Harsh Vardhan on Wednesday announced that citizens will now be |
| [1088](https://www.english-corpora.org/corona/x4.asp?rs=&t=33156562&ID=21408193605) | [21-08-22 LK](https://www.english-corpora.org/corona/x4.asp?rs=&t=33156562&ID=21408193605) | [economynext.com](https://www.english-corpora.org/corona/x3.asp?node=&p=11&w10=coronavirus&w11=vaccination&r=) |  |  |  | 50 percent of Sri Lanka's apparel workforce had got a second **Coronavirus** **vaccination** dose and 11 care centres with 4,500 beds have been built for workers and more |
| [1089](https://www.english-corpora.org/corona/x4.asp?rs=&t=53040046&ID=21330003830) | [21-08-14 PK](https://www.english-corpora.org/corona/x4.asp?rs=&t=53040046&ID=21330003830) | [nation.com.pk](https://www.english-corpora.org/corona/x3.asp?node=&p=11&w10=coronavirus&w11=vaccination&r=) |  |  |  | The prime minister would also launch a mobile application COVID-19 Pass for verification of **coronavirus** **vaccination** certificate to allow immediate verification of the document by organizations or Pakistanis traveling within Pakistan |
| [1090](https://www.english-corpora.org/corona/x4.asp?rs=&t=53042619&ID=21328664990) | [21-08-14 PK](https://www.english-corpora.org/corona/x4.asp?rs=&t=53042619&ID=21328664990) | [brecorder.com](https://www.english-corpora.org/corona/x3.asp?node=&p=11&w10=coronavirus&w11=vaccination&r=) |  |  |  | Wednesday announced no citizen will be allowed air travel after September 10 without complete **coronavirus** **vaccination** certificates. # The decision in this regard was taken in a meeting of the |
| [1091](https://www.english-corpora.org/corona/x4.asp?rs=&t=53042619&ID=21328665041) | [21-08-14 PK](https://www.english-corpora.org/corona/x4.asp?rs=&t=53042619&ID=21328665041) | [brecorder.com](https://www.english-corpora.org/corona/x3.asp?node=&p=11&w10=coronavirus&w11=vaccination&r=) |  |  |  | discuss and review ongoing Covid-19 situation in the country. # The meeting declared **coronavirus** **vaccination** certificate mandatory for air travel after September 10. # The forum announced that the |
| [1092](https://www.english-corpora.org/corona/x4.asp?rs=&t=53060045&ID=21391909008) | [21-08-20 PK](https://www.english-corpora.org/corona/x4.asp?rs=&t=53060045&ID=21391909008) | [tribune.com.pk](https://www.english-corpora.org/corona/x3.asp?node=&p=11&w10=coronavirus&w11=vaccination&r=) |  |  |  | are in critical condition. She told meeting participants that 292,195 citizens were administered **coronavirus** **vaccination** on Monday. # Punjab chief secretary instructed Lahore, Faisalabad, and Sargodha divisions |
| [1093](https://www.english-corpora.org/corona/x4.asp?rs=&t=53025640&ID=21286654317) | [21-08-10 BD](https://www.english-corpora.org/corona/x4.asp?rs=&t=53025640&ID=21286654317) | [bdnews24.com](https://www.english-corpora.org/corona/x3.asp?node=&p=11&w10=coronavirus&w11=vaccination&r=) |  |  |  | . Photo: Asif Mahmud Ove # People line up in long queues for **coronavirus** **vaccination** at Dhaka's Sarkari Karmachari Hospital on Tuesday, Aug 10, 2021. Photo |
| [1094](https://www.english-corpora.org/corona/x4.asp?rs=&t=53043434&ID=21346672982) | [21-08-16 BD](https://www.english-corpora.org/corona/x4.asp?rs=&t=53043434&ID=21346672982) | [bdnews24.com](https://www.english-corpora.org/corona/x3.asp?node=&p=11&w10=coronavirus&w11=vaccination&r=) |  |  |  | , 2021, the first day of the second round of Bangladesh's nationwide **coronavirus** **vaccination** campaign. Photo: Mahmud Zaman Ovi # " Incepta is prepared, but there |
| [1095](https://www.english-corpora.org/corona/x4.asp?rs=&t=53043434&ID=21346673199) | [21-08-16 BD](https://www.english-corpora.org/corona/x4.asp?rs=&t=53043434&ID=21346673199) | [bdnews24.com](https://www.english-corpora.org/corona/x3.asp?node=&p=11&w10=coronavirus&w11=vaccination&r=) |  |  |  | 19, 2021, the first day of the second phase of Bangladesh's **coronavirus** **vaccination** campaign. Photo: Mahmud Zaman Ovi # A cabinet committee on government procurement had |
| [1096](https://www.english-corpora.org/corona/x4.asp?rs=&t=43335293&ID=21360173802) | [21-08-17 MY](https://www.english-corpora.org/corona/x4.asp?rs=&t=43335293&ID=21360173802) | [theborneopost.com](https://www.english-corpora.org/corona/x3.asp?node=&p=11&w10=coronavirus&w11=vaccination&r=) |  |  |  | , said he was made to understand that some of those queuing outside the **coronavirus** **vaccination** site had to come early in order to catch boat rides back to islands near |
| [1097](https://www.english-corpora.org/corona/x4.asp?rs=&t=53037152&ID=21320256835) | [21-08-13 PH](https://www.english-corpora.org/corona/x4.asp?rs=&t=53037152&ID=21320256835) | [newsinfo.inquirer.net](https://www.english-corpora.org/corona/x3.asp?node=&p=11&w10=coronavirus&w11=vaccination&r=) |  |  |  | demonstration demanding political reform and calling for a change to the country's Covid-19 **coronavirus** **vaccination** program, in the southern province of Narathiwat on August 7, 2021. AFP |
| [1098](https://www.english-corpora.org/corona/x4.asp?rs=&t=53037152&ID=21320256882) | [21-08-13 PH](https://www.english-corpora.org/corona/x4.asp?rs=&t=53037152&ID=21320256882) | [newsinfo.inquirer.net](https://www.english-corpora.org/corona/x3.asp?node=&p=11&w10=coronavirus&w11=vaccination&r=) |  |  |  | in Bangkok demanding political reform and calling for a change to the country's **coronavirus** **vaccination** program. # The Thai government has come under fire for the sluggish rollout of |
| [1099](https://www.english-corpora.org/corona/x4.asp?rs=&t=73353944&ID=21348197035) | [21-08-16 PH](https://www.english-corpora.org/corona/x4.asp?rs=&t=73353944&ID=21348197035) | [businessmirror.com.ph](https://www.english-corpora.org/corona/x3.asp?node=&p=11&w10=coronavirus&w11=vaccination&r=) |  |  |  | , Philippines on Friday, August 6, 2021. Thousands of people jammed **coronavirus** **vaccination** centers in the Philippine capital, defying social distancing restrictions, after false news spread |
| [1100](https://www.english-corpora.org/corona/x4.asp?rs=&t=63229364&ID=21309347881) | [21-08-12 KE](https://www.english-corpora.org/corona/x4.asp?rs=&t=63229364&ID=21309347881) | [citizentv.co.ke](https://www.english-corpora.org/corona/x3.asp?node=&p=11&w10=coronavirus&w11=vaccination&r=) |  |  |  | More Ugandans turn up for COVID-19 vaccinations # FILE - A woman receives a **coronavirus** **vaccination** at the Kololo airstrip in Kampala, Uganda, May 31, 2021. |

PAGE:   [**<<**](https://www.english-corpora.org/corona/x3.asp?node=&p=1&w10=coronavirus&w11=vaccination&r=)  [**<**](https://www.english-corpora.org/corona/x3.asp?node=&p=10&w10=coronavirus&w11=vaccination&r=)   11 / 42  [**>**](https://www.english-corpora.org/corona/x3.asp?node=&p=12&w10=coronavirus&w11=vaccination&r=)   [**>>**](https://www.english-corpora.org/corona/x3.asp?node=&p=42&w10=coronavirus&w11=vaccination&r=)

Конец формы

| PAGE:   [**<<**](https://www.english-corpora.org/corona/x3.asp?node=&p=1&w10=coronavirus&w11=vaccination&r=)  [**<**](https://www.english-corpora.org/corona/x3.asp?node=&p=11&w10=coronavirus&w11=vaccination&r=)   12 / 42  [**>**](https://www.english-corpora.org/corona/x3.asp?node=&p=13&w10=coronavirus&w11=vaccination&r=)   [**>>**](https://www.english-corpora.org/corona/x3.asp?node=&p=42&w10=coronavirus&w11=vaccination&r=) |  |
| --- | --- |

Начало формы

| CLICK FOR MORE CONTEXT | [**HELP**](javascript:newFeatures()) | [SAVE](javascript:chooser('s'))    [TRANSLATE](javascript:chooser('t'))    [ANALYZE](javascript:chooser('p')) |
| --- | --- | --- |

| [1101](https://www.english-corpora.org/corona/x4.asp?rs=&t=33085380&ID=21184049818) | [21-07-31 US](https://www.english-corpora.org/corona/x4.asp?rs=&t=33085380&ID=21184049818) | [kvoa.com](https://www.english-corpora.org/corona/x3.asp?node=&p=12&w10=coronavirus&w11=vaccination&r=) |  |  |  | AP) -- Three New Orleans music venues are going to require proof of **coronavirus** **vaccination** or a negative test to listen to music inside. The clubs are Tipitina's |
| --- | --- | --- | --- | --- | --- | --- |
| [1102](https://www.english-corpora.org/corona/x4.asp?rs=&t=33085803&ID=21180656948) | [21-07-31 US](https://www.english-corpora.org/corona/x4.asp?rs=&t=33085803&ID=21180656948) | [abqjournal.com](https://www.english-corpora.org/corona/x3.asp?node=&p=12&w10=coronavirus&w11=vaccination&r=) |  |  |  | : Thursday, June 17th, 2021 at 11:11am # A woman receives a **coronavirus** **vaccination** at the Kololo airstrip in Kampala, Uganda, Monday, May 31, 2021 |
| [1103](https://www.english-corpora.org/corona/x4.asp?rs=&t=73295376&ID=21185349284) | [21-07-31 US](https://www.english-corpora.org/corona/x4.asp?rs=&t=73295376&ID=21185349284) | [pe.com](https://www.english-corpora.org/corona/x3.asp?node=&p=12&w10=coronavirus&w11=vaccination&r=) |  |  |  | the Riverside-Corona Resource Conservation District. 73295378 # **Coronavirus** **vaccination** clinics in Riverside and Lake Elsinore will close amid an ongoing shift to mobile vaccine |
| [1104](https://www.english-corpora.org/corona/x4.asp?rs=&t=87676948&ID=21178870262) | [21-07-30 US](https://www.english-corpora.org/corona/x4.asp?rs=&t=87676948&ID=21178870262) | [The Jerusalem Post Blogs](https://www.english-corpora.org/corona/x3.asp?node=&p=12&w10=coronavirus&w11=vaccination&r=) |  |  |  | at Jerusalem's Misgav Ladach Hospital on June 6, as Israel began its **coronavirus** **vaccination** campaign for 12-to-15 year olds # (photo credit: MARC ISRAEL SELLEM) # |
| [1105](https://www.english-corpora.org/corona/x4.asp?rs=&t=87685164&ID=21187267532) | [21-07-31 US](https://www.english-corpora.org/corona/x4.asp?rs=&t=87685164&ID=21187267532) | [The Independent on MSN.com](https://www.english-corpora.org/corona/x3.asp?node=&p=12&w10=coronavirus&w11=vaccination&r=) |  |  |  | has ditched its plans for university students to be required to show proof of **coronavirus** **vaccination** in order to enter halls of residence and attend lectures -- just five days after |
| [1106](https://www.english-corpora.org/corona/x4.asp?rs=&t=33093045&ID=21210770193) | [21-08-03 US](https://www.english-corpora.org/corona/x4.asp?rs=&t=33093045&ID=21210770193) | [politico.com](https://www.english-corpora.org/corona/x3.asp?node=&p=12&w10=coronavirus&w11=vaccination&r=) |  |  |  | . Driving the Day # RENEWED VACCINATION RATES OVERSHADOWED BY DELTA CONCERNS -- **Coronavirus** **vaccination** rates are springing back amid case surges and Delta variant fears. In the last |
| [1107](https://www.english-corpora.org/corona/x4.asp?rs=&t=33098706&ID=21220312489) | [21-08-04 US](https://www.english-corpora.org/corona/x4.asp?rs=&t=33098706&ID=21220312489) | [aljazeera.com](https://www.english-corpora.org/corona/x3.asp?node=&p=12&w10=coronavirus&w11=vaccination&r=) |  |  |  | number of job openings as consumers unleash pent-up demand in the wake of increasing **coronavirus** **vaccination** rates. # But as firms gear up operations en masse, bottlenecks are forming |
| [1108](https://www.english-corpora.org/corona/x4.asp?rs=&t=33104433&ID=21242108948) | [21-08-06 US](https://www.english-corpora.org/corona/x4.asp?rs=&t=33104433&ID=21242108948) | [deadline.com](https://www.english-corpora.org/corona/x3.asp?node=&p=12&w10=coronavirus&w11=vaccination&r=) |  |  |  | Vaccinating America ", Roker pulled up his sleeve Tuesday and received the Pfizer **coronavirus** **vaccination**, administered by NYC's Lenox Hill Hospital nurse Jessica Callard. Before he... |
| [1109](https://www.english-corpora.org/corona/x4.asp?rs=&t=33107550&ID=21255310470) | [21-08-07 US](https://www.english-corpora.org/corona/x4.asp?rs=&t=33107550&ID=21255310470) | [dailyherald.com](https://www.english-corpora.org/corona/x3.asp?node=&p=12&w10=coronavirus&w11=vaccination&r=) |  |  |  | , Philippines on Friday, August 6, 2021. Thousands of people jammed **coronavirus** **vaccination** centers in the Philippine capital, defying social distancing restrictions, after false news spread |
| [1120](https://www.english-corpora.org/corona/x4.asp?rs=&t=33107678&ID=21257097702) | [21-08-07 US](https://www.english-corpora.org/corona/x4.asp?rs=&t=33107678&ID=21257097702) | [newspressnow.com](https://www.english-corpora.org/corona/x3.asp?node=&p=12&w10=coronavirus&w11=vaccination&r=) [(10)](https://www.english-corpora.org/corona/duplicates1.asp?xx=1119&n=10) |  |  |  | Monday, Aug. 2, 2021. Louisiana has one of the lowest **coronavirus** **vaccination** rates in the nation and is seeing one of the country's worst COVID-19 spikes |
| [1126](https://www.english-corpora.org/corona/x4.asp?rs=&t=33110147&ID=21263792612) | [21-08-08 US](https://www.english-corpora.org/corona/x4.asp?rs=&t=33110147&ID=21263792612) | [twincities.com](https://www.english-corpora.org/corona/x3.asp?node=&p=12&w10=coronavirus&w11=vaccination&r=) [(5)](https://www.english-corpora.org/corona/duplicates1.asp?xx=1125&n=5) |  |  |  | of vaccine mandates at a moment when high-profile companies have informed employees that **coronavirus** **vaccination** requirements are in the works, and some localities have adopted or are contemplating vaccine |
| [1127](https://www.english-corpora.org/corona/x4.asp?rs=&t=43281768&ID=21202539147) | [21-08-02 US](https://www.english-corpora.org/corona/x4.asp?rs=&t=43281768&ID=21202539147) | [wsbtv.com](https://www.english-corpora.org/corona/x3.asp?node=&p=12&w10=coronavirus&w11=vaccination&r=) |  |  |  | Girtz said. # Girtz said there will be a renewed push to increase **coronavirus** **vaccination** rates in Athens. # The Georgia Department of Public Health says the two-week total |
| [1128](https://www.english-corpora.org/corona/x4.asp?rs=&t=53007785&ID=21226157458) | [21-08-04 US](https://www.english-corpora.org/corona/x4.asp?rs=&t=53007785&ID=21226157458) | [wyff4.com](https://www.english-corpora.org/corona/x3.asp?node=&p=12&w10=coronavirus&w11=vaccination&r=) |  |  |  | enacted legislation this year that would restrict public schools and universities from requiring either **coronavirus** **vaccination** or documentation of vaccination status.Once vaccinations were required for measles, " transmission in schools |
| [1129](https://www.english-corpora.org/corona/x4.asp?rs=&t=53007785&ID=21226160110) | [21-08-04 US](https://www.english-corpora.org/corona/x4.asp?rs=&t=53007785&ID=21226160110) | [wyff4.com](https://www.english-corpora.org/corona/x3.asp?node=&p=12&w10=coronavirus&w11=vaccination&r=) |  |  |  | enacted legislation this year that would restrict public schools and universities from requiring either **coronavirus** **vaccination** or documentation of vaccination status. # Once vaccinations were required for measles, " |
| [1130](https://www.english-corpora.org/corona/x4.asp?rs=&t=87692670&ID=21206695701) | [21-08-02 US](https://www.english-corpora.org/corona/x4.asp?rs=&t=87692670&ID=21206695701) | [YAHOO!News](https://www.english-corpora.org/corona/x3.asp?node=&p=12&w10=coronavirus&w11=vaccination&r=) |  |  |  | " he said. " Nobody wants that. " # In mandating the **coronavirus** **vaccination** for federal workers, Biden said he would also ask Defense Department leaders to explore |
| [1131](https://www.english-corpora.org/corona/x4.asp?rs=&t=87694704&ID=21204786116) | [21-08-02 US](https://www.english-corpora.org/corona/x4.asp?rs=&t=87694704&ID=21204786116) | [Chicago Tribune](https://www.english-corpora.org/corona/x3.asp?node=&p=12&w10=coronavirus&w11=vaccination&r=) |  |  |  | the southernmost city in Illinois and the seat of the county with the lowest **coronavirus** **vaccination** rate in the state. # Wissinger said the low uptake isn't due to |
| [1132](https://www.english-corpora.org/corona/x4.asp?rs=&t=87696748&ID=21218360832) | [21-08-03 US](https://www.english-corpora.org/corona/x4.asp?rs=&t=87696748&ID=21218360832) | [Associated Press](https://www.english-corpora.org/corona/x3.asp?node=&p=12&w10=coronavirus&w11=vaccination&r=) |  |  |  | , S.C. Age-eligible South Carolina children would be required to show proof of **coronavirus** **vaccination** in order to attend public schools in the state, under a proposal introduced by |
| [1133](https://www.english-corpora.org/corona/x4.asp?rs=&t=87696748&ID=21218360882) | [21-08-03 US](https://www.english-corpora.org/corona/x4.asp?rs=&t=87696748&ID=21218360882) | [Associated Press](https://www.english-corpora.org/corona/x3.asp?node=&p=12&w10=coronavirus&w11=vaccination&r=) |  |  |  | part of the Democratic state senator's plan to boost South Carolina's lagging **coronavirus** **vaccination** rate, if she's elected to its top office. (AP Photo/Meg Kinnard |
| [1134](https://www.english-corpora.org/corona/x4.asp?rs=&t=87696748&ID=21218360956) | [21-08-03 US](https://www.english-corpora.org/corona/x4.asp?rs=&t=87696748&ID=21218360956) | [Associated Press](https://www.english-corpora.org/corona/x3.asp?node=&p=12&w10=coronavirus&w11=vaccination&r=) |  |  |  | , S.C. Age-eligible South Carolina children would be required to show proof of **coronavirus** **vaccination** in order to attend public schools in the state, under a proposal introduced by |
| [1135](https://www.english-corpora.org/corona/x4.asp?rs=&t=87696748&ID=21218361006) | [21-08-03 US](https://www.english-corpora.org/corona/x4.asp?rs=&t=87696748&ID=21218361006) | [Associated Press](https://www.english-corpora.org/corona/x3.asp?node=&p=12&w10=coronavirus&w11=vaccination&r=) |  |  |  | part of the Democratic state senator's plan to boost South Carolina's lagging **coronavirus** **vaccination** rate, if she's elected to its top office. (AP Photo/Meg Kinnard |
| [1136](https://www.english-corpora.org/corona/x4.asp?rs=&t=87696748&ID=21218361046) | [21-08-03 US](https://www.english-corpora.org/corona/x4.asp?rs=&t=87696748&ID=21218361046) | [Associated Press](https://www.english-corpora.org/corona/x3.asp?node=&p=12&w10=coronavirus&w11=vaccination&r=) |  |  |  | AP) -- Age-eligible South Carolina children would be required to show proof of **coronavirus** **vaccination** in order to attend public schools in the state, under a proposal introduced by |
| [1137](https://www.english-corpora.org/corona/x4.asp?rs=&t=87696748&ID=21218361090) | [21-08-03 US](https://www.english-corpora.org/corona/x4.asp?rs=&t=87696748&ID=21218361090) | [Associated Press](https://www.english-corpora.org/corona/x3.asp?node=&p=12&w10=coronavirus&w11=vaccination&r=) |  |  |  | as part of the state senator's plan to boost South Carolina's lagging **coronavirus** **vaccination** rate, if she's elected to its top office in 2022. # Also |
| [1138](https://www.english-corpora.org/corona/x4.asp?rs=&t=87697512&ID=21216072056) | [21-08-03 US](https://www.english-corpora.org/corona/x4.asp?rs=&t=87697512&ID=21216072056) | [Detroit Free Press on MSN.com](https://www.english-corpora.org/corona/x3.asp?node=&p=12&w10=coronavirus&w11=vaccination&r=) |  |  |  | Bill de Blasio said Tuesday that people will have to start showing proof of **coronavirus** **vaccination** to do many indoor events, including dining, or be denied entry in an |
| [1139](https://www.english-corpora.org/corona/x4.asp?rs=&t=87699310&ID=21215812520) | [21-08-03 US](https://www.english-corpora.org/corona/x4.asp?rs=&t=87699310&ID=21215812520) | [The Independent on MSN.com](https://www.english-corpora.org/corona/x3.asp?node=&p=12&w10=coronavirus&w11=vaccination&r=) |  |  |  | , Eleanor Sly # New York City is expected to announce that proof of **coronavirus** **vaccination** will be required to allow people to access indoor activities, including restaurants and gyms |
| [1140](https://www.english-corpora.org/corona/x4.asp?rs=&t=87704082&ID=21230796198) | [21-08-04 US](https://www.english-corpora.org/corona/x4.asp?rs=&t=87704082&ID=21230796198) | [The Hill](https://www.english-corpora.org/corona/x3.asp?node=&p=12&w10=coronavirus&w11=vaccination&r=) |  |  |  | he won't be joining band on tour over lack of **coronavirus** **vaccination** # The drummer for The Offspring says he was dropped from the band for refusing |
| [1141](https://www.english-corpora.org/corona/x4.asp?rs=&t=87704506&ID=21228407635) | [21-08-04 US](https://www.english-corpora.org/corona/x4.asp?rs=&t=87704506&ID=21228407635) | [The Hill](https://www.english-corpora.org/corona/x3.asp?node=&p=12&w10=coronavirus&w11=vaccination&r=) |  |  |  | all personnel to wear a mask in enclosed areas at all times regardless of **coronavirus** **vaccination** status beginning with this weekend's race at Watkins Glen International in New York. |
| [1142](https://www.english-corpora.org/corona/x4.asp?rs=&t=87708787&ID=21240969504) | [21-08-05 US](https://www.english-corpora.org/corona/x4.asp?rs=&t=87708787&ID=21240969504) | [YAHOO!News](https://www.english-corpora.org/corona/x3.asp?node=&p=12&w10=coronavirus&w11=vaccination&r=) |  |  |  | the day's political news. # Tens of thousands of Pakistanis are thronging **coronavirus** **vaccination** centres daily after officials announced penalties for the unvaccinated, including blocked cell phones and |
| [1143](https://www.english-corpora.org/corona/x4.asp?rs=&t=87708914&ID=21237637922) | [21-08-05 US](https://www.english-corpora.org/corona/x4.asp?rs=&t=87708914&ID=21237637922) | [Associated Press](https://www.english-corpora.org/corona/x3.asp?node=&p=12&w10=coronavirus&w11=vaccination&r=) |  |  |  | (AP) -- Thousands of people jammed **coronavirus** **vaccination** centers in the Philippine capital, defying social distancing restrictions, after false news spread |
| [1146](https://www.english-corpora.org/corona/x4.asp?rs=&t=87710143&ID=21238857432) | [21-08-05 US](https://www.english-corpora.org/corona/x4.asp?rs=&t=87710143&ID=21238857432) | [ABCNews](https://www.english-corpora.org/corona/x3.asp?node=&p=12&w10=coronavirus&w11=vaccination&r=) [(2)](https://www.english-corpora.org/corona/duplicates1.asp?xx=1145&n=2) |  |  |  | and water cannons to disperse protesters who gathered Wednesday in Athens to oppose **coronavirus** **vaccination** requirements proposed by the Greek government. # The demonstration in front of the parliament |
| [1147](https://www.english-corpora.org/corona/x4.asp?rs=&t=87711876&ID=21241130408) | [21-08-05 US](https://www.english-corpora.org/corona/x4.asp?rs=&t=87711876&ID=21241130408) | [Washington Post](https://www.english-corpora.org/corona/x3.asp?node=&p=12&w10=coronavirus&w11=vaccination&r=) |  |  |  | Clapton announced Wednesday that he won't perform at venues that require proof of **coronavirus** **vaccination** for people to attend, becoming one of the first major artists to rebuke a |
| [1148](https://www.english-corpora.org/corona/x4.asp?rs=&t=87711963&ID=21238925927) | [21-08-05 US](https://www.english-corpora.org/corona/x4.asp?rs=&t=87711963&ID=21238925927) | [New York Times](https://www.english-corpora.org/corona/x3.asp?node=&p=12&w10=coronavirus&w11=vaccination&r=) |  |  |  | , worried that **coronavirus** **vaccination** rates among young people are lagging as the new school year approaches, unveiled on |
| [1149](https://www.english-corpora.org/corona/x4.asp?rs=&t=87712211&ID=21237101044) | [21-08-05 US](https://www.english-corpora.org/corona/x4.asp?rs=&t=87712211&ID=21237101044) | [Yahoo](https://www.english-corpora.org/corona/x3.asp?node=&p=12&w10=coronavirus&w11=vaccination&r=) |  |  |  | included wooden buildings more than a century old. # Thousands of people jammed **coronavirus** **vaccination** centers in the Philippine capital, defying social distancing restrictions, after false news spread |
| [1152](https://www.english-corpora.org/corona/x4.asp?rs=&t=87713938&ID=21240000341) | [21-08-05 US](https://www.english-corpora.org/corona/x4.asp?rs=&t=87713938&ID=21240000341) | [Washington Post](https://www.english-corpora.org/corona/x3.asp?node=&p=12&w10=coronavirus&w11=vaccination&r=) [(2)](https://www.english-corpora.org/corona/duplicates1.asp?xx=1151&n=2) |  |  |  | coverage -- were better able to tap into primary care networks during their **coronavirus** **vaccination** rollouts, Schneider said. # Story continues below advertisement # He said the findings |
| [1153](https://www.english-corpora.org/corona/x4.asp?rs=&t=87714003&ID=21249104058) | [21-08-06 US](https://www.english-corpora.org/corona/x4.asp?rs=&t=87714003&ID=21249104058) | [YAHOO!News](https://www.english-corpora.org/corona/x3.asp?node=&p=12&w10=coronavirus&w11=vaccination&r=) |  |  |  | a Florida law prohibiting cruise companies from demanding that passengers show written proof of **coronavirus** **vaccination** before they board a ship. # Florida's Board of Education on Friday unanimously |
| [1155](https://www.english-corpora.org/corona/x4.asp?rs=&t=87718204&ID=21249668256) | [21-08-06 US](https://www.english-corpora.org/corona/x4.asp?rs=&t=87718204&ID=21249668256) | [Yahoo](https://www.english-corpora.org/corona/x3.asp?node=&p=12&w10=coronavirus&w11=vaccination&r=) [(1)](https://www.english-corpora.org/corona/duplicates1.asp?xx=1154&n=1) |  |  |  | will flow through the pipeline each day. # Thousands of people jammed **coronavirus** **vaccination** centers in the Philippine capital, defying social distancing restrictions, after false news spread |
| [1156](https://www.english-corpora.org/corona/x4.asp?rs=&t=87719103&ID=21249431412) | [21-08-06 US](https://www.english-corpora.org/corona/x4.asp?rs=&t=87719103&ID=21249431412) | [SFGate](https://www.english-corpora.org/corona/x3.asp?node=&p=12&w10=coronavirus&w11=vaccination&r=) |  |  |  | has a long way to go, with one of the nation's lowest **coronavirus** **vaccination** rates. Only 37% of Louisiana's population is fully vaccinated against COVID-19, according |
| [1157](https://www.english-corpora.org/corona/x4.asp?rs=&t=87720703&ID=21258773711) | [21-08-07 US](https://www.english-corpora.org/corona/x4.asp?rs=&t=87720703&ID=21258773711) | [Yahoo! Sports](https://www.english-corpora.org/corona/x3.asp?node=&p=12&w10=coronavirus&w11=vaccination&r=) |  |  |  | a Florida law prohibiting cruise companies from demanding that passengers show written proof of **coronavirus** **vaccination** before they board a ship. # ? More than 11 million Californians who get |
| [1160](https://www.english-corpora.org/corona/x4.asp?rs=&t=87725174&ID=21269776634) | [21-08-08 US](https://www.english-corpora.org/corona/x4.asp?rs=&t=87725174&ID=21269776634) | [AOL](https://www.english-corpora.org/corona/x3.asp?node=&p=12&w10=coronavirus&w11=vaccination&r=) [(2)](https://www.english-corpora.org/corona/duplicates1.asp?xx=1159&n=2) |  |  |  | of the over-70s arrive at the EICC to receive their first dose of **coronavirus** **vaccination** on Feb. 1, 2021, in Edinburgh, Scotland. # Jamie Burnett, |
| [1162](https://www.english-corpora.org/corona/x4.asp?rs=&t=87725174&ID=21269777746) | [21-08-08 US](https://www.english-corpora.org/corona/x4.asp?rs=&t=87725174&ID=21269777746) | [AOL](https://www.english-corpora.org/corona/x3.asp?node=&p=12&w10=coronavirus&w11=vaccination&r=) [(1)](https://www.english-corpora.org/corona/duplicates1.asp?xx=1161&n=1) |  |  |  | 2021, in Anaheim, Calif. California's Disneyland opened a giant **coronavirus** **vaccination** center on January 13, some 10 months after the pandemic's appearance forced the |
| [1163](https://www.english-corpora.org/corona/x4.asp?rs=&t=87725532&ID=21267442542) | [21-08-08 US](https://www.english-corpora.org/corona/x4.asp?rs=&t=87725532&ID=21267442542) | [ABC](https://www.english-corpora.org/corona/x3.asp?node=&p=12&w10=coronavirus&w11=vaccination&r=) |  |  |  | club has become the UK's first nightclub to be turned into a pop-up **Coronavirus** **vaccination** centre, as authorities try to attract younger people to get vaccinated. (AP |
| [1164](https://www.english-corpora.org/corona/x4.asp?rs=&t=87725852&ID=21268494735) | [21-08-08 US](https://www.english-corpora.org/corona/x4.asp?rs=&t=87725852&ID=21268494735) | [Yahoo! Sports](https://www.english-corpora.org/corona/x3.asp?node=&p=12&w10=coronavirus&w11=vaccination&r=) |  |  |  | allowed Norwegian Cruise Line Holdings Ltd. to demand that passengers show written proof of **coronavirus** **vaccination** before they board a ship, dealing a major blow to Florida Governor Ron DeSantis |
| [1165](https://www.english-corpora.org/corona/x4.asp?rs=&t=87726105&ID=21267708315) | [21-08-08 US](https://www.english-corpora.org/corona/x4.asp?rs=&t=87726105&ID=21267708315) | [ABC](https://www.english-corpora.org/corona/x3.asp?node=&p=12&w10=coronavirus&w11=vaccination&r=) |  |  |  | temporarily block a Florida law banning cruise companies from asking passengers for proof of **coronavirus** **vaccination** before they board a ship # ByThe Associated Press # August 9, 2021, |
| [1168](https://www.english-corpora.org/corona/x4.asp?rs=&t=87727024&ID=21267752831) | [21-08-08 US](https://www.english-corpora.org/corona/x4.asp?rs=&t=87727024&ID=21267752831) | [SFGate](https://www.english-corpora.org/corona/x3.asp?node=&p=12&w10=coronavirus&w11=vaccination&r=) [(2)](https://www.english-corpora.org/corona/duplicates1.asp?xx=1167&n=2) |  |  |  | has become the UK's first nightclub to be turned into a pop-up **Coronavirus** **vaccination** centre, as authorities try to attract younger people to get vaccinated.Alberto Pezzali/APShow MoreShow Less2of15A |
| [1177](https://www.english-corpora.org/corona/x4.asp?rs=&t=87727060&ID=21268346162) | [21-08-08 US](https://www.english-corpora.org/corona/x4.asp?rs=&t=87727060&ID=21268346162) | [SFGate](https://www.english-corpora.org/corona/x3.asp?node=&p=12&w10=coronavirus&w11=vaccination&r=) [(8)](https://www.english-corpora.org/corona/duplicates1.asp?xx=1176&n=8) |  |  |  | continue to climb because of the delta variant and the state's low **coronavirus** **vaccination** rate. # On the political front, Attorney General Jeff Landry and some other |
| [1178](https://www.english-corpora.org/corona/x4.asp?rs=&t=87727412&ID=21267550857) | [21-08-08 US](https://www.english-corpora.org/corona/x4.asp?rs=&t=87727412&ID=21267550857) | [SFGate](https://www.english-corpora.org/corona/x3.asp?node=&p=12&w10=coronavirus&w11=vaccination&r=) |  |  |  | Decines-Charpieu, France. The European Union has caught up to the once-vaunted U.S. **coronavirus** **vaccination** effort despite a sluggish start, with some 60% of EU residents having received at |
| [1182](https://www.english-corpora.org/corona/x4.asp?rs=&t=53012400&ID=21242617570) | [21-08-06 CA](https://www.english-corpora.org/corona/x4.asp?rs=&t=53012400&ID=21242617570) | [ca.news.yahoo.com](https://www.english-corpora.org/corona/x3.asp?node=&p=12&w10=coronavirus&w11=vaccination&r=) [(3)](https://www.english-corpora.org/corona/duplicates1.asp?xx=1181&n=3) |  |  |  | or # MANILA, Philippines (AP) -- Thousands of people jammed **coronavirus** **vaccination** centers in the Philippine capital, defying social distancing restrictions, after false news spread |
| [1184](https://www.english-corpora.org/corona/x4.asp?rs=&t=53015738&ID=21245379931) | [21-08-06 CA](https://www.english-corpora.org/corona/x4.asp?rs=&t=53015738&ID=21245379931) | [chroniclejournal.com](https://www.english-corpora.org/corona/x3.asp?node=&p=12&w10=coronavirus&w11=vaccination&r=) [(1)](https://www.english-corpora.org/corona/duplicates1.asp?xx=1183&n=1) |  |  |  | Florida law prohibiting cruise companies from demanding that passengers show written proof of **coronavirus** **vaccination** before they board a ship. # Norwegian contends the " vaccine passport " ban |
| [1185](https://www.english-corpora.org/corona/x4.asp?rs=&t=43283315&ID=21208732102) | [21-08-03 GB](https://www.english-corpora.org/corona/x4.asp?rs=&t=43283315&ID=21208732102) | [dailyrecord.co.uk](https://www.english-corpora.org/corona/x3.asp?node=&p=12&w10=coronavirus&w11=vaccination&r=) |  |  |  | per cent of the adult population -- have received their first dose of a **coronavirus** **vaccination** and 396,144 of them -- 74.8 per cent of the adult population -- have received |
| [1186](https://www.english-corpora.org/corona/x4.asp?rs=&t=43293301&ID=21242183947) | [21-08-06 GB](https://www.english-corpora.org/corona/x4.asp?rs=&t=43293301&ID=21242183947) | [bristolpost.co.uk](https://www.english-corpora.org/corona/x3.asp?node=&p=12&w10=coronavirus&w11=vaccination&r=) |  |  |  | vaccination centre closes today - where you can still get a jab # The **coronavirus** **vaccination** centre at Ashton Gate stadium in Bristol (Image: John Myers) # THE |
| [1187](https://www.english-corpora.org/corona/x4.asp?rs=&t=43297489&ID=21257473665) | [21-08-07 GB](https://www.english-corpora.org/corona/x4.asp?rs=&t=43297489&ID=21257473665) | [politico.eu](https://www.english-corpora.org/corona/x3.asp?node=&p=12&w10=coronavirus&w11=vaccination&r=) |  |  |  | of Brits support extending the U.K.'s **coronavirus** **vaccination** campaign to children over the age of 12, according to a new poll. |
| [1188](https://www.english-corpora.org/corona/x4.asp?rs=&t=43302032&ID=21271202293) | [21-08-09 GB](https://www.english-corpora.org/corona/x4.asp?rs=&t=43302032&ID=21271202293) | [metro.co.uk](https://www.english-corpora.org/corona/x3.asp?node=&p=12&w10=coronavirus&w11=vaccination&r=) |  |  |  | stir in April this year when he said young people should not get the **coronavirus** **vaccination**. # While President Joe Biden has said he has no plans to introduce a |
| [1189](https://www.english-corpora.org/corona/x4.asp?rs=&t=63210163&ID=21235220890) | [21-08-05 GB](https://www.english-corpora.org/corona/x4.asp?rs=&t=63210163&ID=21235220890) | [somersetlive.co.uk](https://www.english-corpora.org/corona/x3.asp?node=&p=12&w10=coronavirus&w11=vaccination&r=) |  |  |  | new head of the NHS in England has urged young people to get a **coronavirus** **vaccination**, warning that more than a fifth of those admitted to hospital with Covid-19 are |
| [1190](https://www.english-corpora.org/corona/x4.asp?rs=&t=63201424&ID=21221542429) | [21-08-04 IE](https://www.english-corpora.org/corona/x4.asp?rs=&t=63201424&ID=21221542429) | [intellinews.com](https://www.english-corpora.org/corona/x3.asp?node=&p=12&w10=coronavirus&w11=vaccination&r=) |  |  |  | 10.5% economic advance. According to the bank, despite the low rate of **coronavirus** **vaccination** at the moment in the country, a strong economic recovery is expected. The |
| [1191](https://www.english-corpora.org/corona/x4.asp?rs=&t=63201424&ID=21221542662) | [21-08-04 IE](https://www.english-corpora.org/corona/x4.asp?rs=&t=63201424&ID=21221542662) | [intellinews.com](https://www.english-corpora.org/corona/x3.asp?node=&p=12&w10=coronavirus&w11=vaccination&r=) |  |  |  | more uncertain than usual, the Fund acknowledged, urging Georgia to increase its **coronavirus** **vaccination** rate. # Georgia's consumer price inflation came in at 9.9% year on year |
| [1192](https://www.english-corpora.org/corona/x4.asp?rs=&t=63217515&ID=21272023544) | [21-08-09 IE](https://www.english-corpora.org/corona/x4.asp?rs=&t=63217515&ID=21272023544) | [echolive.ie](https://www.english-corpora.org/corona/x3.asp?node=&p=12&w10=coronavirus&w11=vaccination&r=) |  |  |  | Around 90% of people over the age of 16 have registered to receive a **coronavirus** **vaccination**. # On Thursday, children aged between 12 and 15 can register to receive |
| [1193](https://www.english-corpora.org/corona/x4.asp?rs=&t=73315487&ID=21248016126) | [21-08-06 AU](https://www.english-corpora.org/corona/x4.asp?rs=&t=73315487&ID=21248016126) | [news.com.au](https://www.english-corpora.org/corona/x3.asp?node=&p=12&w10=coronavirus&w11=vaccination&r=) |  |  |  | lost some friends who " refused " or " did not disclose " their **coronavirus** **vaccination** status. # The 52-year-old actress has been publicly supportive of social distancing, wearing |
| [1194](https://www.english-corpora.org/corona/x4.asp?rs=&t=73322609&ID=21263188117) | [21-08-08 AU](https://www.english-corpora.org/corona/x4.asp?rs=&t=73322609&ID=21263188117) | [broadsheet.com.au](https://www.english-corpora.org/corona/x3.asp?node=&p=12&w10=coronavirus&w11=vaccination&r=) |  |  |  | press conference this morning, Premier Daniel Andrews announced an expansion of Victoria's **coronavirus** **vaccination** program. From tomorrow, August 9, people aged 18 to 39 will be |
| [1195](https://www.english-corpora.org/corona/x4.asp?rs=&t=43288797&ID=21225790639) | [21-08-04 IN](https://www.english-corpora.org/corona/x4.asp?rs=&t=43288797&ID=21225790639) | [odishatv.in](https://www.english-corpora.org/corona/x3.asp?node=&p=12&w10=coronavirus&w11=vaccination&r=) |  |  |  | ... # Bhubaneswar: The Union Health Ministry said on Saturday that the much-awaited **coronavirus** **vaccination** drive will kick off on January 16. " It was decided that in view |
| [1196](https://www.english-corpora.org/corona/x4.asp?rs=&t=73324756&ID=21265064289) | [21-08-08 IN](https://www.english-corpora.org/corona/x4.asp?rs=&t=73324756&ID=21265064289) | [republicworld.com](https://www.english-corpora.org/corona/x3.asp?node=&p=12&w10=coronavirus&w11=vaccination&r=) |  |  |  | an amusing video surfaced from Malaysia featuring a Dinosaur taking a jab at the **Coronavirus** **vaccination** centre with an awareness message that has blown the netizens. The 2-minute creative clip |
| [1197](https://www.english-corpora.org/corona/x4.asp?rs=&t=53020452&ID=21264152368) | [21-08-08 PK](https://www.english-corpora.org/corona/x4.asp?rs=&t=53020452&ID=21264152368) | [nation.com.pk](https://www.english-corpora.org/corona/x3.asp?node=&p=12&w10=coronavirus&w11=vaccination&r=) |  |  |  | the lives of the people safe. The meeting agreed to further expedite the **coronavirus** **vaccination** process. # It was decided in the meeting that effective measures will be taken |
| [1198](https://www.english-corpora.org/corona/x4.asp?rs=&t=73299196&ID=21193457917) | [21-08-01 PH](https://www.english-corpora.org/corona/x4.asp?rs=&t=73299196&ID=21193457917) | [philstar.com](https://www.english-corpora.org/corona/x3.asp?node=&p=12&w10=coronavirus&w11=vaccination&r=) |  |  |  | , Philippines -- President Rodrigo Duterte ordered his government on Wednesday to open the **coronavirus** **vaccination** campaign to anyone who wants a shot as his country scrambles to protect the population |
| [1199](https://www.english-corpora.org/corona/x4.asp?rs=&t=73318884&ID=21248486541) | [21-08-06 PH](https://www.english-corpora.org/corona/x4.asp?rs=&t=73318884&ID=21248486541) | [bworldonline.com](https://www.english-corpora.org/corona/x3.asp?node=&p=12&w10=coronavirus&w11=vaccination&r=) |  |  |  | the government to consider its appeal to prioritize more than 215,000 prisoners in its **coronavirus** **vaccination** program # " We stress that no one should be left behind to ensure the |
| [1200](https://www.english-corpora.org/corona/x4.asp?rs=&t=63192919&ID=21189660652) | [21-08-01 KE](https://www.english-corpora.org/corona/x4.asp?rs=&t=63192919&ID=21189660652) | [businessdailyafrica.com](https://www.english-corpora.org/corona/x3.asp?node=&p=12&w10=coronavirus&w11=vaccination&r=) |  |  |  | coastal city of Tel Aviv on June 6, 2021, as Israel begins **coronavirus** **vaccination** campaign for 12 to 15-year-olds. Thank you for reading Nation.Africa # Israel will |

PAGE:   [**<<**](https://www.english-corpora.org/corona/x3.asp?node=&p=1&w10=coronavirus&w11=vaccination&r=)  [**<**](https://www.english-corpora.org/corona/x3.asp?node=&p=11&w10=coronavirus&w11=vaccination&r=)   12 / 42  [**>**](https://www.english-corpora.org/corona/x3.asp?node=&p=13&w10=coronavirus&w11=vaccination&r=)   [**>>**](https://www.english-corpora.org/corona/x3.asp?node=&p=42&w10=coronavirus&w11=vaccination&r=)

Конец формы

| PAGE:   [**<<**](https://www.english-corpora.org/corona/x3.asp?node=&p=1&w10=coronavirus&w11=vaccination&r=)  [**<**](https://www.english-corpora.org/corona/x3.asp?node=&p=12&w10=coronavirus&w11=vaccination&r=)   13 / 42  [**>**](https://www.english-corpora.org/corona/x3.asp?node=&p=14&w10=coronavirus&w11=vaccination&r=)   [**>>**](https://www.english-corpora.org/corona/x3.asp?node=&p=42&w10=coronavirus&w11=vaccination&r=) |  |
| --- | --- |

Начало формы

| CLICK FOR MORE CONTEXT | [**HELP**](javascript:newFeatures()) | [SAVE](javascript:chooser('s'))    [TRANSLATE](javascript:chooser('t'))    [ANALYZE](javascript:chooser('p')) |
| --- | --- | --- |

| [1201](https://www.english-corpora.org/corona/x4.asp?rs=&t=33035007&ID=21019594540) | [21-07-16 US](https://www.english-corpora.org/corona/x4.asp?rs=&t=33035007&ID=21019594540) | [nytimes.com](https://www.english-corpora.org/corona/x3.asp?node=&p=13&w10=coronavirus&w11=vaccination&r=) |  |  |  | The Daily': What the Japanese Think of the Olympics # Despite low **coronavirus** **vaccination** rates and an ambivalent public, Tokyo is still set to host the Games. |
| --- | --- | --- | --- | --- | --- | --- |
| [1202](https://www.english-corpora.org/corona/x4.asp?rs=&t=33035007&ID=21019594575) | [21-07-16 US](https://www.english-corpora.org/corona/x4.asp?rs=&t=33035007&ID=21019594575) | [nytimes.com](https://www.english-corpora.org/corona/x3.asp?node=&p=13&w10=coronavirus&w11=vaccination&r=) |  |  |  | The Daily': What the Japanese Think of the Olympics Despite low **coronavirus** **vaccination** rates and an ambivalent public, Tokyo is still set to host the Games. |
| [1203](https://www.english-corpora.org/corona/x4.asp?rs=&t=33036708&ID=21019995023) | [21-07-16 US](https://www.english-corpora.org/corona/x4.asp?rs=&t=33036708&ID=21019995023) | [aljazeera.com](https://www.english-corpora.org/corona/x3.asp?node=&p=13&w10=coronavirus&w11=vaccination&r=) |  |  |  | fall in jobless claims comes amid worker shortages in pockets of the economy as **coronavirus** **vaccination** rates continue to increase, consumers flush with savings unleash pent-up demand and businesses gear |
| [1204](https://www.english-corpora.org/corona/x4.asp?rs=&t=33039512&ID=21033104447) | [21-07-17 US](https://www.english-corpora.org/corona/x4.asp?rs=&t=33039512&ID=21033104447) | [nytimes.com](https://www.english-corpora.org/corona/x3.asp?node=&p=13&w10=coronavirus&w11=vaccination&r=) |  |  |  | millions of dollars in damages to businesses and the disruption of the nation's **coronavirus** **vaccination** program. # Protests that began last week in the eastern province of KwaZulu-Natal over |
| [1205](https://www.english-corpora.org/corona/x4.asp?rs=&t=33047028&ID=21063369169) | [21-07-20 US](https://www.english-corpora.org/corona/x4.asp?rs=&t=33047028&ID=21063369169) | [houstonchronicle.com](https://www.english-corpora.org/corona/x3.asp?node=&p=13&w10=coronavirus&w11=vaccination&r=) |  |  |  | age 50 and older. # Maine has one of the highest rates of **coronavirus** **vaccination** in the country. The vaccines are available to everyone age 12 and older in |
| [1206](https://www.english-corpora.org/corona/x4.asp?rs=&t=33047827&ID=21063478713) | [21-07-20 US](https://www.english-corpora.org/corona/x4.asp?rs=&t=33047827&ID=21063478713) | [fox4kc.com](https://www.english-corpora.org/corona/x3.asp?node=&p=13&w10=coronavirus&w11=vaccination&r=) |  |  |  | , expect more vaccination promotion # Middle school student Elise Robinson receives her first **coronavirus** **vaccination** on Wednesday, May 12, 2021, in Decatur, Ga. Hundreds of |
| [1207](https://www.english-corpora.org/corona/x4.asp?rs=&t=33051224&ID=21076121288) | [21-07-21 US](https://www.english-corpora.org/corona/x4.asp?rs=&t=33051224&ID=21076121288) | [aljazeera.com](https://www.english-corpora.org/corona/x3.asp?node=&p=13&w10=coronavirus&w11=vaccination&r=) |  |  |  | , Mexico's Foreign Minister Marcelo Ebrard said the country planned to bolster its **coronavirus** **vaccination** effort along the Mexican-US border in an effort to get the restrictions lifted. # |
| [1208](https://www.english-corpora.org/corona/x4.asp?rs=&t=33055551&ID=21090435925) | [21-07-22 US](https://www.english-corpora.org/corona/x4.asp?rs=&t=33055551&ID=21090435925) | [aljazeera.com](https://www.english-corpora.org/corona/x3.asp?node=&p=13&w10=coronavirus&w11=vaccination&r=) |  |  |  | 62 per cent of Israel's 9.3 million inhabitants have already received their first **coronavirus** **vaccination**, and more than 56 per cent have also received the second dose. # |
| [1209](https://www.english-corpora.org/corona/x4.asp?rs=&t=33066662&ID=21127327211) | [21-07-26 US](https://www.english-corpora.org/corona/x4.asp?rs=&t=33066662&ID=21127327211) | [fox4kc.com](https://www.english-corpora.org/corona/x3.asp?node=&p=13&w10=coronavirus&w11=vaccination&r=) |  |  |  | 2021, three vials of the Moderna COVID-19 Vaccine are pictured in a new **coronavirus** **vaccination** center at the' Velodrom' (velodrome-stadium) in Berlin, Germany. The |
| [1210](https://www.english-corpora.org/corona/x4.asp?rs=&t=33074730&ID=21147410515) | [21-07-28 US](https://www.english-corpora.org/corona/x4.asp?rs=&t=33074730&ID=21147410515) | [ndtv.com](https://www.english-corpora.org/corona/x3.asp?node=&p=13&w10=coronavirus&w11=vaccination&r=) |  |  |  | getting COVID vaccinated in rural areas than urban # New Delhi: India's **coronavirus** **vaccination** drive began on January 16 this year and is often touted as the world's |
| [1211](https://www.english-corpora.org/corona/x4.asp?rs=&t=33075368&ID=21153823666) | [21-07-28 US](https://www.english-corpora.org/corona/x4.asp?rs=&t=33075368&ID=21153823666) | [aljazeera.com](https://www.english-corpora.org/corona/x3.asp?node=&p=13&w10=coronavirus&w11=vaccination&r=) |  |  |  | workers, including 4,000 doctors, to further improve healthcare services and boost the **coronavirus** **vaccination** campaign nationwide. # A COVID-19 patient lies in a vehicle outside the Chittagong Medical |
| [1212](https://www.english-corpora.org/corona/x4.asp?rs=&t=33079006&ID=21161496543) | [21-07-29 US](https://www.english-corpora.org/corona/x4.asp?rs=&t=33079006&ID=21161496543) | [aljazeera.com](https://www.english-corpora.org/corona/x3.asp?node=&p=13&w10=coronavirus&w11=vaccination&r=) |  |  |  | his press conference, Powell noted risks to the economic outlook, including slowing **coronavirus** **vaccination** rates and the Delta strain of COVID-19 spreading in some areas of the country. |
| [1213](https://www.english-corpora.org/corona/x4.asp?rs=&t=43242003&ID=21076856405) | [21-07-21 US](https://www.english-corpora.org/corona/x4.asp?rs=&t=43242003&ID=21076856405) | [wsbtv.com](https://www.english-corpora.org/corona/x3.asp?node=&p=13&w10=coronavirus&w11=vaccination&r=) |  |  |  | gas and water cannons to disperse protesters who gathered Wednesday in Athens to oppose **coronavirus** **vaccination** requirements proposed by the Greek government. # The demonstration in front of the parliament |
| [1214](https://www.english-corpora.org/corona/x4.asp?rs=&t=43243265&ID=21079538013) | [21-07-21 US](https://www.english-corpora.org/corona/x4.asp?rs=&t=43243265&ID=21079538013) | [talkingpointsmemo.com](https://www.english-corpora.org/corona/x3.asp?node=&p=13&w10=coronavirus&w11=vaccination&r=) |  |  |  | Parson signed into law a related bill blocking local governments from requiring proof of **coronavirus** **vaccination** for people seeking to access transportation systems or other public services. # It's |
| [1215](https://www.english-corpora.org/corona/x4.asp?rs=&t=52954312&ID=21053508559) | [21-07-19 US](https://www.english-corpora.org/corona/x4.asp?rs=&t=52954312&ID=21053508559) | [pix11.com](https://www.english-corpora.org/corona/x3.asp?node=&p=13&w10=coronavirus&w11=vaccination&r=) |  |  |  | reported in the state since February. # Mississippi has one of the lowest **coronavirus** **vaccination** rates in the nation. # State Rep. Jeramey Anderson of Moss Point posted the |
| [1216](https://www.english-corpora.org/corona/x4.asp?rs=&t=52954706&ID=21053564155) | [21-07-19 US](https://www.english-corpora.org/corona/x4.asp?rs=&t=52954706&ID=21053564155) | [boston.com](https://www.english-corpora.org/corona/x3.asp?node=&p=13&w10=coronavirus&w11=vaccination&r=) |  |  |  | goal, " Brown wrote. # In April, Boston University announced their **coronavirus** **vaccination** requirement for all students to attend classes in the fall semester. The university has |
| [1217](https://www.english-corpora.org/corona/x4.asp?rs=&t=52965849&ID=21091201194) | [21-07-22 US](https://www.english-corpora.org/corona/x4.asp?rs=&t=52965849&ID=21091201194) | [mynorthwest.com](https://www.english-corpora.org/corona/x3.asp?node=&p=13&w10=coronavirus&w11=vaccination&r=) |  |  |  | gas and water cannons to disperse protesters who gathered Wednesday in Athens to oppose **coronavirus** **vaccination** requirements proposed by the Greek government. # The demonstration in front of the parliament |
| [1218](https://www.english-corpora.org/corona/x4.asp?rs=&t=52968511&ID=21099581324) | [21-07-23 US](https://www.english-corpora.org/corona/x4.asp?rs=&t=52968511&ID=21099581324) | [wpxi.com](https://www.english-corpora.org/corona/x3.asp?node=&p=13&w10=coronavirus&w11=vaccination&r=) |  |  |  | 2021, three vials of the Moderna COVID-19 Vaccine are pictured in a new **coronavirus** **vaccination** center at the' Velodrom' (velodrome-stadium) in Berlin, Germany. The |
| [1219](https://www.english-corpora.org/corona/x4.asp?rs=&t=52979464&ID=21140200630) | [21-07-27 US](https://www.english-corpora.org/corona/x4.asp?rs=&t=52979464&ID=21140200630) | [beaumontenterprise.com](https://www.english-corpora.org/corona/x3.asp?node=&p=13&w10=coronavirus&w11=vaccination&r=) |  |  |  | the suspension would be effective Thursday. # Louisiana has one of the lowest **coronavirus** **vaccination** rates in the nation, worsening this latest surge in COVID-19 cases fueled by the |
| [1220](https://www.english-corpora.org/corona/x4.asp?rs=&t=52989828&ID=21162439840) | [21-07-29 US](https://www.english-corpora.org/corona/x4.asp?rs=&t=52989828&ID=21162439840) | [abc7news.com](https://www.english-corpora.org/corona/x3.asp?node=&p=13&w10=coronavirus&w11=vaccination&r=) |  |  |  | private sector, Uber announced it'll delay office its reopening and make the **coronavirus** **vaccination** mandatory for U.S. employees. # Following in the footsteps of companies like Facebook and |
| [1221](https://www.english-corpora.org/corona/x4.asp?rs=&t=52990410&ID=21162500470) | [21-07-29 US](https://www.english-corpora.org/corona/x4.asp?rs=&t=52990410&ID=21162500470) | [mynorthwest.com](https://www.english-corpora.org/corona/x3.asp?node=&p=13&w10=coronavirus&w11=vaccination&r=) |  |  |  | file photo. Three New Orleans music venues are going to require proof of **coronavirus** **vaccination** or a negative test to get in the club. The clubs Tipitina's, |
| [1222](https://www.english-corpora.org/corona/x4.asp?rs=&t=52990410&ID=21162500580) | [21-07-29 US](https://www.english-corpora.org/corona/x4.asp?rs=&t=52990410&ID=21162500580) | [mynorthwest.com](https://www.english-corpora.org/corona/x3.asp?node=&p=13&w10=coronavirus&w11=vaccination&r=) |  |  |  | the most well-known New Orleans music venues, you'll have to get a **coronavirus** **vaccination** or a negative coronavirus test. # The clubs -- Tipitina's, the Maple |
| [1223](https://www.english-corpora.org/corona/x4.asp?rs=&t=63181419&ID=21149731466) | [21-07-28 US](https://www.english-corpora.org/corona/x4.asp?rs=&t=63181419&ID=21149731466) | [fox5sandiego.com](https://www.english-corpora.org/corona/x3.asp?node=&p=13&w10=coronavirus&w11=vaccination&r=) |  |  |  | at least partially vaccinated. Children under 12 are not eligible to receive a **coronavirus** **vaccination**. # " This is turning into the pandemic of the unvaccinated, so I |
| [1224](https://www.english-corpora.org/corona/x4.asp?rs=&t=73280990&ID=21132297048) | [21-07-26 US](https://www.english-corpora.org/corona/x4.asp?rs=&t=73280990&ID=21132297048) | [adn.com](https://www.english-corpora.org/corona/x3.asp?node=&p=13&w10=coronavirus&w11=vaccination&r=) |  |  |  | which has said it was the nation's first health system to impose a **coronavirus** **vaccination** mandate. Some staff vocally opposed the rule - including more than 150 workers who |
| [1225](https://www.english-corpora.org/corona/x4.asp?rs=&t=73280990&ID=21132297144) | [21-07-26 US](https://www.english-corpora.org/corona/x4.asp?rs=&t=73280990&ID=21132297144) | [adn.com](https://www.english-corpora.org/corona/x3.asp?node=&p=13&w10=coronavirus&w11=vaccination&r=) |  |  |  | Emanuel said that the University of Pennsylvania Health System, which imposed its own **coronavirus** **vaccination** mandate two months ago, also has seen a similar uptake in shots. # |
| [1226](https://www.english-corpora.org/corona/x4.asp?rs=&t=73281015&ID=21126896651) | [21-07-26 US](https://www.english-corpora.org/corona/x4.asp?rs=&t=73281015&ID=21126896651) | [wbal.com](https://www.english-corpora.org/corona/x3.asp?node=&p=13&w10=coronavirus&w11=vaccination&r=) |  |  |  | procedures that might require overnight stays. # Louisiana has one of the lowest **coronavirus** **vaccination** rates in the nation, worsening this latest surge in COVID-19 cases fueled by the |
| [1227](https://www.english-corpora.org/corona/x4.asp?rs=&t=73289789&ID=21156631727) | [21-07-28 US](https://www.english-corpora.org/corona/x4.asp?rs=&t=73289789&ID=21156631727) | [news.trust.org](https://www.english-corpora.org/corona/x3.asp?node=&p=13&w10=coronavirus&w11=vaccination&r=) |  |  |  | ) - Philippine President Rodrigo Duterte ordered his government on Wednesday to open the **coronavirus** **vaccination** campaign to anyone who wants a shot as his country scrambles to protect the population |
| [1228](https://www.english-corpora.org/corona/x4.asp?rs=&t=87596224&ID=21029097325) | [21-07-16 US](https://www.english-corpora.org/corona/x4.asp?rs=&t=87596224&ID=21029097325) | [CNBC](https://www.english-corpora.org/corona/x3.asp?node=&p=13&w10=coronavirus&w11=vaccination&r=) |  |  |  | this weekend and mask restrictions have been loosened for those that have received the **coronavirus** **vaccination**. While the box office hasn't quite caught up to the ticket sales it |
| [1230](https://www.english-corpora.org/corona/x4.asp?rs=&t=87603780&ID=21038097898) | [21-07-17 US](https://www.english-corpora.org/corona/x4.asp?rs=&t=87603780&ID=21038097898) | [New York Times](https://www.english-corpora.org/corona/x3.asp?node=&p=13&w10=coronavirus&w11=vaccination&r=) [(1)](https://www.english-corpora.org/corona/duplicates1.asp?xx=1229&n=1) |  |  |  | for any state or local entity, including public hospitals, to require **coronavirus** **vaccination** as a condition of education or employment until two years after the Food and Drug |
| [1231](https://www.english-corpora.org/corona/x4.asp?rs=&t=87612089&ID=21062931810) | [21-07-19 US](https://www.english-corpora.org/corona/x4.asp?rs=&t=87612089&ID=21062931810) | [New York Post](https://www.english-corpora.org/corona/x3.asp?node=&p=13&w10=coronavirus&w11=vaccination&r=) |  |  |  | dean at the Medical College of Wisconsin. # Differences are also seen in **coronavirus** **vaccination** rates, with 27.5% of Blacks, about 34% of American Indians, 37.6% of |
| [1235](https://www.english-corpora.org/corona/x4.asp?rs=&t=87620211&ID=21071406392) | [21-07-20 US](https://www.english-corpora.org/corona/x4.asp?rs=&t=87620211&ID=21071406392) | [YAHOO!News](https://www.english-corpora.org/corona/x3.asp?node=&p=13&w10=coronavirus&w11=vaccination&r=) [(3)](https://www.english-corpora.org/corona/duplicates1.asp?xx=1234&n=3) |  |  |  | Boris Johnson is facing a huge backlash over his plans to make full **coronavirus** **vaccination** compulsory for entry to nightclubs and other crowded venues. # The prime minister made |
| [1236](https://www.english-corpora.org/corona/x4.asp?rs=&t=87622829&ID=21072754844) | [21-07-20 US](https://www.english-corpora.org/corona/x4.asp?rs=&t=87622829&ID=21072754844) | [New York Times](https://www.english-corpora.org/corona/x3.asp?node=&p=13&w10=coronavirus&w11=vaccination&r=) |  |  |  | the shots' safety and effectiveness. # Amid a widening partisan divide over **coronavirus** **vaccination**, most Republicans have either stoked or ignored the flood of misinformation reaching their constituents |
| [1237](https://www.english-corpora.org/corona/x4.asp?rs=&t=87624060&ID=21073084702) | [21-07-20 US](https://www.english-corpora.org/corona/x4.asp?rs=&t=87624060&ID=21073084702) | [Yahoo](https://www.english-corpora.org/corona/x3.asp?node=&p=13&w10=coronavirus&w11=vaccination&r=) |  |  |  | of the time, and it is not people who have gotten their full **coronavirus** **vaccination**. # " As the CDC director said, this is a pandemic of the |
| [1238](https://www.english-corpora.org/corona/x4.asp?rs=&t=87625510&ID=21083497661) | [21-07-21 US](https://www.english-corpora.org/corona/x4.asp?rs=&t=87625510&ID=21083497661) | [YAHOO!Finance](https://www.english-corpora.org/corona/x3.asp?node=&p=13&w10=coronavirus&w11=vaccination&r=) |  |  |  | about the shots' safety and effectiveness. Amid a widening partisan divide over **coronavirus** **vaccination**, most Republicans have either stoked or ignored the flood of misinformation reaching their constituents |
| [1239](https://www.english-corpora.org/corona/x4.asp?rs=&t=87626125&ID=21084248095) | [21-07-21 US](https://www.english-corpora.org/corona/x4.asp?rs=&t=87626125&ID=21084248095) | [YAHOO!News](https://www.english-corpora.org/corona/x3.asp?node=&p=13&w10=coronavirus&w11=vaccination&r=) |  |  |  | gas and water cannons to disperse protesters who gathered Wednesday in Athens to oppose **coronavirus** **vaccination** requirements proposed by the Greek government. The demonstration in front of the parliament building |
| [1240](https://www.english-corpora.org/corona/x4.asp?rs=&t=87627152&ID=21085354943) | [21-07-21 US](https://www.english-corpora.org/corona/x4.asp?rs=&t=87627152&ID=21085354943) | [YAHOO!News](https://www.english-corpora.org/corona/x3.asp?node=&p=13&w10=coronavirus&w11=vaccination&r=) |  |  |  | about the shots' safety and effectiveness. Amid a widening partisan divide over **coronavirus** **vaccination**, most Republicans have either stoked or ignored the flood of misinformation reaching their constituents |
| [1241](https://www.english-corpora.org/corona/x4.asp?rs=&t=87627318&ID=21084812497) | [21-07-21 US](https://www.english-corpora.org/corona/x4.asp?rs=&t=87627318&ID=21084812497) | [Al Jazeera on MSN.com](https://www.english-corpora.org/corona/x3.asp?node=&p=13&w10=coronavirus&w11=vaccination&r=) |  |  |  | , Mexico's Foreign Minister Marcelo Ebrard said the country planned to bolster its **coronavirus** **vaccination** effort along the Mexican-US border in an effort to get the restrictions lifted. # |
| [1242](https://www.english-corpora.org/corona/x4.asp?rs=&t=87637762&ID=21105634280) | [21-07-23 US](https://www.english-corpora.org/corona/x4.asp?rs=&t=87637762&ID=21105634280) | [The Motley Fool](https://www.english-corpora.org/corona/x3.asp?node=&p=13&w10=coronavirus&w11=vaccination&r=) |  |  |  | keep people out of the hospital. # As the U.S. rolled out its **coronavirus** **vaccination** campaign, cases of COVID-19 plunged -- and Americans allowed themselves to imagine a return |
| [1243](https://www.english-corpora.org/corona/x4.asp?rs=&t=87645002&ID=21113891214) | [21-07-24 US](https://www.english-corpora.org/corona/x4.asp?rs=&t=87645002&ID=21113891214) | [YAHOO!News](https://www.english-corpora.org/corona/x3.asp?node=&p=13&w10=coronavirus&w11=vaccination&r=) |  |  |  | is heating up again, with increasing calls for all Americans, regardless of **coronavirus** **vaccination** status, to return to wearing face coverings in indoor public places to help thwart |
| [1244](https://www.english-corpora.org/corona/x4.asp?rs=&t=87647801&ID=21120858131) | [21-07-25 US](https://www.english-corpora.org/corona/x4.asp?rs=&t=87647801&ID=21120858131) | [YAHOO!News](https://www.english-corpora.org/corona/x3.asp?node=&p=13&w10=coronavirus&w11=vaccination&r=) |  |  |  | with the virus, Mr Harmon refused the Covid vaccine and posted jokes about **coronavirus** **vaccination** online. On social media he claimed that he wasn't " anti-vax " but |
| [1245](https://www.english-corpora.org/corona/x4.asp?rs=&t=87655199&ID=21136097647) | [21-07-26 US](https://www.english-corpora.org/corona/x4.asp?rs=&t=87655199&ID=21136097647) | [The Washington Post on MSN.com](https://www.english-corpora.org/corona/x3.asp?node=&p=13&w10=coronavirus&w11=vaccination&r=) |  |  |  | which has said it was the nation's first health system to impose a **coronavirus** **vaccination** mandate when the policy was announced in March, faced protests from some staffers -- |
| [1246](https://www.english-corpora.org/corona/x4.asp?rs=&t=87655199&ID=21136097745) | [21-07-26 US](https://www.english-corpora.org/corona/x4.asp?rs=&t=87655199&ID=21136097745) | [The Washington Post on MSN.com](https://www.english-corpora.org/corona/x3.asp?node=&p=13&w10=coronavirus&w11=vaccination&r=) |  |  |  | # Emanuel said the University of Pennsylvania Health System, which imposed its own **coronavirus** **vaccination** mandate two months ago, also has seen a similar uptake in shots. # |
| [1247](https://www.english-corpora.org/corona/x4.asp?rs=&t=87656172&ID=21135386778) | [21-07-26 US](https://www.english-corpora.org/corona/x4.asp?rs=&t=87656172&ID=21135386778) | [New York Times](https://www.english-corpora.org/corona/x3.asp?node=&p=13&w10=coronavirus&w11=vaccination&r=) |  |  |  | parents and teachers. Regulators will be required to balance potential side effects of **coronavirus** **vaccination** against the risks of Covid-19. # Members of a Centers for Disease Control and |
| [1248](https://www.english-corpora.org/corona/x4.asp?rs=&t=87664427&ID=21157823800) | [21-07-28 US](https://www.english-corpora.org/corona/x4.asp?rs=&t=87664427&ID=21157823800) | [YAHOO!Finance](https://www.english-corpora.org/corona/x3.asp?node=&p=13&w10=coronavirus&w11=vaccination&r=) |  |  |  | much patience for. " # Carolina Panthers quarterback Sam Darnold has had two **coronavirus** **vaccination** shots and will be considered fully vaccinated on July 30, according to a person |
| [1250](https://www.english-corpora.org/corona/x4.asp?rs=&t=87668367&ID=21160937849) | [21-07-28 US](https://www.english-corpora.org/corona/x4.asp?rs=&t=87668367&ID=21160937849) | [New York Daily News](https://www.english-corpora.org/corona/x3.asp?node=&p=13&w10=coronavirus&w11=vaccination&r=) [(1)](https://www.english-corpora.org/corona/duplicates1.asp?xx=1249&n=1) |  |  |  | practices get underway. (Nick Wass/AP) # The Ravens tout a **coronavirus** **vaccination** rate of about 90%, one of the highest in the league. Their entire |
| [1251](https://www.english-corpora.org/corona/x4.asp?rs=&t=87670496&ID=21170593731) | [21-07-29 US](https://www.english-corpora.org/corona/x4.asp?rs=&t=87670496&ID=21170593731) | [Business Insider](https://www.english-corpora.org/corona/x3.asp?node=&p=13&w10=coronavirus&w11=vaccination&r=) |  |  |  | Israel's advanced health data records had given it a huge advantage in the **coronavirus** **vaccination** roll-out and looking to the future the fact that Israel has the world's second-largest |
| [1252](https://www.english-corpora.org/corona/x4.asp?rs=&t=87670887&ID=21170612155) | [21-07-29 US](https://www.english-corpora.org/corona/x4.asp?rs=&t=87670887&ID=21170612155) | [ABC7 San Francisco](https://www.english-corpora.org/corona/x3.asp?node=&p=13&w10=coronavirus&w11=vaccination&r=) |  |  |  | private sector, Uber announced it'll delay office its reopening and make the **coronavirus** **vaccination** mandatory for U.S. employees. # Following in the footsteps of companies like Facebook and |
| [1253](https://www.english-corpora.org/corona/x4.asp?rs=&t=87671935&ID=21169002863) | [21-07-29 US](https://www.english-corpora.org/corona/x4.asp?rs=&t=87671935&ID=21169002863) | [The Independent on MSN.com](https://www.english-corpora.org/corona/x3.asp?node=&p=13&w10=coronavirus&w11=vaccination&r=) |  |  |  | ) will be accessible only for people who have had completed a course of **coronavirus** **vaccination** at least two weeks earlier. # It does not apply to citizens and residents |
| [1254](https://www.english-corpora.org/corona/x4.asp?rs=&t=87671979&ID=21170960078) | [21-07-29 US](https://www.english-corpora.org/corona/x4.asp?rs=&t=87671979&ID=21170960078) | [CNBC](https://www.english-corpora.org/corona/x3.asp?node=&p=13&w10=coronavirus&w11=vaccination&r=) |  |  |  | be required to prove their **coronavirus** **vaccination** status or submit to a series of rigorous safety protocols, President Joe Biden announced |
| [1255](https://www.english-corpora.org/corona/x4.asp?rs=&t=87672415&ID=21167695861) | [21-07-29 US](https://www.english-corpora.org/corona/x4.asp?rs=&t=87672415&ID=21167695861) | [SFGate](https://www.english-corpora.org/corona/x3.asp?node=&p=13&w10=coronavirus&w11=vaccination&r=) |  |  |  | file photo. Three New Orleans music venues are going to require proof of **coronavirus** **vaccination** or a negative test to get in the club. The clubs Tipitina's, |
| [1257](https://www.english-corpora.org/corona/x4.asp?rs=&t=87673182&ID=21170200060) | [21-07-29 US](https://www.english-corpora.org/corona/x4.asp?rs=&t=87673182&ID=21170200060) | [Washington Post](https://www.english-corpora.org/corona/x3.asp?node=&p=13&w10=coronavirus&w11=vaccination&r=) [(1)](https://www.english-corpora.org/corona/duplicates1.asp?xx=1256&n=1) |  |  |  | but Biden said that he would ask the Defense Department to examine adding **coronavirus** **vaccination** to the list of inoculations that military members are already required to have. # |
| [1258](https://www.english-corpora.org/corona/x4.asp?rs=&t=87673292&ID=21170207161) | [21-07-29 US](https://www.english-corpora.org/corona/x4.asp?rs=&t=87673292&ID=21170207161) | [Fox News](https://www.english-corpora.org/corona/x3.asp?node=&p=13&w10=coronavirus&w11=vaccination&r=) |  |  |  | Huntington Beach, Orange County, has taken a stand against **coronavirus** **vaccination** policies, calling them " treasonous, anti-American stupidity, " in a sign posted |
| [1259](https://www.english-corpora.org/corona/x4.asp?rs=&t=52960669&ID=21082280469) | [21-07-21 CA](https://www.english-corpora.org/corona/x4.asp?rs=&t=52960669&ID=21082280469) | [digitaljournal.com](https://www.english-corpora.org/corona/x3.asp?node=&p=13&w10=coronavirus&w11=vaccination&r=) |  |  |  | --' Race against time' # Tunisia has also struggled to get its **coronavirus** **vaccination** campaign off the ground. Fewer than a million people have been fully vaccinated, |
| [1260](https://www.english-corpora.org/corona/x4.asp?rs=&t=52967263&ID=21101156507) | [21-07-23 CA](https://www.english-corpora.org/corona/x4.asp?rs=&t=52967263&ID=21101156507) | [ca.sports.yahoo.com](https://www.english-corpora.org/corona/x3.asp?node=&p=13&w10=coronavirus&w11=vaccination&r=) |  |  |  | was facing a backlash over his plans to make **coronavirus** **vaccination** compulsory for nightclubs and other crowded venues in the autumn. # Clubs, backbench |
| [1261](https://www.english-corpora.org/corona/x4.asp?rs=&t=52983387&ID=21154872592) | [21-07-28 CA](https://www.english-corpora.org/corona/x4.asp?rs=&t=52983387&ID=21154872592) | [prpeak.com](https://www.english-corpora.org/corona/x3.asp?node=&p=13&w10=coronavirus&w11=vaccination&r=) |  |  |  | he does not plan to require the use of masks in schools though the **coronavirus** **vaccination** rate in Mississippi remains among the lowest in the nation. Health official say they |
| [1262](https://www.english-corpora.org/corona/x4.asp?rs=&t=33047471&ID=21068730595) | [21-07-20 GB](https://www.english-corpora.org/corona/x4.asp?rs=&t=33047471&ID=21068730595) | [portsmouth.co.uk](https://www.english-corpora.org/corona/x3.asp?node=&p=13&w10=coronavirus&w11=vaccination&r=) |  |  |  | a message on its website, adding that it will prioritise the delivery of **coronavirus** **vaccination** letters and testing kits. # The company said it aims to deliver at least |
| [1263](https://www.english-corpora.org/corona/x4.asp?rs=&t=43267711&ID=21161858080) | [21-07-29 GB](https://www.english-corpora.org/corona/x4.asp?rs=&t=43267711&ID=21161858080) | [churchtimes.co.uk](https://www.english-corpora.org/corona/x3.asp?node=&p=13&w10=coronavirus&w11=vaccination&r=) |  |  |  | London, reminds those aged 18 and over that they are eligible for a **coronavirus** **vaccination** # A painted sign on the pavement at a pedestrian crossing in Brixton, south |
| [1264](https://www.english-corpora.org/corona/x4.asp?rs=&t=43269162&ID=21163911672) | [21-07-29 GB](https://www.english-corpora.org/corona/x4.asp?rs=&t=43269162&ID=21163911672) | [kentonline.co.uk](https://www.english-corpora.org/corona/x3.asp?node=&p=13&w10=coronavirus&w11=vaccination&r=) |  |  |  | Thomas # Everyone over the age of 18 is now invited to have their **coronavirus** **vaccination**. # Cllr Thomas said " a huge effort " has gone into contacting people |
| [1265](https://www.english-corpora.org/corona/x4.asp?rs=&t=63161222&ID=21089473666) | [21-07-22 GB](https://www.english-corpora.org/corona/x4.asp?rs=&t=63161222&ID=21089473666) | [dailypost.co.uk](https://www.english-corpora.org/corona/x3.asp?node=&p=13&w10=coronavirus&w11=vaccination&r=) |  |  |  | validate your NHS Covid Pass. In Scotland you can get a record of **coronavirus** **vaccination** status. In Northern Ireland you can apply for a vaccine certificate Get the |
| [1266](https://www.english-corpora.org/corona/x4.asp?rs=&t=87640976&ID=21107769628) | [21-07-23 GB](https://www.english-corpora.org/corona/x4.asp?rs=&t=87640976&ID=21107769628) | [The Sun](https://www.english-corpora.org/corona/x3.asp?node=&p=13&w10=coronavirus&w11=vaccination&r=) |  |  |  | Gareth Southgate has urged young people to get their freedom back by supporting the **coronavirus** **vaccination** programme. In the UK, more than 46 million people have now had a |
| [1267](https://www.english-corpora.org/corona/x4.asp?rs=&t=63162586&ID=21103062228) | [21-07-23 IE](https://www.english-corpora.org/corona/x4.asp?rs=&t=63162586&ID=21103062228) | [trtworld.com](https://www.english-corpora.org/corona/x3.asp?node=&p=13&w10=coronavirus&w11=vaccination&r=) |  |  |  | minister of making " criminal " decisions that led to overcrowding and chaos at **coronavirus** **vaccination** centres. # The centres opened for young people on Tuesday and tens of thousands |
| [1268](https://www.english-corpora.org/corona/x4.asp?rs=&t=63176997&ID=21155460889) | [21-07-28 IE](https://www.english-corpora.org/corona/x4.asp?rs=&t=63176997&ID=21155460889) | [intellinews.com](https://www.english-corpora.org/corona/x3.asp?node=&p=13&w10=coronavirus&w11=vaccination&r=) |  |  |  | . The Iraqi population numbers 39mn. # Iran is attempting to accelerate its **coronavirus** **vaccination** programme. Tehran has several times complained that US sanctions, which Washington says are |
| [1269](https://www.english-corpora.org/corona/x4.asp?rs=&t=73259829&ID=21077981126) | [21-07-21 AU](https://www.english-corpora.org/corona/x4.asp?rs=&t=73259829&ID=21077981126) | [canberratimes.com.au](https://www.english-corpora.org/corona/x3.asp?node=&p=13&w10=coronavirus&w11=vaccination&r=) |  |  |  | close its border to South Australia and expand its **coronavirus** **vaccination** rollout which is the worst-performing in the nation. Premier Mark McGowan says the hard |
| [1272](https://www.english-corpora.org/corona/x4.asp?rs=&t=73271422&ID=21109701082) | [21-07-24 AU](https://www.english-corpora.org/corona/x4.asp?rs=&t=73271422&ID=21109701082) | [au.news.yahoo.com](https://www.english-corpora.org/corona/x3.asp?node=&p=13&w10=coronavirus&w11=vaccination&r=) [(2)](https://www.english-corpora.org/corona/duplicates1.asp?xx=1271&n=2) |  |  |  | per cent of all Icelanders over age 16 have received at least one **coronavirus** **vaccination**. # Iceland had watched cases peter out until just recently: The domestic 14-day |
| [1273](https://www.english-corpora.org/corona/x4.asp?rs=&t=73291283&ID=21166594893) | [21-07-29 AU](https://www.english-corpora.org/corona/x4.asp?rs=&t=73291283&ID=21166594893) | [mamamia.com.au](https://www.english-corpora.org/corona/x3.asp?node=&p=13&w10=coronavirus&w11=vaccination&r=) |  |  |  | leaders will start charting a course to fully reopening the nation based on new **coronavirus** **vaccination** rate modelling. # Prime Minister Scott Morrison will on Friday chair a national cabinet |
| [1274](https://www.english-corpora.org/corona/x4.asp?rs=&t=33049659&ID=21078436621) | [21-07-21 NZ](https://www.english-corpora.org/corona/x4.asp?rs=&t=33049659&ID=21078436621) | [nz.news.yahoo.com](https://www.english-corpora.org/corona/x3.asp?node=&p=13&w10=coronavirus&w11=vaccination&r=) |  |  |  | to more child abuse material. # More pharmacies will be brought into the **coronavirus** **vaccination** rollout sooner under a plan to fuel national jab rates.More than 110 have started administering |
| [1280](https://www.english-corpora.org/corona/x4.asp?rs=&t=73256571&ID=21070828407) | [21-07-20 IN](https://www.english-corpora.org/corona/x4.asp?rs=&t=73256571&ID=21070828407) | [ibtimes.co.in](https://www.english-corpora.org/corona/x3.asp?node=&p=13&w10=coronavirus&w11=vaccination&r=) [(5)](https://www.english-corpora.org/corona/duplicates1.asp?xx=1279&n=5) |  |  |  | 318 per 1,000 people. # Adding up to the worries, the **coronavirus** **vaccination** is progressing in Indonesia at a snail pace, and it could result in more |
| [1281](https://www.english-corpora.org/corona/x4.asp?rs=&t=73256602&ID=21065550987) | [21-07-20 IN](https://www.english-corpora.org/corona/x4.asp?rs=&t=73256602&ID=21065550987) | [ibtimes.co.in](https://www.english-corpora.org/corona/x3.asp?node=&p=13&w10=coronavirus&w11=vaccination&r=) |  |  |  | more than 48.5 percent of the American population are already vaccinated # As the **coronavirus** **vaccination** rollout is progressing steadily in various countries, a top expert has warned that Covid |
| [1282](https://www.english-corpora.org/corona/x4.asp?rs=&t=73265160&ID=21092042441) | [21-07-22 IN](https://www.english-corpora.org/corona/x4.asp?rs=&t=73265160&ID=21092042441) | [ibtimes.co.in](https://www.english-corpora.org/corona/x3.asp?node=&p=13&w10=coronavirus&w11=vaccination&r=) |  |  |  | The dire warning regarding a possible third wave comes at a time when the **coronavirus** **vaccination** rollout in the country is progressing in full swing. And now, a nationwide |
| [1283](https://www.english-corpora.org/corona/x4.asp?rs=&t=73277251&ID=21129127436) | [21-07-26 IN](https://www.english-corpora.org/corona/x4.asp?rs=&t=73277251&ID=21129127436) | [theweek.in](https://www.english-corpora.org/corona/x3.asp?node=&p=13&w10=coronavirus&w11=vaccination&r=) |  |  |  | . The bench passed the directions on a number of PILs with regard to **coronavirus** **vaccination** and adherence to restrictions imposed by the government to deal with the disease. The |
| [1285](https://www.english-corpora.org/corona/x4.asp?rs=&t=52949127&ID=21040541166) | [21-07-18 PK](https://www.english-corpora.org/corona/x4.asp?rs=&t=52949127&ID=21040541166) | [tribune.com.pk](https://www.english-corpora.org/corona/x3.asp?node=&p=13&w10=coronavirus&w11=vaccination&r=) [(1)](https://www.english-corpora.org/corona/duplicates1.asp?xx=1284&n=1) |  |  |  | # Contract-based data entry operators, working on the registration desk at the **coronavirus** **vaccination** centre of the Karachi Expo Centre, resorted to protests and stopped working as they |
| [1286](https://www.english-corpora.org/corona/x4.asp?rs=&t=52949127&ID=21040541502) | [21-07-18 PK](https://www.english-corpora.org/corona/x4.asp?rs=&t=52949127&ID=21040541502) | [tribune.com.pk](https://www.english-corpora.org/corona/x3.asp?node=&p=13&w10=coronavirus&w11=vaccination&r=) |  |  |  | # The process of vaccination has been resumed at the venue. The biggest **coronavirus** **vaccination** centre of the province is set up at the Karachi Expo where people above 18 |
| [1287](https://www.english-corpora.org/corona/x4.asp?rs=&t=52949434&ID=21043638074) | [21-07-18 PK](https://www.english-corpora.org/corona/x4.asp?rs=&t=52949434&ID=21043638074) | [dailytimes.com.pk](https://www.english-corpora.org/corona/x3.asp?node=&p=13&w10=coronavirus&w11=vaccination&r=) |  |  |  | rate now higher than in US # European Union leaders on Saturday celebrated their **coronavirus** **vaccination** programme reaching a higher proportion of its people than in the US, which had |
| [1288](https://www.english-corpora.org/corona/x4.asp?rs=&t=52957891&ID=21069631414) | [21-07-20 PK](https://www.english-corpora.org/corona/x4.asp?rs=&t=52957891&ID=21069631414) | [samaa.tv](https://www.english-corpora.org/corona/x3.asp?node=&p=13&w10=coronavirus&w11=vaccination&r=) |  |  |  | this purpose. # The government has, on the other hand, made **coronavirus** **vaccination** mandatory for everyone. People won't be allowed inside gyms, restaurants, hotels |
| [1289](https://www.english-corpora.org/corona/x4.asp?rs=&t=52970211&ID=21109186629) | [21-07-24 PK](https://www.english-corpora.org/corona/x4.asp?rs=&t=52970211&ID=21109186629) | [samaa.tv](https://www.english-corpora.org/corona/x3.asp?node=&p=13&w10=coronavirus&w11=vaccination&r=) |  |  |  | vaccinated against the novel coronavirus. The National Command and Operation Centre has made **coronavirus** **vaccination** certificates mandatory for domestic air travel from August 1. Get yourself vaccinated by July |
| [1291](https://www.english-corpora.org/corona/x4.asp?rs=&t=52973289&ID=21120154265) | [21-07-25 PK](https://www.english-corpora.org/corona/x4.asp?rs=&t=52973289&ID=21120154265) | [tribune.com.pk](https://www.english-corpora.org/corona/x3.asp?node=&p=13&w10=coronavirus&w11=vaccination&r=) [(1)](https://www.english-corpora.org/corona/duplicates1.asp?xx=1290&n=1) |  |  |  | # Meanwhile, the Punjab government decided on Saturday to launch a door-to-door **coronavirus** **vaccination** campaign from July 26 (tomorrow) in five cities of the province -- Lahore |
| [1292](https://www.english-corpora.org/corona/x4.asp?rs=&t=52974135&ID=21118974627) | [21-07-25 PK](https://www.english-corpora.org/corona/x4.asp?rs=&t=52974135&ID=21118974627) | [samaa.tv](https://www.english-corpora.org/corona/x3.asp?node=&p=13&w10=coronavirus&w11=vaccination&r=) |  |  |  | vaccinated against the novel coronavirus. The National Command and Operation Centre has made **coronavirus** **vaccination** certificates mandatory for domestic air travel from August 1. |
| [1294](https://www.english-corpora.org/corona/x4.asp?rs=&t=52984579&ID=21155028494) | [21-07-28 PK](https://www.english-corpora.org/corona/x4.asp?rs=&t=52984579&ID=21155028494) | [nation.com.pk](https://www.english-corpora.org/corona/x3.asp?node=&p=13&w10=coronavirus&w11=vaccination&r=) [(1)](https://www.english-corpora.org/corona/duplicates1.asp?xx=1293&n=1) |  |  |  | minister urged citizens. # " There are no side effects of the **coronavirus** **vaccination**, " Rahu said. The citizens should avoid bringing elders and children to vegetable |
| [1295](https://www.english-corpora.org/corona/x4.asp?rs=&t=43248608&ID=21099355598) | [21-07-23 SG](https://www.english-corpora.org/corona/x4.asp?rs=&t=43248608&ID=21099355598) | [sundayguardianlive.com](https://www.english-corpora.org/corona/x3.asp?node=&p=13&w10=coronavirus&w11=vaccination&r=) |  |  |  | vaccinated, according to the Russian website, Gogov, which gives the official **coronavirus** **vaccination** statistics. The Kremlin has conceded that it is now impossible to reach its 60% |
| [1296](https://www.english-corpora.org/corona/x4.asp?rs=&t=73256752&ID=21068229523) | [21-07-20 PH](https://www.english-corpora.org/corona/x4.asp?rs=&t=73256752&ID=21068229523) | [news.abs-cbn.com](https://www.english-corpora.org/corona/x3.asp?node=&p=13&w10=coronavirus&w11=vaccination&r=) |  |  |  | # JERUSALEM - Israel on Monday walked back part of its policy for third **coronavirus** **vaccination** shots, saying that contrary to a recent announcement, oncology patients should actually stick |
| [1297](https://www.english-corpora.org/corona/x4.asp?rs=&t=73278667&ID=21132014002) | [21-07-26 PH](https://www.english-corpora.org/corona/x4.asp?rs=&t=73278667&ID=21132014002) | [sunstar.com.ph](https://www.english-corpora.org/corona/x3.asp?node=&p=13&w10=coronavirus&w11=vaccination&r=) |  |  |  | - # July 26, 2021 # BAGUIO CITY -- Persons who use fake **coronavirus** **vaccination** cards to avoid undergoing testing or to avail themselves of benefits face charges of falsification |
| [1298](https://www.english-corpora.org/corona/x4.asp?rs=&t=63148922&ID=21053931798) | [21-07-19 ZA](https://www.english-corpora.org/corona/x4.asp?rs=&t=63148922&ID=21053931798) | [businesslive.co.za](https://www.english-corpora.org/corona/x3.asp?node=&p=13&w10=coronavirus&w11=vaccination&r=) |  |  |  | July 2021 - 19:34 Tamar Kahn # The government expects to reach a daily **coronavirus** **vaccination** target of 250,000 doses a day by the end of the week, as inoculation |
| [1299](https://www.english-corpora.org/corona/x4.asp?rs=&t=63184342&ID=21164560238) | [21-07-29 ZA](https://www.english-corpora.org/corona/x4.asp?rs=&t=63184342&ID=21164560238) | [heraldlive.co.za](https://www.english-corpora.org/corona/x3.asp?node=&p=13&w10=coronavirus&w11=vaccination&r=) |  |  |  | enhance its world-class parks, reserves and heritage sites. # With SA's **coronavirus** **vaccination** programme now on track, the tourism sector is hard at work preparing for the |
| [1300](https://www.english-corpora.org/corona/x4.asp?rs=&t=63178124&ID=21149287483) | [21-07-28 KE](https://www.english-corpora.org/corona/x4.asp?rs=&t=63178124&ID=21149287483) | [capitalfm.co.ke](https://www.english-corpora.org/corona/x3.asp?node=&p=13&w10=coronavirus&w11=vaccination&r=) |  |  |  | announced on Tuesday that it would require city officials to provide proof of a **coronavirus** **vaccination** or a weekly negative test. # " The fourth wave is here, and |

PAGE:   [**<<**](https://www.english-corpora.org/corona/x3.asp?node=&p=1&w10=coronavirus&w11=vaccination&r=)  [**<**](https://www.english-corpora.org/corona/x3.asp?node=&p=12&w10=coronavirus&w11=vaccination&r=)   13 / 42  [**>**](https://www.english-corpora.org/corona/x3.asp?node=&p=14&w10=coronavirus&w11=vaccination&r=)   [**>>**](https://www.english-corpora.org/corona/x3.asp?node=&p=42&w10=coronavirus&w11=vaccination&r=)

Конец формы

| PAGE:   [**<<**](https://www.english-corpora.org/corona/x3.asp?node=&p=1&w10=coronavirus&w11=vaccination&r=)  [**<**](https://www.english-corpora.org/corona/x3.asp?node=&p=13&w10=coronavirus&w11=vaccination&r=)   14 / 42  [**>**](https://www.english-corpora.org/corona/x3.asp?node=&p=15&w10=coronavirus&w11=vaccination&r=)   [**>>**](https://www.english-corpora.org/corona/x3.asp?node=&p=42&w10=coronavirus&w11=vaccination&r=) |  |
| --- | --- |

Начало формы

| CLICK FOR MORE CONTEXT | [**HELP**](javascript:newFeatures()) | [SAVE](javascript:chooser('s'))    [TRANSLATE](javascript:chooser('t'))    [ANALYZE](javascript:chooser('p')) |
| --- | --- | --- |

| [1301](https://www.english-corpora.org/corona/x4.asp?rs=&t=33007973&ID=20933225662) | [21-07-08 US](https://www.english-corpora.org/corona/x4.asp?rs=&t=33007973&ID=20933225662) | [thehill.com](https://www.english-corpora.org/corona/x3.asp?node=&p=14&w10=coronavirus&w11=vaccination&r=) |  |  |  | U.S. approaches a **coronavirus** **vaccination** rate of 70 percent, racial disparities in who has gotten vaccinated persist, raising |
| --- | --- | --- | --- | --- | --- | --- |
| [1302](https://www.english-corpora.org/corona/x4.asp?rs=&t=33012897&ID=20947071798) | [21-07-09 US](https://www.english-corpora.org/corona/x4.asp?rs=&t=33012897&ID=20947071798) | [politico.com](https://www.english-corpora.org/corona/x3.asp?node=&p=14&w10=coronavirus&w11=vaccination&r=) |  |  |  | Thursday that " it is absolutely the government's business " to push for **coronavirus** **vaccination**. # " It is taxpayers' business, if we have to continue to |
| [1303](https://www.english-corpora.org/corona/x4.asp?rs=&t=33013176&ID=20947152269) | [21-07-09 US](https://www.english-corpora.org/corona/x4.asp?rs=&t=33013176&ID=20947152269) | [thehill.com](https://www.english-corpora.org/corona/x3.asp?node=&p=14&w10=coronavirus&w11=vaccination&r=) |  |  |  | and Arkansas. # Yet Parson pushed back on the Biden administration's door-to-door **coronavirus** **vaccination** strategy, arguing that it was not a " welcome strategy. " # He |
| [1304](https://www.english-corpora.org/corona/x4.asp?rs=&t=33021632&ID=20965702509) | [21-07-11 US](https://www.english-corpora.org/corona/x4.asp?rs=&t=33021632&ID=20965702509) | [nydailynews.com](https://www.english-corpora.org/corona/x3.asp?node=&p=14&w10=coronavirus&w11=vaccination&r=) |  |  |  | York's mass **coronavirus** **vaccination** sites -- including the hub at Manhattan's Javits Center -- will shut down this |
| [1305](https://www.english-corpora.org/corona/x4.asp?rs=&t=33033709&ID=21007583844) | [21-07-15 US](https://www.english-corpora.org/corona/x4.asp?rs=&t=33033709&ID=21007583844) | [timesunion.com](https://www.english-corpora.org/corona/x3.asp?node=&p=14&w10=coronavirus&w11=vaccination&r=) |  |  |  | Bruce Vanderhoff, chief medical officer for the Ohio Department of Health, said **coronavirus** **vaccination** trends have led to the development of " two Ohios " just as the highly |
| [1306](https://www.english-corpora.org/corona/x4.asp?rs=&t=43190068&ID=20934330213) | [21-07-08 US](https://www.english-corpora.org/corona/x4.asp?rs=&t=43190068&ID=20934330213) | [khou.com](https://www.english-corpora.org/corona/x3.asp?node=&p=14&w10=coronavirus&w11=vaccination&r=) |  |  |  | among Triad counties giving vaccine incentives # As **coronavirus** **vaccination** rates drop in the Triad, local health departments are getting creative about how to |
| [1307](https://www.english-corpora.org/corona/x4.asp?rs=&t=52921521&ID=20955644258) | [21-07-10 US](https://www.english-corpora.org/corona/x4.asp?rs=&t=52921521&ID=20955644258) | [pix11.com](https://www.english-corpora.org/corona/x3.asp?node=&p=14&w10=coronavirus&w11=vaccination&r=) |  |  |  | (AP) -- The Mediterranean island nation of Malta is requiring proof of **coronavirus** **vaccination** for visitors 13 and up, the first European Union nation to do so, |
| [1308](https://www.english-corpora.org/corona/x4.asp?rs=&t=52931186&ID=20990060376) | [21-07-13 US](https://www.english-corpora.org/corona/x4.asp?rs=&t=52931186&ID=20990060376) | [bangordailynews.com](https://www.english-corpora.org/corona/x3.asp?node=&p=14&w10=coronavirus&w11=vaccination&r=) |  |  |  | Maine -- Maine health officials said the state is going to continue its pop-up **coronavirus** **vaccination** clinics through the summer. # Maine has one of the highest COVID-19 vaccination rates |
| [1309](https://www.english-corpora.org/corona/x4.asp?rs=&t=63097660&ID=20900169749) | [21-07-05 US](https://www.english-corpora.org/corona/x4.asp?rs=&t=63097660&ID=20900169749) | [axios.com](https://www.english-corpora.org/corona/x3.asp?node=&p=14&w10=coronavirus&w11=vaccination&r=) |  |  |  | registered with the national health service and may lack access to the country's **coronavirus** **vaccination** campaign. # President Biden during a Fourth of July BBQ event to celebrate Independence |
| [1310](https://www.english-corpora.org/corona/x4.asp?rs=&t=73194623&ID=20872027100) | [21-07-02 US](https://www.english-corpora.org/corona/x4.asp?rs=&t=73194623&ID=20872027100) | [forward.com](https://www.english-corpora.org/corona/x3.asp?node=&p=14&w10=coronavirus&w11=vaccination&r=) |  |  |  | symbol of the Holocaust, during a recent speech decrying the state government's **coronavirus** **vaccination** campaign as well as restrictions that have been in place since the pandemic began. |
| [1311](https://www.english-corpora.org/corona/x4.asp?rs=&t=73204781&ID=20902702970) | [21-07-05 US](https://www.english-corpora.org/corona/x4.asp?rs=&t=73204781&ID=20902702970) | [theweek.com](https://www.english-corpora.org/corona/x3.asp?node=&p=14&w10=coronavirus&w11=vaccination&r=) |  |  |  | Sunday that he would continue wearing a mask in any place that has low **coronavirus** **vaccination** rates. Fauci told Chuck Todd, anchor of NBC's Meet the Press, |
| [1312](https://www.english-corpora.org/corona/x4.asp?rs=&t=73235782&ID=21002525329) | [21-07-14 US](https://www.english-corpora.org/corona/x4.asp?rs=&t=73235782&ID=21002525329) | [pe.com](https://www.english-corpora.org/corona/x3.asp?node=&p=14&w10=coronavirus&w11=vaccination&r=) |  |  |  | 73235782 # While the weekly number of Riverside County **coronavirus** **vaccination** doses continues to fall, the county has topped 1 million residents who are fully |
| [1313](https://www.english-corpora.org/corona/x4.asp?rs=&t=87504771&ID=20868194480) | [21-07-01 US](https://www.english-corpora.org/corona/x4.asp?rs=&t=87504771&ID=20868194480) | [YAHOO!News](https://www.english-corpora.org/corona/x3.asp?node=&p=14&w10=coronavirus&w11=vaccination&r=) |  |  |  | the Indonesian city of Bekasi on the outskirts of Jakarta are holding a mass **coronavirus** **vaccination** for 25,000 people at the city's main stadium Thursday. Officials say more than |
| [1314](https://www.english-corpora.org/corona/x4.asp?rs=&t=87505771&ID=20868232714) | [21-07-01 US](https://www.english-corpora.org/corona/x4.asp?rs=&t=87505771&ID=20868232714) | [MarketWatch](https://www.english-corpora.org/corona/x3.asp?node=&p=14&w10=coronavirus&w11=vaccination&r=) |  |  |  | recuperate from side effects, while two in three say their employer has encouraged **coronavirus** **vaccination**, according to the latest polling from KFF, a healthcare think tank. # |
| [1315](https://www.english-corpora.org/corona/x4.asp?rs=&t=87505771&ID=20868233242) | [21-07-01 US](https://www.english-corpora.org/corona/x4.asp?rs=&t=87505771&ID=20868233242) | [MarketWatch](https://www.english-corpora.org/corona/x3.asp?node=&p=14&w10=coronavirus&w11=vaccination&r=) |  |  |  | Experts told MarketWatch this month that colleges were likely to lead the way on **coronavirus** **vaccination** mandates in educational settings once the vaccine gained full FDA approval, and more companies |
| [1316](https://www.english-corpora.org/corona/x4.asp?rs=&t=87509622&ID=20865784779) | [21-07-01 US](https://www.english-corpora.org/corona/x4.asp?rs=&t=87509622&ID=20865784779) | [MarketWatch](https://www.english-corpora.org/corona/x3.asp?node=&p=14&w10=coronavirus&w11=vaccination&r=) |  |  |  | of Holon near Tel Aviv on June 21, 2021, as Israel begins **coronavirus** **vaccination** campaign for 12 to 15-year-olds. # jack guez/Agence France-Presse/Getty Images # Email icon # |
| [1317](https://www.english-corpora.org/corona/x4.asp?rs=&t=87515121&ID=20877519615) | [21-07-02 US](https://www.english-corpora.org/corona/x4.asp?rs=&t=87515121&ID=20877519615) | [Fox News](https://www.english-corpora.org/corona/x3.asp?node=&p=14&w10=coronavirus&w11=vaccination&r=) |  |  |  | mocked earlier in June for publishing a report that boosted reports on China's **coronavirus** **vaccination** rates, without appearing to question China's too-good-to-be-true statistics. # Stilwell agrees that |
| [1318](https://www.english-corpora.org/corona/x4.asp?rs=&t=87522972&ID=20896104112) | [21-07-04 US](https://www.english-corpora.org/corona/x4.asp?rs=&t=87522972&ID=20896104112) | [New York Times](https://www.english-corpora.org/corona/x3.asp?node=&p=14&w10=coronavirus&w11=vaccination&r=) |  |  |  | a social security number, she has had no access to the country's **coronavirus** **vaccination** campaign. # But over the weekend, she was one of nearly 900 people |
| [1319](https://www.english-corpora.org/corona/x4.asp?rs=&t=87525358&ID=20895825694) | [21-07-04 US](https://www.english-corpora.org/corona/x4.asp?rs=&t=87525358&ID=20895825694) | [New York Times](https://www.english-corpora.org/corona/x3.asp?node=&p=14&w10=coronavirus&w11=vaccination&r=) |  |  |  | Credit... Amanda Lucier for The New York Times # When it came to **coronavirus** **vaccination**, the third time was the charm for Esther Jones, a dialysis nurse in |
| [1322](https://www.english-corpora.org/corona/x4.asp?rs=&t=87526924&ID=20903357762) | [21-07-05 US](https://www.english-corpora.org/corona/x4.asp?rs=&t=87526924&ID=20903357762) | [The Motley Fool](https://www.english-corpora.org/corona/x3.asp?node=&p=14&w10=coronavirus&w11=vaccination&r=) [(2)](https://www.english-corpora.org/corona/duplicates1.asp?xx=1321&n=2) |  |  |  | as her 9-year-old daughter. # As more and more people opt for **coronavirus** **vaccination**, we expect fewer cases of the illness. And that means less need for |
| [1323](https://www.english-corpora.org/corona/x4.asp?rs=&t=87528422&ID=20903150119) | [21-07-05 US](https://www.english-corpora.org/corona/x4.asp?rs=&t=87528422&ID=20903150119) | [YAHOO!News](https://www.english-corpora.org/corona/x3.asp?node=&p=14&w10=coronavirus&w11=vaccination&r=) |  |  |  | who would do anything to get vaccines. " # When it came to **coronavirus** **vaccination**, the third time was the charm for Esther Jones, a dialysis nurse in |
| [1325](https://www.english-corpora.org/corona/x4.asp?rs=&t=87531865&ID=20911930803) | [21-07-06 US](https://www.english-corpora.org/corona/x4.asp?rs=&t=87531865&ID=20911930803) | [The Baltimore Sun](https://www.english-corpora.org/corona/x3.asp?node=&p=14&w10=coronavirus&w11=vaccination&r=) [(1)](https://www.english-corpora.org/corona/duplicates1.asp?xx=1324&n=1) |  |  |  | in Baltimore, which has lagged behind much of the state increasing its **coronavirus** **vaccination** rate, city officials announced just last week that they'll begin restoring public-facing services |
| [1326](https://www.english-corpora.org/corona/x4.asp?rs=&t=87531932&ID=20910241479) | [21-07-06 US](https://www.english-corpora.org/corona/x4.asp?rs=&t=87531932&ID=20910241479) | [The Boston Globe](https://www.english-corpora.org/corona/x3.asp?node=&p=14&w10=coronavirus&w11=vaccination&r=) |  |  |  | so that individuals get vaccinated. # A person gets vaccinated at a drive-thru **coronavirus** **vaccination** site in Columbus, Ohio, on May 13, four days before before the |
| [1327](https://www.english-corpora.org/corona/x4.asp?rs=&t=87540088&ID=20924943879) | [21-07-07 US](https://www.english-corpora.org/corona/x4.asp?rs=&t=87540088&ID=20924943879) | [Reuters on MSN.com](https://www.english-corpora.org/corona/x3.asp?node=&p=14&w10=coronavirus&w11=vaccination&r=) |  |  |  | they had detained a health worker in the Kaliningrad region for allegedly selling fake **coronavirus** **vaccination** certificates. # Moscow and several Russian regions have resorted to tough measures to encourage |
| [1328](https://www.english-corpora.org/corona/x4.asp?rs=&t=87542666&ID=20923703575) | [21-07-07 US](https://www.english-corpora.org/corona/x4.asp?rs=&t=87542666&ID=20923703575) | [The Washington Post on MSN.com](https://www.english-corpora.org/corona/x3.asp?node=&p=14&w10=coronavirus&w11=vaccination&r=) |  |  |  | wave of infections starting in May has not receded. # Turkmenistan is making **coronavirus** **vaccination** mandatory for all adults, Reuters reported Wednesday, citing the Central Asian nation's |
| [1329](https://www.english-corpora.org/corona/x4.asp?rs=&t=87543394&ID=20924508264) | [21-07-07 US](https://www.english-corpora.org/corona/x4.asp?rs=&t=87543394&ID=20924508264) | [New York Times](https://www.english-corpora.org/corona/x3.asp?node=&p=14&w10=coronavirus&w11=vaccination&r=) |  |  |  | photo by Henning Schacht # When Chancellor Angela Merkel of Germany received her second **coronavirus** **vaccination** recently, she was given a Moderna shot to follow the AstraZeneca one she received |
| [1330](https://www.english-corpora.org/corona/x4.asp?rs=&t=87543680&ID=20939707197) | [21-07-08 US](https://www.english-corpora.org/corona/x4.asp?rs=&t=87543680&ID=20939707197) | [YAHOO!News](https://www.english-corpora.org/corona/x3.asp?node=&p=14&w10=coronavirus&w11=vaccination&r=) |  |  |  | $720-billion economy avoid a contraction and mount a strong recovery helped by a speedy **coronavirus** **vaccination** campaign. # Now, authorities want the lenders to be ready for another burst |
| [1331](https://www.english-corpora.org/corona/x4.asp?rs=&t=87546723&ID=20937206539) | [21-07-08 US](https://www.english-corpora.org/corona/x4.asp?rs=&t=87546723&ID=20937206539) | [Chicago Tribune](https://www.english-corpora.org/corona/x3.asp?node=&p=14&w10=coronavirus&w11=vaccination&r=) |  |  |  | names were drawn from a database of residents who had received at least one **coronavirus** **vaccination** by July 1, were to be notified immediately by phone and email. Their |
| [1332](https://www.english-corpora.org/corona/x4.asp?rs=&t=87555349&ID=20952479657) | [21-07-09 US](https://www.english-corpora.org/corona/x4.asp?rs=&t=87555349&ID=20952479657) | [New York Times](https://www.english-corpora.org/corona/x3.asp?node=&p=14&w10=coronavirus&w11=vaccination&r=) |  |  |  | Although those in attendance at Wembley were required to provide proof of a full **coronavirus** **vaccination** or a negative test result taken 48 hours before the game, few people wore |
| [1333](https://www.english-corpora.org/corona/x4.asp?rs=&t=87557689&ID=20965264402) | [21-07-10 US](https://www.english-corpora.org/corona/x4.asp?rs=&t=87557689&ID=20965264402) | [The Hill](https://www.english-corpora.org/corona/x3.asp?node=&p=14&w10=coronavirus&w11=vaccination&r=) |  |  |  | announcement this week that it was seeking authorization for a third dose of its **coronavirus** **vaccination**. # Pfizer and six people familiar with the plans confirmed to the newspaper that |
| [1334](https://www.english-corpora.org/corona/x4.asp?rs=&t=87558013&ID=20962740057) | [21-07-10 US](https://www.english-corpora.org/corona/x4.asp?rs=&t=87558013&ID=20962740057) | [New York Post](https://www.english-corpora.org/corona/x3.asp?node=&p=14&w10=coronavirus&w11=vaccination&r=) |  |  |  | # During the 20 minute interview, Harris said she's working to improve **coronavirus** **vaccination** rates among black people, less than a quarter of whom are fully vaccinated. |
| [1335](https://www.english-corpora.org/corona/x4.asp?rs=&t=87559977&ID=20965137747) | [21-07-10 US](https://www.english-corpora.org/corona/x4.asp?rs=&t=87559977&ID=20965137747) | [The Independent on MSN.com](https://www.english-corpora.org/corona/x3.asp?node=&p=14&w10=coronavirus&w11=vaccination&r=) |  |  |  | two of the measures being used by companies to encourage employees to have the **coronavirus** **vaccination**. # Amazon, SkyMolson Coors and Three UK are the latest firms to join |
| [1336](https://www.english-corpora.org/corona/x4.asp?rs=&t=87564550&ID=20970069130) | [21-07-11 US](https://www.english-corpora.org/corona/x4.asp?rs=&t=87564550&ID=20970069130) | [Washington Post](https://www.english-corpora.org/corona/x3.asp?node=&p=14&w10=coronavirus&w11=vaccination&r=) |  |  |  | won a year's worth of Metro rides as part of the city's **coronavirus** **vaccination** lottery. (Amanda Voisard/The Washington Post) # For Sung-ha Jou, a 44-year-old |
| [1337](https://www.english-corpora.org/corona/x4.asp?rs=&t=87567712&ID=20980438681) | [21-07-12 US](https://www.english-corpora.org/corona/x4.asp?rs=&t=87567712&ID=20980438681) | [The Independent on MSN.com](https://www.english-corpora.org/corona/x3.asp?node=&p=14&w10=coronavirus&w11=vaccination&r=) |  |  |  | Andy Gregory # A new advertisement by the Australian government aimed at boosting the **coronavirus** **vaccination** rate in the country has created a stir, reports Namita Singh. # The |
| [1338](https://www.english-corpora.org/corona/x4.asp?rs=&t=87568139&ID=20982657484) | [21-07-12 US](https://www.english-corpora.org/corona/x4.asp?rs=&t=87568139&ID=20982657484) | [Patch](https://www.english-corpora.org/corona/x3.asp?node=&p=14&w10=coronavirus&w11=vaccination&r=) |  |  |  | been damaged or lost. " # Vaccine passports refer to enforced proof of **coronavirus** **vaccination** -- a controversial subject in the discussion of health privacy. New Jersey does not |
| [1339](https://www.english-corpora.org/corona/x4.asp?rs=&t=87568200&ID=20980129330) | [21-07-12 US](https://www.english-corpora.org/corona/x4.asp?rs=&t=87568200&ID=20980129330) | [CNN on MSN.com](https://www.english-corpora.org/corona/x3.asp?node=&p=14&w10=coronavirus&w11=vaccination&r=) |  |  |  | in Australia, with social media users criticizing its targeting of young people for **coronavirus** **vaccination** -- the majority of whom are not yet eligible to receive the recommended shot. |
| [1340](https://www.english-corpora.org/corona/x4.asp?rs=&t=87577276&ID=20995278171) | [21-07-13 US](https://www.english-corpora.org/corona/x4.asp?rs=&t=87577276&ID=20995278171) | [Yahoo](https://www.english-corpora.org/corona/x3.asp?node=&p=14&w10=coronavirus&w11=vaccination&r=) |  |  |  | pressure from Republican state lawmakers. # The health department will also stop holding **coronavirus** **vaccination** events at schools and sending postcards with reminders to teenagers to get a second COVID |
| [1341](https://www.english-corpora.org/corona/x4.asp?rs=&t=87580173&ID=21005978228) | [21-07-14 US](https://www.english-corpora.org/corona/x4.asp?rs=&t=87580173&ID=21005978228) | [Patch](https://www.english-corpora.org/corona/x3.asp?node=&p=14&w10=coronavirus&w11=vaccination&r=) |  |  |  | With Biden Rodrigo, 18, was in Washington D.C. Wednesday to encourage **coronavirus** **vaccination** among youth. Posted Wed, Jul 14, 2021 at 12:52 pm PT |
| [1342](https://www.english-corpora.org/corona/x4.asp?rs=&t=87580173&ID=21005978343) | [21-07-14 US](https://www.english-corpora.org/corona/x4.asp?rs=&t=87580173&ID=21005978343) | [Patch](https://www.english-corpora.org/corona/x3.asp?node=&p=14&w10=coronavirus&w11=vaccination&r=) |  |  |  | the help Wednesday of an 18-year-old pop phenom from Southwest Riverside County to encourage **coronavirus** **vaccination** among young people. # Olivia Rodrigo -- who was born in Temecula and went |
| [1343](https://www.english-corpora.org/corona/x4.asp?rs=&t=87594219&ID=21029593601) | [21-07-16 US](https://www.english-corpora.org/corona/x4.asp?rs=&t=87594219&ID=21029593601) | [CBS Baltimore](https://www.english-corpora.org/corona/x3.asp?node=&p=14&w10=coronavirus&w11=vaccination&r=) |  |  |  | at Lakefront at Columbia on the Fourth of July, you can get a **coronavirus** **vaccination** while you're there. # Care 4 Your Health will be at the Lakefront |
| [1345](https://www.english-corpora.org/corona/x4.asp?rs=&t=87596689&ID=21032889502) | [21-07-16 US](https://www.english-corpora.org/corona/x4.asp?rs=&t=87596689&ID=21032889502) | [Washington Examiner](https://www.english-corpora.org/corona/x3.asp?node=&p=14&w10=coronavirus&w11=vaccination&r=) [(1)](https://www.english-corpora.org/corona/duplicates1.asp?xx=1344&n=1) |  |  |  | Boston University School of Medicine found that Ohio's lottery did not increase **coronavirus** **vaccination** rates when compared with other states that did not offer a lottery incentive. The |
| [1346](https://www.english-corpora.org/corona/x4.asp?rs=&t=52926729&ID=20973277763) | [21-07-12 CA](https://www.english-corpora.org/corona/x4.asp?rs=&t=52926729&ID=20973277763) | [princegeorgecitizen.com](https://www.english-corpora.org/corona/x3.asp?node=&p=14&w10=coronavirus&w11=vaccination&r=) |  |  |  | workers will be suspended if they refuse to get vaccinated. Italy made the **coronavirus** **vaccination** obligatory for health care workers and pharmacists, and those who opt out risk suspension |
| [1347](https://www.english-corpora.org/corona/x4.asp?rs=&t=52933306&ID=20996867979) | [21-07-14 CA](https://www.english-corpora.org/corona/x4.asp?rs=&t=52933306&ID=20996867979) | [chroniclejournal.com](https://www.english-corpora.org/corona/x3.asp?node=&p=14&w10=coronavirus&w11=vaccination&r=) |  |  |  | . # Meanwhile, Malta has eased off a new rule requiring proof of **coronavirus** **vaccination** to enter the country after the European Commission raised concerns that it might impede the |
| [1349](https://www.english-corpora.org/corona/x4.asp?rs=&t=33009358&ID=20929609074) | [21-07-08 GB](https://www.english-corpora.org/corona/x4.asp?rs=&t=33009358&ID=20929609074) | [portsmouth.co.uk](https://www.english-corpora.org/corona/x3.asp?node=&p=14&w10=coronavirus&w11=vaccination&r=) [(1)](https://www.english-corpora.org/corona/duplicates1.asp?xx=1348&n=1) |  |  |  | this week, passengers from selected destinations will be able to upload their **coronavirus** **vaccination** certificate before boarding. How will it work? Sign up to our |
| [1350](https://www.english-corpora.org/corona/x4.asp?rs=&t=43176609&ID=20881780723) | [21-07-03 GB](https://www.english-corpora.org/corona/x4.asp?rs=&t=43176609&ID=20881780723) | [politico.eu](https://www.english-corpora.org/corona/x3.asp?node=&p=14&w10=coronavirus&w11=vaccination&r=) |  |  |  | Some 300 health care workers in northern Italy lodged a legal challenge against mandatory **coronavirus** **vaccination**, according to Italian media. " We force people to take a risk under |
| [1351](https://www.english-corpora.org/corona/x4.asp?rs=&t=43176917&ID=20881808172) | [21-07-03 GB](https://www.english-corpora.org/corona/x4.asp?rs=&t=43176917&ID=20881808172) | [expressandstar.com](https://www.english-corpora.org/corona/x3.asp?node=&p=14&w10=coronavirus&w11=vaccination&r=) |  |  |  | half-hearted, such as limiting restaurant opening hours on weekend nights. # A **coronavirus** **vaccination** centre at an exhibition in Moscow (AP) # In Moscow, however, |
| [1352](https://www.english-corpora.org/corona/x4.asp?rs=&t=43177435&ID=20883513173) | [21-07-03 GB](https://www.english-corpora.org/corona/x4.asp?rs=&t=43177435&ID=20883513173) | [grimsbytelegraph.co.uk](https://www.english-corpora.org/corona/x3.asp?node=&p=14&w10=coronavirus&w11=vaccination&r=) |  |  |  | offered the **coronavirus** **vaccination**, more than half of parents would allow their children to get it, according |
| [1353](https://www.english-corpora.org/corona/x4.asp?rs=&t=43180421&ID=20897011121) | [21-07-05 GB](https://www.english-corpora.org/corona/x4.asp?rs=&t=43180421&ID=20897011121) | [politico.eu](https://www.english-corpora.org/corona/x3.asp?node=&p=14&w10=coronavirus&w11=vaccination&r=) |  |  |  | : A group of health professionals launched a petition asking the government to make **coronavirus** **vaccination** obligatory for " any person who, in a public or private prevention or care |
| [1354](https://www.english-corpora.org/corona/x4.asp?rs=&t=43181161&ID=20899083517) | [21-07-05 GB](https://www.english-corpora.org/corona/x4.asp?rs=&t=43181161&ID=20899083517) | [shropshirestar.com](https://www.english-corpora.org/corona/x3.asp?node=&p=14&w10=coronavirus&w11=vaccination&r=) |  |  |  | " get the jab, grab a poke " initiative for those receiving the **coronavirus** **vaccination** at a centre in Belfast was commended on Monday. # The South Eastern Health |
| [1355](https://www.english-corpora.org/corona/x4.asp?rs=&t=43186100&ID=20926140323) | [21-07-08 GB](https://www.english-corpora.org/corona/x4.asp?rs=&t=43186100&ID=20926140323) | [dailyrecord.co.uk](https://www.english-corpora.org/corona/x3.asp?node=&p=14&w10=coronavirus&w11=vaccination&r=) |  |  |  | per cent of the adult population -- have received their first dose of a **coronavirus** **vaccination** and 347,867 of them -- 65.7 per cent of the adult population -- have received |
| [1356](https://www.english-corpora.org/corona/x4.asp?rs=&t=43193996&ID=20947760799) | [21-07-09 GB](https://www.english-corpora.org/corona/x4.asp?rs=&t=43193996&ID=20947760799) | [shropshirestar.com](https://www.english-corpora.org/corona/x3.asp?node=&p=14&w10=coronavirus&w11=vaccination&r=) |  |  |  | launched this week, passengers from selected destinations will be able to upload their **coronavirus** **vaccination** certificate before boarding. # On arrival at the airport, they will then be |
| [1357](https://www.english-corpora.org/corona/x4.asp?rs=&t=43198292&ID=20960191552) | [21-07-10 GB](https://www.english-corpora.org/corona/x4.asp?rs=&t=43198292&ID=20960191552) | [politico.eu](https://www.english-corpora.org/corona/x3.asp?node=&p=14&w10=coronavirus&w11=vaccination&r=) |  |  |  | end of March. # NHS STAFF FACING ABUSE: NHS staff working in **coronavirus** **vaccination** centers have reported abuse, threats and aggression from people wanting the get their second |
| [1358](https://www.english-corpora.org/corona/x4.asp?rs=&t=43198356&ID=20957426673) | [21-07-10 GB](https://www.english-corpora.org/corona/x4.asp?rs=&t=43198356&ID=20957426673) | [chroniclelive.co.uk](https://www.english-corpora.org/corona/x3.asp?node=&p=14&w10=coronavirus&w11=vaccination&r=) |  |  |  | two of the measures being used by companies to encourage employees to have the **coronavirus** **vaccination**. # Amazon, Sky, Molson Coors and Three UK are the latest firms |
| [1362](https://www.english-corpora.org/corona/x4.asp?rs=&t=43199245&ID=20957523840) | [21-07-10 GB](https://www.english-corpora.org/corona/x4.asp?rs=&t=43199245&ID=20957523840) | [hulldailymail.co.uk](https://www.english-corpora.org/corona/x3.asp?node=&p=14&w10=coronavirus&w11=vaccination&r=) [(3)](https://www.english-corpora.org/corona/duplicates1.asp?xx=1361&n=3) |  |  |  | this week, passengers from selected destinations will be able to upload their **coronavirus** **vaccination** certificate before boarding. # On arrival at the airport, they will then be |
| [1363](https://www.english-corpora.org/corona/x4.asp?rs=&t=43199620&ID=20954730252) | [21-07-10 GB](https://www.english-corpora.org/corona/x4.asp?rs=&t=43199620&ID=20954730252) | [derbytelegraph.co.uk](https://www.english-corpora.org/corona/x3.asp?node=&p=14&w10=coronavirus&w11=vaccination&r=) |  |  |  | two of the measures being used by companies to encourage employees to have the **coronavirus** **vaccination**. # Amazon, Sky, Molson Coors and Three UK are the latest firms |
| [1368](https://www.english-corpora.org/corona/x4.asp?rs=&t=43222138&ID=21023601468) | [21-07-16 GB](https://www.english-corpora.org/corona/x4.asp?rs=&t=43222138&ID=21023601468) | [poundsterlinglive.com](https://www.english-corpora.org/corona/x3.asp?node=&p=14&w10=coronavirus&w11=vaccination&r=) [(4)](https://www.english-corpora.org/corona/duplicates1.asp?xx=1367&n=4) |  |  |  | may draw support in the coming week from an acceleration of Britain's **coronavirus** **vaccination** programme, although international factors and Bank of England (BoE) monetary policy may |
| [1369](https://www.english-corpora.org/corona/x4.asp?rs=&t=63139282&ID=21027599376) | [21-07-16 GB](https://www.english-corpora.org/corona/x4.asp?rs=&t=63139282&ID=21027599376) | [dailypost.co.uk](https://www.english-corpora.org/corona/x3.asp?node=&p=14&w10=coronavirus&w11=vaccination&r=) |  |  |  | treatment. All three are understood to have already received both doses of a **coronavirus** **vaccination**. It is unclear if or how many of the patients had underlying health conditions |
| [1370](https://www.english-corpora.org/corona/x4.asp?rs=&t=73204638&ID=20900778710) | [21-07-05 GB](https://www.english-corpora.org/corona/x4.asp?rs=&t=73204638&ID=20900778710) | [news.stv.tv](https://www.english-corpora.org/corona/x3.asp?node=&p=14&w10=coronavirus&w11=vaccination&r=) |  |  |  | Scotland, all health boards in the country's mainland are now offering drop-in **coronavirus** **vaccination** clinics. # From Monday, anyone aged 18 and over can attend for their |
| [1371](https://www.english-corpora.org/corona/x4.asp?rs=&t=87574934&ID=20993362705) | [21-07-13 GB](https://www.english-corpora.org/corona/x4.asp?rs=&t=87574934&ID=20993362705) | [The Sun](https://www.english-corpora.org/corona/x3.asp?node=&p=14&w10=coronavirus&w11=vaccination&r=) |  |  |  | and medicine shortages in the coming weeks, as well as disruption to the **coronavirus** **vaccination** drive. # A statement from the military said " pre-deployment processes had started " |
| [1372](https://www.english-corpora.org/corona/x4.asp?rs=&t=63093858&ID=20899727976) | [21-07-05 IE](https://www.english-corpora.org/corona/x4.asp?rs=&t=63093858&ID=20899727976) | [trtworld.com](https://www.english-corpora.org/corona/x3.asp?node=&p=14&w10=coronavirus&w11=vaccination&r=) |  |  |  | Russia launches booster shots amid soaring infections # Russian health authorities have launched booster **coronavirus** **vaccination** for those who had been immunised more than six months ago, as the country |
| [1373](https://www.english-corpora.org/corona/x4.asp?rs=&t=63097198&ID=20902010173) | [21-07-05 IE](https://www.english-corpora.org/corona/x4.asp?rs=&t=63097198&ID=20902010173) | [echolive.ie](https://www.english-corpora.org/corona/x3.asp?node=&p=14&w10=coronavirus&w11=vaccination&r=) |  |  |  | Health Minister Stephen Donnelly confirmed that people aged 18 to 34 can receive their **coronavirus** **vaccination** up to two months early under the accelerated programme. # It follows updated advice |
| [1374](https://www.english-corpora.org/corona/x4.asp?rs=&t=73215240&ID=20946134674) | [21-07-09 AU](https://www.english-corpora.org/corona/x4.asp?rs=&t=73215240&ID=20946134674) | [news.com.au](https://www.english-corpora.org/corona/x3.asp?node=&p=14&w10=coronavirus&w11=vaccination&r=) |  |  |  | for other sectors discussed # The federal government is finalising its decision to make **coronavirus** **vaccination** mandatory for all aged care workers across Australia. # National Cabinet also discussed papers |
| [1375](https://www.english-corpora.org/corona/x4.asp?rs=&t=73241055&ID=21012231201) | [21-07-15 IN](https://www.english-corpora.org/corona/x4.asp?rs=&t=73241055&ID=21012231201) | [bloombergquint.com](https://www.english-corpora.org/corona/x3.asp?node=&p=14&w10=coronavirus&w11=vaccination&r=) |  |  |  | Rate Spikes as Deadline Approaches # (Bloomberg) -- Saudi Arabia's daily **coronavirus** **vaccination** rate has accelerated to more than 400,000 ahead of a deadline that would ban those |
| [1377](https://www.english-corpora.org/corona/x4.asp?rs=&t=43222356&ID=21023630684) | [21-07-16 PK](https://www.english-corpora.org/corona/x4.asp?rs=&t=43222356&ID=21023630684) | [paktribune.com](https://www.english-corpora.org/corona/x3.asp?node=&p=14&w10=coronavirus&w11=vaccination&r=) [(1)](https://www.english-corpora.org/corona/duplicates1.asp?xx=1376&n=1) |  |  |  | new guidelines for travellers and made it mandatory for them to carry a **coronavirus** **vaccination** certificate issued by the National Database and Registration Authority (Nadra) and attested by |
| [1378](https://www.english-corpora.org/corona/x4.asp?rs=&t=52920292&ID=20961107891) | [21-07-10 PK](https://www.english-corpora.org/corona/x4.asp?rs=&t=52920292&ID=20961107891) | [nation.com.pk](https://www.english-corpora.org/corona/x3.asp?node=&p=14&w10=coronavirus&w11=vaccination&r=) |  |  |  | implementation of the SOPs. He also directed the provinces to ramp up their **coronavirus** **vaccination** drives. # The NCOC also issued special instructions for the occasion of Eidul Azha |
| [1379](https://www.english-corpora.org/corona/x4.asp?rs=&t=52925036&ID=20967183123) | [21-07-11 PK](https://www.english-corpora.org/corona/x4.asp?rs=&t=52925036&ID=20967183123) | [tribune.com.pk](https://www.english-corpora.org/corona/x3.asp?node=&p=14&w10=coronavirus&w11=vaccination&r=) |  |  |  | regard to the vaccination process. # The province's top bureaucrat said the **coronavirus** **vaccination** campaign was a national cause and negligence in this regard would not be tolerated. |
| [1380](https://www.english-corpora.org/corona/x4.asp?rs=&t=73239933&ID=21009562359) | [21-07-15 MY](https://www.english-corpora.org/corona/x4.asp?rs=&t=73239933&ID=21009562359) | [khmertimeskh.com](https://www.english-corpora.org/corona/x3.asp?node=&p=14&w10=coronavirus&w11=vaccination&r=) |  |  |  | 333 voting in favour and 298 voting against the proposal. # India's **coronavirus** **vaccination** drive is hobbling once again. In the seven days till Sunday, the number |
| [1392](https://www.english-corpora.org/corona/x4.asp?rs=&t=43210187&ID=20989522463) | [21-07-13 SG](https://www.english-corpora.org/corona/x4.asp?rs=&t=43210187&ID=20989522463) | [theonlinecitizen.com](https://www.english-corpora.org/corona/x3.asp?node=&p=14&w10=coronavirus&w11=vaccination&r=) [(11)](https://www.english-corpora.org/corona/duplicates1.asp?xx=1391&n=11) |  |  |  | after 200 workers infected # A Malaysian **coronavirus** **vaccination** centre was shut down Tuesday after more than 200 workers tested positive for COVID-19, |
| [1393](https://www.english-corpora.org/corona/x4.asp?rs=&t=73204562&ID=20900774479) | [21-07-05 PH](https://www.english-corpora.org/corona/x4.asp?rs=&t=73204562&ID=20900774479) | [bworldonline.com](https://www.english-corpora.org/corona/x3.asp?node=&p=14&w10=coronavirus&w11=vaccination&r=) |  |  |  | said intrazonal travel for fully vaccinated individuals is subject to the presentation of a **coronavirus** **vaccination** card issued by a legitimate establishment, or a certificate of quarantine completion showing the |
| [1394](https://www.english-corpora.org/corona/x4.asp?rs=&t=43209321&ID=20984874738) | [21-07-13 HK](https://www.english-corpora.org/corona/x4.asp?rs=&t=43209321&ID=20984874738) | [asiatimes.com](https://www.english-corpora.org/corona/x3.asp?node=&p=14&w10=coronavirus&w11=vaccination&r=) |  |  |  | . Photo: AFP / Farid Bin Tajuddin / Anadolu Agency # A Malaysian **coronavirus** **vaccination** center was shut down Tuesday after more than 200 workers tested positive for Covid-19, |
| [1395](https://www.english-corpora.org/corona/x4.asp?rs=&t=63125703&ID=20985902956) | [21-07-13 ZA](https://www.english-corpora.org/corona/x4.asp?rs=&t=63125703&ID=20985902956) | [moneyweb.co.za](https://www.english-corpora.org/corona/x3.asp?node=&p=14&w10=coronavirus&w11=vaccination&r=) |  |  |  | KwaZulu-Natal on Tuesday, and violence prompted the provincial health department to halt its **coronavirus** **vaccination** program. Stores were also targeted in East London in the Eastern Cape province, |
| [1396](https://www.english-corpora.org/corona/x4.asp?rs=&t=52915111&ID=20927410341) | [21-07-08 GH](https://www.english-corpora.org/corona/x4.asp?rs=&t=52915111&ID=20927410341) | [newsghana.com.gh](https://www.english-corpora.org/corona/x3.asp?node=&p=14&w10=coronavirus&w11=vaccination&r=) |  |  |  | , the United States has, as expected, narrowly fallen short of a **coronavirus** **vaccination** target set by President Joe Biden. # Biden had wanted 70 per cent of |
| [1397](https://www.english-corpora.org/corona/x4.asp?rs=&t=63105867&ID=20935471360) | [21-07-08 KE](https://www.english-corpora.org/corona/x4.asp?rs=&t=63105867&ID=20935471360) | [citizentv.co.ke](https://www.english-corpora.org/corona/x3.asp?node=&p=14&w10=coronavirus&w11=vaccination&r=) |  |  |  | Biden's goal of 70% of the country's adults having at least one **coronavirus** **vaccination** in time for the holiday, but it came close at 67.1%. # Health |
| [1398](https://www.english-corpora.org/corona/x4.asp?rs=&t=63106559&ID=20931774527) | [21-07-08 TZ](https://www.english-corpora.org/corona/x4.asp?rs=&t=63106559&ID=20931774527) | [ippmedia.com](https://www.english-corpora.org/corona/x3.asp?node=&p=14&w10=coronavirus&w11=vaccination&r=) |  |  |  | delegation to the Tokyo Olympics will not travel to either Rwanda or Qatar for **Coronavirus** **vaccination** as directed by the IOC. # The IOC directed that athletes travelling to Japan |
| [1399](https://www.english-corpora.org/corona/x4.asp?rs=&t=63106559&ID=20931774573) | [21-07-08 TZ](https://www.english-corpora.org/corona/x4.asp?rs=&t=63106559&ID=20931774573) | [ippmedia.com](https://www.english-corpora.org/corona/x3.asp?node=&p=14&w10=coronavirus&w11=vaccination&r=) |  |  |  | . # " We will travel straight to Japan for the Games, the **Coronavirus** **vaccination** directed by IOC in Rwanda and Qatar is not mandatory, it is voluntary. |
| [1400](https://www.english-corpora.org/corona/x4.asp?rs=&t=87600737&ID=21031226588) | [21-07-16 --](https://www.english-corpora.org/corona/x4.asp?rs=&t=87600737&ID=21031226588) | [Mass](https://www.english-corpora.org/corona/x3.asp?node=&p=14&w10=coronavirus&w11=vaccination&r=) |  |  |  | of serving AAA members who make appointments. For details about the Commonwealth's **coronavirus** **vaccination** locations and other information, please visit: ERROR? |

PAGE:   [**<<**](https://www.english-corpora.org/corona/x3.asp?node=&p=1&w10=coronavirus&w11=vaccination&r=)  [**<**](https://www.english-corpora.org/corona/x3.asp?node=&p=13&w10=coronavirus&w11=vaccination&r=)   14 / 42  [**>**](https://www.english-corpora.org/corona/x3.asp?node=&p=15&w10=coronavirus&w11=vaccination&r=)   [**>>**](https://www.english-corpora.org/corona/x3.asp?node=&p=42&w10=coronavirus&w11=vaccination&r=)

Конец формы

| PAGE:   [**<<**](https://www.english-corpora.org/corona/x3.asp?node=&p=1&w10=coronavirus&w11=vaccination&r=)  [**<**](https://www.english-corpora.org/corona/x3.asp?node=&p=14&w10=coronavirus&w11=vaccination&r=)   15 / 42  [**>**](https://www.english-corpora.org/corona/x3.asp?node=&p=16&w10=coronavirus&w11=vaccination&r=)   [**>>**](https://www.english-corpora.org/corona/x3.asp?node=&p=42&w10=coronavirus&w11=vaccination&r=) |  |
| --- | --- |

Начало формы

| CLICK FOR MORE CONTEXT | [**HELP**](javascript:newFeatures()) | [SAVE](javascript:chooser('s'))    [TRANSLATE](javascript:chooser('t'))    [ANALYZE](javascript:chooser('p')) |
| --- | --- | --- |

| [1401](https://www.english-corpora.org/corona/x4.asp?rs=&t=32943838&ID=20733556239) | [21-06-20 US](https://www.english-corpora.org/corona/x4.asp?rs=&t=32943838&ID=20733556239) | [nytimes.com](https://www.english-corpora.org/corona/x3.asp?node=&p=15&w10=coronavirus&w11=vaccination&r=) |  |  |  | Luce for The New York Times # New York State will open nine pop-up **coronavirus** **vaccination** sites this weekend at or near early polling locations in ZIP codes that have relatively |
| --- | --- | --- | --- | --- | --- | --- |
| [1402](https://www.english-corpora.org/corona/x4.asp?rs=&t=32946915&ID=20740546913) | [21-06-21 US](https://www.english-corpora.org/corona/x4.asp?rs=&t=32946915&ID=20740546913) | [aljazeera.com](https://www.english-corpora.org/corona/x3.asp?node=&p=15&w10=coronavirus&w11=vaccination&r=) |  |  |  | on Monday revealed allocation plans for 55 million **coronavirus** **vaccination** doses, amid what officials say are logistical challenges that have delayed a commitment to |
| [1403](https://www.english-corpora.org/corona/x4.asp?rs=&t=32949382&ID=20756642560) | [21-06-22 US](https://www.english-corpora.org/corona/x4.asp?rs=&t=32949382&ID=20756642560) | [washingtonexaminer.com](https://www.english-corpora.org/corona/x3.asp?node=&p=15&w10=coronavirus&w11=vaccination&r=) |  |  |  | in the United States healthcare system. # The White House will refocus its **coronavirus** **vaccination** efforts Tuesday on young adults between the ages of 18 and 26 as the White |
| [1407](https://www.english-corpora.org/corona/x4.asp?rs=&t=32957993&ID=20770239884) | [21-06-23 US](https://www.english-corpora.org/corona/x4.asp?rs=&t=32957993&ID=20770239884) | [wkyc.com](https://www.english-corpora.org/corona/x3.asp?node=&p=15&w10=coronavirus&w11=vaccination&r=) [(3)](https://www.english-corpora.org/corona/duplicates1.asp?xx=1406&n=3) |  |  |  | . # The effort was designed as an incentive to help boost declining **coronavirus** **vaccination** numbers, and while it did work in the initial days and weeks, those |
| [1409](https://www.english-corpora.org/corona/x4.asp?rs=&t=32970406&ID=20802352397) | [21-06-26 US](https://www.english-corpora.org/corona/x4.asp?rs=&t=32970406&ID=20802352397) | [wftv.com](https://www.english-corpora.org/corona/x3.asp?node=&p=15&w10=coronavirus&w11=vaccination&r=) [(1)](https://www.english-corpora.org/corona/duplicates1.asp?xx=1408&n=1) |  |  |  | Monday, May 31, 2021 file photo, a woman receives a **coronavirus** **vaccination** at the Kololo airstrip in Kampala, Uganda. Virus cases are surging in Uganda |
| [1410](https://www.english-corpora.org/corona/x4.asp?rs=&t=32974198&ID=20815868891) | [21-06-27 US](https://www.english-corpora.org/corona/x4.asp?rs=&t=32974198&ID=20815868891) | [registerguard.com](https://www.english-corpora.org/corona/x3.asp?node=&p=15&w10=coronavirus&w11=vaccination&r=) |  |  |  | death count remained at 2,763. # The Oregon Health Authority continues to track **coronavirus** **vaccination** distribution; the seven-day running average is 7,755 doses per day, continuing the state |
| [1411](https://www.english-corpora.org/corona/x4.asp?rs=&t=32978689&ID=20826542589) | [21-06-28 US](https://www.english-corpora.org/corona/x4.asp?rs=&t=32978689&ID=20826542589) | [ndtv.com](https://www.english-corpora.org/corona/x3.asp?node=&p=15&w10=coronavirus&w11=vaccination&r=) |  |  |  | On Friday, thousands of opposition activists rallied in Pretoria to demand a faster **coronavirus** **vaccination** rollout. # President Cyril Ramaphosa is expected to broaden lockdown restrictions during an address |
| [1412](https://www.english-corpora.org/corona/x4.asp?rs=&t=43142860&ID=20780309575) | [21-06-24 US](https://www.english-corpora.org/corona/x4.asp?rs=&t=43142860&ID=20780309575) | [talkingpointsmemo.com](https://www.english-corpora.org/corona/x3.asp?node=&p=15&w10=coronavirus&w11=vaccination&r=) |  |  |  | of Holon near Tel Aviv on June 21, 2021, as Israel begins **coronavirus** **vaccination** campaign for 12 to... A boy receives a dose of the Pfizer/BioNTech Covid-19 vaccine |
| [1413](https://www.english-corpora.org/corona/x4.asp?rs=&t=43142860&ID=20780309615) | [21-06-24 US](https://www.english-corpora.org/corona/x4.asp?rs=&t=43142860&ID=20780309615) | [talkingpointsmemo.com](https://www.english-corpora.org/corona/x3.asp?node=&p=15&w10=coronavirus&w11=vaccination&r=) |  |  |  | of Holon near Tel Aviv on June 21, 2021, as Israel begins **coronavirus** **vaccination** campaign for 12 to 15-year-olds. - Israel is now urging more 12- to 15-year-olds to |
| [1414](https://www.english-corpora.org/corona/x4.asp?rs=&t=43168083&ID=20851170859) | [21-06-30 US](https://www.english-corpora.org/corona/x4.asp?rs=&t=43168083&ID=20851170859) | [hollywoodlife.com](https://www.english-corpora.org/corona/x3.asp?node=&p=15&w10=coronavirus&w11=vaccination&r=) |  |  |  | beers. # The visit was part of White House efforts to ramp up **coronavirus** **vaccination** efforts in Texas, a state with a low vaccination rate. At the stadium |
| [1415](https://www.english-corpora.org/corona/x4.asp?rs=&t=52846535&ID=20714203403) | [21-06-18 US](https://www.english-corpora.org/corona/x4.asp?rs=&t=52846535&ID=20714203403) | [ksby.com](https://www.english-corpora.org/corona/x3.asp?node=&p=15&w10=coronavirus&w11=vaccination&r=) |  |  |  | offers digital record of **coronavirus** **vaccination** # (Copyright 2021 The Associated Press. All rights reserved. This material may |
| [1416](https://www.english-corpora.org/corona/x4.asp?rs=&t=52880881&ID=20827714523) | [21-06-28 US](https://www.english-corpora.org/corona/x4.asp?rs=&t=52880881&ID=20827714523) | [pri.org](https://www.english-corpora.org/corona/x3.asp?node=&p=15&w10=coronavirus&w11=vaccination&r=) |  |  |  | Now it's changing physical spaces, too. # With the rollout of **coronavirus** **vaccination** campaigns, several countries having begun the easing of lockdown restrictions. As restrictions lift |
| [1417](https://www.english-corpora.org/corona/x4.asp?rs=&t=63040811&ID=20712407843) | [21-06-18 US](https://www.english-corpora.org/corona/x4.asp?rs=&t=63040811&ID=20712407843) | [fox5sandiego.com](https://www.english-corpora.org/corona/x3.asp?node=&p=15&w10=coronavirus&w11=vaccination&r=) |  |  |  | get digital record of **coronavirus** **vaccination** SUBSCRIBE NOW Breaking News # SACRAMENTO, Calif. (AP) -- California |
| [1418](https://www.english-corpora.org/corona/x4.asp?rs=&t=87426281&ID=20709143048) | [21-06-17 US](https://www.english-corpora.org/corona/x4.asp?rs=&t=87426281&ID=20709143048) | [Providence Journal](https://www.english-corpora.org/corona/x3.asp?node=&p=15&w10=coronavirus&w11=vaccination&r=) |  |  |  | Among the most critical issues in his community, Javier said, is the **coronavirus** **vaccination** rate. Pointing to a lack of education on the significance of the shots, |
| [1419](https://www.english-corpora.org/corona/x4.asp?rs=&t=87427979&ID=20721397158) | [21-06-18 US](https://www.english-corpora.org/corona/x4.asp?rs=&t=87427979&ID=20721397158) | [The Hill](https://www.english-corpora.org/corona/x3.asp?node=&p=15&w10=coronavirus&w11=vaccination&r=) |  |  |  | reportedly said. # Pushback against mandated vaccines has been seen nationwide amid the **coronavirus** **vaccination** effort. # Some Republican-led states have passed laws banning businesses from requiring people to |
| [1420](https://www.english-corpora.org/corona/x4.asp?rs=&t=87433957&ID=20720177485) | [21-06-18 US](https://www.english-corpora.org/corona/x4.asp?rs=&t=87433957&ID=20720177485) | [Washington Post](https://www.english-corpora.org/corona/x3.asp?node=&p=15&w10=coronavirus&w11=vaccination&r=) |  |  |  | the event in Hampton, featuring speakers, free food trucks and a mobile **coronavirus** **vaccination** facility. # The celebration of Juneteenth was intended to " push people to think |
| [1421](https://www.english-corpora.org/corona/x4.asp?rs=&t=87436136&ID=20730250219) | [21-06-19 US](https://www.english-corpora.org/corona/x4.asp?rs=&t=87436136&ID=20730250219) | [The Washington Post on MSN.com](https://www.english-corpora.org/corona/x3.asp?node=&p=15&w10=coronavirus&w11=vaccination&r=) |  |  |  | 8 -- the poorest segment of the city and the area with the lowest **coronavirus** **vaccination** rate -- to talk to residents about the virus's disproportionately heavy impact on Black |
| [1422](https://www.english-corpora.org/corona/x4.asp?rs=&t=87439852&ID=20738611131) | [21-06-20 US](https://www.english-corpora.org/corona/x4.asp?rs=&t=87439852&ID=20738611131) | [YAHOO!News](https://www.english-corpora.org/corona/x3.asp?node=&p=15&w10=coronavirus&w11=vaccination&r=) |  |  |  | per cent of the population, have received at least one dose of a **coronavirus** **vaccination** across the US, while 44.9 per cent have had two doses, the CDC |
| [1423](https://www.english-corpora.org/corona/x4.asp?rs=&t=87439852&ID=20738611438) | [21-06-20 US](https://www.english-corpora.org/corona/x4.asp?rs=&t=87439852&ID=20738611438) | [YAHOO!News](https://www.english-corpora.org/corona/x3.asp?node=&p=15&w10=coronavirus&w11=vaccination&r=) |  |  |  | Cackler said. # In Missouri, 43.5 people have had at least one **coronavirus** **vaccination**, while in Kansas that number sits at 43.6 per cent. Over 600,000 people |
| [1424](https://www.english-corpora.org/corona/x4.asp?rs=&t=87441873&ID=20739734304) | [21-06-20 US](https://www.english-corpora.org/corona/x4.asp?rs=&t=87441873&ID=20739734304) | [SILive](https://www.english-corpora.org/corona/x3.asp?node=&p=15&w10=coronavirus&w11=vaccination&r=) |  |  |  | Zara Pinar, 15, of Prince's Bay, receives her first Pfizer **coronavirus** **vaccination** at the vaccine hub at the former Babies R Us in New Springville. ( |
| [1425](https://www.english-corpora.org/corona/x4.asp?rs=&t=87444758&ID=20749166680) | [21-06-21 US](https://www.english-corpora.org/corona/x4.asp?rs=&t=87444758&ID=20749166680) | [Evening Standard on MSN.com](https://www.english-corpora.org/corona/x3.asp?node=&p=15&w10=coronavirus&w11=vaccination&r=) |  |  |  | second vaccine doses in race against Delta variant # Wales will speed up its **coronavirus** **vaccination** programme by bringing forward second jabs for people aged over 50, the country's |
| [1427](https://www.english-corpora.org/corona/x4.asp?rs=&t=87449425&ID=20747463796) | [21-06-21 US](https://www.english-corpora.org/corona/x4.asp?rs=&t=87449425&ID=20747463796) | [SFGate](https://www.english-corpora.org/corona/x3.asp?node=&p=15&w10=coronavirus&w11=vaccination&r=) [(1)](https://www.english-corpora.org/corona/duplicates1.asp?xx=1426&n=1) |  |  |  | Greens earlier this year amid discontent with a slow start to Germany's **coronavirus** **vaccination** campaign, a scandal over some lawmakers' involvement with lucrative mask procurement deals and |
| [1428](https://www.english-corpora.org/corona/x4.asp?rs=&t=87453796&ID=20763216069) | [21-06-22 US](https://www.english-corpora.org/corona/x4.asp?rs=&t=87453796&ID=20763216069) | [KTTV](https://www.english-corpora.org/corona/x3.asp?node=&p=15&w10=coronavirus&w11=vaccination&r=) |  |  |  | # California on Friday announced it will offer residents a digital record of their **coronavirus** **vaccination** that they can use to access businesses or events that require proof of inoculation. |
| [1429](https://www.english-corpora.org/corona/x4.asp?rs=&t=87454858&ID=20762200892) | [21-06-22 US](https://www.english-corpora.org/corona/x4.asp?rs=&t=87454858&ID=20762200892) | [Yahoo](https://www.english-corpora.org/corona/x3.asp?node=&p=15&w10=coronavirus&w11=vaccination&r=) |  |  |  | on behalf of the Ohio Manufacturers Association. He argued that Ohio's lagging **coronavirus** **vaccination** rate is slowing the state's economic recovery. # In reaction, the bill |
| [1430](https://www.english-corpora.org/corona/x4.asp?rs=&t=87454986&ID=20763262500) | [21-06-22 US](https://www.english-corpora.org/corona/x4.asp?rs=&t=87454986&ID=20763262500) | [MarketWatch](https://www.english-corpora.org/corona/x3.asp?node=&p=15&w10=coronavirus&w11=vaccination&r=) |  |  |  | of sensitive events, according to people familiar with the matter, despite a **coronavirus** **vaccination** campaign that has topped 1 billion doses. # The provisional timeline of the second |
| [1431](https://www.english-corpora.org/corona/x4.asp?rs=&t=87456263&ID=20761344657) | [21-06-22 US](https://www.english-corpora.org/corona/x4.asp?rs=&t=87456263&ID=20761344657) | [New York Times](https://www.english-corpora.org/corona/x3.asp?node=&p=15&w10=coronavirus&w11=vaccination&r=) |  |  |  | has had to organize regional elections, handle the expansion of the area's **coronavirus** **vaccination** program and intervene in a dispute between rivals for the post of mayor in the |
| [1432](https://www.english-corpora.org/corona/x4.asp?rs=&t=87460015&ID=20773323723) | [21-06-23 US](https://www.english-corpora.org/corona/x4.asp?rs=&t=87460015&ID=20773323723) | [The Washington Post on MSN.com](https://www.english-corpora.org/corona/x3.asp?node=&p=15&w10=coronavirus&w11=vaccination&r=) |  |  |  | flights overseen by the Federal Aviation Administration dipped below 11,000. Now, with **coronavirus** **vaccination** rates trending upward and case numbers relatively low, Americans are ready to travel again |
| [1433](https://www.english-corpora.org/corona/x4.asp?rs=&t=87462793&ID=20776074108) | [21-06-23 US](https://www.english-corpora.org/corona/x4.asp?rs=&t=87462793&ID=20776074108) | [Evening Standard on MSN.com](https://www.english-corpora.org/corona/x3.asp?node=&p=15&w10=coronavirus&w11=vaccination&r=) |  |  |  | news agency that other countries are " catching up " with the UK's **coronavirus** **vaccination** programme. # But Thursday's review of the lists which determine the quarantine and |
| [1434](https://www.english-corpora.org/corona/x4.asp?rs=&t=87466992&ID=20787433000) | [21-06-24 US](https://www.english-corpora.org/corona/x4.asp?rs=&t=87466992&ID=20787433000) | [Reuters](https://www.english-corpora.org/corona/x3.asp?node=&p=15&w10=coronavirus&w11=vaccination&r=) |  |  |  | . REUTERS/Issei Kato # TOKYO, June 10 (Reuters) - The main **coronavirus** **vaccination** centre in the Japanese capital of Tokyo will begin booking shots for people under 65 |
| [1435](https://www.english-corpora.org/corona/x4.asp?rs=&t=87472277&ID=20801373171) | [21-06-25 US](https://www.english-corpora.org/corona/x4.asp?rs=&t=87472277&ID=20801373171) | [Forbes](https://www.english-corpora.org/corona/x3.asp?node=&p=15&w10=coronavirus&w11=vaccination&r=) |  |  |  | # Under the ADA, workplace health initiatives (which would likely include a **coronavirus** **vaccination** program at work) can only solicit medical information from employees if participation in the |
| [1436](https://www.english-corpora.org/corona/x4.asp?rs=&t=87476304&ID=20798422656) | [21-06-25 US](https://www.english-corpora.org/corona/x4.asp?rs=&t=87476304&ID=20798422656) | [USA Today](https://www.english-corpora.org/corona/x3.asp?node=&p=15&w10=coronavirus&w11=vaccination&r=) |  |  |  | vaccines for everybody else # The U.S. and U.K. have some of the highest **coronavirus** **vaccination** rates in the world. Johnson has said G-7 leaders in Cornwall will be asked |
| [1437](https://www.english-corpora.org/corona/x4.asp?rs=&t=87477679&ID=20812404096) | [21-06-26 US](https://www.english-corpora.org/corona/x4.asp?rs=&t=87477679&ID=20812404096) | [Cleveland.com](https://www.english-corpora.org/corona/x3.asp?node=&p=15&w10=coronavirus&w11=vaccination&r=) |  |  |  | the Ohio Division of Wildlife to hold a " Shoot for a Shot " **coronavirus** **vaccination** clinic on Saturday. Licensed shooters who get the vaccine at the clinic will receive |
| [1438](https://www.english-corpora.org/corona/x4.asp?rs=&t=87477955&ID=20810953729) | [21-06-26 US](https://www.english-corpora.org/corona/x4.asp?rs=&t=87477955&ID=20810953729) | [The Baltimore Sun](https://www.english-corpora.org/corona/x3.asp?node=&p=15&w10=coronavirus&w11=vaccination&r=) |  |  |  | attractions. Large conventions will once again be possible after the state closes the **coronavirus** **vaccination** and testing site at the convention center by Oct. 1. # The center's |
| [1439](https://www.english-corpora.org/corona/x4.asp?rs=&t=87478703&ID=20809760432) | [21-06-26 US](https://www.english-corpora.org/corona/x4.asp?rs=&t=87478703&ID=20809760432) | [Houston Chronicle](https://www.english-corpora.org/corona/x3.asp?node=&p=15&w10=coronavirus&w11=vaccination&r=) |  |  |  | a mass **coronavirus** **vaccination** centre held in Arsenal's Emirates Stadium, in north London, Friday June 25 |
| [1440](https://www.english-corpora.org/corona/x4.asp?rs=&t=87480697&ID=20812305921) | [21-06-26 US](https://www.english-corpora.org/corona/x4.asp?rs=&t=87480697&ID=20812305921) | [UPI.com](https://www.english-corpora.org/corona/x3.asp?node=&p=15&w10=coronavirus&w11=vaccination&r=) |  |  |  | # Sobyanin told The Moscow Times earlier this month 1.3 million people have received **coronavirus** **vaccination** in about six months and another 1 million residents will be vaccinated by the end |
| [1441](https://www.english-corpora.org/corona/x4.asp?rs=&t=87483164&ID=20818393288) | [21-06-27 US](https://www.english-corpora.org/corona/x4.asp?rs=&t=87483164&ID=20818393288) | [The Washington Post on MSN.com](https://www.english-corpora.org/corona/x3.asp?node=&p=15&w10=coronavirus&w11=vaccination&r=) |  |  |  | a physician, signals she is ready for the next person to receive a **coronavirus** **vaccination** at a weekend clinic in Philadelphia. (Caroline Gutman for The Washington Post) |
| [1442](https://www.english-corpora.org/corona/x4.asp?rs=&t=87483164&ID=20818397026) | [21-06-27 US](https://www.english-corpora.org/corona/x4.asp?rs=&t=87483164&ID=20818397026) | [The Washington Post on MSN.com](https://www.english-corpora.org/corona/x3.asp?node=&p=15&w10=coronavirus&w11=vaccination&r=) |  |  |  | , " he said. # Chidinma Nwakanma applies makeup before working at a **coronavirus** **vaccination** clinic. She says she " felt a certain responsibility as a Black physician, |
| [1443](https://www.english-corpora.org/corona/x4.asp?rs=&t=87485058&ID=20818907274) | [21-06-27 US](https://www.english-corpora.org/corona/x4.asp?rs=&t=87485058&ID=20818907274) | [The Washington Post on MSN.com](https://www.english-corpora.org/corona/x3.asp?node=&p=15&w10=coronavirus&w11=vaccination&r=) |  |  |  | championship matches July 10 and 11. All ticket holders must show proof of **coronavirus** **vaccination** or a negative test taken within the previous 48 hours and remain masked when not |
| [1444](https://www.english-corpora.org/corona/x4.asp?rs=&t=87486044&ID=20818552488) | [21-06-27 US](https://www.english-corpora.org/corona/x4.asp?rs=&t=87486044&ID=20818552488) | [Washington Post](https://www.english-corpora.org/corona/x3.asp?node=&p=15&w10=coronavirus&w11=vaccination&r=) |  |  |  | by nearly 30,000 over the past seven days. Britain has a high nationwide **coronavirus** **vaccination** rate, with about 75 percent of adults having received a first dose. More |
| [1445](https://www.english-corpora.org/corona/x4.asp?rs=&t=87496737&ID=20845711252) | [21-06-29 US](https://www.english-corpora.org/corona/x4.asp?rs=&t=87496737&ID=20845711252) | [Yahoo](https://www.english-corpora.org/corona/x3.asp?node=&p=15&w10=coronavirus&w11=vaccination&r=) |  |  |  | continuing to work to persuade residents of rural parts of the state, where **coronavirus** **vaccination** rates remain low, to get their shots. The Omaha World-Herald reported that vaccination |
| [1446](https://www.english-corpora.org/corona/x4.asp?rs=&t=87503043&ID=20854154329) | [21-06-30 US](https://www.english-corpora.org/corona/x4.asp?rs=&t=87503043&ID=20854154329) | [Fox News](https://www.english-corpora.org/corona/x3.asp?node=&p=15&w10=coronavirus&w11=vaccination&r=) |  |  |  | month, CNN was mocked for publishing a report that boasted of China's **coronavirus** **vaccination** rates... according to data provided by China. The liberal network raised eyebrows with |
| [1447](https://www.english-corpora.org/corona/x4.asp?rs=&t=87504123&ID=20852872453) | [21-06-30 US](https://www.english-corpora.org/corona/x4.asp?rs=&t=87504123&ID=20852872453) | [SFGate](https://www.english-corpora.org/corona/x3.asp?node=&p=15&w10=coronavirus&w11=vaccination&r=) |  |  |  | the Indonesian city of Bekasi on the outskirts of Jakarta are holding a mass **coronavirus** **vaccination** for 25,000 people at the city's main stadium Thursday. # Officials say more |
| [1448](https://www.english-corpora.org/corona/x4.asp?rs=&t=87504123&ID=20852874168) | [21-06-30 US](https://www.english-corpora.org/corona/x4.asp?rs=&t=87504123&ID=20852874168) | [SFGate](https://www.english-corpora.org/corona/x3.asp?node=&p=15&w10=coronavirus&w11=vaccination&r=) |  |  |  | # THE HAGUE, Netherlands -- The Dutch government says it is extending the **coronavirus** **vaccination** program to cover children ages 12 to 17 to help deal with the delta variant |
| [1449](https://www.english-corpora.org/corona/x4.asp?rs=&t=63047767&ID=20746152239) | [21-06-21 CA](https://www.english-corpora.org/corona/x4.asp?rs=&t=63047767&ID=20746152239) | [therecord.com](https://www.english-corpora.org/corona/x3.asp?node=&p=15&w10=coronavirus&w11=vaccination&r=) |  |  |  | although she didn't believe it would alter plans to complete the nation's **coronavirus** **vaccination** rollout by the year's end. # New Zealand plans to use only the |
| [1450](https://www.english-corpora.org/corona/x4.asp?rs=&t=63077223&ID=20835714162) | [21-06-29 CA](https://www.english-corpora.org/corona/x4.asp?rs=&t=63077223&ID=20835714162) | [leaderpost.com](https://www.english-corpora.org/corona/x3.asp?node=&p=15&w10=coronavirus&w11=vaccination&r=) |  |  |  | tickets had sold. # Williams said attendees must present photo ID and a **coronavirus** **vaccination** card the day of the concert to enter at the lower price, which was |
| [1451](https://www.english-corpora.org/corona/x4.asp?rs=&t=43134100&ID=20751503907) | [21-06-22 GB](https://www.english-corpora.org/corona/x4.asp?rs=&t=43134100&ID=20751503907) | [inews.co.uk](https://www.english-corpora.org/corona/x3.asp?node=&p=15&w10=coronavirus&w11=vaccination&r=) |  |  |  | jabs. We do not yet know the duration of protection delivered by a **coronavirus** **vaccination**, so it would make sense to offer booster jabs to those who are most |
| [1453](https://www.english-corpora.org/corona/x4.asp?rs=&t=43138196&ID=20770433503) | [21-06-23 GB](https://www.english-corpora.org/corona/x4.asp?rs=&t=43138196&ID=20770433503) | [blogs.lse.ac.uk](https://www.english-corpora.org/corona/x3.asp?node=&p=15&w10=coronavirus&w11=vaccination&r=) [(1)](https://www.english-corpora.org/corona/duplicates1.asp?xx=1452&n=1) |  |  |  | you are in England, you can find more information on booking your **coronavirus** **vaccination** here. # This post was written in June 2021. See here for the |
| [1454](https://www.english-corpora.org/corona/x4.asp?rs=&t=43154427&ID=20813188871) | [21-06-27 GB](https://www.english-corpora.org/corona/x4.asp?rs=&t=43154427&ID=20813188871) | [expressandstar.com](https://www.english-corpora.org/corona/x3.asp?node=&p=15&w10=coronavirus&w11=vaccination&r=) |  |  |  | aged between 18 and 29 have received a jab just three weeks after the **coronavirus** **vaccination** programme was opened up to those in their 20s. # The achievement comes as |
| [1455](https://www.english-corpora.org/corona/x4.asp?rs=&t=43155414&ID=20820717059) | [21-06-28 GB](https://www.english-corpora.org/corona/x4.asp?rs=&t=43155414&ID=20820717059) | [walesonline.co.uk](https://www.english-corpora.org/corona/x3.asp?node=&p=15&w10=coronavirus&w11=vaccination&r=) |  |  |  | A mother-of-three from Leicester died after suffering a rare catastrophic reaction to the AstraZeneca **coronavirus** **vaccination**, her family has said. # Lucy Taberer, 47, fell seriously ill |
| [1456](https://www.english-corpora.org/corona/x4.asp?rs=&t=43161682&ID=20840576457) | [21-06-29 GB](https://www.english-corpora.org/corona/x4.asp?rs=&t=43161682&ID=20840576457) | [express.co.uk](https://www.english-corpora.org/corona/x3.asp?node=&p=15&w10=coronavirus&w11=vaccination&r=) |  |  |  | you come from Great Britain, you have to go into quarantine. # **Coronavirus** **vaccination** rates compared (Image: Express) # " And that's not the case |
| [1457](https://www.english-corpora.org/corona/x4.asp?rs=&t=43166730&ID=20847914251) | [21-06-30 GB](https://www.english-corpora.org/corona/x4.asp?rs=&t=43166730&ID=20847914251) | [manchestereveningnews.co.uk](https://www.english-corpora.org/corona/x3.asp?node=&p=15&w10=coronavirus&w11=vaccination&r=) |  |  |  | programme to maintain protection for people in the winter months. " # Mass **coronavirus** **vaccination** centres have been popping up across the country (Image: PA) # The |
| [1458](https://www.english-corpora.org/corona/x4.asp?rs=&t=87499935&ID=20854314899) | [21-06-30 GB](https://www.english-corpora.org/corona/x4.asp?rs=&t=87499935&ID=20854314899) | [CNN](https://www.english-corpora.org/corona/x3.asp?node=&p=15&w10=coronavirus&w11=vaccination&r=) |  |  |  | can we encourage them to get vaccinated, " Kono said. # Large-scale **coronavirus** **vaccination** sites run by the state in Tokyo and Osaka will begin offering shots to younger |
| [1459](https://www.english-corpora.org/corona/x4.asp?rs=&t=63053571&ID=20771440060) | [21-06-23 IE](https://www.english-corpora.org/corona/x4.asp?rs=&t=63053571&ID=20771440060) | [universitytimes.ie](https://www.english-corpora.org/corona/x3.asp?node=&p=15&w10=coronavirus&w11=vaccination&r=) |  |  |  | school children. # Adults aged 36 are able to register to receive a **coronavirus** **vaccination** from today. # In Harris's plan, the government and higher education institutions |
| [1460](https://www.english-corpora.org/corona/x4.asp?rs=&t=63053664&ID=20768561431) | [21-06-23 IE](https://www.english-corpora.org/corona/x4.asp?rs=&t=63053664&ID=20768561431) | [universitytimes.ie](https://www.english-corpora.org/corona/x3.asp?node=&p=15&w10=coronavirus&w11=vaccination&r=) |  |  |  | receive them as late as October. # Damien McCallion, who oversees the **coronavirus** **vaccination** rollout, today told the Oireachtas health committee that " for those in their 20s |
| [1462](https://www.english-corpora.org/corona/x4.asp?rs=&t=63060945&ID=20793456338) | [21-06-25 IE](https://www.english-corpora.org/corona/x4.asp?rs=&t=63060945&ID=20793456338) | [intellinews.com](https://www.english-corpora.org/corona/x3.asp?node=&p=15&w10=coronavirus&w11=vaccination&r=) [(1)](https://www.english-corpora.org/corona/duplicates1.asp?xx=1461&n=1) |  |  |  | The Iraqi population numbers 39mn. # Iran is attempting to accelerate its **coronavirus** **vaccination** programme. Tehran has several times complained that US sanctions, which Washington says are |
| [1463](https://www.english-corpora.org/corona/x4.asp?rs=&t=63071786&ID=20828083482) | [21-06-28 IE](https://www.english-corpora.org/corona/x4.asp?rs=&t=63071786&ID=20828083482) | [universitytimes.ie](https://www.english-corpora.org/corona/x3.asp?node=&p=15&w10=coronavirus&w11=vaccination&r=) |  |  |  | vaccinated by September. # Last week, Damien McCallion, who oversees the **coronavirus** **vaccination** rollout, told the Oireachtas health committee that " those in their 20s " will |
| [1464](https://www.english-corpora.org/corona/x4.asp?rs=&t=73150964&ID=20755628404) | [21-06-22 AU](https://www.english-corpora.org/corona/x4.asp?rs=&t=73150964&ID=20755628404) | [au.news.yahoo.com](https://www.english-corpora.org/corona/x3.asp?node=&p=15&w10=coronavirus&w11=vaccination&r=) |  |  |  | project for the delivering of medical supplies as it seeks to bolster its faltering **coronavirus** **vaccination** drive. # The closing date for expressions of interest was Tuesday, although the |
| [1465](https://www.english-corpora.org/corona/x4.asp?rs=&t=73165223&ID=20790902030) | [21-06-25 AU](https://www.english-corpora.org/corona/x4.asp?rs=&t=73165223&ID=20790902030) | [theage.com.au](https://www.english-corpora.org/corona/x3.asp?node=&p=15&w10=coronavirus&w11=vaccination&r=) |  |  |  | why it had not been made compulsory for workers to have been given a **coronavirus** **vaccination**. " Everyone who comes in has to sign a form saying they have had |
| [1466](https://www.english-corpora.org/corona/x4.asp?rs=&t=73165908&ID=20791042962) | [21-06-25 AU](https://www.english-corpora.org/corona/x4.asp?rs=&t=73165908&ID=20791042962) | [bendigoadvertiser.com.au](https://www.english-corpora.org/corona/x3.asp?node=&p=15&w10=coronavirus&w11=vaccination&r=) |  |  |  | for a redevelopment of the old hospital. Bendigo Health chair Bob Cameron said **coronavirus** **vaccination** numbers remained strong in Greater Bendigo. " When it comes to vaccine numbers, |
| [1467](https://www.english-corpora.org/corona/x4.asp?rs=&t=73165908&ID=20791043100) | [21-06-25 AU](https://www.english-corpora.org/corona/x4.asp?rs=&t=73165908&ID=20791043100) | [bendigoadvertiser.com.au](https://www.english-corpora.org/corona/x3.asp?node=&p=15&w10=coronavirus&w11=vaccination&r=) |  |  |  | any COVID-19 symptoms is urged to get tested. To register to receive your **coronavirus** **vaccination** head to: **35;467;TOOLONG - With Australian Associated Press Our journalists work hard to provide |
| [1468](https://www.english-corpora.org/corona/x4.asp?rs=&t=73175045&ID=20825256619) | [21-06-28 AU](https://www.english-corpora.org/corona/x4.asp?rs=&t=73175045&ID=20825256619) | [au.news.yahoo.com](https://www.english-corpora.org/corona/x3.asp?node=&p=15&w10=coronavirus&w11=vaccination&r=) |  |  |  | border with NSW if there's further community transmission. # People at a **coronavirus** **vaccination** at the Rocklea Showgrounds in Brisbane. Source: AAP/Dan Peled # " If we |
| [1469](https://www.english-corpora.org/corona/x4.asp?rs=&t=32946327&ID=20740467456) | [21-06-21 NZ](https://www.english-corpora.org/corona/x4.asp?rs=&t=32946327&ID=20740467456) | [horsetalk.co.nz](https://www.english-corpora.org/corona/x3.asp?node=&p=15&w10=coronavirus&w11=vaccination&r=) |  |  |  | in Japan and abroad, including the status of the different variants of the **coronavirus** **Vaccination** of those involved with the Games will continue, the IOC said. # " |
| [1470](https://www.english-corpora.org/corona/x4.asp?rs=&t=73146335&ID=20735022636) | [21-06-20 IN](https://www.english-corpora.org/corona/x4.asp?rs=&t=73146335&ID=20735022636) | [ibtimes.co.in](https://www.english-corpora.org/corona/x3.asp?node=&p=15&w10=coronavirus&w11=vaccination&r=) |  |  |  | aimed at preparing the human body to resist the coronavirus infection # As the **coronavirus** **vaccination** rollout is progressing steadily in India, women in the nation are very much concerned |
| [1471](https://www.english-corpora.org/corona/x4.asp?rs=&t=73161460&ID=20769505947) | [21-06-23 IN](https://www.english-corpora.org/corona/x4.asp?rs=&t=73161460&ID=20769505947) | [indiatvnews.com](https://www.english-corpora.org/corona/x3.asp?node=&p=15&w10=coronavirus&w11=vaccination&r=) |  |  |  | issued by nation's health directorate said. # Pakistan on Tuesday launched its **coronavirus** **vaccination** campaign after vaccines arrived from China, with a doctor becoming the first person in |
| [1473](https://www.english-corpora.org/corona/x4.asp?rs=&t=73167474&ID=20797159557) | [21-06-25 IN](https://www.english-corpora.org/corona/x4.asp?rs=&t=73167474&ID=20797159557) | [digit.in](https://www.english-corpora.org/corona/x3.asp?node=&p=15&w10=coronavirus&w11=vaccination&r=) [(1)](https://www.english-corpora.org/corona/duplicates1.asp?xx=1472&n=1) |  |  |  | book your COVID-19 vaccine slot? Paytm now allows you to book your **coronavirus** **vaccination** slot in a few easy steps. Paytm has become one of the most popular |
| [1474](https://www.english-corpora.org/corona/x4.asp?rs=&t=73182286&ID=20842842381) | [21-06-29 IN](https://www.english-corpora.org/corona/x4.asp?rs=&t=73182286&ID=20842842381) | [ibtimes.co.in](https://www.english-corpora.org/corona/x3.asp?node=&p=15&w10=coronavirus&w11=vaccination&r=) |  |  |  | potential side effects of the vaccine as it interacts with painkillers # As the **coronavirus** **vaccination** rollout is progressing steadily in India, and in different parts of the world, |
| [1475](https://www.english-corpora.org/corona/x4.asp?rs=&t=73185175&ID=20843225517) | [21-06-29 IN](https://www.english-corpora.org/corona/x4.asp?rs=&t=73185175&ID=20843225517) | [ibtimes.co.in](https://www.english-corpora.org/corona/x3.asp?node=&p=15&w10=coronavirus&w11=vaccination&r=) |  |  |  | the vaccine for the fetus or the child are not determined yet. # **Coronavirus** **vaccination** helps pregnant women to stay safe # According to the health ministry, even if |
| [1476](https://www.english-corpora.org/corona/x4.asp?rs=&t=87472661&ID=20799133626) | [21-06-25 IN](https://www.english-corpora.org/corona/x4.asp?rs=&t=87472661&ID=20799133626) | [The Financial Express](https://www.english-corpora.org/corona/x3.asp?node=&p=15&w10=coronavirus&w11=vaccination&r=) |  |  |  | of supplying vaccines to other nations who are in need of it. # **Coronavirus** **vaccination** drive in India: As India has been ramping up its coronavirus vaccination drive, |
| [1477](https://www.english-corpora.org/corona/x4.asp?rs=&t=87472661&ID=20799133639) | [21-06-25 IN](https://www.english-corpora.org/corona/x4.asp?rs=&t=87472661&ID=20799133639) | [The Financial Express](https://www.english-corpora.org/corona/x3.asp?node=&p=15&w10=coronavirus&w11=vaccination&r=) |  |  |  | # Coronavirus vaccination drive in India: As India has been ramping up its **coronavirus** **vaccination** drive, a group of experts who are included in the national COVID-19 task force |
| [1478](https://www.english-corpora.org/corona/x4.asp?rs=&t=52853077&ID=20736239357) | [21-06-20 PK](https://www.english-corpora.org/corona/x4.asp?rs=&t=52853077&ID=20736239357) | [brecorder.com](https://www.english-corpora.org/corona/x3.asp?node=&p=15&w10=coronavirus&w11=vaccination&r=) |  |  |  | " huge number " of poorer countries have had to suspend their **coronavirus** **vaccination** programmes due to a lack of doses, the World Health Organization said Friday. |
| [1479](https://www.english-corpora.org/corona/x4.asp?rs=&t=52860239&ID=20754967336) | [21-06-22 PK](https://www.english-corpora.org/corona/x4.asp?rs=&t=52860239&ID=20754967336) | [en.dailypakistan.com.pk](https://www.english-corpora.org/corona/x3.asp?node=&p=15&w10=coronavirus&w11=vaccination&r=) |  |  |  | project for the delivery of medical supplies as it seeks to bolster its faltering **coronavirus** **vaccination** drive. 52860240 TikToker who cheated |
| [1480](https://www.english-corpora.org/corona/x4.asp?rs=&t=52865309&ID=20765370081) | [21-06-23 PK](https://www.english-corpora.org/corona/x4.asp?rs=&t=52865309&ID=20765370081) | [tribune.com.pk](https://www.english-corpora.org/corona/x3.asp?node=&p=15&w10=coronavirus&w11=vaccination&r=) |  |  |  | in to improve vaccination drive # **Coronavirus** **vaccination** availability across the country improved with the arrival of 2 million doses purchased from China |
| [1481](https://www.english-corpora.org/corona/x4.asp?rs=&t=52873631&ID=20803072054) | [21-06-26 PK](https://www.english-corpora.org/corona/x4.asp?rs=&t=52873631&ID=20803072054) | [brecorder.com](https://www.english-corpora.org/corona/x3.asp?node=&p=15&w10=coronavirus&w11=vaccination&r=) |  |  |  | vaccination centers will remain closed on Sunday, announced the city's deputy commissioner on Saturday |
| [1482](https://www.english-corpora.org/corona/x4.asp?rs=&t=52873631&ID=20803072170) | [21-06-26 PK](https://www.english-corpora.org/corona/x4.asp?rs=&t=52873631&ID=20803072170) | [brecorder.com](https://www.english-corpora.org/corona/x3.asp?node=&p=15&w10=coronavirus&w11=vaccination&r=) |  |  |  | a few vaccination centers. Recently, the Punjab government increased the number of **coronavirus** **vaccination** centers to 657 in the province, including 57 in Lahore. # Vaccine shortages |
| [1483](https://www.english-corpora.org/corona/x4.asp?rs=&t=52884281&ID=20841479428) | [21-06-29 PK](https://www.english-corpora.org/corona/x4.asp?rs=&t=52884281&ID=20841479428) | [tribune.com.pk](https://www.english-corpora.org/corona/x3.asp?node=&p=15&w10=coronavirus&w11=vaccination&r=) |  |  |  | overwhelmed a **coronavirus** **vaccination** centre as the nation grapples with a shortage of shots. PHOTO: AFP # |
| [1484](https://www.english-corpora.org/corona/x4.asp?rs=&t=52884281&ID=20841479452) | [21-06-29 PK](https://www.english-corpora.org/corona/x4.asp?rs=&t=52884281&ID=20841479452) | [tribune.com.pk](https://www.english-corpora.org/corona/x3.asp?node=&p=15&w10=coronavirus&w11=vaccination&r=) |  |  |  | of shots. PHOTO: AFP # Hundreds of desperate Pakistanis overwhelmed a government-run **coronavirus** **vaccination** centre in Islamabad on Monday as the nation grapples with a shortage of life-saving shots |
| [1485](https://www.english-corpora.org/corona/x4.asp?rs=&t=52884328&ID=20834806984) | [21-06-29 PK](https://www.english-corpora.org/corona/x4.asp?rs=&t=52884328&ID=20834806984) | [pakistantoday.com.pk](https://www.english-corpora.org/corona/x3.asp?node=&p=15&w10=coronavirus&w11=vaccination&r=) |  |  |  | 52884328 # ISLAMABAD: Hundreds of desperate people overwhelmed a government-run **coronavirus** **vaccination** centre in Islamabad Monday as the nation grapples with a shortage of life-saving shots even |
| [1486](https://www.english-corpora.org/corona/x4.asp?rs=&t=52884823&ID=20834864076) | [21-06-29 PK](https://www.english-corpora.org/corona/x4.asp?rs=&t=52884823&ID=20834864076) | [samaa.tv](https://www.english-corpora.org/corona/x3.asp?node=&p=15&w10=coronavirus&w11=vaccination&r=) |  |  |  | : Covid-19 vaccination mandatory for animal sellers # The government has made **coronavirus** **vaccination** mandatory for all sellers and vendors at cattle markets this year. On Tuesday, |
| [1488](https://www.english-corpora.org/corona/x4.asp?rs=&t=52888354&ID=20849976298) | [21-06-30 PK](https://www.english-corpora.org/corona/x4.asp?rs=&t=52888354&ID=20849976298) | [dailytimes.com.pk](https://www.english-corpora.org/corona/x3.asp?node=&p=15&w10=coronavirus&w11=vaccination&r=) [(1)](https://www.english-corpora.org/corona/duplicates1.asp?xx=1487&n=1) |  |  |  | doses of the AstraZeneca vaccine in March with which it started a nationwide **coronavirus** **vaccination** programme. " The federal government has also recently received communication from the Covax facility |
| [1489](https://www.english-corpora.org/corona/x4.asp?rs=&t=52881652&ID=20837810946) | [21-06-29 BD](https://www.english-corpora.org/corona/x4.asp?rs=&t=52881652&ID=20837810946) | [dhakatribune.com](https://www.english-corpora.org/corona/x3.asp?node=&p=15&w10=coronavirus&w11=vaccination&r=) |  |  |  | are in stock now. # It is to be mentioned that the national **coronavirus** **vaccination** program started on February 7 in the country. Vaccination activities run every day from |
| [1490](https://www.english-corpora.org/corona/x4.asp?rs=&t=43134929&ID=20751596241) | [21-06-22 HK](https://www.english-corpora.org/corona/x4.asp?rs=&t=43134929&ID=20751596241) | [thestandard.com.hk](https://www.english-corpora.org/corona/x3.asp?node=&p=15&w10=coronavirus&w11=vaccination&r=) |  |  |  | Health departments across Taiwan are investigating the deaths of 67 people after receiving the **coronavirus** **vaccination**, in an effort to determine whether there was any correlation, the Central Epidemic |
| [1491](https://www.english-corpora.org/corona/x4.asp?rs=&t=43134929&ID=20751596241) | [21-06-22 HK](https://www.english-corpora.org/corona/x4.asp?rs=&t=43134929&ID=20751596241) | [thestandard.com.hk](https://www.english-corpora.org/corona/x3.asp?node=&p=15&w10=coronavirus&w11=vaccination&r=) |  |  |  | Health departments across Taiwan are investigating the deaths of 67 people after receiving the **coronavirus** **vaccination**, in an effort to determine whether there was any correlation, the Central Epidemic |
| [1492](https://www.english-corpora.org/corona/x4.asp?rs=&t=52853386&ID=20736272096) | [21-06-20 GH](https://www.english-corpora.org/corona/x4.asp?rs=&t=52853386&ID=20736272096) | [newsghana.com.gh](https://www.english-corpora.org/corona/x3.asp?node=&p=15&w10=coronavirus&w11=vaccination&r=) |  |  |  | to Receive **Coronavirus** **vaccination** in Ghana # The World Bank today approved a $200 million Ghana COVID-19 Emergency Preparedness |
| [1493](https://www.english-corpora.org/corona/x4.asp?rs=&t=87495665&ID=20845376862) | [21-06-29 --](https://www.english-corpora.org/corona/x4.asp?rs=&t=87495665&ID=20845376862) | [The Japan Times](https://www.english-corpora.org/corona/x3.asp?node=&p=15&w10=coronavirus&w11=vaccination&r=) |  |  |  | mass **coronavirus** **vaccination** centers will start inoculating people between the ages of 18 and 64 on Thursday in |
| [1494](https://www.english-corpora.org/corona/x4.asp?rs=&t=32987326&ID=20856703847) | [21-07-01 US](https://www.english-corpora.org/corona/x4.asp?rs=&t=32987326&ID=20856703847) | [sfchronicle.com](https://www.english-corpora.org/corona/x3.asp?node=&p=15&w10=coronavirus&w11=vaccination&r=) |  |  |  | Thursday, July 1, 2021. Russian health authorities on Thursday launched booster **coronavirus** **vaccination** for those who had been immunized more than six months ago, as the country |
| [1495](https://www.english-corpora.org/corona/x4.asp?rs=&t=32987834&ID=20859703310) | [21-07-01 US](https://www.english-corpora.org/corona/x4.asp?rs=&t=32987834&ID=20859703310) | [aljazeera.com](https://www.english-corpora.org/corona/x3.asp?node=&p=15&w10=coronavirus&w11=vaccination&r=) |  |  |  | were expecting and signals that the US jobs market is continuing to heal as **coronavirus** **vaccination** rates climb, business-sapping COVID-19 restrictions roll back, and American consumers -- whose spending |
| [1496](https://www.english-corpora.org/corona/x4.asp?rs=&t=32988047&ID=20859731456) | [21-07-01 US](https://www.english-corpora.org/corona/x4.asp?rs=&t=32988047&ID=20859731456) | [dailyherald.com](https://www.english-corpora.org/corona/x3.asp?node=&p=15&w10=coronavirus&w11=vaccination&r=) |  |  |  | 2021, three vials of the Moderna COVID-19 Vaccine are pictured in a new **coronavirus** **vaccination** center at the' Velodrom' (velodrome-stadium) in Berlin, Germany. The |
| [1497](https://www.english-corpora.org/corona/x4.asp?rs=&t=52894006&ID=20858281507) | [21-07-01 US](https://www.english-corpora.org/corona/x4.asp?rs=&t=52894006&ID=20858281507) | [wpxi.com](https://www.english-corpora.org/corona/x3.asp?node=&p=15&w10=coronavirus&w11=vaccination&r=) |  |  |  | czar resigned in February after a well-known journalist said he had been given a **coronavirus** **vaccination** preferentially after asking the official for one. There were also reports of lawmakers, |
| [1498](https://www.english-corpora.org/corona/x4.asp?rs=&t=52892411&ID=20858091620) | [21-07-01 PK](https://www.english-corpora.org/corona/x4.asp?rs=&t=52892411&ID=20858091620) | [tribune.com.pk](https://www.english-corpora.org/corona/x3.asp?node=&p=15&w10=coronavirus&w11=vaccination&r=) |  |  |  | a lawsuit brought by employees of a hospital who had challenged the hospital's **coronavirus** **vaccination** requirement, precisely because the mandated inoculation is for the safety of everyone and not |
| [1499](https://www.english-corpora.org/corona/x4.asp?rs=&t=52893157&ID=20861022559) | [21-07-01 PK](https://www.english-corpora.org/corona/x4.asp?rs=&t=52893157&ID=20861022559) | [samaa.tv](https://www.english-corpora.org/corona/x3.asp?node=&p=15&w10=coronavirus&w11=vaccination&r=) |  |  |  | and Karachi. On June 28, a fight broke out at a mass **coronavirus** **vaccination** centre in Islamabad's F-9 after the security staff barred people from entering the building |
| [1500](https://www.english-corpora.org/corona/x4.asp?rs=&t=63082231&ID=20858623060) | [21-07-01 ZA](https://www.english-corpora.org/corona/x4.asp?rs=&t=63082231&ID=20858623060) | [ewn.co.za](https://www.english-corpora.org/corona/x3.asp?node=&p=15&w10=coronavirus&w11=vaccination&r=) |  |  |  | million doses of the AstraZeneca vaccine in March with which it started a nationwide **coronavirus** **vaccination** programme. # " The federal government has also recently received communication from the Covax |

PAGE:   [**<<**](https://www.english-corpora.org/corona/x3.asp?node=&p=1&w10=coronavirus&w11=vaccination&r=)  [**<**](https://www.english-corpora.org/corona/x3.asp?node=&p=14&w10=coronavirus&w11=vaccination&r=)   15 / 42  [**>**](https://www.english-corpora.org/corona/x3.asp?node=&p=16&w10=coronavirus&w11=vaccination&r=)   [**>>**](https://www.english-corpora.org/corona/x3.asp?node=&p=42&w10=coronavirus&w11=vaccination&r=)

Конец формы

| PAGE:   [**<<**](https://www.english-corpora.org/corona/x3.asp?node=&p=1&w10=coronavirus&w11=vaccination&r=)  [**<**](https://www.english-corpora.org/corona/x3.asp?node=&p=15&w10=coronavirus&w11=vaccination&r=)   16 / 42  [**>**](https://www.english-corpora.org/corona/x3.asp?node=&p=17&w10=coronavirus&w11=vaccination&r=)   [**>>**](https://www.english-corpora.org/corona/x3.asp?node=&p=42&w10=coronavirus&w11=vaccination&r=) |  |
| --- | --- |

Начало формы

| CLICK FOR MORE CONTEXT | [**HELP**](javascript:newFeatures()) | [SAVE](javascript:chooser('s'))    [TRANSLATE](javascript:chooser('t'))    [ANALYZE](javascript:chooser('p')) |
| --- | --- | --- |

| [1501](https://www.english-corpora.org/corona/x4.asp?rs=&t=32902019&ID=20598661936) | [21-06-08 US](https://www.english-corpora.org/corona/x4.asp?rs=&t=32902019&ID=20598661936) | [washingtonexaminer.com](https://www.english-corpora.org/corona/x3.asp?node=&p=16&w10=coronavirus&w11=vaccination&r=) |  |  |  | on dangerous constitutional ground by passing laws forbidding private businesses to require proof of **coronavirus** **vaccination** for entrance to their premises. # If challenged by business owners with proper standing |
| --- | --- | --- | --- | --- | --- | --- |
| [1502](https://www.english-corpora.org/corona/x4.asp?rs=&t=32904485&ID=20604407477) | [21-06-08 US](https://www.english-corpora.org/corona/x4.asp?rs=&t=32904485&ID=20604407477) | [wftv.com](https://www.english-corpora.org/corona/x3.asp?node=&p=16&w10=coronavirus&w11=vaccination&r=) |  |  |  | , Nepal, Tuesday, June 8, 2021. Nepal resumed its stalled **coronavirus** **vaccination** campaign on Tuesday with 1 million doses given by China after the Himalayan nation made |
| [1503](https://www.english-corpora.org/corona/x4.asp?rs=&t=32904485&ID=20604409662) | [21-06-08 US](https://www.english-corpora.org/corona/x4.asp?rs=&t=32904485&ID=20604409662) | [wftv.com](https://www.english-corpora.org/corona/x3.asp?node=&p=16&w10=coronavirus&w11=vaccination&r=) |  |  |  | for services. # # KATHMANDU, Nepal -- Nepal has resumed its stalled **coronavirus** **vaccination** campaign with 1 million doses given by China after the Himalayan nation made international pleas |
| [1504](https://www.english-corpora.org/corona/x4.asp?rs=&t=32906742&ID=20613285185) | [21-06-09 US](https://www.english-corpora.org/corona/x4.asp?rs=&t=32906742&ID=20613285185) | [twincities.com](https://www.english-corpora.org/corona/x3.asp?node=&p=16&w10=coronavirus&w11=vaccination&r=) |  |  |  | Hubbard, director of Anoka County Public Health, said in a statement that **coronavirus** **vaccination** rates are higher there than what is typically seen for influenza vaccines. # Hubbard |
| [1505](https://www.english-corpora.org/corona/x4.asp?rs=&t=32908201&ID=20611088130) | [21-06-09 US](https://www.english-corpora.org/corona/x4.asp?rs=&t=32908201&ID=20611088130) | [wftv.com](https://www.english-corpora.org/corona/x3.asp?node=&p=16&w10=coronavirus&w11=vaccination&r=) |  |  |  | are the latest sites to offer perks to parents lining up to get a **coronavirus** **vaccination**. # Running through July 4, they are offering free child care to parents |
| [1506](https://www.english-corpora.org/corona/x4.asp?rs=&t=32911593&ID=20639713743) | [21-06-11 US](https://www.english-corpora.org/corona/x4.asp?rs=&t=32911593&ID=20639713743) | [ndtv.com](https://www.english-corpora.org/corona/x3.asp?node=&p=16&w10=coronavirus&w11=vaccination&r=) |  |  |  | getting the shot. HIGHLIGHTS # Drink enough water before and after the **Coronavirus** **Vaccination** # Stay stress free and relax for a few days after the vaccination # Do |
| [1507](https://www.english-corpora.org/corona/x4.asp?rs=&t=32915581&ID=20640294671) | [21-06-11 US](https://www.english-corpora.org/corona/x4.asp?rs=&t=32915581&ID=20640294671) | [ketv.com](https://www.english-corpora.org/corona/x3.asp?node=&p=16&w10=coronavirus&w11=vaccination&r=) |  |  |  | people. The advisers continued to direct doctors to report cases of myocarditis following **coronavirus** **vaccination**. # It's not clear what changed by June 1, when the CDC |
| [1508](https://www.english-corpora.org/corona/x4.asp?rs=&t=32920104&ID=20654135627) | [21-06-13 US](https://www.english-corpora.org/corona/x4.asp?rs=&t=32920104&ID=20654135627) | [kcci.com](https://www.english-corpora.org/corona/x3.asp?node=&p=16&w10=coronavirus&w11=vaccination&r=) |  |  |  | of his achievements, including diplomatic treaties with four Arab states and a successful **coronavirus** **vaccination** drive, before belittling the man who is replacing him.He accused Bennett of abandoning Israel |
| [1510](https://www.english-corpora.org/corona/x4.asp?rs=&t=32922131&ID=20661575547) | [21-06-14 US](https://www.english-corpora.org/corona/x4.asp?rs=&t=32922131&ID=20661575547) | [freemalaysiatoday.com](https://www.english-corpora.org/corona/x3.asp?node=&p=16&w10=coronavirus&w11=vaccination&r=) [(1)](https://www.english-corpora.org/corona/duplicates1.asp?xx=1509&n=1) |  |  |  | 7. (AP pic) # BANGKOK: Thailand's recently launched **coronavirus** **vaccination** campaign was hit by confusion in the capital on Monday after at least 20 hospitals |
| [1511](https://www.english-corpora.org/corona/x4.asp?rs=&t=32922819&ID=20661673750) | [21-06-14 US](https://www.english-corpora.org/corona/x4.asp?rs=&t=32922819&ID=20661673750) | [ketv.com](https://www.english-corpora.org/corona/x3.asp?node=&p=16&w10=coronavirus&w11=vaccination&r=) |  |  |  | received their shots so far.The South has been home to some of the lowest **coronavirus** **vaccination** rates in the country, with Mississippi, Alabama, Louisiana, Arkansas, Tennessee |
| [1513](https://www.english-corpora.org/corona/x4.asp?rs=&t=32925762&ID=20675577682) | [21-06-15 US](https://www.english-corpora.org/corona/x4.asp?rs=&t=32925762&ID=20675577682) | [pilotonline.com](https://www.english-corpora.org/corona/x3.asp?node=&p=16&w10=coronavirus&w11=vaccination&r=) [(1)](https://www.english-corpora.org/corona/duplicates1.asp?xx=1512&n=1) |  |  |  | 2021. (Stephen M. Katz/The Virginian-Pilot) # VIRGINIA BEACH -- A **coronavirus** **vaccination** clinic that has been providing shots to Virginia Beach residents for months will give its |
| [1514](https://www.english-corpora.org/corona/x4.asp?rs=&t=52812198&ID=20605261380) | [21-06-08 US](https://www.english-corpora.org/corona/x4.asp?rs=&t=52812198&ID=20605261380) | [wyff4.com](https://www.english-corpora.org/corona/x3.asp?node=&p=16&w10=coronavirus&w11=vaccination&r=) |  |  |  | COVID-19. Public health officials say about 1.6 million South Carolinians have completed the **coronavirus** **vaccination** process, or about 38% of the eligible population. # GREENVILLE, S.C. |
| [1516](https://www.english-corpora.org/corona/x4.asp?rs=&t=52812754&ID=20602773037) | [21-06-08 US](https://www.english-corpora.org/corona/x4.asp?rs=&t=52812754&ID=20602773037) | [mynorthwest.com](https://www.english-corpora.org/corona/x3.asp?node=&p=16&w10=coronavirus&w11=vaccination&r=) [(1)](https://www.english-corpora.org/corona/duplicates1.asp?xx=1515&n=1) |  |  |  | China # KATHMANDU, Nepal (AP) -- Nepal resumed its stalled **coronavirus** **vaccination** campaign on Tuesday with 1 million doses given by China after the Himalayan nation made |
| [1517](https://www.english-corpora.org/corona/x4.asp?rs=&t=52819937&ID=20625813994) | [21-06-10 US](https://www.english-corpora.org/corona/x4.asp?rs=&t=52819937&ID=20625813994) | [wsoctv.com](https://www.english-corpora.org/corona/x3.asp?node=&p=16&w10=coronavirus&w11=vaccination&r=) |  |  |  | to fully vaccinated people. The CovPass will let users download proof of their **coronavirus** **vaccination** status onto a smartphone app, allowing them easy access to restaurants, museums or |
| [1518](https://www.english-corpora.org/corona/x4.asp?rs=&t=87358443&ID=20583464304) | [21-06-06 US](https://www.english-corpora.org/corona/x4.asp?rs=&t=87358443&ID=20583464304) | [Patch](https://www.english-corpora.org/corona/x3.asp?node=&p=16&w10=coronavirus&w11=vaccination&r=) |  |  |  | 13, and her brother, Jackson, 15, proudly show off their **coronavirus** **vaccination** cards after getting their first Pfizer shots on Monday. (Melanie Skane) |
| [1519](https://www.english-corpora.org/corona/x4.asp?rs=&t=87361745&ID=20596195752) | [21-06-07 US](https://www.english-corpora.org/corona/x4.asp?rs=&t=87361745&ID=20596195752) | [Japan Today](https://www.english-corpora.org/corona/x3.asp?node=&p=16&w10=coronavirus&w11=vaccination&r=) |  |  |  | the team's home field, Tokyo Dome, to be used as a **coronavirus** **vaccination** venue for free, starting in August. # Giants owner Toshikazu Yamaguchi made the |
| [1521](https://www.english-corpora.org/corona/x4.asp?rs=&t=87363529&ID=20595523219) | [21-06-07 US](https://www.english-corpora.org/corona/x4.asp?rs=&t=87363529&ID=20595523219) | [YAHOO!News](https://www.english-corpora.org/corona/x3.asp?node=&p=16&w10=coronavirus&w11=vaccination&r=) [(1)](https://www.english-corpora.org/corona/duplicates1.asp?xx=1520&n=1) |  |  |  | # GENEVA -- The emergencies chief of the United Nations health agency says **coronavirus** **vaccination** coverage of over 80% percent is needed to significantly lower the chance that an imported |
| [1522](https://www.english-corpora.org/corona/x4.asp?rs=&t=87366750&ID=20595904550) | [21-06-07 US](https://www.english-corpora.org/corona/x4.asp?rs=&t=87366750&ID=20595904550) | [SFGate](https://www.english-corpora.org/corona/x3.asp?node=&p=16&w10=coronavirus&w11=vaccination&r=) |  |  |  | . # As of Saturday, about 1.6 million South Carolinians had completed the **coronavirus** **vaccination** process, or about 38% of the eligible population, according to public health officials |
| [1523](https://www.english-corpora.org/corona/x4.asp?rs=&t=87366904&ID=20595156717) | [21-06-07 US](https://www.english-corpora.org/corona/x4.asp?rs=&t=87366904&ID=20595156717) | [The Motley Fool](https://www.english-corpora.org/corona/x3.asp?node=&p=16&w10=coronavirus&w11=vaccination&r=) |  |  |  | Why Moderna Stock Popped Today The biotech is ramping up its global **coronavirus** **vaccination** efforts. # Joe honed his investing skills as an analyst for Stock Advisor, |
| [1524](https://www.english-corpora.org/corona/x4.asp?rs=&t=87368903&ID=20606845927) | [21-06-08 US](https://www.english-corpora.org/corona/x4.asp?rs=&t=87368903&ID=20606845927) | [Skift](https://www.english-corpora.org/corona/x3.asp?node=&p=16&w10=coronavirus&w11=vaccination&r=) |  |  |  | guessed that the best metric of resumed travel spending was a high rate of **coronavirus** **vaccination** in a local area, you would only be partly right. On the one |
| [1525](https://www.english-corpora.org/corona/x4.asp?rs=&t=87376133&ID=20618052894) | [21-06-09 US](https://www.english-corpora.org/corona/x4.asp?rs=&t=87376133&ID=20618052894) | [MarketWatch](https://www.english-corpora.org/corona/x3.asp?node=&p=16&w10=coronavirus&w11=vaccination&r=) |  |  |  | of SARS-CoV-2 is spreading widely across the country, the data suggest A **coronavirus** **vaccination** being administered in late May at the Twickenham rugby stadium in southwest London. # |
| [1526](https://www.english-corpora.org/corona/x4.asp?rs=&t=87376512&ID=20618066685) | [21-06-09 US](https://www.english-corpora.org/corona/x4.asp?rs=&t=87376512&ID=20618066685) | [YAHOO!News](https://www.english-corpora.org/corona/x3.asp?node=&p=16&w10=coronavirus&w11=vaccination&r=) |  |  |  | State Emergency Service chief officer Tim Wiebusch said. # Nepal resumed its stalled **coronavirus** **vaccination** campaign on Tuesday with 1 million doses given by China after the Himalayan nation made |
| [1527](https://www.english-corpora.org/corona/x4.asp?rs=&t=87378971&ID=20620636851) | [21-06-09 US](https://www.english-corpora.org/corona/x4.asp?rs=&t=87378971&ID=20620636851) | [Forbes](https://www.english-corpora.org/corona/x3.asp?node=&p=16&w10=coronavirus&w11=vaccination&r=) |  |  |  | HK$30 million ($3.9 million) to residents to boost the city's sluggish **coronavirus** **vaccination** rate. # Li's flagship companies CK Hutchison and CK Asset Holdings, together |
| [1528](https://www.english-corpora.org/corona/x4.asp?rs=&t=87379639&ID=20620412146) | [21-06-09 US](https://www.english-corpora.org/corona/x4.asp?rs=&t=87379639&ID=20620412146) | [New York Times](https://www.english-corpora.org/corona/x3.asp?node=&p=16&w10=coronavirus&w11=vaccination&r=) |  |  |  | , not Breathatonix. Advertisement # William Shakespeare, 81, received a **coronavirus** **vaccination** in December, the second person in Britain to do so.Credit... Pool photo by |
| [1529](https://www.english-corpora.org/corona/x4.asp?rs=&t=87380669&ID=20621861933) | [21-06-09 US](https://www.english-corpora.org/corona/x4.asp?rs=&t=87380669&ID=20621861933) | [The Independent on MSN.com](https://www.english-corpora.org/corona/x3.asp?node=&p=16&w10=coronavirus&w11=vaccination&r=) |  |  |  | Joe Middleton # Boris Johnson has said the Government will assess the impact the **coronavirus** **vaccination** programme is having before deciding whether to go ahead with the next stage of lockdown |
| [1530](https://www.english-corpora.org/corona/x4.asp?rs=&t=87382908&ID=20631094643) | [21-06-10 US](https://www.english-corpora.org/corona/x4.asp?rs=&t=87382908&ID=20631094643) | [YAHOO!News](https://www.english-corpora.org/corona/x3.asp?node=&p=16&w10=coronavirus&w11=vaccination&r=) |  |  |  | from $18 bln in paragraph 13) # TOKYO (Reuters) -The main **coronavirus** **vaccination** centre in the Japanese capital of Tokyo will begin booking shots for people under 65 |
| [1531](https://www.english-corpora.org/corona/x4.asp?rs=&t=87383208&ID=20631105770) | [21-06-10 US](https://www.english-corpora.org/corona/x4.asp?rs=&t=87383208&ID=20631105770) | [YAHOO!News](https://www.english-corpora.org/corona/x3.asp?node=&p=16&w10=coronavirus&w11=vaccination&r=) |  |  |  | 's jowly canine, who recently became the unofficial mascot of the state's **coronavirus** **vaccination** effort. # " West Virginia " has of late meant just two people, |
| [1532](https://www.english-corpora.org/corona/x4.asp?rs=&t=87383714&ID=20630702196) | [21-06-10 US](https://www.english-corpora.org/corona/x4.asp?rs=&t=87383714&ID=20630702196) | [SFGate](https://www.english-corpora.org/corona/x3.asp?node=&p=16&w10=coronavirus&w11=vaccination&r=) |  |  |  | season. # The CovPass will allow users to download a proof of their **coronavirus** **vaccination** on an app to their smart phones, which will allow them to easily enter |
| [1533](https://www.english-corpora.org/corona/x4.asp?rs=&t=87386642&ID=20631710403) | [21-06-10 US](https://www.english-corpora.org/corona/x4.asp?rs=&t=87386642&ID=20631710403) | [Bloomberg on MSN.com](https://www.english-corpora.org/corona/x3.asp?node=&p=16&w10=coronavirus&w11=vaccination&r=) |  |  |  | Takes Aim at Fake Vaccine Cards (4:33 p.m. NY) # Forging **coronavirus** **vaccination** cards would be a felony in New York under a bill passed by state lawmakers |
| [1534](https://www.english-corpora.org/corona/x4.asp?rs=&t=87388323&ID=20642750068) | [21-06-11 US](https://www.english-corpora.org/corona/x4.asp?rs=&t=87388323&ID=20642750068) | [Chicago Tribune](https://www.english-corpora.org/corona/x3.asp?node=&p=16&w10=coronavirus&w11=vaccination&r=) |  |  |  | several store chains to have recently eased their mask-wearing policies. # With local **coronavirus** **vaccination** rates among the highest in the state and hospitalization numbers the lowest they've been |
| [1535](https://www.english-corpora.org/corona/x4.asp?rs=&t=87390783&ID=20645033094) | [21-06-11 US](https://www.english-corpora.org/corona/x4.asp?rs=&t=87390783&ID=20645033094) | [The Independent on MSN.com](https://www.english-corpora.org/corona/x3.asp?node=&p=16&w10=coronavirus&w11=vaccination&r=) |  |  |  | . # " They discussed their countries' respective efforts to roll out a **coronavirus** **vaccination** programme and defeat the pandemic. They agreed to continue working closely together to share |
| [1536](https://www.english-corpora.org/corona/x4.asp?rs=&t=87391023&ID=20642879666) | [21-06-11 US](https://www.english-corpora.org/corona/x4.asp?rs=&t=87391023&ID=20642879666) | [Reuters](https://www.english-corpora.org/corona/x3.asp?node=&p=16&w10=coronavirus&w11=vaccination&r=) |  |  |  | in crisis-fighting mode until job markets are healed. # The fast rollout of **coronavirus** **vaccination** programs this year and the broad easing of restrictions on commerce and movement seemed to |
| [1537](https://www.english-corpora.org/corona/x4.asp?rs=&t=87391772&ID=20645066211) | [21-06-11 US](https://www.english-corpora.org/corona/x4.asp?rs=&t=87391772&ID=20645066211) | [USA TODAY on MSN.com](https://www.english-corpora.org/corona/x3.asp?node=&p=16&w10=coronavirus&w11=vaccination&r=) |  |  |  | vaccines for everybody else # The U.S. and U.K. have some of the highest **coronavirus** **vaccination** rates in the world. Johnson has said G-7 leaders in Cornwall will be asked |
| [1538](https://www.english-corpora.org/corona/x4.asp?rs=&t=87401067&ID=20661053846) | [21-06-13 US](https://www.english-corpora.org/corona/x4.asp?rs=&t=87401067&ID=20661053846) | [Politico](https://www.english-corpora.org/corona/x3.asp?node=&p=16&w10=coronavirus&w11=vaccination&r=) |  |  |  | schedule, a federally-recommended series of shots. But while the CDC has encouraged **coronavirus** **vaccination**, the way the shots are being given under an emergency authorization puts them in |
| [1539](https://www.english-corpora.org/corona/x4.asp?rs=&t=87401435&ID=20659921165) | [21-06-13 US](https://www.english-corpora.org/corona/x4.asp?rs=&t=87401435&ID=20659921165) | [StarTribune](https://www.english-corpora.org/corona/x3.asp?node=&p=16&w10=coronavirus&w11=vaccination&r=) |  |  |  | tickets had sold. # Williams said attendees must present photo ID and a **coronavirus** **vaccination** card the day of the concert to enter at the lower price, which was |
| [1540](https://www.english-corpora.org/corona/x4.asp?rs=&t=87403905&ID=20669948732) | [21-06-14 US](https://www.english-corpora.org/corona/x4.asp?rs=&t=87403905&ID=20669948732) | [YAHOO!News](https://www.english-corpora.org/corona/x3.asp?node=&p=16&w10=coronavirus&w11=vaccination&r=) |  |  |  | of seats in the lower house of parliament. # Thailand's recently launched **coronavirus** **vaccination** campaign was hit by confusion in the capital on Monday after at least 20 hospitals |
| [1543](https://www.english-corpora.org/corona/x4.asp?rs=&t=87408210&ID=20680135696) | [21-06-15 US](https://www.english-corpora.org/corona/x4.asp?rs=&t=87408210&ID=20680135696) | [YAHOO!News](https://www.english-corpora.org/corona/x3.asp?node=&p=16&w10=coronavirus&w11=vaccination&r=) [(2)](https://www.english-corpora.org/corona/duplicates1.asp?xx=1542&n=2) |  |  |  | shots. Maryland is in the process of closing many of its mass **coronavirus** **vaccination** sites around the state in the next six weeks and transferring resources to smaller, |
| [1544](https://www.english-corpora.org/corona/x4.asp?rs=&t=87408210&ID=20680136704) | [21-06-15 US](https://www.english-corpora.org/corona/x4.asp?rs=&t=87408210&ID=20680136704) | [YAHOO!News](https://www.english-corpora.org/corona/x3.asp?node=&p=16&w10=coronavirus&w11=vaccination&r=) |  |  |  | ... # Thailand's prime minister apologised and took the blame on Tuesday for **coronavirus** **vaccination** delays, while thousands of private companies and public organisations raced to secure doses imported |
| [1545](https://www.english-corpora.org/corona/x4.asp?rs=&t=87412532&ID=20680744047) | [21-06-15 US](https://www.english-corpora.org/corona/x4.asp?rs=&t=87412532&ID=20680744047) | [CNN on MSN.com](https://www.english-corpora.org/corona/x3.asp?node=&p=16&w10=coronavirus&w11=vaccination&r=) |  |  |  | can we encourage them to get vaccinated, " Kono said. # Large-scale **coronavirus** **vaccination** sites run by the state in Tokyo and Osaka will begin offering shots to younger |
| [1546](https://www.english-corpora.org/corona/x4.asp?rs=&t=87413258&ID=20683061652) | [21-06-15 US](https://www.english-corpora.org/corona/x4.asp?rs=&t=87413258&ID=20683061652) | [The Boston Globe](https://www.english-corpora.org/corona/x3.asp?node=&p=16&w10=coronavirus&w11=vaccination&r=) |  |  |  | recently launched an initiative with the Biden administration to provide people free rides to **coronavirus** **vaccination** appointments. And social networks and search engines have been working with public health authorities |
| [1547](https://www.english-corpora.org/corona/x4.asp?rs=&t=87414226&ID=20695623206) | [21-06-16 US](https://www.english-corpora.org/corona/x4.asp?rs=&t=87414226&ID=20695623206) | [YAHOO!News](https://www.english-corpora.org/corona/x3.asp?node=&p=16&w10=coronavirus&w11=vaccination&r=) |  |  |  | . # Thailand's prime minister apologised and took the blame on Tuesday for **coronavirus** **vaccination** delays, while thousands of private companies and public organisations raced to secure doses imported |
| [1548](https://www.english-corpora.org/corona/x4.asp?rs=&t=52810670&ID=20605071323) | [21-06-08 CA](https://www.english-corpora.org/corona/x4.asp?rs=&t=52810670&ID=20605071323) | [toronto.citynews.ca](https://www.english-corpora.org/corona/x3.asp?node=&p=16&w10=coronavirus&w11=vaccination&r=) |  |  |  | a gathering and march. # KATHMANDU, Nepal -- Nepal resumed its stalled **coronavirus** **vaccination** campaign on Tuesday with 1 million doses given by China after the Himalayan nation made |
| [1549](https://www.english-corpora.org/corona/x4.asp?rs=&t=52831735&ID=20665014162) | [21-06-14 CA](https://www.english-corpora.org/corona/x4.asp?rs=&t=52831735&ID=20665014162) | [arabtimesonline.com](https://www.english-corpora.org/corona/x3.asp?node=&p=16&w10=coronavirus&w11=vaccination&r=) |  |  |  | adopt it. The Ministry of Health, meanwhile, announced shifting Monday's **coronavirus** **vaccination** appointments from Sheikh Jaber Bridge's center to Kuwait International Fairs Ground in Mishref -- |
| [1550](https://www.english-corpora.org/corona/x4.asp?rs=&t=43077846&ID=20578478366) | [21-06-06 GB](https://www.english-corpora.org/corona/x4.asp?rs=&t=43077846&ID=20578478366) | [expressandstar.com](https://www.english-corpora.org/corona/x3.asp?node=&p=16&w10=coronavirus&w11=vaccination&r=) |  |  |  | them to rebook their appointment. # Subscribe to our daily newsletter! # **Coronavirus** **vaccination** # People who have been contacted to bring forward their second coronavirus vaccine appointment are |
| [1551](https://www.english-corpora.org/corona/x4.asp?rs=&t=43080477&ID=20580819354) | [21-06-06 GB](https://www.english-corpora.org/corona/x4.asp?rs=&t=43080477&ID=20580819354) | [thestar.co.uk](https://www.english-corpora.org/corona/x3.asp?node=&p=16&w10=coronavirus&w11=vaccination&r=) |  |  |  | just the unifying event we need right now. # The UK's successful **coronavirus** **vaccination** programme means more than 39 million adults have had at least one jab to protect |
| [1552](https://www.english-corpora.org/corona/x4.asp?rs=&t=43085546&ID=20601931193) | [21-06-08 GB](https://www.english-corpora.org/corona/x4.asp?rs=&t=43085546&ID=20601931193) | [birminghammail.co.uk](https://www.english-corpora.org/corona/x3.asp?node=&p=16&w10=coronavirus&w11=vaccination&r=) |  |  |  | . " # People can book their vaccine by visiting the NHS Book a **coronavirus** **vaccination** website or calling 119. 43085547 # The county council |
| [1553](https://www.english-corpora.org/corona/x4.asp?rs=&t=43085850&ID=20601962855) | [21-06-08 GB](https://www.english-corpora.org/corona/x4.asp?rs=&t=43085850&ID=20601962855) | [inews.co.uk](https://www.english-corpora.org/corona/x3.asp?node=&p=16&w10=coronavirus&w11=vaccination&r=) |  |  |  | vaccine. # A Government adviser has warned that allowing people to post their **coronavirus** **vaccination** status on their dating app profiles could fuel division and even alienate people from getting |
| [1554](https://www.english-corpora.org/corona/x4.asp?rs=&t=43089372&ID=20615899959) | [21-06-09 GB](https://www.english-corpora.org/corona/x4.asp?rs=&t=43089372&ID=20615899959) | [express.co.uk](https://www.english-corpora.org/corona/x3.asp?node=&p=16&w10=coronavirus&w11=vaccination&r=) |  |  |  | # People aged 25 to 29 are soon to be able to book their **coronavirus** **vaccination** according to the Health Secretary Matt Hancock's latest Covid announcement. After months of |
| [1555](https://www.english-corpora.org/corona/x4.asp?rs=&t=43089600&ID=20611207283) | [21-06-09 GB](https://www.english-corpora.org/corona/x4.asp?rs=&t=43089600&ID=20611207283) | [inews.co.uk](https://www.english-corpora.org/corona/x3.asp?node=&p=16&w10=coronavirus&w11=vaccination&r=) |  |  |  | ? # A health worker prepares a dose of the AstraZeneca/Oxford vaccine at a **coronavirus** **vaccination** centre (Photo: GABRIEL BOUYS / AFP) # Due to the link between |
| [1556](https://www.english-corpora.org/corona/x4.asp?rs=&t=43102068&ID=20649543578) | [21-06-12 GB](https://www.english-corpora.org/corona/x4.asp?rs=&t=43102068&ID=20649543578) | [express.co.uk](https://www.english-corpora.org/corona/x3.asp?node=&p=16&w10=coronavirus&w11=vaccination&r=) |  |  |  | this indirect protection through exposure found many people died as a result and therefore **coronavirus** **vaccination** was seen as the safest and easiest way to reach it. READ MORE |
| [1557](https://www.english-corpora.org/corona/x4.asp?rs=&t=43109988&ID=20678241380) | [21-06-15 GB](https://www.english-corpora.org/corona/x4.asp?rs=&t=43109988&ID=20678241380) | [birminghammail.co.uk](https://www.english-corpora.org/corona/x3.asp?node=&p=16&w10=coronavirus&w11=vaccination&r=) |  |  |  | Immunisation. # Anyone aged 23 or over is currently able to book a **coronavirus** **vaccination**. # Sir Simon Stevens, chief executive of the NHS in England, said |
| [1558](https://www.english-corpora.org/corona/x4.asp?rs=&t=63033871&ID=20704863945) | [21-06-17 IE](https://www.english-corpora.org/corona/x4.asp?rs=&t=63033871&ID=20704863945) | [intellinews.com](https://www.english-corpora.org/corona/x3.asp?node=&p=16&w10=coronavirus&w11=vaccination&r=) |  |  |  | email system. 63033871 Moscow introduces mandatory **coronavirus** **vaccination** # Russia developed the first working vaccine against coronavirus, but Moscow has the lowest |
| [1559](https://www.english-corpora.org/corona/x4.asp?rs=&t=73090360&ID=20590689481) | [21-06-07 AU](https://www.english-corpora.org/corona/x4.asp?rs=&t=73090360&ID=20590689481) | [indaily.com.au](https://www.english-corpora.org/corona/x3.asp?node=&p=16&w10=coronavirus&w11=vaccination&r=) |  |  |  | Spurrier also announced that SA Health would run a " super Thursday " late-night **coronavirus** **vaccination** drive to encourage more aged-care workers to get vaccinated. # She said the Wayville |
| [1560](https://www.english-corpora.org/corona/x4.asp?rs=&t=73134054&ID=20703122642) | [21-06-17 AU](https://www.english-corpora.org/corona/x4.asp?rs=&t=73134054&ID=20703122642) | [mamamia.com.au](https://www.english-corpora.org/corona/x3.asp?node=&p=16&w10=coronavirus&w11=vaccination&r=) |  |  |  | " **26;413;TOOLONG # The Australian Technical Advisory Group on Immunisation says the benefits of **coronavirus** **vaccination** for people aged 60 and over were greater than in younger people. # " |
| [1561](https://www.english-corpora.org/corona/x4.asp?rs=&t=32908662&ID=20624660455) | [21-06-10 NZ](https://www.english-corpora.org/corona/x4.asp?rs=&t=32908662&ID=20624660455) | [newshub.co.nz](https://www.english-corpora.org/corona/x3.asp?node=&p=16&w10=coronavirus&w11=vaccination&r=) |  |  |  | variants drive record fatalities elsewhere in Southeast Asia. # Japan # The main **coronavirus** **vaccination** centre in the Japanese capital of Tokyo will begin booking shots for people under 65 |
| [1562](https://www.english-corpora.org/corona/x4.asp?rs=&t=32927709&ID=20684580162) | [21-06-16 NZ](https://www.english-corpora.org/corona/x4.asp?rs=&t=32927709&ID=20684580162) | [scoop.co.nz](https://www.english-corpora.org/corona/x3.asp?node=&p=16&w10=coronavirus&w11=vaccination&r=) |  |  |  | to laugh or get angry at being patronised in this fashion. # The **coronavirus** **vaccination** rollout looks to be a signal example of inept organisation. We aren't getting |
| [1563](https://www.english-corpora.org/corona/x4.asp?rs=&t=73091045&ID=20590776754) | [21-06-07 IN](https://www.english-corpora.org/corona/x4.asp?rs=&t=73091045&ID=20590776754) | [businesstoday.in](https://www.english-corpora.org/corona/x3.asp?node=&p=16&w10=coronavirus&w11=vaccination&r=) |  |  |  | 's Srivastava, focus for FPIs would continue to be on the pace of **coronavirus** **vaccination** drive in India and how soon India gains back economic momentum. # " Though |
| [1564](https://www.english-corpora.org/corona/x4.asp?rs=&t=73096496&ID=20606007253) | [21-06-08 IN](https://www.english-corpora.org/corona/x4.asp?rs=&t=73096496&ID=20606007253) | [scroll.in](https://www.english-corpora.org/corona/x3.asp?node=&p=16&w10=coronavirus&w11=vaccination&r=) |  |  |  | . Prakash Singh/AFP # The Centre on Tuesday released revised its guidelines for the **coronavirus** **vaccination** drive, saying allocation of doses to states and Union Territories will depend on their |
| [1565](https://www.english-corpora.org/corona/x4.asp?rs=&t=73096496&ID=20606007569) | [21-06-08 IN](https://www.english-corpora.org/corona/x4.asp?rs=&t=73096496&ID=20606007569) | [scroll.in](https://www.english-corpora.org/corona/x3.asp?node=&p=16&w10=coronavirus&w11=vaccination&r=) |  |  |  | in the vaccine supply schedule, " the guidelines said. # India's **coronavirus** **vaccination** drive has struggled to keep pace with the demand since the fourth phase of inoculation |
| [1566](https://www.english-corpora.org/corona/x4.asp?rs=&t=73097098&ID=20606064322) | [21-06-08 IN](https://www.english-corpora.org/corona/x4.asp?rs=&t=73097098&ID=20606064322) | [scroll.in](https://www.english-corpora.org/corona/x3.asp?node=&p=16&w10=coronavirus&w11=vaccination&r=) |  |  |  | Punit Paranjpe/AFP # The Centre on Tuesday informed the Bombay High Court that near-to-door **coronavirus** **vaccination** would be a more feasible option than door-to-door inoculation for the elderly and those bed-ridden |
| [1567](https://www.english-corpora.org/corona/x4.asp?rs=&t=73129593&ID=20694173059) | [21-06-16 IN](https://www.english-corpora.org/corona/x4.asp?rs=&t=73129593&ID=20694173059) | [businesstoday.in](https://www.english-corpora.org/corona/x3.asp?node=&p=16&w10=coronavirus&w11=vaccination&r=) |  |  |  | the first death following **coronavirus** **vaccination**. The panel studying vaccine side-effects has confirmed the death of a 68-year-old man due |
| [1568](https://www.english-corpora.org/corona/x4.asp?rs=&t=73130244&ID=20690818564) | [21-06-16 IN](https://www.english-corpora.org/corona/x4.asp?rs=&t=73130244&ID=20690818564) | [orfonline.org](https://www.english-corpora.org/corona/x3.asp?node=&p=16&w10=coronavirus&w11=vaccination&r=) |  |  |  | the shortages seen nation-wide -- understanding vaccine hesitancy is important not just for the **coronavirus** **vaccination** drive, but for other large scale immunisation programmes as well as other pandemics that |
| [1570](https://www.english-corpora.org/corona/x4.asp?rs=&t=32918087&ID=20656810577) | [21-06-13 LK](https://www.english-corpora.org/corona/x4.asp?rs=&t=32918087&ID=20656810577) | [adaderana.lk](https://www.english-corpora.org/corona/x3.asp?node=&p=16&w10=coronavirus&w11=vaccination&r=) [(1)](https://www.english-corpora.org/corona/duplicates1.asp?xx=1569&n=1) |  |  |  | the China-made jab. # In addition, 396 more people completed the **coronavirus** **vaccination** process as they received the second dose of Oxford-AstraZeneca vaccine on Wednesday. The total |
| [1571](https://www.english-corpora.org/corona/x4.asp?rs=&t=32918087&ID=20656810613) | [21-06-13 LK](https://www.english-corpora.org/corona/x4.asp?rs=&t=32918087&ID=20656810613) | [adaderana.lk](https://www.english-corpora.org/corona/x3.asp?node=&p=16&w10=coronavirus&w11=vaccination&r=) |  |  |  | with this jab now stands at to 354,651. # Sri Lanka ramped up **coronavirus** **vaccination** upon the receipt of 2 million doses of Sinopharm vaccine this week. One million |
| [1572](https://www.english-corpora.org/corona/x4.asp?rs=&t=52803765&ID=20579306845) | [21-06-06 PK](https://www.english-corpora.org/corona/x4.asp?rs=&t=52803765&ID=20579306845) | [samaa.tv](https://www.english-corpora.org/corona/x3.asp?node=&p=16&w10=coronavirus&w11=vaccination&r=) |  |  |  | coronavirus **vaccination** drive in villages # Punjab has decided to start the Covid-19 vaccination drive in its |
| [1573](https://www.english-corpora.org/corona/x4.asp?rs=&t=52804076&ID=20581360047) | [21-06-06 PK](https://www.english-corpora.org/corona/x4.asp?rs=&t=52804076&ID=20581360047) | [tribune.com.pk](https://www.english-corpora.org/corona/x3.asp?node=&p=16&w10=coronavirus&w11=vaccination&r=) |  |  |  | " he added. # Meanwhile in Rawalpindi, the total district tally of **coronavirus** **vaccination** reached 397,397 with the inoculation of 12,824 more persons against the deadly virus during the |
| [1574](https://www.english-corpora.org/corona/x4.asp?rs=&t=52804559&ID=20579398204) | [21-06-06 PK](https://www.english-corpora.org/corona/x4.asp?rs=&t=52804559&ID=20579398204) | [en.dailypakistan.com.pk](https://www.english-corpora.org/corona/x3.asp?node=&p=16&w10=coronavirus&w11=vaccination&r=) |  |  |  | to get vaccinated against COVID-19. # The chief minister has also has declared **coronavirus** **vaccination** mandatory for every citizen of the province in a move to avoid the resurgence of |
| [1575](https://www.english-corpora.org/corona/x4.asp?rs=&t=52808055&ID=20589544222) | [21-06-07 PK](https://www.english-corpora.org/corona/x4.asp?rs=&t=52808055&ID=20589544222) | [en.dailypakistan.com.pk](https://www.english-corpora.org/corona/x3.asp?node=&p=16&w10=coronavirus&w11=vaccination&r=) |  |  |  | Pakistan opens first-ever drive-through vaccination center in Lahore Share # The first-ever drive-through **coronavirus** **vaccination** center was inaugurated in Lahore, capital of Punjab province, on Monday to facilitate |
| [1576](https://www.english-corpora.org/corona/x4.asp?rs=&t=52811357&ID=20602617048) | [21-06-08 PK](https://www.english-corpora.org/corona/x4.asp?rs=&t=52811357&ID=20602617048) | [tribune.com.pk](https://www.english-corpora.org/corona/x3.asp?node=&p=16&w10=coronavirus&w11=vaccination&r=) |  |  |  | been made mandatory for students. " # Further, the NCOC has made **coronavirus** **vaccination** mandatory for teachers and non-teaching staff. # According to the Express News, the |
| [1577](https://www.english-corpora.org/corona/x4.asp?rs=&t=52815316&ID=20612036000) | [21-06-09 PK](https://www.english-corpora.org/corona/x4.asp?rs=&t=52815316&ID=20612036000) | [nation.com.pk](https://www.english-corpora.org/corona/x3.asp?node=&p=16&w10=coronavirus&w11=vaccination&r=) |  |  |  | target population of 67m by the end of 2021. # The number of **coronavirus** **vaccination** centres have been increased to 677 in the province and a central vaccination centre will |
| [1578](https://www.english-corpora.org/corona/x4.asp?rs=&t=52829334&ID=20656212318) | [21-06-13 PK](https://www.english-corpora.org/corona/x4.asp?rs=&t=52829334&ID=20656212318) | [dailytimes.com.pk](https://www.english-corpora.org/corona/x3.asp?node=&p=16&w10=coronavirus&w11=vaccination&r=) |  |  |  | Muhammad Sohail Khawaja on Tuesday inaugurated **Coronavirus** **Vaccination** facility at Gujranwala Business Center for its workers' and their families. # Omar |
| [1579](https://www.english-corpora.org/corona/x4.asp?rs=&t=52835032&ID=20673910378) | [21-06-15 PK](https://www.english-corpora.org/corona/x4.asp?rs=&t=52835032&ID=20673910378) | [tribune.com.pk](https://www.english-corpora.org/corona/x3.asp?node=&p=16&w10=coronavirus&w11=vaccination&r=) |  |  |  | Headquarters and THQ Hospitals, had trained staff of the department of health at **coronavirus** **vaccination** centres, who were bound to immunise the citizens without any waiting in the first |
| [1581](https://www.english-corpora.org/corona/x4.asp?rs=&t=52835032&ID=20673910416) | [21-06-15 PK](https://www.english-corpora.org/corona/x4.asp?rs=&t=52835032&ID=20673910416) | [tribune.com.pk](https://www.english-corpora.org/corona/x3.asp?node=&p=16&w10=coronavirus&w11=vaccination&r=) [(1)](https://www.english-corpora.org/corona/duplicates1.asp?xx=1580&n=1) |  |  |  | doctors and paramedical staff of the health department, had been deployed at **coronavirus** **vaccination** centres in the district where they were performing their official duties. # However, |
| [1583](https://www.english-corpora.org/corona/x4.asp?rs=&t=52835032&ID=20673910514) | [21-06-15 PK](https://www.english-corpora.org/corona/x4.asp?rs=&t=52835032&ID=20673910514) | [tribune.com.pk](https://www.english-corpora.org/corona/x3.asp?node=&p=16&w10=coronavirus&w11=vaccination&r=) [(1)](https://www.english-corpora.org/corona/duplicates1.asp?xx=1582&n=1) |  |  |  | no harm has been reported so far. # The DC inaugurated the **coronavirus** **vaccination** camp at the Metropolitan Corporation in Bhakkar. # Speaking on the occasion, Bhakkar |
| [1585](https://www.english-corpora.org/corona/x4.asp?rs=&t=52835181&ID=20678811831) | [21-06-15 PK](https://www.english-corpora.org/corona/x4.asp?rs=&t=52835181&ID=20678811831) | [suchtv.pk](https://www.english-corpora.org/corona/x3.asp?node=&p=16&w10=coronavirus&w11=vaccination&r=) [(1)](https://www.english-corpora.org/corona/duplicates1.asp?xx=1584&n=1) |  |  |  | (ECL). Rasheed was speaking to the media after inaugurating a **coronavirus** **vaccination** facility at CDA Medical Centre, Qazafi Chowk, G-9 Markaz, in Islamabad. |
| [1586](https://www.english-corpora.org/corona/x4.asp?rs=&t=52835990&ID=20678918902) | [21-06-15 PK](https://www.english-corpora.org/corona/x4.asp?rs=&t=52835990&ID=20678918902) | [dnd.com.pk](https://www.english-corpora.org/corona/x3.asp?node=&p=16&w10=coronavirus&w11=vaccination&r=) |  |  |  | plan to vaccinate all dwellers of Lahore within six months and open as many **Coronavirus** **Vaccination** Centers as possible in this regard. # The Deputy Commissioner Lahore also urged the |
| [1587](https://www.english-corpora.org/corona/x4.asp?rs=&t=52843078&ID=20704719657) | [21-06-17 PK](https://www.english-corpora.org/corona/x4.asp?rs=&t=52843078&ID=20704719657) | [pakistantoday.com.pk](https://www.english-corpora.org/corona/x3.asp?node=&p=16&w10=coronavirus&w11=vaccination&r=) |  |  |  | public awareness campaign has been launched in collaboration with the private sector. The **coronavirus** **vaccination** centres have been formed at government and the privately owned hospitals, the meeting was |
| [1588](https://www.english-corpora.org/corona/x4.asp?rs=&t=52816871&ID=20628160307) | [21-06-10 BD](https://www.english-corpora.org/corona/x4.asp?rs=&t=52816871&ID=20628160307) | [unb.com.bd](https://www.english-corpora.org/corona/x3.asp?node=&p=16&w10=coronavirus&w11=vaccination&r=) |  |  |  | # AP/UNB - AP/UNB # A woman receives the AstraZeneca vaccine during a mass **coronavirus** **vaccination** for public transport workers at the Kampung Rambutan Bus Terminal in Jakarta, Indonesia, |
| [1589](https://www.english-corpora.org/corona/x4.asp?rs=&t=52840717&ID=20699961421) | [21-06-17 BD](https://www.english-corpora.org/corona/x4.asp?rs=&t=52840717&ID=20699961421) | [unb.com.bd](https://www.english-corpora.org/corona/x3.asp?node=&p=16&w10=coronavirus&w11=vaccination&r=) |  |  |  | , surges worsen shortages of vaccines # A woman receives a **coronavirus** **vaccination** at the Kololo airstrip in Kampala, Uganda, Monday, May 31, 2021 |
| [1590](https://www.english-corpora.org/corona/x4.asp?rs=&t=43105356&ID=20655895284) | [21-06-13 SG](https://www.english-corpora.org/corona/x4.asp?rs=&t=43105356&ID=20655895284) | [ucanews.com](https://www.english-corpora.org/corona/x3.asp?node=&p=16&w10=coronavirus&w11=vaccination&r=) |  |  |  | government-run hospital, told UCA News that medical experts can explain to people that **coronavirus** **vaccination** is as safe as taking jabs for malaria and hepatitis. # " We can |
| [1591](https://www.english-corpora.org/corona/x4.asp?rs=&t=43111155&ID=20675963753) | [21-06-15 SG](https://www.english-corpora.org/corona/x4.asp?rs=&t=43111155&ID=20675963753) | [channelnewsasia.com](https://www.english-corpora.org/corona/x3.asp?node=&p=16&w10=coronavirus&w11=vaccination&r=) |  |  |  | says he regrets not prioritizing **coronavirus** **vaccination** Golf: Rahm says he regrets not prioritizing coronavirus vaccination # Spain's Jon |
| [1592](https://www.english-corpora.org/corona/x4.asp?rs=&t=43111155&ID=20675963764) | [21-06-15 SG](https://www.english-corpora.org/corona/x4.asp?rs=&t=43111155&ID=20675963764) | [channelnewsasia.com](https://www.english-corpora.org/corona/x3.asp?node=&p=16&w10=coronavirus&w11=vaccination&r=) |  |  |  | regrets not prioritizing coronavirus vaccination Golf: Rahm says he regrets not prioritizing **coronavirus** **vaccination** # Spain's Jon Rahm watches during a US Open practice round after limited preparation |
| [1593](https://www.english-corpora.org/corona/x4.asp?rs=&t=43111155&ID=20675963828) | [21-06-15 SG](https://www.english-corpora.org/corona/x4.asp?rs=&t=43111155&ID=20675963828) | [channelnewsasia.com](https://www.english-corpora.org/corona/x3.asp?node=&p=16&w10=coronavirus&w11=vaccination&r=) |  |  |  | Bookmark # SAN DIEGO, California: Jon Rahm acknowledges that not prioritising a **coronavirus** **vaccination** proved very costly, but he has been cleared to play again and vows to |
| [1594](https://www.english-corpora.org/corona/x4.asp?rs=&t=43116046&ID=20689164619) | [21-06-16 SG](https://www.english-corpora.org/corona/x4.asp?rs=&t=43116046&ID=20689164619) | [asiaone.com](https://www.english-corpora.org/corona/x3.asp?node=&p=16&w10=coronavirus&w11=vaccination&r=) |  |  |  | Prime Minister apologised and took the blame on Tuesday (June 15) for **coronavirus** **vaccination** delays, while thousands of private companies and public organisations raced to secure doses imported |
| [1595](https://www.english-corpora.org/corona/x4.asp?rs=&t=52810860&ID=20602548972) | [21-06-08 NG](https://www.english-corpora.org/corona/x4.asp?rs=&t=52810860&ID=20602548972) | [proshareng.com](https://www.english-corpora.org/corona/x3.asp?node=&p=16&w10=coronavirus&w11=vaccination&r=) |  |  |  | # May 2021 Inflation Expectation: Inflation in Advanced Climes Sees Uptrend - As **coronavirus** **vaccination** and bolder steps at reopening economies gains momentum, concerns around the build-up of inflationary |
| [1596](https://www.english-corpora.org/corona/x4.asp?rs=&t=52838895&ID=20693212304) | [21-06-16 NG](https://www.english-corpora.org/corona/x4.asp?rs=&t=52838895&ID=20693212304) | [nairametrics.com](https://www.english-corpora.org/corona/x3.asp?node=&p=16&w10=coronavirus&w11=vaccination&r=) |  |  |  | as major economies continue to reopen from the Covid-19 lockdown, assisted by the **coronavirus** **vaccination** programmes. # According to a report from oilprice.com, analysts had initially predicted a |
| [1597](https://www.english-corpora.org/corona/x4.asp?rs=&t=87371712&ID=20606982717) | [21-06-08 --](https://www.english-corpora.org/corona/x4.asp?rs=&t=87371712&ID=20606982717) | [France 24](https://www.english-corpora.org/corona/x3.asp?node=&p=16&w10=coronavirus&w11=vaccination&r=) |  |  |  | for use in the bloc. # After a sluggish start, France's **coronavirus** **vaccination** rollout has gained pace in recent weeks, with some 23 million people -- a |
| [1598](https://www.english-corpora.org/corona/x4.asp?rs=&t=87398206&ID=20658956224) | [21-06-13 --](https://www.english-corpora.org/corona/x4.asp?rs=&t=87398206&ID=20658956224) | [mainichi.jp](https://www.english-corpora.org/corona/x3.asp?node=&p=16&w10=coronavirus&w11=vaccination&r=) |  |  |  | under 65 in Japan at same time # TOKYO (Kyodo) -- The **coronavirus** **vaccination** rollout for people under the age of 65 in Japan will be carried out at |
| [1599](https://www.english-corpora.org/corona/x4.asp?rs=&t=87406327&ID=20670233360) | [21-06-14 --](https://www.english-corpora.org/corona/x4.asp?rs=&t=87406327&ID=20670233360) | [The Peninsula](https://www.english-corpora.org/corona/x3.asp?node=&p=16&w10=coronavirus&w11=vaccination&r=) |  |  |  | facility. # The Ministry of Public Health has been able to give the **Coronavirus** **vaccination** to a large percentage of teachers and administrators in schools, as the State of |
| [1600](https://www.english-corpora.org/corona/x4.asp?rs=&t=87406327&ID=20670233360) | [21-06-14 --](https://www.english-corpora.org/corona/x4.asp?rs=&t=87406327&ID=20670233360) | [The Peninsula](https://www.english-corpora.org/corona/x3.asp?node=&p=16&w10=coronavirus&w11=vaccination&r=) |  |  |  | facility. # The Ministry of Public Health has been able to give the **Coronavirus** **vaccination** to a large percentage of teachers and administrators in schools, as the State of |

PAGE:   [**<<**](https://www.english-corpora.org/corona/x3.asp?node=&p=1&w10=coronavirus&w11=vaccination&r=)  [**<**](https://www.english-corpora.org/corona/x3.asp?node=&p=15&w10=coronavirus&w11=vaccination&r=)   16 / 42  [**>**](https://www.english-corpora.org/corona/x3.asp?node=&p=17&w10=coronavirus&w11=vaccination&r=)   [**>>**](https://www.english-corpora.org/corona/x3.asp?node=&p=42&w10=coronavirus&w11=vaccination&r=)

Конец формы

| PAGE:   [**<<**](https://www.english-corpora.org/corona/x3.asp?node=&p=1&w10=coronavirus&w11=vaccination&r=)  [**<**](https://www.english-corpora.org/corona/x3.asp?node=&p=16&w10=coronavirus&w11=vaccination&r=)   17 / 42  [**>**](https://www.english-corpora.org/corona/x3.asp?node=&p=18&w10=coronavirus&w11=vaccination&r=)   [**>>**](https://www.english-corpora.org/corona/x3.asp?node=&p=42&w10=coronavirus&w11=vaccination&r=) |  |
| --- | --- |

Начало формы

| CLICK FOR MORE CONTEXT | [**HELP**](javascript:newFeatures()) | [SAVE](javascript:chooser('s'))    [TRANSLATE](javascript:chooser('t'))    [ANALYZE](javascript:chooser('p')) |
| --- | --- | --- |

| [1601](https://www.english-corpora.org/corona/x4.asp?rs=&t=32868657&ID=20490247929) | [21-05-28 US](https://www.english-corpora.org/corona/x4.asp?rs=&t=32868657&ID=20490247929) | [bostonherald.com](https://www.english-corpora.org/corona/x3.asp?node=&p=17&w10=coronavirus&w11=vaccination&r=) |  |  |  | , a Super Bowl trip and other prizes to eligible customers who get a **coronavirus** **vaccination** at one of its pharmacies, the company announced Thursday. # Woonsocket, R.I.-based |
| --- | --- | --- | --- | --- | --- | --- |
| [1602](https://www.english-corpora.org/corona/x4.asp?rs=&t=87301223&ID=20483630346) | [21-05-27 US](https://www.english-corpora.org/corona/x4.asp?rs=&t=87301223&ID=20483630346) | [YAHOO!News](https://www.english-corpora.org/corona/x3.asp?node=&p=17&w10=coronavirus&w11=vaccination&r=) |  |  |  | the traditional start of the summer driving season, as fuel demand surges alongside **coronavirus** **vaccination** rates. Further, some areas of the country, such as Washington, D.C. |
| [1603](https://www.english-corpora.org/corona/x4.asp?rs=&t=87303560&ID=20485233392) | [21-05-27 US](https://www.english-corpora.org/corona/x4.asp?rs=&t=87303560&ID=20485233392) | [Yahoo](https://www.english-corpora.org/corona/x3.asp?node=&p=17&w10=coronavirus&w11=vaccination&r=) |  |  |  | over a third of the overall total. This month, India opened its **coronavirus** **vaccination** campaign to everyone aged 18 or older. # The group behind the SolarWinds cyber |
| [1620](https://www.english-corpora.org/corona/x4.asp?rs=&t=87306449&ID=20486744665) | [21-05-27 US](https://www.english-corpora.org/corona/x4.asp?rs=&t=87306449&ID=20486744665) | [Reuters](https://www.english-corpora.org/corona/x3.asp?node=&p=17&w10=coronavirus&w11=vaccination&r=) [(16)](https://www.english-corpora.org/corona/duplicates1.asp?xx=1619&n=16) |  |  |  | essential work, healthcare, grocery shopping, exercise or to get a **coronavirus** **vaccination**. Masks must be worn at all times when they leave homes. # The |
| [1621](https://www.english-corpora.org/corona/x4.asp?rs=&t=87306510&ID=20483566089) | [21-05-27 US](https://www.english-corpora.org/corona/x4.asp?rs=&t=87306510&ID=20483566089) | [Yahoo](https://www.english-corpora.org/corona/x3.asp?node=&p=17&w10=coronavirus&w11=vaccination&r=) |  |  |  | over a third of the overall total. This month, India opened its **coronavirus** **vaccination** campaign to everyone aged 18 or older. # The group behind the SolarWinds cyber |
| [1629](https://www.english-corpora.org/corona/x4.asp?rs=&t=87307425&ID=20493870021) | [21-05-28 US](https://www.english-corpora.org/corona/x4.asp?rs=&t=87307425&ID=20493870021) | [Variety](https://www.english-corpora.org/corona/x3.asp?node=&p=17&w10=coronavirus&w11=vaccination&r=) [(7)](https://www.english-corpora.org/corona/duplicates1.asp?xx=1628&n=7) |  |  |  | . Safety protocols will also be in place, mandating proof of a **coronavirus** **vaccination** or negative test result within 48 hours of attendance. # This year's convention |
| [1630](https://www.english-corpora.org/corona/x4.asp?rs=&t=87307637&ID=20495565927) | [21-05-28 US](https://www.english-corpora.org/corona/x4.asp?rs=&t=87307637&ID=20495565927) | [YAHOO!News](https://www.english-corpora.org/corona/x3.asp?node=&p=17&w10=coronavirus&w11=vaccination&r=) |  |  |  | brings her into close contact with patients, but says she was denied a **coronavirus** **vaccination** as her superiors did not consider her to be a frontline worker. In despair |
| [1631](https://www.english-corpora.org/corona/x4.asp?rs=&t=87308775&ID=20496654142) | [21-05-28 US](https://www.english-corpora.org/corona/x4.asp?rs=&t=87308775&ID=20496654142) | [Politico](https://www.english-corpora.org/corona/x3.asp?node=&p=17&w10=coronavirus&w11=vaccination&r=) |  |  |  | own, according to the disclosure. # Following the meeting he spoke about **coronavirus** **vaccination** efforts and the new CDC guidance on masks in the Rose Garden. Chief of |
| [1632](https://www.english-corpora.org/corona/x4.asp?rs=&t=87311731&ID=20493410649) | [21-05-28 US](https://www.english-corpora.org/corona/x4.asp?rs=&t=87311731&ID=20493410649) | [National Review](https://www.english-corpora.org/corona/x3.asp?node=&p=17&w10=coronavirus&w11=vaccination&r=) |  |  |  | Director Dr. Anthony Fauci stands by during an event to commemorate the 50 millionth **coronavirus** **vaccination** in the South Court Auditorium at the White House, February 25, 2021. |
| [1633](https://www.english-corpora.org/corona/x4.asp?rs=&t=87312088&ID=20497473394) | [21-05-28 US](https://www.english-corpora.org/corona/x4.asp?rs=&t=87312088&ID=20497473394) | [New York Times](https://www.english-corpora.org/corona/x3.asp?node=&p=17&w10=coronavirus&w11=vaccination&r=) |  |  |  | California announced on Thursday a $116.5 million giveaway to residents who have received a **coronavirus** **vaccination** in an effort to motivate people to get a shot. # Friday, June |
| [1635](https://www.english-corpora.org/corona/x4.asp?rs=&t=87312723&ID=20502481237) | [21-05-29 US](https://www.english-corpora.org/corona/x4.asp?rs=&t=87312723&ID=20502481237) | [YAHOO!News](https://www.english-corpora.org/corona/x3.asp?node=&p=17&w10=coronavirus&w11=vaccination&r=) [(1)](https://www.english-corpora.org/corona/duplicates1.asp?xx=1634&n=1) |  |  |  | her arms for Vanessa right away. # NHS staff to face compulsory **coronavirus** **vaccination** Hancock facing fresh questions over Covid in care homes Vaccine clot patients' closely monitored |
| [1636](https://www.english-corpora.org/corona/x4.asp?rs=&t=87313597&ID=20503024574) | [21-05-29 US](https://www.english-corpora.org/corona/x4.asp?rs=&t=87313597&ID=20503024574) | [ABC](https://www.english-corpora.org/corona/x3.asp?node=&p=17&w10=coronavirus&w11=vaccination&r=) |  |  |  | Oklahoma state agencies will be barred from requiring a mask or **coronavirus** **vaccination** as a condition of being allowed to enter a state building or office under an |
| [1638](https://www.english-corpora.org/corona/x4.asp?rs=&t=87314923&ID=20502616559) | [21-05-29 US](https://www.english-corpora.org/corona/x4.asp?rs=&t=87314923&ID=20502616559) | [Washington Post](https://www.english-corpora.org/corona/x3.asp?node=&p=17&w10=coronavirus&w11=vaccination&r=) [(1)](https://www.english-corpora.org/corona/duplicates1.asp?xx=1637&n=1) |  |  |  | had sold. # Williams said attendees must present photo ID and a **coronavirus** **vaccination** card the day of the concert to enter at the lower price, which was |
| [1639](https://www.english-corpora.org/corona/x4.asp?rs=&t=87315723&ID=20502674690) | [21-05-29 US](https://www.english-corpora.org/corona/x4.asp?rs=&t=87315723&ID=20502674690) | [NBC News](https://www.english-corpora.org/corona/x3.asp?node=&p=17&w10=coronavirus&w11=vaccination&r=) |  |  |  | Oklahoma state agencies will be barred from requiring a mask or **coronavirus** **vaccination** as a condition of being allowed to enter a state building or office under an |
| [1640](https://www.english-corpora.org/corona/x4.asp?rs=&t=87323952&ID=20521776363) | [21-05-31 US](https://www.english-corpora.org/corona/x4.asp?rs=&t=87323952&ID=20521776363) | [Washington Examiner](https://www.english-corpora.org/corona/x3.asp?node=&p=17&w10=coronavirus&w11=vaccination&r=) |  |  |  | CDC study finds # Counties with higher percentages of minorities have higher rates of **coronavirus** **vaccination** than those with lower percentages, according to new research from the Centers for Disease |
| [1641](https://www.english-corpora.org/corona/x4.asp?rs=&t=43054382&ID=20500947019) | [21-05-29 GB](https://www.english-corpora.org/corona/x4.asp?rs=&t=43054382&ID=20500947019) | [thenational.scot](https://www.english-corpora.org/corona/x3.asp?node=&p=17&w10=coronavirus&w11=vaccination&r=) |  |  |  | Exeter centre Henry Slade has clarified comments he made about refusing to take the **coronavirus** **vaccination** on the grounds he is healthy. # Slade, a Type-1 diabetic, told |
| [1642](https://www.english-corpora.org/corona/x4.asp?rs=&t=43057333&ID=20515383043) | [21-05-31 GB](https://www.english-corpora.org/corona/x4.asp?rs=&t=43057333&ID=20515383043) | [express.co.uk](https://www.english-corpora.org/corona/x3.asp?node=&p=17&w10=coronavirus&w11=vaccination&r=) |  |  |  | ? WHO warns about reinfection from new COVID variants # The UK's **coronavirus** **vaccination** programme has been a rousing success in what has been a catastrophic coronavirus response. |
| [1643](https://www.english-corpora.org/corona/x4.asp?rs=&t=43057377&ID=20518830271) | [21-05-31 GB](https://www.english-corpora.org/corona/x4.asp?rs=&t=43057377&ID=20518830271) | [lexology.com](https://www.english-corpora.org/corona/x3.asp?node=&p=17&w10=coronavirus&w11=vaccination&r=) |  |  |  | The Senate's four Hispanic Democrats have urged the Biden administration to boost lagging **coronavirus** **vaccination** rates in Latino communities through greater outreach. Sens. Alex Padilla (CA) |
| [1644](https://www.english-corpora.org/corona/x4.asp?rs=&t=87308046&ID=20495581152) | [21-05-28 GB](https://www.english-corpora.org/corona/x4.asp?rs=&t=87308046&ID=20495581152) | [Sky](https://www.english-corpora.org/corona/x3.asp?node=&p=17&w10=coronavirus&w11=vaccination&r=) |  |  |  | until " they hear otherwise from health officials. # But the HSE said **coronavirus** **vaccination** appointments were not affected by the attack and are going ahead as planned. # |
| [1645](https://www.english-corpora.org/corona/x4.asp?rs=&t=87322832&ID=20520669070) | [21-05-31 IE](https://www.english-corpora.org/corona/x4.asp?rs=&t=87322832&ID=20520669070) | [The Irish Times](https://www.english-corpora.org/corona/x3.asp?node=&p=17&w10=coronavirus&w11=vaccination&r=) |  |  |  | hospitals implement their business continuity plans, " it said. # Although the **coronavirus** **vaccination** programme was not affected by the attacks, the centre said the HSE has curbed |
| [1646](https://www.english-corpora.org/corona/x4.asp?rs=&t=73065121&ID=20516571342) | [21-05-31 AU](https://www.english-corpora.org/corona/x4.asp?rs=&t=73065121&ID=20516571342) | [canberratimes.com.au](https://www.english-corpora.org/corona/x3.asp?node=&p=17&w10=coronavirus&w11=vaccination&r=) |  |  |  | 73065121 # Medical experts will review a decision not to make **coronavirus** **vaccination** mandatory for aged care workers after staff in Melbourne contracted the deadly disease. Victoria |
| [1647](https://www.english-corpora.org/corona/x4.asp?rs=&t=73065408&ID=20516602759) | [21-05-31 AU](https://www.english-corpora.org/corona/x4.asp?rs=&t=73065408&ID=20516602759) | [honey.nine.com.au](https://www.english-corpora.org/corona/x3.asp?node=&p=17&w10=coronavirus&w11=vaccination&r=) |  |  |  | Duke and Duchess of Cambridge will be by her side, having received the **coronavirus** **vaccination** and shared photos on social media to encourages others to receive their jabs. |
| [1648](https://www.english-corpora.org/corona/x4.asp?rs=&t=73065580&ID=20520053963) | [21-05-31 AU](https://www.english-corpora.org/corona/x4.asp?rs=&t=73065580&ID=20520053963) | [bendigoadvertiser.com.au](https://www.english-corpora.org/corona/x3.asp?node=&p=17&w10=coronavirus&w11=vaccination&r=) |  |  |  | review a decision not to make **coronavirus** **vaccination** mandatory for aged care workers after staff in Melbourne contracted the deadly disease. Victoria |
| [1649](https://www.english-corpora.org/corona/x4.asp?rs=&t=32867685&ID=20491698251) | [21-05-28 NZ](https://www.english-corpora.org/corona/x4.asp?rs=&t=32867685&ID=20491698251) | [theinformant.co.nz](https://www.english-corpora.org/corona/x3.asp?node=&p=17&w10=coronavirus&w11=vaccination&r=) |  |  |  | the conservative federal government of being behind the shutdown, in part because of **Coronavirus** **vaccination** campaign slow. If more people were vaccinated, the conditions would be very different |
| [1650](https://www.english-corpora.org/corona/x4.asp?rs=&t=73064674&ID=20511929960) | [21-05-30 IN](https://www.english-corpora.org/corona/x4.asp?rs=&t=73064674&ID=20511929960) | [timesnownews.com](https://www.english-corpora.org/corona/x3.asp?node=&p=17&w10=coronavirus&w11=vaccination&r=) |  |  |  | terms of fighting with the COVID pandemic. As the third phase of the **coronavirus** **vaccination** drive was kickstarted on May 1st of this year, which aims to vaccinate all |
| [1651](https://www.english-corpora.org/corona/x4.asp?rs=&t=43056289&ID=20510671875) | [21-05-30 PK](https://www.english-corpora.org/corona/x4.asp?rs=&t=43056289&ID=20510671875) | [paktribune.com](https://www.english-corpora.org/corona/x3.asp?node=&p=17&w10=coronavirus&w11=vaccination&r=) |  |  |  | and Green' campaign. # In addition, Barrister Murtaza Wahab inaugurated a **coronavirus** **vaccination** centre at Karachi University. He was accompanied by Dr Iraqi, PPP MPA Sadia |
| [1652](https://www.english-corpora.org/corona/x4.asp?rs=&t=43058659&ID=20517336525) | [21-05-31 SG](https://www.english-corpora.org/corona/x4.asp?rs=&t=43058659&ID=20517336525) | [nationnews.com](https://www.english-corpora.org/corona/x3.asp?node=&p=17&w10=coronavirus&w11=vaccination&r=) |  |  |  | Condividi # London -- A record 598 389 people were given their first **coronavirus** **vaccination** in one day across the United Kingdom on Saturday, government figures show. As |
| [1654](https://www.english-corpora.org/corona/x4.asp?rs=&t=62965924&ID=20489751513) | [21-05-28 ZA](https://www.english-corpora.org/corona/x4.asp?rs=&t=62965924&ID=20489751513) | [citizen.co.za](https://www.english-corpora.org/corona/x3.asp?node=&p=17&w10=coronavirus&w11=vaccination&r=) [(1)](https://www.english-corpora.org/corona/duplicates1.asp?xx=1653&n=1) |  |  |  | are being jabbed with needles rather than vampire fangs this weekend in a **coronavirus** **vaccination** drive. # The government has turned to local vaccination drives and 24-hour " marathons |
| [1655](https://www.english-corpora.org/corona/x4.asp?rs=&t=32883415&ID=20534957524) | [21-06-02 US](https://www.english-corpora.org/corona/x4.asp?rs=&t=32883415&ID=20534957524) | [jpost.com](https://www.english-corpora.org/corona/x3.asp?node=&p=17&w10=coronavirus&w11=vaccination&r=) |  |  |  | . # (photo credit: REUTERS/MASSIMO PINCA/FILE PHOTO) # Advertisement # The **coronavirus** **vaccination** for children aged 12-15 is set to begin next week, the Health Ministry announced |
| [1656](https://www.english-corpora.org/corona/x4.asp?rs=&t=32883471&ID=20540349585) | [21-06-02 US](https://www.english-corpora.org/corona/x4.asp?rs=&t=32883471&ID=20540349585) | [bostonherald.com](https://www.english-corpora.org/corona/x3.asp?node=&p=17&w10=coronavirus&w11=vaccination&r=) |  |  |  | , adding that barbers and stylists are key advocates in the community. # **Coronavirus** **vaccination** rates across the country have increased as cases and deaths have declined significantly. # |
| [1657](https://www.english-corpora.org/corona/x4.asp?rs=&t=32883471&ID=20540349180) | [21-06-02 US](https://www.english-corpora.org/corona/x4.asp?rs=&t=32883471&ID=20540349180) | [bostonherald.com](https://www.english-corpora.org/corona/x3.asp?node=&p=17&w10=coronavirus&w11=vaccination&r=) |  |  |  | hitting the gas on **coronavirus** **vaccination** with prizes and incentives in an effort to achieve his long-desired goal of declaring independence |
| [1658](https://www.english-corpora.org/corona/x4.asp?rs=&t=52786333&ID=20524727545) | [21-06-01 US](https://www.english-corpora.org/corona/x4.asp?rs=&t=52786333&ID=20524727545) | [bangordailynews.com](https://www.english-corpora.org/corona/x3.asp?node=&p=17&w10=coronavirus&w11=vaccination&r=) |  |  |  | Robert F. Bukaty / AP # Maine has one of the highest rates of **coronavirus** **vaccination** in the country, but some of the rural counties in the state lag behind |
| [1659](https://www.english-corpora.org/corona/x4.asp?rs=&t=52786724&ID=20524785761) | [21-06-01 US](https://www.english-corpora.org/corona/x4.asp?rs=&t=52786724&ID=20524785761) | [fox40.com](https://www.english-corpora.org/corona/x3.asp?node=&p=17&w10=coronavirus&w11=vaccination&r=) |  |  |  | front of the Olympic Rings, in Tokyo. Japan, seriously behind in **coronavirus** **vaccination** efforts, is scrambling to boost daily shots as the start of the Olympics in |
| [1660](https://www.english-corpora.org/corona/x4.asp?rs=&t=52791132&ID=20536359489) | [21-06-02 US](https://www.english-corpora.org/corona/x4.asp?rs=&t=52791132&ID=20536359489) | [bangordailynews.com](https://www.english-corpora.org/corona/x3.asp?node=&p=17&w10=coronavirus&w11=vaccination&r=) |  |  |  | 52791132 # PORTLAND, Maine -- Maine has one of the highest rates of **coronavirus** **vaccination** in the country, but some of the rural counties in the state lag behind |
| [1661](https://www.english-corpora.org/corona/x4.asp?rs=&t=87329435&ID=20532776854) | [21-06-01 US](https://www.english-corpora.org/corona/x4.asp?rs=&t=87329435&ID=20532776854) | [USA Today](https://www.english-corpora.org/corona/x3.asp?node=&p=17&w10=coronavirus&w11=vaccination&r=) |  |  |  | are fully approved before getting vaccinated. # ? Indiana University has modified its **coronavirus** **vaccination** requirement, making it optional that students and employees provide proof of getting the shots |
| [1662](https://www.english-corpora.org/corona/x4.asp?rs=&t=87334248&ID=20545086434) | [21-06-02 US](https://www.english-corpora.org/corona/x4.asp?rs=&t=87334248&ID=20545086434) | [NBC Chicago](https://www.english-corpora.org/corona/x3.asp?node=&p=17&w10=coronavirus&w11=vaccination&r=) |  |  |  | . # Arwady noted that such precautions are especially important for people heading to **coronavirus** **vaccination** sites. # " I don't want you coming to a COVID vaccination site |
| [1663](https://www.english-corpora.org/corona/x4.asp?rs=&t=87334837&ID=20544855507) | [21-06-02 US](https://www.english-corpora.org/corona/x4.asp?rs=&t=87334837&ID=20544855507) | [The Baltimore Sun](https://www.english-corpora.org/corona/x3.asp?node=&p=17&w10=coronavirus&w11=vaccination&r=) |  |  |  | beginning to wind down some of the mass **coronavirus** **vaccination** sites, beginning with the M&T Bank Stadium in Baltimore and Ripken Stadium in Aberdeen |
| [1664](https://www.english-corpora.org/corona/x4.asp?rs=&t=87336218&ID=20543928043) | [21-06-02 US](https://www.english-corpora.org/corona/x4.asp?rs=&t=87336218&ID=20543928043) | [New York Times](https://www.english-corpora.org/corona/x3.asp?node=&p=17&w10=coronavirus&w11=vaccination&r=) |  |  |  | 's goal of having 70 percent of the adult population get at least one **coronavirus** **vaccination** by July 4. So far, 63 percent of adult Americans have received at |
| [1665](https://www.english-corpora.org/corona/x4.asp?rs=&t=87338441&ID=20544266129) | [21-06-02 US](https://www.english-corpora.org/corona/x4.asp?rs=&t=87338441&ID=20544266129) | [WGAL](https://www.english-corpora.org/corona/x3.asp?node=&p=17&w10=coronavirus&w11=vaccination&r=) |  |  |  | can have in children? Number of Pennsylvanians vaccinatedAs of May 20, 9,969,849 **coronavirus** **vaccination** doses have been administered in the commonwealth. The number of people who have been |
| [1667](https://www.english-corpora.org/corona/x4.asp?rs=&t=87338668&ID=20558126671) | [21-06-03 US](https://www.english-corpora.org/corona/x4.asp?rs=&t=87338668&ID=20558126671) | [The Hill](https://www.english-corpora.org/corona/x3.asp?node=&p=17&w10=coronavirus&w11=vaccination&r=) [(1)](https://www.english-corpora.org/corona/duplicates1.asp?xx=1666&n=1) |  |  |  | . # But amid a new wave of coronavirus infections and a lagging **coronavirus** **vaccination** rate, there has been growing pressure from inside and outside of Japan to cancel |
| [1668](https://www.english-corpora.org/corona/x4.asp?rs=&t=52787759&ID=20538584187) | [21-06-02 CA](https://www.english-corpora.org/corona/x4.asp?rs=&t=52787759&ID=20538584187) | [jamaica-gleaner.com](https://www.english-corpora.org/corona/x3.asp?node=&p=17&w10=coronavirus&w11=vaccination&r=) |  |  |  | venue for local residents over 65 years old. Japan, seriously behind in **coronavirus** **vaccination** efforts, is scrambling to boost daily shots as the start of the Olympics in |
| [1669](https://www.english-corpora.org/corona/x4.asp?rs=&t=43060948&ID=20526596102) | [21-06-01 GB](https://www.english-corpora.org/corona/x4.asp?rs=&t=43060948&ID=20526596102) | [shropshirestar.com](https://www.english-corpora.org/corona/x3.asp?node=&p=17&w10=coronavirus&w11=vaccination&r=) |  |  |  | " a small number of staff who are " hesitant " about receiving a **coronavirus** **vaccination**, a board report says. # Subscribe to our daily newsletter! # Shropshire |
| [1670](https://www.english-corpora.org/corona/x4.asp?rs=&t=43063268&ID=20540751962) | [21-06-02 GB](https://www.english-corpora.org/corona/x4.asp?rs=&t=43063268&ID=20540751962) | [walesonline.co.uk](https://www.english-corpora.org/corona/x3.asp?node=&p=17&w10=coronavirus&w11=vaccination&r=) |  |  |  | of people in their 30s in England have received a **coronavirus** **vaccination** in a period of little over two weeks, new figures reveal. # NHS |
| [1671](https://www.english-corpora.org/corona/x4.asp?rs=&t=43063274&ID=20540754183) | [21-06-02 GB](https://www.english-corpora.org/corona/x4.asp?rs=&t=43063274&ID=20540754183) | [chroniclelive.co.uk](https://www.english-corpora.org/corona/x3.asp?node=&p=17&w10=coronavirus&w11=vaccination&r=) |  |  |  | . # The Government is aiming to offer everyone aged over 50 their second **coronavirus** **vaccination** by June 21. # It will also consider whether to vaccinate teenagers to boost |
| [1672](https://www.english-corpora.org/corona/x4.asp?rs=&t=43063477&ID=20540776526) | [21-06-02 GB](https://www.english-corpora.org/corona/x4.asp?rs=&t=43063477&ID=20540776526) | [walesonline.co.uk](https://www.english-corpora.org/corona/x3.asp?node=&p=17&w10=coronavirus&w11=vaccination&r=) |  |  |  | third of all adults in Gwent have received both doses of the **coronavirus** **vaccination**, according to latest figures. # Aneurin Bevan University Health Board, which covers |
| [1673](https://www.english-corpora.org/corona/x4.asp?rs=&t=43067413&ID=20547171919) | [21-06-03 GB](https://www.english-corpora.org/corona/x4.asp?rs=&t=43067413&ID=20547171919) | [yorkshirepost.co.uk](https://www.english-corpora.org/corona/x3.asp?node=&p=17&w10=coronavirus&w11=vaccination&r=) |  |  |  | spread of the new variant. # Staff and volunteers at work at a **coronavirus** **vaccination** centre at Twickenham rugby stadium, south-west London, where up to 15,000 doses are |
| [1674](https://www.english-corpora.org/corona/x4.asp?rs=&t=43067761&ID=20549846339) | [21-06-03 GB](https://www.english-corpora.org/corona/x4.asp?rs=&t=43067761&ID=20549846339) | [inews.co.uk](https://www.english-corpora.org/corona/x3.asp?node=&p=17&w10=coronavirus&w11=vaccination&r=) |  |  |  | # More than half of people in their 30s in England have received a **coronavirus** **vaccination** in a period of little over two weeks. # The minister defended his colleague |
| [1675](https://www.english-corpora.org/corona/x4.asp?rs=&t=52795815&ID=20548150035) | [21-06-03 GB](https://www.english-corpora.org/corona/x4.asp?rs=&t=52795815&ID=20548150035) | [news.sky.com](https://www.english-corpora.org/corona/x3.asp?node=&p=17&w10=coronavirus&w11=vaccination&r=) |  |  |  | scientists, the UK medicines regulator the MHRA and the vaccines taskforce for the **coronavirus** **vaccination** rollout. # More than 65.6 million vaccines have now been administered across the UK |
| [1676](https://www.english-corpora.org/corona/x4.asp?rs=&t=62979042&ID=20527571045) | [21-06-01 GB](https://www.english-corpora.org/corona/x4.asp?rs=&t=62979042&ID=20527571045) | [thewestmorlandgazette.co.uk](https://www.english-corpora.org/corona/x3.asp?node=&p=17&w10=coronavirus&w11=vaccination&r=) |  |  |  | showed more than half of people in their 30s in England have received a **coronavirus** **vaccination** in a period of little over two weeks. # (PA Graphics) # |
| [1677](https://www.english-corpora.org/corona/x4.asp?rs=&t=62980888&ID=20541960526) | [21-06-02 IE](https://www.english-corpora.org/corona/x4.asp?rs=&t=62980888&ID=20541960526) | [intellinews.com](https://www.english-corpora.org/corona/x3.asp?node=&p=17&w10=coronavirus&w11=vaccination&r=) |  |  |  | five figures for the first time since December. # In terms of its **coronavirus** **vaccination** programme, Iran has vaccinated roughly 80% of its medical staff dealing with COVID-19. |
| [1678](https://www.english-corpora.org/corona/x4.asp?rs=&t=73077413&ID=20548832094) | [21-06-03 AU](https://www.english-corpora.org/corona/x4.asp?rs=&t=73077413&ID=20548832094) | [themandarin.com.au](https://www.english-corpora.org/corona/x3.asp?node=&p=17&w10=coronavirus&w11=vaccination&r=) |  |  |  | imposed health restrictions' # People line up to be vaccinated at a mass **coronavirus** **vaccination** hub at the Melbourne Convention and Exhibition Centre in Melbourne. (AAP Image/Luis Ascui |
| [1679](https://www.english-corpora.org/corona/x4.asp?rs=&t=73079543&ID=20551781226) | [21-06-03 AU](https://www.english-corpora.org/corona/x4.asp?rs=&t=73079543&ID=20551781226) | [examiner.com.au](https://www.english-corpora.org/corona/x3.asp?node=&p=17&w10=coronavirus&w11=vaccination&r=) |  |  |  | review a decision not to make **coronavirus** **vaccination** mandatory for aged care workers after staff in Melbourne contracted the deadly disease. Victoria |
| [1680](https://www.english-corpora.org/corona/x4.asp?rs=&t=73072938&ID=20528434862) | [21-06-01 IN](https://www.english-corpora.org/corona/x4.asp?rs=&t=73072938&ID=20528434862) | [ibtimes.co.in](https://www.english-corpora.org/corona/x3.asp?node=&p=17&w10=coronavirus&w11=vaccination&r=) |  |  |  | newborn baby apparently had the antibodies in his body, as his mother received **coronavirus** **vaccination** in the third trimester of her pregnancy. # Medical experts who analyzed this case |
| [1681](https://www.english-corpora.org/corona/x4.asp?rs=&t=73084788&ID=20572412734) | [21-06-05 IN](https://www.english-corpora.org/corona/x4.asp?rs=&t=73084788&ID=20572412734) | [ibtimes.co.in](https://www.english-corpora.org/corona/x3.asp?node=&p=17&w10=coronavirus&w11=vaccination&r=) |  |  |  | 3.0 Mission' Covid Suraksha' campaign. # Given the growing demand for **coronavirus** **vaccination**, a pharma company in Maharashtra has stepped forward to prepare for mass production of |
| [1682](https://www.english-corpora.org/corona/x4.asp?rs=&t=43074998&ID=20562527430) | [21-06-04 PK](https://www.english-corpora.org/corona/x4.asp?rs=&t=43074998&ID=20562527430) | [paktribune.com](https://www.english-corpora.org/corona/x3.asp?node=&p=17&w10=coronavirus&w11=vaccination&r=) |  |  |  | Sindh Health Department would contact the persons who had received the first dose of **coronavirus** **vaccination** in order to ask them to complete the vaccination process. # She claimed that |
| [1683](https://www.english-corpora.org/corona/x4.asp?rs=&t=43074998&ID=20562527332) | [21-06-04 PK](https://www.english-corpora.org/corona/x4.asp?rs=&t=43074998&ID=20562527332) | [paktribune.com](https://www.english-corpora.org/corona/x3.asp?node=&p=17&w10=coronavirus&w11=vaccination&r=) |  |  |  | vaccination of students of religious seminaries and this stage would come with commencement of **coronavirus** **vaccination** of people below 30 years of age since the seminary students were mostly of younger |
| [1684](https://www.english-corpora.org/corona/x4.asp?rs=&t=43074998&ID=20562527317) | [21-06-04 PK](https://www.english-corpora.org/corona/x4.asp?rs=&t=43074998&ID=20562527317) | [paktribune.com](https://www.english-corpora.org/corona/x3.asp?node=&p=17&w10=coronavirus&w11=vaccination&r=) |  |  |  | , " she said. # She said the Sindh government intended to do **coronavirus** **vaccination** of students of religious seminaries and this stage would come with commencement of coronavirus vaccination |
| [1685](https://www.english-corpora.org/corona/x4.asp?rs=&t=52789822&ID=20536216050) | [21-06-02 PK](https://www.english-corpora.org/corona/x4.asp?rs=&t=52789822&ID=20536216050) | [samaa.tv](https://www.english-corpora.org/corona/x3.asp?node=&p=17&w10=coronavirus&w11=vaccination&r=) |  |  |  | vaccines were transported through a special plane of the Pakistan International Airlines. # **Coronavirus** **vaccination** of Pakistanis above 18 years will start from June 3. # On May 29 |
| [1686](https://www.english-corpora.org/corona/x4.asp?rs=&t=52789822&ID=20536215907) | [21-06-02 PK](https://www.english-corpora.org/corona/x4.asp?rs=&t=52789822&ID=20536215907) | [samaa.tv](https://www.english-corpora.org/corona/x3.asp?node=&p=17&w10=coronavirus&w11=vaccination&r=) |  |  |  | , said that over 5.2 million people have been vaccinated across the country. **Coronavirus** **vaccination** of Pakistanis above 18 years will start from June 3. On May 29, |
| [1687](https://www.english-corpora.org/corona/x4.asp?rs=&t=52790232&ID=20536271916) | [21-06-02 PK](https://www.english-corpora.org/corona/x4.asp?rs=&t=52790232&ID=20536271916) | [suchtv.pk](https://www.english-corpora.org/corona/x3.asp?node=&p=17&w10=coronavirus&w11=vaccination&r=) |  |  |  | launch major campaign for **coronavirus** **vaccination**: Asad # File photoThe head of the National Command and Operation Centre (NCOC |
| [1688](https://www.english-corpora.org/corona/x4.asp?rs=&t=52790330&ID=20536277087) | [21-06-02 PK](https://www.english-corpora.org/corona/x4.asp?rs=&t=52790330&ID=20536277087) | [samaa.tv](https://www.english-corpora.org/corona/x3.asp?node=&p=17&w10=coronavirus&w11=vaccination&r=) |  |  |  | Over 5.2 million people have been inoculated so far. # Pakistan will begin **coronavirus** **vaccination** of people above 18 years from June 3. In the last 24 hours, |
| [1689](https://www.english-corpora.org/corona/x4.asp?rs=&t=52790330&ID=20536276904) | [21-06-02 PK](https://www.english-corpora.org/corona/x4.asp?rs=&t=52790330&ID=20536276904) | [samaa.tv](https://www.english-corpora.org/corona/x3.asp?node=&p=17&w10=coronavirus&w11=vaccination&r=) |  |  |  | . Over 5.2 million people have been inoculated so far. Pakistan will begin **coronavirus** **vaccination** of people above 18 years from June 3. In the last 24 hours, |
| [1690](https://www.english-corpora.org/corona/x4.asp?rs=&t=52792270&ID=20541827210) | [21-06-02 PK](https://www.english-corpora.org/corona/x4.asp?rs=&t=52792270&ID=20541827210) | [dnd.com.pk](https://www.english-corpora.org/corona/x3.asp?node=&p=17&w10=coronavirus&w11=vaccination&r=) |  |  |  | the contents of the joint statement and said encouraging the masses for using the **Coronavirus** **vaccination** was the duty of Ulema and Mashaikh. # President Arif Alvi said that vaccination |
| [1691](https://www.english-corpora.org/corona/x4.asp?rs=&t=52794584&ID=20553388599) | [21-06-03 PK](https://www.english-corpora.org/corona/x4.asp?rs=&t=52794584&ID=20553388599) | [suchtv.pk](https://www.english-corpora.org/corona/x3.asp?node=&p=17&w10=coronavirus&w11=vaccination&r=) |  |  |  | unvaccinated Sindh government employees should not be released if they did not get the **coronavirus** **vaccination** and issued directives to the finance ministry in this regard. # According to the |
| [1692](https://www.english-corpora.org/corona/x4.asp?rs=&t=52794877&ID=20553419911) | [21-06-03 PK](https://www.english-corpora.org/corona/x4.asp?rs=&t=52794877&ID=20553419911) | [dailytimes.com.pk](https://www.english-corpora.org/corona/x3.asp?node=&p=17&w10=coronavirus&w11=vaccination&r=) |  |  |  | get anti-COVID jabs in 24 hours # RAWALPINDI: The total district tally of **coronavirus** **vaccination** reached 397,397 on Thursday, with the inoculation of 12,824 more persons against the deadly |
| [1693](https://www.english-corpora.org/corona/x4.asp?rs=&t=52794877&ID=20553419962) | [21-06-03 PK](https://www.english-corpora.org/corona/x4.asp?rs=&t=52794877&ID=20553419962) | [dailytimes.com.pk](https://www.english-corpora.org/corona/x3.asp?node=&p=17&w10=coronavirus&w11=vaccination&r=) |  |  |  | health workers while 371,199 people including senior citizens have received their doses of the **coronavirus** **vaccination** so far. # The report said that the district health authority has registered 33 |
| [1694](https://www.english-corpora.org/corona/x4.asp?rs=&t=52797844&ID=20561054846) | [21-06-04 PK](https://www.english-corpora.org/corona/x4.asp?rs=&t=52797844&ID=20561054846) | [en.dailypakistan.com.pk](https://www.english-corpora.org/corona/x3.asp?node=&p=17&w10=coronavirus&w11=vaccination&r=) |  |  |  | whole of the nation approach' Share # ISLAMABAD -- Pakistan has started **coronavirus** **vaccination** for people over the age of 18 years. # More than five million people |
| [1695](https://www.english-corpora.org/corona/x4.asp?rs=&t=52798074&ID=20562858575) | [21-06-04 PK](https://www.english-corpora.org/corona/x4.asp?rs=&t=52798074&ID=20562858575) | [en.dailypakistan.com.pk](https://www.english-corpora.org/corona/x3.asp?node=&p=17&w10=coronavirus&w11=vaccination&r=) |  |  |  | 926,695. The positive rate stands at 3.93 percent. # Pakistan has started **coronavirus** **vaccination** for those who are over 18 years old. # Earlier this week, National |
| [1696](https://www.english-corpora.org/corona/x4.asp?rs=&t=73070544&ID=20528106628) | [21-06-01 PH](https://www.english-corpora.org/corona/x4.asp?rs=&t=73070544&ID=20528106628) | [news.abs-cbn.com](https://www.english-corpora.org/corona/x3.asp?node=&p=17&w10=coronavirus&w11=vaccination&r=) |  |  |  | ? # South Korean authorities said on Sunday they will move ahead with a **coronavirus** **vaccination** drive this week, after deciding to continue using AstraZeneca PLC's vaccine for all |
| [1697](https://www.english-corpora.org/corona/x4.asp?rs=&t=43069191&ID=20552725690) | [21-06-03 HK](https://www.english-corpora.org/corona/x4.asp?rs=&t=43069191&ID=20552725690) | [hongkongfp.com](https://www.english-corpora.org/corona/x3.asp?node=&p=17&w10=coronavirus&w11=vaccination&r=) |  |  |  | programme to children as young as 12 # Hong Kong will expand its BioNTech **coronavirus** **vaccination** programme to children as young as 12, reducing the age limit from 16, |
| [1698](https://www.english-corpora.org/corona/x4.asp?rs=&t=43078673&ID=20580562008) | [21-06-06 HK](https://www.english-corpora.org/corona/x4.asp?rs=&t=43078673&ID=20580562008) | [thestandard.com.hk](https://www.english-corpora.org/corona/x3.asp?node=&p=17&w10=coronavirus&w11=vaccination&r=) |  |  |  | that looks into adverse clinical events following **coronavirus** **vaccination** says it will no longer make public announcements every time a recently inoculated person dies |
| [1699](https://www.english-corpora.org/corona/x4.asp?rs=&t=52785257&ID=20527009425) | [21-06-01 NG](https://www.english-corpora.org/corona/x4.asp?rs=&t=52785257&ID=20527009425) | [legit.ng](https://www.english-corpora.org/corona/x3.asp?node=&p=17&w10=coronavirus&w11=vaccination&r=) |  |  |  | - The federal government on Tuesday, June 1, announced that some special **coronavirus** **vaccination** sites have been set up across the country to ensure that every Nigerian who had |
| [1700](https://www.english-corpora.org/corona/x4.asp?rs=&t=52785476&ID=20529432522) | [21-06-01 NG](https://www.english-corpora.org/corona/x4.asp?rs=&t=52785476&ID=20529432522) | [channelstv.com](https://www.english-corpora.org/corona/x3.asp?node=&p=17&w10=coronavirus&w11=vaccination&r=) |  |  |  | our gradual return to our normal lives with the commencement of the second dose **coronavirus** **vaccination**. With this, we hope for brighter days ahead as we collectively fight to |

| PAGE:   [**<<**](https://www.english-corpora.org/corona/x3.asp?node=&p=1&w10=coronavirus&w11=vaccination&r=)  [**<**](https://www.english-corpora.org/corona/x3.asp?node=&p=16&w10=coronavirus&w11=vaccination&r=)   17 / 42  [**>**](https://www.english-corpora.org/corona/x3.asp?node=&p=18&w10=coronavirus&w11=vaccination&r=)   [**>>**](https://www.english-corpora.org/corona/x3.asp?node=&p=42&w10=coronavirus&w11=vaccination&r=) | | |
| --- | --- | --- |
| PAGE:   [**<<**](https://www.english-corpora.org/corona/x3.asp?node=&p=1&w10=coronavirus&w11=vaccination&r=)  [**<**](https://www.english-corpora.org/corona/x3.asp?node=&p=17&w10=coronavirus&w11=vaccination&r=)   18 / 42  [**>**](https://www.english-corpora.org/corona/x3.asp?node=&p=19&w10=coronavirus&w11=vaccination&r=)   [**>>**](https://www.english-corpora.org/corona/x3.asp?node=&p=42&w10=coronavirus&w11=vaccination&r=) |  |  |

Начало формы

| CLICK FOR MORE CONTEXT | [**HELP**](javascript:newFeatures()) | [SAVE](javascript:chooser('s'))    [TRANSLATE](javascript:chooser('t'))    [ANALYZE](javascript:chooser('p')) |
| --- | --- | --- |

| [1701](https://www.english-corpora.org/corona/x4.asp?rs=&t=32839031&ID=20369136976) | [21-05-16 US](https://www.english-corpora.org/corona/x4.asp?rs=&t=32839031&ID=20369136976) | [courant.com](https://www.english-corpora.org/corona/x3.asp?node=&p=18&w10=coronavirus&w11=vaccination&r=) |  |  |  | and hang out. " # A life-size blue whale display hanging above the **coronavirus** **vaccination** site at the American Museum of Natural History in New York sports a bandage above |
| --- | --- | --- | --- | --- | --- | --- |
| [1702](https://www.english-corpora.org/corona/x4.asp?rs=&t=32846792&ID=20404536403) | [21-05-19 US](https://www.english-corpora.org/corona/x4.asp?rs=&t=32846792&ID=20404536403) | [registerguard.com](https://www.english-corpora.org/corona/x3.asp?node=&p=18&w10=coronavirus&w11=vaccination&r=) |  |  |  | He had underlying conditions. Rural Americans less likely to be vaccinated # **Coronavirus** **vaccination** rates across the nation are lower in rural counties than in urban ones in both |
| [1703](https://www.english-corpora.org/corona/x4.asp?rs=&t=32848794&ID=20415109205) | [21-05-20 US](https://www.english-corpora.org/corona/x4.asp?rs=&t=32848794&ID=20415109205) | [nbcchicago.com](https://www.english-corpora.org/corona/x3.asp?node=&p=18&w10=coronavirus&w11=vaccination&r=) |  |  |  | . # Arwady noted that such precautions are especially important for people heading to **coronavirus** **vaccination** sites. # " I don't want you coming to a COVID vaccination site |
| [1704](https://www.english-corpora.org/corona/x4.asp?rs=&t=32849819&ID=20412448587) | [21-05-20 US](https://www.english-corpora.org/corona/x4.asp?rs=&t=32849819&ID=20412448587) | [pilotonline.com](https://www.english-corpora.org/corona/x3.asp?node=&p=18&w10=coronavirus&w11=vaccination&r=) |  |  |  | , will keep us going in the right direction. # Virginia's average **coronavirus** **vaccination** rate has slowed way down over the past month. In mid-April, the state |
| [1705](https://www.english-corpora.org/corona/x4.asp?rs=&t=32857174&ID=20445120402) | [21-05-24 US](https://www.english-corpora.org/corona/x4.asp?rs=&t=32857174&ID=20445120402) | [chron.com](https://www.english-corpora.org/corona/x3.asp?node=&p=18&w10=coronavirus&w11=vaccination&r=) |  |  |  | passports as Alabama becomes the latest state to try to prohibit proof of a **coronavirus** **vaccination** to enter a business, school or event. # The legislation by Republican Sen. |
| [1708](https://www.english-corpora.org/corona/x4.asp?rs=&t=62940394&ID=20405596503) | [21-05-19 US](https://www.english-corpora.org/corona/x4.asp?rs=&t=62940394&ID=20405596503) | [tapinto.net](https://www.english-corpora.org/corona/x3.asp?node=&p=18&w10=coronavirus&w11=vaccination&r=) [(2)](https://www.english-corpora.org/corona/duplicates1.asp?xx=1707&n=2) |  |  |  | , 2021 at 1:04 PM # FREEHOLD, NJ -- A Monmouth County **coronavirus** **vaccination** clinic for 12- to 18-year-olds will be held on Wednesday, May 19 at Brookdale |
| [1709](https://www.english-corpora.org/corona/x4.asp?rs=&t=73030311&ID=20413478969) | [21-05-20 US](https://www.english-corpora.org/corona/x4.asp?rs=&t=73030311&ID=20413478969) | [fox13now.com](https://www.english-corpora.org/corona/x3.asp?node=&p=18&w10=coronavirus&w11=vaccination&r=) |  |  |  | cases, four additional deaths # Middle school student Elise Robinson receives her first **coronavirus** **vaccination** on Wednesday, May 12, 2021 # Posted at 12:49 PM, May 20 |
| [1710](https://www.english-corpora.org/corona/x4.asp?rs=&t=73047284&ID=20459400122) | [21-05-25 US](https://www.english-corpora.org/corona/x4.asp?rs=&t=73047284&ID=20459400122) | [nbcwashington.com](https://www.english-corpora.org/corona/x3.asp?node=&p=18&w10=coronavirus&w11=vaccination&r=) |  |  |  | News is making finding information on when, how and where to obtain your **coronavirus** **vaccination** easier with its Plan Your Vaccine website. Key Charts and Graphs # The |
| [1711](https://www.english-corpora.org/corona/x4.asp?rs=&t=87240981&ID=20375259798) | [21-05-16 US](https://www.english-corpora.org/corona/x4.asp?rs=&t=87240981&ID=20375259798) | [Washington Post](https://www.english-corpora.org/corona/x3.asp?node=&p=18&w10=coronavirus&w11=vaccination&r=) |  |  |  | Ferebee said the school system is working with health officials to use schools as **coronavirus** **vaccination** sites, but he did not say how or when that would happen. The |
| [1712](https://www.english-corpora.org/corona/x4.asp?rs=&t=87240981&ID=20375259931) | [21-05-16 US](https://www.english-corpora.org/corona/x4.asp?rs=&t=87240981&ID=20375259931) | [Washington Post](https://www.english-corpora.org/corona/x3.asp?node=&p=18&w10=coronavirus&w11=vaccination&r=) |  |  |  | city leaders and activists thinking about how to educate students and their families about **coronavirus** **vaccination**. # " It's hard to crack, " said Lane, who also |
| [1713](https://www.english-corpora.org/corona/x4.asp?rs=&t=87240981&ID=20375260279) | [21-05-16 US](https://www.english-corpora.org/corona/x4.asp?rs=&t=87240981&ID=20375260279) | [Washington Post](https://www.english-corpora.org/corona/x3.asp?node=&p=18&w10=coronavirus&w11=vaccination&r=) |  |  |  | Lane said city and health advocates need to decide whether to direct messages about **coronavirus** **vaccination** to children, to families, or a combination. # Story continues below advertisement |
| [1714](https://www.english-corpora.org/corona/x4.asp?rs=&t=87243553&ID=20386684788) | [21-05-17 US](https://www.english-corpora.org/corona/x4.asp?rs=&t=87243553&ID=20386684788) | [The National Interest](https://www.english-corpora.org/corona/x3.asp?node=&p=18&w10=coronavirus&w11=vaccination&r=) |  |  |  | Now could be the time for America to play a greater role in global **coronavirus** **vaccination**, both out of generosity and self-interest. # As more coronavirus vaccine shots are |
| [1715](https://www.english-corpora.org/corona/x4.asp?rs=&t=87244759&ID=20387773293) | [21-05-17 US](https://www.english-corpora.org/corona/x4.asp?rs=&t=87244759&ID=20387773293) | [The Baltimore Sun](https://www.english-corpora.org/corona/x3.asp?node=&p=18&w10=coronavirus&w11=vaccination&r=) |  |  |  | Maryland's health secretary warned Monday that the next phase of the state's **coronavirus** **vaccination** campaign will be costly and time-consuming, as officials work to reach people who have |
| [1716](https://www.english-corpora.org/corona/x4.asp?rs=&t=87249252&ID=20386903470) | [21-05-17 US](https://www.english-corpora.org/corona/x4.asp?rs=&t=87249252&ID=20386903470) | [Washington Post](https://www.english-corpora.org/corona/x3.asp?node=&p=18&w10=coronavirus&w11=vaccination&r=) |  |  |  | spaces in March. Just when India needed it the most, its **coronavirus** **vaccination** drive is faltering # NEW DELHI -- As India confronts a devastating coronavirus outbreak where |
| [1717](https://www.english-corpora.org/corona/x4.asp?rs=&t=87250615&ID=20395312396) | [21-05-18 US](https://www.english-corpora.org/corona/x4.asp?rs=&t=87250615&ID=20395312396) | [Associated Press](https://www.english-corpora.org/corona/x3.asp?node=&p=18&w10=coronavirus&w11=vaccination&r=) |  |  |  | on Tuesday followed CDC guidelines and dropped plans to tie business occupancy to **coronavirus** **vaccination** rates. # The unanimous vote came after public speakers expressed anger and frustration with |
| [1718](https://www.english-corpora.org/corona/x4.asp?rs=&t=87250615&ID=20395312538) | [21-05-18 US](https://www.english-corpora.org/corona/x4.asp?rs=&t=87250615&ID=20395312538) | [Associated Press](https://www.english-corpora.org/corona/x3.asp?node=&p=18&w10=coronavirus&w11=vaccination&r=) |  |  |  | health minister who has faced criticism for the slow pace of the nation's **coronavirus** **vaccination** effort. # Prime Minister Denys Shmyhal held Health Minister Maksym Stepanov responsible for the |
| [1719](https://www.english-corpora.org/corona/x4.asp?rs=&t=87250615&ID=20395313536) | [21-05-18 US](https://www.english-corpora.org/corona/x4.asp?rs=&t=87250615&ID=20395313536) | [Associated Press](https://www.english-corpora.org/corona/x3.asp?node=&p=18&w10=coronavirus&w11=vaccination&r=) |  |  |  | -- A U.S. government report finds rural counties are behind urban counties in their **coronavirus** **vaccination** efforts, a gap that could slow the fight against the coronavirus. # The |
| [1720](https://www.english-corpora.org/corona/x4.asp?rs=&t=87252313&ID=20395021951) | [21-05-18 US](https://www.english-corpora.org/corona/x4.asp?rs=&t=87252313&ID=20395021951) | [USA TODAY on MSN.com](https://www.english-corpora.org/corona/x3.asp?node=&p=18&w10=coronavirus&w11=vaccination&r=) |  |  |  | 37.4% of the population. Rural vaccination rates lower than urban rates # **Coronavirus** **vaccination** rates across the nation are lower in rural counties than in urban ones in both |
| [1722](https://www.english-corpora.org/corona/x4.asp?rs=&t=87253236&ID=20397471521) | [21-05-18 US](https://www.english-corpora.org/corona/x4.asp?rs=&t=87253236&ID=20397471521) | [YAHOO!News](https://www.english-corpora.org/corona/x3.asp?node=&p=18&w10=coronavirus&w11=vaccination&r=) [(1)](https://www.english-corpora.org/corona/duplicates1.asp?xx=1721&n=1) |  |  |  | on Tuesday followed CDC guidelines and dropped plans to tie business occupancy to **coronavirus** **vaccination** rates. Nevada Gov. Steve Sisolak on Friday aligned state requirements with CDC recommendations |
| [1723](https://www.english-corpora.org/corona/x4.asp?rs=&t=87254407&ID=20395830484) | [21-05-18 US](https://www.english-corpora.org/corona/x4.asp?rs=&t=87254407&ID=20395830484) | [SFGate](https://www.english-corpora.org/corona/x3.asp?node=&p=18&w10=coronavirus&w11=vaccination&r=) |  |  |  | lawmakers on Tuesday followed CDC guidelines and dropped plans to tie business occupancy to **coronavirus** **vaccination** rates. # The unanimous vote came after public speakers expressed anger and frustration with |
| [1724](https://www.english-corpora.org/corona/x4.asp?rs=&t=87254407&ID=20395830624) | [21-05-18 US](https://www.english-corpora.org/corona/x4.asp?rs=&t=87254407&ID=20395830624) | [SFGate](https://www.english-corpora.org/corona/x3.asp?node=&p=18&w10=coronavirus&w11=vaccination&r=) |  |  |  | health minister who has faced criticism for the slow pace of the nation's **coronavirus** **vaccination** effort. # Prime Minister Denys Shmyhal held Health Minister Maksym Stepanov responsible for the |
| [1725](https://www.english-corpora.org/corona/x4.asp?rs=&t=87254407&ID=20395831620) | [21-05-18 US](https://www.english-corpora.org/corona/x4.asp?rs=&t=87254407&ID=20395831620) | [SFGate](https://www.english-corpora.org/corona/x3.asp?node=&p=18&w10=coronavirus&w11=vaccination&r=) |  |  |  | -- A U.S. government report finds rural counties are behind urban counties in their **coronavirus** **vaccination** efforts, a gap that could slow the fight against the coronavirus. # The |
| [1726](https://www.english-corpora.org/corona/x4.asp?rs=&t=87254481&ID=20398164597) | [21-05-18 US](https://www.english-corpora.org/corona/x4.asp?rs=&t=87254481&ID=20398164597) | [SFGate](https://www.english-corpora.org/corona/x3.asp?node=&p=18&w10=coronavirus&w11=vaccination&r=) |  |  |  | Monday approved a ban on so-called vaccine passports that would prohibit proof of a **coronavirus** **vaccination** to enter a business, school or event. # The legislation would " prohibit |
| [1727](https://www.english-corpora.org/corona/x4.asp?rs=&t=87255281&ID=20398201775) | [21-05-18 US](https://www.english-corpora.org/corona/x4.asp?rs=&t=87255281&ID=20398201775) | [MarketWatch](https://www.english-corpora.org/corona/x3.asp?node=&p=18&w10=coronavirus&w11=vaccination&r=) |  |  |  | on Tuesday followed CDC guidelines and dropped plans to tie business occupancy to **coronavirus** **vaccination** rates. # The unanimous vote came after public speakers expressed anger and frustration with |
| [1728](https://www.english-corpora.org/corona/x4.asp?rs=&t=87259919&ID=20407698702) | [21-05-19 US](https://www.english-corpora.org/corona/x4.asp?rs=&t=87259919&ID=20407698702) | [New York Post](https://www.english-corpora.org/corona/x3.asp?node=&p=18&w10=coronavirus&w11=vaccination&r=) |  |  |  | Pass is a free, voluntary digital platform for New Yorkers to prove their **coronavirus** **vaccination** status, or recent history of a negative COVID-19 test. # " Similar to |
| [1729](https://www.english-corpora.org/corona/x4.asp?rs=&t=87261295&ID=20411042036) | [21-05-19 US](https://www.english-corpora.org/corona/x4.asp?rs=&t=87261295&ID=20411042036) | [Washington Post](https://www.english-corpora.org/corona/x3.asp?node=&p=18&w10=coronavirus&w11=vaccination&r=) |  |  |  | organization suing on behalf of a group of L.A. public-school teachers says that mandating **coronavirus** **vaccination** before full approval amounts to nonconsensual medical experimentation, and the New Mexico detention center |
| [1730](https://www.english-corpora.org/corona/x4.asp?rs=&t=87264984&ID=20419667236) | [21-05-20 US](https://www.english-corpora.org/corona/x4.asp?rs=&t=87264984&ID=20419667236) | [New York Times](https://www.english-corpora.org/corona/x3.asp?node=&p=18&w10=coronavirus&w11=vaccination&r=) |  |  |  | managed by the Border Patrol earlier this year. # Image # A FEMA-supported **coronavirus** **vaccination** site in Miami. By late April, nearly 3,000 FEMA staff were working on |
| [1731](https://www.english-corpora.org/corona/x4.asp?rs=&t=87264984&ID=20419666222) | [21-05-20 US](https://www.english-corpora.org/corona/x4.asp?rs=&t=87264984&ID=20419666222) | [New York Times](https://www.english-corpora.org/corona/x3.asp?node=&p=18&w10=coronavirus&w11=vaccination&r=) |  |  |  | for the migrant children surging across the Southern border. They've been running **coronavirus** **vaccination** sites in Colorado, Massachusetts and Washington. And they are still managing the recovery |
| [1732](https://www.english-corpora.org/corona/x4.asp?rs=&t=87269392&ID=20419565460) | [21-05-20 US](https://www.english-corpora.org/corona/x4.asp?rs=&t=87269392&ID=20419565460) | [Washington Post](https://www.english-corpora.org/corona/x3.asp?node=&p=18&w10=coronavirus&w11=vaccination&r=) |  |  |  | with bottles, stones and fireworks while celebrating promotion. # With Germany's **coronavirus** **vaccination** campaign well under way, fans may return in larger numbers when the new season |
| [1733](https://www.english-corpora.org/corona/x4.asp?rs=&t=87272678&ID=20428381477) | [21-05-21 US](https://www.english-corpora.org/corona/x4.asp?rs=&t=87272678&ID=20428381477) | [The Hill](https://www.english-corpora.org/corona/x3.asp?node=&p=18&w10=coronavirus&w11=vaccination&r=) |  |  |  | " according to the announcement. # ADVERTISEMENT # Many schools have announced a **coronavirus** **vaccination** requirement. The University of Virginia also announced a vaccine requirement on Friday. # |
| [1734](https://www.english-corpora.org/corona/x4.asp?rs=&t=87275209&ID=20426381194) | [21-05-21 US](https://www.english-corpora.org/corona/x4.asp?rs=&t=87275209&ID=20426381194) | [SFGate](https://www.english-corpora.org/corona/x3.asp?node=&p=18&w10=coronavirus&w11=vaccination&r=) |  |  |  | COVID-19 vaccine, the number of Ohioans age 16 and older who started the **coronavirus** **vaccination** process jumped 33 per cent. However, vaccination rates are still well below where |
| [1736](https://www.english-corpora.org/corona/x4.asp?rs=&t=87278385&ID=20434565265) | [21-05-22 US](https://www.english-corpora.org/corona/x4.asp?rs=&t=87278385&ID=20434565265) | [Washington Post](https://www.english-corpora.org/corona/x3.asp?node=&p=18&w10=coronavirus&w11=vaccination&r=) [(1)](https://www.english-corpora.org/corona/duplicates1.asp?xx=1735&n=1) |  |  |  | Mall on Wednesday call on the United States to commit to a global **coronavirus** **vaccination** plan that includes sharing vaccine formulas with the world. (Saul Loeb/AFP/Getty Images) |
| [1737](https://www.english-corpora.org/corona/x4.asp?rs=&t=87285449&ID=20449734868) | [21-05-24 US](https://www.english-corpora.org/corona/x4.asp?rs=&t=87285449&ID=20449734868) | [New York Times](https://www.english-corpora.org/corona/x3.asp?node=&p=18&w10=coronavirus&w11=vaccination&r=) |  |  |  | said the federal government will not issue a digital system that tracks people's **coronavirus** **vaccination** status. # " The government is not now nor will we be supporting a |
| [1738](https://www.english-corpora.org/corona/x4.asp?rs=&t=87288714&ID=20448376294) | [21-05-24 US](https://www.english-corpora.org/corona/x4.asp?rs=&t=87288714&ID=20448376294) | [The Washington Post on MSN.com](https://www.english-corpora.org/corona/x3.asp?node=&p=18&w10=coronavirus&w11=vaccination&r=) |  |  |  | increase, cases and other metrics keep dropping in D.C. area # **Coronavirus** **vaccination** rates in the D.C. area are rising, and covid-19 metrics continue to fall: |
| [1739](https://www.english-corpora.org/corona/x4.asp?rs=&t=87289918&ID=20460887603) | [21-05-25 US](https://www.english-corpora.org/corona/x4.asp?rs=&t=87289918&ID=20460887603) | [Digital Trends](https://www.english-corpora.org/corona/x3.asp?node=&p=18&w10=coronavirus&w11=vaccination&r=) |  |  |  | features ahead of expected travel rebound # With **coronavirus** **vaccination** programs allowing travel to open up in some parts of the world, Airbnb is |
| [1740](https://www.english-corpora.org/corona/x4.asp?rs=&t=87291707&ID=20460684140) | [21-05-25 US](https://www.english-corpora.org/corona/x4.asp?rs=&t=87291707&ID=20460684140) | [Forbes](https://www.english-corpora.org/corona/x3.asp?node=&p=18&w10=coronavirus&w11=vaccination&r=) |  |  |  | 87291707 # As more of the United States population receives the **coronavirus** **vaccination**, property owners and commercial tenants are wondering whether it's time to throw the |
| [1741](https://www.english-corpora.org/corona/x4.asp?rs=&t=87292949&ID=20462525928) | [21-05-25 US](https://www.english-corpora.org/corona/x4.asp?rs=&t=87292949&ID=20462525928) | [Business Insider](https://www.english-corpora.org/corona/x3.asp?node=&p=18&w10=coronavirus&w11=vaccination&r=) |  |  |  | , the United States is opening back up again as **coronavirus** **vaccination** rates rise. And consumers are shopping accordingly. # Alarm clocks, teeth whiteners |
| [1742](https://www.english-corpora.org/corona/x4.asp?rs=&t=87294061&ID=20461328978) | [21-05-25 US](https://www.english-corpora.org/corona/x4.asp?rs=&t=87294061&ID=20461328978) | [New York Times](https://www.english-corpora.org/corona/x3.asp?node=&p=18&w10=coronavirus&w11=vaccination&r=) |  |  |  | positive. # No vaccine provides perfect protection, and so-called breakthrough infections after **coronavirus** **vaccination** are rare and unlikely to lead to serious illness. Federal health officials have told |
| [1743](https://www.english-corpora.org/corona/x4.asp?rs=&t=87297866&ID=20475820396) | [21-05-26 US](https://www.english-corpora.org/corona/x4.asp?rs=&t=87297866&ID=20475820396) | [ESPN](https://www.english-corpora.org/corona/x3.asp?node=&p=18&w10=coronavirus&w11=vaccination&r=) |  |  |  | push # NEW YORK -- Major League Baseball joined many of its clubs in **coronavirus** **vaccination** efforts, sending retired players Todd Zeile and Yonder Alonso to a New York City |
| [1744](https://www.english-corpora.org/corona/x4.asp?rs=&t=87298401&ID=20473078670) | [21-05-26 US](https://www.english-corpora.org/corona/x4.asp?rs=&t=87298401&ID=20473078670) | [Yahoo](https://www.english-corpora.org/corona/x3.asp?node=&p=18&w10=coronavirus&w11=vaccination&r=) |  |  |  | for essential work, healthcare, grocery shopping, exercise or to get a **coronavirus** **vaccination**. Several infected contacts have visited crowded hot spots that included sports stadiums and one |
| [1746](https://www.english-corpora.org/corona/x4.asp?rs=&t=87298805&ID=20473909508) | [21-05-26 US](https://www.english-corpora.org/corona/x4.asp?rs=&t=87298805&ID=20473909508) | [Washington Times](https://www.english-corpora.org/corona/x3.asp?node=&p=18&w10=coronavirus&w11=vaccination&r=) [(1)](https://www.english-corpora.org/corona/duplicates1.asp?xx=1745&n=1) |  |  |  | # The future freedoms of American citizens depend on the outcome of this **coronavirus** **vaccination** fight. It's all about choice. It's all about the power of |
| [1747](https://www.english-corpora.org/corona/x4.asp?rs=&t=87299781&ID=20476538535) | [21-05-26 US](https://www.english-corpora.org/corona/x4.asp?rs=&t=87299781&ID=20476538535) | [Yahoo](https://www.english-corpora.org/corona/x3.asp?node=&p=18&w10=coronavirus&w11=vaccination&r=) |  |  |  | for essential work, healthcare, grocery shopping, exercise or to get a **coronavirus** **vaccination**. Several infected contacts have visited crowded hot spots that included sports stadiums and one |
| [1750](https://www.english-corpora.org/corona/x4.asp?rs=&t=87300424&ID=20473985972) | [21-05-26 US](https://www.english-corpora.org/corona/x4.asp?rs=&t=87300424&ID=20473985972) | [SFGate](https://www.english-corpora.org/corona/x3.asp?node=&p=18&w10=coronavirus&w11=vaccination&r=) [(2)](https://www.english-corpora.org/corona/duplicates1.asp?xx=1749&n=2) |  |  |  | measure, Gross included multiple references to employers including hospitals requiring proof of **coronavirus** **vaccination** as a condition of employment. # Bill sponsor Rep. Jennifer Gross is a nurse |
| [1751](https://www.english-corpora.org/corona/x4.asp?rs=&t=87301407&ID=20484448423) | [21-05-27 US](https://www.english-corpora.org/corona/x4.asp?rs=&t=87301407&ID=20484448423) | [YAHOO!News](https://www.english-corpora.org/corona/x3.asp?node=&p=18&w10=coronavirus&w11=vaccination&r=) |  |  |  | the traditional start of the summer driving season, as fuel demand surges alongside **coronavirus** **vaccination** rates. Further, some areas of the country, such as Washington, D.C. |
| [1752](https://www.english-corpora.org/corona/x4.asp?rs=&t=87303066&ID=20486165167) | [21-05-27 US](https://www.english-corpora.org/corona/x4.asp?rs=&t=87303066&ID=20486165167) | [Business Insider](https://www.english-corpora.org/corona/x3.asp?node=&p=18&w10=coronavirus&w11=vaccination&r=) |  |  |  | Hub to vaccinate residents in early May. The solution manages a variety of **coronavirus** **vaccination** processes, including: making appointments, verifying a person's identity at the vaccination |
| [1753](https://www.english-corpora.org/corona/x4.asp?rs=&t=87305646&ID=20486329770) | [21-05-27 US](https://www.english-corpora.org/corona/x4.asp?rs=&t=87305646&ID=20486329770) | [Yahoo](https://www.english-corpora.org/corona/x3.asp?node=&p=18&w10=coronavirus&w11=vaccination&r=) |  |  |  | over a third of the overall total. This month, India opened its **coronavirus** **vaccination** campaign to everyone aged 18 or older. # The group behind the SolarWinds cyber |
| [1756](https://www.english-corpora.org/corona/x4.asp?rs=&t=87306533&ID=20484058189) | [21-05-27 US](https://www.english-corpora.org/corona/x4.asp?rs=&t=87306533&ID=20484058189) | [Washington Post](https://www.english-corpora.org/corona/x3.asp?node=&p=18&w10=coronavirus&w11=vaccination&r=) [(2)](https://www.english-corpora.org/corona/duplicates1.asp?xx=1755&n=2) |  |  |  | Twitter: MarkJenkinsDC. 87306533 # As a **coronavirus** **vaccination** tent was set up in the hope of inoculating more residents in Maryville, Tenn. |
| [1757](https://www.english-corpora.org/corona/x4.asp?rs=&t=87306674&ID=20487710824) | [21-05-27 US](https://www.english-corpora.org/corona/x4.asp?rs=&t=87306674&ID=20487710824) | [Yahoo](https://www.english-corpora.org/corona/x3.asp?node=&p=18&w10=coronavirus&w11=vaccination&r=) |  |  |  | over a third of the overall total. This month, India opened its **coronavirus** **vaccination** campaign to everyone aged 18 or older. # The group behind the SolarWinds cyber |
| [1760](https://www.english-corpora.org/corona/x4.asp?rs=&t=43034037&ID=20443087254) | [21-05-24 GB](https://www.english-corpora.org/corona/x4.asp?rs=&t=43034037&ID=20443087254) | [chroniclelive.co.uk](https://www.english-corpora.org/corona/x3.asp?node=&p=18&w10=coronavirus&w11=vaccination&r=) [(2)](https://www.english-corpora.org/corona/duplicates1.asp?xx=1759&n=2) |  |  |  | be eligible to get a **coronavirus** **vaccination** from Tuesday, Health Secretary Matt Hancock has announced. # And those aged 36 |
| [1761](https://www.english-corpora.org/corona/x4.asp?rs=&t=43044826&ID=20465010409) | [21-05-26 GB](https://www.english-corpora.org/corona/x4.asp?rs=&t=43044826&ID=20465010409) | [grimsbytelegraph.co.uk](https://www.english-corpora.org/corona/x3.asp?node=&p=18&w10=coronavirus&w11=vaccination&r=) |  |  |  | national booking system to select an alternative suitable date and time to book a **coronavirus** **vaccination** - NHS (www.nhs.uk) or ring 119 to book. Experts say it's |
| [1762](https://www.english-corpora.org/corona/x4.asp?rs=&t=43047229&ID=20465322953) | [21-05-26 GB](https://www.english-corpora.org/corona/x4.asp?rs=&t=43047229&ID=20465322953) | [dorsetecho.co.uk](https://www.english-corpora.org/corona/x3.asp?node=&p=18&w10=coronavirus&w11=vaccination&r=) |  |  |  | 30 and over invited to get their first **coronavirus** **vaccination** Coronavirus vaccination programme rolls out to all over 30s # The NHS Covid vaccination |
| [1763](https://www.english-corpora.org/corona/x4.asp?rs=&t=43047229&ID=20465322956) | [21-05-26 GB](https://www.english-corpora.org/corona/x4.asp?rs=&t=43047229&ID=20465322956) | [dorsetecho.co.uk](https://www.english-corpora.org/corona/x3.asp?node=&p=18&w10=coronavirus&w11=vaccination&r=) |  |  |  | 30 and over invited to get their first coronavirus vaccination **Coronavirus** **vaccination** programme rolls out to all over 30s # The NHS Covid vaccination programme will today |
| [1764](https://www.english-corpora.org/corona/x4.asp?rs=&t=43048091&ID=20481553179) | [21-05-27 GB](https://www.english-corpora.org/corona/x4.asp?rs=&t=43048091&ID=20481553179) | [lexology.com](https://www.english-corpora.org/corona/x3.asp?node=&p=18&w10=coronavirus&w11=vaccination&r=) |  |  |  | Department says states can use federal money to offer cash incentives aiming to boost **coronavirus** **vaccination** rates. The update comes in guidance updated May 25 detailing how state and local |
| [1765](https://www.english-corpora.org/corona/x4.asp?rs=&t=87257312&ID=20406475277) | [21-05-19 GB](https://www.english-corpora.org/corona/x4.asp?rs=&t=87257312&ID=20406475277) | [CNN](https://www.english-corpora.org/corona/x3.asp?node=&p=18&w10=coronavirus&w11=vaccination&r=) |  |  |  | doses # From CNN Health's Maggie Fox # The first nationwide study of **coronavirus** **vaccination**, done in Israel, shows Pfizer/BioNtech's vaccine works far better after two doses |
| [1766](https://www.english-corpora.org/corona/x4.asp?rs=&t=73040006&ID=20441791412) | [21-05-24 AU](https://www.english-corpora.org/corona/x4.asp?rs=&t=73040006&ID=20441791412) | [illawarramercury.com.au](https://www.english-corpora.org/corona/x3.asp?node=&p=18&w10=coronavirus&w11=vaccination&r=) |  |  |  | streak without a locally acquired case. * People entering Australia could provide their **coronavirus** **vaccination** status through a new pre-check-in digital declaration when international borders reopen, with a trial |
| [1767](https://www.english-corpora.org/corona/x4.asp?rs=&t=73040006&ID=20441792012) | [21-05-24 AU](https://www.english-corpora.org/corona/x4.asp?rs=&t=73040006&ID=20441792012) | [illawarramercury.com.au](https://www.english-corpora.org/corona/x3.asp?node=&p=18&w10=coronavirus&w11=vaccination&r=) |  |  |  | without a locally acquired case. # * People entering Australia could provide their **coronavirus** **vaccination** status through a new pre-check-in digital declaration when international borders reopen, with a trial |
| [1768](https://www.english-corpora.org/corona/x4.asp?rs=&t=73049297&ID=20472324476) | [21-05-26 AU](https://www.english-corpora.org/corona/x4.asp?rs=&t=73049297&ID=20472324476) | [bendigoadvertiser.com.au](https://www.english-corpora.org/corona/x3.asp?node=&p=18&w10=coronavirus&w11=vaccination&r=) |  |  |  | could provide **coronavirus** **vaccination** status through a new pre-check-in digital declaration when international borders reopen. Home Affairs boss |
| [1769](https://www.english-corpora.org/corona/x4.asp?rs=&t=73049297&ID=20472324797) | [21-05-26 AU](https://www.english-corpora.org/corona/x4.asp?rs=&t=73049297&ID=20472324797) | [bendigoadvertiser.com.au](https://www.english-corpora.org/corona/x3.asp?node=&p=18&w10=coronavirus&w11=vaccination&r=) |  |  |  | on vaccination could be trialled next year. # People entering Australia could provide **coronavirus** **vaccination** status through a new pre-check-in digital declaration when international borders reopen. # Home Affairs |
| [1770](https://www.english-corpora.org/corona/x4.asp?rs=&t=43016720&ID=20377246164) | [21-05-17 IN](https://www.english-corpora.org/corona/x4.asp?rs=&t=43016720&ID=20377246164) | [odishatv.in](https://www.english-corpora.org/corona/x3.asp?node=&p=18&w10=coronavirus&w11=vaccination&r=) |  |  |  | # New Delhi: In a bid to speed up the pace of the **coronavirus** **vaccination** drive, Union Health Minister Harsh Vardhan on Wednesday announced that citizens will now be |
| [1771](https://www.english-corpora.org/corona/x4.asp?rs=&t=73030818&ID=20413535179) | [21-05-20 IN](https://www.english-corpora.org/corona/x4.asp?rs=&t=73030818&ID=20413535179) | [zeebiz.com](https://www.english-corpora.org/corona/x3.asp?node=&p=18&w10=coronavirus&w11=vaccination&r=) |  |  |  | in the country. Source: PTI # In order to ramp up the **Coronavirus** **vaccination** process in the country, the government is planning to produce Oxford-AstraZeneca's Covishield, |
| [1772](https://www.english-corpora.org/corona/x4.asp?rs=&t=73040669&ID=20446788783) | [21-05-24 IN](https://www.english-corpora.org/corona/x4.asp?rs=&t=73040669&ID=20446788783) | [thehansindia.com](https://www.english-corpora.org/corona/x3.asp?node=&p=18&w10=coronavirus&w11=vaccination&r=) |  |  |  | Coronavirus **vaccination** to resume today in state for above 45 years of age # Coronavirus vaccination to |
| [1773](https://www.english-corpora.org/corona/x4.asp?rs=&t=73040669&ID=20446788797) | [21-05-24 IN](https://www.english-corpora.org/corona/x4.asp?rs=&t=73040669&ID=20446788797) | [thehansindia.com](https://www.english-corpora.org/corona/x3.asp?node=&p=18&w10=coronavirus&w11=vaccination&r=) |  |  |  | Coronavirus vaccination to resume today in state for above 45 years of age # **Coronavirus** **vaccination** to resume today in state for above 45 years of age # Highlights The |
| [1774](https://www.english-corpora.org/corona/x4.asp?rs=&t=73045516&ID=20453939743) | [21-05-25 IN](https://www.english-corpora.org/corona/x4.asp?rs=&t=73045516&ID=20453939743) | [theweek.in](https://www.english-corpora.org/corona/x3.asp?node=&p=18&w10=coronavirus&w11=vaccination&r=) |  |  |  | Deputy Chief Minister Manish Sisodia accused the central government of " mismanaging " the **coronavirus** **vaccination** programme, saying it has asked states to float global tenders for jabs without giving |
| [1775](https://www.english-corpora.org/corona/x4.asp?rs=&t=73045904&ID=20453975170) | [21-05-25 IN](https://www.english-corpora.org/corona/x4.asp?rs=&t=73045904&ID=20453975170) | [ibtimes.co.in](https://www.english-corpora.org/corona/x3.asp?node=&p=18&w10=coronavirus&w11=vaccination&r=) |  |  |  | died due to a prolonged illness, and it is not connected with the **coronavirus** **vaccination**. # William Shakespeare receiving Covid vaccineReuters # While receiving the coronavirus vaccine in December |
| [1776](https://www.english-corpora.org/corona/x4.asp?rs=&t=73045912&ID=20453979997) | [21-05-25 IN](https://www.english-corpora.org/corona/x4.asp?rs=&t=73045912&ID=20453979997) | [theprint.in](https://www.english-corpora.org/corona/x3.asp?node=&p=18&w10=coronavirus&w11=vaccination&r=) |  |  |  | famous Hindi saying, " Ram bharose ". # On the issue of **coronavirus** **vaccination**, the court had suggested that big business houses that take benefits under taxation laws |
| [1777](https://www.english-corpora.org/corona/x4.asp?rs=&t=73046105&ID=20454017750) | [21-05-25 IN](https://www.english-corpora.org/corona/x4.asp?rs=&t=73046105&ID=20454017750) | [theweekendleader.com](https://www.english-corpora.org/corona/x3.asp?node=&p=18&w10=coronavirus&w11=vaccination&r=) |  |  |  | Response, which is the European Commission's campaign for universal access to affordable **coronavirus** **vaccination**, treatment and testing. # The campaign launched in May last year has raised |
| [1778](https://www.english-corpora.org/corona/x4.asp?rs=&t=73048347&ID=20456883896) | [21-05-25 IN](https://www.english-corpora.org/corona/x4.asp?rs=&t=73048347&ID=20456883896) | [zeebiz.com](https://www.english-corpora.org/corona/x3.asp?node=&p=18&w10=coronavirus&w11=vaccination&r=) |  |  |  | recommendations of NEGVAC, the union government on Wednesday has deferred the **coronavirus** **vaccination** by three months after the recovery from the illness. # In its recommendation, |
| [1779](https://www.english-corpora.org/corona/x4.asp?rs=&t=73050434&ID=20469565512) | [21-05-26 IN](https://www.english-corpora.org/corona/x4.asp?rs=&t=73050434&ID=20469565512) | [nyoooz.com](https://www.english-corpora.org/corona/x3.asp?node=&p=18&w10=coronavirus&w11=vaccination&r=) |  |  |  | Chief Minister Manish Sisodia on Monday accused the Centre of " mismanaging " the **coronavirus** **vaccination** programme in the country, specifically saying that the entire vaccination programme for the youth |
| [1780](https://www.english-corpora.org/corona/x4.asp?rs=&t=87288335&ID=20449601990) | [21-05-24 IN](https://www.english-corpora.org/corona/x4.asp?rs=&t=87288335&ID=20449601990) | [Mint](https://www.english-corpora.org/corona/x3.asp?node=&p=18&w10=coronavirus&w11=vaccination&r=) |  |  |  | The advisory said that the only " official " online link to register for **coronavirus** **vaccination** in the country is the portal-- http: //cowin.gov.in. # It asked users to |
| [1781](https://www.english-corpora.org/corona/x4.asp?rs=&t=43029396&ID=20423980003) | [21-05-21 PK](https://www.english-corpora.org/corona/x4.asp?rs=&t=43029396&ID=20423980003) | [paktribune.com](https://www.english-corpora.org/corona/x3.asp?node=&p=18&w10=coronavirus&w11=vaccination&r=) |  |  |  | years fall under the categories mentioned below, they can simply walk into any **coronavirus** **vaccination** centre, show a valid visa, and get vaccinated, the NCOC sPakistanis working |
| [1782](https://www.english-corpora.org/corona/x4.asp?rs=&t=52742750&ID=20380377708) | [21-05-17 PK](https://www.english-corpora.org/corona/x4.asp?rs=&t=52742750&ID=20380377708) | [thenews.com.pk](https://www.english-corpora.org/corona/x3.asp?node=&p=18&w10=coronavirus&w11=vaccination&r=) |  |  |  | administered to the people on May 15, raising the total district tally of **coronavirus** **vaccination** to 179,084. He said that 19,200 health workers while 159,884 people including senior citizens |
| [1783](https://www.english-corpora.org/corona/x4.asp?rs=&t=52742907&ID=20377665303) | [21-05-17 PK](https://www.english-corpora.org/corona/x4.asp?rs=&t=52742907&ID=20377665303) | [tribune.com.pk](https://www.english-corpora.org/corona/x3.asp?node=&p=18&w10=coronavirus&w11=vaccination&r=) |  |  |  | Furthermore, DHA Chief Executive Officer Dr Faiza Kanwal said that there were 30 **coronavirus** **vaccination** centres currently operating in the district, including three big hospitals. She added the |
| [1784](https://www.english-corpora.org/corona/x4.asp?rs=&t=52743087&ID=20383007977) | [21-05-17 PK](https://www.english-corpora.org/corona/x4.asp?rs=&t=52743087&ID=20383007977) | [thenews.com.pk](https://www.english-corpora.org/corona/x3.asp?node=&p=18&w10=coronavirus&w11=vaccination&r=) |  |  |  | administered to the people on May 15, raising the total district tally of **coronavirus** **vaccination** to 179,084. He said that 19,200 health workers while 159,884 people including senior citizens |
| [1785](https://www.english-corpora.org/corona/x4.asp?rs=&t=52746851&ID=20392329555) | [21-05-18 PK](https://www.english-corpora.org/corona/x4.asp?rs=&t=52746851&ID=20392329555) | [suchtv.pk](https://www.english-corpora.org/corona/x3.asp?node=&p=18&w10=coronavirus&w11=vaccination&r=) |  |  |  | 's Sputnik V vaccine imported by a pharmaceutical company. # Pakistan in its **coronavirus** **vaccination** drive mostly used vaccines imported from China. The country on Sunday received another consignment |
| [1787](https://www.english-corpora.org/corona/x4.asp?rs=&t=52759403&ID=20435223808) | [21-05-23 PK](https://www.english-corpora.org/corona/x4.asp?rs=&t=52759403&ID=20435223808) | [samaa.tv](https://www.english-corpora.org/corona/x3.asp?node=&p=18&w10=coronavirus&w11=vaccination&r=) [(1)](https://www.english-corpora.org/corona/duplicates1.asp?xx=1786&n=1) |  |  |  | going for the pilgrimage will have to quarantine for three days. # **Coronavirus** **vaccination** is a must, he pointed out. # Minister for Religious Affairs Noorul Haq |
| [1788](https://www.english-corpora.org/corona/x4.asp?rs=&t=52741377&ID=20382753796) | [21-05-17 BD](https://www.english-corpora.org/corona/x4.asp?rs=&t=52741377&ID=20382753796) | [theindependentbd.com](https://www.english-corpora.org/corona/x3.asp?node=&p=18&w10=coronavirus&w11=vaccination&r=) |  |  |  | facilities while economic activities with various incentives is also going on. # " **Coronavirus** **vaccination** has already begun... The government is making strong efforts to procure vaccines |
| [1789](https://www.english-corpora.org/corona/x4.asp?rs=&t=52741377&ID=20382754206) | [21-05-17 BD](https://www.english-corpora.org/corona/x4.asp?rs=&t=52741377&ID=20382754206) | [theindependentbd.com](https://www.english-corpora.org/corona/x3.asp?node=&p=18&w10=coronavirus&w11=vaccination&r=) |  |  |  | their treatment facilities while economic activities with various incentives is also going on. **Coronavirus** **vaccination** has already begun... The government is making strong efforts to procure vaccines |
| [1790](https://www.english-corpora.org/corona/x4.asp?rs=&t=52745192&ID=20393544067) | [21-05-18 BD](https://www.english-corpora.org/corona/x4.asp?rs=&t=52745192&ID=20393544067) | [dhakatribune.com](https://www.english-corpora.org/corona/x3.asp?node=&p=18&w10=coronavirus&w11=vaccination&r=) |  |  |  | facilities while economic activities with various incentives is also going on. # " **Coronavirus** **vaccination** has already begun... The government is making strong efforts to procure vaccines |
| [1791](https://www.english-corpora.org/corona/x4.asp?rs=&t=43018912&ID=20390332248) | [21-05-18 SG](https://www.english-corpora.org/corona/x4.asp?rs=&t=43018912&ID=20390332248) | [sg.news.yahoo.com](https://www.english-corpora.org/corona/x3.asp?node=&p=18&w10=coronavirus&w11=vaccination&r=) |  |  |  | recreational facilities have been closed since Friday.More from South China Morning Post:Taiwan's low **coronavirus** **vaccination** rate under scrutiny as it battles spike in casesTaiwan coronavirus surge won't advance its |
| [1792](https://www.english-corpora.org/corona/x4.asp?rs=&t=73021453&ID=20381466282) | [21-05-17 PH](https://www.english-corpora.org/corona/x4.asp?rs=&t=73021453&ID=20381466282) | [news.abs-cbn.com](https://www.english-corpora.org/corona/x3.asp?node=&p=18&w10=coronavirus&w11=vaccination&r=) |  |  |  | administered to children. Read more? # The government should speed up its **coronavirus** **vaccination** efforts and tap the help of the private sector, a former adviser to the |
| [1793](https://www.english-corpora.org/corona/x4.asp?rs=&t=73046210&ID=20454027842) | [21-05-25 PH](https://www.english-corpora.org/corona/x4.asp?rs=&t=73046210&ID=20454027842) | [news.abs-cbn.com](https://www.english-corpora.org/corona/x3.asp?node=&p=18&w10=coronavirus&w11=vaccination&r=) |  |  |  | with a political ally facing possible impeachment. Read more? # Israel's **coronavirus** **vaccination** campaign, the world's fastest per capita, shifted to booster shots on Sunday |
| [1794](https://www.english-corpora.org/corona/x4.asp?rs=&t=73051004&ID=20466837722) | [21-05-26 PH](https://www.english-corpora.org/corona/x4.asp?rs=&t=73051004&ID=20466837722) | [ph.news.yahoo.com](https://www.english-corpora.org/corona/x3.asp?node=&p=18&w10=coronavirus&w11=vaccination&r=) |  |  |  | for essential work, healthcare, grocery shopping, exercise or to get a **coronavirus** **vaccination**. Several infected contacts have visited crowded hot spots that included sports stadiums and one |
| [1795](https://www.english-corpora.org/corona/x4.asp?rs=&t=73055227&ID=20479610862) | [21-05-27 PH](https://www.english-corpora.org/corona/x4.asp?rs=&t=73055227&ID=20479610862) | [ph.news.yahoo.com](https://www.english-corpora.org/corona/x3.asp?node=&p=18&w10=coronavirus&w11=vaccination&r=) |  |  |  | over a third of the overall total. This month, India opened its **coronavirus** **vaccination** campaign to everyone aged 18 or older. # Jailed Hong Kong media tycoon Jimmy |
| [1796](https://www.english-corpora.org/corona/x4.asp?rs=&t=43015107&ID=20377060435) | [21-05-17 HK](https://www.english-corpora.org/corona/x4.asp?rs=&t=43015107&ID=20377060435) | [nepal24hours.com](https://www.english-corpora.org/corona/x3.asp?node=&p=18&w10=coronavirus&w11=vaccination&r=) |  |  |  | Free the Vaccine, " calling on the US to commit to a global **coronavirus** **vaccination** plan that includes sharing vaccine formulas with the world to help ensure that every nation |
| [1797](https://www.english-corpora.org/corona/x4.asp?rs=&t=62962791&ID=20482396030) | [21-05-27 ZA](https://www.english-corpora.org/corona/x4.asp?rs=&t=62962791&ID=20482396030) | [timeslive.co.za](https://www.english-corpora.org/corona/x3.asp?node=&p=18&w10=coronavirus&w11=vaccination&r=) |  |  |  | for essential work, healthcare, grocery shopping, exercise or to get a **coronavirus** **vaccination**. # Several infected contacts have visited crowded hot spots that included sports stadiums and |
| [1798](https://www.english-corpora.org/corona/x4.asp?rs=&t=52761840&ID=20443934548) | [21-05-24 NG](https://www.english-corpora.org/corona/x4.asp?rs=&t=52761840&ID=20443934548) | [channelstv.com](https://www.english-corpora.org/corona/x3.asp?node=&p=18&w10=coronavirus&w11=vaccination&r=) |  |  |  | says the benefits outweigh the risks. # The scientific body overseeing Britain's **coronavirus** **vaccination** programme has recommended that under-40s be offered an alternative to AstraZeneca jabs. # AstraZeneca |
| [1799](https://www.english-corpora.org/corona/x4.asp?rs=&t=62961307&ID=20466515120) | [21-05-26 KE](https://www.english-corpora.org/corona/x4.asp?rs=&t=62961307&ID=20466515120) | [theeastafrican.co.ke](https://www.english-corpora.org/corona/x3.asp?node=&p=18&w10=coronavirus&w11=vaccination&r=) |  |  |  | 2021 # A health worker prepares a dose of the AstraZeneca/Oxford vaccine at a **coronavirus** **vaccination** centre at the Wizink Center in Madrid on May 12, 2021. PHOTO AFP |
| [1800](https://www.english-corpora.org/corona/x4.asp?rs=&t=62960287&ID=20472089510) | [21-05-26 JM](https://www.english-corpora.org/corona/x4.asp?rs=&t=62960287&ID=20472089510) | [jamaicaobserver.com](https://www.english-corpora.org/corona/x3.asp?node=&p=18&w10=coronavirus&w11=vaccination&r=) |  |  |  | says the benefits outweigh the risks. # The scientific body overseeing Britain's **coronavirus** **vaccination** programme has recommended that under-40s be offered an alternative to AstraZeneca jabs. # AstraZeneca |

| PAGE:   [**<<**](https://www.english-corpora.org/corona/x3.asp?node=&p=1&w10=coronavirus&w11=vaccination&r=)  [**<**](https://www.english-corpora.org/corona/x3.asp?node=&p=17&w10=coronavirus&w11=vaccination&r=)   18 / 42  [**>**](https://www.english-corpora.org/corona/x3.asp?node=&p=19&w10=coronavirus&w11=vaccination&r=)   [**>>**](https://www.english-corpora.org/corona/x3.asp?node=&p=42&w10=coronavirus&w11=vaccination&r=) | | |
| --- | --- | --- |
| PAGE:   [**<<**](https://www.english-corpora.org/corona/x3.asp?node=&p=1&w10=coronavirus&w11=vaccination&r=)  [**<**](https://www.english-corpora.org/corona/x3.asp?node=&p=18&w10=coronavirus&w11=vaccination&r=)   19 / 42  [**>**](https://www.english-corpora.org/corona/x3.asp?node=&p=20&w10=coronavirus&w11=vaccination&r=)   [**>>**](https://www.english-corpora.org/corona/x3.asp?node=&p=42&w10=coronavirus&w11=vaccination&r=) |  |  |

Начало формы

| CLICK FOR MORE CONTEXT | [**HELP**](javascript:newFeatures()) | [SAVE](javascript:chooser('s'))    [TRANSLATE](javascript:chooser('t'))    [ANALYZE](javascript:chooser('p')) |
| --- | --- | --- |

| [1801](https://www.english-corpora.org/corona/x4.asp?rs=&t=32815882&ID=20295417204) | [21-05-10 US](https://www.english-corpora.org/corona/x4.asp?rs=&t=32815882&ID=20295417204) | [dailyherald.com](https://www.english-corpora.org/corona/x3.asp?node=&p=19&w10=coronavirus&w11=vaccination&r=) |  |  |  | 's levels. # # LANSING, Mich. - Michigan has passed a 55% **coronavirus** **vaccination** rate, a benchmark that will lead to the easing of in-office work restrictions in |
| --- | --- | --- | --- | --- | --- | --- |
| [1802](https://www.english-corpora.org/corona/x4.asp?rs=&t=32817021&ID=20303476058) | [21-05-11 US](https://www.english-corpora.org/corona/x4.asp?rs=&t=32817021&ID=20303476058) | [firstpost.com](https://www.english-corpora.org/corona/x3.asp?node=&p=19&w10=coronavirus&w11=vaccination&r=) |  |  |  | made open for all senior citizens. # In order to ramp up the **coronavirus** **vaccination** drive in the country, the Centre had last month announced a' liberalised and |
| [1803](https://www.english-corpora.org/corona/x4.asp?rs=&t=32817299&ID=20309282074) | [21-05-11 US](https://www.english-corpora.org/corona/x4.asp?rs=&t=32817299&ID=20309282074) | [politico.com](https://www.english-corpora.org/corona/x3.asp?node=&p=19&w10=coronavirus&w11=vaccination&r=) |  |  |  | six governors -- three Democrats and three Republicans -- who scored invites to share **coronavirus** **vaccination** best practices with President Joe Biden this afternoon. GOP Govs. Spencer Cox of |
| [1804](https://www.english-corpora.org/corona/x4.asp?rs=&t=32822005&ID=20317671169) | [21-05-12 US](https://www.english-corpora.org/corona/x4.asp?rs=&t=32822005&ID=20317671169) | [politico.com](https://www.english-corpora.org/corona/x3.asp?node=&p=19&w10=coronavirus&w11=vaccination&r=) |  |  |  | said Monday that its vaccine advisory committee will meet on June 10 to discuss **coronavirus** **vaccination** in children, particularly those younger than 11. FDA does not have to take |
| [1805](https://www.english-corpora.org/corona/x4.asp?rs=&t=32822059&ID=20321567645) | [21-05-12 US](https://www.english-corpora.org/corona/x4.asp?rs=&t=32822059&ID=20321567645) | [politico.com](https://www.english-corpora.org/corona/x3.asp?node=&p=19&w10=coronavirus&w11=vaccination&r=) |  |  |  | to do. " # She gave her comments during meeting of the Statewide **Coronavirus** **Vaccination** Community Education and Engagement Task Force, a panel of faith, community and medical |
| [1806](https://www.english-corpora.org/corona/x4.asp?rs=&t=32822507&ID=20317775300) | [21-05-12 US](https://www.english-corpora.org/corona/x4.asp?rs=&t=32822507&ID=20317775300) | [bostonherald.com](https://www.english-corpora.org/corona/x3.asp?node=&p=19&w10=coronavirus&w11=vaccination&r=) |  |  |  | age 12-15 in Massachusetts. # Baker said there are no plans to mandate **coronavirus** **vaccination** in public school students. # Shortly after the ACIP meeting, CVS announced coronavirus |
| [1807](https://www.english-corpora.org/corona/x4.asp?rs=&t=32825220&ID=20318231073) | [21-05-12 US](https://www.english-corpora.org/corona/x4.asp?rs=&t=32825220&ID=20318231073) | [twincities.com](https://www.english-corpora.org/corona/x3.asp?node=&p=19&w10=coronavirus&w11=vaccination&r=) |  |  |  | Elise Robinson receives her first **coronavirus** **vaccination** on Wednesday, May 12, 2021, in Decatur, Ga. Hundreds of |
| [1808](https://www.english-corpora.org/corona/x4.asp?rs=&t=32827524&ID=20334687128) | [21-05-13 US](https://www.english-corpora.org/corona/x4.asp?rs=&t=32827524&ID=20334687128) | [bostonherald.com](https://www.english-corpora.org/corona/x3.asp?node=&p=19&w10=coronavirus&w11=vaccination&r=) |  |  |  | departures elsewhere over a law that bans businesses from asking for proof of a **coronavirus** **vaccination**. Norwegian hasn't operated in the U.S. since the federal government shut down all |
| [1809](https://www.english-corpora.org/corona/x4.asp?rs=&t=32830669&ID=20351735702) | [21-05-14 US](https://www.english-corpora.org/corona/x4.asp?rs=&t=32830669&ID=20351735702) | [ndtv.com](https://www.english-corpora.org/corona/x3.asp?node=&p=19&w10=coronavirus&w11=vaccination&r=) |  |  |  | further information as it becomes available, " it added, stressing Ireland's **coronavirus** **vaccination** programme was unaffected and " going ahead as planned ". # Another ransomware attack |
| [1810](https://www.english-corpora.org/corona/x4.asp?rs=&t=32837025&ID=20368816794) | [21-05-16 US](https://www.english-corpora.org/corona/x4.asp?rs=&t=32837025&ID=20368816794) | [washingtonexaminer.com](https://www.english-corpora.org/corona/x3.asp?node=&p=19&w10=coronavirus&w11=vaccination&r=) |  |  |  | which significantly slowed gas delivery in Southeastern states this week. # Ireland's **coronavirus** **vaccination** program has not been disrupted due to the attack yet, and scheduled appointments to |
| [1811](https://www.english-corpora.org/corona/x4.asp?rs=&t=32837752&ID=20370215966) | [21-05-16 US](https://www.english-corpora.org/corona/x4.asp?rs=&t=32837752&ID=20370215966) | [pilotonline.com](https://www.english-corpora.org/corona/x3.asp?node=&p=19&w10=coronavirus&w11=vaccination&r=) |  |  |  | n't see it making much difference to the vast majority of Republicans who oppose **coronavirus** **vaccination** on political grounds, however, which is a shame, given that the single |
| [1812](https://www.english-corpora.org/corona/x4.asp?rs=&t=43005506&ID=20335605785) | [21-05-13 US](https://www.english-corpora.org/corona/x4.asp?rs=&t=43005506&ID=20335605785) | [wsbtv.com](https://www.english-corpora.org/corona/x3.asp?node=&p=19&w10=coronavirus&w11=vaccination&r=) |  |  |  | your homebound patients to call this number. " # As interest in mass **coronavirus** **vaccination** sites dwindles nationwide, providers are ramping up efforts to find and reach millions of |
| [1813](https://www.english-corpora.org/corona/x4.asp?rs=&t=52722005&ID=20304991555) | [21-05-11 US](https://www.english-corpora.org/corona/x4.asp?rs=&t=52722005&ID=20304991555) | [pri.org](https://www.english-corpora.org/corona/x3.asp?node=&p=19&w10=coronavirus&w11=vaccination&r=) |  |  |  | of the coronavirus walks by masked residents lining up for COVID-19 vaccine at a **coronavirus** **vaccination** site with a board displaying the slogan, " Timely vaccination to build the Great |
| [1814](https://www.english-corpora.org/corona/x4.asp?rs=&t=52726411&ID=20319724923) | [21-05-12 US](https://www.english-corpora.org/corona/x4.asp?rs=&t=52726411&ID=20319724923) | [ibtimes.com](https://www.english-corpora.org/corona/x3.asp?node=&p=19&w10=coronavirus&w11=vaccination&r=) |  |  |  | the centuries-old union. # Johnson's government, after rolling out a successful **coronavirus** **vaccination** drive, is intent on reopening the economy and " levelling up " prosperity across |
| [1815](https://www.english-corpora.org/corona/x4.asp?rs=&t=62918254&ID=20324479685) | [21-05-12 US](https://www.english-corpora.org/corona/x4.asp?rs=&t=62918254&ID=20324479685) | [reuters.com](https://www.english-corpora.org/corona/x3.asp?node=&p=19&w10=coronavirus&w11=vaccination&r=) |  |  |  | . # Japan's vaccine booking system crashes # Technical problems derailed Japan's **coronavirus** **vaccination** booking system on Wednesday, compounding frustration over the government's handling of new outbreaks |
| [1816](https://www.english-corpora.org/corona/x4.asp?rs=&t=62920837&ID=20339769841) | [21-05-13 US](https://www.english-corpora.org/corona/x4.asp?rs=&t=62920837&ID=20339769841) | [q13fox.com](https://www.english-corpora.org/corona/x3.asp?node=&p=19&w10=coronavirus&w11=vaccination&r=) |  |  |  | . # All state residents age 16 and up have been eligible for a **coronavirus** **vaccination** since April 15. As of this week, more than 6 million doses of |
| [1817](https://www.english-corpora.org/corona/x4.asp?rs=&t=73009062&ID=20340449369) | [21-05-13 US](https://www.english-corpora.org/corona/x4.asp?rs=&t=73009062&ID=20340449369) | [kwch.com](https://www.english-corpora.org/corona/x3.asp?node=&p=19&w10=coronavirus&w11=vaccination&r=) |  |  |  | to ages 12+ in Kansas # Middle school student Elise Robinson receives her first **coronavirus** **vaccination** on Wednesday, May 12, 2021, in Decatur, Ga. Hundreds of |
| [1818](https://www.english-corpora.org/corona/x4.asp?rs=&t=87189989&ID=20268736913) | [21-05-07 US](https://www.english-corpora.org/corona/x4.asp?rs=&t=87189989&ID=20268736913) | [Omaha.com](https://www.english-corpora.org/corona/x3.asp?node=&p=19&w10=coronavirus&w11=vaccination&r=) |  |  |  | April 23, 2021. Appointments are no longer necessary at any of the **coronavirus** **vaccination** sites run by New York City. New York City Mayor Bill de Blasio announced |
| [1822](https://www.english-corpora.org/corona/x4.asp?rs=&t=87192755&ID=20266810199) | [21-05-07 US](https://www.english-corpora.org/corona/x4.asp?rs=&t=87192755&ID=20266810199) | [The Washington Post on MSN.com](https://www.english-corpora.org/corona/x3.asp?node=&p=19&w10=coronavirus&w11=vaccination&r=) [(3)](https://www.english-corpora.org/corona/duplicates1.asp?xx=1821&n=3) |  |  |  | accounts or in its overwhelmingly positive Google reviews: to buy a fake **coronavirus** **vaccination** card. # Agents with the California Department of Alcoholic Beverage Control arrested Old Corner |
| [1823](https://www.english-corpora.org/corona/x4.asp?rs=&t=87198604&ID=20285405126) | [21-05-09 US](https://www.english-corpora.org/corona/x4.asp?rs=&t=87198604&ID=20285405126) | [Mercury News](https://www.english-corpora.org/corona/x3.asp?node=&p=19&w10=coronavirus&w11=vaccination&r=) |  |  |  | # # NEW YORK -- Appointments are no longer necessary at any of the **coronavirus** **vaccination** sites run by New York City. # Mayor Bill de Blasio announced anyone eligible |
| [1824](https://www.english-corpora.org/corona/x4.asp?rs=&t=87199372&ID=20287440590) | [21-05-09 US](https://www.english-corpora.org/corona/x4.asp?rs=&t=87199372&ID=20287440590) | [YAHOO!News](https://www.english-corpora.org/corona/x3.asp?node=&p=19&w10=coronavirus&w11=vaccination&r=) |  |  |  | , as dozens of travelers from Latin America waited their turn at a pop-up **coronavirus** **vaccination** booth. # Sweating under the Sunday afternoon sun, visitors checked into an online |
| [1825](https://www.english-corpora.org/corona/x4.asp?rs=&t=87205095&ID=20302458031) | [21-05-10 US](https://www.english-corpora.org/corona/x4.asp?rs=&t=87205095&ID=20302458031) | [CNN](https://www.english-corpora.org/corona/x3.asp?node=&p=19&w10=coronavirus&w11=vaccination&r=) |  |  |  | consultancy Globsec found that Romanian and Bulgarian respondents were least inclined to get a **coronavirus** **vaccination** among people surveyed in 10 Central and Eastern European countries. |
| [1826](https://www.english-corpora.org/corona/x4.asp?rs=&t=87205244&ID=20300826694) | [21-05-10 US](https://www.english-corpora.org/corona/x4.asp?rs=&t=87205244&ID=20300826694) | [Forbes](https://www.english-corpora.org/corona/x3.asp?node=&p=19&w10=coronavirus&w11=vaccination&r=) |  |  |  | 's Where The Drop Off Is The Worst Topline # The U.S.'s **coronavirus** **vaccination** rate took yet another significant tumble over the past week, with all but four |
| [1827](https://www.english-corpora.org/corona/x4.asp?rs=&t=87207873&ID=20302278902) | [21-05-10 US](https://www.english-corpora.org/corona/x4.asp?rs=&t=87207873&ID=20302278902) | [Politico](https://www.english-corpora.org/corona/x3.asp?node=&p=19&w10=coronavirus&w11=vaccination&r=) |  |  |  | from Suffolk County Community College prepare to get vaccinated during a press conference on **coronavirus** **vaccination** at Suffolk County Community College in Brentwood, N.Y. Michael M. Santiago/Getty Images # |
| [1828](https://www.english-corpora.org/corona/x4.asp?rs=&t=87207903&ID=20299085797) | [21-05-10 US](https://www.english-corpora.org/corona/x4.asp?rs=&t=87207903&ID=20299085797) | [Washington Post](https://www.english-corpora.org/corona/x3.asp?node=&p=19&w10=coronavirus&w11=vaccination&r=) |  |  |  | weight off everyone's shoulders, " Campbell said. # Australia's national **coronavirus** **vaccination** rollout is behind schedule and Olympic participants needed special government approval to access vaccines this |
| [1829](https://www.english-corpora.org/corona/x4.asp?rs=&t=87209617&ID=20313233495) | [21-05-11 US](https://www.english-corpora.org/corona/x4.asp?rs=&t=87209617&ID=20313233495) | [ABC](https://www.english-corpora.org/corona/x3.asp?node=&p=19&w10=coronavirus&w11=vaccination&r=) |  |  |  | are accused of trying to inoculate patients with empty syringes during the country's **coronavirus** **vaccination** campaign # ByThe Associated Press # May 12, 2021, 12:04 AM # * |
| [1830](https://www.english-corpora.org/corona/x4.asp?rs=&t=87209617&ID=20313233545) | [21-05-11 US](https://www.english-corpora.org/corona/x4.asp?rs=&t=87209617&ID=20313233545) | [ABC](https://www.english-corpora.org/corona/x3.asp?node=&p=19&w10=coronavirus&w11=vaccination&r=) |  |  |  | are accused of trying to inoculate patients with empty syringes during the country's **coronavirus** **vaccination** campaign. # The ministry said the three known cases were reported in the capital |
| [1831](https://www.english-corpora.org/corona/x4.asp?rs=&t=87212710&ID=20312146143) | [21-05-11 US](https://www.english-corpora.org/corona/x4.asp?rs=&t=87212710&ID=20312146143) | [Washington Post](https://www.english-corpora.org/corona/x3.asp?node=&p=19&w10=coronavirus&w11=vaccination&r=) |  |  |  | his granddaughters. # Whether it's his policies -- his management of the **coronavirus** **vaccination** drive and his decision to withdraw U.S. troops from Afghanistan have won majority support in |
| [1832](https://www.english-corpora.org/corona/x4.asp?rs=&t=87212996&ID=20316879366) | [21-05-11 US](https://www.english-corpora.org/corona/x4.asp?rs=&t=87212996&ID=20316879366) | [MarketWatch](https://www.english-corpora.org/corona/x3.asp?node=&p=19&w10=coronavirus&w11=vaccination&r=) |  |  |  | some kind of vaccine mandate, although no U.S. state has yet made the **coronavirus** **vaccination** mandatory; only 44 states and Washington D.C. grant religious exemptions, and even fewer |
| [1833](https://www.english-corpora.org/corona/x4.asp?rs=&t=87213201&ID=20312175471) | [21-05-11 US](https://www.english-corpora.org/corona/x4.asp?rs=&t=87213201&ID=20312175471) | [Yahoo](https://www.english-corpora.org/corona/x3.asp?node=&p=19&w10=coronavirus&w11=vaccination&r=) |  |  |  | 0830 Attachment Q2 Company announcement no. 13 202021 # Technical problems derailed Japan's **coronavirus** **vaccination** booking system on Wednesday, compounding frustration over the government's handling of new outbreaks |
| [1835](https://www.english-corpora.org/corona/x4.asp?rs=&t=87213585&ID=20316510995) | [21-05-11 US](https://www.english-corpora.org/corona/x4.asp?rs=&t=87213585&ID=20316510995) | [Slate](https://www.english-corpora.org/corona/x3.asp?node=&p=19&w10=coronavirus&w11=vaccination&r=) [(1)](https://www.english-corpora.org/corona/duplicates1.asp?xx=1834&n=1) |  |  |  | letter Monday that it will not employ any staff that has received a **coronavirus** **vaccination** and those that have gotten vaccinated will be asked to isolate from students, who |
| [1836](https://www.english-corpora.org/corona/x4.asp?rs=&t=87214614&ID=20313138053) | [21-05-11 US](https://www.english-corpora.org/corona/x4.asp?rs=&t=87214614&ID=20313138053) | [SFGate](https://www.english-corpora.org/corona/x3.asp?node=&p=19&w10=coronavirus&w11=vaccination&r=) |  |  |  | are accused of trying to inoculate patients with empty syringes during the country's **coronavirus** **vaccination** campaign. # The ministry said the three known cases were reported in the capital |
| [1837](https://www.english-corpora.org/corona/x4.asp?rs=&t=87218335&ID=20331678904) | [21-05-12 US](https://www.english-corpora.org/corona/x4.asp?rs=&t=87218335&ID=20331678904) | [Washington Post](https://www.english-corpora.org/corona/x3.asp?node=&p=19&w10=coronavirus&w11=vaccination&r=) |  |  |  | was thrilled to receive her **coronavirus** **vaccination** in Memphis in March. " When I hear people say,' Well, |
| [1838](https://www.english-corpora.org/corona/x4.asp?rs=&t=87219355&ID=20331745443) | [21-05-12 US](https://www.english-corpora.org/corona/x4.asp?rs=&t=87219355&ID=20331745443) | [SFGate](https://www.english-corpora.org/corona/x3.asp?node=&p=19&w10=coronavirus&w11=vaccination&r=) |  |  |  | Georgia clinic after vaccine approved # 1of12Middle school student Elise Robinson receives her first **coronavirus** **vaccination** on Wednesday, May 12, 2021, in Decatur, Ga. Hundreds of |
| [1839](https://www.english-corpora.org/corona/x4.asp?rs=&t=87219355&ID=20331745510) | [21-05-12 US](https://www.english-corpora.org/corona/x4.asp?rs=&t=87219355&ID=20331745510) | [SFGate](https://www.english-corpora.org/corona/x3.asp?node=&p=19&w10=coronavirus&w11=vaccination&r=) |  |  |  | , left, waits with her mother, Anya Robinson, for her first **coronavirus** **vaccination** on Wednesday, May 12, 2021, in Decatur, Ga. Hundreds of |
| [1840](https://www.english-corpora.org/corona/x4.asp?rs=&t=87219355&ID=20331745573) | [21-05-12 US](https://www.english-corpora.org/corona/x4.asp?rs=&t=87219355&ID=20331745573) | [SFGate](https://www.english-corpora.org/corona/x3.asp?node=&p=19&w10=coronavirus&w11=vaccination&r=) |  |  |  | # 3of124of12Middle school student Meredith Rogers, center, waits to receive her first **coronavirus** **vaccination** on Wednesday, May 12, 2021, in Decatur, Ga. Hundreds of |
| [1841](https://www.english-corpora.org/corona/x4.asp?rs=&t=87219355&ID=20331745639) | [21-05-12 US](https://www.english-corpora.org/corona/x4.asp?rs=&t=87219355&ID=20331745639) | [SFGate](https://www.english-corpora.org/corona/x3.asp?node=&p=19&w10=coronavirus&w11=vaccination&r=) |  |  |  | Rogers, left, waits outside the DeKalb Pediatric Center after receiving her first **coronavirus** **vaccination** on Wednesday, May 12, 2021, in Decatur, Ga. Hundreds of |
| [1842](https://www.english-corpora.org/corona/x4.asp?rs=&t=87219766&ID=20332114221) | [21-05-12 US](https://www.english-corpora.org/corona/x4.asp?rs=&t=87219766&ID=20332114221) | [NBC News](https://www.english-corpora.org/corona/x3.asp?node=&p=19&w10=coronavirus&w11=vaccination&r=) |  |  |  | are accused of trying to inoculate patients with empty syringes during the country's **coronavirus** **vaccination** campaign. # The ministry said the three known cases were reported in the capital |
| [1843](https://www.english-corpora.org/corona/x4.asp?rs=&t=87221392&ID=20333089828) | [21-05-12 US](https://www.english-corpora.org/corona/x4.asp?rs=&t=87221392&ID=20333089828) | [New York Times](https://www.english-corpora.org/corona/x3.asp?node=&p=19&w10=coronavirus&w11=vaccination&r=) |  |  |  | this month.Credit... Brittainy Newman for The New York Times # All state-run mass **coronavirus** **vaccination** sites in New York will allow anyone 16 or older to walk in without an |
| [1844](https://www.english-corpora.org/corona/x4.asp?rs=&t=87221602&ID=20330203957) | [21-05-12 US](https://www.english-corpora.org/corona/x4.asp?rs=&t=87221602&ID=20330203957) | [Washington Post](https://www.english-corpora.org/corona/x3.asp?node=&p=19&w10=coronavirus&w11=vaccination&r=) |  |  |  | with prior illnesses. And the CDC says it has no evidence to establish **coronavirus** **vaccination** as the cause of any death. # Johnson also said, " I'm |
| [1845](https://www.english-corpora.org/corona/x4.asp?rs=&t=87222329&ID=20344938726) | [21-05-13 US](https://www.english-corpora.org/corona/x4.asp?rs=&t=87222329&ID=20344938726) | [Associated Press](https://www.english-corpora.org/corona/x3.asp?node=&p=19&w10=coronavirus&w11=vaccination&r=) |  |  |  | of the package. # The first lady opened her brief remarks at a **coronavirus** **vaccination** clinic by saying Manchin's " integrity and commitment to this state is steadfast. |
| [1846](https://www.english-corpora.org/corona/x4.asp?rs=&t=87225795&ID=20347865058) | [21-05-13 US](https://www.english-corpora.org/corona/x4.asp?rs=&t=87225795&ID=20347865058) | [The Washington Post on MSN.com](https://www.english-corpora.org/corona/x3.asp?node=&p=19&w10=coronavirus&w11=vaccination&r=) |  |  |  | has slowed down # Julian Boyce, 14, receives his first Pfizer **coronavirus** **vaccination** at NYC Health + Hospitals/Harlem from nurse Kenia Georges in New York on May 13 |
| [1847](https://www.english-corpora.org/corona/x4.asp?rs=&t=87226010&ID=20343701898) | [21-05-13 US](https://www.english-corpora.org/corona/x4.asp?rs=&t=87226010&ID=20343701898) | [TheStreet.com](https://www.english-corpora.org/corona/x3.asp?node=&p=19&w10=coronavirus&w11=vaccination&r=) |  |  |  | further $100 will be paid to new employees who can provide proof of a **coronavirus** **vaccination**, the company said. # The job plans followed a statement from McDonald's |
| [1848](https://www.english-corpora.org/corona/x4.asp?rs=&t=87227884&ID=20347930812) | [21-05-13 US](https://www.english-corpora.org/corona/x4.asp?rs=&t=87227884&ID=20347930812) | [Computerworld](https://www.english-corpora.org/corona/x3.asp?node=&p=19&w10=coronavirus&w11=vaccination&r=) |  |  |  | want serious advice on how to handle employees who refuse to get with the **coronavirus** **vaccination** program, it's time to find an employment law-savvy attorney. # Personally, |
| [1849](https://www.english-corpora.org/corona/x4.asp?rs=&t=87228217&ID=20344901401) | [21-05-13 US](https://www.english-corpora.org/corona/x4.asp?rs=&t=87228217&ID=20344901401) | [Washington Post](https://www.english-corpora.org/corona/x3.asp?node=&p=19&w10=coronavirus&w11=vaccination&r=) |  |  |  | . " # Claire Boogaard, the medical director for the Children's Hospital **coronavirus** **vaccination** program, said more than 6,000 12-to 15-year-olds had registered in advance to be vaccinated |
| [1850](https://www.english-corpora.org/corona/x4.asp?rs=&t=87235159&ID=20367283747) | [21-05-15 US](https://www.english-corpora.org/corona/x4.asp?rs=&t=87235159&ID=20367283747) | [Gizmodo](https://www.english-corpora.org/corona/x3.asp?node=&p=19&w10=coronavirus&w11=vaccination&r=) |  |  |  | detailed instructions on how to create fake official Centers for Disease Control and Prevention **coronavirus** **vaccination** cards rather than just get the damn shot. Doing so, the FBI says |
| [1851](https://www.english-corpora.org/corona/x4.asp?rs=&t=52720933&ID=20304821448) | [21-05-11 CA](https://www.english-corpora.org/corona/x4.asp?rs=&t=52720933&ID=20304821448) | [ca.finance.yahoo.com](https://www.english-corpora.org/corona/x3.asp?node=&p=19&w10=coronavirus&w11=vaccination&r=) |  |  |  | year ago as the coronavirus pandemic hit. # Technical problems derailed Japan's **coronavirus** **vaccination** booking system on Wednesday, compounding frustration over the government's handling of new outbreaks |
| [1852](https://www.english-corpora.org/corona/x4.asp?rs=&t=52723913&ID=20319323285) | [21-05-12 CA](https://www.english-corpora.org/corona/x4.asp?rs=&t=52723913&ID=20319323285) | [ca.finance.yahoo.com](https://www.english-corpora.org/corona/x3.asp?node=&p=19&w10=coronavirus&w11=vaccination&r=) |  |  |  | the Company's business and financial results, including the impact and timing of **coronavirus** **vaccination** programs; estimated fluctuations in global oil demand and supply levels, including anticipated future |
| [1853](https://www.english-corpora.org/corona/x4.asp?rs=&t=52723913&ID=20319323625) | [21-05-12 CA](https://www.english-corpora.org/corona/x4.asp?rs=&t=52723913&ID=20319323625) | [ca.finance.yahoo.com](https://www.english-corpora.org/corona/x3.asp?node=&p=19&w10=coronavirus&w11=vaccination&r=) |  |  |  | 's ability to maintain safe and efficient operations; the impact and timing of **coronavirus** **vaccination** programs; issues with vessel operations; higher than expected costs and expenses, off-hire |
| [1854](https://www.english-corpora.org/corona/x4.asp?rs=&t=52731141&ID=20339239308) | [21-05-13 CA](https://www.english-corpora.org/corona/x4.asp?rs=&t=52731141&ID=20339239308) | [chroniclejournal.com](https://www.english-corpora.org/corona/x3.asp?node=&p=19&w10=coronavirus&w11=vaccination&r=) |  |  |  | are accused of trying to inoculate patients with empty syringes during the country's **coronavirus** **vaccination** campaign. # The ministry said the three known cases were reported in the capital |
| [1855](https://www.english-corpora.org/corona/x4.asp?rs=&t=43001729&ID=20335194122) | [21-05-13 GB](https://www.english-corpora.org/corona/x4.asp?rs=&t=43001729&ID=20335194122) | [lexology.com](https://www.english-corpora.org/corona/x3.asp?node=&p=19&w10=coronavirus&w11=vaccination&r=) |  |  |  | has reached agreements with ride-sharing companies Uber and Lyft to offer free rides to **coronavirus** **vaccination** sites through July 4, the White House announced Tuesday. The federal government is |
| [1856](https://www.english-corpora.org/corona/x4.asp?rs=&t=62916252&ID=20324190716) | [21-05-12 IE](https://www.english-corpora.org/corona/x4.asp?rs=&t=62916252&ID=20324190716) | [theparliamentmagazine.eu](https://www.english-corpora.org/corona/x3.asp?node=&p=19&w10=coronavirus&w11=vaccination&r=) |  |  |  | , been a big improvement in the epidemiological situation and progress made in the **Coronavirus** **vaccination** campaign. # Coincidentally, the newly-launched Conference on the Future of Europe is due |
| [1857](https://www.english-corpora.org/corona/x4.asp?rs=&t=62923278&ID=20352211096) | [21-05-14 IE](https://www.english-corpora.org/corona/x4.asp?rs=&t=62923278&ID=20352211096) | [irishmirror.ie](https://www.english-corpora.org/corona/x3.asp?node=&p=19&w10=coronavirus&w11=vaccination&r=) |  |  |  | on emergency ambulance call handling and dispatch nationally. " # 09:33Colin Brennan **Coronavirus** **vaccination** programme was unaffected # Ireland's health service operator shut down all its IT systems |
| [1858](https://www.english-corpora.org/corona/x4.asp?rs=&t=62923278&ID=20352211130) | [21-05-14 IE](https://www.english-corpora.org/corona/x4.asp?rs=&t=62923278&ID=20352211130) | [irishmirror.ie](https://www.english-corpora.org/corona/x3.asp?node=&p=19&w10=coronavirus&w11=vaccination&r=) |  |  |  | protect it from what it described as a significant ransomware attack but said its **coronavirus** **vaccination** programme was unaffected. # The head of the Health Service Executive (HSE) |
| [1859](https://www.english-corpora.org/corona/x4.asp?rs=&t=72996451&ID=20308677946) | [21-05-11 AU](https://www.english-corpora.org/corona/x4.asp?rs=&t=72996451&ID=20308677946) | [au.finance.yahoo.com](https://www.english-corpora.org/corona/x3.asp?node=&p=19&w10=coronavirus&w11=vaccination&r=) |  |  |  | 72996451 Bo Bichette Latest stories # Technical problems derailed Japan's **coronavirus** **vaccination** booking system on Wednesday, compounding frustration over the government's handling of new outbreaks |
| [1860](https://www.english-corpora.org/corona/x4.asp?rs=&t=42991638&ID=20292649537) | [21-05-10 IN](https://www.english-corpora.org/corona/x4.asp?rs=&t=42991638&ID=20292649537) | [orissapost.com](https://www.english-corpora.org/corona/x3.asp?node=&p=19&w10=coronavirus&w11=vaccination&r=) |  |  |  | Test Championship final against New Zealand. # In order to ramp up the **coronavirus** **vaccination** drive in India, the Centre had last month announced a' liberalised and accelerated |
| [1861](https://www.english-corpora.org/corona/x4.asp?rs=&t=43005793&ID=20341596810) | [21-05-13 IN](https://www.english-corpora.org/corona/x4.asp?rs=&t=43005793&ID=20341596810) | [odishatv.in](https://www.english-corpora.org/corona/x3.asp?node=&p=19&w10=coronavirus&w11=vaccination&r=) |  |  |  | the states and UTs to review the preparedness for the nationwide mock drill for **coronavirus** **vaccination** scheduled for Friday. The second nationwide mock drill on the Covid19 vaccination will be |
| [1862](https://www.english-corpora.org/corona/x4.asp?rs=&t=43010164&ID=20361589960) | [21-05-15 IN](https://www.english-corpora.org/corona/x4.asp?rs=&t=43010164&ID=20361589960) | [odishatv.in](https://www.english-corpora.org/corona/x3.asp?node=&p=19&w10=coronavirus&w11=vaccination&r=) |  |  |  | minister... # New Delhi: The Union Cabinet on Tuesday allowed administration of **coronavirus** **vaccination** for people above 45 years of age from April 1, irrespective of the co-morbidities |
| [1863](https://www.english-corpora.org/corona/x4.asp?rs=&t=43012527&ID=20369391951) | [21-05-16 IN](https://www.english-corpora.org/corona/x4.asp?rs=&t=43012527&ID=20369391951) | [odishatv.in](https://www.english-corpora.org/corona/x3.asp?node=&p=19&w10=coronavirus&w11=vaccination&r=) |  |  |  | ... # Bhubaneswar: The Union Health Ministry said on Saturday that the much-awaited **coronavirus** **vaccination** drive will kick off on January 16. " It was decided that in view |
| [1864](https://www.english-corpora.org/corona/x4.asp?rs=&t=62922022&ID=20336978366) | [21-05-13 IN](https://www.english-corpora.org/corona/x4.asp?rs=&t=62922022&ID=20336978366) | [ptinews.com](https://www.english-corpora.org/corona/x3.asp?node=&p=19&w10=coronavirus&w11=vaccination&r=) |  |  |  | made open for all senior citizens. # In order to ramp up the **coronavirus** **vaccination** drive in the country, the Centre had last month announced a' liberalised and |
| [1865](https://www.english-corpora.org/corona/x4.asp?rs=&t=72988942&ID=20273033806) | [21-05-08 IN](https://www.english-corpora.org/corona/x4.asp?rs=&t=72988942&ID=20273033806) | [nyoooz.com](https://www.english-corpora.org/corona/x3.asp?node=&p=19&w10=coronavirus&w11=vaccination&r=) |  |  |  | weeks. # The AAP-led Delhi Government on Friday decided to organise a mass **coronavirus** **vaccination** drive for all the media houses, including electronic print and digital media. # |
| [1866](https://www.english-corpora.org/corona/x4.asp?rs=&t=72989703&ID=20271126165) | [21-05-08 IN](https://www.english-corpora.org/corona/x4.asp?rs=&t=72989703&ID=20271126165) | [siasat.com](https://www.english-corpora.org/corona/x3.asp?node=&p=19&w10=coronavirus&w11=vaccination&r=) |  |  |  | made open for all senior citizens. # In order to ramp up the **coronavirus** **vaccination** drive in the country, the Indian government had last month announced a " liberalised |
| [1868](https://www.english-corpora.org/corona/x4.asp?rs=&t=72993471&ID=20297888918) | [21-05-10 IN](https://www.english-corpora.org/corona/x4.asp?rs=&t=72993471&ID=20297888918) | [thestatesman.com](https://www.english-corpora.org/corona/x3.asp?node=&p=19&w10=coronavirus&w11=vaccination&r=) [(1)](https://www.english-corpora.org/corona/duplicates1.asp?xx=1867&n=1) |  |  |  | with AstraZeneca in this regard, while assuring India that its own anti- **coronavirus** **vaccination** programme will not be disturbed due to this supply. " The stock of 50 |
| [1869](https://www.english-corpora.org/corona/x4.asp?rs=&t=72996900&ID=20305896462) | [21-05-11 IN](https://www.english-corpora.org/corona/x4.asp?rs=&t=72996900&ID=20305896462) | [firstpost.com](https://www.english-corpora.org/corona/x3.asp?node=&p=19&w10=coronavirus&w11=vaccination&r=) |  |  |  | made open for all senior citizens. # In order to ramp up the **coronavirus** **vaccination** drive in the country, the Centre had last month announced a' liberalised and |
| [1871](https://www.english-corpora.org/corona/x4.asp?rs=&t=73000392&ID=20328845143) | [21-05-12 IN](https://www.english-corpora.org/corona/x4.asp?rs=&t=73000392&ID=20328845143) | [devdiscourse.com](https://www.english-corpora.org/corona/x3.asp?node=&p=19&w10=coronavirus&w11=vaccination&r=) [(1)](https://www.english-corpora.org/corona/duplicates1.asp?xx=1870&n=1) |  |  |  | to control the disease's spread. * Technical problems derailed Japan's **coronavirus** **vaccination** booking system, compounding frustration over the government's handling of new outbreaks of infections |
| [1872](https://www.english-corpora.org/corona/x4.asp?rs=&t=73000558&ID=20324873327) | [21-05-12 IN](https://www.english-corpora.org/corona/x4.asp?rs=&t=73000558&ID=20324873327) | [theweek.in](https://www.english-corpora.org/corona/x3.asp?node=&p=19&w10=coronavirus&w11=vaccination&r=) |  |  |  | was made open for all senior citizens. In order to ramp up the **coronavirus** **vaccination** drive in the country, the Centre had last month announced a' liberalised and |
| [1873](https://www.english-corpora.org/corona/x4.asp?rs=&t=73001252&ID=20324943169) | [21-05-12 IN](https://www.english-corpora.org/corona/x4.asp?rs=&t=73001252&ID=20324943169) | [punemirror.indiatimes.com](https://www.english-corpora.org/corona/x3.asp?node=&p=19&w10=coronavirus&w11=vaccination&r=) |  |  |  | # It will be used to travel outside France for people showing proof of **coronavirus** **vaccination**, a recent negative test or recovery from a Covid-19 infection. # Opposition parties |
| [1874](https://www.english-corpora.org/corona/x4.asp?rs=&t=73007069&ID=20343106419) | [21-05-13 IN](https://www.english-corpora.org/corona/x4.asp?rs=&t=73007069&ID=20343106419) | [thehansindia.com](https://www.english-corpora.org/corona/x3.asp?node=&p=19&w10=coronavirus&w11=vaccination&r=) |  |  |  | made open for all senior citizens. # In order to ramp up the **coronavirus** **vaccination** drive in the country, the Centre had last month announced a' liberalised and |
| [1877](https://www.english-corpora.org/corona/x4.asp?rs=&t=73018600&ID=20370007844) | [21-05-16 IN](https://www.english-corpora.org/corona/x4.asp?rs=&t=73018600&ID=20370007844) | [in.news.yahoo.com](https://www.english-corpora.org/corona/x3.asp?node=&p=19&w10=coronavirus&w11=vaccination&r=) [(2)](https://www.english-corpora.org/corona/duplicates1.asp?xx=1876&n=2) |  |  |  | the view of COVID-19, the Karnataka government has decided to shift all **coronavirus** **vaccination** centres from hospitals and Primary Health Centre (PHCs) to schools, colleges, |
| [1878](https://www.english-corpora.org/corona/x4.asp?rs=&t=87216142&ID=20331285917) | [21-05-12 IN](https://www.english-corpora.org/corona/x4.asp?rs=&t=87216142&ID=20331285917) | [Indiatimes](https://www.english-corpora.org/corona/x3.asp?node=&p=19&w10=coronavirus&w11=vaccination&r=) |  |  |  | ramp up the **coronavirus** **vaccination** drive in the country, the Indian government on April 19 announced a " liberalised |
| [1879](https://www.english-corpora.org/corona/x4.asp?rs=&t=87216142&ID=20331286100) | [21-05-12 IN](https://www.english-corpora.org/corona/x4.asp?rs=&t=87216142&ID=20331286100) | [Indiatimes](https://www.english-corpora.org/corona/x3.asp?node=&p=19&w10=coronavirus&w11=vaccination&r=) |  |  |  | need to know about the registration process-When can you register? The registration for **coronavirus** **vaccination** began at 4pm on 28 April, 2021. Where can you register for the |
| [1880](https://www.english-corpora.org/corona/x4.asp?rs=&t=87216142&ID=20331286426) | [21-05-12 IN](https://www.english-corpora.org/corona/x4.asp?rs=&t=87216142&ID=20331286426) | [Indiatimes](https://www.english-corpora.org/corona/x3.asp?node=&p=19&w10=coronavirus&w11=vaccination&r=) |  |  |  | date, place and time of vaccination. In order to ramp up the **coronavirus** **vaccination** drive in the country, the Indian government on April 19 announced a " liberalised |
| [1881](https://www.english-corpora.org/corona/x4.asp?rs=&t=87235673&ID=20367769246) | [21-05-15 IN](https://www.english-corpora.org/corona/x4.asp?rs=&t=87235673&ID=20367769246) | [Indiatimes](https://www.english-corpora.org/corona/x3.asp?node=&p=19&w10=coronavirus&w11=vaccination&r=) |  |  |  | 19 had announced a' liberalised' policy in order to ramp up the **coronavirus** **vaccination** drive in the country and made all above 18 years of age eligible to get |
| [1882](https://www.english-corpora.org/corona/x4.asp?rs=&t=87235673&ID=20367769303) | [21-05-15 IN](https://www.english-corpora.org/corona/x4.asp?rs=&t=87235673&ID=20367769303) | [Indiatimes](https://www.english-corpora.org/corona/x3.asp?node=&p=19&w10=coronavirus&w11=vaccination&r=) |  |  |  | years from today. # Here's all you need to know about the **coronavirus** **vaccination** drive Phase 3-As per the health ministry, more than 2.45 crore beneficiaries registered themselves |
| [1883](https://www.english-corpora.org/corona/x4.asp?rs=&t=42995277&ID=20310273149) | [21-05-11 LK](https://www.english-corpora.org/corona/x4.asp?rs=&t=42995277&ID=20310273149) | [colombogazette.com](https://www.english-corpora.org/corona/x3.asp?node=&p=19&w10=coronavirus&w11=vaccination&r=) |  |  |  | capable of breaching vaccine # As Sri Lanka looks to expand the **coronavirus** **vaccination** programe, the United Nations (UN) has warned that vaccination is not a |
| [1884](https://www.english-corpora.org/corona/x4.asp?rs=&t=52730681&ID=20342027718) | [21-05-13 LK](https://www.english-corpora.org/corona/x4.asp?rs=&t=52730681&ID=20342027718) | [nation.lk](https://www.english-corpora.org/corona/x3.asp?node=&p=19&w10=coronavirus&w11=vaccination&r=) |  |  |  | said. # First lady Jill Biden talks to a student who received her **coronavirus** **vaccination** Thursday # Jennifer Garner said two of her three children were getting vaccinated on Tuesday |
| [1885](https://www.english-corpora.org/corona/x4.asp?rs=&t=52717776&ID=20296515488) | [21-05-10 PK](https://www.english-corpora.org/corona/x4.asp?rs=&t=52717776&ID=20296515488) | [brecorder.com](https://www.english-corpora.org/corona/x3.asp?node=&p=19&w10=coronavirus&w11=vaccination&r=) |  |  |  | admitted across the country, it added. # Meanwhile, the NCOC said **coronavirus** **vaccination** centres will remain shut for the first two days on Eidul Fitr. |
| [1887](https://www.english-corpora.org/corona/x4.asp?rs=&t=52721810&ID=20304962154) | [21-05-11 PK](https://www.english-corpora.org/corona/x4.asp?rs=&t=52721810&ID=20304962154) | [pakistantoday.com.pk](https://www.english-corpora.org/corona/x3.asp?node=&p=19&w10=coronavirus&w11=vaccination&r=) [(1)](https://www.english-corpora.org/corona/duplicates1.asp?xx=1886&n=1) |  |  |  | Minister Dr Azra Pechuho hinted at turning the Karachi Expo Centre into a **coronavirus** **vaccination** centre. She had also visited the centre to review the proposal and get a |
| [1888](https://www.english-corpora.org/corona/x4.asp?rs=&t=52730588&ID=20342019557) | [21-05-13 PK](https://www.english-corpora.org/corona/x4.asp?rs=&t=52730588&ID=20342019557) | [brecorder.com](https://www.english-corpora.org/corona/x3.asp?node=&p=19&w10=coronavirus&w11=vaccination&r=) |  |  |  | " he tweeted. # On May 3, Pakistan opened registration for the **coronavirus** **vaccination** for people aged between 40 to 49 years across the country. # The vaccine |
| [1889](https://www.english-corpora.org/corona/x4.asp?rs=&t=52734239&ID=20350607520) | [21-05-14 PK](https://www.english-corpora.org/corona/x4.asp?rs=&t=52734239&ID=20350607520) | [samaa.tv](https://www.english-corpora.org/corona/x3.asp?node=&p=19&w10=coronavirus&w11=vaccination&r=) |  |  |  | in Samanabad, Faisalabad. Photo: Online # Listen to the story # **Coronavirus** **vaccination** centres will remain open across the country on Saturday and Sunday, Federal Minister Asad |
| [1890](https://www.english-corpora.org/corona/x4.asp?rs=&t=52734239&ID=20350607598) | [21-05-14 PK](https://www.english-corpora.org/corona/x4.asp?rs=&t=52734239&ID=20350607598) | [samaa.tv](https://www.english-corpora.org/corona/x3.asp?node=&p=19&w10=coronavirus&w11=vaccination&r=) |  |  |  | however, to continue vaccinating people during Eid holidays. Pakistan formally launched the **coronavirus** **vaccination** drive on February 3 as the country continues to battle the virus. The country |
| [1891](https://www.english-corpora.org/corona/x4.asp?rs=&t=52734239&ID=20350607625) | [21-05-14 PK](https://www.english-corpora.org/corona/x4.asp?rs=&t=52734239&ID=20350607625) | [samaa.tv](https://www.english-corpora.org/corona/x3.asp?node=&p=19&w10=coronavirus&w11=vaccination&r=) |  |  |  | virus. The country has so far vaccinated over 3.8 million people. # **Coronavirus** **vaccination** centres will remain open across the country on Saturday and Sunday, Federal Minister Asad |
| [1892](https://www.english-corpora.org/corona/x4.asp?rs=&t=52734239&ID=20350607706) | [21-05-14 PK](https://www.english-corpora.org/corona/x4.asp?rs=&t=52734239&ID=20350607706) | [samaa.tv](https://www.english-corpora.org/corona/x3.asp?node=&p=19&w10=coronavirus&w11=vaccination&r=) |  |  |  | , to continue vaccinating people during Eid holidays. # Pakistan formally launched the **coronavirus** **vaccination** drive on February 3 as the country continues to battle the virus. The country |
| [1893](https://www.english-corpora.org/corona/x4.asp?rs=&t=52736801&ID=20359746313) | [21-05-15 PK](https://www.english-corpora.org/corona/x4.asp?rs=&t=52736801&ID=20359746313) | [samaa.tv](https://www.english-corpora.org/corona/x3.asp?node=&p=19&w10=coronavirus&w11=vaccination&r=) |  |  |  | Shops allowed to stay open till 8pm # 70% passengers in trains # For **coronavirus** **vaccination**, first register at 1166, then visit the centre # The NCOC will meet |
| [1894](https://www.english-corpora.org/corona/x4.asp?rs=&t=52739676&ID=20372340211) | [21-05-16 PK](https://www.english-corpora.org/corona/x4.asp?rs=&t=52739676&ID=20372340211) | [samaa.tv](https://www.english-corpora.org/corona/x3.asp?node=&p=19&w10=coronavirus&w11=vaccination&r=) |  |  |  | . # The World Bank has decided to give Pakistan $153 million for the **coronavirus** **vaccination** drive. # Pakistanis are celebrating the third day of Eid-ul-Fitr indoors. # The |
| [1895](https://www.english-corpora.org/corona/x4.asp?rs=&t=43011614&ID=20369279000) | [21-05-16 MY](https://www.english-corpora.org/corona/x4.asp?rs=&t=43011614&ID=20369279000) | [thestar.com.my](https://www.english-corpora.org/corona/x3.asp?node=&p=19&w10=coronavirus&w11=vaccination&r=) |  |  |  | a statement. # A national committee in charge of monitoring effects of novel **coronavirus** **vaccination** launched an investigation earlier this month after a 22-year-old man in Jakarta died a day |
| [1896](https://www.english-corpora.org/corona/x4.asp?rs=&t=72997241&ID=20308824363) | [21-05-11 PH](https://www.english-corpora.org/corona/x4.asp?rs=&t=72997241&ID=20308824363) | [ph.news.yahoo.com](https://www.english-corpora.org/corona/x3.asp?node=&p=19&w10=coronavirus&w11=vaccination&r=) |  |  |  | to Pregnancy, Baby & Raising Kids. # Technical problems derailed Japan's **coronavirus** **vaccination** booking system on Wednesday, compounding frustration over the government's handling of new outbreaks |
| [1897](https://www.english-corpora.org/corona/x4.asp?rs=&t=73001614&ID=20320920906) | [21-05-12 PH](https://www.english-corpora.org/corona/x4.asp?rs=&t=73001614&ID=20320920906) | [bworldonline.com](https://www.english-corpora.org/corona/x3.asp?node=&p=19&w10=coronavirus&w11=vaccination&r=) |  |  |  | the Straits Times' Indonesian Correspondent Wahyudi Soeriaatmadja reported that " Indonesia has made **coronavirus** **vaccination** for citizens compulsory, " and that " people who are eligible for vaccination but |
| [1898](https://www.english-corpora.org/corona/x4.asp?rs=&t=73008194&ID=20343225459) | [21-05-13 PH](https://www.english-corpora.org/corona/x4.asp?rs=&t=73008194&ID=20343225459) | [cnnphilippines.com](https://www.english-corpora.org/corona/x3.asp?node=&p=19&w10=coronavirus&w11=vaccination&r=) |  |  |  | also demonstrated that being fully vaccinated matters. # The first nationwide study of **coronavirus** **vaccination**, done in Israel, showed Pfizer/BioNtech's vaccine works far better after two doses |
| [1899](https://www.english-corpora.org/corona/x4.asp?rs=&t=62920574&ID=20342623311) | [21-05-13 ZA](https://www.english-corpora.org/corona/x4.asp?rs=&t=62920574&ID=20342623311) | [cnbcafrica.com](https://www.english-corpora.org/corona/x3.asp?node=&p=19&w10=coronavirus&w11=vaccination&r=) |  |  |  | coronavirus infections, the Nikkei newspaper reported. # Technical problems derailed Japan's **coronavirus** **vaccination** booking system, compounding frustration over the government's handling of new outbreaks of infections |
| [1900](https://www.english-corpora.org/corona/x4.asp?rs=&t=87215575&ID=20333123573) | [21-05-12 --](https://www.english-corpora.org/corona/x4.asp?rs=&t=87215575&ID=20333123573) | [Express & Star](https://www.english-corpora.org/corona/x3.asp?node=&p=19&w10=coronavirus&w11=vaccination&r=) |  |  |  | ! # Royal Navy personnel prepare to give vaccines to the public at a **coronavirus** **vaccination** centre set up at Bath Racecourse (Ben Birchall/PA) # People may be able |

| PAGE:   [**<<**](https://www.english-corpora.org/corona/x3.asp?node=&p=1&w10=coronavirus&w11=vaccination&r=)  [**<**](https://www.english-corpora.org/corona/x3.asp?node=&p=18&w10=coronavirus&w11=vaccination&r=)   19 / 42  [**>**](https://www.english-corpora.org/corona/x3.asp?node=&p=20&w10=coronavirus&w11=vaccination&r=)   [**>>**](https://www.english-corpora.org/corona/x3.asp?node=&p=42&w10=coronavirus&w11=vaccination&r=) | | |
| --- | --- | --- |
| PAGE:   [**<<**](https://www.english-corpora.org/corona/x3.asp?node=&p=1&w10=coronavirus&w11=vaccination&r=)  [**<**](https://www.english-corpora.org/corona/x3.asp?node=&p=19&w10=coronavirus&w11=vaccination&r=)   20 / 42  [**>**](https://www.english-corpora.org/corona/x3.asp?node=&p=21&w10=coronavirus&w11=vaccination&r=)   [**>>**](https://www.english-corpora.org/corona/x3.asp?node=&p=42&w10=coronavirus&w11=vaccination&r=) |  |  |

Начало формы

| CLICK FOR MORE CONTEXT | [**HELP**](javascript:newFeatures()) | [SAVE](javascript:chooser('s'))    [TRANSLATE](javascript:chooser('t'))    [ANALYZE](javascript:chooser('p')) |
| --- | --- | --- |

| [1901](https://www.english-corpora.org/corona/x4.asp?rs=&t=32766541&ID=20107430609) | [21-04-24 US](https://www.english-corpora.org/corona/x4.asp?rs=&t=32766541&ID=20107430609) | [nytimes.com](https://www.english-corpora.org/corona/x3.asp?node=&p=20&w10=coronavirus&w11=vaccination&r=) |  |  |  | demand for hiring, " she said. # Warmer weather, more extensive **coronavirus** **vaccination** efforts and a stream of government assistance that has enabled consumer spending have all contributed |
| --- | --- | --- | --- | --- | --- | --- |
| [1902](https://www.english-corpora.org/corona/x4.asp?rs=&t=32766706&ID=20104882837) | [21-04-24 US](https://www.english-corpora.org/corona/x4.asp?rs=&t=32766706&ID=20104882837) | [mercurynews.com](https://www.english-corpora.org/corona/x3.asp?node=&p=20&w10=coronavirus&w11=vaccination&r=) |  |  |  | # # NEW YORK -- Appointments are no longer necessary at any of the **coronavirus** **vaccination** sites run by New York City. # Mayor Bill de Blasio announced anyone eligible |
| [1903](https://www.english-corpora.org/corona/x4.asp?rs=&t=32767444&ID=20107648287) | [21-04-24 US](https://www.english-corpora.org/corona/x4.asp?rs=&t=32767444&ID=20107648287) | [jpost.com](https://www.english-corpora.org/corona/x3.asp?node=&p=20&w10=coronavirus&w11=vaccination&r=) |  |  |  | fully vaccinated or has recovered from COVID-19. # Throughout March, Israel's **coronavirus** **vaccination** rate remains one of the highest in the world. Nearly 60% of the state |
| [1904](https://www.english-corpora.org/corona/x4.asp?rs=&t=42952669&ID=20142437780) | [21-04-27 US](https://www.english-corpora.org/corona/x4.asp?rs=&t=42952669&ID=20142437780) | [computerworld.com](https://www.english-corpora.org/corona/x3.asp?node=&p=20&w10=coronavirus&w11=vaccination&r=) |  |  |  | want serious advice on how to handle employees who refuse to get with the **coronavirus** **vaccination** program, it's time to find an employment law-savvy attorney. # Personally, |
| [1905](https://www.english-corpora.org/corona/x4.asp?rs=&t=42963803&ID=20170282819) | [21-04-30 US](https://www.english-corpora.org/corona/x4.asp?rs=&t=42963803&ID=20170282819) | [talkingpointsmemo.com](https://www.english-corpora.org/corona/x3.asp?node=&p=20&w10=coronavirus&w11=vaccination&r=) |  |  |  | illness nationally, the analysis found no relationship between counties' racial demographics and **coronavirus** **vaccination** rates. # The rollout has largely relied less on government outreach than on individual |
| [1906](https://www.english-corpora.org/corona/x4.asp?rs=&t=52669846&ID=20098878422) | [21-04-23 US](https://www.english-corpora.org/corona/x4.asp?rs=&t=52669846&ID=20098878422) | [wpxi.com](https://www.english-corpora.org/corona/x3.asp?node=&p=20&w10=coronavirus&w11=vaccination&r=) |  |  |  | wrapped up a three-day tour of the U.S. Southwest, where she stopped at **coronavirus** **vaccination** clinics in New Mexico and Arizona and met with female tribal leaders who shared their |
| [1907](https://www.english-corpora.org/corona/x4.asp?rs=&t=52670423&ID=20096377928) | [21-04-23 US](https://www.english-corpora.org/corona/x4.asp?rs=&t=52670423&ID=20096377928) | [wsoctv.com](https://www.english-corpora.org/corona/x3.asp?node=&p=20&w10=coronavirus&w11=vaccination&r=) |  |  |  | April 23, 2021. Appointments are no longer necessary at any of the **coronavirus** **vaccination** sites run by New York City. New York City Mayor Bill de Blasio announced |
| [1908](https://www.english-corpora.org/corona/x4.asp?rs=&t=52687335&ID=20162758180) | [21-04-29 US](https://www.english-corpora.org/corona/x4.asp?rs=&t=52687335&ID=20162758180) | [dailyprogress.com](https://www.english-corpora.org/corona/x3.asp?node=&p=20&w10=coronavirus&w11=vaccination&r=) |  |  |  | hospitalizations. # All state residents over age 16 have been eligible for a **coronavirus** **vaccination** since April 15. As of Thursday, more than 5.2 million doses of vaccine |
| [1909](https://www.english-corpora.org/corona/x4.asp?rs=&t=52689699&ID=20170679964) | [21-04-30 US](https://www.english-corpora.org/corona/x4.asp?rs=&t=52689699&ID=20170679964) | [mynorthwest.com](https://www.english-corpora.org/corona/x3.asp?node=&p=20&w10=coronavirus&w11=vaccination&r=) |  |  |  | reach 1,000 of the islands' most vulnerable residents. # Uganda started its **coronavirus** **vaccination** campaign in mid-March, targeting more than 20 million people at the highest risk of |
| [1911](https://www.english-corpora.org/corona/x4.asp?rs=&t=72939759&ID=20099817083) | [21-04-23 US](https://www.english-corpora.org/corona/x4.asp?rs=&t=72939759&ID=20099817083) | [pe.com](https://www.english-corpora.org/corona/x3.asp?node=&p=20&w10=coronavirus&w11=vaccination&r=) [(1)](https://www.english-corpora.org/corona/duplicates1.asp?xx=1910&n=1) |  |  |  | 72939759 # Riverside County residents no longer need an appointment to receive a **coronavirus** **vaccination** from a county-run clinic. # " We were finding that our appointments were leveling |
| [1912](https://www.english-corpora.org/corona/x4.asp?rs=&t=72940540&ID=20099948307) | [21-04-23 US](https://www.english-corpora.org/corona/x4.asp?rs=&t=72940540&ID=20099948307) | [adn.com](https://www.english-corpora.org/corona/x3.asp?node=&p=20&w10=coronavirus&w11=vaccination&r=) |  |  |  | 72940540 # WASHINGTON - Pregnant women confused by conflicting recommendations regarding **coronavirus** **vaccination** over the past few months now have clear guidance from the Centers for Disease Control |
| [1913](https://www.english-corpora.org/corona/x4.asp?rs=&t=72957019&ID=20150722804) | [21-04-28 US](https://www.english-corpora.org/corona/x4.asp?rs=&t=72957019&ID=20150722804) | [thesouthern.com](https://www.english-corpora.org/corona/x3.asp?node=&p=20&w10=coronavirus&w11=vaccination&r=) |  |  |  | April 23, 2021. Appointments are no longer necessary at any of the **coronavirus** **vaccination** sites run by New York City. New York City Mayor Bill de Blasio announced |
| [1915](https://www.english-corpora.org/corona/x4.asp?rs=&t=87112521&ID=20112326228) | [21-04-24 US](https://www.english-corpora.org/corona/x4.asp?rs=&t=87112521&ID=20112326228) | [YAHOO!News](https://www.english-corpora.org/corona/x3.asp?node=&p=20&w10=coronavirus&w11=vaccination&r=) [(1)](https://www.english-corpora.org/corona/duplicates1.asp?xx=1914&n=1) |  |  |  | up a three-day tour of the U.S. Southwest, where she stopped at **coronavirus** **vaccination** clinics in New Mexico and Arizona and met with female tribal leaders who shared their |
| [1916](https://www.english-corpora.org/corona/x4.asp?rs=&t=87114312&ID=20112756970) | [21-04-24 US](https://www.english-corpora.org/corona/x4.asp?rs=&t=87114312&ID=20112756970) | [YAHOO!News](https://www.english-corpora.org/corona/x3.asp?node=&p=20&w10=coronavirus&w11=vaccination&r=) |  |  |  | has a pretty... # Appointments are no longer mandatory at any of the **coronavirus** **vaccination** sites run by New York City, including its newest and maybe coolest location: |
| [1917](https://www.english-corpora.org/corona/x4.asp?rs=&t=87115954&ID=20114349728) | [21-04-24 US](https://www.english-corpora.org/corona/x4.asp?rs=&t=87115954&ID=20114349728) | [The Motley Fool](https://www.english-corpora.org/corona/x3.asp?node=&p=20&w10=coronavirus&w11=vaccination&r=) |  |  |  | course, many employers are already offering workers paid time off to get a **coronavirus** **vaccination**. In fact, Dollar General, Aldi, and Trader Joe's were among |
| [1918](https://www.english-corpora.org/corona/x4.asp?rs=&t=87125453&ID=20133192817) | [21-04-26 US](https://www.english-corpora.org/corona/x4.asp?rs=&t=87125453&ID=20133192817) | [Forbes](https://www.english-corpora.org/corona/x3.asp?node=&p=20&w10=coronavirus&w11=vaccination&r=) |  |  |  | one of the most successful vaccine rollouts in the world, the U.S.'s **coronavirus** **vaccination** rate has been on a downwards trend since mid-April, with officials sounding the alarm |
| [1919](https://www.english-corpora.org/corona/x4.asp?rs=&t=87126889&ID=20135719582) | [21-04-26 US](https://www.english-corpora.org/corona/x4.asp?rs=&t=87126889&ID=20135719582) | [NBC News](https://www.english-corpora.org/corona/x3.asp?node=&p=20&w10=coronavirus&w11=vaccination&r=) |  |  |  | not get an annual flu shot, so he wondered, why take the **coronavirus** **vaccination**? He understood Covid-19 was far more contagious and deadly, but as someone who |
| [1920](https://www.english-corpora.org/corona/x4.asp?rs=&t=87127892&ID=20134780213) | [21-04-26 US](https://www.english-corpora.org/corona/x4.asp?rs=&t=87127892&ID=20134780213) | [Washington Times](https://www.english-corpora.org/corona/x3.asp?node=&p=20&w10=coronavirus&w11=vaccination&r=) |  |  |  | Bowser announced Monday that the city will switch to walk-up **coronavirus** **vaccination** sites and ease certain restrictions next month. # Beginning May 1, the mayor |
| [1921](https://www.english-corpora.org/corona/x4.asp?rs=&t=87135622&ID=20156188341) | [21-04-28 US](https://www.english-corpora.org/corona/x4.asp?rs=&t=87135622&ID=20156188341) | [The Hill](https://www.english-corpora.org/corona/x3.asp?node=&p=20&w10=coronavirus&w11=vaccination&r=) |  |  |  | from podcaster Joe Rogan who suggested young, healthy Americans should not receive a **coronavirus** **vaccination**. # " You're talking about yourself in a vacuum, " Fauci said |
| [1922](https://www.english-corpora.org/corona/x4.asp?rs=&t=87144123&ID=20165495094) | [21-04-29 US](https://www.english-corpora.org/corona/x4.asp?rs=&t=87144123&ID=20165495094) | [YAHOO!News](https://www.english-corpora.org/corona/x3.asp?node=&p=20&w10=coronavirus&w11=vaccination&r=) |  |  |  | arms. " All state residents over age 16 have be eligible for a **coronavirus** **vaccination** since April 15. # Secretary of State Antony Blinken and other top Biden administration |
| [1923](https://www.english-corpora.org/corona/x4.asp?rs=&t=87147332&ID=20165705166) | [21-04-29 US](https://www.english-corpora.org/corona/x4.asp?rs=&t=87147332&ID=20165705166) | [SFGate](https://www.english-corpora.org/corona/x3.asp?node=&p=20&w10=coronavirus&w11=vaccination&r=) |  |  |  | . " # All state residents over age 16 have be eligible for a **coronavirus** **vaccination** since April 15. As of Thursday, more than 5.2 million doses of vaccine |
| [1924](https://www.english-corpora.org/corona/x4.asp?rs=&t=87149345&ID=20177615287) | [21-04-30 US](https://www.english-corpora.org/corona/x4.asp?rs=&t=87149345&ID=20177615287) | [The Baltimore Sun](https://www.english-corpora.org/corona/x3.asp?node=&p=20&w10=coronavirus&w11=vaccination&r=) |  |  |  | Kamala Harris visited Baltimore to tout the nation's -- and Maryland's -- **coronavirus** **vaccination** campaign during her and President Joe Biden's first 100 days in office, the |
| [1926](https://www.english-corpora.org/corona/x4.asp?rs=&t=87151258&ID=20178422608) | [21-04-30 US](https://www.english-corpora.org/corona/x4.asp?rs=&t=87151258&ID=20178422608) | [ESPN](https://www.english-corpora.org/corona/x3.asp?node=&p=20&w10=coronavirus&w11=vaccination&r=) [(1)](https://www.english-corpora.org/corona/duplicates1.asp?xx=1925&n=1) |  |  |  | there. " # Snead said that he has received one of two **coronavirus** **vaccination** doses. # Despite not owning a first-round pick, the Rams made a splash |
| [1927](https://www.english-corpora.org/corona/x4.asp?rs=&t=87151684&ID=20178965643) | [21-04-30 US](https://www.english-corpora.org/corona/x4.asp?rs=&t=87151684&ID=20178965643) | [The San Bernardino Sun](https://www.english-corpora.org/corona/x3.asp?node=&p=20&w10=coronavirus&w11=vaccination&r=) |  |  |  | the already existing pent-up demand of U.S. households, another lies in the increased **coronavirus** **vaccination** rates not only in California but elsewhere in the nation, and finally the $1.9 |
| [1928](https://www.english-corpora.org/corona/x4.asp?rs=&t=87153375&ID=20179034144) | [21-04-30 US](https://www.english-corpora.org/corona/x4.asp?rs=&t=87153375&ID=20179034144) | [Medscape](https://www.english-corpora.org/corona/x3.asp?node=&p=20&w10=coronavirus&w11=vaccination&r=) |  |  |  | recovered from COVID-19 or have been fully vaccinated with proposals expected next week. **Coronavirus** **vaccination** should be possible for everyone in Germany by June at the latest. Figures released |
| [1929](https://www.english-corpora.org/corona/x4.asp?rs=&t=87153913&ID=20176433749) | [21-04-30 US](https://www.english-corpora.org/corona/x4.asp?rs=&t=87153913&ID=20176433749) | [Today](https://www.english-corpora.org/corona/x3.asp?node=&p=20&w10=coronavirus&w11=vaccination&r=) |  |  |  | answered some of the most common questions about COVID-19 vaccine cards. # Your **coronavirus** **vaccination** card includes personal information like your full name and birthday, and information about where |
| [1930](https://www.english-corpora.org/corona/x4.asp?rs=&t=52665997&ID=20100633290) | [21-04-23 CA](https://www.english-corpora.org/corona/x4.asp?rs=&t=52665997&ID=20100633290) | [jamaica-gleaner.com](https://www.english-corpora.org/corona/x3.asp?node=&p=20&w10=coronavirus&w11=vaccination&r=) |  |  |  | the coronavirus walks by masked residents lining up for the COVID-19 vaccine at a **coronavirus** **vaccination** site in China. # BEIJING (AP) -- Around 200 million Chinese, |
| [1931](https://www.english-corpora.org/corona/x4.asp?rs=&t=52688389&ID=20174748634) | [21-04-30 CA](https://www.english-corpora.org/corona/x4.asp?rs=&t=52688389&ID=20174748634) | [570news.com](https://www.english-corpora.org/corona/x3.asp?node=&p=20&w10=coronavirus&w11=vaccination&r=) |  |  |  | . " # All state residents over age 16 have be eligible for a **coronavirus** **vaccination** since April 15. As of Thursday, more than 5.2 million doses of vaccine |
| [1932](https://www.english-corpora.org/corona/x4.asp?rs=&t=42957372&ID=20163708130) | [21-04-29 GB](https://www.english-corpora.org/corona/x4.asp?rs=&t=42957372&ID=20163708130) | [inews.co.uk](https://www.english-corpora.org/corona/x3.asp?node=&p=20&w10=coronavirus&w11=vaccination&r=) |  |  |  | last summer about his intention to step down but wanted to see through the **coronavirus** **vaccination** programme. # Following the announcement, Lord Prior said the country owed Sir Simon |
| [1933](https://www.english-corpora.org/corona/x4.asp?rs=&t=42960244&ID=20171934066) | [21-04-30 GB](https://www.english-corpora.org/corona/x4.asp?rs=&t=42960244&ID=20171934066) | [manchestereveningnews.co.uk](https://www.english-corpora.org/corona/x3.asp?node=&p=20&w10=coronavirus&w11=vaccination&r=) |  |  |  | Covid-19 vaccinations have given vital protection to vulnerable people. # Since the first **coronavirus** **vaccination** session held on March 3 some 44 service users have been vaccinated at hostels, |
| [1934](https://www.english-corpora.org/corona/x4.asp?rs=&t=72945039&ID=20118364978) | [21-04-25 AU](https://www.english-corpora.org/corona/x4.asp?rs=&t=72945039&ID=20118364978) | [honey.nine.com.au](https://www.english-corpora.org/corona/x3.asp?node=&p=20&w10=coronavirus&w11=vaccination&r=) |  |  |  | landed. # It is unknown whether the Duke of Sussex has received a **coronavirus** **vaccination**, which would excuse him from self-isolation. # The Queen celebrated her birthday in |
| [1935](https://www.english-corpora.org/corona/x4.asp?rs=&t=72947048&ID=20129132803) | [21-04-26 AU](https://www.english-corpora.org/corona/x4.asp?rs=&t=72947048&ID=20129132803) | [brisbanetimes.com.au](https://www.english-corpora.org/corona/x3.asp?node=&p=20&w10=coronavirus&w11=vaccination&r=) |  |  |  | are too frail to travel to mass **coronavirus** **vaccination** hubs are being put on vaccine waiting lists stretching out to September by their general |
| [1936](https://www.english-corpora.org/corona/x4.asp?rs=&t=72954252&ID=20152825007) | [21-04-28 AU](https://www.english-corpora.org/corona/x4.asp?rs=&t=72954252&ID=20152825007) | [illawarramercury.com.au](https://www.english-corpora.org/corona/x3.asp?node=&p=20&w10=coronavirus&w11=vaccination&r=) |  |  |  | jump to conclusions amid reports of a man dying eight days after receiving a **coronavirus** **vaccination**. Chief Nursing Officer Alison McMillan has moved to assure the public robust processes are |
| [1937](https://www.english-corpora.org/corona/x4.asp?rs=&t=52673046&ID=20117969566) | [21-04-25 IN](https://www.english-corpora.org/corona/x4.asp?rs=&t=52673046&ID=20117969566) | [boldsky.com](https://www.english-corpora.org/corona/x3.asp?node=&p=20&w10=coronavirus&w11=vaccination&r=) |  |  |  | conditions. # As per the recent announcement by the Indian government, the **coronavirus** **vaccination** drive will be accelerated to phase 3 in which people over 18 will be vaccinated |
| [1938](https://www.english-corpora.org/corona/x4.asp?rs=&t=72942047&ID=20106902910) | [21-04-24 IN](https://www.english-corpora.org/corona/x4.asp?rs=&t=72942047&ID=20106902910) | [deccanchronicle.com](https://www.english-corpora.org/corona/x3.asp?node=&p=20&w10=coronavirus&w11=vaccination&r=) |  |  |  | TelanganaCMO) # HYDERABAD: Chief Minister K. Chandrashekar Rao on Saturday announced that **Coronavirus** **vaccination** will be done on the entire population in the state free of cost and irrespective |
| [1940](https://www.english-corpora.org/corona/x4.asp?rs=&t=72945125&ID=20117206282) | [21-04-25 IN](https://www.english-corpora.org/corona/x4.asp?rs=&t=72945125&ID=20117206282) | [theweek.in](https://www.english-corpora.org/corona/x3.asp?node=&p=20&w10=coronavirus&w11=vaccination&r=) [(1)](https://www.english-corpora.org/corona/duplicates1.asp?xx=1939&n=1) |  |  |  | , no reason to complain now: Vardhan # The Centre liberalised the **coronavirus** **vaccination** strategy to allow states, private hospitals and industrial establishments following requests from nearly all |
| [1941](https://www.english-corpora.org/corona/x4.asp?rs=&t=72948031&ID=20126841079) | [21-04-26 IN](https://www.english-corpora.org/corona/x4.asp?rs=&t=72948031&ID=20126841079) | [cnbctv18.com](https://www.english-corpora.org/corona/x3.asp?node=&p=20&w10=coronavirus&w11=vaccination&r=) |  |  |  | doses. # The list comes as the inoculation drive at a number of **coronavirus** **vaccination** centres across Mumbai including the Bandra Kurla Complex (BKC) jumbo vaccination centre has |
| [1942](https://www.english-corpora.org/corona/x4.asp?rs=&t=72949452&ID=20129568059) | [21-04-26 IN](https://www.english-corpora.org/corona/x4.asp?rs=&t=72949452&ID=20129568059) | [businesstoday.in](https://www.english-corpora.org/corona/x3.asp?node=&p=20&w10=coronavirus&w11=vaccination&r=) |  |  |  | . # Both vaccines are the only ones currently being used in India's **coronavirus** **vaccination** drive and are available to the central government at Rs 150 per dose. The |
| [1943](https://www.english-corpora.org/corona/x4.asp?rs=&t=72951777&ID=20144532227) | [21-04-27 IN](https://www.english-corpora.org/corona/x4.asp?rs=&t=72951777&ID=20144532227) | [bgr.in](https://www.english-corpora.org/corona/x3.asp?node=&p=20&w10=coronavirus&w11=vaccination&r=) |  |  |  | for COVID-19 vaccine using www.cowin.gov.in and Aarogya Setu. # The second phase of **coronavirus** **vaccination** begins in India from March 1, 2021. PM Narendra Modi is the first |
| [1944](https://www.english-corpora.org/corona/x4.asp?rs=&t=72959473&ID=20165097775) | [21-04-29 IN](https://www.english-corpora.org/corona/x4.asp?rs=&t=72959473&ID=20165097775) | [zeebiz.com](https://www.english-corpora.org/corona/x3.asp?node=&p=20&w10=coronavirus&w11=vaccination&r=) |  |  |  | this is happening and when people will be able to book appointments for the **coronavirus** **vaccination**. # The central government earlier this month has allowed everyone above 18 years of |
| [1945](https://www.english-corpora.org/corona/x4.asp?rs=&t=72962043&ID=20173707598) | [21-04-30 IN](https://www.english-corpora.org/corona/x4.asp?rs=&t=72962043&ID=20173707598) | [bangaloremirror.indiatimes.com](https://www.english-corpora.org/corona/x3.asp?node=&p=20&w10=coronavirus&w11=vaccination&r=) |  |  |  | confirms the arrival of vaccines. # Registration for the third phase of the **coronavirus** **vaccination** drive is all set to begin today. People over 18 years of age will |
| [1946](https://www.english-corpora.org/corona/x4.asp?rs=&t=32769353&ID=20108069292) | [21-04-24 PK](https://www.english-corpora.org/corona/x4.asp?rs=&t=32769353&ID=20108069292) | [geo.tv](https://www.english-corpora.org/corona/x3.asp?node=&p=20&w10=coronavirus&w11=vaccination&r=) |  |  |  | says. # KARACHI: Information Minister Fawad Chaudhry claimed on Saturday Pakistan's **coronavirus** **vaccination** plan was better than the one being implemented in Europe, but did not explain |
| [1947](https://www.english-corpora.org/corona/x4.asp?rs=&t=52683915&ID=20149223101) | [21-04-28 PK](https://www.english-corpora.org/corona/x4.asp?rs=&t=52683915&ID=20149223101) | [pakistantoday.com.pk](https://www.english-corpora.org/corona/x3.asp?node=&p=20&w10=coronavirus&w11=vaccination&r=) |  |  |  | ... 52683915 # LAHORE: Lahore's largest **coronavirus** **vaccination** centre remained closed during vaccination hours without prior notice on Wednesday which led to citizens |
| [1948](https://www.english-corpora.org/corona/x4.asp?rs=&t=52683915&ID=20149223242) | [21-04-28 PK](https://www.english-corpora.org/corona/x4.asp?rs=&t=52683915&ID=20149223242) | [pakistantoday.com.pk](https://www.english-corpora.org/corona/x3.asp?node=&p=20&w10=coronavirus&w11=vaccination&r=) |  |  |  | not been changed. # It is worth mentioning here that the government started **coronavirus** **vaccination** for citizens aged 40 and above on Tuesday as the country continues to grapple with |
| [1949](https://www.english-corpora.org/corona/x4.asp?rs=&t=42950966&ID=20139220737) | [21-04-27 MY](https://www.english-corpora.org/corona/x4.asp?rs=&t=42950966&ID=20139220737) | [malaymail.com](https://www.english-corpora.org/corona/x3.asp?node=&p=20&w10=coronavirus&w11=vaccination&r=) |  |  |  | may be taking profits on equities, but sentiment remains positive due to rising **coronavirus** **vaccination** rates in many countries. # " There are two reasons to remain positive on |
| [1950](https://www.english-corpora.org/corona/x4.asp?rs=&t=42951144&ID=20139239553) | [21-04-27 MY](https://www.english-corpora.org/corona/x4.asp?rs=&t=42951144&ID=20139239553) | [theedgemarkets.com](https://www.english-corpora.org/corona/x3.asp?node=&p=20&w10=coronavirus&w11=vaccination&r=) |  |  |  | Powell is likely to face questions over whether an improving labour market and rising **coronavirus** **vaccination** warrant a withdrawal of monetary easing. # " Most analysts though expect him to |
| [1951](https://www.english-corpora.org/corona/x4.asp?rs=&t=72962553&ID=20173776964) | [21-04-30 MY](https://www.english-corpora.org/corona/x4.asp?rs=&t=72962553&ID=20173776964) | [khmertimeskh.com](https://www.english-corpora.org/corona/x3.asp?node=&p=20&w10=coronavirus&w11=vaccination&r=) |  |  |  | man -- three days short of his 60th birthday -- shows up to a **coronavirus** **vaccination** center. It's a Wednesday. He says he would like to get an |
| [1952](https://www.english-corpora.org/corona/x4.asp?rs=&t=42946229&ID=20124892706) | [21-04-26 SG](https://www.english-corpora.org/corona/x4.asp?rs=&t=42946229&ID=20124892706) | [thenewsminute.com](https://www.english-corpora.org/corona/x3.asp?node=&p=20&w10=coronavirus&w11=vaccination&r=) |  |  |  | last-mile delivery for the entire population. # In order to ramp up the **coronavirus** **vaccination** drive in the country, the Indian government on Monday announced a liberalised and accelerated |
| [1953](https://www.english-corpora.org/corona/x4.asp?rs=&t=42951711&ID=20136266625) | [21-04-27 SG](https://www.english-corpora.org/corona/x4.asp?rs=&t=42951711&ID=20136266625) | [ucanews.com](https://www.english-corpora.org/corona/x3.asp?node=&p=20&w10=coronavirus&w11=vaccination&r=) |  |  |  | are now signed up to Daily newsletter # People over 40 can register for **coronavirus** **vaccination** starting April 27, Asad Umar, who heads the National Command Operation Center ( |
| [1954](https://www.english-corpora.org/corona/x4.asp?rs=&t=42961331&ID=20169821135) | [21-04-30 HK](https://www.english-corpora.org/corona/x4.asp?rs=&t=42961331&ID=20169821135) | [nepal24hours.com](https://www.english-corpora.org/corona/x3.asp?node=&p=20&w10=coronavirus&w11=vaccination&r=) |  |  |  | 's first 100 days in office. # The gains have come as large-scale **coronavirus** **vaccination** programs help people return to jobs and normal behaviors after more than a year of |
| [1955](https://www.english-corpora.org/corona/x4.asp?rs=&t=32790746&ID=20193355060) | [21-05-02 US](https://www.english-corpora.org/corona/x4.asp?rs=&t=32790746&ID=20193355060) | [pilotonline.com](https://www.english-corpora.org/corona/x3.asp?node=&p=20&w10=coronavirus&w11=vaccination&r=) |  |  |  | Circle in Norfolk. (Kristen Zeis) # NORFOLK -- The federally run **coronavirus** **vaccination** clinic based at Military Circle mall had given 50,000 shots to people throughout Hampton Roads |
| [1956](https://www.english-corpora.org/corona/x4.asp?rs=&t=32798231&ID=20215455618) | [21-05-04 US](https://www.english-corpora.org/corona/x4.asp?rs=&t=32798231&ID=20215455618) | [twincities.com](https://www.english-corpora.org/corona/x3.asp?node=&p=20&w10=coronavirus&w11=vaccination&r=) |  |  |  | . # Data from the Centers for Disease Control and Prevention shows Louisiana's **coronavirus** **vaccination** rate is well behind most states. About 27% of state residents are fully vaccinated |
| [1957](https://www.english-corpora.org/corona/x4.asp?rs=&t=32798372&ID=20222003597) | [21-05-04 US](https://www.english-corpora.org/corona/x4.asp?rs=&t=32798372&ID=20222003597) | [nydailynews.com](https://www.english-corpora.org/corona/x3.asp?node=&p=20&w10=coronavirus&w11=vaccination&r=) |  |  |  | of U.S. adults by July 4 # President Biden is shooting for a new **coronavirus** **vaccination** goal of administering at least one dose to 70% of American adults by July 4 |
| [1958](https://www.english-corpora.org/corona/x4.asp?rs=&t=32803329&ID=20230843122) | [21-05-05 US](https://www.english-corpora.org/corona/x4.asp?rs=&t=32803329&ID=20230843122) | [jpost.com](https://www.english-corpora.org/corona/x3.asp?node=&p=20&w10=coronavirus&w11=vaccination&r=) |  |  |  | during police activities in Isawiya. The apex, perhaps, was the large-scale **coronavirus** **vaccination** operation and the inclusion of Arab residents in support and benevolent programs, with food |
| [1959](https://www.english-corpora.org/corona/x4.asp?rs=&t=32804180&ID=20249636063) | [21-05-06 US](https://www.english-corpora.org/corona/x4.asp?rs=&t=32804180&ID=20249636063) | [nbcnews.com](https://www.english-corpora.org/corona/x3.asp?node=&p=20&w10=coronavirus&w11=vaccination&r=) |  |  |  | newswire the Press Trust of India. # Barriers are seen outside a Covid-19 **coronavirus** **vaccination** center after they were closed for three days.Punit Paranjpe / AFP - Getty Images # |
| [1960](https://www.english-corpora.org/corona/x4.asp?rs=&t=62899229&ID=20246420206) | [21-05-06 US](https://www.english-corpora.org/corona/x4.asp?rs=&t=62899229&ID=20246420206) | [ctpost.com](https://www.english-corpora.org/corona/x3.asp?node=&p=20&w10=coronavirus&w11=vaccination&r=) |  |  |  | deaths. # All state residents over age 16 have been eligible for a **coronavirus** **vaccination** since April 15. As of this week, more than 5.6 million doses of |
| [1961](https://www.english-corpora.org/corona/x4.asp?rs=&t=87160390&ID=20200643497) | [21-05-02 US](https://www.english-corpora.org/corona/x4.asp?rs=&t=87160390&ID=20200643497) | [The Motley Fool](https://www.english-corpora.org/corona/x3.asp?node=&p=20&w10=coronavirus&w11=vaccination&r=) |  |  |  | three other catalysts in its sails. # First, the U.S. and global **coronavirus** **vaccination** campaigns are underway. In the U.S., almost 38% of the adult population is |
| [1962](https://www.english-corpora.org/corona/x4.asp?rs=&t=87164398&ID=20214878675) | [21-05-03 US](https://www.english-corpora.org/corona/x4.asp?rs=&t=87164398&ID=20214878675) | [Fox News](https://www.english-corpora.org/corona/x3.asp?node=&p=20&w10=coronavirus&w11=vaccination&r=) |  |  |  | surprised that children as young as six years old are now being forced into **coronavirus** **vaccination** trials? In fact, a short time ago, news broke that starting next |
| [1963](https://www.english-corpora.org/corona/x4.asp?rs=&t=87165596&ID=20214646737) | [21-05-03 US](https://www.english-corpora.org/corona/x4.asp?rs=&t=87165596&ID=20214646737) | [The Washington Post on MSN.com](https://www.english-corpora.org/corona/x3.asp?node=&p=20&w10=coronavirus&w11=vaccination&r=) |  |  |  | will give hope to travelers from the United States and other countries with aggressive **coronavirus** **vaccination** programs who are eager to return to some of the globe's most popular destinations |
| [1964](https://www.english-corpora.org/corona/x4.asp?rs=&t=87165596&ID=20214648009) | [21-05-03 US](https://www.english-corpora.org/corona/x4.asp?rs=&t=87165596&ID=20214648009) | [The Washington Post on MSN.com](https://www.english-corpora.org/corona/x3.asp?node=&p=20&w10=coronavirus&w11=vaccination&r=) |  |  |  | businesses, schools and other entities from requiring people to provide documentation of their **coronavirus** **vaccination**. # " I think that's the evidence-based thing to do, " he |
| [1965](https://www.english-corpora.org/corona/x4.asp?rs=&t=87171856&ID=20227964309) | [21-05-04 US](https://www.english-corpora.org/corona/x4.asp?rs=&t=87171856&ID=20227964309) | [Fox Business](https://www.english-corpora.org/corona/x3.asp?node=&p=20&w10=coronavirus&w11=vaccination&r=) |  |  |  | on expectations of an economic recovery and strong company profits this year as large-scale **coronavirus** **vaccination** programs help people return to jobs and normal activities after more than a year of |
| [1966](https://www.english-corpora.org/corona/x4.asp?rs=&t=87173330&ID=20225270398) | [21-05-04 US](https://www.english-corpora.org/corona/x4.asp?rs=&t=87173330&ID=20225270398) | [The Guardian on MSN.com](https://www.english-corpora.org/corona/x3.asp?node=&p=20&w10=coronavirus&w11=vaccination&r=) |  |  |  | drawbridge pulled up behind them. We then need to add Australia's domestic **coronavirus** **vaccination** program running behind schedule. This is a story of bad luck and bad judgments |
| [1967](https://www.english-corpora.org/corona/x4.asp?rs=&t=87173894&ID=20229367411) | [21-05-04 US](https://www.english-corpora.org/corona/x4.asp?rs=&t=87173894&ID=20229367411) | [New York Times](https://www.english-corpora.org/corona/x3.asp?node=&p=20&w10=coronavirus&w11=vaccination&r=) |  |  |  | education. Most recently, it was instrumental in forming Covax, the global **coronavirus** **vaccination** program. For now, the foundation says little will change in how it is |
| [1968](https://www.english-corpora.org/corona/x4.asp?rs=&t=87177313&ID=20239841462) | [21-05-05 US](https://www.english-corpora.org/corona/x4.asp?rs=&t=87177313&ID=20239841462) | [The Boston Globe on MSN.com](https://www.english-corpora.org/corona/x3.asp?node=&p=20&w10=coronavirus&w11=vaccination&r=) |  |  |  | the statement reads. 87177313 # Massachusetts' **coronavirus** **vaccination** campaign has been among the most successful in the country, with 57.3 percent of |
| [1969](https://www.english-corpora.org/corona/x4.asp?rs=&t=87178842&ID=20241456529) | [21-05-05 US](https://www.english-corpora.org/corona/x4.asp?rs=&t=87178842&ID=20241456529) | [Reuters on MSN.com](https://www.english-corpora.org/corona/x3.asp?node=&p=20&w10=coronavirus&w11=vaccination&r=) |  |  |  | before he moved on to the next home on Tuesday. # The U.S. **coronavirus** **vaccination** campaign has reached a tipping point, with supply outstripping demand due to factors including |
| [1970](https://www.english-corpora.org/corona/x4.asp?rs=&t=87179283&ID=20243165968) | [21-05-05 US](https://www.english-corpora.org/corona/x4.asp?rs=&t=87179283&ID=20243165968) | [Yahoo](https://www.english-corpora.org/corona/x3.asp?node=&p=20&w10=coronavirus&w11=vaccination&r=) |  |  |  | it has seen " meaningful improvement " in bookings in March and April as **coronavirus** **vaccination** rates rise and more people feel confident about traveling again. # It was a |
| [1971](https://www.english-corpora.org/corona/x4.asp?rs=&t=87181277&ID=20243968001) | [21-05-05 US](https://www.english-corpora.org/corona/x4.asp?rs=&t=87181277&ID=20243968001) | [CNN](https://www.english-corpora.org/corona/x3.asp?node=&p=20&w10=coronavirus&w11=vaccination&r=) |  |  |  | doses # From CNN Health's Maggie Fox # The first nationwide study of **coronavirus** **vaccination**, done in Israel, shows Pfizer/BioNtech's vaccine works far better after two doses |
| [1972](https://www.english-corpora.org/corona/x4.asp?rs=&t=87182934&ID=20254459067) | [21-05-06 US](https://www.english-corpora.org/corona/x4.asp?rs=&t=87182934&ID=20254459067) | [CBS News](https://www.english-corpora.org/corona/x3.asp?node=&p=20&w10=coronavirus&w11=vaccination&r=) |  |  |  | is offering a free mini cheesecake through Memorial Day to anyone who shows their **coronavirus** **vaccination** card at their location in Downtown Brooklyn. Nathan's Famous hot dog at |
| [1973](https://www.english-corpora.org/corona/x4.asp?rs=&t=87185440&ID=20253992887) | [21-05-06 US](https://www.english-corpora.org/corona/x4.asp?rs=&t=87185440&ID=20253992887) | [Politico](https://www.english-corpora.org/corona/x3.asp?node=&p=20&w10=coronavirus&w11=vaccination&r=) |  |  |  | by Martin Finucane and Felice J. Freyer, Boston Globe: " Massachusetts' **coronavirus** **vaccination** campaign has been among the most successful in the country, with 57.3 percent of |
| [1974](https://www.english-corpora.org/corona/x4.asp?rs=&t=87187071&ID=20255620949) | [21-05-06 US](https://www.english-corpora.org/corona/x4.asp?rs=&t=87187071&ID=20255620949) | [SC Media](https://www.english-corpora.org/corona/x3.asp?node=&p=20&w10=coronavirus&w11=vaccination&r=) |  |  |  | intermittent lockdowns and endless government announcements, many countries are now rolling out their **coronavirus** **vaccination** programs. And with vaccinations either underway or about to begin, some countries have |
| [1976](https://www.english-corpora.org/corona/x4.asp?rs=&t=87189989&ID=20268738005) | [21-05-07 US](https://www.english-corpora.org/corona/x4.asp?rs=&t=87189989&ID=20268738005) | [Omaha.com](https://www.english-corpora.org/corona/x3.asp?node=&p=20&w10=coronavirus&w11=vaccination&r=) [(1)](https://www.english-corpora.org/corona/duplicates1.asp?xx=1975&n=1) |  |  |  | 23, 2021. Appointments are no longer necessary at any of the **coronavirus** **vaccination** sites run by New York City. New York City Mayor Bill de Blasio announced |
| [1979](https://www.english-corpora.org/corona/x4.asp?rs=&t=52711059&ID=20260666176) | [21-05-07 CA](https://www.english-corpora.org/corona/x4.asp?rs=&t=52711059&ID=20260666176) | [lfpress.com](https://www.english-corpora.org/corona/x3.asp?node=&p=20&w10=coronavirus&w11=vaccination&r=) [(2)](https://www.english-corpora.org/corona/duplicates1.asp?xx=1978&n=2) |  |  |  | photo a health worker prepares a dose of the AstraZeneca/Oxford vaccine at a **coronavirus** **vaccination** centre at at the Wanda Metropolitano stadium in Madrid on March 24, 2021. |
| [1980](https://www.english-corpora.org/corona/x4.asp?rs=&t=42976960&ID=20247610227) | [21-05-06 GB](https://www.english-corpora.org/corona/x4.asp?rs=&t=42976960&ID=20247610227) | [spectator.co.uk](https://www.english-corpora.org/corona/x3.asp?node=&p=20&w10=coronavirus&w11=vaccination&r=) |  |  |  | week, 29 per cent of the adult population had received both doses of **coronavirus** **vaccination**; 65 per cent the first dose. In the seven days up to the |
| [1981](https://www.english-corpora.org/corona/x4.asp?rs=&t=62889316&ID=20217378014) | [21-05-04 IE](https://www.english-corpora.org/corona/x4.asp?rs=&t=62889316&ID=20217378014) | [intellinews.com](https://www.english-corpora.org/corona/x3.asp?node=&p=20&w10=coronavirus&w11=vaccination&r=) |  |  |  | the trashed Turkish lira, rife unemployment, the pandemic and the acutely inadequate **coronavirus** **vaccination** programme in the country. # Another common feeling among the young is that when |
| [1982](https://www.english-corpora.org/corona/x4.asp?rs=&t=62892872&ID=20238270141) | [21-05-05 IE](https://www.english-corpora.org/corona/x4.asp?rs=&t=62892872&ID=20238270141) | [universitytimes.ie](https://www.english-corpora.org/corona/x3.asp?node=&p=20&w10=coronavirus&w11=vaccination&r=) |  |  |  | hired as paid vaccinators in centres across Ireland as the HSE speeds up the **coronavirus** **vaccination** programme. # The Irish Mirrorreported today that Ireland East Hospital Group has advertised positions |
| [1983](https://www.english-corpora.org/corona/x4.asp?rs=&t=72973914&ID=20217795226) | [21-05-04 AU](https://www.english-corpora.org/corona/x4.asp?rs=&t=72973914&ID=20217795226) | [miragenews.com](https://www.english-corpora.org/corona/x3.asp?node=&p=20&w10=coronavirus&w11=vaccination&r=) |  |  |  | Seven per cent of the public think it is not worth getting the second **coronavirus** **vaccination** as it doesn't really increase your level of protection. # But this belief |
| [1984](https://www.english-corpora.org/corona/x4.asp?rs=&t=62898769&ID=20251324443) | [21-05-06 IN](https://www.english-corpora.org/corona/x4.asp?rs=&t=62898769&ID=20251324443) | [ptinews.com](https://www.english-corpora.org/corona/x3.asp?node=&p=20&w10=coronavirus&w11=vaccination&r=) |  |  |  | made open for all senior citizens. # In order to ramp up the **coronavirus** **vaccination** drive in the country, the Indian government had last month announced a " liberalised |
| [1986](https://www.english-corpora.org/corona/x4.asp?rs=&t=72964882&ID=20185769191) | [21-05-01 IN](https://www.english-corpora.org/corona/x4.asp?rs=&t=72964882&ID=20185769191) | [scroll.in](https://www.english-corpora.org/corona/x3.asp?node=&p=20&w10=coronavirus&w11=vaccination&r=) [(1)](https://www.english-corpora.org/corona/duplicates1.asp?xx=1985&n=1) |  |  |  | to provide that. # Congress leader Rahul Gandhi on Thursday demanded free **coronavirus** **vaccination** for all Indians, a day after the Centre started registrations for the third phase |
| [1987](https://www.english-corpora.org/corona/x4.asp?rs=&t=72964890&ID=20185772050) | [21-05-01 IN](https://www.english-corpora.org/corona/x4.asp?rs=&t=72964890&ID=20185772050) | [timesnownews.com](https://www.english-corpora.org/corona/x3.asp?node=&p=20&w10=coronavirus&w11=vaccination&r=) |  |  |  | extended for one more week. From today, the third phase of the **coronavirus** **vaccination** drive has begun but a handful number of states have started the drive and other |
| [1988](https://www.english-corpora.org/corona/x4.asp?rs=&t=72965394&ID=20185853834) | [21-05-01 IN](https://www.english-corpora.org/corona/x4.asp?rs=&t=72965394&ID=20185853834) | [businesstoday.in](https://www.english-corpora.org/corona/x3.asp?node=&p=20&w10=coronavirus&w11=vaccination&r=) |  |  |  | 72965394 # These states are being extremely judicious with their vaccination drives # **Coronavirus** **vaccination** for all those between 18 and 45 years of age has started only in 6 |
| [1989](https://www.english-corpora.org/corona/x4.asp?rs=&t=72965394&ID=20185854053) | [21-05-01 IN](https://www.english-corpora.org/corona/x4.asp?rs=&t=72965394&ID=20185854053) | [businesstoday.in](https://www.english-corpora.org/corona/x3.asp?node=&p=20&w10=coronavirus&w11=vaccination&r=) |  |  |  | forward successfully, " UP Chief Minister Yogi Adityanath said. # GUJARAT # **Coronavirus** **vaccination** drive for all those above 18 years of age has begun in the 10 worst |
| [1990](https://www.english-corpora.org/corona/x4.asp?rs=&t=72979097&ID=20238922325) | [21-05-05 IN](https://www.english-corpora.org/corona/x4.asp?rs=&t=72979097&ID=20238922325) | [timesnownews.com](https://www.english-corpora.org/corona/x3.asp?node=&p=20&w10=coronavirus&w11=vaccination&r=) |  |  |  | extended for one more week. From today, the third phase of the **coronavirus** **vaccination** drive has begun but a handful number of states have started the drive and other |
| [1991](https://www.english-corpora.org/corona/x4.asp?rs=&t=72979209&ID=20232857829) | [21-05-05 IN](https://www.english-corpora.org/corona/x4.asp?rs=&t=72979209&ID=20232857829) | [scroll.in](https://www.english-corpora.org/corona/x3.asp?node=&p=20&w10=coronavirus&w11=vaccination&r=) |  |  |  | Adityanath says journalists and their families would receive special consideration and priority in the **coronavirus** **vaccination** and separate inoculation centres would be set up for them, reports The Indian Express |
| [1992](https://www.english-corpora.org/corona/x4.asp?rs=&t=72982560&ID=20249075953) | [21-05-06 IN](https://www.english-corpora.org/corona/x4.asp?rs=&t=72982560&ID=20249075953) | [timesnownews.com](https://www.english-corpora.org/corona/x3.asp?node=&p=20&w10=coronavirus&w11=vaccination&r=) |  |  |  | third phase of the **coronavirus** **vaccination** drive has begun partially in the nation as the county is facing a supply deficit |
| [1993](https://www.english-corpora.org/corona/x4.asp?rs=&t=72982560&ID=20249076032) | [21-05-06 IN](https://www.english-corpora.org/corona/x4.asp?rs=&t=72982560&ID=20249076032) | [timesnownews.com](https://www.english-corpora.org/corona/x3.asp?node=&p=20&w10=coronavirus&w11=vaccination&r=) |  |  |  | and was about to arrive before the starting of the third phase of the **coronavirus** **vaccination** drive i.e. May 1. Currently, we have received only 1.5 lakh vaccine doses |
| [1994](https://www.english-corpora.org/corona/x4.asp?rs=&t=52698340&ID=20206326644) | [21-05-03 PK](https://www.english-corpora.org/corona/x4.asp?rs=&t=52698340&ID=20206326644) | [suchtv.pk](https://www.english-corpora.org/corona/x3.asp?node=&p=20&w10=coronavirus&w11=vaccination&r=) |  |  |  | . # It is noteworthy that over four million people have registered themselves for **coronavirus** **vaccination** across Pakistan. Overall 40,33,170 people have got them registered including 24,49,032 male and 15,84,140 |
| [1995](https://www.english-corpora.org/corona/x4.asp?rs=&t=52698340&ID=20206326701) | [21-05-03 PK](https://www.english-corpora.org/corona/x4.asp?rs=&t=52698340&ID=20206326701) | [suchtv.pk](https://www.english-corpora.org/corona/x3.asp?node=&p=20&w10=coronavirus&w11=vaccination&r=) |  |  |  | have opted for vaccination. In Sindh 7,70,101 persons have got them registered for **coronavirus** **vaccination**, while in Khyber Pakhtunkhwa 4,92,295 people have been in vaccination list. |
| [1996](https://www.english-corpora.org/corona/x4.asp?rs=&t=52701826&ID=20216772384) | [21-05-04 PK](https://www.english-corpora.org/corona/x4.asp?rs=&t=52701826&ID=20216772384) | [samaa.tv](https://www.english-corpora.org/corona/x3.asp?node=&p=20&w10=coronavirus&w11=vaccination&r=) |  |  |  | . Follow SAMAA English on Facebook, Twitter, and Instagram. # The **coronavirus** **vaccination** drive for Pakistanis above 18 years will start after Eid if there are no problems |
| [1997](https://www.english-corpora.org/corona/x4.asp?rs=&t=52705932&ID=20231644580) | [21-05-05 PK](https://www.english-corpora.org/corona/x4.asp?rs=&t=52705932&ID=20231644580) | [samaa.tv](https://www.english-corpora.org/corona/x3.asp?node=&p=20&w10=coronavirus&w11=vaccination&r=) |  |  |  | . According to Sultan, people below this age can't get vaccinated. **Coronavirus** **vaccination** in the country began on January 1, 2021 but the process to procure the |
| [1998](https://www.english-corpora.org/corona/x4.asp?rs=&t=52711722&ID=20258328173) | [21-05-07 PH](https://www.english-corpora.org/corona/x4.asp?rs=&t=52711722&ID=20258328173) | [entertainment.inquirer.net](https://www.english-corpora.org/corona/x3.asp?node=&p=20&w10=coronavirus&w11=vaccination&r=) |  |  |  | measures will be required. It is possible theaters will mandate proof of a **coronavirus** **vaccination** for audience members, New York budget director Robert Mujica said. # " Will |
| [1999](https://www.english-corpora.org/corona/x4.asp?rs=&t=72973482&ID=20210735442) | [21-05-03 PH](https://www.english-corpora.org/corona/x4.asp?rs=&t=72973482&ID=20210735442) | [bworldonline.com](https://www.english-corpora.org/corona/x3.asp?node=&p=20&w10=coronavirus&w11=vaccination&r=) |  |  |  | of the cluster of essential workers who are next in line as priority for **coronavirus** **vaccination**. # Tourism Secretary Bernadette Romulo-Puyat, who has been lobbying for the inoculation of |
| [2000](https://www.english-corpora.org/corona/x4.asp?rs=&t=52691723&ID=20180350198) | [21-05-01 NG](https://www.english-corpora.org/corona/x4.asp?rs=&t=52691723&ID=20180350198) | [leadership.ng](https://www.english-corpora.org/corona/x3.asp?node=&p=20&w10=coronavirus&w11=vaccination&r=) |  |  |  | a sum of? 100 million as part of its humanitarian initiative to support **coronavirus** **vaccination** campaigns in Africa. # The Commissioner for Crisis Management, Janez Lenarcic disclosed that |

There have been also used and analyzed different artucles from Internet additionally, such as:

1. 'My body, my choice' protesters say of COVID-19 vaccine mandate at Henry Ford Health System: Christina Hall and Emma Stein, Detroit Free Press  7/17/2021: https://www.msn.com/en-us/news/us/my-body-my-choice-protesters-say-of-covid-19-vaccine-mandate-at-henry-ford-health-system/ar-AAMgYO1
2. [2. ](https://web.whatsapp.com/send?text=http%3a%2f%2fa.msn.com%2f01%2fen-us%2fAAMgYO1%3focid%3dsw) Hundreds of thousands protest discriminatory COVID-19 vaccine passports in France: <https://multidimensionalocean.wordpress.com/2021/08/24/hundreds-of-thousands-protest-discriminatory-covid-19-vaccine-passports-in-france/>
3. [3. ](mailto:?subject=%27My%20body%2c%20my%20choice%27%20protesters%20say%20of%20COVID-19%20vaccine%20mandate%20at%20Henry%20Ford%20Health%20System&body=I%20thought%20you%20would%20be%20interested%20in%20this%20story%20I%20found%20on%20MSN%3a%20%27My%20body%2c%20my%20choice%27%20protesters%20say%20of%20COVID-19%20vaccine%20mandate%20at%20Henry%20Ford%20Health%20System%20http%3a%2f%2fa.msn.com%2f01%2fen-us%2fAAMgYO1%3focid%3dse) Citizens all over the world are rising up against vaccine passports, lockdowns

https://sociable.co/government-and-policy/citizens-against-vaccine-passports-lockdowns-medical-apartheid/

4. Anti-vax groups rack up victories against Covid-19 push

<https://www.politico.com/news/2021/06/14/anti-vax-groups-covid-victories-493482>

5. A Hospital Finds an Unlikely Group Opposing Vaccination: Its Workers

<https://www.nytimes.com/2021/08/22/nyregion/staten-island-covid-vaccine-workers.html>

6. Dennis Chong and James N. Druckman. FRAMING THEORY file:///C:/Users/User/Desktop/%D0%9D%D0%90%D0%A3%D0%9A%D0%90/%D0%A1%D0%A2%D0%90%D0%A2%D0%AC%D0%98/2022/Scopus.%20Peter%20Furco's%20suggestion/Literature/Chong%20Druckman%20Annual%20Review%202007.pdf
7. COVID-19 vaccine development differs from others in past
8. <https://www.wtkr.com/homepage-showcase/covid-19-vaccine-development-differs-from-others-in-past>
9. [Issa Kohler-Hausmann](http://sociology.as.nyu.edu/object/soc.gradstudents#k). Discrimination: <https://www.oxfordbibliographies.com/view/document/obo-9780199756384/obo-9780199756384-0013.xml#:~:text=Introduction.%20Discrimination%20is%20an%20action%20or%20practice%20that,as%20an%20explanation%20for%20other%20observed%20social%20phenomenon>**.**
10. Tajfel H., (1970)Experiments in Intergroup Discrimination: http://www.holah.karoo.net/tajfestudy.htm

And many others…
